# Abstracts from the International Society for Therapeutic Ultrasound Conference 2017: Nanjing, China. 31 May - 02 June 2017

**DOI:** 10.1186/s40349-018-0110-x

**Published:** 2018-05-15

## Oral Presentations

### O1 Neuroinflammation after disrupting the blood brain barrier with pulsed focused ultrasound and microbubbles imaged by 18F-DPA-714 PET and MRI

#### Zsofia I. Kovacs, Georgios Z. Papadakis, Tsang-Wei Tu, Sanhita Sinharay, William C. Reid, Bobbi Lewis, Dima A. Hammoud, Joseph A. Frank

##### National Institutes of Health, Bethesda, Maryland, United States

###### **Correspondence:** Zsofia I. Kovacs

**OBJECTIVES** Blood brain barrier (BBB) disruption with MR-guided pulsed focused ultrasound (pFUS) and microbubbles (MB) has been advocated as a noninvasive adjuvant treatment for malignancies and neurodegenerative diseases. A sterile inflammatory reaction has been recently described in the brain as a result of pFUS+MB (Kovacs et al. 2016). However, one potential issue of weekly pFUS+MB treatments is the lack of data on the long-term effects on inflammation. The purpose of this study was to evaluate the effects of multiple weekly courses of pFUS+MB exposures in the rat brain using micro-Positron Emmision Tomography (PET) and 18F-DPA-714, a marker of translocator protein (TSPO) upregulation/microglial activation and an indication of neuroinflammation.

**METHODS** Female rats were assigned to three different groups based on the number of weekly pFUS+MB: Group 1: pFUS+MB x1, PET scans performed 24 hours later (n=6) ; Group 2: pFUS+MB x2 with PET scans performed within 10 days after 2nd sonication (n=5) ; and Group 3: pFUS+MB x6 with PET scans performed 7-9 days later (n=5). The left striatum (str) and right hippocampus (hc) were targeted in all animals. 100 μl of MB (OptisonTM, GE Healthcare, Little Chalfont, UK) was administered intravenously over 1 minute starting 30 secs before pFUS. Acoustic energy was delivered to the brain using “BBB configuration function” based on algorithm reported (O’Reilly et al. 2012) to determine optimal acoustic pressure for BBB opening *via* 1.5*f*_0_ and 2.5*f*_0_ ultra harmonic acoustic emission detection for every single pulse (9 focal points, 120 sec/9 focal points – striatum, 120 sec/4 focal points – hippocampus) using an 825 kHz hydrophone with a single-element spherical FUS transducer (center frequency: 589.636 kHz; focal number: 0.8; aperture: 7.5 cm; RK-100, FUS Instruments, Toronto, Ontario, Canada). T2* map were created from multiecho gradient echo sequence at 3T (Achieva, Philips Healthcare, Andover, MA) through the rat brain with TE=7 msec, echo train length 5 and echo spacing 7 and Tr=1500 msec. T2* maps were created by fitting signal intensity at each voxel to a single exponential fit with in-house software and histogram analysis was performed on volume of interests (VOI). Static microPET/CT scans emission data was acquired 30-60 min after injection of 18F-DPA-714. VOIs were drawn in the targeted areas and uptake was compared to the contralateral unaffected side. Uptake values were normalized to cerebellum.

**RESULTS** 18F-DPA-714 uptake was increased at the sonication sites in all locations (Fig. 1). The ratio of the percent increase in SUV between pFUS+MB treated striatum and hippocampus to contralateral side is depicted in graph (mean+/-SEM) clearly showing large *increase* in uptake for both regions compared to normal brain. The neuroinflammatory changes persisted for at least 14 days after 2 weekly sonications. The coefficient of variation for PET scans was <10%. This corresponded to Iba1 activation visible on histology. Figure 2 contains normalized histograms from VOI for Group 2 and Group 3 rats derived from pFUS+MB treated (ipsilateral) and contralateral brain that shows a shift to lower T2* values for sonicated regions.

**Fig. 1 (abstract O1).**
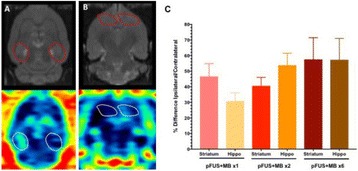
18F­DPA­714 PET scans (co­registered to MRI template) in two rats: A: Hippocampus uptake 24 hours after one sonication. B: Striatum uptake 10 days after six weekly sonications. C: Graph of % difference between sonicated ipsilateral and contralateral striatum and hippocampus regions for Groups 1, 2 and 3

**Fig. 2 (abstract O1).**
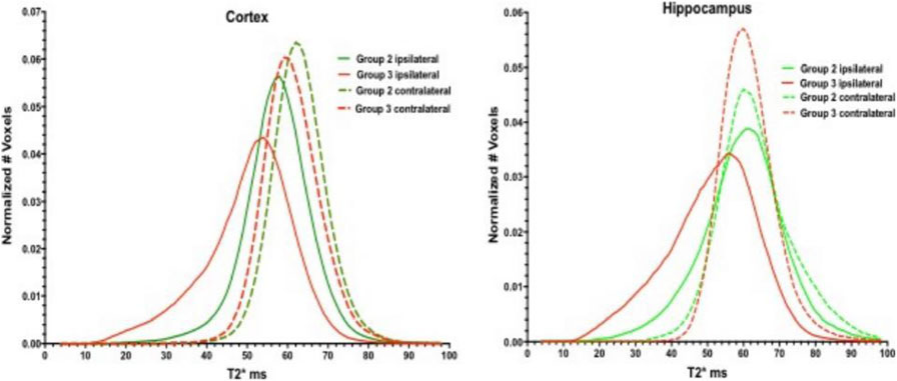
Mean normalized histograms derived from VOI from pFUS+MB treated cortex/striatum and hippocampus (ipsilateral) compared to contralateral brain forGroup 2 and 3 cohorts of rats. There is clear shift to lower T2* values for sonicated regions for 2x v

**CONCLUSIONS** Rats receiving pFUS+MB to open the BBB showed a clear upregulation of TSPO expression consistent with microglial activation/neuroinflammation, even after one sonication session. Histograms derived from T2* maps MRI clearly shows that sonication with BBB algorithm results in left shift in T2* values that would be consistent with hypointense voxels on T2*w MRI and abnormatilies on histolology. These preliminary results contradict current assumptions that the effects of pFUS+MB are confined primarily to the endothelium and vessel wall. Further assessment of the long-term effects of pFUS+MB is necessary before this approach can be widely implemented in clinical trials.

**References**

Kovacs, Z. I., et al. (2017). "Disrupting the blood-brain barrier by focused ultrasound induces sterile inflammation." Proc Natl Acad Sci U S A 114(1): E75-E84.

O'Reilly, M. A. and K. Hynynen (2012). "Blood-brain barrier: real-time feedback-controlled focused ultrasound disruption by using an acoustic emissions-based controller." Radiology 263(1): 96-106.

### O2 Long term effects of pulsed focused ultrasound and microbubbles detected by multivariate imaging modalities

#### Zsofia I. Kovacs, Tsang-Wei Tu, Georgios Z. Papadakis, William C. Reid, Dima A. Hammoud, Joseph A. Frank

##### National Institutes of Health, Bethesda, Maryland, United States

###### **Correspondence:** Zsofia I. Kovacs

**OBJECTIVES** Blood brain barrier (BBB) opening by Guided Pulsed Focused Ultrasound (pFUS) and microbubbles (MB) is a non-invasive treatment of various central nervous system diseases. However, the potential adverse effects of repeated pFUS+MB exposure have not been thoroughly elucidated and may limit clinical translation. To date MRI scans of repeated BBB opening by pFUS+MB have been achieved without hemorrhage, edema and behavioral changes in non-human primates (Arvanitis, *et al*. 2016; Downs, *et al*. 2015). By incorporating detailed multimodal imaging, we characterized the long term effects of single or repeated pFUS+MB in the rat brain. The purpose of the study is to reveal the morphological and pathological changes following repeated BBB opening in the striatum and hippocampus as monitored by 3T and 9.4T MRI, FDG-positron emission tomography (PET) and histology over 13 weeks.

**METHODS** pFUS+MB (Optison^TM^, GE Healthcare, Little Chalfont, UK) 0.3 – 0.5 MPa, 10 ms burst length, 1 % duty cycle, 9 focal points, 120 sec/9 focal points – striatum, 120 sec/4 focal points – hippocampus, 589.636 kHz; focal number: 0.8; aperture: 7.5 cm; (FUS Instruments, Toronto, Ontario, Canada) was targeted in female rats (n=6/group) either once or six weekly to the striatum and the contralateral hippocampus. 100 μL of MB were administered intravenously over 1 minute starting 30 secs before pFUS. Rats received 3 daily doses of 300mg/kg 5-Bromo-2′-deoxy-uridine (BrdU, Sigma-Aldrich, St. Louis, MO) intraperitoneally before sonication to label proliferating cells *in vivo*. T2, T2* and Gd-enhanced T1-weighted images were obtained by 3.0T MRI (Achieva, Philips Healthcare, Andover, MA), T2, T2* and diffusion tensor imaging (DTI) were performed by 9.4T MRI (Bruker, Billerica, MA). Parameters for DTI: 3D spin echo EPI; TR/TE 700 ms/37 msec; b-value 800 sec/mm^2^ with 17 encoding directions; voxel size 200 μm (isotropic). Fractional anisotropy (FA) and the asymmetry of magnetization transfer ratio (MTRasym) were derived for mapping structural injury and glucose levels. Rats received ~1.1 mCi of 18F-FDG *via* intravenously to quantitate glucose uptake by PET/CT (Inveon, Siemens, Munich, Germany). PET emission data was acquired for 60 min. Dynamic images were reconstructed and image analyses was performed (PMOD Technologies Ltd., Zurich, Switzerland). Animals were euthanized 7 or 13 weeks after the first pFUS treatment. Histological evaluation of brain and tracking of BrdU tagged cells was performed.

**RESULTS** Gd-enhanced T1-weighted images after each sonication demonstrated BBB disruption in the striatum and the hippocampus at 3T. Gd T1 enhancement, T2 and T2* abnormalities were not seen in the brain 1-day post pFUS+MB at 9.4T MRI. Hypointense regions appeared on T2* MRI 2 weeks post pFUS+MB consistent with development of microhemorrhages within the parenchyma. White matter fiber structure- and gray matter-abnormalities on DTI MRI were detected in regions with the T2* abnormalities suggestive of increased astrogliosis and transient axonal damage. MRI findings following pFUS+MB x6 demonstrated more pronounced evidence of damage within the parenchyma and atrophy. FDG-PET did not show differences between sonicated and contralateral cortex or hippocampus at any time point. BrdU showed evidence of increased neurogenesis in pFUS+MB treated regions.

**CONCLUSIONS** The long term effects of pFUS+MB exposures in rats revealed that both single and repeated pFUS+MB cause structural injury at the location of sonication up to 13 weeks post treatment based on advanced imaging techniques. Histological evidence showed that associated with the pathological changes observed by MRI, there was evidence of neuronal damage and loss, neurogenesis and activated microglia. These results suggest the importance of long term monitor of the brain following low intensity pFUS+MB before its clinical translation.

**References**

Arvanitis, C. D., et al. (2016). "Cavitation-enhanced nonthermal ablation in deep brain targets: feasibility in a large animal model." J Neurosurg 124(5): 1450-1459.

Downs, M. E., et al. (2015). "Long-Term Safety of Repeated Blood-Brain Barrier Opening *via* Focused Ultrasound with Microbubbles in Non-Human Primates Performing a Cognitive Task." PLoS One 10(5): e0125911.

### O3 Characterization of different microbubbles in assisting focused ultrasound-induced blood-brain barrier opening

#### Sheng-Kai Wu^1^, Po-Chun Chu^2, 3^, Wen Yen Chai^3, 4^, Shih-Tsung Kang^5^, Chih-Hung Tsai^3^, Ching-Hsiang Fan^5^, Chih-Kuang Yeh^5^, Hao-Li Liu^3^

##### ^1^Institute of Biomedical Engineering, National Taiwan University, Taipei, Taiwan; ^2^Department of Research and Development, NaviFUS corp, Taipei, Taiwan; ^3^Department of Electrical Engineering, Chang-Gung University, Taoyuan City, Taiwan; ^4^Department of Diagnostic Radiology and Intervention, Chang-Gung Memorial Hospital, Taoyuan City, Taiwan; ^5^Department of Biomedical Engineering and Environmental Sciences, National Tsing Hua University, Hsinchu, Taiwan

###### **Correspondence:** Sheng-Kai Wu

**This abstract is not included as it has already been published:**

Wu S-K, Chu P-C, Chai WY, Kang S-T, Tsai C-H, Fan C-H, Yeh C-K, Liu H-L. Characterization of Different Microbubbles in Assisting Focused Ultrasound-Induced Blood-Brain Barrier Opening. Sci Rep. 2017; 7. Available from: http://www.nature.com/articles/srep46689 doi:10.1038/srep46689

### O4 Development of an A-Synuclein (SNCA)-based mouse model for Parkinson's disease by ultrasound-guided CNS delivery

#### Chung-Yin Lin^1^, Yu-Chien Lin^2^, Hao-Li Liu^2^

##### ^1^Institute for Radiological Research, Chang Gung University, Taoyuan City, Taiwan; ^2^Department of Electrical Engineering, Chang Gung University, Taoyuan City, Taiwan

###### **Correspondence:** Chung-Yin Lin

**OBJECTIVES** Parkinson’s disease (PD) is the second most common neurodegenerative disease characterized by the progressive degeneration of dopaminergic neurons in the substantia nigra (SN) and the presence of α-synuclein-containing inclusion bodies in the cytoplasm of neurons. In this study, we propose to develop a novel asynuclein over-expression PD mouse model *via* ultrasound-guided CNS delivery of SNCA gene.

**METHODS** A plasmid encoding both the green fluorescent protein (GFP) gene and the SNCA gene was prepared. A gene-liposome system, in which the liposomes are designed to carry SNCA plasmid DNA, forms liposomal-plasmid DNA (LpDNA) complex. Ultrasound (US) exposure used the SonoPore KTAC-4000 to induce BBB opening (1.0-MHz, voltage = 85 V, burst length = 10 ms, 10% duty cycle, and 3-min sonication duration). The longitudinal expression of GFP was quantitated *via* an *in vivo* imaging system (IVIS). The SNCA gene expression level was confirmed *via* immunoblotting, and histological staining will be used to identify transfected cells *via* fluorescent microscopy. Dopamine and metabolic DOPAC protein levels in the brain were determined *via* HPLC.

**RESULTS** With longitudinal observation of IVIS monitoring, animals with US treatment showed significant promotion of LpDNA release into the SN and demonstrated enhanced expression of genes upon sonication with US-BBB opening, while both the GFP and α-synuclein protein expression were successfully measured *via* Western blotting. Immunoblotting and histological staining confirmed the expression of reporter genes in neuronal cells.

**CONCLUSIONS** This study suggests that IV administration of LpDNA in combination with US-BBB opening can provide effective gene delivery and expression in the SN, demonstrating the potential to achieve non-invasive and targeted gene delivery for α-synuclein over-expression PD mouse model.

### O5 Experimental and numerical assesment of Doxorubicin pharmacokinetics in brain metastasis from breast cancer after focused ultrasound-induced blood-brain/blood-tumor barrier disruption

#### Costas Arvanitis^1,4^, Vasileios Askoxylakis^2^, Yutong Guo^1^, Jonas Kloepper^2^, Meenal Datta^2^, Miguel Bernabeu^3^, Dai Fukumura^2^, Nathan McDannold^5^, Rakesh Jain^2^

##### ^1^Mechanical Engineering, Georgia Institute of Technology, Atlanta, Georgia, USA; ^2^Radiation Oncology, Massachusetts General Hospital, Harvard Medical School, Boston, Massachusetts, USA; ^3^Centre for Medical Informatics, University of Edinburgh, Edinburgh, UK; ^4^Biomedical Engineering, Georgia Institute of Technology, Atlanta, Georgia, USA; ^5^Radiology, Brigham and Women's Hospital, Harvard Medical School, Boston, Massachusetts, USA

###### **Correspondence:** Costas Arvanitis

**OBJECTIVES** Blood brain and blood tumor barriers (BBB and BTB) constitute a major obstacle to the transport of therapeutics in brain tumors. Focused ultrasound (FUS), when combined with circulating microbubbles, provides a noninvasive method to locally and transiently disrupt the BBB/BTB. While several studies have demonstrated its potential for targeted drug delivery, there is a lack of fundamental understanding of the impact of this method on the pharmacokinetics of anticancer agents in the brain microenvironment. In this study, we examine the impact of FUS-induced BBB/BTB disruption on the transport of the chemotherapeutic agent doxorubicin (Dox) in a model of breast cancer brain metastases using intravital microscopy and drug transport mathematical modeling.

**METHODS** Human HER2-amplified and estrogen dependent BT474 breast cancer cells, genetically modified to express green fluorescent protein, were stereotactically implanted in the brain of mice with cranial windows. At a tumor size of ~20-40mm^3^, BBB/BTB disruption was performed using FUS exposures by a custom built portable FUS system and concurrent i.v. administration of microbubbles. To cover the entire tumor and its periphery four non-overlapping sonications were performed. Shortly after sonication, the auto-fluorescent chemotherapeutic agent Dox (7.5mg/kg) was administered i.v. The pharmacokinetics of Dox, including intratumoral uptake and clearance, were measured for 15 minutes using intravital multiphoton microscopy. The Dox cellular kinetics were also determined. For mathematical analysis, we developed a numerical model combining the Navier-Stokes and Brinkman equations for flow modeling in the tumor vasculature and interstitial space, coupled with a convection-diffusion-reaction model of drug transport based on Michaelis-Menten kinetics. The model parameters (vessel permeability, interstitium porosity, rate of cellular transmembrane transport, tissue hydraulic conductivity, and pressure difference driving flow across the vessel wall and interstitium) were inferred, using a numerical optimization procedure, from the experimentally determined Dox kinetics under the different conditions tested. Finally, based on the fitted values, a sensitivity analysis was performed and the most important parameters that affect the Dox transport and cellular uptake in the tumor interstitium were determined.

**RESULTS** Multiphoton microscopy revealed up to one order of magnitude higher Dox extravasation after FUS- BBB/BTB disruption as compared to control. In addition, a five-fold increase of Dox penetration was found after FUS- BBB/BTB disruption compared to control (>100μm vs. <20μm, based on Dox penetration regression). The numerical model indicated that only the vessel diffusion coefficient (4.71 ± 1.7μm^2^/s Vs 0.41 ± 0.1μm^2^/s, a tenfold increase) and the pressure difference (4.3 ± 0.45mmHg vs 2.9 ± 0.32mmHg) were significantly different between the FUS- BBB/BTB disruption and the control. While the increase in vessel diffusion coefficient was anticipated, the increase in pressure difference, which lead to a system’s Peclet number greater than one (Pe>1), suggested a fundamental change in the interstitial transport mechanism. Assessment of the temporal evolution of the drug concentration in the interstitial space in the experimental data verified that the transport after FUS-BBB/BTB disruption was convection dominated. Single cell analysis revealed significant Dox uptake by endothelial cells (EC), suggesting that microbubble vibrations can lead to significant changes in cellular transmembrane transport. Interestingly, in the FUS treated animals’ stroma cells (SC) appeared to take-up the drug at higher rate than the endothelial cells (uptake slope 0.44 vs 0.93 in EC and SC respectively). These differences in the uptake curves led to significant changes in the rate of cellular transmembrane transport in the numerical model during parameter fit. Finally, sensitivity analysis showed that FUS-BBB/BTB disruption makes drug uptake more sensitive to all the parameters of the system, suggesting that the main barrier to drug transport has been overcome. Interestingly, the BBB/BTB permeability remains an important parameter of the system even after FUS, indicating that further disruption may be beneficial.

**CONCLUSIONS** The *in vivo* and in silico data demonstrate significant changes in the tumor microenvironment after FUS-BBB/BTB disruption. The most notable changes included: i) increase in BBB/BTB permeability, ii) transition to convection dominated drug transport, and iii) increased cellular transmembrane transport in endothelial cells and stroma cells. Sensitivity analysis showed that the system has become more amenable to interventions, suggesting that FUS can lead to the development of new therapeutic strategies to treat brain tumors.

### O6 Acoustic emissions during blood-brain barrier disruption with focused ultrasound and real-time feedback control under infusion administration of microbubbles – feasibility study in rodent model

#### Chenchen Bing^1^, Debra Szczepanski^1^, Imalka Munaweera^1^, Yu Hong^1^, Ian Corbin^2^, Rajiv Chopra^1,2^

##### ^1^Radiology, UT Southwestern Medical Center, Dallas, Texas, USA; ^2^Advanced Imaging Research Center, UT Southwestern Medical Center, Dallas, Texas, USA

###### **Correspondence:** Chenchen Bing

**OBJECTIVES** Glioblastoma multiforme (GBM) is the most lethal primary brain tumor, with aggressive and fatal progression of disease inevitable. During the earlyinfiltration of GBM cells into normal brain regions surrounding the primary tumor, this peritumoral environment has an intact blood-brain barrier (BBB) which inhibitsthe delivery of therapeutic agents. BBB disruption with focused ultrasound could be used to achieve peritumoral delivery of therapeutic agents to tackle infiltrative cancerprogression. However, variability in vasculature structure and function around a tumor leads to heterogeneous perfusion of microbubbles which can impact the acousticthresholds required for BBB disruption. A more reliable means of BBB disruption is desired. Prior studies suggest that by integrating an acoustic emission detector and afeedback control algorithm to evaluate the intensity of acoustic emission at sub-/ultra-harmonics, more controlled and consistent BBB opening can be achieved. The goalof this study was to evaluate the acoustic emissions and BBB opening in rodents and validate the feasibility of real-time feedback control under intravenous infusionadministration of Optison microbubbles.

**METHODS** A custom-built focused ultrasound transducer with a central frequency of *f*_0_ = 0.5MHz was attached to a stereotactic system and used to deliver ultrasoundenergy into the target brain region. One piezocomposite hydrophone with resonant frequency at 0.75MHz was built to acquire the signals emitted from stimulatedmicrobubbles. A feedback control algorithm was implemented in LabVIEW to quantify the area under curve (AUC) within sub-/ultra-harmonic bands during theultrasound exposure and to adjust the focal pressure accordingly based on the difference between current AUC and a desired threshold. Initial *in vitro* tests wereperformed in which microbubble was infused into a single tube (0.08 mL/min, tubing I.D. = 1mm), and the AUC was quantified at 0.5*f*_0_, 1.5*f*_0_ and 2.5*f*_0_ as a function offocal pressure and microbubble concentration. Due to the significant response detected at 1.5*f*_0_ (0.75MHz), this specific frequency band was used for controlling in thefollowing experiment. The ability to maintain acoustic emissions at a target AUC level was also evaluate *in vitro*. Next an in-vivo study was performed in a rat model toevaluate the acoustic emissions and the feasibility of real-time control of the AUC at a target level. Acoustic emissions from a bolus injection and continuous infusionwere evaluated. For bolus injection, a fixed pressure level of 0.54 MPa was applied, while for infusion experiment, the feedback control was used to control the AUC atvarious levels. Evans blue dye was used as an indicator of BBB opening.

**RESULTS** A minimum transmit focal pressure of 0.33 - 0.41 MPa was required to trigger the bubble activity in the sub/ultra-harmonic bands *in vitro*, with the greatestchanges occurring at 0.75 MHz (1.5*f*_0_) (Fig. 1B). With varying concentration of microbubbles, the feedback control algorithm could adjust the focal pressure accordinglyto achieve a consistent harmonic emission (Fig. 1C). By observing the in-vivo AUC response of bolus injection used previously to successfully open the BBB, a thresholdAUC value was selected at 1.5*f*_0_ and tested with a continuous infusion of microbubbles. Successful maintenance of the AUC at different target value was achieved invivo at multiple locations in the brain, and BBB opening was confirmed as leakage of Evans Blue at the target locations (Fig. 1D).

**Fig. 1 (abstract O6).**
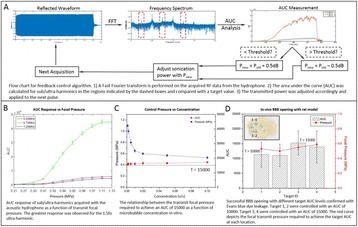
See text for description

**CONCLUSIONS** In this study, a stereotactic BBB opening system was extended to incorporate a feedback control algorithm based on the sub-/ultra-harmonic emissionsfrom microbubbles. The AUC responses were characterized and stable feedback control was demonstrated both in-vitro and in-vivo. Using infusion injection instead ofbolus injection, combined with the real-time feedback control system, a more reliable BBB opening can be achieved across a larger brain region for applications focusedon drug delivery to peritumoral regions.

### O7 Unilateral focused ultrasound-induced blood-brain barrier opening redistributes hyperphosphorylated Tau in an Alzheimer's mouse model

#### Maria Eleni Karakatsani^1^, Tara Kugelman^1^, Shutao Wang^1^, Karen Duff^3^, Elisa E. Konofagou^1,2^

##### ^1^Biomedical Engineering, Columbia University, New York, New York, USA; ^2^Radiology, Columbia University, New York, New York, USA; ^3^Pathology and Cell Biology, Columbia University, New York, New York, USA

###### **Correspondence:** Maria Eleni Karakatsani

**OBJECTIVES** Focused ultrasound has been shown to interact with Alzheimer’s pathology and particularly to trigger a mechanism that results in the reduction of the amyloid plaque load. However, a less studied interaction is that of ultrasound with the tangle formation that has been implicated in the cognitive decline of Alzheimer’s patients. Tau pathology can be characterized by increased density of the hyperphosphorylated tau protein that results in tangle formation. At the early stages of Alzheimer’s disease, tau protein can be localized primarily in the axons while in late pathology, somatodendritic tau is more pronounced. With the current study we investigate the interaction of focused ultrasound-induced blood-brain barrier opening with the tau distribution. Moreover, the unilateral sonication of the transgenic brain provides a unique opportunity to explore potential bilateral effects.

**METHODS** For this study the initial cohort included 10 mice of the rTg4510 line (3.5 months old), 5 of which were randomly assigned to the control group that did not receive any sonication and 5 to the treatment group. The treatment group received a double sonication covering almost the entire hippocampal region once per week for 4 consecutive weeks. The day after the last sonication the mice were sacrificed. The brains were sectioned and counterstained for tau protein (AT8) as well as microglia activation (CD68). The images were acquired by means of confocal microscopy over a z-series to account for depth differences and enabling co-visualization of the tau protein and microglia distribution. A custom algorithm was constructed to quantify the number of cells and the axonal distribution of the tau-marker. Background noise was automatically removed by color-based segmentation using k-means clustering and the cells were detected by the Hough transform. The axons were quantified based on their length marked by the tau protein. The same brain slices were utilized to quantify the hippocampal density of the CD68 marker by intensity-based quantification.

**RESULTS** Figure 1 shows two representative examples of the control and the treatment group. Following the hippocampal formation of the control brain, it can be observed that both somatodendritic and axonal tau (red) are evident. In particular, the tau marker engulfs the cell bodies and the entire in-plane length of the axons can be detected. Although the cell bodies affected by tau protein are also evident in the animals that received four sonications, the axonal tau was less pronounced. More specifically, the axonal distribution of the tau protein was not continuous. Differences across hemispheres were only detected in the treatment group. Moreover, the phagocytic microglia (green) seem almost absent in the control brains while they can be observed in both hemispheres of the treatment group. Quantification of the tau distribution and density are currently ongoing.

**Fig. 1 (abstract O7).**
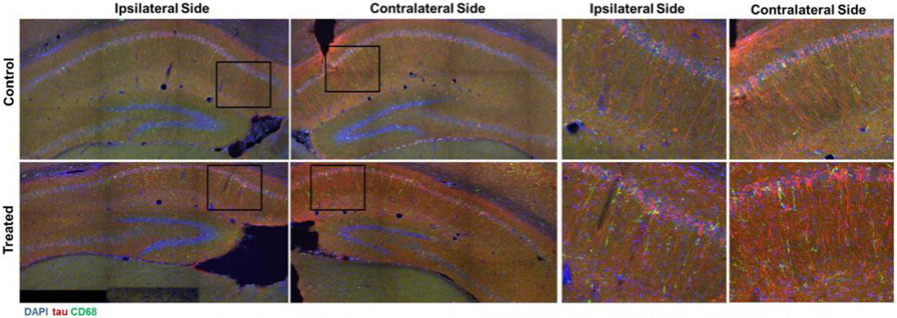
Hippocampal formation counterstained for tau protein with AT8 (red) and microglia activation with CD68 (green). The first row corresponds to the ipsilateral and contralateral side of a control brain and their magnified regions. The second row corresponds to the ipsilateral and contralateral side of a treated brain and the corresponding magnified regions

**CONCLUSIONS** Focused ultrasound-induced blood-brain barrier opening affects tau distribution after unilateral application. Axonal tau was significantly less pronounced in the sonicated region. Whether the tau protein reduced after sonication has been transferred to other cell bodies or has been entirely eliminated from the brain remains to be determined by the ongoing quantification process. Microglia were clearly activated by the sonication but its relationship to tau re-distribution remains to be established.

### O8 Evaluation of a three hydrophone-based method for cavitation localization

#### Maxime Lafond^1^, Cyril Lafon^2^, Jean-Louis Mestas^2^, Shin-ichiro Umemura^1^

##### ^1^Graduate School of Biomedical Engineering - Umemura-Yoshizawa Laboratory, Tohoku University, Sendai, Miyagi, Japan; ^2^LabTAU, INSERM U1032, Université deLyon, Lyon, France

###### **Correspondence:** Maxime Lafond

**OBJECTIVES** In the context of clinical applications, the complete characterization of the cavitation activity includes also the cavitation localization. However, the current methods for cavitation localization present various disadvantages such as the limited bandwidth of the echo imaging probes, high numerical cost and the need forexpensive equipment. This limits strongly the transfer of cavitation-based applications toward their clinical use. We propose to apply a source localization algorithm to cavitation monitoring and characterization. In the context of cavitation-related therapy, it is expected to increase the reliability level cavitation applications, giving it morecredit for clinical transfer. In order to overcome these limitations, we intend to use a PVDF hydrophone network to localize the bubble cloud, considered as a simple acoustic source.

A classic method for source localization is triangulation. The localization of the cavitation cloud is deduced from the delays obtained between threereceptors with known positions. In our case, the receptors are PVDF hydrophones. Two confocal transducers are emitting a pulse at 1.1 MHz in order to generate cavitation in the optical field of a high-speed camera. The signals from the three hydrophones were recorded during the US pulse on a digital oscilloscope and the delays between the hydrophones were calculated by finding the delay maximizing the inter-correlation between the recorded signals. The source position calculated from thedelays was finally superimposed over the images from the camera (Fig. 1). The positions calculated with this method were compared to the positions of the clouds visually estimated. The mean discrepancy was calculated. The method was firstly applied using the signals with full frequency bandwidth. Then, the post-processing operation was repeated after keeping only the bandwidth of 200 kHz around the sub-harmonic frequency (550 kHz). Also, simulations are performed to evaluate the versatility of the method in various test cases. Notably, spatial spreading of the source, source separation and the influence of the hydrophones repartition are evaluated.

**RESULTS** The high-speed camera observations were compared with the localization technique for each one of 8 independent pulses. The position of the cavitation cloudis calculated with a discrepancy of 3.1±1.8 mm in the case of the full frequency bandwidth. By processing the data only in the 200 kHz frequency band around the 550kHz sub-harmonic, the accuracy is improved to 1.4±0.8 mm. The analysis of the discrepancies indicates that there is not any systematic error associated (mean spatialerror tends toward zero). Simulations in test cases also validated the potential of the method by showing its accuracy in a wide range of configurations.

**CONCLUSIONS** A method of cavitation localization based on a three hydrophone network was explored. Localizations performed with this method were corroborated by high-speed observation in water. Also, simulations show that the method can be applied in a wide range of cases. It was shown that the use of a relatively narrow frequency bandwidth around the sub-harmonic frequency permitted to enhance the accuracy of the method. It is hypothesized that this comes from the fact that this signalis emitted only by oscillating bubbles and not by reflections of the excitation signal. In addition to the results shown in this study, this method could be augmented by a complete characterization of the cavitation event by the analysis of the cavitation signals. Also, by adding a hydrophone, 3-dimensional localization can be performed at minimal numerical cost. The obtained results and the possibilities that could be explored in the future give credit to this technique as a versatile tool for cavitationposition monitoring, a major step for clinical transfer in all applications related to cavitation.

**Fig. 1 (abstract O8).**
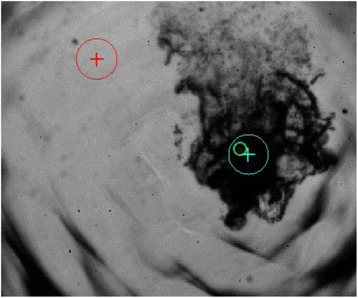
Comparison between high­speed camera observation and the position of the cavitation cloud calculated with the method based on three hydrophones. While the algorithm based on the full frequency bandwidth (red) gives an approximate position of the cavitation cloud, the one based on a bandwidth reduced around the subharmonic frequency (blue) give an enhanced localization of the cavitation cloud. The green circle denotes for the focal point of the US apparatus, used for the superimposition of the calculated position over the high­speed images

### O9 Design study and experimental validation of a novel phased array based on Fermat’s spiral

#### Pascal Ramaekers^1^, Martijn de Greef^1^, Rémi Berriet^2^, Chrit Moonen^1^, Mario Ries^1^

##### ^1^UMC Utrecht, Utrecht, Netherlands; ^2^Imasonic SAS, Voray-sur-l'Ognon, France

###### **Correspondence:** Pascal Ramaekers

**OBJECTIVES** Previous work has shown numerically that a phased array transducer based on Fermat’s spiral could potentially provide improvements for extracorporeal HIFU therapy, in particular with respect to the energy exposure of the near field. In a first step, this design study numerically evaluated phased array designs based onFermat’s spiral for three different spirals and two different element types. The transducers were evaluated on three different aspects: element size distribution, beamsteering capabilities, and pressure distribution in the near field. In a second step, one of the designs was evaluated experimentally through hydrophone measurements.

**METHODS** For the numerical evaluation of the different phased array designs, the arrays were populated by 256 elements and evaluated for two different element types: circular elements of 3.8 mm diameter (sparse arrays), and element shapes generated by applying Voronoi tessellation to the predefined element positions (tessellatedarrays). Three different spirals were used to determine the phased array element positions. The first spiral had a divergence angle of φ = 87.85°. Such a design has beenshown to minimize peak grating lobe levels in a study on 2D ultrasound imaging arrays based on Fermat’s spiral. The second spiral was constructed using φ = 137.51° (the golden angle). Previous work has shown that using the golden angle optimizes the packing efficiency of the spiral, which ensures the most uniform element size distribution. This has practical implications with respect to construction and electrical matching of the Voronoi tessellated array. The third spiral was also based on thegolden angle, but with a Taylor tapering window characterized by a sidelobe level of -30 dB applied. It has been shown for array antennas that such a tapering windowreduces sidelobe levels of the array. All transducers were designed using an aperture diameter and radius of curvature of 16 cm (f-number = 1). An overview of theevaluated tessellated arrays is shown in Fig. 1. Acoustic simulations were performed under free-field conditions using propagation of the angular spectrum of planewaves. For the experimental validation of one of the designs, pressure measurements were conducted as a function of instantaneous acoustic output power using a fiberoptic probe hydrophone (FOPH 2000, RP Acoustics).

**RESULTS** Considering the element size distribution for the Voronoi tessellated arrays, the array based on φ = 137.51° was shown to be the most uniform, with all element sizes within 20% difference from the mean element size. The array based on φ = 87.85° had 6 notable outliers, but was otherwise comparable to the array basedon φ = 137.51°. The array based on φ = 137.51° and a tapering window was shown to have a highly nonuniform distribution of element sizes, with only 89 elements within 20% difference from the mean element size. Maximum pressure levels obtained for off-axis sonications are shown in Fig. 2. The sparse arrays performed comparable in terms of beam steering, with the tapered array giving a slightly better performance than the other two. For the tessellated arrays the tapered array gave the best off-axis performance, followed by the array basedon φ = 137.51°. The worst performance is observed for the array based on φ = 87.85°. Figure 3 shows cumulative histograms of pressure levels in the near field (10 < Z < 90 mm prefocal) for an on axis sonication for each of the six arrays. The sparse arrays gave a highly similar performance in terms of near field pressure distribution. The tessellated arrays substantially reduced near field pressure levels compared to thesparse arrays. Comparing the tessellated arrays, the best performance was observed for the array based on φ = 137.51. Figure 4 shows pressure as a function of time for the hydrophone measurements on one of the tessellated transducers for different instantaneous acoustic output powers. Rarefactional and compressional pressures ranged from 14.47 and 66.53 MPa respectively at 100 W, to 40.00 and 237.50 MPa at 800 W.

**CONCLUSIONS** Tessellated arrays provide an improvement over sparse arrays in terms of near field energy exposure. This comes at the cost of reduced beam steering capabilities, but for extracorporeal HIFU applications the beam steering capabilities of the tessellated arrays are sufficient within the relevant steering range. Out of the three tessellated designs, the array based on φ = 137.51° is technically the most favorable as it has the most uniform element size distribution. The tapered array performs slightly better in terms of beam steering, but also induces higher pressure levels in the near field.

**Fig. 1 (abstract O9).**
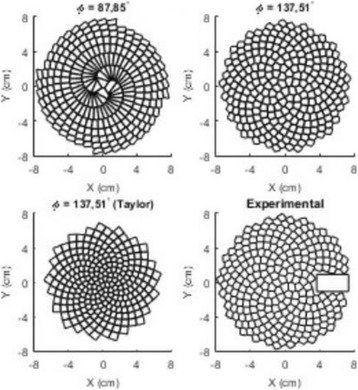
See text for description

**Fig. 2 (abstract O9).**
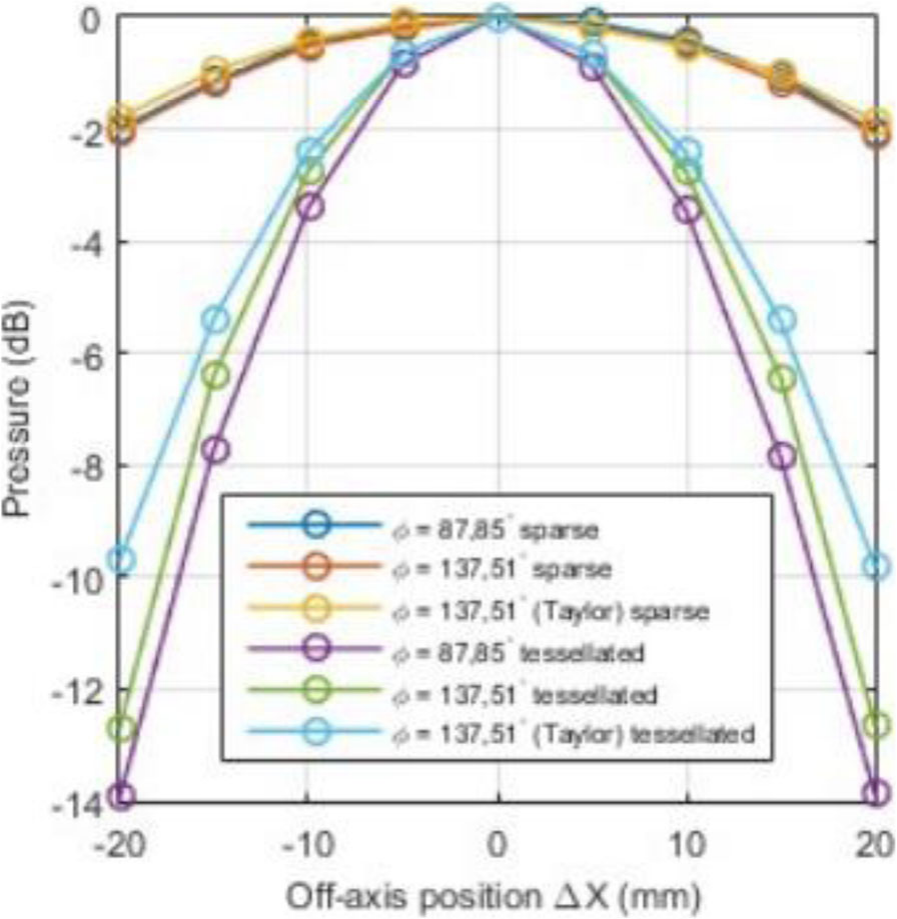
See text for description

**Fig. 3 (abstract O9).**
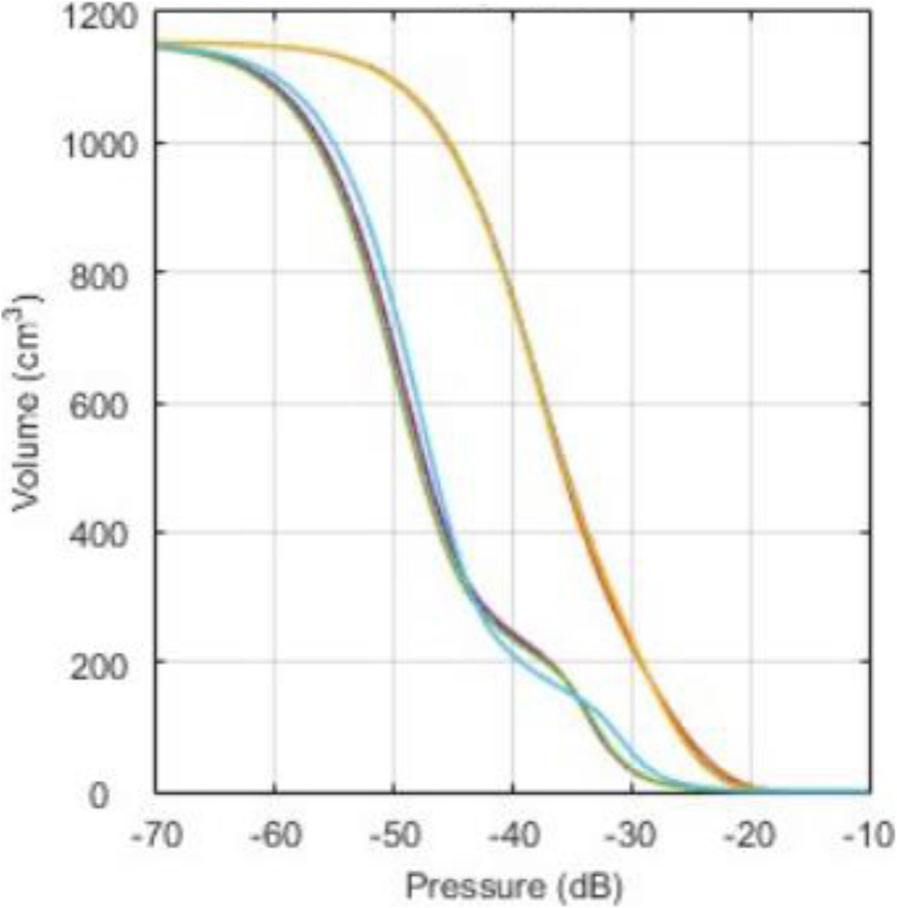
See text for description

**Fig. 4 (abstract O9).**
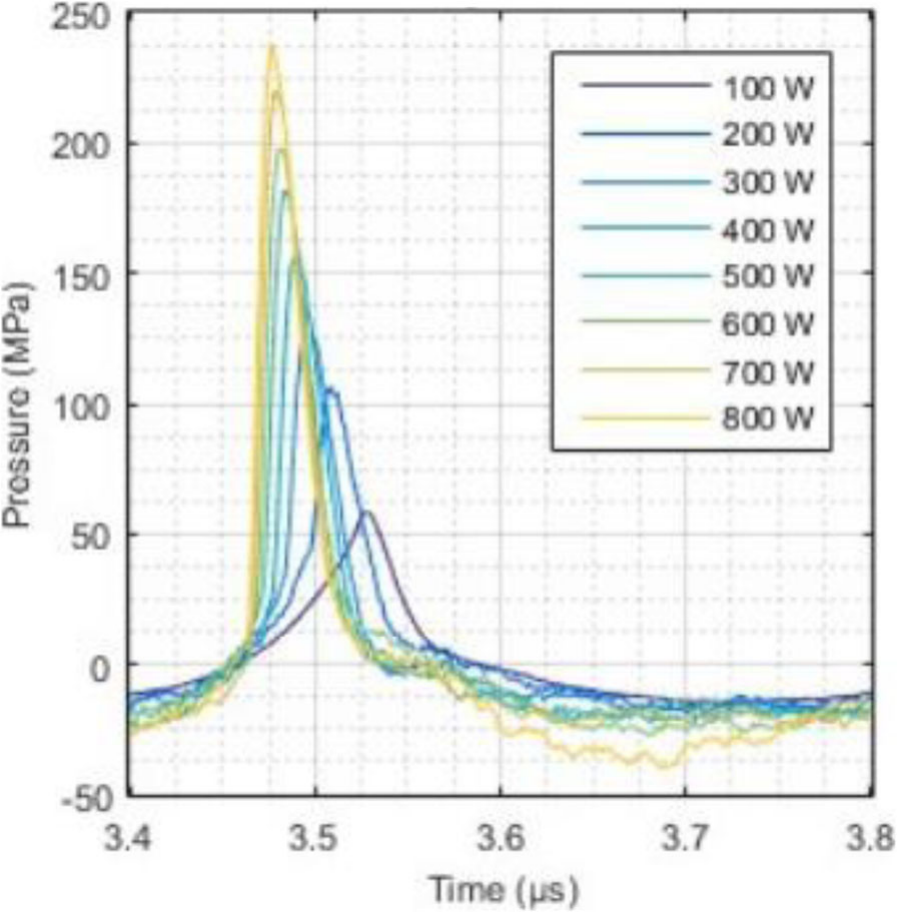
See text for description

### O10 Real-time control of the stable cavitation activity in free and vessel-confined bubble clouds

#### Corentin Cornu^1^, Matthieu Guedra^1^, Jean-Christophe Bera^1^, Wen-Shiang Chen^2^, Hao-Li Liu^3^, Claude Inserra^1^

##### ^1^Univ Lyon, Université Lyon 1, INSERM, LabTAU, F-69003, LYON, France, Lyon, France; ^2^Department of Physical Medicine & Rehabilitation, National TaiwanUniversity Hospital, Taipei, Taiwan; ^3^Department of Electrical Engineering, Chang Gung University, Taoyuan City, Taiwan

###### **Correspondence:** Corentin Cornu

**OBJECTIVES** Even if bubble collapses are commonly thought to be the key element of cell permeabilization for drug delivery applications, recent works have demonstrated the possibility of transfecting cells by gentle oscillating bubbles (stable cavitation), possibly resulting in lower cell lysis or tissue damages. Nevertheless, in a bubble cloud, both stable and inertial cavitation activities would naturally coexist, thus making difficult to quantify the contribution of both regimes on the drug deliveryprocess. In this work, we present a regulation system aiming at distinguishing the two regimes and controlling the stable cavitation activity during time.

**METHODS** A feedback-loop process is implemented on the level of the subharmonic component emitted from a bubble cloud and recorded by a needle hydrophone (Fig. 1). The cloud is created by a focused transducer emitting a 550 kHz pulsed sine wave (duty cycle: 0.2; cycle duration: 250 ms; sonication duration: 60 s) in a watertank, either in the bulk (free configuration) or in a plastic tube located at the focal point (vessel-confined configuration). The feedback-loop performs a real-time modulation of the applied voltage at a 250μs loop rate, allowing to control the subharmonic emission (SC index), as well as measuring inertial cavitation activity (broadband noise emission, IC index).

**RESULTS** Without regulation (open loop), the measure of the mean SC is not reproducible for a given acoustic intensity (Fig. 2-a), and the SC and IC indices oscillate during all the sonication time (Fig. 2-b). In contrast, the regulation system leads to reproducible results, showing a SC index which always reaches the feedback-looptarget value (Fig. 2-c) and remains constant during time (24 dB in Fig. 2-d). Moreover, as showed in Fig. 2-c, the feedback-loop allows achieving a high subharmonic response (up to 20dB) without broadband noise emission (0-5 dB), thus limiting the inertial cavitation effect. Finally, for a given high SC level, the results show a gain up to 20% of acoustic energy in comparison to the sonication without regulation.

**CONCLUSIONS** Evidences of control of the stable cavitation activity are reported, associated with (1) the control of the stochastic behavior of the bubble population, (2) a stable SC index during time, (3) the minimization of inertial cavitation activity, thus limiting the destructive effects due to bubble collapses, and (4) the saving of acoustic energy required for initiating stable cavitation. [Work supported by the French National Research Agency, LabEx CeLyA (ANR-10-LABX-0060) and granted bythe ANR-MOST project CARIBBBOU (ANR-15-CE19-0003)]

**Fig. 1 (abstract O10).**
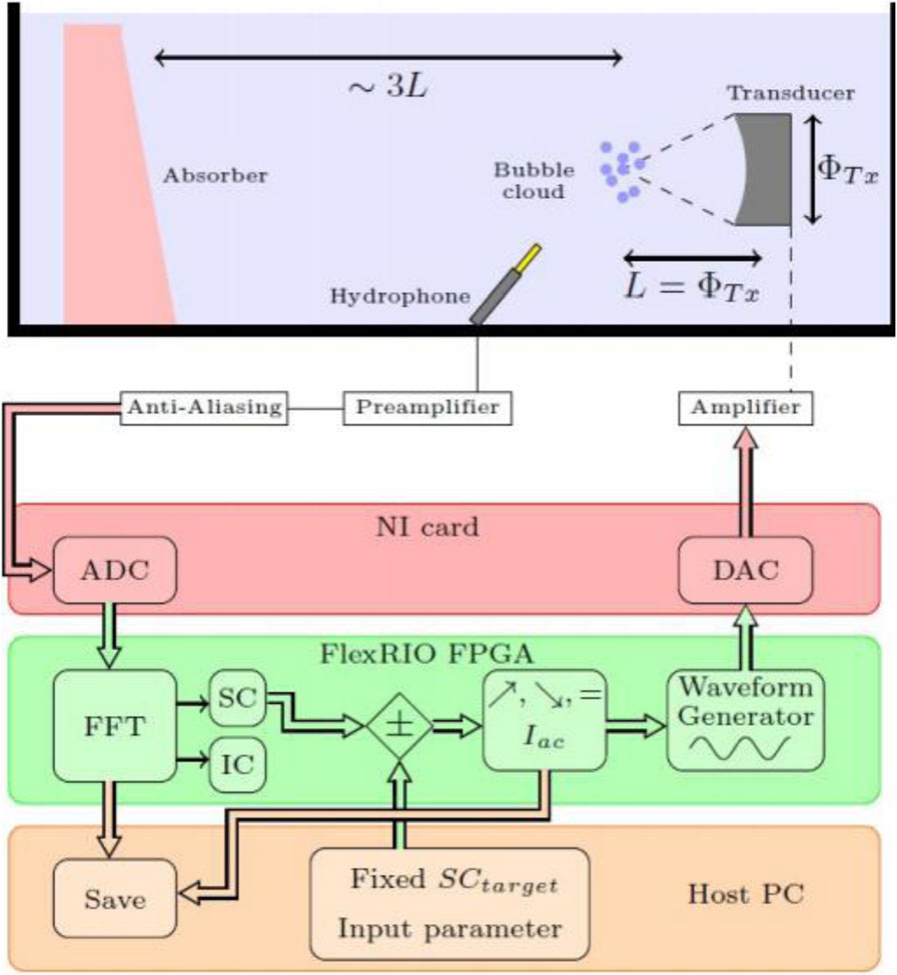
Schematic representation of the experimental in­vitro device with focused transducer in a water tank. Real­time monitoring and feedback­loop process are described

**Fig. 2 (abstract O10).**
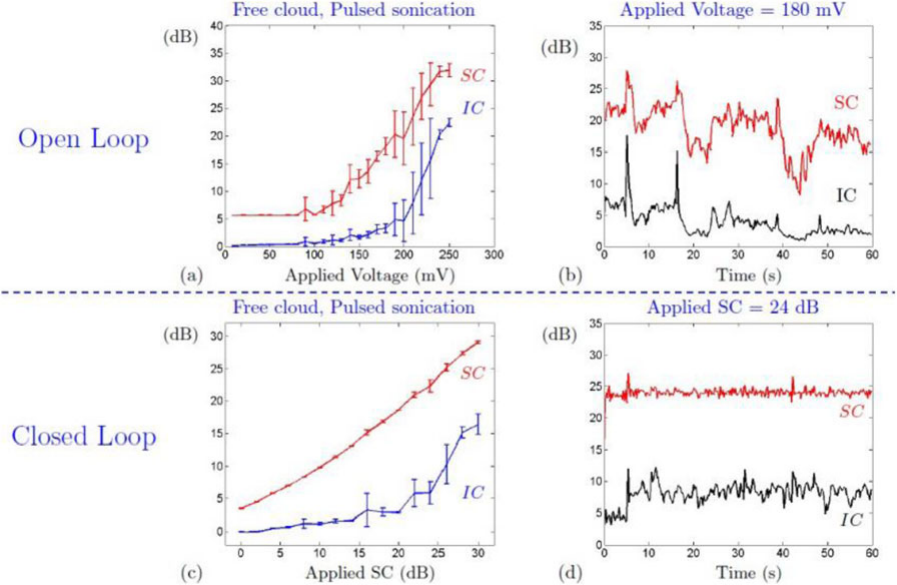
Reproducibility experiments in pulsed sonication for the SC (subharmonic emission level) and IC (broadband noise level) indicators as a function of the applied voltage in open loop (2-a) and the SC target value in closed loop (2-c). Examples of temporal stability curves for the evolution of the SC and IC indicators versus time at the applied voltage 180 mV in open loop (2-b) and at the SC target value 24 dB in closed loop (2-d)

### O11 Research platform for rodent studies of wavefront engineered ultrasonic neuromodulation

#### Steve Krupa^1^, Eilon Hazan^1,3^, Omer Naor^1^, Michael Plaksin^1^, Inbar Brosh^1^, Noam Maimon^2^, Yoav Levy^2^, Eitan Kimmel^1^, Itamar Kahn^3^, Shy Shoham^1^

##### ^1^Department of Biomedical Engineering, Technion I.I.T, Haifa, Israel; ^2^Insightec LTD, Tirat HaCarmel, Israel; ^3^Department of Medicine, Technion I.I.T., Haifa, Israel

###### **Correspondence:** Steve Krupa

**This abstract is not included as it has already been published:**

Shoham S, Krupa S, Hazan E, Naor O, Levy Y, Maimon N, Brosh I, Kimmel E, Kahn I. A126 Research platform for rodent studies of wavefront engineered ultrasonic neuromodulation. J Ther Ultrasound. 2016; 4(Suppl 1):31. Available from: https://jtultrasound.biomedcentral.com/articles/10.1186/s40349-016-0076-5

### O12 Image-guided dual-target brain stimulation on mouse by array ultrasound

#### Guofeng Li, Jiehan Hong, Qiuju Jiang, Peitian Mu, Ge Yang, Congzhi Wang, Weibao Qiu, Hairong Zheng

##### Shenzhen Institutes of Advanced Technology, Chinese Academy of Sciences, Shenzhen, China

###### **Correspondence:** Guofeng Li

**OBJECTIVES** Ultrasonic neuromodulation has become a promising approach for neural science and clinical application. Currently single element focused ultrasound transducer was widely used for performing ultrasonic stimulation. However, the stimulated position is fixed, and is hard to be precisely identified, when performing the brain stimulation. It is important to precisely steer the focus of ultrasound to predetermined targets to achieve multi-position neurostimulation. According to the classical ultrasound imaging technique by array transducer, special array transducer can be used to perform dual functions of ultrasound imaging and neurostimulation. This study investigates the feasibility of using linear array ultrasound transducer system to achieve image-guided dual-target ultrasound brain stimulation on mouse.

**METHODS** A dual modes ultrasound system, showed as Fig. 1(A), is developed to perform B-mode imaging for predetermination of stimulated target and to produceacoustic radiation force for neurostimulation. The stimulation position was controlled by beamformer inside the field programmable gate array device. Brain imaging was done first and followed by image guided stimulation. 5 male BALB/c mice, 8-10 weeks old, 20g (+/-25%) in weight were used. Mouse was anesthetized byintraperitoneal injection of ketamine (70mg/kg) and xylazine (7mg/kg) cocktail. The mouse's hair was cropped by scissor and removed by depilatory. The mouse was laid prone on an automatic heating pad (69002, RWD Life Science Co.) at 37 degrees centigrade and with its head gently immobilized using stereotaxic frame (68028, RWDLife Science Co.). Different positions were selected to evaluate the effect. Stimulated effect was evaluated by Electromyography signals recorded by an electrophysiological acquisition system (MedLab-U8C502, MedEase Ltd., Nanjing, China). The motion responses of mouse were captured by a camera (HD1080P, AoniLtd., Shenzhen, China) in real-time.

**RESULTS** Figure 1(B) demonstrates a B-mode image of a mouse brain for predetermining two different stimulation targets. The exact positions of the targets can be selected by the computer mouse, and are pointed out by the arrows. Figure 1(C) indicates the different effects of the EMG responses evoked by the ultrasound stimuli on the target A and B. Our results indicate that imaged-guided dual-target brain stimulation by array ultrasound can evoke different motion responses on mouse.

**CONCLUSIONS** The imaged-guided dual-target brain stimulation system can achieve dual targets brain stimulation with varying spatial and temporal conditions underthe guidance of B-mode imaging, which offers a useful tool for the applications on neural circuits studies and clinical therapies.

**Fig. 1 (abstract O12).**
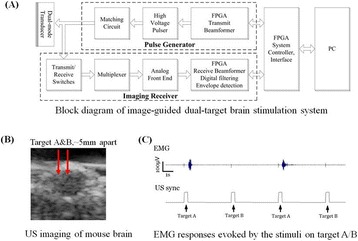
(A) shows the block diagram of the proposed imaged­guided dual­target ultrasound brain stimulation system. Figure (B) demonstrates a B­mode image of a mousebrain for predetermining two different stimulation targets. The exact positions of the targets can be selected by the computer mouse, and are pointed out by the arrows. Figure(C) indicates the different effects of the EMG responses evoked by the ultrasound stimuli on the target A and B

### O13 Pseudo-random gated sonications for reducing cavitation during thermal ablation in the brain

#### Cyril Lafon^2,1^, David Moore^3^, Matthew Eames^3^, John Snell^3,4^, James Larner^2^, Neal Kassell^3,4^

##### ^1^LabTAU, INSERM, Lyon, France; ^2^Department of Radiation Oncology, University of Virginia, Charlottesville, Virginia, USA; ^3^FUS Foundation, Charlottesville, Virginia, USA; ^4^Department of Neurosurgery, University of Virginia, Charlottesville, Virginia, USA

###### **Correspondence:** Cyril Lafon

**OBJECTIVES** Transcranial HIFU is now used in clinics for treating essential tremor and proposed for many other brain disorders. This promising treatment modality still faces several limitations. HIFU-induced thermal ablation in the brain requires high energy resulting eventually in undesired cavitation and potential side effects. Original strategies should be tested to increase treatment safety and efficacy. The goals of the present work were: 1- to evaluate the potential increase of the cavitation threshold using pseudo-random gated sonications and 2- to assess the heating and steering capabilities with such sonications. The performance of pseudo-random sonications was compared with conventional continuous exposures.

**METHODS** The experiments were performed with the transcranial MR-compatible ExAblate Neuro system (InSightec). It is a 1024-element, 30 cm diameter, 15 cm focal length, transducer operating at a frequency of 660 kHz. Four methods of sonication were compared: continuous wave (CW), gated emissions with pseudo-random 2ms (p-Rand2ms) or 33 us (p-Rand33us) -period codes and 30 kHz square (Squ30kHz). The duty cycle (DC) was set to 50% for the gated sonications. The cavitation threshold was first evaluated in water. For each condition, electrical driving power was increased step by step from 20 to 500W and the acoustical noise was recorded with the integrated passive cavitation detectors. The spectral energy was averaged over the 250-500 kHz bandwidth and the sonication time (10s). Heating trials were then performed in a hydrogel tissue mimicking material (TMM, ATS Laboratories). The electrical power was set to 15, 30 or 60 W, the exposureduration to 9 or 18 s. For a fair comparison with CW, 50% DC sonications had either the electrical power or the exposure duration doubled. The temperature was measured by MR-thermometry when focusing at the geometrical focus and when steering the beam off-focus by 5mm-steps.

**RESULTS** The assumption was made that the occurrence of cavitation resulted in an inflexion of the scattered energy vs. power curve. Due to frequency modulation, harmonics occur in the bandwidth of interest for the p-Rand33us and Squ 30kHz sonications and their energy increased also with power. The threshold was then harder to assess. Sudden changes in the scattered energy occurred at electrical powers of 180, 400, 500, 400W for CW, p-R and 2ms, p-Rand33us and Squ30kHz respectively. Unsurprisingly, higher thresholds were measured when repeating the experiments in more degassed water. These settings did not allow to heat the TMM when electronically steering the focus more than 15mm-off the geometrical focus. Doubling the exposure duration did not allow to compensate the 50% DC because of thermal diffusion. At a power of 30W, a maximal temperature rise of 9°C was measured with gated sonications applied for18s while CW 30W sonication for 9 s gave a 13°C increase in temperature. However, the heating patterns obtained at 60W with p-R and 2ms and 30 W in CW were very similar for a constant 9s duration. For the same conditions, lower temperature rises were measured for p-R and 33us and Squ30kHz. Several assumptions can be made to explain this difference: the gating is too rapid for reaching steady state, the amplifier struggles to switch so quickly, or cavitation enhanced heating is reduced.

**CONCLUSIONS** Coded sonications were proposed using rapid random phase shifts (Chapelon et al. 1996) or chirp (Tang and Clement 2009). In the present work, it has been demonstrated on a clinical device that randomly gated signals also increase the cavitation threshold in water. The general idea consists in breaking the dynamic of the bubbles moving from monochromatic to broadband sonications. It was then necessary to check that the heating and steering capabilities of the system were preserved. Among the tested gates, 2ms period pseudo-random codes seem to be the modulation that increases the cavitation threshold the most while preserving heating capabilities. Indeed, very fast switches may be associated to electronic difficulties for non-demonstrated benefit in terms of cavitation reduction. Work sponsored by the FUS Foundation through the Robert Merkin Fellowship and performed with the technical support of InSightec.

### O14 A flat array with 4096 elements for MR-guided focused ultrasound therapy

#### Yuexi Huang^1^, Ben Lucht, Rohan Ramdoyal^1^, Samuel Gunaseelan^1^, Tyler Portelli^1^, Ping Wu^1^, Kullervo Hynynen^1,2^

##### ^1^Sunnybrook Research Institute, Toronto, Ontario, Canada; ^2^Department of Medical Biophysics, University of Toronto, Toronto, Ontario, Canada

###### **Correspondence:** Ben Lucht

**OBJECTIVES** MR-guided focused ultrasound has been demonstrated in various applications for non-invasive thermal ablations. The current clinical devices for body applications use spherically curved phased arrays, which improve focus quality for a limited number of transducer elements (~200), at the expense of a limited steering range, therefore mainly rely on mechanical positioning for covering a large treatment volume. In this study, we developed a fully populated flat array, which allows for amuch wider steering range. Feasibility using this new design for thermal ablation and hyperthermia over large target volumes was demonstrated in animal studies.

**METHODS** A flat array of 14 cm in diameter with 4096 elements was manufactured in house with center-to-center element spacing of half-wavelength at the centre frequency of approximately 500 kHz. Custom driving electronics were built using Application-Specific Integrated Circuit (ASIC) technology. The MR-compatible arrayand driving system were mounted on the standard bed of an MR scanner (MR750, GE Healthcare, Milwaukee, WI, USA). In vivo studies were performed on 16 pigs on thigh muscles. Acoustic power up to 240W over 50s were applied with MR thermometry monitoring. Focus was either stationary during sonications or steered in circularpattern laterally or along the acoustic beam. The minimum delay between steering spots for loading new phasing parameters was 3 ms. Lesions were measured by T2-weighted imaging (FRFSE, TR 5000ms, TE 100ms). For hyperthermia applications, multi-foci sonication pattern was applied to achieve heating over a large volume (30cm3) for 15 min at 43°C.

**RESULTS** The array focused energy well by using geometric beam-steering phase delays and was able to provide sustained acoustic power with repeated treatments.Temperature was above 60°C at focus. Good volumes of thermal coagulation were achieved with a wide steering range as large as 12 cm. For hyperthermia, heating around 43°C over the large volume was observed by MR thermometry.

**CONCLUSIONS** In this study we demonstrate for the first time that fully electronically steered arrays are feasible. With a flat design and transducer element spacing at half-wavelength, wide steering was achieved without sacrificing focus quality. A wide steering range also eliminates the need for a motorized positioning system, which simplifies system design and allows precise MR thermometry without motion induced artifacts.

### O15 Effects of rarefactional pressure from pulsed focused ultrasound in murine melanoma and breast tumor models: implications for immunotherapy

#### Omer Aydin, Scott R. Burks, Saejeong Kim, Joseph A. Frank

##### Radiology and Imaging Sciences, NIH Clinical Center, Bethesda, Maryland, USA

###### **Correspondence:** Omer Aydin

**OBJECTIVES** Focused ultrasound (FUS) has long been used for thermal ablation of malignant tumors. The potential role for nonthermal pulsed FUS (pFUS) as amethod to modulate the innate immune response to tumors is under investigation. We have previously characterized the molecular changes in numerous tissues and have demonstrated changes in the tissue microenvironment and immune cell phenotypes following pFUS. However, the parameters needed to maximize pFUS-inducedmolecular changes in tumor microenvironments that could modulate immunotherapeutic response are essentially uninvestigated. We treated the B16 murine melanomaand 4T1 breast cancer flank tumors with pFUS of 1 MHz over a range of peak negative pressures (PNP) (1–8 MPa) without microbubble infusions and evaluated the molecular response to sonication.

**METHODS** Subcutaneous B16 or 4T1 tumors were established in the legs of C57BL/6 (B16) or BALB/c (4T1) mice and allowed to reach ~8 mm in diameter. Magnetic resonance-image-guided pFUS with passive cavitation detection (RK100, FUS Instruments, Toronto, ON, Canada) or ultrasound-image-guided pFUS (VIFU, Alpinion Medical Systems, Bothell, WA) were administered at 1 MHz over a range of PNP values (1, 2, 4, 6, and 8 MPa). One hundred sonications were delivered to each rasterpoint using a 10% duty cycle and a pulse repetition frequency of 10 Hz. During treatment planning, the entire tumor volume was covered and 2-mm spacing was used between points. Twenty-four hours after pFUS, tumors were harvested for histology and molecular analyses.

**RESULTS** During pFUS, half-harmonic emissions were only detected only at 8 MPa PNP in B16 tumors and at 6 and 8 MPa PNP in 4T1 tumors. Twenty-four hours after pFUS, we examined expression of intercellular adhesion molecule (ICAM), vascular cell adhesion molecule (VCAM), and cyclooxygenase (COX2) in each tumortype. ICAM was elevated in 4T1 and B16 tumors between 2 and 4 MPa (P <0.05) compared to untreated tumors (Fig. 1). We did not observe statistically significant elevations in COX2 for B16 tumors, but COX2 was elevated in 4T1 tumors treated with pFUS as 4, 6, and 8 MPa (P <0.05). VCAM expression was not upregulated bypFUS in either tumor type at any pressure. Furthermore, H&E staining revealed wide-spread coagulative necrosis and red blood cell extravasation in both tumor typestreated at 8 MPa compared to lower pressures.

**CONCLUSIONS** We found that pFUS could induce molecular changes in B16 and 4T1 tumors that are consistent with molecular response that can influence immune cell tropism. Interestingly, we demonstrate that there may be optimal rarefaction pressures to induce appropriate molecular changes in tumors. For example, the greatest increases in ICAM, that could facilitate immune cell infiltration, was observed below the cavitation threshold in both tumor types. Furthermore, we show that molecular responses can be fundamentally different across tumor types (e.g. changes in COX2 in 4T1 tumors, but not B16 tumors), that may reflect differences in the physiological and functional responses. These results suggest it may be possible to use pFUS to modulate immune function, but that a greater understanding of pFUS molecular effectsis needed. Given the heterogeneous response across tumor types, that each pFUS parameters may need to be empirically evaluated.

**Fig. 1 (abstract O15).**
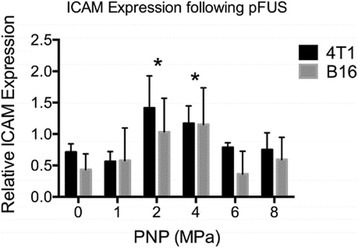
See text for description

### O16 Boiling histotripsy ablation of renal carcinoma in the Eker rat produces significant changes in the immune system

#### George R. Schade^1^, Wayne Brisbane^1^, Stella Whang^2^, Yak-Nam Wang^3^, Kayla Gravelle^2^, Venu Pillarisetty^4^, W. Conrad Liles^5^, Vera Khokhlova^3^, Michael Bailey^3^, Tatiana D. Khokhlova^2^, Joo Ha Hwang^2^

##### ^1^Department of Urology, University of Washington, Seattle, Washington, USA; ^2^Department of Gastroenterology, University of Washington, Seattle, Washington, USA; ^3^Center for Industrial and Medical Ultrasound, University of Washington, Seattle, Washington, USA; ^4^Department of Surgery, University of Washington, Seattle, Washington, USA; ^5^Department of Medicine, University of Washington, Seattle, Washington, USA

###### **Correspondence:** George R. Schade

**OBJECTIVES** Focused ultrasound (FUS) exposures have demonstrated the ability to modulate the immune system to stimulate an anti-tumor immune response. Ourgroup has been developing boiling histotripsy (BH) as a non-invasive treatment for renal carcinoma (RCC) and have previously shown short-term changes in systemicand local cytokines and infiltration of CD8+ T-cells following BH treatment *in vivo*. We characterized the long-term immune response to BH RCC tumor ablation in a ratmodel.

**METHODS** RCC bearing genotyped Eker rats (Tsc2 heterozygotes) were randomly assigned to transcutaneous BH or FUS SHAM procedure targeting ~0.5 cc of RCC or non-tumor bearing normal kidney. BH was delivered with a 1.5 MHz US-guided small animal FUS system (VIFU-2000, Alpinion) operated at duty cycles of 1-2%, 10-20ms pulses, 525-600 W electric power. Following treatment rats were recovered, underwent serial US imaging surveillance and serial blood draws, and were survived for7, 14, or 56 days. Following euthanasia, the treated and contralateral kidney, tumor draining lymph nodes (TDLN), and spleen were collected. Flow-cytometry was performed on processed tissues and blood to analyze for changes in circulating and local immune cell populations.

**RESULTS** BH treatment was associated with significant alterations to the immune system within 2 weeks vs. SHAM treatment with characteristics of an anti-tumor immune response (see Fig. 1). Specifically, BH was associated with a significant 3.4-fold (p=0.048) increase in splenic CD11c+ antigen presenting dendritic cells. BH also produced alterations in CD4+ helper T-cell populations with a significant 1.2-fold (0.041) increase in CD4+ CD62L- CD44+ effector memory cells and near significant 1.8-fold (p=0.08) increase in central memory CD4+ CD62L+ CD44- cells in TDLNs. Finally, BH treatment resulted in significant alterations in cytotoxicCD8+ T-cell populations. Specifically, BH treatment was associated with significantly increased CD8+ CD62L- CD44+ effector memory cells in both TDLNs and spleen (3.0-fold, p<0.01 and 2.9-fold, p=0.03, respectively) and central memory CD8+ CD62L+ CD44- cells in TDLNs (6.8-fold, p <0.01) with a corresponding significant decrease in CD8+ CD62L+ CD44+ naïve cells in TDLNs (0.4-fold, p=0.01).

**CONCLUSIONS** These data represent the first extensive analysis of the immune response to BH tumor treatments and suggest that BH RCC ablation produces significant changes to the immune system suggestive of an anti-tumor response. Analysis of longer-term survival (56 days) is ongoing and will demonstrate if these changes are long-lived. Future studies will further evaluate the specificity of this response and whether it can improve clinically relevant outcomes. Funding: The Focused Ultrasound Foundation, The Urology Care Foundation, and NIH K01EB015745 and R01CA154451.

**Fig. 1 (abstract O16).**
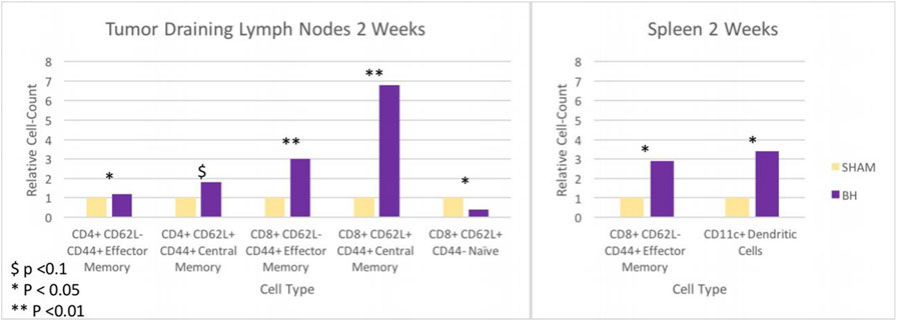
Flow cytometry results from tumor draining lymph nodes and spleens 2 weeks following SHAM or BH treatment of RCC

### O17 Potentiating checkpoint inhibitor therapy with ultrasound stimulated microbubbles

#### Sharshi Bulner^2^, Aaron Prodeus^1, 2^, Jean Gariepy^1, 2^, Kullervo Hynynen^1, 2^, David Goertz^1, 2^

##### ^1^University of Toronto, Toronto, Ontario, Canada; ^2^Sunny brook Research Institute, Toronto, Ontario, Canada

###### **Correspondence:** Sharshi Bulner

**OBJECTIVES** Immunotherapies are playing an increasingly prominent role in the treatment of cancer, driven in large part by the success of checkpoint pathway inhibitors such as anti-PD-1 monoclonal antibodies. These approaches inhibit the ability of tumors to evade the immune system and boost the activity of anti-tumor Tcells. However, potent and durable results are to date only obtained in subsets of patients with certain cancers (e.g. melanoma). They are less effective in tumors with low immunogenicity, and those with low levels of tumor infiltrating lymphocytes (TILs). As a result, it is increasingly recognized that combination therapies will be required. While it is known that different forms of therapeutic ultrasound can elicit a spectrum of immune responses, it remains to be established if they can be harnessed to potentiate the effects of checkpoint inhibitors. In this study we examine the combination of anti-PD-1 therapy with ultrasound stimulated microbubbles (USMBs). Inparticular, we use a level of ultrasound exposure that is non-thermal, but elicits a significant degree of vascular injury.

**METHODS** A murine colorectal cancer cell line (CT26.wt) was employed to initate tumors subcutaneously in the hind limbs of female Balb/c mice. There were four study groups: microbubbles-only (control/sham treatments), Anti PD-1 (drug-only group), USMBs (ultrasound-only treatment group) and USMBs + Anti PD-1(combined treatment group). Experiments commenced when tumors were well established, in the range of 50-100 mm3. The immunotherapy drug used in this study is ananti-mouse PD-1 (clone: RMP1-4, Bioxcell) and was administered at a dosage of 200 μg intraperitoneally prior to ultrasound or sham treatments, and then subsequentlyadministered every 3 days for a total of 5 doses. The microbubbles employed were an experimental phospholipid encapsulated agent administered in bolus form prior toinsonation. Ultrasound exposures were delivered with a single element focused 1 MHz transducer with the entire tumors within the -3dB beam width. The pulsing scheme was to send 50 0.1 ms long pulses (1.6 MPa peak negative pressure) spaced 1 ms apart, which was repeated at 10 second intervals for a duration of 3 minutes. This low duty cycle approach avoided thermal elevations and has been previously established to induce vascular injury in tumors. Antitumour effects was assessed withlongitudinal experiments (n=5-7 per group), where tumor growth was evaluated every three days until ethical size endpoints were reached. Two sets of acute experiments were conducted for all groups, where tissue (tumors, spleens and tumor draining lymph nodes) was harvested at either 3 or 7 day time points post induction (4-6 pergroup). Tumor tissue underwent flow cytomtery analysis to assess immune cell status. Splenic and lymphatic tissue was subject to cell count analysis and ELISPOT to quantify the expression of IFN-gamma as a metric of T-cell activation.

**RESULTS** It was observed that the combined treatment group had a significantly smaller tumor volume compared to the sham group, USMB-only group and anti PD-1group at Day 6 (p<0.001, p<0.05 and p<0.05, respectively) and at Day 9 (p<0.001, p<0.001, p<0.001, respectively). Using a size dependant endpoint (>1000 mm3), the combined treatment group resulted in a significantly longer survival time compared to MBs (p<0.01), USMB (p<0.05) and anti PD-1 (p<0.01). Median survival between the combined treatment group and individual treatments was 16 days versus 10 days. Flow cytometry showed that the USMB treatments produced elevated levels oftumor infiltrating leukocytes (TILs - CD45+) and that the ratio of cytotoxic T-cells (CD8+) to regulatory T cells (CD45+ Foxp3+) was increased. At Day 3, the combined therapy group had significantly higher TIL and cytotoxic T-cell levels than the anti PD-1 monotherapy (p<0.05) group.

**CONCLUSIONS** The results demonstrate that USMB treatments in combination with anti PD-1 therapy resulted in a significant inhibition of tumor growth. The data suggest that USMB therapy may be potentiating anti PD-1 therapy through a T-cell dependent mechanism. This approach is a means by which to enhance checkpoint inhibitor effects without the deliterious side effects associated with adding other immunotherapy or chemotherapy agents.

### O18 Proteomic and histological effects of pulsed focused ultrasound in the rat heart

#### Kee W. Jang^1^, Tsang-Wei Tu^1^, Scott R. Burks^1^, Bobbie K. Lewis^1^, Joseph A. Frank^1,2^

##### ^1^Radiology and Imaging Sciences, National Institutes of Health, Bethesda, Maryland, USA; ^2^National Institute of Biomedical Imaging and Bioengineering, National Institutes of Health, Bethesda, Maryland, USA

###### **Correspondence:** Kee W. Jang

**OBJECTIVES** The purpose of this study was to investigate the effects of pulsed focused ultrasound (pFUS) exposures to the rat heart and evaluate the proteomic and histological changes temporally following sonication.

**METHODS** Eight to ten week old female Sprague Dawley rats were imaged on a 3T MR scanner (Achieva, Philips Healthcare, USA) and T2-weighted MR images were acquired with 8.9ms repetition time (TR), 4.5ms echo time (TE) in 1mm slice thickness through the chest wall in sagittal plane. The MR images were used as guidance for pFUS to target the left ventricular apical myocardium (center frequency 1.1MHz; PNP 3MPa; 10ms bursts; 1Hz PRF; 100 sonications/point, 40 points to the apex, RK100, FUS Instruments, CAN). Following pFUS treatment, proteomic analysis was performed of sonicated myocardium lysate (n=5/timepoint) over 24 hrs along withcardiac injury markers from the myocardium and serum (s) using single- or multi- plexed ELISA. In order to monitor and examine the activity of cardiac function, electrocardiogram (EKG) was recorded and analyzed at pre-determined time points using EKG recording module (IX-ECG12, iWorx Systems, Inc., NH). pFUS treatedmyocardium (n=3) were stained with haematoxylin and eosin (H&E) to evaluate the morphology. Statistical analysis was performed using ANOVA with multiple comparisons to sham control with p<0.05.

**RESULTS** pFUS treatment to the myocardial apex induced a delayed pro-inflammatory cytokine expression with significant elevations in interleukin (IL) 1α, 1β, 17tumor necrosis factor alpha (TNFα) interferon gamma (IFNγ) and anti-inflammatory factors including IL4, 10 along with chemo-attractant factor regulated on activation, normal T cell expressed and secreted (RANTES). Significant (p<0.05) cardiac biomarkers including cardiac troponin (cTn) and N-terminal pro b-type natri-uretic peptide(NT-proBNP) were also detected in both serum and myocardium that would be indicative of cardiac injury (Fig. 1). The expression of proinflammatory cytokines including became significant (p<0.05) at 12 through 24 hrs after pFUS treatment. The increase in cyclo oxygenase 2 (COX2) at 24 hours would suggest that the molecular changes would be occurring through NFkB pathway. The significantly increased levels of cardiac injury markers, NT-proBNP and cTnI, in both myocardium and serum were observed after 18 hrs following pFUS treatment. Analysis of EKG signals revealed that the heart rate declined immediately after pFUS and within 1 hour returned back to normal (Fig. 2). H&E stains did not show morphologic changes in the apex in pFUS treated myocardium compared to sham controls (Fig. 3).

**CONCLUSIONS** We demonstrated a reproducible and delayed mild cardiac injury model using MR-guided pFUS the rat. Proteomic changes and cardiac markers wouldindicate that the evolution of inflammatory response to low pressure pFUS (3MPa) starting approximately 6 hours following pFUS would suggest that sonication is causing a delayed cardiac injury. Further histological and transcriptomic analysis will be needed to understand the pathophysiological effects of pFUS to the heart and whether the model can be used as a basis to evaluate myocardial contusion.

**Fig. 1 (abstract O18).**
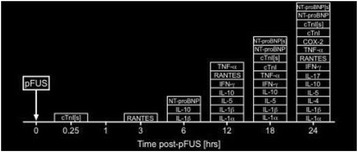
Proteomic analysis of pFUS treatment in a rat myocardium. Pro­ and anti­ inflammatory cytokines including cardiac injury biomarkers showed delayed enhancement following pFUS treatment and the increased expression lasted the end of pre­determined time points. Significance was determined by ANOVA p <0.05

**Fig. 2 (abstract O18).**
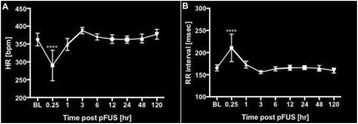
Analysis of EKG before and after pFUS. (A) Heart rate (HR) was immediately declined following pFUS and returned back to normal shortly. (B) Transient elevation of RR interval confirmed the changes of HR following pFUS treatment. Significance was determined by ANOVA ****p<0.0001

**Fig. 3 (abstract O18).**
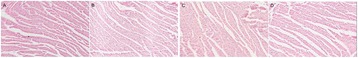
H&E stain of pFUS treated myocardium. Compared to SHAM (A), There was not difference in myocardial morphology different at post 6 hrs (B), post 24 hrs (C)and post 120 hrs (D) following pFUS. Bar = 100μm

### O19 CMUT transducers for interstitial MR-guided high intensity ultrasound heating: *in-vivo* feasibility in a pig brain model

#### William Apoutou N'Djin^1,2^, Jeremy Vion^1,2^_,_ Loïc DAUNIZEAU^1,2^, Christopher Bawiec^2^, Guillaume BOUCHOUX^1, 3^, Nicolas Sénégond^4^, Jean-Yves Chapelon^1,2^, Alexandre CARPENTIER^3,5^

##### ^1^LabTAU, INSERM, U1032, Lyon, Rhone-Alpes, France; ^2^Univ Lyon, Université Lyon 1, Lyon, F-69003, France; ^3^CarThera Research Team, Brain and Spine Institute, Paris, France; ^4^Vermon SA, Tours, France; ^5^Department of Neurosurgery, Assistance Publique, Hopitaux de Paris, Pitie Salpetriere, Paris, France

###### **Correspondence:** William Apoutou N'Djin

**OBJECTIVES** Biomedical Capacitive Micro-machined Ultrasound Transducers (CMUTs) have been mainly developed for imaging purposes. CMUTs exhibit several advantages, such as ease of miniaturization (cell size: micron size), inherently broad bandwidth (>several MHz) and good MR compatibility. This technology also has potential for generating high intensity ultrasound, which could have an interest in developing ultrasound thermal ablation therapies. To date however, the feasibility for generating high intensity ultrasound with CMUTs has only been described in a few modeling and in-vitro studies. The use of CMUTs in continuous wave (CW) mode ofoperation remains challenging since it requires the development of robust cell structures and dedicated driving strategies. Here, we present an in-vivo study investigating the feasibility of generating directional ultrasound-induced heating and thermal damage in brain tissue, with CMUTs designed for interstitial high intensity contact ultrasound (HICU) applications under magnetic resonance (MR) guidance.

**METHODS** Two types of CMUT prototypes were fabricated using a series production batch of CMUTs and a wafer bonding based process. A first intermediary CMUTprototype was made of 5 columns of 4-element 1D-linear arrays mounted on a 16 cm long, 5 mm wide rigid PCB. The elements were electrically coupled 2 by 2 within agiven column (element size: 2.7 mm x 0.8 mm). A final prototype was a multi-faceted CMUT-based HICU catheter incorporating ten 1D-linear arrays, 32.4 mm long and 0.8 mm wide. The arrays were mounted on a cylindrical 9-French flexible catheters (20 cm long), which formed a prism-shaped 2D array for multidirectional radial ultrasound exposures. CMUT prototypes were used in a porcine model for generating thermal ablations in brain tissue interstitially. Preliminary numerical simulations allowed identifying a range of surface ultrasound intensities (Iac) suitable for inducing thermal ablation in brain tissues with these CMUT designs (Iac > 10 W•cm-2). CMUTs were used in CW mode (HICU sequence: 4s ON/1s OFF, f = 7.9 MHz), and the bias and driving voltages were chosen in order to operate in the collapses napback regime and reach the intensity level required for thermal HICU lesioning. Ultrasound exposures conditions were applied through an escalation dose process (total exposure time: from 3 to 15 min, up to 10 active CMUT elements). HICU-induced thermal heating generated with CMUTs was evaluated *in vivo* on 10 pigs and monitored under real-time multi-planar magnetic resonance thermometry (MRT) (Fig. 1).

**RESULTS** Overall, directional HICU-induced temperature increases were generated in the in-vivo porcine brain with CMUTs. The heating pattern could be monitoredwith excellent time-space resolution beyond a radius of 1 mm around the CMUT device (12 MRT maps every 1s, temperature standard deviation: ± 2.5°C). Heating patterns extended over 1 cm from the CMUT elements within 2 min exposures. HICU exposures could be performed continuously without a water-circulating coolingsystem. After 6 min, the temperature of the brain tissue was increased locally above the 55°C threshold necessary for the creation of irreversible thermal damage (△Tmax> 35 °C). Treatment volumes > 1.5 cm3 could be completed within 13 min. Just after treatment, tissue changes were visible on T1- and T2-weighted anatomical images. Tissue ablation boundaries detected on T1w and T2w images, respectively hypo and hyper signal boundaries, correlated well with the 55°C isotherm boundaries (MRTmaps). Several contrasts were observable on MR images within the lesion area, which were consistent with previous studies reporting the presence of coagulation andliquefaction necrosis in brain after high intensity ultrasound exposures. Gross sample and histological analyses confirmed the presence of brain tissue coagulations.

**CONCLUSIONS** The feasibility of HICU therapy with CMUTs has been established *in vivo*. Further investigations are ongoing to improve the robustness of the CMUTdevices and increase the treatment volumes. This project was supported by CarThera, the French National Research Agency (ANR, 2010) and Single InterministerialFund (FUI, 2013).

**Fig. 1 (abstract O19).**
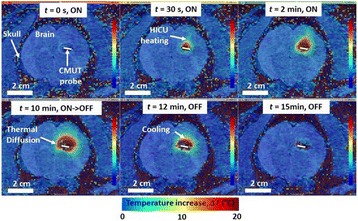
In-vivo interstitial HICU heating induced in a porcine brain with CMUTs under MR temperature monitoring

### O20 Real-time HIFU beam imaging using beamforming in an ultrasound scanner

#### Kazuhiro Matsui^1^, Françoise CHAVRIER^2, 4^, Takashi Azuma^3^, Ichiro Sakuma^1, 5^, William Apoutou N'Djin^2, 4^, Rémi Souchon^2,4^

##### ^1^Engineering, The University of Tokyo, Tokyo, Japan; ^2^LabTAU, INSERM unité 1032, Lyon, France; ^3^Medicine, The University of Tokyo, Tokyo, Japan; ^4^Univ Lyon, Université Lyon 1, Lyon, France; ^5^Medical Device Development and Regulation Research Center, Tokyo, Japan

###### **Correspondence:** Kazuhiro Matsui

**OBJECTIVES** High Intensity Focused Ultrasound (HIFU) therapies can currently be assisted by medical imaging for guidance or monitoring of some tissue characteristics (e.g. anatomical structures, physiological parameters), but no visualization of the HIFU beam itself is currently provided for assisting the treatment targeting. In biological tissues, however, the HIFU beam may be strongly attenuated, or may even focus outside the desired target region, for example due to uncontrollable experimental parameters (acoustic coupling at the interface of the ultrasound source, anatomical barriers such as bones, gas), or due to multiple tissue layers having different ultrasoundpropagation properties (e.g. muscle, skin or fat). These experimental parameters are difficult to quantify precisely a priori, and cannot be completely accounted for during calculation of the focusing parameters.There is therefore a risk of applying the HIFU energy and thus creating a therapeutic effect (e.g. thermal lesion) at a wrong position inside the medium, which potential deleterious consequences on the efficacy, accuracy, and safety of the treatment.

The object of this study is to provide a method for imaging, in real time, the HIFU beam inside an acoustically propagative medium using beamforming in an ultrasound scanner.Novelty and advantagesThe novelty of the method can be found in its implementation of beamforming in an ultrasound scanner, which is analogous to the backward reconstruction using time reversal. The advantage of this method is its real-time imaging capability owing to digital parallel processing of the scanner. Further advantage is simplicity of the imaging system without requiring additional equipment aside from an ultrasound scanner and an ultrasound array serving as a time-reversal mirror.

**METHODS** Imaging technique:The method for imaging in real time the HIFU beam comprising steps of: 1) emitting a HIFU beam in the tissues; 2) measuring voltage signal waveform values at a reception region using an ultrasound array probe serving as a time-reversal mirror; 3) calculating acoustic field values in the tissues using the reconstruction algorithm implemented in a ultrasound beamformers with digital parallel processing units; 4) generating the HIFU beam image. The experimental set-up included a mono-element HIFU spherical transducer (emitter, focal distance: 90 mm, working frequency: 2.4 MHz) and a commercial ultrasound imaging probe (receiver, model: L7-4, ATL-Philips) for collecting the emitted HIFU signals. In this experiment, the emitter was aligned with the receiver face to face each other. For the wave field reconstruction, an ultrafast ultrasound scanner (Vantage 256, Verasonics) was used for the signal recording and processing *via* plane-wave beamforming. Evaluations: The feasibility of the method was evaluated using either the time-reversal reconstruction or the beamforming reconstruction, with and without HIFU beam aberrations. First, in the experiment in water without HIFU beam aberrations, performance of the method was demonstrated in comparison with the reference field obtained by numerical calculation of the forward propagation using the Rayleigh integral and hydrophone scanning. Then, in the experiment with HIFU beam aberrations induced by heterogeneous hydrogel, HIFU beam visibility was evaluated with referred to the HIFU pressure field measured by hydrophone scanning.

**RESULTS** In a lossless medium (water), qualitative agreements were found between the amplitude of the reference HIFU field from numerical calculation (Fig. 1a) andthe HIFU fields obtained from real experimental acquisitions after time-reversal and beamforming reconstructions (Fig. 1b and c). In the case of a heterogeneous medium including multiple bubbles and cracks, the HIFU beam was aberrated and split by the ultrasound propagation through the aberrators (Fig. 2). The aberrated HIFU fields obtained by time-reversal reconstruction (Fig. 2a and d) and using the beamforming reconstruction (Fig. 2b and e), closely matches the reference field obtainedby hydrophone measurement (Fig. 2c).

**CONCLUSIONS** The results indicated the feasibility of the method, although reconstruction errors were observed, arising from the finite aperture size of the probe or from large path lengths from the source. Despite this limitation, the proposed method may be acceptable as an easy, simple, and real-time HIFU beam imaging techniqueused for therapy guidance if the HIFU emitter and its receiver can be located in a proper face-to-face orientation.

**Fig. 1 (abstract O20).**
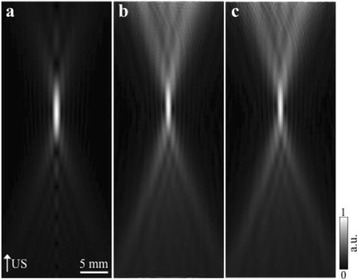
HIFU amplitude field in a lossless medium (water). The horizontal and vertical axes are respectively x­ and z­axes. Each image is normalized by maximum amplitude. (a)Numerical calculation *via* forward propagation using Rayleigh integral. (b) Experimental acquisition using backward reconstruction using Rayleigh integral. (c)Experimental acquisition using backward reconstruction using beamforming

**Fig. 2 (abstract O20).**
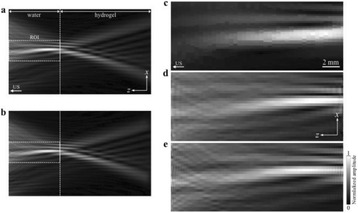
HIFU amplitude field with aberrations obtained experimentally. Each image is normalized by maximum amplitude. (a) Wide­view image obtained by backward reconstruction using Rayleigh integral. (b) Wide­view image obtained by backward reconstruction using beamforming. (c) Reference image obtained by hydrophone measurements in the ROI of 8 × 20 mm2. (d) Reconstructed image *via* Rayleigh integral in the ROI. (e) Reconstructed image *via* beamforming in the ROI

### O21 Low-intensity ultrasound extends lifetimes of transplanted mesenchymal stromal cells in murine muscle

#### Scott R. Burks, Matthew Nagle, Saejeong Kim, Joseph A. Frank

##### Radiology and Imaging Sciences, NIH Clinical Center, Bethesda, Maryland, USA

##### **Correspondence:** Scott R. Burks

**This abstract is not included as it has already been published:**

Burks S, Nagle M, Kim S, Milo B, Frank J. A90 Low-intensity ultrasound prolongs lifetimes of transplanted mesenchymal stem cells. J Ther Ultrasound. 2016; 4(Suppl 1):31. Available from: https://jtultrasound.biomedcentral.com/articles/10.1186/s40349-016-0076-5

### O22 Impact of microbubble-enhanced radiofrequency ablation of rabbit liver

#### Zhong Chen, Xueyan Qiao

##### Department of Ultrasound, Xinqiao Hospital, The Third Military Medical University, Chongqing, China

###### **Correspondence:** Zhong Chen

**OBJECTIVES** To investigate the synergistic effect of combining microbubble-enhanced ultrasound (MEUS) and radiofrequency ablation (RFA) on normal rabbit liver.

**METHODS** Eighteen surgically exposed rabbit livers were treated with MEUS immediately followed by RFA. The other 18 livers were treated with RFA alone. The therapeutic ultrasound of MEUS was operated at a pressure amplitude of 4.3 MPa and a duty cycle of 0.22%. Contrast-enhanced ultrasound (CEUS) was used to evaluatethe liver circulation. Twenty livers were removed for volume measurement of ablation and the rest livers were also harvested for histological examination 24h after treatment. Serum ALT and AST levels of 10 rabbits were examined to monitor any changes of liver function.

**RESULTS** MEUS treatment for 5 min successfully shut down or reduced the blood perfusion of rabbit livers. The average peak intensity (PI) of CEUS dropped significantly from 84.0±4.3% to 49.3±5.1%. The average volume of ablation zone (4.55±0.83 cm3) treated by MEUS plus RFA combination was significantly bigger than that of RFA treatment alone (1.63±0.29 cm3) (Fig. 1). The levels of serum ALT and AST did rise after both of two treatments but recovered to normal eight days later (Figs. 2, 3).

**CONCLUSIONS** Liver RFA can be greatly enhanced by combining MEUS pretreatment.

**Fig. 1 (abstract O22).**
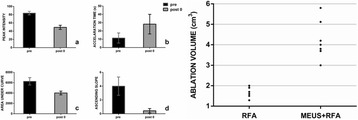
See text for description

**Fig. 2 (abstract O22).**
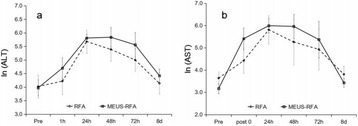
See text for description

**Fig. 3 (abstract O22).**
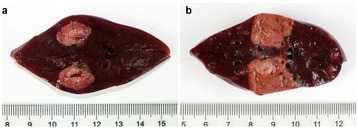
See text for description

### O23 *In vitro* and *in vivo* investigation of high intensity focused ultrasound (HIFU) hat-type ablation mode

#### Hongya Dai

##### Biomedical Engineering, Chongqing Medical University, ChongQing, ChongQing, China

**This abstract is not included as it has already been published:**

Dai H, Chen F, Yan S, Ding X, Ma D, Wen J, Xu D, Zou X. In Vitro and In Vivo Investigation of High-Intensity Focused Ultrasound (HIFU) Hat-Type Ablation Mode. Med Sci Monit. 2017; 23:3373-3382. Available from: https://www.medscimonit.com/abstract/index/idArt/902528 DOI: 10.12659/MSM.902528

### O24 Image-based predicition of focusing gain *in situ* using dual-mode ultrasound arrays

#### Brogan T. McWilliams, Dalong Liu, Emad S. Ebbini

##### Electrical Engineering, University of Minnesota, Minneapolis, Minnesota, USA

###### **Correspondence:** Brogan T. McWilliams

**OBJECTIVES** We have previously described a dual-mode ultrasound array (DMUA) system for therapeutic image-guided focused ultrasound (IgFUS) applications. This system has been shown to provide closed-loop spatiotemporal control of FUS beams with 2 millisecond and submillimeter time and spatial resolutions, respectively. With appropriate feedback (e.g. temperature) this system offers the promise of delivering precision HIFU therapy, which would improve the safety and efficacy of IgFUS procedures. A key performance parameter for HIFU therapy is the focusing gain, which is a characteristic of the transducer (array). The imaging capability of the DMUA allow for the estimation and characterization of the precise imaging path of the HIFU beam through the bolus and intervening tissue to HIFU target. The objective of this paper is to validate an image-based approach for the estimation of the heating rate *in situ* utilizing simplified propagation models and calibration power measurements.

**METHODS** In this study, we used a 3.5-MHz, 64-element DMUA prototype with concave aperture (40-mm radius of curvature with f# = 1). This prototype was used for generating the HIFU beam as well as collecting the corresponding imaging data. Figure 1 shows the *in vitro* setup used to provide a validation for the image-based approach. In particular, an acoustic power meter (Omega, Ohmic instruments Easton, MD) was modified to allow the measurement of the insertion loss due to a slab of tissue-mimicking phantom between the DMUA and the tip of the cone (treated as the target). The TM phantom was fabricated from animal skin bovine gelatine, graphite,1-propanol, glutaraldehyde, and deionized water. Absorption of the phantom is predominately due to the presence of graphite and was determined to be 0.6 and 1.0dB/cm/MHz for two 4-mm disk-shaped slaps. Two modes of the imaging were used to characterize the FUS beam propagation through the tissue: 1) Synthetic-aperture(SA) imaging, which provides larger field of view (FoV) to characterize the propagation medium, and 2) Single-transmit focus (STF), which provides specific feedback about the interactions between the FUS beam and the tissue in its path to the target. The STF imaging is performed using the same beamforming parameters of the intended therapeutic HIFU beam, but at diagnostic levels and with sub-microsecond pulse duration. HIFU was applied at 4 different frequencies (2.4 to 4.2 MHz in stepsof 0.6 MHz). HIFU shots of 1-sec durations were used and repeated 4 times. SA and STF images were collected before, during and after the application of therapeutic HIFU. The STF frame rate was 400 fps, which was helpful to fully characterize the incidence of cavitation and/or instability in the power measurement.

**RESULTS** Figure 2 shows SA and STF images with and without the phantom slab in place. From Fig. 2.b an approximated dimesion of the beam at the geometric focus was determined by the -6 dB contour to be approximately 0.3 x 0.5 mm. Furthermore, using a phantom that results in speckle reflections shown in Fig. 2.d) the beam dimensions can be observed as it traverses through the phantom. Figure 3.c) shows the thickness of the phantom to be about 4mm and ending at approximately 39mm. Figure 3 shows the average (n=4) precentage of power absorbed by the respective phantom disks and validates the approach described in methods at variousfrequencies. The calculated and measured power absorption show similar trends with respect to frequency. Figure 4 shows an SA image of a rat's left carotid artery *in vivo*from an image-guided targeting experiment performed using the DMUA prototype described above. The skin interface was identified in imaging to be approximately 34mm. Based on the acoustic power delivered and using reported numbers of acoustic properties of muscle the calculated heating rate was 9629°C/s. This is consistent with imaging data of the delivered therapy exhibiting a bubbles from boiling conditions within <100 ms.

**CONCLUSIONS** An image-based method for real-time estimation of the focusing gain using DMUA imaging data was validated *in vitro* at multiple frequencies. The method has been applied for the estimation of the focusing gain *in situ* from real-time DMUA imaging data *in vivo*. As described, STF imaging of the phantom slab allowed for measuring the beam dimensions and the FUS interaction with the tissue and could be used in future studies to extract details of an inhomogeneous medium to provide accurate estimates of the focusing gain (or heating rate). Further validation of the calculated heating rate will be performed *in vitro* and *in vivo* by measuringtemperature with thermocouples in the vicinity of the focus.

**Fig. 1 (abstract O24).**
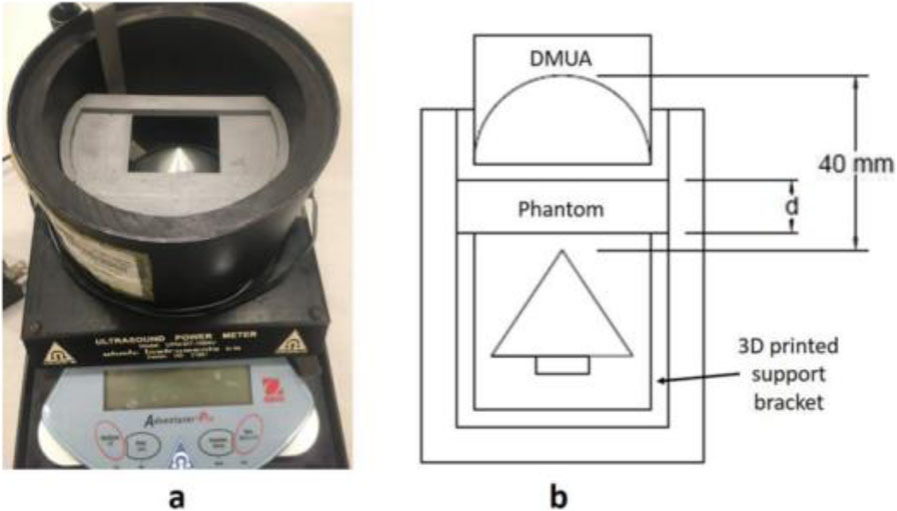
a) shows a digital photo of the power meter with the 3D printed support bracket and slot placed into the water tank. b) shows a cross section along the center toshow orientation of the DMUA and phantom with respect to the cone. the length d is adjustable for disk1 and 2 this length was set to 4 mm

**Fig. 2 (abstract O24).**
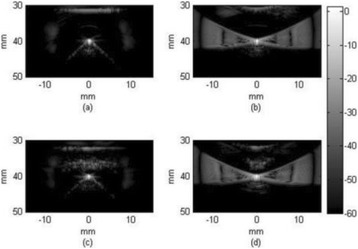
(a) SA image of the power meter without the phantom slab. (b) STF image of the power meter without the phantom slab. (c) SA image of the power meter with the phantom slab. (d) STF image of the power meter with the phantom slab

**Fig. 3 (abstract O24).**
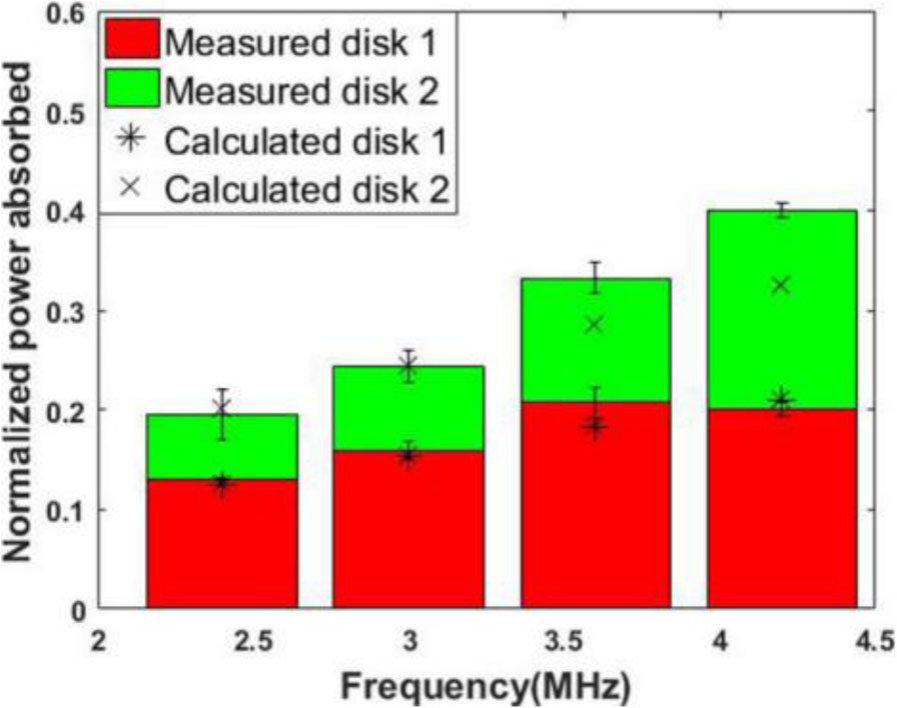
Percentage of power in therapy shot absorbed by phantom slab. Disk 1 and 2 corresponds to 0.6 and 1.0 dB/cmMHz respectively and are both a thickness of 4mm

**Fig. 4 (abstract O24).**
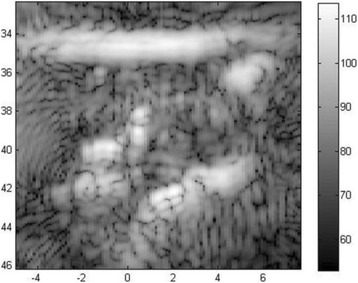
SA imaging of therapy plane with carotid body *in vivo*

### O25 An automatic approach to lesion planning for robotic HIFU

#### Tom Williamson, Scott Everitt, Ranjaka De Mel, Sunita Chauhan

##### Mechanical and Aerospace Engineering, Monash University, Melbourne, Victoria, Australia

###### **Correspondence:** Tom Williamson

**OBJECTIVES** High intensity focused ultrasound (HIFU) based ablation has been found to be predictable and successful for treatment of ‘carcinoma *in situ*’ in variousorgans. At this stage, the neoplasm is generally spherical in shape while, conventionally, focused ultrasound (FUS) treatment involves ‘raster’ scanning the formation ofthe HIFU lesions in the ROI, an approach that will generally not conform to the spherical tumor geometry. This may lead to two major undesirable effects: large gaps or overlaps at the tumor margins (physical spacing isn’t optimized) and between individual lesions, or significant ‘lesion-to-lesion’ interaction creating uncertainty in the shape, size and extent of the subsequent lesions due to the remnant effect of previously laid lesions. Furthermore, the gaps between adjacent lesions may have a seeding effect, leading to further growth of malignancies due to the untreated sections. To avoid lesion interactions, several authors suggested a pre-defined time delay betweenlesions or change in exposure parameters. The former results in unnecessary treatment delays while the latter involves capacious real-time computations for optimizing the treatment (dynamically computing thermal dose) as well as remotely and frequently switching on/off high power equipment. In order to overcome these deficiencies, we propose a method for determining the optimal lesion arrangement within any arbitrary tumor size and shape, based on an extension of the bubble packing algorithm first described by Shimada in 1995 [1]. The original algorithm was extended to allow lesions to take any arbitrary position andorientation within the specified tumor volume, and evaluated on spherical and ellipsoidal tumor models.

**METHODS** The bubble packing algorithm first described by Shimada was extended to the case of arbitrary tumor position and orientation. The existing approach utilizes a model in which overlapping lesions apply forces to adjacent lesions until an equilibrium is reached. Additional forces are applied at the boundary of the tumor to ensure lesions remain within the tumor volume. In this work, the model has been extended such that in addition to the force, a torque is applied resulting in changes in lesion orientation and allowing the optimization of lesion configuration in all six dimensions. Initial evaluation of the technique was performed based on two simplified tumor models. While calculating and fitting the lesions, we assumed our HIFU probe focus as a3×7×3mm ellipsoidal volume and a 20mm diameter spherical target tumor volume, and a 10×5×5 mm ellipsoidal volume. As lesion placement may be constrained to a single acoustic window located some distance from the organ, the case of fixed lesion orientation was also considered as well as the free orientation and traditional raster approach. During raster lesion placement, lesions were generated at evenly spaced intervals throughout the tumor and surrounding volume; those lesions of which the centroid fell outside the tumor volume were then excluded. To allow direct comparison of the approaches, the number of lesions in the optimized cases was set to the same as that utilized for the raster case. In each case Monte-Carlo were utilized to estimate the total portion of tumor tissue destroyed, as well as the total volume overlap and volume of healthy tissue (i.e. that outside of the defined tumor region) ablated. Points were randomly generated within a domain covering twice the tumor volume, with those falling within the targeted region but none of the lesion sites considered as untreated, those within both the tumor volume and one (or more) lesion volume considered as treated, while those outside of the tumor volume but within one or more of the lesions considered as ablated healthy tissue. The optimization process was completed 10 times for each configuration.

**RESULTS** The modified bubble packing approach was successfully implemented in C++ and all described evaluation cases successfully performed. The achieved results demonstrate that the introduction of the bubble packing approach leads to an improvement of approximately 10% in total treated volume as compared to the raster approach, as well as a decrease in ablated healthy tissue of up to 20% (Fig. 1, Table 1). Slight differences in the free and fixed orientation cases, specifically increases in overlap and decreases in ablation of healthy tissue were observed with the introduction of orientation into the optimization framework.

**CONCLUSIONS** This work described the development and evaluation of a general automated method for the treatment planning of lesion positions during HIFU. Theapproach allows the determination of optimal lesion locations and orientations, and can be applied to arbitrary tumor shapes and sizes as well as arbitrary lesion configurations. Evaluation of the approach on spherical and ellipsoidal tumor models demonstrated the feasibility of the approach.

**Fig. 1 (abstract O25).**
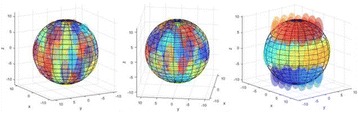
See text for description

**Table 1 (abstract O25).**
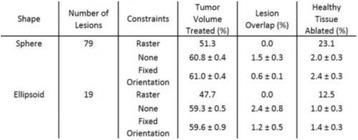
Summarized results of algorithm evaluation

### O26 Extracorporeal high-intensity focused ultrasound treatment of the placental unit: *in vivo* study using a monkey model of pregnancy

#### David Melodelima^1^, Jonathan Caloone^1^, Anthony Kocot^1^, Cyril Huissoud^2^

##### ^1^LabTAU - U1032, INSERM, Lyon, Rhône Alpes, France; ^2^CHU Croix Rousse, LYON, France

###### **Correspondence:** David Melodelima

**OBJECTIVES** Fetal surgery represents an interesting approach for several fetal diseases, particularly for the treatment of twin-to-twin transfusion syndrome (TTTS). Although fetoscopy increases survival rate, it is also considered invasive and responsible for fetal and maternal complications that can affect neonatal outcome. These complications are partially associated with the opening of the amniotic cavity. A completely non-invasive treatment that could occlude deep anastomoses would prevent the risks of invasive fetoscopy while offering the potential for more effective therapy. The efficacy of high-intensity focused ultrasound (HIFU) is clinically proven for non-invasive tissue ablation. We previously developed a toroidal HIFU transducer that enables the destruction of large tissue volumes. In a recent study, we demonstrated the ability to induce lesions in human placenta using this toroidal-shaped transducer without damaging intervening tissues within an *ex vivo* model. The effectiveness of this HIFU device applied to the perfused placental unit must be studied in a preclinical animal study under conditions similar to those in humans before starting a clinical trial. Here, we report a feasibility study using a monkey model of pregnancy. The 3 objectives of this work were (i) to evaluate the feasibility and reproducibility of HIFU lesions in the placenta of pregnant monkeys *in vivo*; (ii) to study the lesions using ultrasound images, gross pathology, and microscopy; and (iii) to evaluate local andgeneral tolerance to the treatment.

**METHODS** A toroidal HIFU transducer working at 2.5 MHz and composed of 32 ring-shaped emitters was used. An ultrasound probe working at 7.5 MHz was placed in the center of the HIFU transducer. The imaging plane was aligned with the HIFU acoustic axis. The acoustic parameters used during HIFU exposures were selected according to preliminary simulations taking into account the attenuation coefficient of placentas, measured previously. In vivo experiments were then performed. Eight pregnant monkeys were exposed to HIFU treatment after anesthesia. Lesions on placental tissues were performed non-invasively by placing the HIFU probe on the skin. Fetal and maternal parameters, such as maternal heart rate, fetal heart rate, and subcutaneous and intra-amniotic fluid temperature, were recorded during HIFU exposure. A C-section was performed immediately after insonification to extract the placenta, inspect the fetus and inspect the maternal abdominal cavity. Placental HIFU lesions were studied using ultrasound images, gross pathology and histology.

**RESULTS** The average gestational age of the animals was 72±4 days. Thirteen HIFU exposures were performed on placentas. The parameters used in this study wereacoustic powers of 65, 80, 110 and 120 W applied for 30, 15, 20 and 20 seconds, respectively. This gradual increase in the total energy delivered was used to determine aset of parameters to create reproducible lesions in the placentas without any complications. Six placental lesions were observed with average diameters of 6.4 ± 0.5 mmand 7.8 ± 0.7 mm and an average depth of 3.8 ± 1.5 mm. Ultrasound images revealed a hyperechoic region that was well correlated with the macroscopic analyses of the HIFU lesions. Necrosis of placental tissues exposed to HIFU was confirmed with macroscopic and microscopic analyses. No significant variation in maternal and fetalparameters was observed during HIFU exposure. Histological examination demonstrated coagulative necrosis within the core of the lesion. Terminal villi were split with trophoblast desquamation, mesenchymal involution and fibrinoid deposits containing fragmented red blood cells. Congestion and hemorrhagic villitis were seen at theborder of the lesion. No damage was seen in the chorionic vessels wall or endothelial cells. There was no inflammation.

**CONCLUSIONS** This study demonstrates in a monkey model of pregnancy the feasibility of HIFU treatment applied to the placenta for potential application to treattwin-to-twin transfusion syndrome. We report here the first use of a completely non-invasive treatment of the placenta in pregnant animals. The primary aims of this study were to assess the efficacy and safety of this technique for the pregnant monkey and fetus. The presence of well-defined and controlled lesions in the placenta without complications was encouraging. Five HIFU lesions were created in the anterior placenta, and one was created in the posterior placenta. The insonification of the posterior placenta was possible without any complications, but the risk to the fetus is much more important.

### O27 Reasons of different therapeutic efficacy of HIFU ablation for uterine fibroids with different MRI-T2W signal: thermal, acoustic and histopathological properties study

#### Faqi Li, Haoran Huang, Jianbo Ran, Zonggui Chen, Man Luo, Fei Li, Qi Wang, Jie Xu, Huan Liu, Zhibiao Wang

##### College of Biomedical Engineering, State Key Laboratory of Ultrasound Engineering in Medicine Co-founded by Chongqing and the Ministry of Science and Technology, Chongqing Key Laboratory of Ultrasound in Medicine and Engineering, Chongqing Medical University, Chongqing, China

###### **Correspondence:** Faqi Li

**OBJECTIVES** To explore the causes of the differences in the therapeutic efficacy of HIFU ablation for uterine fibroids with different T2-weighted MRI signals from the perspective of thermal, acoustic and histopathologic characteristics.

**METHODS** Totally 47 uterine fibroid specimens were collected after surgery. According to the preoperative T2-weighted MRI signal, the uterine fibroids were classified into four categories, which were hypo-intense, isointense, heterogeneous hyper-intense and homogeneous hyper-intense. The T2-weighted MRI signal of preoperative uterine fibroid was analyzed quantitatively to the signal intensity. Part of the uterine fibroids specimens were undergone the sound velocity, sound attenuation, specific heat capacity and thermal conductivity measurement, and others were used to analyze the content of tissue smooth muscle cell (SMC) and collagenous fiber (CF) by histopathological observation. HIFU irradiation was made in the uterine fibroids specimens. Besides, with the clinical application and follow-up survey, the therapeutic effect of HIFU treatment for the uterine fibroids was analyzed retrospectively.

**RESULTS** There were the differences among the sound velocity, sound attenuation, specific heat capacity and thermal conductivity of the four groups of uterine fibroids, as well as the content of tissue smooth muscle cell and collagen fiber. The therapeutic efficacy has significant differences in four groups of uterine fibroids, the groups from the hypo-intense to homogeneous hyper-intense, the difficulty of HIFU ablation have been gradually increased. There were correlations between the signal intensity, acoustic, thermal properties, histopathological features and therapeutic efficacy of HIFU ablation for the uterine fibroids of different T2-weighted MRI signal.

**CONCLUSIONS** There are some differences in the sound velocity, sound attenuation, number of SMC and CF content among the uterine fibroids of different MRIT2WI signal, which may be one of the important factors to different therapeutic efficacy of HIFU ablation. (This study was supported by the National Natural ScienceFoundation of China (Grant Nos. 81127901, 11574039, 11274404))

### O28 Image-guided ablation of the carotid body *in vivo*: a potential noninvasive treatment for hypertension

#### Dalong Liu^2,1^, Raj Aravalli^1^, Emad S. Ebbini^1^

##### ^1^Electrical and Computer Engineering, University of Minnesota, Minneapolis, Minnesota, USA; ^2^Siemens, Seattle, Washington, USA

###### **Correspondence:** Dalong Liu

**OBJECTIVES** The carotid body (CB) is a neurovascular structure located near the carotid bifurcation. It is an arterial chemoreceptor, which produces neural output reflecting the partial pressure of O2 and CO2 as well as pH and temperature. The CB chemoreflex-evoked sympatho-excitatory responses are known to be enhanced in both human patients and animal model of hypertension. The main aim of this study is to establish the feasibility and safety of localized ablation of the carotid body usingimage-guided focused ultrasound (IgFUS) in normotensive and hypertensive rat models.

**METHODS** Normotensive and hypertensive rats (275 - 330 gm) were treated using our dual-mode ultrasound array (DMUA) system for IgFUS using IACUC-approved protocol. In each experiment, the rat was placed in supine on stereotaxic stage with a heating pad placed in between to regulate body temperature. The hair was shaved and removed using depilatory cream. The DMUA was positioned using a 3 stage motor under real-time imaging guidance. Synthetic-aperture (SA) Imaging was performed to locate the carotid artery bifurcation based on a 3D scan of the treatment volume containing the target CB. The vessel pulsation was used to track the common (CCA), external (ECA) and internal (ICA) arteries in each plane. Once the carotid bifurcation plane was determined, four (4) treatment planes were definedoffset by 0.5, 1, 1.5 and 2 mm from the bifurcation in the caudal-to-rostral direction. In each plane, IgFUS was delivered at 2 - 6 locations around the ECA using a closed loop dose control (CLC) algorithm based on real-time DMUA imaging feedback (described previously at ISTU2015). Briefly, the echogenicity changes indicative of cavitation and/or tissue boiling are detected using DMUA imaging at up to 500 frames per second. These changes are used to adjust the HIFU exposure from frame to frame to avoid over-exposure to the target. The locations of the HIFU shots were chosen to assess the nature and extent of damage due to a "prescribed HIFU dose" under CLC in and around the target volume, including muscle and connective tissues. For each shot, the initial *in situ* HIFU intensity was between 5 - 10 kW/cm2, but this was quickly adjusted down by the CLC upon detection of the echogenicity change in beamformed DMUA imaging data. The exposure time was 0.5 sec for each shot with minimum 40 ms at the initial intensity to ensure the echogenicity change is due to HIFU-induced change. Animals were survived for 48 - 96 hours after the treatment to allow for histological evaluation of HIFU-induced lesions (H&E) and the affected neural structures (Chromagranin A). Histology sections corresponding to the DMUA imaging slices during treatment were produced (50 μm per section covering the treatment volume.)

**RESULTS** DMUA imaging data was collected before, during and after each HIFU shot at frame rates between 200 - 500 fps to document the lesion detection capabilitiesof the real-time guidance system. The system also logged the instantaneous intensity values used by the CLC algorithm. Figure 1 shows an example guidance and monitoring display from our DMUA system. The false color overlay represents the echogenicity change due to a HIFU shot placed just to the left of the right ECA nearthe bifurcation. The spatiotemporal maps (temporal-axial and temporal-lateral) demonstrate the high degree of localization of the echogenicity change in DMUA imaging. The line profiles show the echogenicity change (in dB) at three points corresponding to the target (red), vessel wall (blue) and skin (orange). Figure 2 shows a typical H&E histology slide showing several discrete HIFU-induced shots (arrow heads) around the ECA.

**CONCLUSIONS** The results confirmed our ability to precisely control the characteristics of HIFU-induced lesions very close to the vessel walls without damaging the endothelium, i.e. no danger of vessel perforation. Furthermore, the results confirmed that CLC was essential to avoid overexposure due to the heterogeneity of the tissues in the target volume with significant variation in local absorption. open-loop control results, not shown, often resulted in either overexposure or underexposure depending on the value of the initial HIFU intensity. The results also demonstrated the safety of the procedure as all the animals tolerated this procedure well (over 32 animals at thetime of writing). Overall, the results show the feasibility of delivering prescribed levels of HIFU exposure to extremely small neurovascular structures with potential application in the treatment of hypertension, targeted neuromodulation and other image-guided interventions. Based on the results reported herein, a proof-of-concept study in 56 normotensive and spontaneously hypertensive rats is currently under way.

**Fig. 1 (abstract O28).**
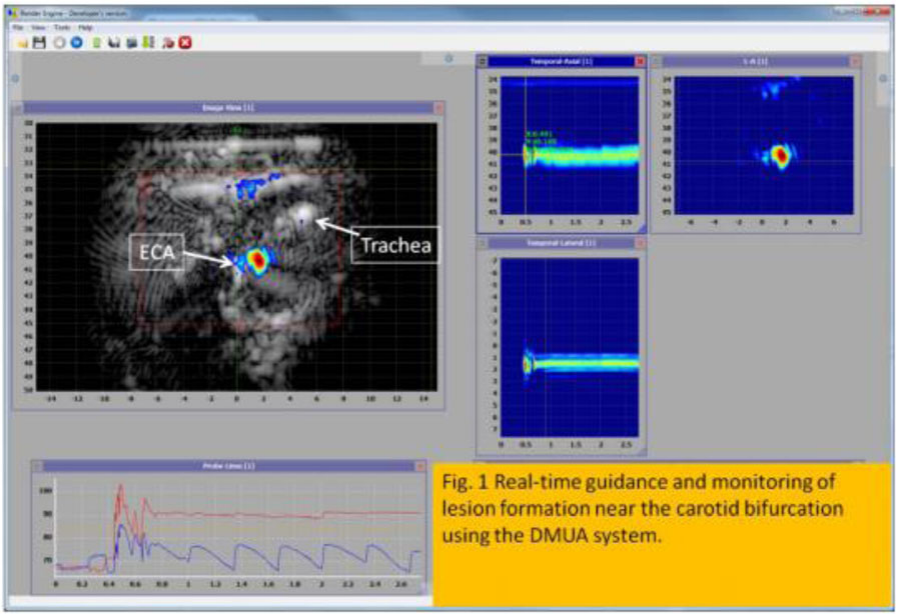
See text for description

**Fig. 2 (abstract O28).**
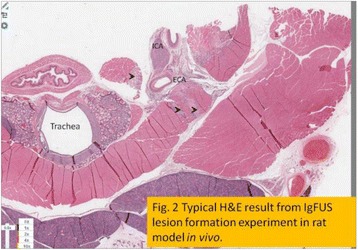
See text for description

### O29 Computer-aided transcranial ultrasound for time-efficient blood-brain barrier opening in primates

#### Shih-Ying Wu^1^, Christian Aurup^1^, Carlos J. Sierra Sánchez^1^, Julien Grondin^1^, Wenlan Zheng^1^, Vincent Ferrera^2^, Elisa E. Konofagou^1,3^

##### ^1^Biomedical Engineering, Columbia University, New York, New York, USA; ^2^Neuroscience, Columbia University, New York, New York, USA, ^3^Radiology, Columbia University, New York, New York, USA

###### **Correspondence:** Shih-Ying Wu

**OBJECTIVES** Focused ultrasound (FUS) with microbubbles holds great promise to noninvasively treat central-nervous-system diseases such as Parkinson’s and Alzheimer’s disease through blood-brain barrier (BBB) opening and enhanced delivery of pharmaceuticals. While research has shown the need of repetitive application for treatment efficacy, currently there exists no clinical system designed to satisfy the requirement. In fact, the commonly-used magnetic resonance-guided FUS (MRgFUS) system hinders the re-application owing to its low speed, flexibility, and high cost due to the dependence upon the MRI scanner. Therefore, in this study, a time-efficient transcranial FUS and monitoring system has been developed using computer-aided neuronavigation without requiring an online MRI, with the protocol from in silico planning, real-time targeting and monitoring, to post-treatment assessment evaluated in non-human primates *in vivo* in preparation for clinical trials.

**METHODS** An arm-free, real-time neuronavigation system designed for primates (both monkeys and humans) was customized to be used for FUS application and monitoring purposes. The ultrasound system consisted of a FUS treatment unit controlled by a customized program in Matlab with a single-element, 0.5-MHz FUS transducer triggered by a function generator after 50-dB amplification, and an acoustic monitoring unit with a programmable acoustic signal acquisition system and an array of acoustic detectors synchronized with the FUS system for real-time passive acoustic mapping and storage of the entire acoustic signals. Both the FUS and acoustic mapping were guided with the neuronavigation system during the FUS procedure. The system was tested in 4 rhesus macaques with BBB opening in both sedate (animal under anesthesia lying prone on an operating table) and awake setups (animal trained to sit in a customized chair), and the demonstrated protocol covered from in silico preplanning and simulation, real-time targeting and acoustic mapping guided by neuronavigation, to post-treatment assessment of BBB opening effectivenss and safety in the MRI including contrast-enhanced T1-weighted imaging, T2-weighted imaging, and susceptibility-weighted imaging (SWI).

**RESULTS** Simulation revealed inter-animal variation (21%) due to varying skull density and thickness and can be compensated before sonication to ensure treatment reproducibility. In the *in vivo* experiments, for the first time the noninvasive FUS treatment was achieved within 30 min outside the MRI system for primates, and targeting flexibility allowed BBB opening in both the peripheral cerebral cortex and deeply-seated subcortical structures with the use of a free-guide arm and the inflatable bladder system of the FUS transducer to couple with the scalp. Moreover, the achieved targeting accuracy were proximal to the predicted 2-mm limit in simulation. The accuracy in the awake animal setting (3.0 mm) was found to be comparable to the sedate animal setting (3.2 mm), which was higher compared to the frame-based stereotaxis due to the improvement of lateral positioning of the animal, and the focal shift in the acoustic beam path was due to the skull distortion. On the other hand, real-time cavitation mapping was performed with neuronavigation guidance during the entire sonication, with the frequency spectra data showed a dramatic increase of the cavitation signal (harmonics and ultraharmonics) after injecting microbubbles. The acoustic mapping provided real-time spatial monitoring during the sonication and successfully confirmed the location of BBB opening. Finally, in all the experiments performed, no acute damage such as hemorrhage (SWI) or edema (T2-weighted imaging) was detected upon radiologic examination 2 h after sonication.

**CONCLUSIONS** This computer-aided system developed enabled rapid and flexible transcranial FUS applications for primates outside of the MRI scanner without the use of a stereotactic frame. It will greatly facilitate both preclinical and clinical use for BBB opening and drug delivery to treat neurodegenerative disease and brain tumors.

### O30 Rapid short-pulse (Rasp) sequences – A new therapeutic ultrasound exposure paradigm to enhance drug delivery to the brain *in vivo*

#### Sophie Morse, Antonios Pouliopoulos, Tiffany Chan, Julien Lin, James J. Choi

##### Bioengineering, Imperial College London, London, UK

###### **Correspondence:** Sophie Morse

**OBJECTIVES** One third of the worldwide disease burden is caused by brain diseases, such as dementia, Parkinson’s and brain cancer. Despite efforts to develop new treatments, there remains no cure for these diseases. A major reason for the lack of therapies is that most drugs cannot enter the brain, because they are blocked by the blood-brain barrier (BBB). A promising solution uses focused ultrasound and microbubbles to noninvasively and locally increase the BBB permeability so that drugs can enter the brain. This technology has successfully delivered a wide range of drugs, such as antibodies and nanoparticles, and is currently being tested in human patients for the treatment of brain cancers. Despite encouraging results, the current technology generates a poor drug distribution and too much toxicity and damage for use in other brain diseases, such as dementia, which affects 46 million people worldwide. Our group has recently shown *in vitro* that these limitations may be due to the conventional ultrasound sequences used to disrupt the BBB. These sequences consist of long-pulses emitted at a slow rate and generate a mixture of both desired and undesired cavitation activities. We have recently developed and tested a new low pressure rapid short-pulse (RaSP) sequence *in vitro*, designed to promote the desired cavitation activity in the correct locations (e.g., capillaries). This new sequence is evaluated here for its ability to improve the *in vivo* efficiency and safety of ultrasound-mediated drug delivery to the brain.

**METHODS** Rapid short-pulse (RaSP) sequences consist of short pulses separated by off-time intervals in the range of μs. In vitro studies have shown that these sequences prolong the lifetime of microbubbles and increase their mobility during the off-time intervals, allowing lightly stimulated microbubbles to freely distribute throughout the capillaries, which are the target microstructures of drug transfer. The better spatial and temporal distribution of microbubble activity allows a more uniform enhancement of the BBB permeability and therefore a more uniform distribution of the delivered drug. We tested here whether RaSP sequences used *in vivo* in C57BL/6 mice would improve the efficiency and safety of brain drug delivery. We compared our RaSP sequence (peak-negative pressure: 400 kPa; pulse length (PL): 5 cycles; pulse repetition frequency (PRF): 1.25 kHz; burst length: 10 ms) to the current gold standard, conventional sequence at the same acoustic pressure (PL: 10,000 cycles; PRF: 0.5 Hz; burst length: 10 ms). Fluorescently-tagged (Texas Red) 3 kDa dextran and microbubbles were intravenously injected in mice while sonicating the left hippocampus with a 1 MHz focused ultrasound transducer. A 7.5 MHz passive cavitation detector captured the microbubble acoustic emissions. The relative dose and distribution of the drug were quantified by calculating the normalised optical density (NOD, average increase in fluorescence in the targeted area normalised by the control) and the coefficient of variation (COV, standard deviation over the average fluorescence intensity in the targeted region). Safety was assessed by haematoxylin and eosin (H&E) histological staining.

**RESULTS** Despite emitting 150 times less acoustic energy, RaSP sequences delivered a dextran dose of the same order of magnitude as the long-pulse sequences (Fig. 2). Moreover, the drug distribution was significantly more uniform using RaSP sequences (Fig. 1), as indicated by the coefficient of variation (Fig. 2). Compared to conventional sequences, RaSP sequences produced less arterial effects, such as dextran accumulation in or along the arteries (Fig. 1c). This suggests that RaSP sequences may reduce the likelihood and magnitude of unnecessary treatment of the arteries. Acoustic emissions from our short-pulses were more stable than those from the longpulses, with the energy smoothly decreasing over time. The energy levels of the acoustic emissions from the exposure to long-pulses varied greatly between pulses and within each pulse.

**CONCLUSIONS** These results suggest that RaSP sequences can improve the spatial distribution of dextran delivery to the brain by producing a more uniform distribution of acoustic cavitation activity. Low pressure RaSP sequences could deliver a more efficient and safe dose of drugs across the blood-brain barrier to treat diseases such as Alzheimer’s, Parkinson’s and other neurodegenerative diseases.

**Fig. 1 (abstract O30).**
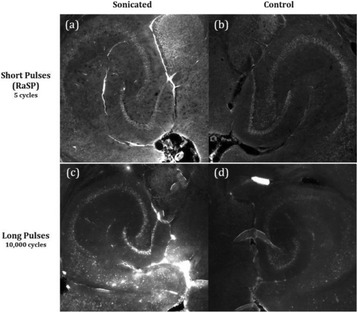
*In vivo* brain drug delivery distributions using (a, b) rapid short­pulse (RaSP) or (c, d) conventional sequences. The (a, c) left hippocampus of the mouse brain was sonicated *in vivo* after systemic delivery of fluorescently­labelled 3­kDa dextran and microbubbles. The (b, d) right hippocampus was not treated and acted as our control (b, d). Sonication using (a) a RaSP sequence delivered a greater dose of dextran to the brain parenchyma with less delivery to arteries than (c) conventional sequences

**Fig. 2 (abstract O30).**
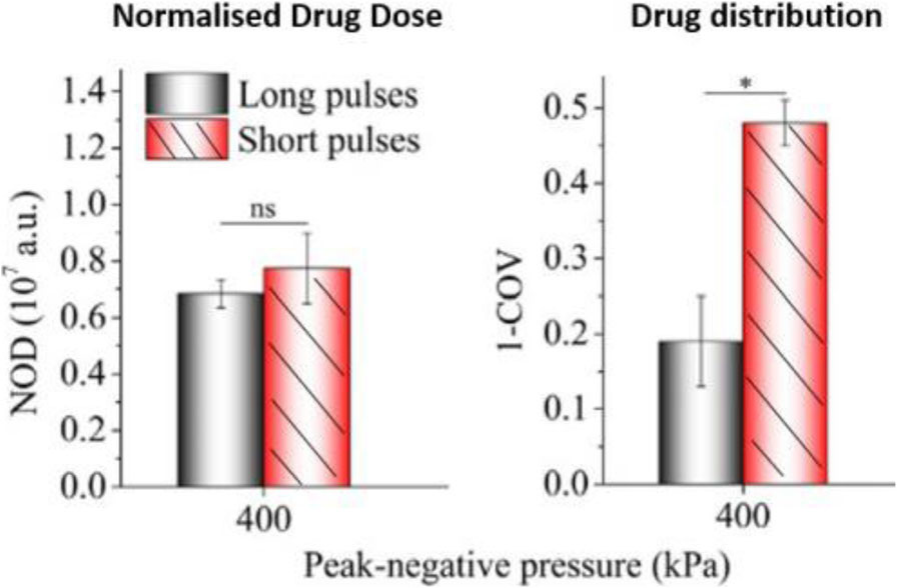
Quantification of the normalised drug dose and drug distribution delivered using RaSP and conventional sequences. The normalised drug dose was quantified with the normalised optical density (NOD), i.e. the average increase in fluorescence in the targeted area normalised by the control. The drug distribution was quantified with the coefficient of variation (COV), i.e. the standard deviation over the average fluorescence intensity in the targeted region (* p < 0.01; ns = not significant)

### O31 Magnetic resonance guided focused ultrasound surgery (MRgFUS) Treatment of osteoid osteoma: a prospective development study

#### Maayan Kimhy, Alessandro Napoli, Marco Marra Marcozzi

##### Dipartimento di Scienze Radiologiche, Oncologiche E Anatomo-Patologiche, Sapienza Università di Roma, Rome, Italy

###### **Correspondence:** Maayan Kimhy

**This abstract is not included as it has already been published:**

Nappoli A, Zaccagna F, Catocci G, Giulia B, Caliolo G, Andrani F, Catalano C. Magnetic resonance guided focused ultrasound surgery (MRgFUS) treatment of osteoid osteoma: a prospective development study. J Ther Ultrasound. 2015; 3(Suppl 1): O44. Available from: https://jtultrasound.biomedcentral.com/articles/10.1186/2050-5736-3-S1-O44

### O32 Transoesophageal HIFU for cardiac ablation: experiments on beating hearts

#### Paul Greillier^1^, Bénédicte Ankou^2^, Ali Zorgani^1^, Francis Bessière^2^, Fabrice Marquet^3^, Julie Magat^3^, Sandrine Melot-Dusseau^4^, Romain Lacoste^4^, Bruno Quesson^3^, Mathieu Pernot^5^, Philippe Chevalier^2^, Cyril Lafon^1, 6^

##### ^1^LabTau - U1032, INSERM, LYON, Rhône, France; ^2^Hôpital Louis-Pradel, Lyon, France; ^3^IHU-LIRYC - CHU Bordeaux, Pessac, France; ^4^Station de primatologie -CNRS- UPS846, Rousset, France; ^5^Institut Langevin - Ondes et Images - ESPCI ParisTech, CNRS UMR 7587, Paris, France; ^6^University of Virginia, Charlottesville, Virginia, USA

###### **Correspondence:** Paul Greillier

**This abstract is not included as it has already been published:**

Greillier P, Ankou B, Bessière, Zorgani A, Pioche M, Kwiecinski W, Magat J, Melot-Dusseau S, Lacoste R, Quesson B, Pernot M, Catheline S, Chevalier P, Lafon C. A75 Trans esophageal HIFU for cardiac ablation: first experiment in non-human primate. J Ther Ultrasound. 2016; 4(Suppl 1):31. Available from: https://jtultrasound.biomedcentral.com/articles/10.1186/s40349-016-0076-5

### O33 In-vivo investigation of the combination of focused ultrasound and radiotherapy, using photoacoustic imaging as aplanning and monitoring tool

#### Marcia M. Costa, Anant Shah, Ian Rivens, Tuathan O'Shea, Carol Box, Jeff Bamber, Gail ter Haar

##### Radiotherapy and Imaging, The Institute of Cancer Research, Sutton, UK

###### **Correspondence:** Marcia M. Costa

**OBJECTIVES** Tumour hypoxia is a limiting microenvironmental factor for radiotherapy (RT) success, since decreased oxygenation renders cells radioresistant, whereashigh intensity focused ultrasound (HIFU) tissue ablation is independent of oxygen levels. Imaging blood oxygenation, which in part measures hypoxia, can be achieved non-invasively using photoacoustic imaging (PAI). The technique relies on the generationof acoustic waves (1-50MHz) by the tissue upon absorption of short pulses of light. These waves can be detected using an ultrasound transducer and an image of relativeoptical absorption, can then be reconstructed providing anatomical information. Furthermore, light is absorbed differently by oxy- (HbO2) and deoxy-haemoglobin (Hb), and it is thus possible to calculate their relative proportions and, consequently, the distribution of oxygen saturation (sO2=HbO2/(Hb+HbO2)) in tissue [1]. In this paper, we describe a combinatorial approach of HIFU and radiotherapy, using the former to target hypoxic (radioresistant) tumour volumes, in order to investigateif this improves treatment outcome. PAI was used for HIFU treatment planning and assessing the treatment ouctcome. Furthermore, the relationship between the pretreatment sO2 values of tumours and radiotherapy response was also investigated.

**METHODS** This study used a subcutaneously implanted human head and neck tumour (CALR) in immunosuppressed mice, which was found to produce relativelyhypoxic tumours at a tumour size suitable for studies. Ultrasound imaging guided HIFU-alone (N=12) and computed tomography imaging guided radiotherapy-alone (N=13) treatments were performed using dedicated small animal platforms: VIFU-2000 (Alpinion, Washington, USA) and SARRP (225KeV manufacturer details, Xstrahl, Camberley, UK), respectively. Tumours had a mean volume of 209 (+/-23 mm3). PAI (Multispectral Optoacoustic Tomography (MSOT), iThera, Munich) was used to measure blood distribution and its oxygen saturation non-invasively with the aim of identifying a main hypoxic tumour region for HIFU exposure. The HIFU treatment regime was a ‘spiral in’ pattern of 6 exposures, ISPTA (free field) = 1.1 kW.cm^-2^ and 10 second duration, per exposure. Exposures were done 1mm apart, in two rows, 3 exposures per row. Uniform whole tumour radiation exposures of 10 Gy, in a single fraction, were used. Animals were followed-up 60 days after treatment, using calliper measurements to calculate tumour volume. Once the treatment responses for individual treatments had been established, HIFU and RT were combined (total N=25), these being delivered 15 minutes apart. Both RTfollowed by HIFU and (N=12) the reverse treatment order (N=13) were investigated.

**RESULTS** The HIFU-alone treatments resulted in 6 animals with no tumour control and another 6 with an average tumour growth delay of 6 (+/-4) days. The resultant hypointense lesion appearance in the Photoacoustic Imaging scan immediately after treatment is shown in Fig. 1B. Surrounding the lesion, is a hyperintense region which spectral analysis showed to have increased haemoglobin content compared to the pre-HIFU imaging (Fig. 1A). It is possible that this increase in haemoglobin signal is due to resultant haemorrhage (vascular disruption) caused by HIFU. Further histological studies are being undertaken to confirm these results. Radiotherapy alone treatments resulted in a 46% treatment response, defined as full tumour remission or growth inhibition within the follow up-period. Spectral analysis of the photoacoustic signal showed a statistically significant difference between the sO2 values of responders (high sO2) and non-responders (low sO2). The combined treatments outcome, compared to the 10Gy-alone, showed an improvement in treatment response both for percentage of responders (32% for RT+HFU and40% for HIFU+RT) and survival (Fig. 2).

**CONCLUSIONS** This preliminary study in a cell line known to be relatively radioresistant has shown a therapeutic benefit from combining hypoxia targeted HIFU with whole tumour, in terms of improved outcome at lower doses. This effect is possibly due to the combination of ablating radioresistant hypoxic areas and using RT for further kill of the cells in better vascularised margins. Furthermore, PAI analysis showed the possibility of using this technique in adapting the treatment planning forradiotherapy in clinic, as a relationship between the sO2 measured values in tumour and the success of treatment was established. This project could have a major positive impact on the treatment of tumours that are characteristically hypoxic, such as those of head and neck, if such combinationtreatments can be translated into clinical practice. [1] Xia et al., Electromagn Waves (Camb). 2014; 147: 1–22.

**Fig. 1 (abstract O33).**
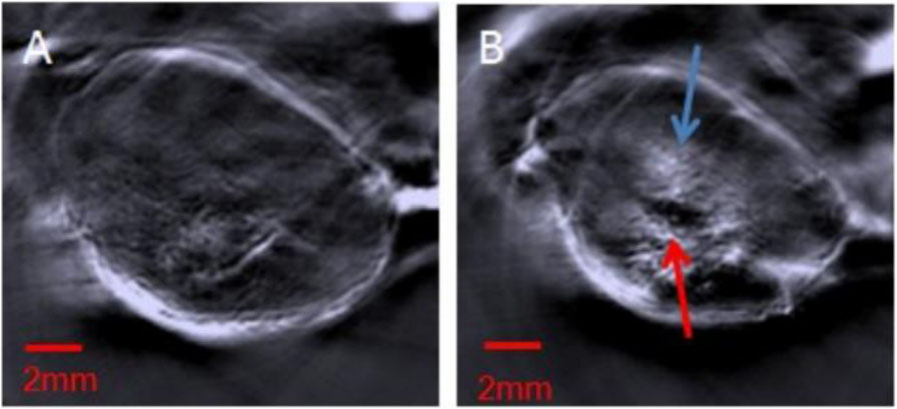
A: Pre-HIFU photoacoustic imaging scan of CAL^R^ tumour. B: Post-HIFU scan showing the lesion (red arrow) and a hypertensity rim (blue arrow)

**Fig. 2 (abstract O33).**
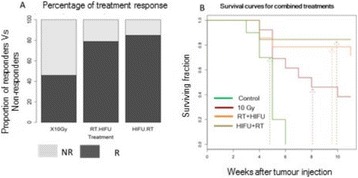
Treatment response A: proportion of responders (R) and non-responders (NR) of 10gy-alone and combined treatments, showing an increase of responders for the latter group. B: Survival curves show an improvement in the overall survival of animals treated with the combined treatment compared to RT treatment alone. Arrows point to the average survival time of each treatment group

### O34 Intracellular vaporization of antibody - conjugated phase - change nano-droplets for selective cancer therapy

#### Ayumu Ishijima^1^, Takashi Azuma^1^, Kosuke Minamihata^2^, Satoshi Yamaguchi^1^, Shinya Yamahira^1^, Etsuko Kobayashi^1^, Mariko Iijima^1^, Yoshikazu Shibasaki^1^, TeruyukiNagamune^1^, Ichiro Sakuma^1^

##### ^1^The University of Tokyo, Tokyo, Japan; ^2^Kyushu University, Fukuoka, Japan

###### **Correspondence:** Ayumu Ishijima

**OBJECTIVES** Recent advances in nanomedicine provide the opportunity to reduce systemic toxicities of chemotherapies. However, drug resistance remains a major challenge in cancer treatment research. Here we developed a nanomedicine composed of a phase-change nano-droplet (PCND)1,2 and an anti-cancer antibody (9E5), proposing the concept of ultrasound cancer therapy with intracellular vaporization to physically treat cancer.

**METHOD**S 9E5-conjugated PCNDs (140 ± 120 nm) consists of a PFC liquid core (a mixture of perfluoropentane and perfluorohexane), a phospholipid shell, and antibody 9E5. The 9E5 human anti-epiregulin (EREG) antibody was selected for active targeting of PCNDs. EREG, the cell-membrane–expressed ligand of epidermal growth factor receptor, is expressed and integrated into the plasma membrane at relatively high levels in a variety of human cancers.3 9E5 was conjugated to PCNDs using the biotin streptavidin-biotin binding technique. Biotinylated-9E5 and Alexa Fluor 647-conjugated streptavidin (SA-AF647) were bound to biotinylated PCND.The human colonic adenocarcinoma cell line DLD1 and the human gastric cancer cell line AGS were selected as high and low EREG-expressing cancer cell lines, respectively. To demonstrate the selective targeting capability of 9E5-conjugated PCNDs to DLD1 cells, we used SA-AF647 as a fluorescence probe. The targeted cells were observed by confocal laser scanning microscopy (CLSM), and the number of PCNDs attached and the fraction of bound DLD1 cells were quantitatively measuredby flow cytometer. Next, PCND-accumulated cells were exposed to ultrasound (100 cycles, 4 MHz, a peak negative pressure of 1.5 MPa), and its cytotoxic effects were visualized. The intracellular vaporization of 9E5-conjugated PCNDs in DLD1 cells was observed by a high-speed imaging system recording 101 subsequent frames at 0.25 Mfps. Furthermore, we quantitatively evaluated the cytotoxic capabilities of vaporized PCNDs using PI staining and flow cytometer after exposure of 5 cycles at 5 MHz of pulsed ultrasound with a peak negative pressure of 4.6 MPa using an ultrasound imaging probe.

**RESULTS** CLSM images of DLD1 cells treated with 9E5-conjugated PCNDs show the fluorescence signal inside the cells after 3 hours of incubation (Fig. 1a), whereas no clear fluorescence signals were observed from the other types of cells. Flow cytometry analysis (N = 5) indicated that the 97.8 ± 0.5% of DLD1 cells were targeted by 9E5-conjugated PCNDs, whereas 1.4 ± 0.3% of DLD1 cells were targeted by non-9E5-conjugated PCNDs, similar to that of the control (4.4 ± 1.6%). Furthermore, the ratio ofAGS cells targeted by 9E5-conjugated and non–9E5-conjugated PCNDs were close to the levels of the control. The intracellular vaporization observed by high-speed imaging revealed that cell membranes were ruptured or broken into several parts during this initial stage ofvaporization (Fig. 1b). High-speed images clearly show that intracellular vaporization caused a significant disturbance in cell morphology and destroyed the cells. The fraction of viable cells after ultrasound exposure was measured by flow cytometry (N = 5). Cell viability was significantly reduced to 43.0 ± 5.6% for 9E5-conjugatedPCNDs-treated DLD1 cells, while there was no significant cell viability decrease for PCNDs without 9E5 conjugation (95.8 ± 2.0%) and without ultrasound exposure. Furthermore, the viability of AGS cells did not decrease (98.0 ± 0.2%). These data indicate that PCND conjugated with 9E5 can sufficiently kill DLD1 cells with high selectivity. The addition of free 9E5 to DLD1 cells before treating/co-treating with 9E5-conjugated PCNDs significantly reduced the number of PI-stained cells (89.5 ±10.2%).

**CONCLUSION** In order to obtain cytotoxicity with droplet vaporization, previous reports combined anti-cancer drugs such as doxorubicin with droplets.4 Our studies revealed that anticancer drug free 9E5-conjugated PCNDs are selectively internalized into targeted cancer cells and kill the cells dynamically by ultrasound-induced intracellular vaporization. In vitro experiments show that 9E5-conjugated PCND targets 97.8% of high EREG-expressing cancer cells and kills 57% of those targeted upon exposure to ultrasound. Furthermore, direct observation of the intracellular vaporization process revealed the significant morphological alterations of cells and the release of intracellular contents. This work was supported by a grant for the TSBMI from the MEXT of Japan. A.I. was supported by a Grant-in-Aid from the JSPS Research Fellows.

**References**

1. Kawabata K et al. Jpn J Appl Phys 2005; 44: 4548.

2. Ishijima A et al. Ultrasonics 2016; 69: 97–105.

3. Lee YH et al. Biochem Biophys Res Commun 2013; 441: 1011–1017.

4. Wang CH et al. Biomaterials 2012; 33: 1939–1947.

**Fig. 1 (abstract O34).**
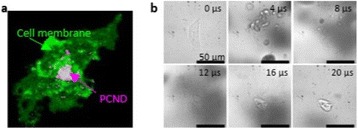
(a) Internalization of AF647­labeled 9E5­conjugated PCND. CLSM observation of 9E5 antibody­mediated accumulation and internalization of AF647­labeled9E5­conjugated PCNDs into DLD1 cells. (b) High­speed imaging of intracellular vaporization

### O35 Dual mode time reversal cavity for US shockwave therapy and 3D imaging

#### J. Robin^1,2^, B. Arnal^1^, M. Tanter^1^, M. Pernot^1^

##### ^1^Institut Langevin, Paris, France; ^2^Université Paris 7, Paris, France

###### **Correspondence:** J. Robin

**OBJECTIVES** The utility of Time Reversal Cavities (TRC) for medical ultrasound has been demonstrated for several applications. They have indeed shown promising results for shock wave therapy, as they allow high intensity pulses focusing in large regions of interest with only electronical steering and a limited number of transducer elements [1, 2]. TRCs have also been used as compact emitter-receivers for pulse echo imaging [3 - 5]. In this work we present a dual mode cavity that can both perform shock wave therapy and 3D imaging in a large region of interest with 128 transducer elements. This dual mode capability is particularly interesting in transcostal applications where part of the therapeutic beam is shadowed by the ribs lowering significantly the focal pressure. 3D volumetric imaging of the ribcage is used to perform an adaptive focusing of the therapeutic beam by transmitting selectively the ultrasonic energy through the ribs. Adaptive focusing results in an increased efficiency and higher pressure at the focal zone.

**METHODS** The leaky TRC is made of 2 orthogonal steel rod forests in a reverberating cavity. The cavity itself is 20*5*5 cm^3^ with steel walls, and filled with water. A high power matrix array transducer (128-elements, 1 MHz, Imasonics, France) is placed in the back of the cavity, opposite the aperture. For shock wave therapy experiments, the probe was driven by custom multi-channel electronics (Corelec, France). 40 μs US pulses emitted through the cavity were spread to up to 1 ms, picked up by a HNC 200 hydrophone (Onda, Sunnyvale, CA) and stored. This process will further on be called calibration step. Time reversal focusing (TRF) by compressing these signals in space and time allowed us to reach the needed pressures. Steering the focal spot over a large volume was achieved by moving the hydrophone (42*42 mm2 area, 1.5 mm grid step). We then re-emitted the reversed signal at a pulse repetition frequency of 260 Hz, targeting an Ultracal phantom to form lesions. For imaging experiments, the probe was driven by a Verasonic HIFU system. The same calibration step was used, but instead of TRF, inverse filter (IF) focusing was used. The focus quality was assessed by hydrophone scanning. The signals corresponding to a focus on each grid point were successively emitted in free field, and the backscattered signals were recorded on the transducers and stored, to constitute a reference library. An object was then placed in the focal plane, and the same process was repeated. For each grid point, the reference was subtracted from the backscattered signals, and an image of the object was reconstructed using the IF signals for focusing on reception. We used 2D simulations (Acel) to evaluate the pressure gain with selective propagation of the therapeutic beam through the intercostal space. Calibration step on grid points in front of the cavity aperture was performed in free space. Reflectors mimicking a rib cage were then added in the propagating medium, blocking part of the aperture. The grid points were used as virtual transducers, and a delay law for a focus behind the ribs was applied. Either all the grid points or only those in the intercostal spaces were used. The total power was the same. Peak pressure was recorded on target in both cases.

**RESULTS** Hydrophone measurements showed the device could create thin isotropic focal spots in a 200 cm^3^ region of interest, with very limited decrease of the peak pressure Therapy: Targeting the Ultracal® phantom in 7 different positions, we formed clear isotropic lesions in a 2.5x4 cm^2^ region, with only electronical steering. Imaging: The focal spots obtained with IF focusing showed central lobes with a 2 λ wide - 6 dB area, and a global -20 dB contrast. Side lobes were visible in one direction, but remained 10 dB below the main focus. We imaged either 2 steel wires, or 2 human ribs. In both cases, the object was clearly visible on the final image. The imaging window (4x4cm^2^) coincides with the device aperture. Coupling imaging and therapy: Simulations showed a 21 % increase in the focal peak pressure when turning off the virtual transducers placed in front of the ribs

**CONCLUSIONS** We built a dual mode TRC that can both perform US shock wave therapy and create a 3D image of the surrounding medium. Though the image resolution is quite low due the low frequency used, it is sufficient to visualize strongly reflecting structures like the rib cage. This could be particularly interesting in transcostal therapy of the heart or the liver. Preliminary results indeed show that selective propagation of the therapeutic beam through the intercostal space would increase the peak pressure on target.

**References**

[1] Arnal et al, Appl Phys Lett, 101 1-5, 2012

[2] Robin et al, IEEE IUS, 2015

[3] Sarvazyan et al, Acoust Phys 55 630–7, 2009

[4] Luong et al, Sci Rep 6 36096, 2016

[5] Montaldo et al, Appl Phys Lett 2004 (No Image Selected)

### O36 The effects of steroids on the myocardial reduction induced by myocardial cavitation-enabled therapy (MCET)

#### Y. I. Zhu^1^, X. Lu^2^, C. Dou^2^, D. L. Miller^2^, O. D. Kripfgans^2, 1^

##### ^1^O.D. Kripfgans, Biomedical Engineering, University of Michigan, Ann Arbor, Michigan, USA; ^2^Department of Radiology, University of Michigan, Ann Arbor, Michigan, USA

###### **Correspondence:** Y. I. Zhu

**OBJECTIVES** Myocardial Cavitation Enabled Therapy (MCET) has been proposed as a means to achieve minimally invasive myocardial reduction using ultrasound to produce scattered microlesions by cavitating contrast agent microbubbles. Previous studies have demonstrated the efficacy of MCET to induce damage. The purpose of this work was to study the treatment effect of the steroid methylprednisolone (MP), in terms of swelling reduction following MCET and inhibition of fibrous tissue formation during long-term healing process.

**METHODS** Dahl/SS rats from Charles River were used as an *in vivo* model for HCM. Under ketamin/zylazine IP anesthesia, contrast agent was infused at a rate of 5μL/min/kg (tail vein catheter). The shaved and depilated left thorax was aimed at with a cardiac phased array (10S, Vivid 7, GE Healthcare) to center on the left ventricular myocardium. In this arrangement a 19 mm diameter single element therapy transducer was co-aligned to aim at a registered region of interest in the field of view of the 10S array. For therapy 10-cycle tone bursts at 1.5 MHz, 4 repetitions at 0.25 ms pulse interval, i.e. 4.0 kHz PRF, were sent every 8 heartbeats, aligned with trigger end systole (RR/3, using ECG gating). Peak negative free field pressures of 4 MPa were used to induce cavitation for 10 min. Therapy and sham therapy groups were followed up with MP administered at 0, 3, 6 and 24 hours after ultrasound exposure. Specifically, 30 mg/kg was chosen as high dose while 1 mg/kg was used as low dose alternative. Myocardial wall thickness 24 hours after therapy, measured from echocardiography was used to gauge the effect of initial myocardial swelling. White blood cell count was carried out 24 hours after therapy. Hearts were removed after 4 weeks and examined for evidence of the MP treatment effect. Histological sections with Masson’s trichrome staining were quantitatively analyzed for extent of fibrosis, i.e. tissue scarring.

**RESULTS** Myocardial wall thickness from echocardiography was measured as: sham 1.69±1.6 mm; therapy only 2.68±0.21 mm; therapy with low MP 2.29±0.22 mm; and therapy with high MP 2.01±0.14 mm. High dose of MP had a 25% wall swelling reduction with a p-value of 0.003 relative to the therapy only group. Absolute neutrophil count demonstrated the efficacy of MP: sham 5.0±1.1 109/L; therapy only 5.4±0.9 109/L; therapy with low MP 3.6±1.4 109/L; and therapy with high MP 2.7±1.2 109/L. High dose of MP had a 45% neutrophil count reduction though with a p-value of 0.026 relative to the therapy only group. Fibrosis densities were quantified for the treated regions as to represent the extent of scarring with results therapy only 25.6±4.0%; therapy with low MP 25.7±1.6%; and therapy with high MP 20.6±0.4%. High dose MP on average reduced fibrosis formation of 20% though is underpowered in this study with a p-value of 0.154.

**CONCLUSIONS** In this MCET rodent study, reduction trend of swelling wall thickness and neutrophil count has shown that MP is effective to inhibit the inflammatory response and reduce swelling right after therapy. Additionally, long-term study of fibrosis density analysis indicated that MP potentially helps reducing fibrosis formation. Cavitation enhanced therapy maybe a possible avenue for non-invasive tissue reduction for treatment of hypertrophic cardiomyopathy.

### O37 Application of MR-ARFI to monitoring the stiffness changes of porcine muscle after HIFU therapy

#### Yangzi Qiao, Chao Zou, Xin Liu, Hairong Zheng

##### Shenzhen Institutes of Advanced Technology, Chinese Academy, Shenzhen, China

###### **Correspondence:** Yangzi Qiao

**OBJECTIVES** HIFU with the capability to treat deep tumor precisely is a fast developing therapeutic method. Due to the multiple contrast and unique thermometry ability, MRI has been the most popular imaging modality for HIFU guidance. Stiffness is an intrinsic property of tissue, and can be monitored by MR-ARFI during HIFUtherapy. However, the MR-ARFI based tissue stiffness evaluation results were quite different in reports. It is expected that the tissue becomes stiffer after coagulation, producing decreased displacement. But in some other reports, the coagulation or stiffer implant leads to an increased displacement. The inconsistency of the reports may due to the different acoustic parameters of different tissue. In this study, MR-ARFI was applied to monitoring the stiffness changes of porcine muscle during HIFU therapy. The results demonstrate that ARFI induced displacement change is strongly correlated to coagulation. The displacement difference map can be used to depict the coagulation region, especially for small coagulation, making MR-ARFI an important complementary method for tissue monitoring during HIFU therapy.

**METHODS** All experiments were conducted on a 3T MR system (TIM Trio, Siemens). Ex vivo porcine muscle were sonicated by a 1.0MHz HIFU transducer (Imasonic). The input electric power was set from 8W to 100W, and transferred to the samples continuously in 10s to 60s. The segment SE-EPI (sEPI) sequence was usedfor ARFI imaging. The amplitude of displacement-encoding gradients (DEG) was 32mT/m, with input HIFU power of 55W and duty cycle of 2.5%. The duration of each DEG was 10ms.

Imaging protocol was: TR=600ms, TE=36ms, slice thickness=5mm, resolution=1.6*1.6mm2, matrix=54*128, EPI factor=9. Acquisition time=8.4s. Two sets of images with opposite polarity of DEG were used to quantify the displacement in each measurement. Ten measurements of ARFI were scanned before and immediately after HIFU sonication. T-test was used to determine whether tissue displacements have significantly changed. Temperature rise was monitored by GREduring HIFU sonication. The protocol was: TR=29ms, TE=10ms with the same FOV and resolution. The ambient temperature was 19°C. T2w image was acquired after HIFU sonication with TR=5000ms, TE=89ms, resolution=0.8*0.8 mm2, matrix=108*256.

**RESULTS** Figure 1(a) shows the displacement maps overlaid on anatomical image. No coagulation was found with 60 s sonication under 32 W power. The peak temperature change was 17.3°C in the end of the sonication, resulting in a peak temperature around 36°C. The averaged maximal displacement before sonication was 3.41±0.46 μm, while the averaged maximal displacement after sonication was 3.36±0.26 μm. The maximal displacement was constant during the successive 10 measurements after sonication, indicating the maximal displacement might be independent of temperature change (Figure 1(b)). It is also shown in Figure 1(c) there is no signification difference between the maximal displacements before and after HIFU sonication with *p* = 0.18 from *t*-test. Figure 2 shows another case with input power 82 W under 30s sonication, coagulation occurred. The peak temperature was around 85°C (peak temperature change was 66.7°C), much higher than the protein denature temperature. The displacement maps before and after sonication differed a lot. The maximal displacement significantly increased after sonication, with an averaged increment of 1.86 μm (p<0.01). The averaged maximal displacement before sonication was 3.46±0.17 μm, while the averaged maximal displacement after sonication was 5.32±0.28 μm. T2w image and displacement difference map (the difference between the displacement before and after HIFU sonication) were compared for visualization of thecoagulation. In T2w image, the coagulation was pointed out by a red dashed circle (Fig. 3). In displacement difference map, the region where displacement before and after HIFUsonication show significant difference (p<0.01) was overlaid on the anatomic image. In another sonication (with input power 82W, 10s sonication), The peak temperature was around 48.2°C (peak temperature change was 29.2°C). However, the coagulation can be hardly recognized in T2w image, while it is still visible in displacement difference map. The averaged maximal displacement before sonication was 3.45±0.12 μm. While the averaged maximal displacement after sonication was 5.06±0.19 μm. The coagulation area is marked in Fig. 4(c).

**CONCLUSIONS** In conclusion, MR-ARFI can detect significant changes of porcine muscle displacement while coagulation occurred. After heating the displacement significantly increased at the coagulation region. The displacement difference map can be used to visualize and evaluate therapy effect, especially for small coagulation. This makes MR-ARFI an important complementary method for tissue monitoring during HIFU therapy.

**Fig. 1 (abstract O37).**
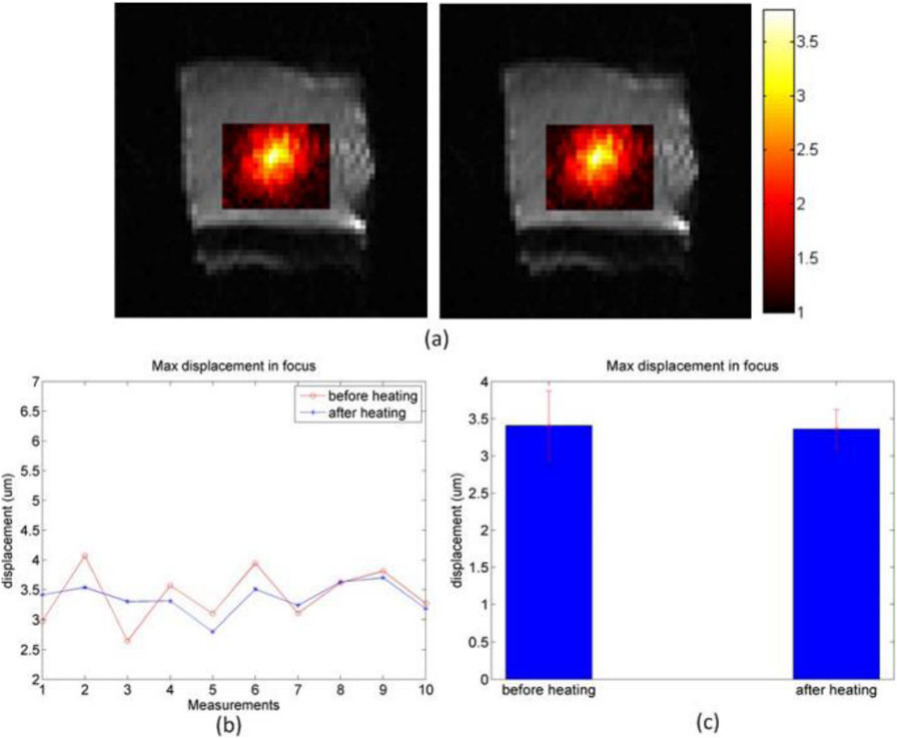
(a) The displacement map before (left) and after (right) HIFU sonication. (b) The maximal displacement in focus. (c) The averaged maximal displacement with inputpower of 32W, 60s continuous

**Fig. 2 (abstract O37).**
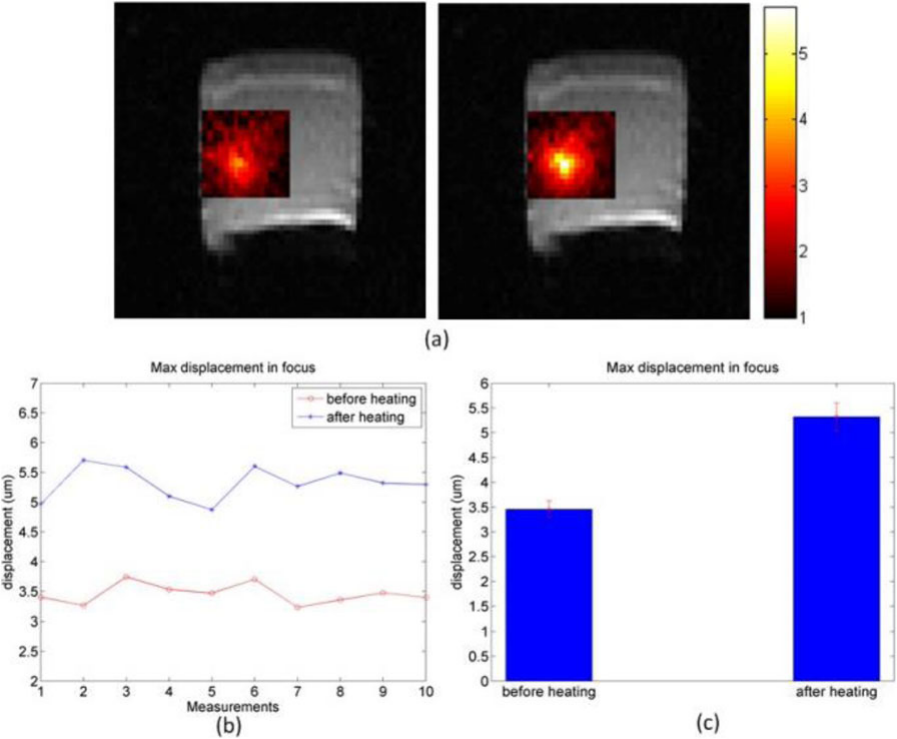
(a) The displacement map before (left) and after (right) HIFU sonication. (b) maximal displacement in focus. (c) The averaged maximal displacement with input power of 82W, 30s continuous sonication

**Fig. 3 (abstract O37).**
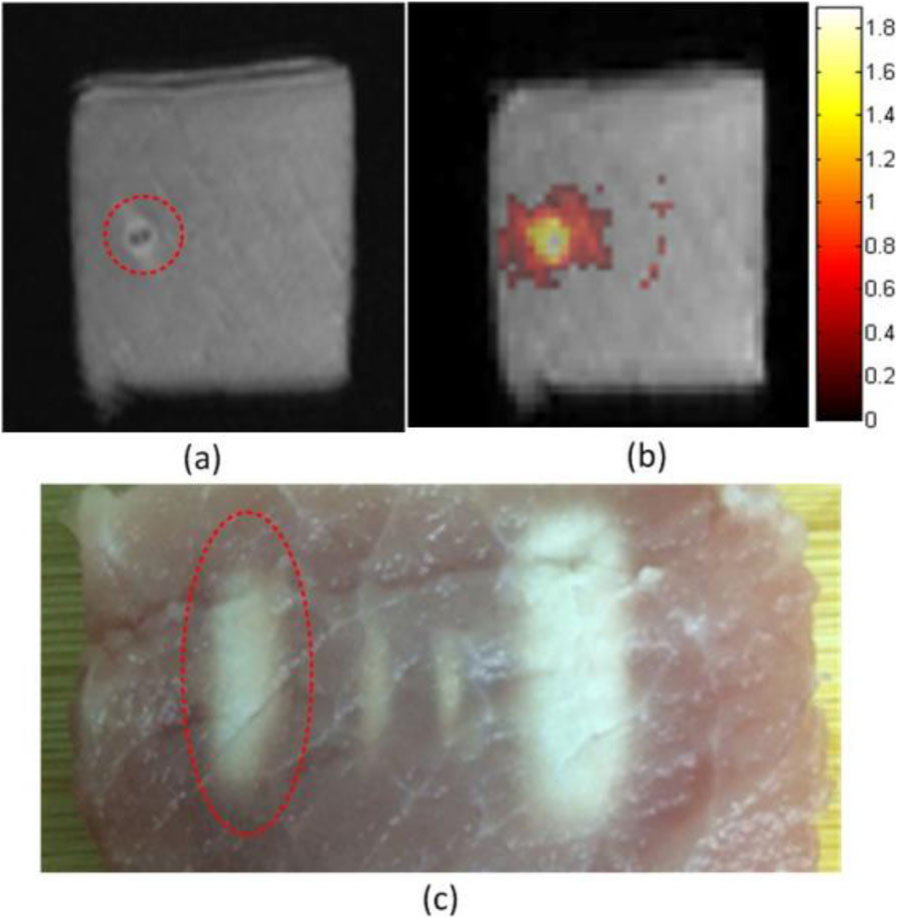
(a) T2w image, (b) displacement difference map, and (c) photo of coagulation with input power of 82W, 30s continuous sonication

**Fig. 4 (abstract O37).**
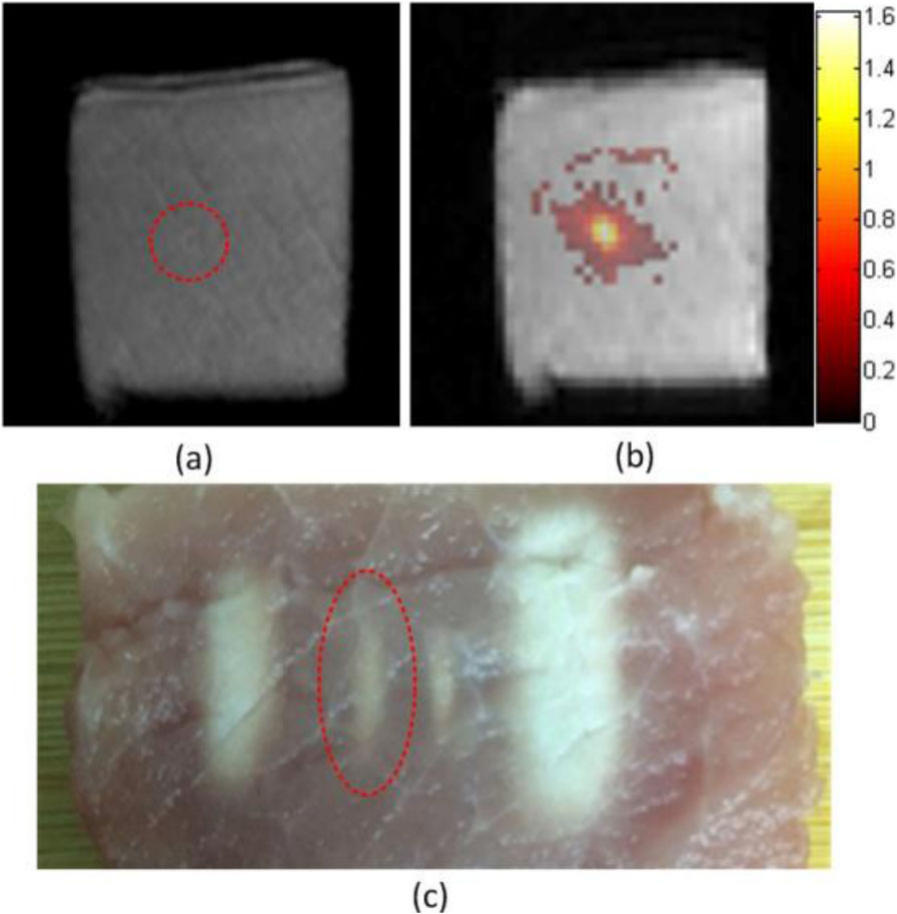
(a) T2w image, (b) displacement difference map, and (c) photo of coagulation with input power of 82W, 10s continuous sonication

### O39 Long-term oncologic outcomes of focal magnetic resonance guided focused ultrasound treatment for locally confined prostate cancer

#### Alexander Nosov, Sergey Reva, Maria Berkut

##### Onco-urological Department, N.N. Petrov Research Institute of Oncology, Saint-Petersburg, Russian Federation

###### **Correspondence:** Alexander Nosov

**OBJECTIVES** Progress in different diagnostic and treatment modalities of prostate cancer (PCa) have strengthened support for the use of focal high-intensity focused ultrasound (HIFU). However, important questions remain regarding candidate selection, treatment, and outcomes. We assessed long-term oncologic outcomes of focalHIFU in a small single-center cohort of low-risk PCa patients.

**METHODS** Twenty-two patients with low risk PCa (PSA<10 ng/ml, Gleason score less than 7, or clinical stage cT2b and less) were underwent for focal HIFU ablation (ExAblate 2100 for a prostate device, InSightec) with GE MRI suite and endorectal FUS transducer from March 2009 to January 2010. Among them, 8 patients were available for long term (a median time – 7.3 years) follow-up. Desired treatment target (Region of Treatment, ROT) and vulnerable structures (nerve vascular bundles) were marked on acquired magnetic resonance guided (MRg) planning images. Pre- and post-reatment strategy, rate and follow-up schedule was designed at PCa001 andPCa002 studies and described previously; prospective parts of these studies was closed after 6-month follow up.

**RESULTS** The average patient’s age 64 (49-73) years. Median pre-HIFU PSA level and post-HIFU PSA nadir was 7.6 and 3.9 ng/ml, respectively. Biochemicalrecurrence (BCR, defined as nadir + 2 ng/ml) was observed in 7 (87.5%) cases. Medium time to progression was 18 (3-32) months. In 4 (50%) cases local progression was confirmed by prostate biopsy and after metastatic process exclusion salvage radical prostatectomy (RPE). Generally, surgery after ablation was severe than in naïve patients; however, operative characteristics (operative time, blood loss, hospital stay) were comparable with historical cohort. During follow-up time, systemic treatment (hormonal therapy) was prescribed for 5 patients, as a result of distant metastatic progression after prostatectomy (2) and without secondary local treatment (3). Cancerspecific survival (CSS) and overall survival (OS) was both 100%.

**CONCLUSIONS** Despite acceptable results of survival, we found that almost all patients are progressed during follow-up. These data are in contrast with previously published data. However, patients in our series were younger than in historical cohort. We concluded that the use of focal high-intensity focused ultrasound in selected patients represents a strategy combining benefit of active surveillance and radical treatment in patients with low risk PCa. However, this concept should be evaluated in large prospective controlled studies.

### O40 Rapid diffusion-weighted imaging immediately after sonication using outer volume suppression pulses and single-shot, spiral acquisition

#### Steven P. Allen^1^, Xue Feng^1^, Helen L. Sporkin^1^, Jeff Elias^2^, Kim Butts-Pauly^3^, Craig H. Meyer^1^

##### ^1^Biomedical Engineering, University of Virginia, Charlottesville, Virginia, USA; ^2^Neurological Surgery, University of Virginia Hospital, Charlottesville, Virginia, USA; ^3^Radiology, Stanford University, Palo Alto, Virginia, USA

###### **Correspondence:** Steven P. Allen

**OBJECTIVES** There remains a critical need to predict the long term effects of a sonication during MR-guided, focused ultrasound thalamotomy. Current prediction methods include calculations based on the observed temperature trajectory, observations of the patient’s tremor score, and T2-weighted magnetic resonance imaging (T2-MRI). Each of these methods provides an imperfect estimate of the ultimate treatment effect. Diffusion-weighted MR imaging (DW-MRI) can potentially provide accurate lesion visualization and prediction at earlier time points than T2-MRI. This is analogous toischemic stroke, where DW-MRI is able to observe infarcts at early time points. To date, DW-MRI has achieved very limited success because of technical difficulties introduced by the focused ultrasound system such as eddy currents, magnetic field inhomogeneity, and patient motion. A dual-echo, steady-state sequence has found moderate success at identifying lesions in a pig model [1]. Here, we propose a single-shot, spiral DW-MRI sequence coupled with an outer volume suppression preparation sequence [2] as a means to quickly obtain diffusion weighted images of sub thalamic nuclei. See Fig. 1. The single-shot, spiral acquisition reduces sequence sensitivity to bulk motion and eddy currents. Meanwhile, the outer volume suppression pulse reduces the imaging field of view to a region that can be successfully supported by the spiral trajectory.

**METHODS** The proposed sequence is displayed in Fig. 1. A single-shot, spiral acquisition is used during the readout portion of a Stejskal-Tanner diffusion-weightedsequence. The spiral gradient samples a 4 cm diameter circular plane at 1.5 mm resolution using a variable density trajectory of 10 ms duration. The sequence is prepended by an outer volume suppression pulse consisting of an 8 ms long BIR-4 adiabatic excitation pulse and a 4 ms long cylindrical tip-back pulse as described in [2]. The preparation pulse reduces the field of available magnetizaiton to a cylinder approximately 3.5 cm in diameter. This sequence was validated using the the bodycoil of a 3T MR scanner (GE, Waukesha, WI) and a human volunteer. Imaging parameters are; TR: 1 s, TE: 45 ms, FOV: 5 cm, resolution: 1.5 mm, slice thickness: 0.8cm, bValue: 750 smm-2, averages: 16. We will measure the time course of the diffusion-weighted signal after sonication by subjecting two instances of a porcine model with craniotomy to focused ultrasoundablation (ExAblate Neuro, INSIGHTEC, Haifa, Israel) while under anesthesia. A total of 8 lesions will be made in each animal and DW and T2-weighted images will be acquired at time points spaced by 2 minutes from immediately after sonication to 20 minutes after sonication using the proposed sequence for DW-MRI and a fast-spin echo sequence with spiral acquisitons for T2-MRI. We will then compare the time course of the DW-MRI signal immediately after ablation to the time course of the T2-MRI signal.

**RESULTS** Figure 2A and B demonstrate the outer volume suppression sequence using an enlarged field of view and a six-shot, interleaved spiral acquisition. By applying the suppression preparation pulse, the field of view is successfully reduced to a region about the right ventricle. A single-shot spiral image with reduced field of view of this region, as well as an identical image with diffusion weighting, are displayed in Fig. 2C and D, respectively. Diffusion-weighted contrast can be observed in the suppressed signal of the ventricle. The poor transmit and recieve gains of the body coil do not prevent the successful implementation of this sequence.

**CONCLUSIONS** Single-shot, spiral acquisition with an outer volume suppression preparation pulse shows promise as a motion-insensitive sequence to capture diffusion weighted contrast in the thalamus.

**References**

[1] Plata et al. “A feasibility study on monitoring the evolution of apparent diffusion coefficient decrease during thermal ablation,” Med. Phys., 42(8), 5130–5137, 2015

[2] Smith et al. “Reduced field of view MRI with rapid, B1-robust outer volume suppression,” Magn. Reson. Med., 67(5), 1316–1323, 2012.

**Fig. 1 (abstract O40).**
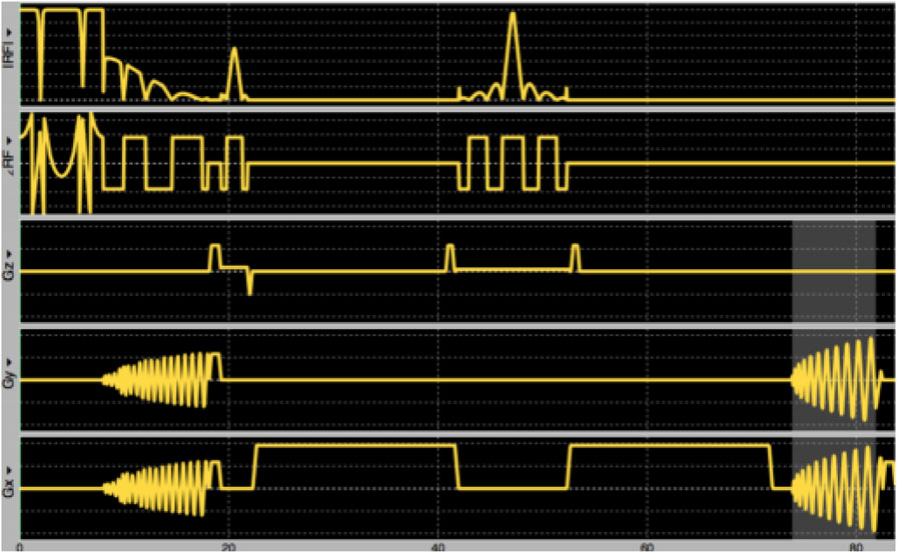
The proposed diffusion­weighted sequence. An outer volume suppression pulse is prepended to a Stejskal­Tanner diffusion­weighted imaging pulse using a single­shot spiral trajectory for image acquisiton

**Fig. 2 (abstract O40).**
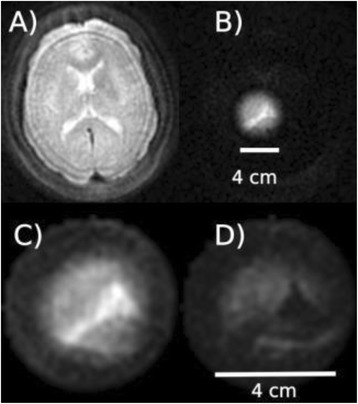
Validation of the proposed sequence in a human volunteer using the body coil of a 3T MR scanner. (A) Image acquired with a nominal field of view. (B) The same sequence as (A), but prepended with the outer volume suppression pulse. The preparation pulse reduces the region of excited magnetization to a small cylinder. (C) The reduced field of view imaged with a single­shot spiral acquisiton. (D) Same as (C) but with a diffusion­weighting of 750 smm­2. Diffusion­weighting can be observed inthe suppression of the ventricle. The poor transmit and recieve gains of the body coil do not prevent the successful implementation of this sequence

### O41 Selection of MR-HIFU hyperthermia treatment sites based on MR thermometry evaluation in healthy volunteers

#### Satya V.V.N. Kothapalli^1^, Michael Altman^2^, Ari Partanen^3^, Lifei Zhu^1^, Galen Cheng^1^, H. Michael Gach^2^, William Straube^2^, Dennis Hallahan^2^, Hong Chen^1,2^

##### ^1^Biomedical Engieering, Washington University in Saint Louis, Saint Louis, Missouri, USA; ^2^Department of Radiation Oncology, Washington University in St. Louis, Saint Louis, Missouri, USA; ^3^Clinical Science MR Therapy, Philips, Andover, Massachusetts, USA

###### **Correspondence:** Satya V.V.N. Kothapalli

**OBJECTIVES** Magnetic resonance imaging-guided high-intensity focused ultrasound (MR-HIFU) is a promising noninvasive technique for mild hyperthermia, enabling targeted and localized hyperthermia therapy deep within the body under real-time MRI-based temperature monitoring and control. However, the narrow therapeutic window (41-43°C) and long treatment duration (30-60 minutes) pose a great challenge on MR thermometry. As a first step toward effective clinical translation of MRHIFU hyperthermia, this study identified preferable treatment sites based on the accuracy and precision of MR thermometry performed on healthy volunteers.

**METHODS** A total of 15 healthy volunteers (age 18-45 y and body weight 45-90 kg) were recruited. A clinical MR-HIFU system (Sonalleve V2, Philips, Vantaa, Finland) was used with the patient tabletop docked into the MRI bore (Ingenia 1.5T, Philips, Best, the Netherlands). Volunteers were positioned above the tabletop’s acoustic window and a standard HIFU-compatible 3-ch pelvis RF receive coil was secured over the target anatomy. For MR image acquisition, the pelvis coil was used together with the 2-ch RF receive coil integrated in the HIFU tabletop. A dynamic multi-slice fast-field-echo pulse sequence (sequence (TR=41 ms; TE=19 ms;voxel=2.5×2.5×7 mm2; FOV=400×400 mm2)) was used for real-time MRI (without HIFU sonication) together with the proton resonance frequency shift (PRFS) method to calculate temperature maps. Eight volunteers were subjected to a shorter scanning period (5 mins) targeting the upper body, pelvis, and lower extremity. Seven volunteers were subjected to a longer scanning period (30 mins) targeting the lower extremities, i.e., thigh and calf. The precision of MR thermometry was quantified by the temporal temperature uncertainty, which calculated the temporal standard deviation for each pixel within an 18×18 mm2 ROI. The accuracy of MR thermometry wasquantified within the same ROI by the absolute error of measured temperatures compared to body temperature (37°C). Temperature monitoring with both uncertainty and absolute error lower than 1°C is expected to be satisfactory for hyperthermia.

**RESULTS** MR thermometry measurements based on 5-min scans at the chest, pelvis, and lower extremity had an uncertainty of 2.53°C±0.48°C, 1.89°C±0.50°C, and0.50°C±0.04°C, respectively, and an absolute error of 0.63°C±0.63°C, 2.88°C±0.87°C, and 0.08°C±0.13°C, respectively. MR thermometry measurements based on 30-min scans at the lower extremity found the uncertainty and the absolute error of the MR thermometry to be 0.52°C±0.13°C and 0.12°C±0.06°C, respectively. No significant difference was found between 5-min scanning and 30-min scanning for the lower extremity. Among the three anatomical sites, only the lower extremity had satisfactory temperature accuracy and precision according to the suggested criterion.

**CONCLUSIONS** This study constitutes the first evaluation of MR thermometry performance at different body sites for long scan times that are relevant in clinical MRgHIFU hyperthermia therapy. Motion induced by respiration and cardiac activity poses a technical challenge on the application of this treatment on chest and pelvis. Theextremity was found to be a preferable site for MR-HIFU hyperthermia using the suggested criteria that both the uncertainty and absolute error need to be under 1°C.

### O42 Behaviour of bubbles generated by acoustic droplet vaporization within inter-tissue region

#### Y. Ho, C. Yeh

##### Department of Biomedical Engineering and Environmental Sciences, National Tsing Hua University

###### **Correspondence:** Y. Ho

**OBJECTIVES** Treatment resistance is a critical consideration in cancer research. The tumor cells which extensively proliferate away from vessels show treatment resistance due to the restricted drug penetration and biological mutation of hypoxia. Acoustic droplet vaporization (ADV) can convert nanodroplets into gaseous bubbles (ADV-Bs) and simultaneously disrupt vessels to improves the penetration of nanodroplets, ADV-Bs, and drugs. Previous studies have been proved the mechanical force of bubble cavitation can directly induce *in vitro* and intravascular bioeffects. Therefore, our study investigated the behaviors of ADV-Bs in vessels and tissue triggered by ultrasound (US), and then evaluated the feasibility of using intertissue ADV-Bs to treat resistant tumor cells by physical damage.

**METHODS** The window chamber model was used to directly observe vessels and tissue pattern on mouse dorsal skin by the integrated acousto-optical system. Mice were injected 30 μL NDs (304±97 nm) with the concentration of 1.2-1.4×10^13^ NDs/mL to form ADV-Bs by 3-cycle single-pulsed US at 10 MPa. The modulated parameters of US with pulse repeat frequency of 1 Hz were used to stimulate ADV-Bs as following: (1) 100-cycle with 5 and 10 MPa for movement; (2) 3-cycle with 5 MPa for cavitation. Furthermore, the transgenic adenocarcinoma of the mouse prostate (TRAMP) cells with live-cell impermeable dye propidium iodide (PI) were used to evaluate cellular bioeffect induced by ADV-Bs.

**RESULTS** Intravital images showed vascular disruption, intravascular ADV-Bs, and intertissue ADV-Bs after ADV. The intravascular ADV-Bs were moved following the vascular shape and stuck at the branch (black arrows) during US stimulation (Fig. 1. A). On the other hand, the movement of intertissue ADV-Bs was not restricted and the distance per pulse was 48±17 μm for 5 MPa and 88±26 μm for 10 MPa. Cavitation of intertissue ADV-Bs was observed to split into two bubbles and coalesce back to one during US stimulation (Fig. 1. B). In Fig. 1. C, the intracellular enhancement of PI indicated cell membrane damage after ADV-Bs shoving (yellow arrows). The cell and ADV-Bs were blown out of the original position at 15 s because the cell stuck on the ADV-Bs and was pushed by pressure gradient of US.

**CONCLUSIONS** This study revealed intravital imaging to observe ADV-B formation, cavitation, and movement in vessels and tissue. Intertissue ADV-Bs could be pushed to distant tissue by US stimulation, and produced cell membrane damage when ADV-Bs cavitated or shoved cells during movement. Since the treatment resistant tumor cells live away from vessels, these results provided a possibility to touch these cells and damaged them by ADV-B movement and cavitation. Moreover, the cellular bioeffect induced by physical damage also presented a potential way to overcome the resistance of drugs and radiation in tumor therapy.

**Fig. 1 (abstract O42).**
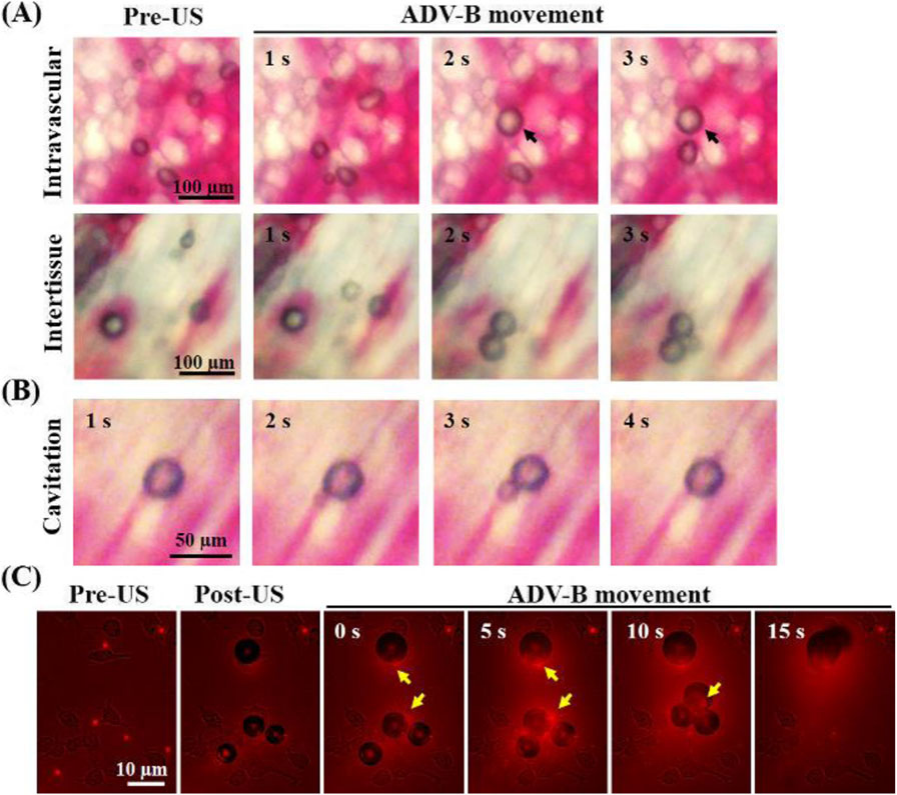
See text for description

### O43 Enhanced ultrasound-mediated trans-membrane drug delivery into a monolayer of endothelial cells during high flow *in vitro* conditions

#### Syed Mohd Nooh Syed Omar, Rob Krams, James J. Choi

##### Bioengineering, Imperial College London, London, UK

###### **Correspondence:** Syed Mohd Nooh Syed Omar

**OBJECTIVES** One of the challenges in the treatment of atherosclerosis and other cardiovascular disease is the inability to deliver therapeutic drugs across the cell membrane effectively and safely. Entire classes of drugs, nucleic acids (e.g., DNA, siRNA and mRNA) and biomolecules for improving treatment, are ineffective, because they cannot enter the cell at safe systemic doses that do not cause side effects throughout the rest of the body. Over the last several decades, ultrasound-mediated trans-membrane drug delivery methods (e.g. sonoporation) have been studied *in vitro* to deliver drugs into cells. Through these studies, it was discovered that several mechanisms of trans-membrane drug delivery exist and that they are highly dependent on the acoustic parameters, microbubble conditions, and the cell-type used. Despite promising results from these study, the advancement from single cell-bubble interactions to clinical use has not been made. This gap in development is largely because the underlying mechanism of trans-membrane drug delivery under high flow condition and for a large population of cells, remains poorly understood and poorly controlled. Our study explores the ultrasound and microbubble-mediated trans-membrane drug delivery efficiency and safety to a monolayer of endothelial cells using a state-of-theart physiologically-relevant cultivation system under different ultrasound exposure conditions. In the end, we will evaluate the critical question on whether safe transmembrane drug delivery can be achieved in such a complex, physiologically relevant environment.

**METHODS** Human umbilical vein endothelial cells (HUVEC) were selected as our model cells since they are well described due to their extensive use in vascular biology and are similar to the arterial endothelial cells we’d like to treat in future studies (e.g., in Atherosclerosis). They were cultured as a monolayer in a flow chamberunder static or pulsatile shear stress (10 dyne/cm2) and at 37°C for 3 days (Fig. 1a, b). A focused ultrasound transducer (0.5 MHz) sonicated the flow chamber in the presence of lipid-shelled microbubbles and during flow conditions (fluid flow rate: 17 ml/min) (Fig. 1c). A centrally-inserted transducer (7.5 MHz) was used to determine the type of microbubble activity generated by passively detecting cavitation emissions during sonication. After sonication, trans-membrane delivery was assessed by quantifying the intracellular uptake of propidium iodide (PI), which is a DNA-binding fluorescent dye that is normally impermeable to the cell membrane. Cell viabilitywas assessed by Calcein-AM. A range of ultrasound parameters (pressure, pulse length and exposure duration) and microbubbles conditions were systematically evaluated to identify different trans-membrane delivery and safety profiles.

**RESULTS** The main finding of our study is that safe trans-membrane drug delivery can be produced in a clinically targetable cell type (i.e., endothelial cells) underphysiologically relevant conditions (i.e., high fluid velocities) (Fig. 1d). This safe delivery is indicated by cells where red propidium iodide is co-localised with green calcein. A range of ultrasound parameters was explored and we were able to characterise different biological effects: cell viability with and without transmembrane delivery, cell death and cell detachment. Trans-membrane delivery increased and cell viability decreased with pressure ranging from 50 kPa to 150 kPa, at pulse length of 500 and 1000 cycles and in the presence of microbubbles (Fig. 2). It was seen that at the highest pressure and pulse length evaluated, transmembrane delivery occurred in9.68% ± 10.96% of cells while only 13.18 ± 7.33% maintained cell viability. At the lowest acoustic pressure and pulse length evaluated, trans-membrane delivery occurred in 1.12% ± 1.35% of cells while cell viability was 92.45% ± 9.04%. There were rapid transitions in cell viability and drug delivery efficiency between 75 kPa and 100 kPa.

**CONCLUSIONS** This study demonstrated that safe trans-membrane drug delivery can be produced in a clinically targetable cell type (i.e., endothelial cells) and under physiologically relevant conditions (i.e., high shear stress condition). However, the trans-membrane delivery efficiency was low. This implies that there is a specific microbubble dynamic within the exposed tissue volume that can produce the desired bio-effect, but that it is being produced in only a small subpopulation of microbubbles. In future works we will identify this safe and effective dynamic and develop pulse sequencing technologies to control it so that we can maximise safe drug delivery.

**Fig. 1 (abstract O43).**
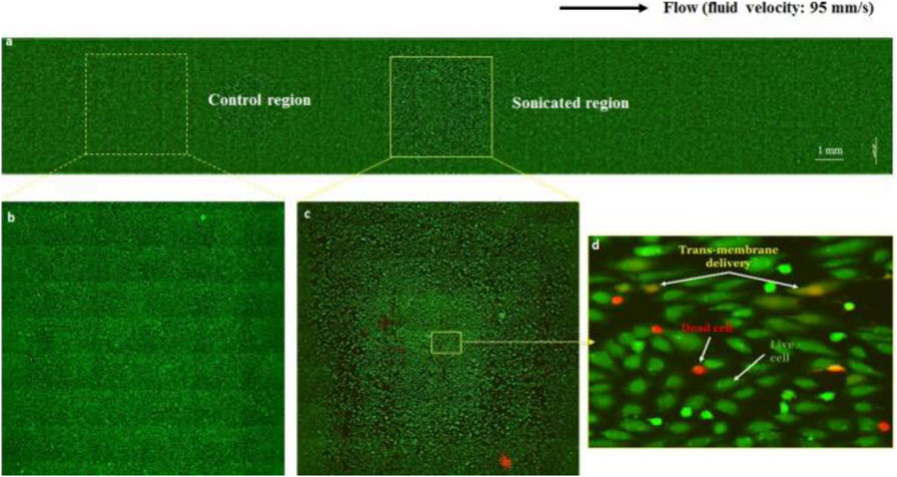
Trans­membrane drug delivery under flow condition. Human umbilical vein endothelial cell (HUVEC) were stained for cell viability by calcein­AM (green cells) and trans­membrane delivery by internalization of propidium iodide (red cells). (a) Microbubbles were made to flow (fluid velocity: 95 mm/s) through a collagen­treatedflow chamber with a channel height of 600 μm and a volume of 150 μL. (b) The regioin (dotted rectangle) acted as non­sonicated control region, while (c) central chamber region acted as sonicated region (solid rectangle). (d) Zoomed in view at delivery region; a population of cell that exhibited a mixture of viable cells (green cells) with and without trans­membrane delivery and dead cell (red cell)

**Fig. 2 (abstract O43).**
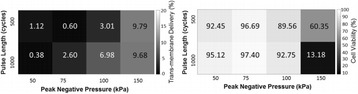
The effect of acoustic pressure and pulse duration on (a) Trans­membrane delivery and (b) cell viability. The percentages of trans­membrane delivery and cellviability are shown with respect to pressure at 50, 75, 100 and 150 kPa with pulse length of 500 and 1000 cycles. Exposure condition: fc=0.5 MHz, PRF=1.4 Hz, Np =500with microbubbles at 1X clinical dose. The results shown represents the mean for at least three independent measurements

### O44 Enhanced angiogenesis and perfusion *via* delivery of basic fibroblast growth factor using an acoustically responsive scaffold

#### Alexander Moncion^1,2^, Melissa Lin^2^, Eric O'Neill^2^, Oliver D. Kripfgans^2, 1^, Renny T. Franceschi^3, 4^, Andrew J. Putnam^4^, Mario L. Fabiilli^1, 2^

##### ^1^Applied Physics Program, University of Michigan, Ann Arbor, Michigan, USA; ^2^Department of Radiology, University of Michigan, Ann Arbor, Michigan, USA; ^3^School of Dentistry, University of Michigan, Ann Arbor, Michigan, USA; ^4^Department of Biomedical Engineering, University of Michigan, AnnArbor, Michigan, USA

###### **Correspondence:** Alexander Moncion

**OBJECTIVES** Regenerative growth factors (GFs) are expressed in a spatially- and temporally-regulated manner during wound healing. However, delivery of GFs using conventional hydrogel scaffolds does not afford such control, which has hindered the translation of GF-based therapies in tissue regeneration. We have developed acoustically-responsive scaffolds (ARSs), which are hydrogels doped with sonosensitive perfluorocarbon emulsions containing encapsulated GFs. When ARSs are exposed to ultrasound (US) above the threshold for acoustic droplet vaporization (ADV), the GF is released from the emulsion. This work studies how ARSs can be used to enhance angiogenesis in an *in vivo* model *via* the controlled delivery of basic fibroblast growth factor (bFGF), which can stimulate blood vessel formation.

**METHODS** bFGF was encapsulated in monodispersed, perfluorohexane (C6F14) double emulsions (mean diameter: 13.4±0.04 μm). ARSs (0.3 mL volume, 10 mg/mLfibrin, 1% (v/v) emulsion) were prepared with 1 μg of bFGF per implant, and injected subcutaneously in the lower back of BALB/c mice. After polymerization, a subset of the implants were exposed to US (2.5 MHz, Pressure = 8 MPa peak negative, 13 cycles, 100 Hz pulse repetition frequency) for 2 min daily. To assess perfusion in and around the implants, the mice were imaged on days 0,1 3, 7, 10, and 14 with a PeriMed Laser Speckle Contrast Imaging (LASCA) system. Blood vessel density in the implants was determined *via* CD31 immunohistochemical staining on days 7 and 14.

**RESULTS** LASCA (Fig. 1): A greater percent change in perfusion, relative to day 0, was observed in ARSs exposed to US (i.e., +US) versus ARSs not exposed to US (i.e., –US) on days 7 (97.0 ± 14.9% vs. 54.0 ± 13.1%) and 10 (46.9 ± 7.0 vs. 14.3 ± 5.0), respectively. CD31 (Fig. 2): More blood vessels were present in the +US ARSthan the –US ARS on day 7 (126.8 ± 23.8 vs. 73.1 ± 21.2 BVs/mm2). Sustained angiogenesis was observed with ARSs when compared to fibrin loaded with bFGF.

**CONCLUSIONS** US enables non-invasive, controlled release of bFGF from an ARS, which locally enhanced angiogenesis and perfusion. These finding could be applied in therapeutic angiogenesis for the treatment of cardiovascular diseases. Future work will attempt to demonstrate efficacy in ischemic pathologies as well as controlled release of multiple bioactive molecules.

**Fig. 1 (abstract O44).**
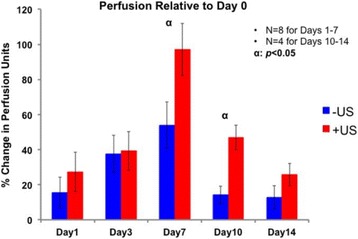
Percent change in perfusion relative to day 0. Peak perfusion was observed on day 7, while the +US condition had statistically greater perfusion both day 7 and 10

**Fig. 2 (abstract O44).**
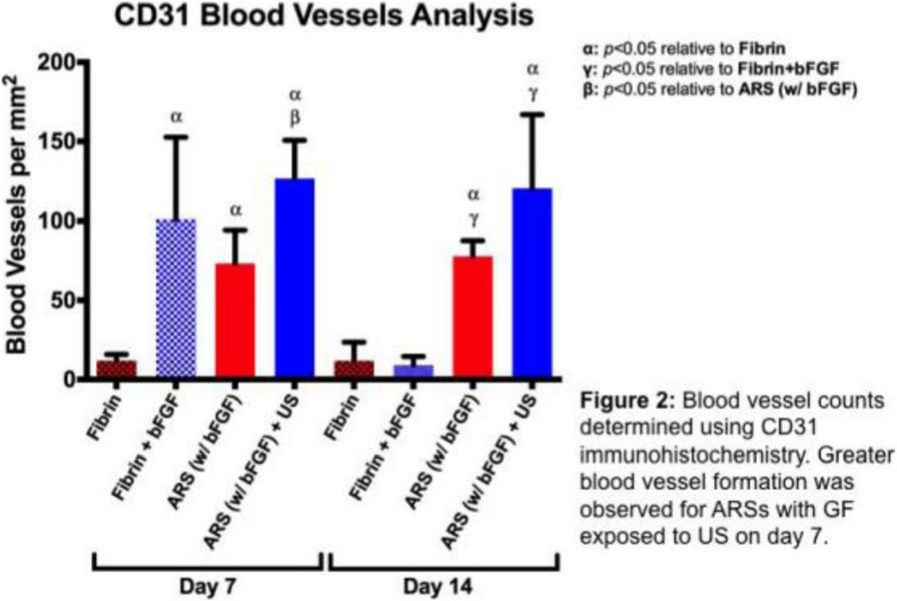
Blood vessel counts determined using CD31 immunohistochemistry. Greater blood vessel formation was observed for ARSs with GF exposed to US on day 7

### O45 Non-invasive peripheral nerve stimulation *via* focused ultrasound

#### M. Downs, S.A. Lee, Y. Baba, B. Hoffman, G. Yang, D. Kim, E. Lumpkin, E. Konofagou

##### Columbia University, New York, New York, United States

###### **Correspondence:** M. Downs

**OBJECTIVES** Focused ultrasound (FUS) has been demonstrated to modulate neuronal activity in both the central (CNS) and peripheral (PNS) nervous system. While modulation of the CNS has successfully elicited both motor and sensory physiological responses, PNS modulation has only been shown to induce inhibitory effects, primarily *ex vivo*. The ability to non-invasively stimulate and inhibit the PNS with FUS would allow clinicians an alternative therapeutic modality to treat peripheral neuropathy, as current treatment procedures can generate systemic side effects or require invasive procedures. In this study, we aim to show that FUS can elicit excitatory effects targeting the PNS and generate downstream physiological responses.

**METHODS** Focused ultrasound was used to target the sciatic nerve in mice while recording EMG responses from the tibialis anterior muscle in sedated mice (n = 25). A single-element HIFU transducer (3.57 MHz, 0.46x3.55 mm focal area) was used for stimulation while initially exploring the following parametric space: 0.7-5.4 MPa peak negative pressure, 4ms-1s stimulation duration, 5-100% duty cycle, 0.01- 1kHz PRF. Targeting of the nerve was conducted through a 18.5 MHz, 128-element imaging probe. Once efficacious parameters had been determined (the ability to elicit subsequent EMG responses after an initial response), the safety of the procedure was explored along with a comparison to electrical stimulation. H&E histology of the targeted region (n = 4 stimulation, n = 1 positive control, n = 1 negative control) were employed in a blinded study to detect damage (red blood cell extravasation, inflammation, cell membrane rupture). Mice (n = 4 stimulation, n = 4 control) completed an open field behavioral test to explore the short-term safety of the experiment. FUS EMG responses (peak-to-peak, delay to signal) were compared to direct electrical stimulation (1-10 V, 1-10 mA, 200500μs). To measure temperature increase during FUS stimulation, thermocouples were embedded in *ex vivo* mouse limbs alongside the sciatic nerve and exposed to FUS stimulation. A force balance was used to determine the acoustic radiation force generated by the transducer to estimate the tissue deformation in the focal region. To confirm neural activity at the single-unit level, a *ex vivo* skin-saphenous nerve prep (n = 15) was also stimulated by FUS with the aforementioned parameters while recording extracellular electrophysiology responses.

**RESULTS** Non-invasive stimulation of the sciatic nerve *via* FUS was shown capable of eliciting both observable leg twitching and measurable EMG responses when using the following FUS parameters: 0.2-5.7 MPa, 35-100% DC (continuous wave), 0.1-1kHz PRF, 0.8-10.5 ms stimulation duration. Increasing the pressure and DC raised the average peak-to-peak EMG response along with the success rate (Fig. 1). Varying the PRF or stimulation duration did not have a significant effect on response. Both delay and peak-to-peak EMG responses for FUS stimulation were found to be comparable to direct electrical stimulation of the sciatic nerve. Mice stimulated with efficacious parameters did not display any significant deviation in behavior compared to the control group or baseline values. The blinded histology study did not detect any damage for the stimulated group, only for the positive control. In *ex vivo* experiments, FUS was able to stimulate action potentials firing in multiple classes of peripheral neurons. Latency to action potential firing was dependent on sensory neuron type and FUS intensity.

**CONCLUSIONS** FUS stimulation of the sciatic nerve was shown capable of eliciting physiological responses *in vivo*. This demonstrates that FUS can be a non-invasive alternative to conventional therapeutic methods. Specific FUS parameters has been identified for successful and safe stimulation. Future work to explore the potential mechanisms of generation of the action potential will dictate the FUS parameters to translate this technique to clinical applications.

**Fig. 1 (abstract O45).**
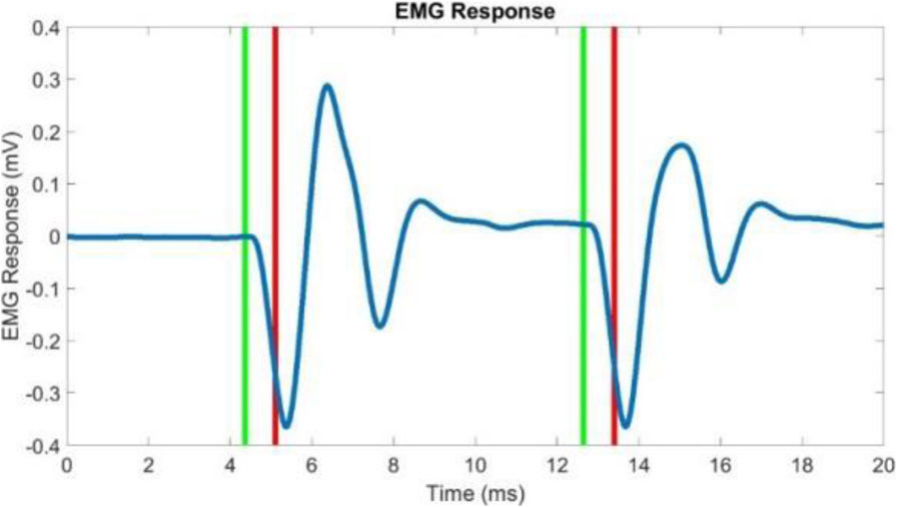
EMG responses to FUS stimulation of the sciatic nerve. The green and red vertical lines are the start and end of FUS stimulation respectively. The blue line is the EMG response to FUS stimulation. Stimulation parameters were as follows: 3.5 MHz, 3.7 MPa, continuous wave, 0.88ms stimulation duration

### O46 The safety and feasibility of high intensity focused ultrasound in treatment of resistant hypertension

#### P. You

##### State Key Laboratory of Ultrasound Engineering in Medicine Co-founded by Chongqing and the Ministry of Science and Technology,Chongqing Key Laboratory of Ultrasound in Medicine and Engineering,College of Biomedical Engineering,Chongqing Medical University, Chongqing, China

**OBJECTIVES** To evaluate the safety and feasibility of high intensity focused ultrasound (HIFU) in the treatment of resistant hypertension.

**METHODS** 40 patients with resistant hypertension underwent the treatment of high intensity focused ultrasound, Intraoperative and postoperative adverse effects were recorded; the drop of blood pressure, types of drug used, peak systolic velocity of renal artery and renal function was followed up to 6 months post treatment.

**RESULTS** (1) All patients completed HIFU treatment successfully.The major discomforts that patients complained during the procedure was pain in the treatment area,which usually disappeared within 24 hours; Base on the SIR classification,most of adverse effects were classified as A to B, no C to F was found.(2) Preoperative and postoperative peak systolic velocity of left and right renal artery were no statistic difference(P=0.635,P=0.688).The comparison between baseline and 1,6 months of blood urea nitrogen, serum creatinine and glomerular filtration rate were no statistic difference (P=0.772, P=0.652, P=0.366). (3) The systolic blood pressure dropped 21.5,23.3,22.4mmHg (P=0.000), diastolic blood pressure dropped 11.1,12.9,12mmHg (P=0.000).24-hour systolic blood pressure dropped 13.6, 15.2, 14.3mmHg (P=0.000),24-hour diastolic blood pressure dropped 5.5,6,4.4mmHg(P=0.000); and types of drug dropped 0.8,0.9,1(P=0.000) in the later 1, 3, 6 months.

**CONCLUSIONS** HIFU can be safely and feasible used in the treatment of resistant hypertension. However, further study about long-term safety and efficacy of HIFU is still needed.

### O47 Efficacy and influential factors of focused ultrasound therapy for NNEDV

#### C. Li^1,2^

##### ^1^The College of Biomedical Engineering, Chongqing Medical University, Chongqing, Chongqing, China; ^2^Haifu Hospital of the First Hospital Affiliated Hospital, Chongqing Medical University, Chongqing, China

**OBJECTIVES** To investigate the effectiveness and safety of focused ultrasound for treating non-neoplastic epithelial disorders of vulva (NNEDV) and to analyse the factors that affect the effectiveness of focused ultrasound.

**METHODS** 136 patients with pathologically confirmed NNEDV underwent focused ultrasound treatment. Patients were followed up on a regular basis after treatment. The efficacy was evaluated based on degrees of vulvar itching, physical signs and changes in pathological finding in local lesion. The relation between age, course, status of menopause, pathological type and curative was analyzed.

**RESULTS** The average follow-up period was 23.8 months (range 3 months to 60 months). The complete remission occurred in 68 of 136 patients. The cure rate was 50% (68/136). 59 were found much more improved. The effective rate was 93.38% (127/136). 9 were ineffective and the inefficiency was 6.6% (9/136). 7 cases recurred and the recurrence rate was 5.51%(7/127). No severe side effects were found during treatment and no complications were observed during follow-up. The age, course of disease and status of menopause were related to the efficacy (c2=21.017, P= 0.000; c2=26.591, P= 0.000; c2=8.199, P= 0.000). There was no significant difference in the efficacy of different pathological types (c2=1.635, P= 0.442).

**CONCLUSIONS** NNEDV can be treated with focused ultrasound effectively and safely. The course of the disease, menopause status and the age of the patients can be considered as the predictive factors.

### O48 Clinical experience of intra-operative high intensity focused ultrasound in patients with colorectal liver metastases: Results of a Phase II study

#### D. Melodelima^1,2^, A. Dupre^1, 2^, Y. Chen^2^, D. Perol^2^, J. Vincenot^1,^ M. Rivoire^1, 2^

##### ^1^LabTAU - U1032, INSERM, Lyon, Rhône Alpes, France; ^2^Centre Leon Berard, Lyon, France

###### **Correspondence:** D. Melodelima

**This abstract is not included as it has already been published:**

Melodelima D, Dupre A, Vincenot J, Chen Y, Perol D, Rivoire M. A49 Clinical experience of intra-operative High Intensity Focused Ultrasound in patients with colorectal liver metastases. Results of a Phase II study. J Ther Ultrasound. 2016; 4(Suppl 1):31. Available from: https://jtultrasound.biomedcentral.com/articles/10.1186/s40349-016-0076-5.

### O49 Catheter-directed thrombolysis of deep vein thrombosis enhanced by intraclot microbubbles and ultrasound: A clinical study

#### Q. Zhu^1^, S. GAO^1^, G. Dong^2^, M. Guo^1^, F. XIE^3^

##### ^1^Department of Ultasound, XinQiao Hospital,Third Military Medical University, Chongqing, China; ^2^Department of Ultrasound, The First Affiliated Hospital of Zhengzhou University, Zhengzhou, Henan, China; ^3^Internal Medicine Cardiology, University of Nebraska Medical Center, Omaha, NE, China

###### **Correspondence:** Q. Zhu

**OBJECTIVES** This study is aimed to investigate the safety and effectiveness of treating deep vein thrombosis (DVT) using catheter-directed therapy (CDT) combined with intraclot microbubble-enhanced ultrasound therapy (IMUT).

**METHODS** Fourteen patients with acute DVT (<14 days) undergoing CDT were consented to accept coordinated IMUT treatment as the experimental group. During CDT process, percutaneous therapeutic ultrasound (TUS) and trans-catheter injection of SonoVue® microbubbles were simultaneously performed for about 20-30 minutes twice a day depending on the length of thrombus. A TUS device (SL-10 Sonolyser, Welld Medical Electronics Co., Ltd., China) equipped with a single-element, non-focused transducer was used for ultrasound thrombolysis. The transducer was operated at the frequency of 1.0 MHz with the duty factor of 0.01 and the peak negative pressure was 750kPa or 1.0 MPa depending on the depth of the thrombus. One vial (5mL) of SonoVue® microbubbles into 15 mL saline was infused constantly into the catheter during the treatment. Figure 1 shows the working parameters of the therapeutic ultrasound device. The time frame could be seen in Fig. 2. The other sixty acute DVT patients treated with the same CDT procedure without combining IMUT were retrospectively reviewed for the treatment days and overall urokinase dosage as the control group. The criteria for terminating thrombolysis and extubation were vessel recanalization confirmed by contrast-enhanced ultrasound (CEUS). The major complications like hemorrhage were monitored. The average treatment days and overall urokinase doses of the two groups were compared by Independent Sample Test.

**RESULTS** Images of a patient in the experiment group were showed in Fig. 3. The average treatment days of the experiment group (4.2 ± 1.5 d) were significantly less than that of the control (11.8 ± 4.4 d) (p<0.01). Also, the overall dosage of urokinase used in the experiment group (3.45 ± 1.66 million IU) dropped significantly about 28.2% when compared to the control group (4.80 ± 2.47 million IU) (p<0.01). Figure 4 presents the results between the two groups. No intracranial and local hemorrhage events happened in both groups.

**CONCLUSIONS** By combining IMUT in CDT treatment of acute DVT, the treatment days and overall urokinase dosage were remarkably reduced. This method may help to short hospital stay and reduce the risk of hemorrhage.

**Fig. 1 (abstract O49).**
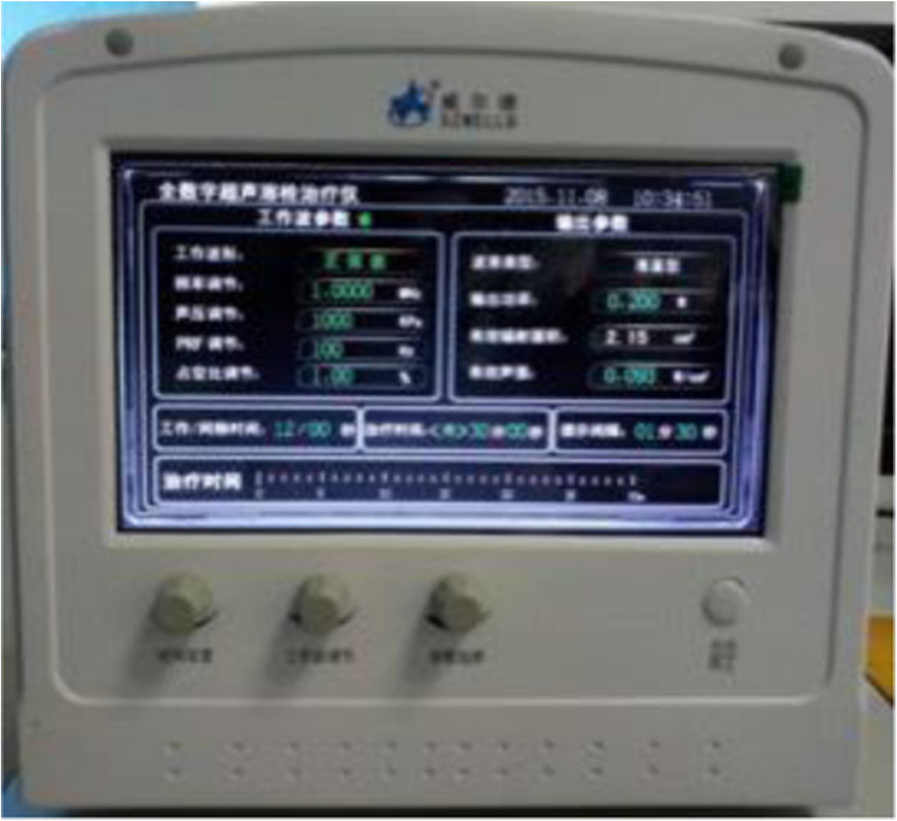
See text for description

**Fig. 2 (abstract O49).**
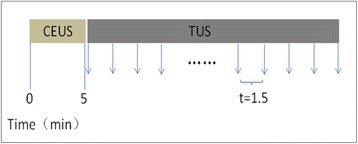
See text for description

**Fig. 3 (abstract O49).**
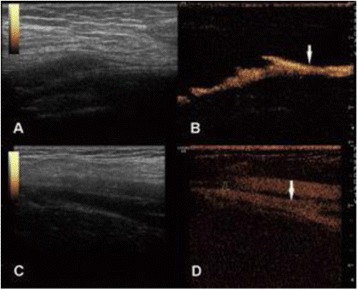
See text for description

**Fig. 4 (abstract O49).**
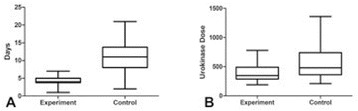
See text for description

### O50 Influence of "T2-RIM Sign" on immediate therapeutic responses to magnetic resonance-guided high-intensity focused ultrasound ablation of uterine fibroids

#### S. Yeo^1^, Y. Kim^2, 3^, H. Lim^3, 4^, H. Rhim^3^, S. Jung^4, 5^, N. Hwang^5^

##### ^1^Radiology, University Hospital of Cologne, Cologne, Germany; ^2^Radiology, Mint Hospital, Seoul, Korea; ^3^Radiology and Center for Imaging Science, Samsung Medical Center, Seoul, Korea; ^4^Health Sciences and Technology, SAIHST, Sungkyunkwan University, Seoul, Korea; ^5^Biostatistics and Clinical Epidemiology Center, Research Institute for Future Medicine, Samsung Medical Center, Seoul, Korea

###### **Correspondence:** S. Yeo

**OBJECTIVES** Magnetic resonance-guided high-intensity focused ultrasound (MR-HIFU) is a thermal ablation technique, which is gaining acceptance as an alternative treatment option for patients with symptomatic uterine fibroids. To date, previous studies have shown that different MRI characteristic of the uterine fibroids, such as high overall signal intensity on T2-weighted MR images and high perfusion, can be correlated with poor therapeutic outcome. Here, we present a study investigating the influence of a high-signal-intensity peripheral rim on T2-weighted MR images (i.e., T2-rim sign) on the immediate therapeutic response of MR-HIFU ablation of uterine fibroids.

**METHODS** This retrospective study was approved by the institutional review board, and patient informed consent was obtained for MR-HIFU ablation. In total, 196 fibroids (diameter 6.2±2.6 cm, range 3.1-13.6 cm) in 123 women (age 43.4±5.0 years, range 26-55 years) who underwent MR-HIFU ablation from January 2013 to April 2016 were included. The presence of a T2-rim sign and its corresponding percent coverage around the uterine fibroids were assessed on T2-weighted MR images acquired on the day of the treatment. The effects of a T2-rim sign on the immediate therapeutic responses (non-perfused volume [NPV] ratio, ablation efficiency [NPV/treatment cell volume], ablation quality [grade 1-5, poor to excellent]) were investigated with univariable and multivariable analyses using GEE (generalized estimating equation) analysis. In multivariable analysis, T2 signal intensity ratio of fibroids-to- skeletal muscle, relative peak enhancement of fibroids, and subcutaneous fat thickness were also considered.

**RESULTS** The presence of a T2-rim sign significantly lowered the NPV ratio (54.0±28.0% vs. 83.7±17.7%), ablation efficiency (0.6±0.5 vs. 1.3±0.6), ablation quality (3.1±1.2 vs. 4.2±0.8), (P<.0001). There were significant negative correlations between the percent coverage of a T2-rim sign and the NPV ratio (ρ=-0.4648, P<0.0001), ablation efficiency (ρ=-0.5086, P<0.0001), and ablation quality (ρ=-0.5086, P<0.0001). GEE analysis showed that the presence of a T2-rim sign and its corresponding percent coverage were independently significant for ablation efficiency and ablation quality (P<0.05).

**CONCLUSIONS** Uterine fibroids with a T2-rim sign showed significantly poorer immediate therapeutic responses to MR-HIFU ablation.

### O51 The current clinical applications of MR guided focused ultrasound surgery in China

#### H. Wang

##### Radiology, Shanghai General Hospital, Shanghai, China

**OBJECTIVES** To demonstrate the history and the current state of clinical applications of MR guided focused ultrasound surgery (MRgFUS) in China.

**METHODS** The history and the current state of clinical applications of MRgFUS from 2011, when it was first introduced into China, was reviewed and surveyed.

**RESULTS** MRgFUS had been approved for the treatment of uterine fibroids by the China Food and Drug Administration (CFDA) at 2013. The other registered clinical study of MRgFUS for CFDA is for the palliation of pain in bone metastasis, which also had been completed at 2015. Moreover, MRgFUS is in ongoing clinical trials for the treatment of adenomyosis of the uterus, uterine incision pregnancy, and osteoid osteoma in some institutions. So far, more than 500 MRgFUS treatments were completed in China. Some institutions are going to start clinical trials for the MRgFUS treatment of facet joint syndrome, painful knee arthritis, breast adenoma, prostate cancer and tremor.

**CONCLUSIONS** Although it was introduced into clinic in China just several years,MRgFUS,the new noninvasive thermal ablation method, already demonstrates its huge potential and prospect.

### O52 A predictive simulation framework for combined focused ultrasound hyperthermia and radiation treatment modelling at a cellular level

#### S. C. Brueningk^1^, G. G. Powathil^2^, P. Ziegenhein^1^, J. Ijaz^3^, I. Rivens^1^, M. Chaplain^4^, U. Oelfke^1^, G. ter Haar^1^

##### ^1^Joint Department of Physics, The Institute of Cancer Research, Sutton, UK; ^2^Swansea University, Swansea, UK; ^3^Bristol University, Bristol, UK; ^4^St. Andrew's University, St. Andrews, UK

###### **Correspondence:** S. C. Brueningk

**OBJECTIVES** Combined radiotherapy (RT) and hyperthermia (HT) treatments offer great potential for the successful treatment of radiation-resistant tumours by thermo-radio-sensitisation. Focused ultrasound (FUS) can be used to induce local HT. For treatment planning, it is essential to quantify the biological effects of such combination treatments. For this purpose, we present a multiscale systems oncology simulation framework. The objectives of this study are (1) to design and simulate *in vitro* FUS experiments, (2) to verify FUS simulation using measured temperature distributions, (3) to predict the cellular effects of combination treatments.

**METHODS** A 3D cellular automaton model (CAM), based on the computational concepts outlined in [1], was implemented in C++, to model cell populations and their treatment response. Each cell undergoes an individual cycle that regulates its treatment response and proliferation. A separate cell survival model was used to calculate surviving fractions for the combinations of radiation and thermal doses delivered. From this, a known proportion of cells would undergo immediate, or reproductive, cell death *via* mitotic catastrophe. The CAM was compared to results from experiments designed to characterise the response of HCT116 cells *in vitro*. Clonogenic assays, cellular growth curves, and flow cytometry analysis were used to assess overall survival, growth dynamics, and cycle distribution after homogeneous heating and/or irradiation. In particular, the influence of the cell kill model used in the simulation (instantaneous vs. reproductive cell death) was tested. A linear propagation model of FUS exposure [2] was implemented. To achieve a heated volume significantly larger than the geometric focus, a transducer was moved at constant speed in a circular trajectory. The majority of the heated cells are not exposed to FUS directly, but are heated by diffusion of thermal energy into the circle’s centre. Heat generation and diffusion were simulated by iterative solution of the bioheat equation with a continuously updated heat source position. In order to verify the accuracy of the FUS model, temperature distributions were both modelled and measured. The experimental arrangement (see Fig. 1) consisted of a cell containing collagen gel sandwiched between two disks of acoustically absorbing PVA gel, in a water tank. This gel sandwich was exposed to heating from the moving focus (0.5mm lateral extent, 0.5-1.5kW/cm^2^ peak intensity) from a single element focused transducer (Sonic Concepts, Bothell, USA, 1.66MHz). The circular trajectory (6-8mm Ø) was traced out for 300s with a period of 1s^-1^. The temperature in the cell layer at the centre of the exposed circle was measured using a fine wire thermocouple (RS Instruments, Corby, UK). Simulations of this arrangement were performed, together with a sensitivity analysis of the thermal conductivity, specific heat capacity, and attenuation coefficient of the materials used. Water parameters were assumed for speed of sound, and PVA gel density. Using these physical and biological model calibrations, the viability distribution in cell layers exposed to FUS mediated HT will be predicted, and compared qualitatively to an experimental cell viability (MTT) assay.

**RESULTS** For biological modelling, the implementation of reproductive rather than immediate cell death was essential for the growth response dynamics of treated cells to be correctly captured. Having calibrated the growth and death rates in the simulation with those of HCT116 cells, more growth curves could correctly be predicted computationally for homogeneous HT, and/or RT treatments (see Fig. 2). Using the experimental arrangement described above, thermal doses in the therapeutic range (50-250 CEM) were achieved in the cell layer within the heated circular area. The simulation of the time-temperature curves was in good agreement with experimental data once the physical parameters had been adjusted (see Fig. 3 left). However, experimental variations in the thermal and attenuation properties, and set-up uncertainties between different gel samples had a strong influence on the time-temperature profiles and thermal dose (see Fig. 3 right). The corresponding measured material properties, and qualitative comparisons of simulated and experimental cell viability data of treated cell-containing gels will be presented.

**CONCLUSIONS** This CAM presented can easily be adapted to different cell lines and treatment scenarios. It therefore has potential for use in future studies of more realistic, tumour-like cell populations (e.g. spheroids), and their response to FUS and RT. Such simulations may help to identify and optimise treatment schedules & exposure conditions.

**Reference**

[1] G. Powathil et al., Semin Cancer Biol, (30, p.13–20), 2015 [2] B. Clarke, Ultrasound Med Biol, (21, p. 353–363), 1995

**Fig. 1 (abstract O52).**
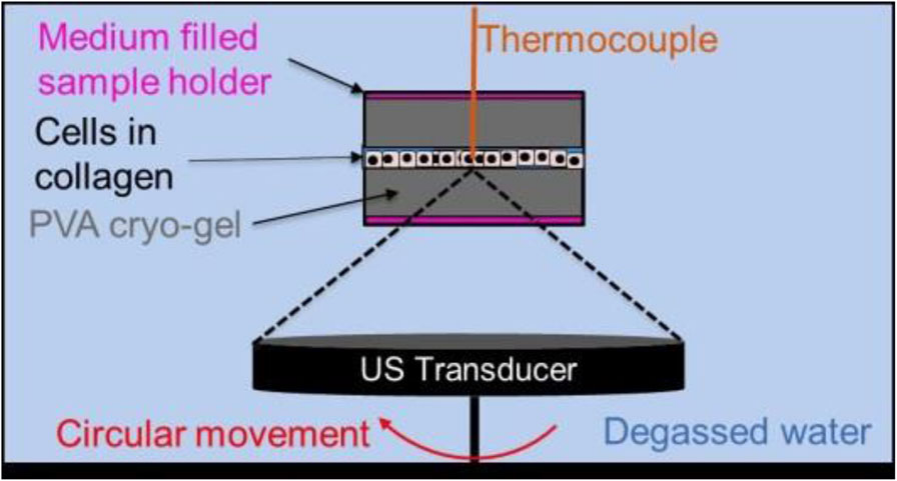
Experimental arrangement used. A gel sandwich consisting of a thin (~300μm thick), cell containing collagen gel and two 6mm thick slices of PVA cryo-gel is enclosed in a sterile sample holder filled with degassed cell culture medium. Top and bottom of the sample holder were sealed with tight Mylar membranes to minimize beam reflection. The sample holder is placed with the cell layer located at the focal plane of a focused single element transducer in a water tank. For treatments, the transducer is moved on a circular trajectory with temperature being monitored by a fine wire thermocouple at the center of the circle at the cell layer

**Fig. 2 (abstract O52).**
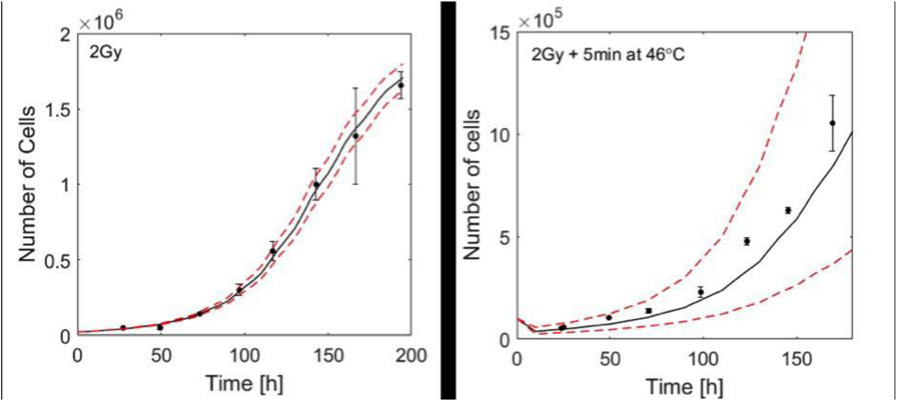
Comparison of experimental and simulated growth curves for HCT116 cells treated with different modalities. Left: 2Gy radiation (22000 cells seeded in 24-well plates, surviving fraction S2Gy = 0.43(0.38,0.49)). Right: Combination of 2Gy radiation and heating for 5min at 46°C (300000 cells seeded in 6-well plates, S2Gy+5min,46C = 0.11(0.06,0.19)). Cells were simulated with a mean doubling time of 19.5h. The experimental data points (black circles) are shown, as are the simulated total cell numbers (solid lines), and simulation of the 95% confidence bounds of the surviving fractions used (dashed lines)

**Fig. 3 (abstract O52).**
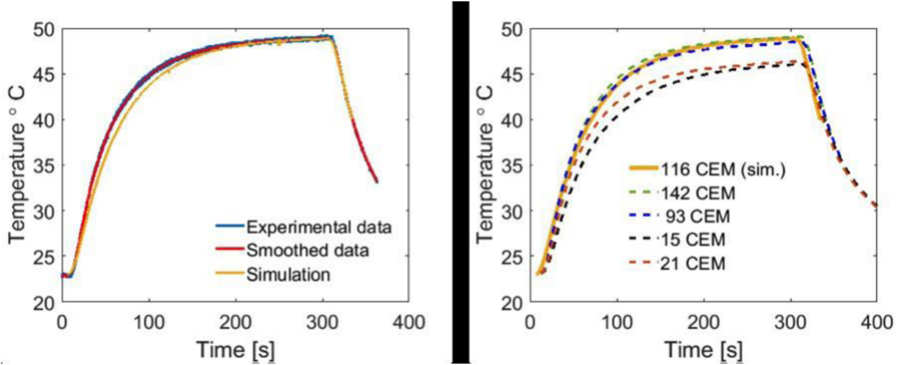
Comparison of experimental and simulated time-temperature curves for moving focus (1143 W/cm^2^ peak intensity, 6mm diameter of the circular trajectory). Left: The logged temperature data (blue) is first smoothed using a moving average (red), and then simulated (yellow) using a thermal conductivity of 0.49Wm^-1^K^-1^, a specific heat capacity of 2000Jkg^-1^K^-1^, and an acoustic absorption coefficient of 0.49nepers/cm at 1.66MHz. Right: Comparison of the simulation (solid yellow line) with repeat measurements in the same experimental set-up for different PVA/gel samples (smoothed data, dashed lines) illustrating experimental uncertainties in thermal dose (15-142CEM)

### O53 Lithotripter shock wave interaction with a bubble near various bio-material

#### S. Ohl^1^, E. Klaseboer^1^, A. Szeri^2^, B. Khoo^3^

##### ^1^Institute of High Performance Computing, Singapore, Singapore; ^2^University of California, Berkeley, Berkeley, California, USA; ^3^National University of Singapore, Singapore, Singapore

###### **Correspondence:** S. Ohl

**OBJECTIVES** The interaction of a lithotripter shockwaves with a gas bubble which is located near bio-materials such as fat, skin, muscle, cornea, cartilage, and bone is studied using numerical simulation. Our model is verified with comparison to experimental observations from Sankin & Zhong (2006) where a bubble collapses near an elastic membrane after it interacts with a lithotripter shock wave. We proceed to perform a systematic study of the various factors influencing the bubble collapse, including wave profile, initial bubble size, and reflection of the shock waves at rigid interface (such as bone). The results may be useful in the design of lithotripster shock wave therapies and the prevention of collateral damages in medical treatments.

**METHODS** The shock waves are modeled as a traveling pressure wave across the domain. The bio-materials have linear elasticity and are different in their density and Young's modulus. The Boundary Element Method is employed to solve the potential flow surrounding the bubble. The bubble is assumed to be adiabatic and initially stable.

**RESULTS** It is seen in Fig. 1 (a) that an oscillating bubble collapses with a jet towards the elastic membrane (Sankin & Zhong, 2006). Our simulation result (Fig. 1(b)) compares well to the experimental observation in bubble shapes and the timing of events. Figure 2 shows the collapse of the bubble near the elastic membrane after being hit by a shock wave at 114 μs. It is observed in both experiment and simulation that the bubble splits as the membrane moves towards the bubble. We use our model to study the effect of initial bubble size in the bubble shock waves interaction near fat tissue. It is seen in Fig. 3(a) that the small bubble of 100 μm in radius collapses with a high speed jet towards the fat tissue (and the fat tissue moves towards the collapsing bubble). However, if a larger bubble is present (in this case, 500 μm in radius), the bubble may split before collapsing (Fig. 3(b)). The fat tissue also moves closer to the bubble just before it collapses. We have also examined the effect of shock wave reflection at rigid interfaces such as bone. Figure 4(a) shows the collapse of a 10 μm near bone tissues (on top) after being hit by the lithotripter shock wave. The shock wave is reflected with 0.54 of the original amplitude at the bone interface. The bubble collapses at 0.148 μs. Figure 4(b) shows the simulation results without the consideration of the shock wave reflection. The bubble collapses only at 0.166 μs. The size of the jet is also significantly larger in this case.

**CONCLUSIONS** The interaction between a stationary bubble near various bio-materials and a lithotripter shock wave has been successfully modeled and simulated using the Boundary Element Method. High speed jets are developed in the bubbles in the direction of travel of the shock waves as previously reported in literature. It is therefore concluded that presence of the bio-materials causes the bubble to collapse faster and with slightly higher jet speed. However, when the initial bubble size is varied, different bubble dynamics is observed. In the case of very large bubble (500 μm in radius), the bubble does not collapse with a jet towards the bio-material, but will split into smaller bubbles. The reflection of the shock wave near rigid boundary such as bone or stone has the effect of causing the bubble to collapse faster with a narrower jet.

**Reference**

Sankin, G. N. & Zhong, P. (2006), ‘Interaction between shock wave and single inertial bubbles near an elastic boundary’, Phys. Rev. E 74, 046304.

**Fig. 1 (abstract O53).**
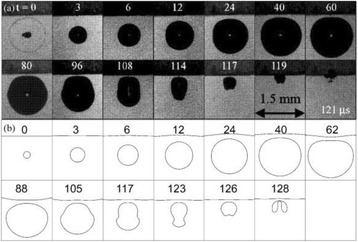
Interaction of an oscillating bubble near an elastic membrane. (a) Experimental results from Sankin & Zhong (2006). (b) Numerical simulation of the bubble collapse. Both bubble shapes and timing of events compare well with experiment

**Fig. 2 (abstract O53).**
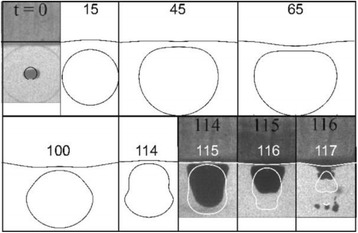
The interaction of a laser-generated bubble near an elastic membrane after being hit by a lithotripter shock wave at 114 μs. The numerical simulation results are overlapped on top of the experimental observations from Sankin & Zhong (2006). It is seen that in both simulation and experiment, the bubble splits as the membrane moves towards the bubble

**Fig. 3 (abstract O53).**
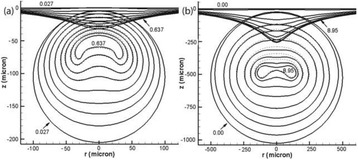
A gas bubble collapses near fat tissue (on top) when it is hit by a lithotripter shock wave from below. (a) The initial bubble size is 100 μm in radius. The bubble collapses with a jet towards the fat interface. The fat tissue moves towards the bubble. (b) The initial bubble radius is 500 μm. The bubble may split before collapsing. The fat tissue moves more towards this bigger bubble

**Fig. 4 (abstract O53).**
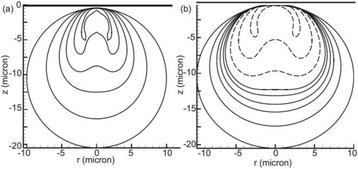
The effect of the shock waves reflection on the collapse of a bubble near bone tissue. The 10 μm bubble is located 10.5 μm from the bone interface. (a) The reflected shock wave is implemented as a downwards traveling wave 0.54 amplitude of the original lithotripter shock wave after it hits the bone interface. The corresponding time for each bubble shape (from outer to inner) is 0, 0.123, 0.137, 0.142, 0.146, and 0.148 μs. (b) Simulation results without shock wave reflection. The bubble collapses only at 0.166 μs. For the dotted line bubble shapes, time from outer to inner are 0.154, 0.160, 0.166 μs

### O54 Three-dimensional passive acoustic localization and mapping for cavitation: a preliminary study

#### S. Lu, X. Du, M. Wan

##### Biomedical Engineering, Xi’an Jiaotong University, Xi’an, China

###### **Correspondence:** S. Lu

**OBJECTIVES** Passive acoustic mapping (PAM) based on a linear array has been widely applied in real-time monitoring of high intensity focused ultrasound (HIFU) therapy. By applying a beamforming algorithm to the passively received acoustic emission of cavitation, the obtained spatial distribution of cavitation can be used for prediction of HIFU lesions. However, two-dimensional (2D) PA.

M can only provide a plane distribution of cavitation, which is not conducive to clinical diagnosis. PAM based on a hemispherical array has been used for three-dimensional (3D) vascular imaging with high resolution in the brain, but it is not suitable for treatment monitoring of other biological tissues, such as liver and kidney. This means that 3D PAM based on an area array for omnibearing monitoring of ultrasound therapy is required. The objective of this work is to develop a three-dimensional super-resolution passive imaging technique for microvessel and an omnibearing monitoring of ultrasound therapy in real time.

**METHODS** A multi-bubble model was used to create acoustic emissions of cavitation source for single bubble and multiple bubbles. The emissions were recorded by a 32 × 32 area array with an aperture size of 38.4 × 38.4 mm. For three-dimensional (3D) localization of single cavitation bubble, the differential times of arrival between elements at various positions and a reference element was firstly calculated by using cross-correlations. Then a paraboloid function derived by Fresnel approximation was used to fit time-delayed curved surface. Through least square fitting, the estimated paraboloid coefficients were used to calculate 3D position of single cavitation source. For passive mapping of extended cavitation region, a 3D passive beamforming algorithm based on time exposure acoustic (TEA) algorithm was applied to the 3D prebeamformed data to generate 3D cavitation images. In the algorithm, the source energy at each imaging location was calculated by integrating the square of the source strength (delay-and-sum of the prebeamformed data) over a time interval.

**RESULTS** Using a paraboloid to fit time-delayed curved surface can accurately localize single cavitation bubble at different positions in three-dimensional (3D) space, which can be used for super-resolution passive imaging of microvesssel. 3D Cavitatiom images of single bubble at (0 mm, 0 mm, 40 mm) has a full-width at half-maximum of 0.27mm × 0.27 mm × 2.03mm, which can be regarded as the point spread function at a fixed position of single cavitation source and given parameters of area array (Fig. 1). 3D cavitatiom images and its cross sections along three axes for multiple bubbles (distributed spatially with a normal distribution, the standard deviation of the distribution is 0.5 mm × 0.5 mm laterally and 1 mm axially) with different number (N = 20 and 50) demonstrated the feasibility of using 3D TEA-PAM to map extended cavitation region (Fig. 2). The “X-type” artifacts in 3D cavitation images were caused by multiple bubbles interfering with each other, there were no actual bubbles in the artifact region. The artifacts were needed to be improved by other technique such as adaptive beamformer. And it should be noted that the lateral resolution of 3D cavitation images is significantly better than axial resolution (Figs. 3, 4). The results can assist in real-time 3D monitoring of ultrasound therapy.

**CONCLUSIONS** 3D PAM based on TEA has the potential of providing a novel method for 3D real-time monitoring of ultrasound therapy.

**Fig. 1 (abstract O54).**
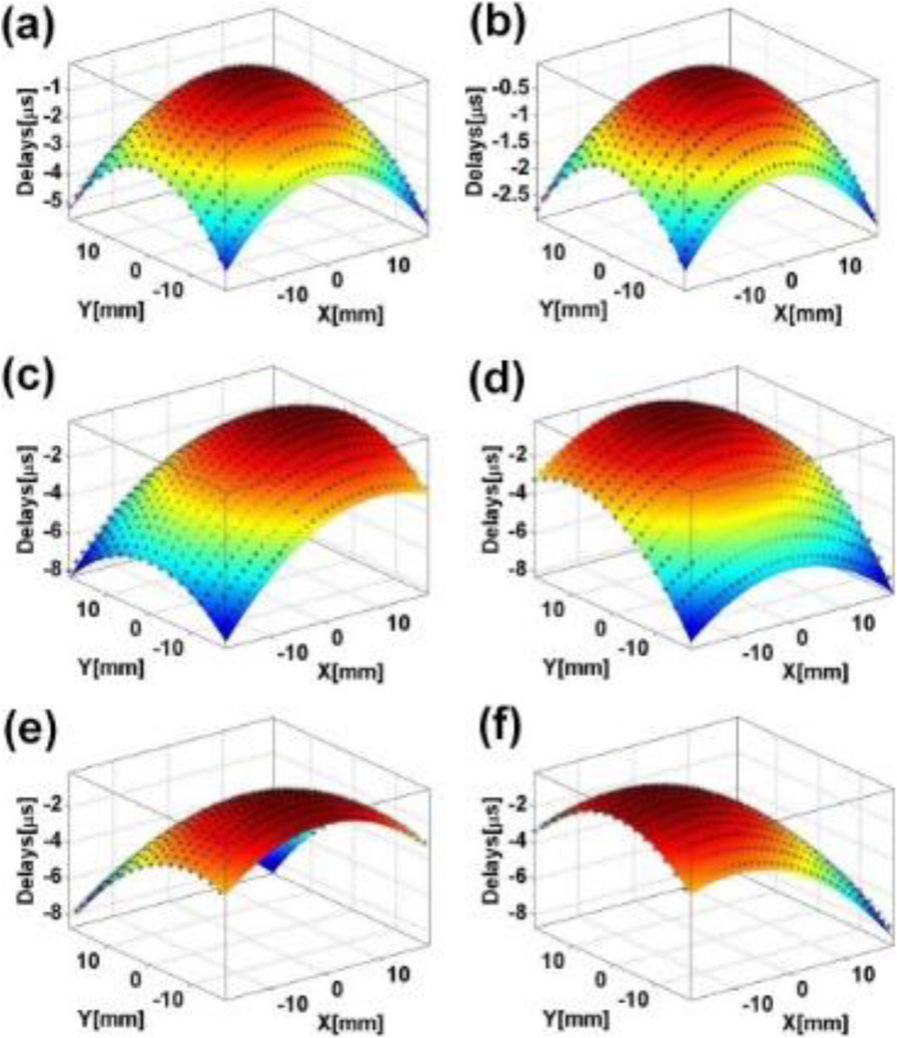
Time delay of the 3D prebeamformed data from single cavitation bubble and the corresponding fitting curved surface. The position of bubble is (a) (0 mm, 0 mm, 40 mm), (b) (0 mm, 0 mm, 80 mm), (c) (9.6 mm, 0 mm, 40 mm), (d) (0 mm, 9.6 mm, 40 mm), (e) (0 mm, -9.6 mm, 40 mm), and (f) (-9.6 mm, 0 mm, 40 mm), respectively

**Fig. 2 (abstract O54).**
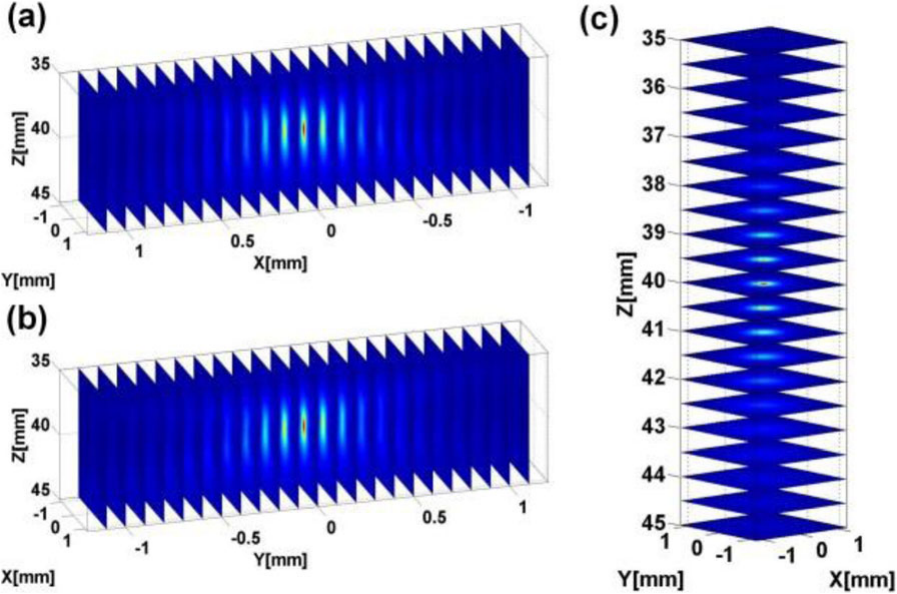
The slices of cavitation images using 3D TEA-PAM algorithms along (a) x axis, (b) y axis, and (c) z axis for single bubble. The position of bubble is (0 mm, 0 mm, 40 mm)

**Fig. 3 (abstract O54).**
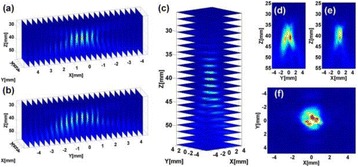
The slices of cavitation images using 3D TEA-PAM algorithms along (a) x axis, (b) y axis, and (c) z axis for multiple bubbles. The cross sections of cavitation images along (d) x axis, (e) y axis, and (f) z axis. The nominal distance from cavitation source to the area array is 40 mm. The number of cavitation bubble is 20, distributed spatially with a normal distribution

**Fig. 4 (abstract O54).**
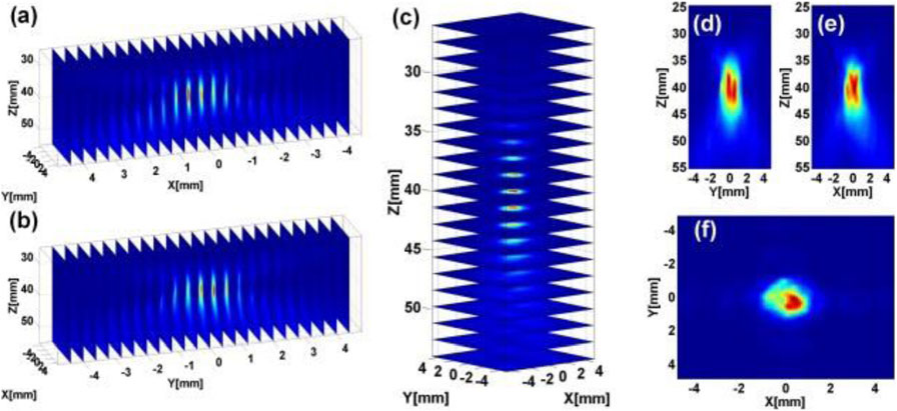
The slices of cavitation images using 3D TEA-PAM algorithms along (a) x axis, (b) y axis, and (c) z axis for multiple bubbles. The cross sections of cavitation images along (d) x axis, (e) y axis, and (f) z axis. The nominal distance from cavitation source to the area array is 40 mm. The number of cavitation bubble is 50, distributed spatially with a normal distribution

### O55 Towards a robust and general ultrasound propagation model for MR HIFU Tumour treatments: the hybrid ray tracer approach

#### D. Modena^1^, L. Sebeke^2^, M. Baragona^3^, A. Elevelt^4^, R. Maessen^3^, D. Bošnački^1^, H. Ten Eikelder^1^

##### ^1^Eindhoven University of Technology, Eindhoven, Netherlands; ^2^University Hospital Cologne, Cologne, Germany; ^3^Department of Multiphysics & Optics, Philips Research Eindhoven, Eindhoven, Netherlands; ^4^Department of Oncology Solutions, Philips Research Eindhoven, Eindhoven, Netherlands

###### **Correspondence:** D. Modena

**OBJECTIVES** Magnetic Resonance-guided high intensity focus ultrasound (MR HIFU) has shown to have a clinical relevance for the treatment of various types of tumours, such as uterine fibroids and bone metastases. MR imaging is used to plan and evaluate the HIFU treatment, whereas MR thermometry using proton resonance frequency (PRF) is used to monitor the procedure. There are different reasons why the development of ultrasound propagation models for MR-HIFU treatment is of interest. Particularly, they allow the improvement of pre-operative therapy planning by predicting the location and the amount of energy deposition during treatment as input for temperature evolution calculation. Furthermore, models can overcome the limitations of the PRF thermometry data, which can be acquired only in soft tissues and which present a low spatial resolution compared to the dimension of the treatment region. In addition, these models can enable model- based control during treatment. General accepted methods for HIFU propagation are the Far Field Approximation (FFA) and the Angular Spectrum Plane Wave method (ASPW). However, the first mentioned method is valid only when one homogeneous medium is present, and the second one is not applicable with no-parallel interfaces. Therefore, in this work we present and validate a ray tracer method, a fast and flexible (easily adaptable to complex geometries, for both soft tissues and bone cases) approach, which will be used as a baseline for an extension towards a patient-personalized HIFU propagation model.

**METHODS** Firstly, the hybrid stochastic ray tracer is presented (see Fig. 1). The model is suitable for the geometry of the 256-element phased array transducer (focal length 14 cm, frequency 1.2 MHz) used in the MR-HIFU system (Sonalleve V2, Philips, Vantaa, Finland). In the model, ultrasound propagation is represented by rays of intensities. The word ‘hybrid’ highlights the fact that the rays leave each transducer element in random directions with an initial intensity derived from the FFA formula. The rays follow the laws of refraction and reflection at an interface between two different media. The interference of ultrasound waves in the focal region is calculated from the phase assigned to each ray and the output of the model is the produced power density, which is represented by a 3D table with 0.2 mm grid size. Secondly, our model is compared with the ASPW method in terms of power produced in the focal region, in a configuration with two propagation media (lossless oil and tissue mimicking gel) with only flat surfaces (see Fig. 2). Thirdly, experimental validation data are acquired. In the experimental set-up the ultrasound waves travel through the lossless material and are focused in the tissue mimicking gel containing Zerdine (CIRS Model 054GS General Purpose Ultrasound Phantom). From the PRF thermometry, the temperature increases in the coronal slice crossing the focal point are recorded (see Fig. 3). Finally, for this configuration the hybrid ray tracer and the ASPW are used to compute the power density, which then functions as source term in the heat equation. The solution of this equation, found by a finite element approach using COMSOL Multhiphysics (COMSOL, Inc., Burlington, MA), can be compared with the experimental data. However, since the thermal parameters of the tissue-mimicking gel are unknown, these parameters are first found by fitting the results of the ray tracer/ASPW-heat equation to the experimental data. In particular, the diffusion coefficient D=λ_t_/(ρc_p_) (where λ_t_ stands for the thermal conductivity, ρ the density, c_p_ the specific heat at constant pressure) has been fitted by considering temperature data from the cooling down phase, and the term β= b/(ρc_p_) (where b represents the absorption coefficient, that is the fraction of power produced which contributes to the temperature increase) has been fitted taking into account the heating up phase.

**RESULTS** The hybrid ray tracer predicts a power density in good agreement with the reference method ASPW. After fitting the thermal parameters, the resulting model temperature prediction are in accordance with the experimental data. Moreover, the thermal parameters found by the fitting, are in the expected range.

**CONCLUSIONS** The hybrid ray tracer is a good starting point to model MR HIFU treatments, it has been developed to overcome the limitations of the other methods for ultrasound propagation, in particular the problematic treatment of no-flat surfaces. Moreover, through the computation of shear and longitudinal waves in the solid material, the method can be applied to the case of the treatment of bone metastases. Ultimately, the hybrid ray tracer discloses advantages such as flexibility and high calculation speed, which make this model a suitable first approach towards a patient- specific and general HIFU propagation model.

**Fig. 1 (abstract O55).**
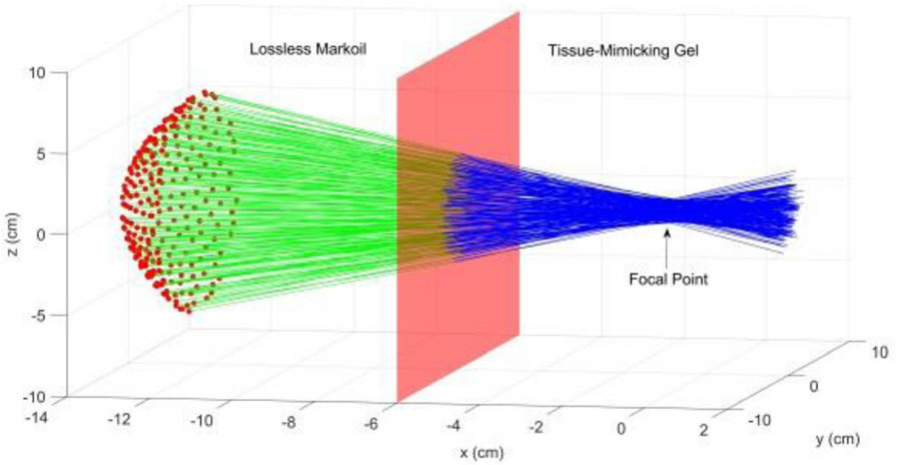
The hybrid ray tracer. In green the rays till the first interface, and in blue the refracted rays in the tissue- mimicking gel

**Fig. 2 (abstract O55).**
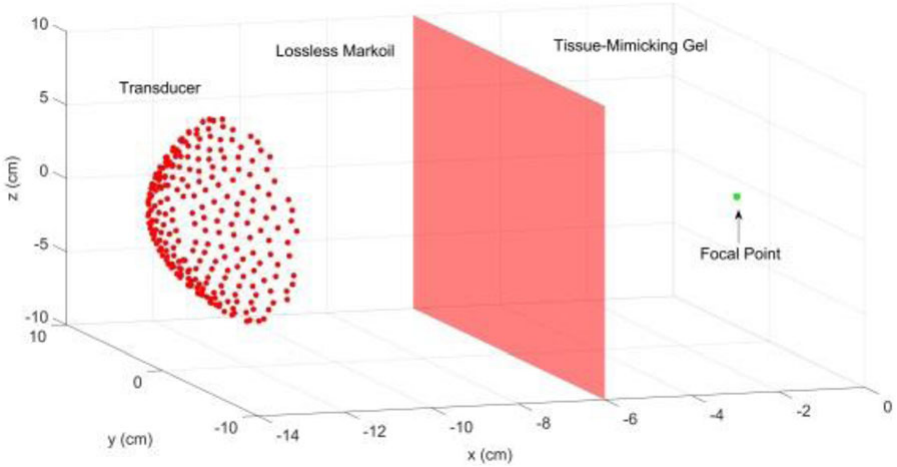
The ASPW and the hybrid ray tracer outputs are compared in a configuration with two homogeneous materials, separated by a flat interface

**Fig. 3 (abstract O55).**
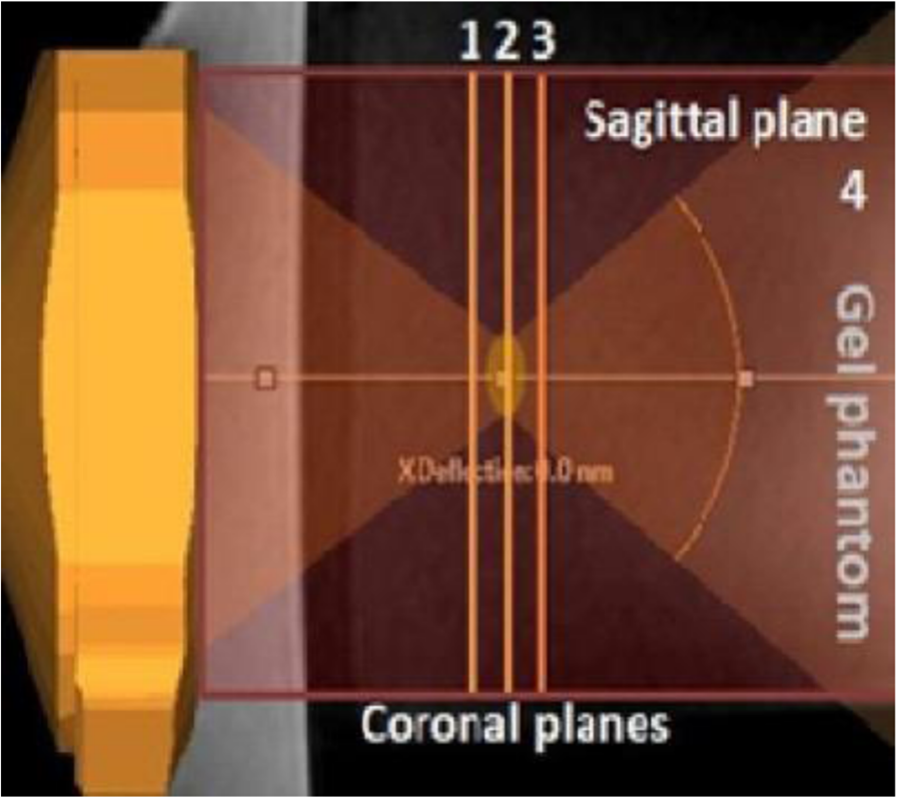
The coronal planes and the sagittal plane

### O56 Experimental validation of full-wave HIFU simulations in heterogeneous media

#### E. Martin, J. Robertson, B. Treeby

##### Medical Physics and Biomedical Engineering, University College London, London, UK

###### **Correspondence:** E. Martin

**OBJECTIVES** Numerical simulations have a wide variety of applications in high-intensity focused ultrasound (HIFU), including transducer design, patient selection, treatment verification, and treatment planning. However, for these simulations to be clinically useful, it is crucial that the accuracy of the numerical model is rigorously established under realistic conditions. The aim of this study was to quantitatively validate the wave models within the open-source k- Wave Toolbox using a series of experimental measurements made with heterogeneous fluid and solid objects in the beam path.

**METHODS** Experiments were performed in a 60 x 60 x 100 cm tank of temperature controlled, degassed, and deionised water. The acoustic field was generated by a single-element spherically focused HIFU transducer driven at 1.1 MHz with a 4 cycle burst. Signals were acquired using a calibrated 75 μm needle hydrophone connected to a 5- axis computer controlled positioning system. Planar scans were acquired both parallel and perpendicular to the beam axis following propagation through the heterogeneity. Measurements were made at several drive levels to give both linear and nonlinear conditions. Two types of medium inclusions were used to obstruct the beam path. The first was a series of fluid filled containers (rectangular, wedge, and planoconvex) constructed from laser cut Perspex and a stretched Mylar membrane. These were filled with either olive oil or glycerol, and were intended for validation of the models within a soft-tissue like medium. The second was a series of 3D printed and resin cast skull bone phantoms derived from a T1 MR image. These were intended for validation of the models when heterogeneities with a larger acoustic impedance mismatch are present. The transducer and medium inclusions were rigidly positioned using a series of custom-made Perspex and 3D printed mounts and opto-mechanical components attached to a breadboard mounted over the water tank. This allowed accurate registration of the source and object positions. For each experiment, a numerical simulation was conducted using the MPI version of the open-source k-Wave Toolbox running on the IT4I Salomon supercomputer. The grid parameters used 6 spatial points per minimum wavelength for the fluid objects, and 10 points for the solid objects. The source conditions were established using free- field measurements and linear acoustic holography. For measurements at other drive levels, the source pressure was assumed to scale linearly with drive voltage. The medium properties within the simulation were specified according to the known position and geometry of the scattering object, and using book values for the material properties. The measured and simulated wave fields were compared using several metrics, including the peak positive and negative focal pressure, focal volume, focal position, arrival time of reflections, and L2 error in the spatially varying wave field. For nonlinear conditions, the fields after spectral decomposition were also compared.

**RESULTS** The simulations and experiments showed close quantitative agreement. Errors were typically < 3% for the peak positive pressure, < 3% for the focal volume, < 1 mm for the focal position, < T/12 for the arrival times, and < 6% for the L2 error. The exception was the measurements made using the resin cast bone phantom, where simulations overestimated the focal volume by ~12%. This discrepancy is likely due to variations in the material properties of the resin from the casting process, which were not captured by the numerical model. For the nonlinear conditions, errors were smallest at the fundamental frequency, but still remained acceptable at the fifth harmonic (the highest harmonic measured with significant signal-to-noise).

**CONCLUSIONS** The results demonstrate that the full-wave models in the open-source k-Wave toolbox can accurately and quantitatively predict the time-varying ultrasound field generated by HIFU transducers in heterogeneous, absorbing media under both linear and nonlinear conditions. These models are likely to find many applications, from simple in silico investigations, through to patient specific treatment planning. It is important to note, however, the close agreement relies on accurate knowledge of the geometry, position, and material properties of the heterogeneities in the beam path. While this is possible in a laboratory situation, the sensitivity of the simulation output to these parameters is not yet fully understood. Further work is needed to clarify the uncertainties in numerical predictions using real biological tissue where the geometry and material properties may not be precisely known.

### O57 Translating microbubbles with millisecond scale ultrasound pulses: implications for controlled transport of bubbles to a boundary

#### C. Acconcia^1,2^, A. Wright^2^, D. Goertz^1,2^

##### ^1^University of Toronto, Toronto, Ontario, Canada; ^2^Sunnybrook Research Institute, Toronto, Ontario, Canada

###### **Correspondence:** C. Acconcia

**OBJECTIVES** To elicit bio-effects, ultrasound (US) stimulated microbubbles (MBs) must be in close proximity to their target. In a situation such as sonothrombolysis, however, the majority of circulating MBs will not be in proximity to the clot boundary. Radiation forces can potentially direct MBs to clot surfaces and recent work (Acconcia et al., JASA 2016) has suggested clot degradation patterns (Fig. 1A) could be influenced by ‘transport pulses’. However, this is a complex process involving a population of MBs flowing in a vessel, with size-dependent radiation and drag forces. We recently conducted experiments to investigate the process of bubble accumulation at a boundary with a view to improving exposure strategies in applications such as sonothrombolysis. The size and spatial distribution of bubbles arriving at a fibrin clot boundary (Fig. 1B) were found to be highly dependent on pressure and flow conditions. The ability to model and control this process is limited by a paucity of data on the translational dynamics of encapsulated MBs under the influence of millisecond scale US pulses. To date, experimental work for individual MBs has focused on the use of shorter (imaging) pulses. The purpose of this work is to directly investigate the translation of individual MBs with millisecond pulses in order to constrain theoretical modeling of this process.

**METHODS** In house developed optical tweezers integrated with a microscope (60x) and high speed camera were employed to select individual Definity MBs and position them in a fluid region away from boundaries. MBs were then subjected to a series of 1 ms length, 1 MHz pulses and were recorded (10 kframes/s) while translating laterally through the optical field of view. Image analysis was employed to quantify the displacements as a function of time with the primary metric of interest being the distance travelled within the first pulse. Exposures were conducted at transmit pressures of 25, 50, 100, 150 and 200 kPa. For each pressure a range of bubble sizes (1.5-9 microns in diameter) were assessed (n=86). The radial oscillations of encapsulated MBs were calculated based on a variant of the modified form of the Rayleigh-Plesset equation proposed by Marmottant et al (JASA 2005). This model requires as inputs estimates of shell elasticity, dilitational viscosity, and a starting effective surface tension. To assess displacement, radiation force along with other relevant forces on the MB (added mass, quasi-steady drag and history force) were solved numerically. The history force integral was evaluated using methods presented by Garbin et al. (2009), and Chung et al. (1982). After empirical modification, the history force integral has been generalized to apply over a larger range of Reynolds numbers. For this finite Reynolds number form of the history force (Takemura et al, JFM 2004), we used the numerical integration scheme proposed by Chung et al. (1982).

**RESULTS** The displacement as a function of MB size and pressure are shown in Fig. 1C. A pronounced feature of the displacement curves is the presence of an effective threshold point in size, below which there is only minimal translation. The threshold size increases with decreasing pressure. Note that single pulse displacements higher than 140 microns were not observed due to field of view constraints. The threshold dependant nature of the onset of rapid displacement could be well accounted for by the threshold dependant nature of oscillations captured by the encapsulated MB model. Example modeling is shown for the 25 kPa case (Fig. 1D), with a local maximum about a peak size, which correlates with the approximate resonant size of Definity at 1 MHz. The model results for up to 100 kPa were found to be in good agreement with data, when employing shell parameters that were within the range of those previously reported for phospholipid agents. Importantly, the inclusion of history force was required to accurately fit the data.

**CONCLUSIONS** This study reports the first size dependant data set for the translation of MBs under the influence of ms scale US pulses at therapeutically relevant pressures. A key feature was threshold size dependent displacements, where the degree of translation was highly sensitive to the radial asymmetry of the oscillations inherent with this model, a behavior that is characterized by compression dominated oscillations for smaller bubbles (<~3-4 microns). The majority of simulations to date have excluded history forces on the basis of Reynolds number arguments, however for the conditions investigated here the inclusion of history force was found to be important. When this constrained MB model was applied to our previously acquired data set of size dependant MB arrivals at a planar surface, good agreement was found, thereby providing a tool to investigate and optimize the control of translating MBs to a surface.

**Fig. 1 (abstract O57).**
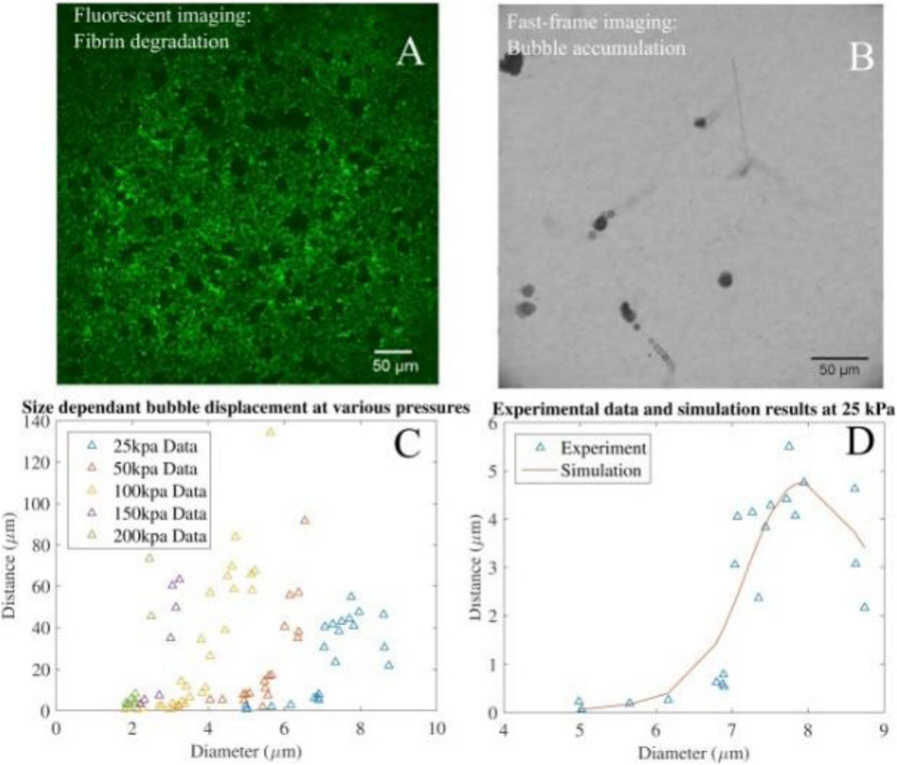
A) Two-photon microscopy of the erosion zone of the flourescently tagged fibrin network of a treated blood clot. Upon arrival, MBs penetrate and disrupt the fibrin network. B) Top view of the size dependant arrival of MBs at a planar fibrin clot boundary. A minimum intensity projection is shown over 100 US pulses. C) Size and pressure dependant displacement of MBs from a single 1 ms pulse. D) A comparison of data and modeling for the 25 kpa case

### O58 A fast non-invasive optical imaging thermometry method for HIFU

#### G. Oweis, H. Daoud

##### Mechanical Eng, American University of Beirut, Beirut, Lebanon

###### **Correspondence:** G. Oweis

**OBJECTIVES** To reduce hyperthermia treatment times, there has been a tendency to operate HIFU in short bursts, at high power levels. This necessitates the introduction of laboratory thermometry methods that are temporally and spatially resolved, and are able to characterize the burst of thermal output in real time. In this work we describe the development of a novel optical imaging method for temperature measurements in a HIFU field that is fast and spatially resolved. The method is non invasive and only requires optical access to operate.

**METHODS** The described thermometry method is based on the temperature-dependence of the optical refractive index in solids, gels, and liquids. As an example, water and polyachrylamide gel have a linear dependence of around k=-1x10^-4^/°C. When a laser ray travels through an axisymmetric HIFU heated spot, it will deflect from its straight unperturbed path. The net angular ray deflection is measured and converted to temperature using a direct formula derived from solving the paraxial eikonal ray equation for a Gaussian phase object. In the experimental setup, a 65 mm aperture HIFU transducer (Sonic Concepts) running at its third harmonic of 1.6 MHz was focused on an optically transparent tissue mimicking phantom cube of 2 cm side length placed in a water bath for acoustic coupling. A laser light sheet of 3 cm height was passed through a custom made comb to chop it into individual rays producing a planar bundle formation. The ray bundle was then focused with a cylindrical lens to allow as many rays as possible to pass through the 0.3 mm heated spot to improve the spatial resolution. The acoustic and optical axes were orthogonal. The ray bundle was imaged at 200 fps, at a location around 25 cm down the laser path from the HIFU spot (providing geometric advantage for imaging) during HIFU irradiation to capture the heating and cooling phases. The ray deflection angles from the individual images (relative to no HIFU image) were extracted and converted into radial temperature profiles. The tissue mimicking phantom cube was made by casting liquid silicone that cured in 24 hrs. K- type thermocouples (TC) of 0.13 mm dia. were cast within the phantom to read temperature. Figure 1 shows a picture of the HIFU installed in the experimental setup and a sample raw image of the ray bundle at the imaging location. Geometric triangulation was used to map the rays from the imaging plane to the HIFU focal plane.

**RESULTS** By comparing sequential ray images, ray deflection maps can be extracted as shown in Fig. 2. The resulting radial distribution of the angular deflection profile at the HIFU focal plane is also shown in the figure. The radial deflection profile is directly substituted in the aforementioed equation (shown in Fig. 2) to produce radial temperature profiels at different time steps as shown in Fig. 3. In this figure, the HIFU was operated to produce a 2 ms burst at a power level of around 20 W. As noted, the optical imaging thermometry is able to resolve the temperature distribution across the 2 mm HIFU heated spot giving a spatial resolution better than 0.1 mm. The temperature profile is narrow with a high peak right after HIFU exposure. The profile widens and cools off with time due to heat diffusion. In Fig. 4 the temporal evolution of the peak in the temperature distribution is plotted alongside the TC reading. The TC signal spikes higher than the optical ray temperature signal, expectedly due to viscous heating. The two curves agree well during the latter cooling phase. The temporal resolution of the method is fixed by the frame rate of the camera which in this case is 0.02 ms. Faster frame rates are straightforward to obtain, but in this case were limited by the available laser power illumination.

**CONCLUSIONS** A fast rise-time and spatially resolved optical imaging ray-bundle thermometry method has been developed and demonstrated with milliseconds long HIFU bursts in a tissue mimicking phantom. Unlike previously proposed thermometry methods such as laser-induced fluorescence which mainly works in liquids, the current ray bundle method works equally well in liquids and solid gels and it does not require the tedious calibration steps. It requires working with optically transparent materials. The simplicity of the experimental setup and ease of image processing, in combination with the availability of lasers and cameras in most laboratories makes this method a viable choice for fast and resolved characterization of HIFU thermal outputs.

**Fig. 1 (abstract O58).**
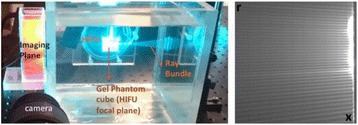
Experimental setup showing the HIFU in the water bath, the focused laser bundle illuminating the tissue phantom, and imager (left); and a sample raw ray bundle image in the imaging plane (right)

**Fig. 2 (abstract O58).**
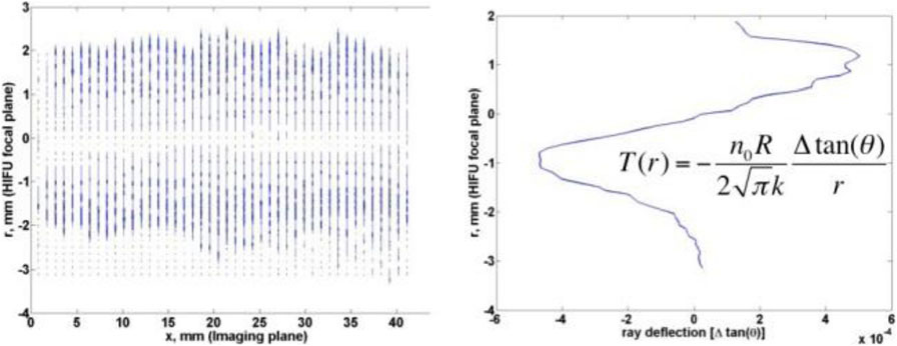
Two dimensional ray deflection map in the imaging plane (left); and the corresponding radial distribution (r) of the angular ray deflections in the HIFU focal plane (right); also shown is the temperature conversion equation

**Fig. 3 (abstract O58).**
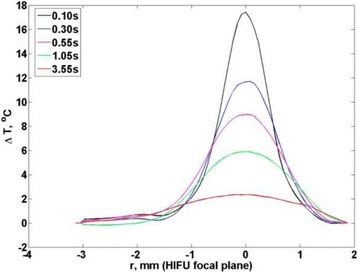
Radial temperature distribution of the HIFU heated spot at increasing times from the HIFU pulse

**Fig. 4 (abstract O58).**
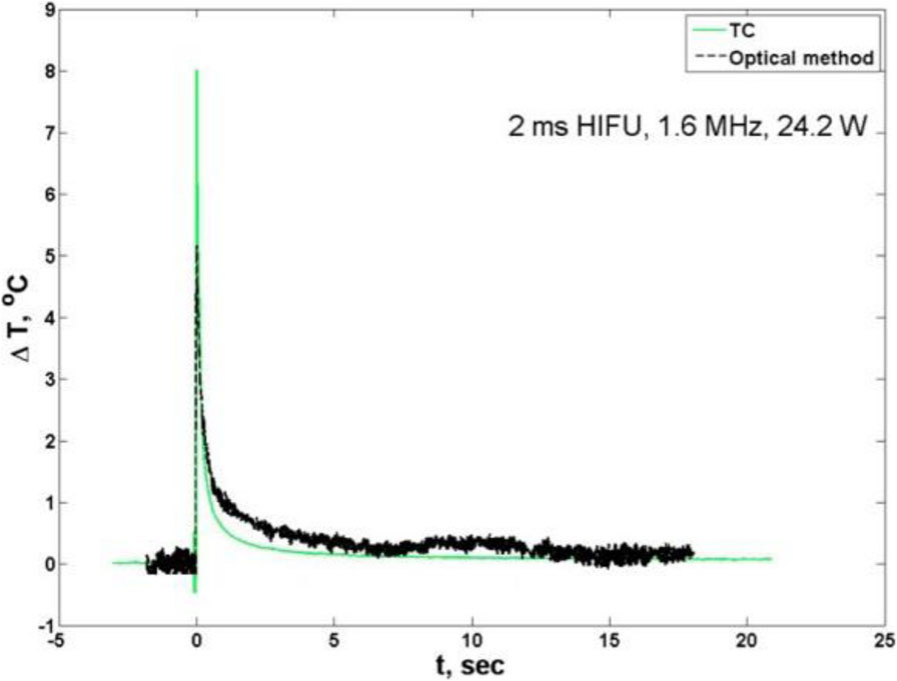
Temporal evolution of the peak temperature at the center of the heated spot using the optical ray method (black) alongside thermocouple readings (green)

### O59 *In vivo* and *ex vivo* monitoring of thermal ablation in a porcine model using ultrasonic nakagami imaging

#### S. Zhang, S. Shang, Y. Han, R. Xu, C. Gu, L. Zhang, M. Wan

##### Department of Biomedical Engineering, Xi'an Jiaotong University, Xi'an, Shaanxi, China

###### **Correspondence:** S. Zhang

**OBJECTIVES** The uses of high-intensity focused ultrasound (HIFU) and microwave ablation (MWA) as a non- invasive or minimally invasive therapeutic technique are being investigated due to the development of the monitoring imaging techniques. The acoustic posterior shadowing effects of cavitation and/or boiling bubbles influence the accuracy for defining the location of thermal lesions when using ultrasonic monitoring imaging. This *in vivo* and *ex vivo* study investigated the feasibility of using ultrasonic Nakagami imaging to evaluate the thermal lesions during HIFU and MWA in a porcine model.

**METHODS** 2-D RF data backscattered from the ablated region were captured by a modified diagnostic ultrasound scanner to estimate ultrasonic Nakagami parameters of the thermal lesions, and to reconstruct the ultrasonic B-mode and Nakagami images during HIFU and MWA. A term contrast-to-noise ratio (CNR) between the thermal lesions and the surrounding normal tissue is used to estimate the contrast resolution of the ultrasonic B-mode and ultrasonic parameter images.

**RESULTS** Unlike Ultrasonic B-mode images, Nakagami images were less affected by the shadow effect in monitoring of thermal ablation, and a fairly complete hyper-echoic region was observed in the Nakagami image. After thermal ablation, a bright hyper-echoic region appeared in ultrasonic Nakagami parameter images as an indicator of the thermal lesion. Mean values of the Nakagami parameter in the thermal lesion region increased to 0.58, 0.71 and 0.91 after 1, 3 and 5 min of thermal ablation. CNR values calculated for Nakagami parameter images increased from 0.13 to approximately 0.61 during thermal ablation and then decreased to 0.26 at the end of the post-ablation stage. The corresponding CNR values calculated for the ultrasonic B-mode images were 0.24, 0.42 and 0.17.

**CONCLUSIONS** This *in vivo* and *ex vivo* study on a porcine model suggested that the Nakagami parameter may have the potential use to evaluate the formation of thermal lesions and the ultrasonic Nakagami imaging may provide an alternative modality for monitoring HIFU and MWA treatment.

### O60 Changes in the optical scattering and absorption spectra of *ex-vivo* chicken breast tissue following exposure to HIFU

#### J.L. Raymond, R. Cleveland, R.A. Roy

##### Department of Engineering Science, University of Oxford, Oxford, UK

###### **Correspondence:** J.L. Raymond

**OBJECTIVES** Real-time acousto-optic (AO) sensing has been shown to non-invasively detect changes in *ex vivo* tissue optical properties during high-intensity focused ultrasound (HIFU) exposures. Baseline changes in optical properties have been previously measured as a function of thermal dose for chicken breast exposed to a temperature- controlled water bath (doi:10.1088/0031-9155/59/13/3249). In this work, the wavelength-dependent optical scattering and absorption coefficients of *ex vivo* chicken breast tissue exposed to HIFU were measured using an integrating sphere spectrophotometric technique.

**METHODS** Thin tissue sections (approximately 2 mm) were mounted on an acoustically transparent membrane such that the bottom surface was coupled to a 37°C water bath and the top surface exposed to air to permit non-contact thermal measurements using an infrared camera. Thermal damage was induced using a focused 1.1-MHz transducer (H-102; Sonic Concepts, Bothell, WA, USA) coupled to an acoustic lens and positioned in the water bath below the tissue sample. Phase-shifts produced by the lens de-focused the beam and resulted in an annular focal zone with the acoustic intensity maximum located 1 mm off-axis. Thus, a larger focal heating area could be produced in the tissue sample with less spatial variation in temperature (and thermal dose) in the region-of-interest (ROI) than for a tightly focused beam. Spatiotemporal surface temperature elevations were measured using an infrared camera (FLIR Systems, Kent, UK) and used to calculate the spatially-dependent thermal dose delivered to the tissue ROI. Optical property changes in the ROI were measured using a dual-beam UV-Vis-NIR spectrometer (Lambda 750s; PerkinElmer, Beaconsfield, UK) equipped with a 100 mm integrating sphere. The exposure intensity and time were varied in order to determine the optical property changes in tissue as a function of the delivered thermal dose.

**RESULTS** Figure 1 plots the optical reduced scattering coefficient (μ_s_') versus optical wavelength (λ) for 10 s exposures at 150, 200 and 250 W/cm^2^. In Fig. 2, the reduced scattering coefficient (μ_s_') at 975 nm is plotted as a function of the measured thermal dose for all sonications. Results show that HIFU-induced thermal damage results in changes in scattering at all optical wavelengths from 400-1300 nm (Fig. 1). Furthermore, the reduced optical scattering coefficient increases dramatically for exposures exceeding approximately 10^3 cumulative equivalent minutes at 43°C (CEM_43_) (Fig. 2).

**CONCLUSIONS** The apparent threshold for optical property changes in chicken breast tissue is broadly consistent with other studies of the thermal dose threshold for lesion formation. AO monitoring of HIFU therapy is feasible and this modality may be useful as an alternative to thermometry and dosimetery. Wavelength-dependent optical property changes can be used to improve the AO sensing of lesion formation during HIFU therapy. [Work supported by the F. V. Hunt Postdoctoral Fellowship of the Acoustical Society of America, the University of Oxford, and EPSRC grant number EP/K02020X/1].

**Fig. 1 (abstract O60).**
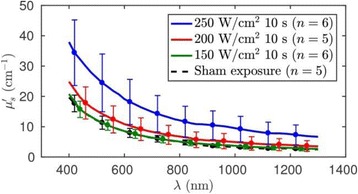
Optical reduced scattering coefficient (μ_s_') versus optical wavelength (λ) for 10 s HIFU exposures at 150, 200 and 250 W/cm^2^

**Fig. 2 (abstract O60).**
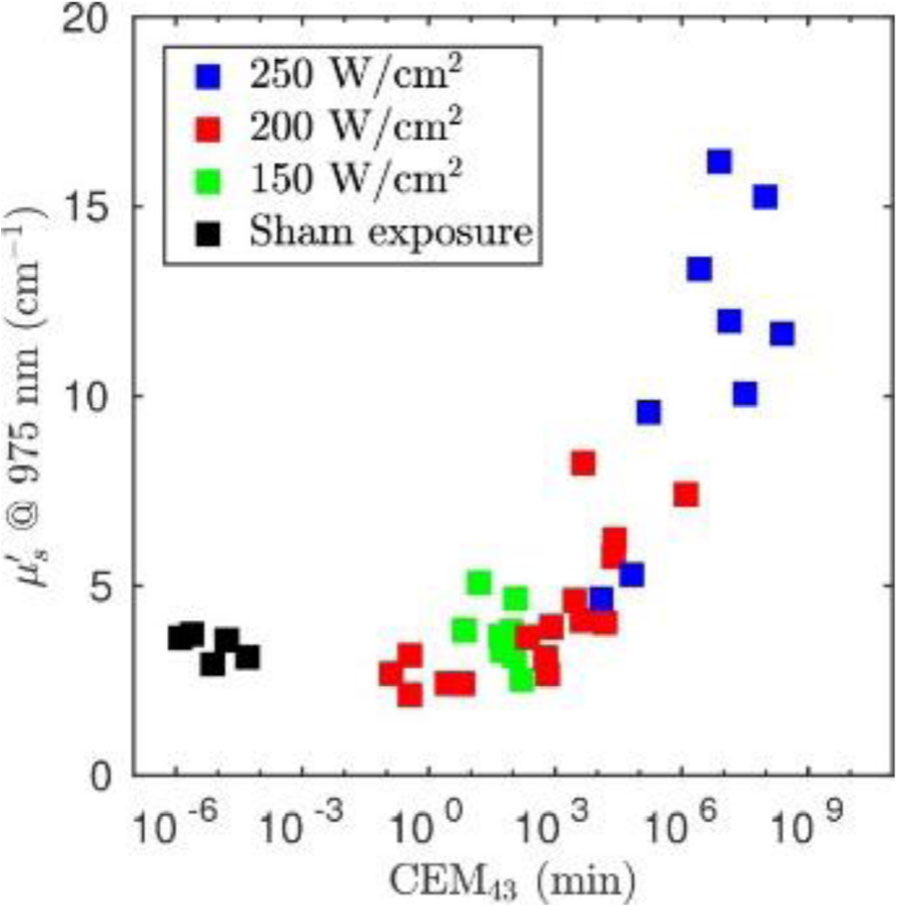
Optical reduced scattering coefficient (μ_s_') at 975 nm as a function of the measured thermal dose for all sonications

### O61 In vivo comparison of ultrasound and magnetic resonance thermometry for guidance of HIFU mild hyperthermia

#### R. Staruch^2, 1^, S. Sethuraman^2^, B. Cheng^1^, J. Kruecker^2^, R. Chopra^1^

##### ^1^Department of Radiology, UT Southwestern Medical Center, Dallas, Texas, USA; ^2^Ultrasound Imaging & Interventions, Philips Research North America, Cambridge, Massachusetts, USA

**OBJECTIVES** To determine the *in vivo* feasibility of using strain-based ultrasound (US) thermometry to monitor mild HIFU heating in muscle tissue, by direct comparison of temperature measurements made simultaneously using US strain estimation, magnetic resonance (MR) thermometry, and implanted optical sensors.

**METHODS** US thermometry in thigh muscle of anesthetized rabbits was performed in a 3T MRI (Ingenia, Philips) using a single-element 5 MHz US transducer (Y109, Sonic Concepts) fitted within the central aperture of a single- element 1.1 MHz HIFU transducer (H102, Sonic Concepts); both had a focal distance of 59 mm. A fiber-optic temperature sensor (T1C, Neoptix) was implanted into the thigh muscle; its location was visualized using 3D T1- weighted MRI (Fig. 1). Transducer location was controlled using an MR-compatible positioning system (RK100, FUS Instruments), to set the US focus at offsets of 0, 2, and 4 mm away from the sensor. US pulse-echo acquisition and HIFU energy deposition were interleaved using an open-architecture US system (Vantage 128 HIFU configuration, Verasonics). Filtered US signals were passed into the MR scan room through a grounded RF penetration panel. US echo shifts were tracked in the raw RF US data to derive thermally-induced strains which are proportional to temperature rise. MR temperature maps were calculated using the proton resonance frequency shift technique from RF-spoiled fast field-echo phase images (echo time 12 ms, in-plane resolution 2 mm, slice thickness 4 mm) acquired across and along the HIFU focus. The temporal resolutions of the MR, US, and fiber-optic acquisitions were 5, 1, and 1 seconds, respectively.

**RESULTS** Simultaneous US and MR temperature mapping data was successfully acquired and compared with invasive fiber-optic measurements during 15 HIFU sonications in 2 rabbits. MR temperature measurements in a 4 x 4 mm ROI at the location of the fiber optic sensor agreed well with optical measurements, with mean difference and temporal variation less than 0.5°C (Fig. 2). US thermal strain profiles acquired at the location of the HIFU focus 2 to 4 mm away from the sensor correlated well with sensor readings (R^2^ = 0.93 ± 0.03, Fig. 3).

**CONCLUSIONS** In *in vivo* rabbit muscle under normal respiration and perfusion, strain-based ultrasound thermometry is feasible in the mild hyperthermia range.

**Fig. 1 (abstract O61).**
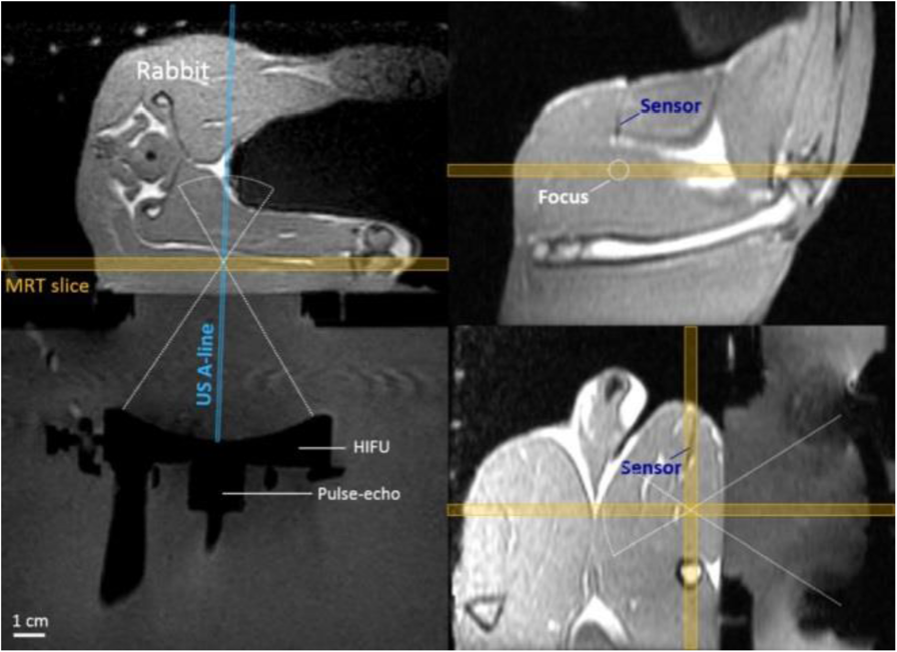
3D T1-weighted MRI of *in vivo* experiment setup for simultaneous ultrasound and MR thermometry in rabbits

**Fig. 2 (abstract O61).**
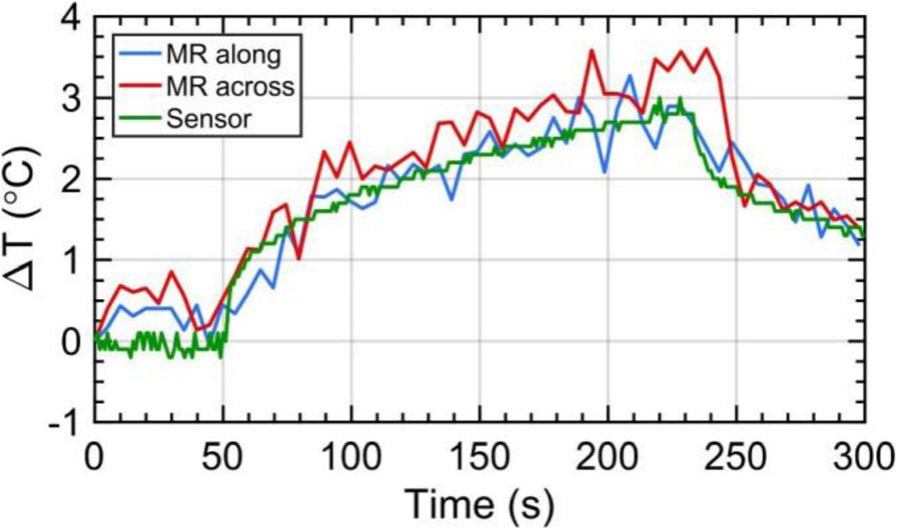
Agreement between MR thermometry and fiber optic sensor in image slices along and across the HIFU focus

**Fig. 3 (abstract O61).**
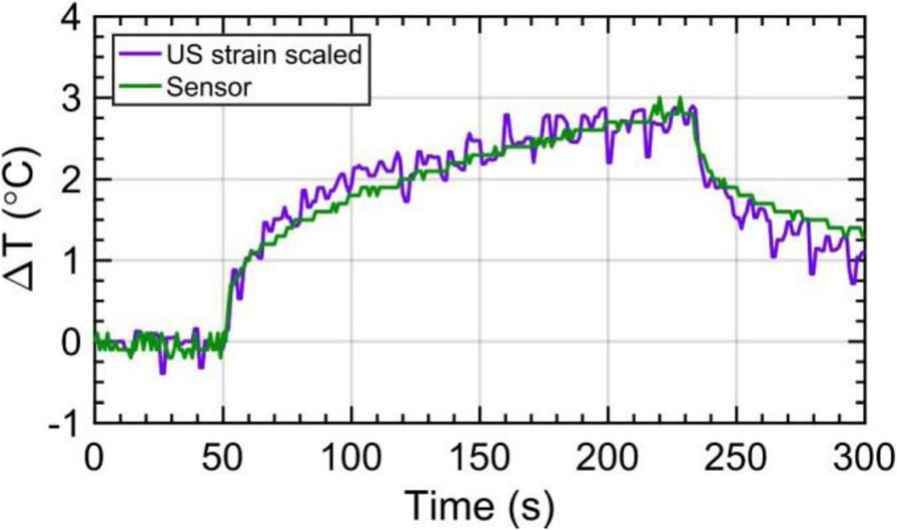
Agreement between scaled US thermal strain and fiber optic sensor in rabbit thigh

### O62 Changes in backscatter of liver tissue due to thermal heating can be used for guiding focused ultrasound ablations

#### V. Barrere^1,2^, D. Melodelima^1,2^

##### ^1^LabTAU, INSERM, Lyon, France; ^2^Université Claude Bernard, Lyon 1, Lyon, France

###### **Correspondence:** V. Barrere

**OBJECTIVES** High-intensity focused ultrasound (HIFU) is a noninvasive therapeutic modality concentrating ultrasonic energy in a small volume of biological tissues. Only in the focal volume the temperature rises above the threshold of thermal coagulation. A non-invasive modality is needed to effectively guide and monitor non-invasive HIFU treatments. Today, magnetic resonance imaging (MRI) and ultrasonic imaging are the two main modalities used in combination with HIFU for guiding the treatment. MRI is superior to ultrasound in visualizing tissue temperature and necrosis but this technique is highly expensive and lacks portability. Ultrasonic imaging has advantages in its inexpensiveness and portability. In addition, in most cases the ultrasound imaging probe is placed such that the imaging plane is aligned with the HIFU acoustic axis. Conventional B-mode imaging shows the spatial distribution in amplitude of the echoes reflected by acoustic impedance mismatches and is widely used to guide and monitor HIFU treatments. However, B-mode imaging provides limited information about the formation of coagulation necrosis due to HIFU. In most cases hyperechoes are visible due to microbubbles generated by either acoustic cavitation or boiling. One of the limitations is that tissue thermal coagulation is not always linked with microbubble generation. In addition, it is difficult to contour precisely the ablated zone based on these hyperechoes. Several methods have been proposed to characterize thermal change based on other parameters, such as ultrasonic backscatter. One of the oldest methods is the temperature estimation from the echo shift due to thermally induced change in speed of sound but is limited to temperature up to 50-55°C. Thermally induced change in ultrasonic attenuation has also been studied and used to monitor HIFU treatment, but the change in the attenuation coefficient is due to coagulation and not to the temperature rise. Techniques for estimating the elasticity of tissue are under also investigation based on the fact that tissue become stiffer when coagulated but not as a function of the temperature. In this study we investigated the change in ultrasonic backscattered energy due to the thermal coagulation itself without microbubble generation from 37°C and up to 70°C.

**METHODS** To minimize the cavitation nuclei in the tissue sample, the tissue was carefully degassed prior to HIFU exposure. A total of 26 experiments were performed in porcine liver. Liver samples were used because the knowledge of ultrasonic backscatter is largely described. The origin of ultrasonic backscatter from liver tissue is attributed to collagenous septae between liver lobules. Ultrasonic backscatter change due to cell death is attributed to the destruction of the cell nuclei. Ultrasonic RF signals at a center frequency of 2.5 MHz backscattered from the tissue before, during and after thermal coagulation due to HIFU exposure at 3MHz were obtained with a pulse-echo transducer and analyzed off-line. The tissue before, during and after the thermal coagulation was also examined by histology using an optical microscope. These results were then compared and discussed to clarify the mechanism of the backscattered energy change due to thermal coagulation induced by HIFU. Long exposure time (120 seconds) was used to observe smooth temperature increase from 37 to 70°C.

**RESULTS** The model predicted a linear increase 10 dB. A linear increase 8 dB was measured in ultrasound backscattered power during experiments. The tissue temperature increase estimated using backscattered energy correlated well (r=0.79) with temperature measurements performed using thermocouples. This linear relationship between changes in the backscattered energy and actual temperature was observed up to 70°C.

**CONCLUSIONS** Successful temperature estimation may allow creating 2D temperature maps during HIFU treatments.

### O63 Tumour characterization of human breast mastectomy specimens using Harmonic Motion Imaging (HMI)

#### Y. Han^1^, S. Wang^1^, T. Payen^1^, E. Konofagou^1,2^

##### ^1^BME, Columbia University, New York, New York, USA; ^2^Radiology, Columbia University, New York, New York, USA

###### **Correspondence:** Y. Han

**OBJECTIVES** Breast cancer is the most common cancer as well as the second leading cause of cancer death among women. There is a need to develop a breast imaging technique for reliable identification and differentiation of breast masses based on stiffness. Recently we have shown that Harmonic Motion Imaging (HMI) can be used to differentiate relative stiffness and monitor HIFU ablations in small lumpectomy human breast specimens. The objective of this study is to apply HMI on post-surgical mastectomy breast specimen with or without skin to mimic the *in vivo* environment and characterize tumor at different depth for better tumor localization and identification before and after HIFU treatment.

**METHODS** Collection and handling of postsurgical breast specimens were approved by the institutional review board (IRB) of Columbia University. Six post-surgical mastectomy breast specimens were obtained immediately after surgery for HMI imaging. The HMI setup consists of a 93-element, 4.5-MHz HIFU transducer confocally aligned with a 64-element 2.5-MHz phased array to transmit and receive through a 4-board VDAS system. The HIFU transducer was driven by an amplitude-modulated sinusoidal signal to vibrate the tissue at focal area. To generate a 2D/3D HMI displacement map, a point-by-point raster scan acquisition was used with a step size of 2 mm. At each spot, the focused ultrasound exposure was 0.06 s long (3-cycle oscillations at 50 Hz), during which 60 RF frames at 1-kHz pulse repetition frequency were acquired for cross-correlation.

**RESULTS** 60x15 mm HMI displacement maps could be generated to map the relative stiffness on the target area within 15 minutes (Fig. 1) indicating lower displacement in the tumor region and higher displacement in the peripheral tissue. In Fig. 1, the average peak-to-peak displacement in the tumor defined on the B-mode was found to equal 6.52±3.65 μm, and 38.70±21.79 μm in the surrounding tissue. A Student’s t-test showed significant difference (P < 0.0001) between the tumor and peripheral tissue in the HMI displacement. In the meanwhile, HMI displacement map showed larger relatively stiffer region compared to tumor region on the B-mode.

**CONCLUSIONS** HMI can successfully map the relative stiffness at variable depths on post-surgical human breast mastectomy specimens with or without the skin. The tumor on the HMI appeared slightly larger than the one delineated on the B-mode. This study laid the foundation for future clinical study on HMI guided focused ultrasound treatment.

**Fig. 1 (abstract O63).**
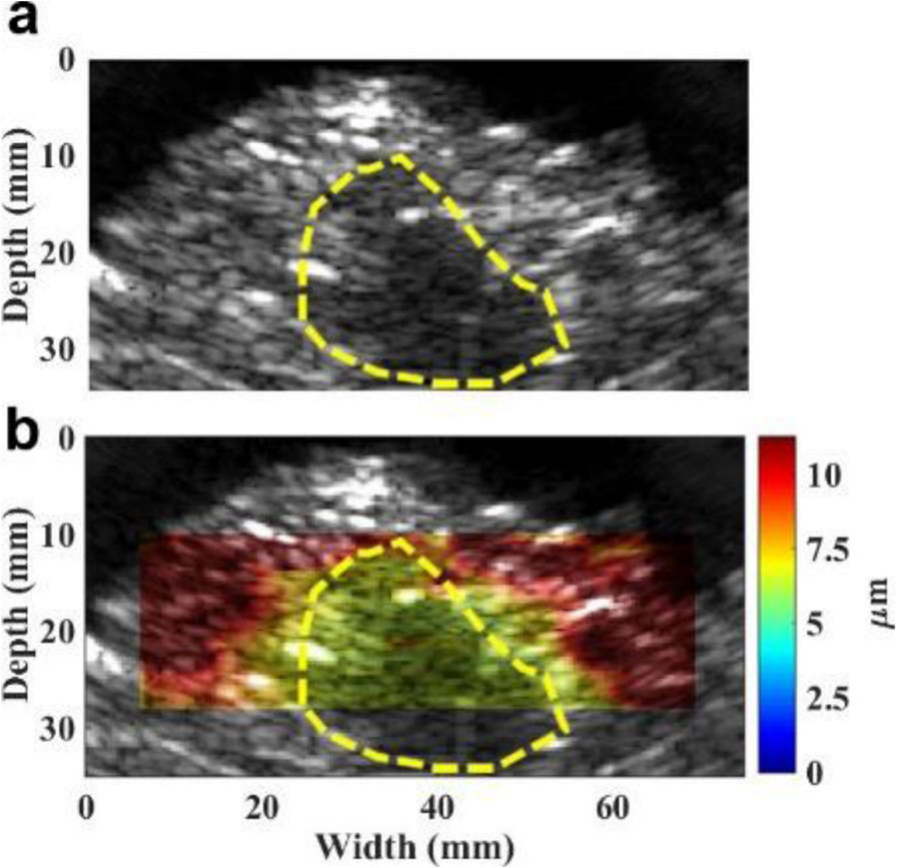
(a) B-mode image of a post-surgical breast mastectomy specimen with tumor appear dark in the yellow dash line. (b) HMI displacement overlay on B-mode image with color bar showing the HMI displacement. *The HMI scan was not completed due to time constraints according to the IRB protocol

### O64 Monitoring of nonlinear scattering during cavitation-enhanced ultrasonic heating for coagulation detection

#### S. Yoshizawa, K. Tomiyasu, R. Iwasaki, R. Takagi, S. Umemura

##### Tohoku University, Sendai, Japan

###### **Correspondence:** S. Yoshizawa

**OBJECTIVES** A noninvasive technique to monitor thermal lesion formation is necessary to ensure the accuracy and safety of high-intensity focused ultrasound (HIFU) treatment. Methods for the coagulation detection and temperature monitoring using ultrasound echo changes, such as the decorrelation of echo signals, have been investigated. On the other hand, methods for the enhancement of HIFU heating using cavitation bubbles have been investigated for the efficient thermal treatment. However, it is difficult to apply the monitoring methods using ultrasound echo changes during cavitation-enhanced HIFU heating because cavitation bubbles tend to produce random echo signals. The objective of this study is to develop a noninvasive technique to detect the thermal lesion formation in cavitation- enhanced ultrasonic heating. In this study, nonlinear components of scattered ultrasound from cavitation bubbles are analyzed and used for the coagulation detection.

**METHODS** Figure 1 shows a schematic of the experimental setup. A degassed chicken breast tissue was used as a target tissue. The water was kept at approximately 36°C. HIFU was generated by a 256-element array transducer (Imasonic) with both diameter and focal length of 120 mm. The transducer was connected to 128-ch staircase voltage amplifiers (Microsonic) by electrically combining each two adjacent elements and driven at 1 MHz. A phased array probe (Hitachi Aloka UST-52105) was set in the central hole of the HIFU transducer and connected to a programmable ultrasound imaging system (Verasonics Vantage 256).

Figure 2 shows the HIFU sequence consisting of high-intensity short pulses to generate bubble clouds, named “trigger pulses”, and following moderate-intensity long bursts for the enhancement of the ultrasonic heating, named “heating bursts”. The focal point of the trigger pulse was electronically scanned at each corner of a regular hexagon 3 mm each side and a ring focal region was generated employing a sector vortex method in the heating burst exposure to cover the six foci of the trigger pulse for the volumetric cavitation-enhanced heating. The total acoustic power for the trigger pulse and heating burst were 1800 and 90 W, respectively. The duration and interval time for trigger pulses at each focal point were 25 and 3 μs, respectively. The trigger pulses were laterally scanned for four times. For heating bursts, the duration and interval time for trigger pulses at each focal point were 5 ms and 4 μs, respectively. The focal spot was scanned 5 times. The subtotal durations of trigger pulses and heating bursts were 0.67 and 50 ms, respectively. Immediately after the end of the heating bursts, a 2-ms interval time was reserved for ultrasonic imaging with plane wave transmissions at a frequency of 1.88 MHz. Ultrasonic RF data were also acquired during the HIFU exposure for the passive coagulation detection.

**RESULTS** Figure 3 shows temporal change in spectral intensity calculated from the RF data during the HIFU exposure after the receive beamforming with the fixed focus at the HIFU geometric focus. The blue and red lines denotes the acoustic signal intensity at 1 and 2 MHz, respectively. Figure 4 shows pulse inversion (PI) images during the HIFU interval time just after HIFU duration started and at the moment when high brightness appeared, 8.0 s after the start of HIFU exposure. The result showed a good correlation between the intensity of the second harmonic HIFU echoes and the emerging high brightness in the PI image. The emerging high brightness was considered as boiling bubbles. It is inferred that the intensity of the second harmonic HIFU echoes increased due to newly generated cavitation bubbles which were caused by the trigger pulse reflected by the boiling bubbles. In other words, when the spectral peak was seen, the treatment region was heated sufficiently and the cavitation threshold in the surrounding region was deceased because of the relatively high temperature. By stopping the HIFU exposure at this moment, overheating would be avoided.

**CONCLUSIONS** In this study, the nonlinear scattering during cavitation-enhanced ultrasonic heating was monitored with an ultrasound imaging probe. The intensity of the second harmonic HIFU echo increased at the moment when high brightness appeared in the PI image, indicating the coagulation in the HIFU focal region. The results will be shown and discussed in the presentation when the HIFU exposure is automatically stopped by detecting the second harmonic intensity exceeded the certain threshold.

**Fig. 1 (abstract O64).**
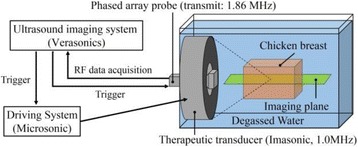
Schematic of experimental setup

**Fig. 2 (abstract O64).**
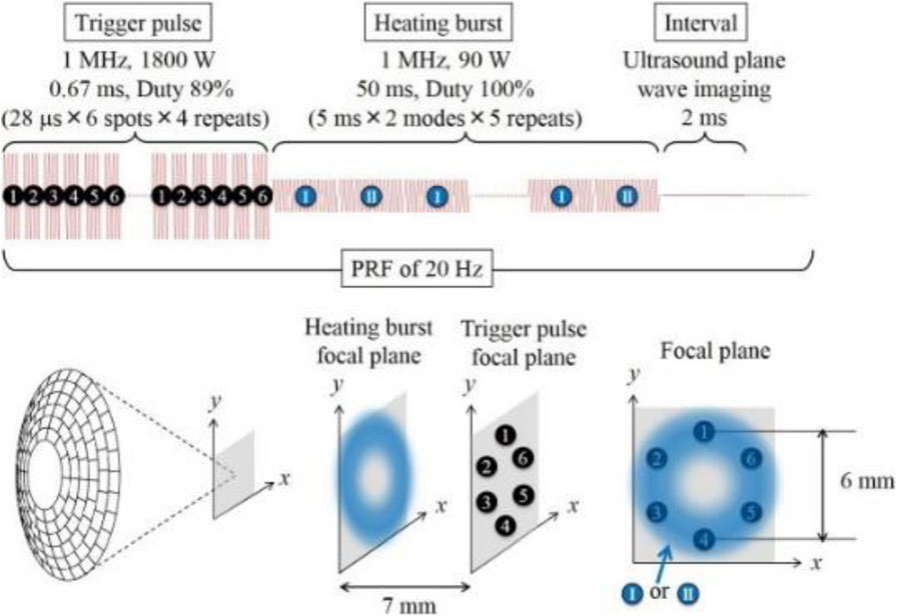
HIFU sequence consisting of high-intensity short pulses to generate bubble clouds and following moderate- intensity long bursts for the enhancement of the ultrasonic heating

**Fig. 3 (abstract O64).**
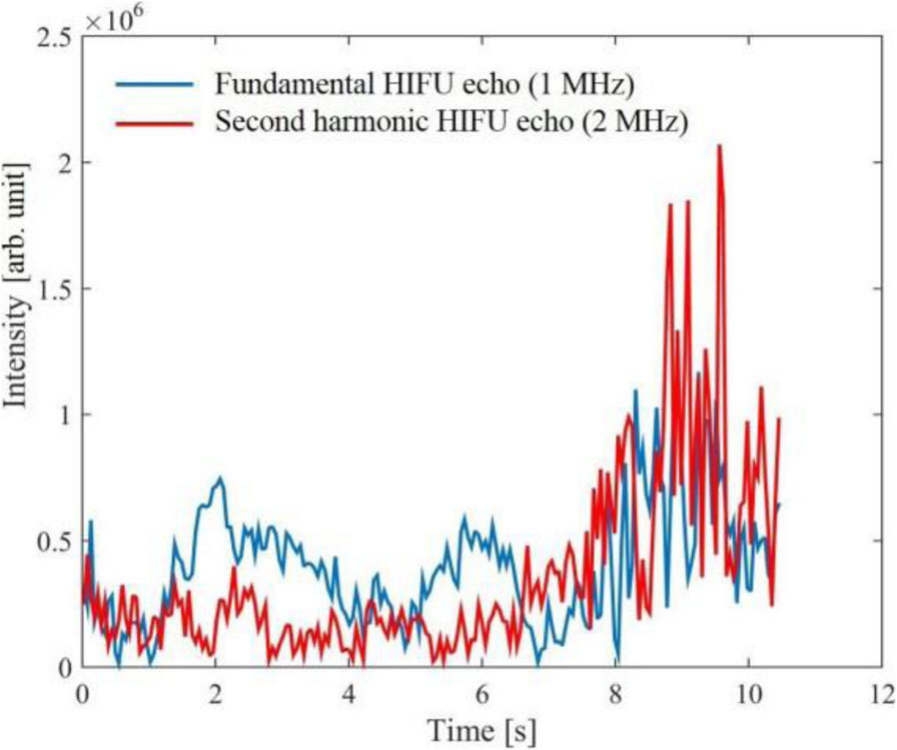
Spectral intensity calculated from the RF data during the HIFU exposure after the receive beamforming with the fixed focus at the HIFU geometric focus

**Fig. 4 (abstract O64).**
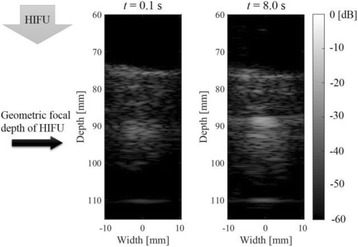
PI images during the HIFU interval time just after HIFU duration started and at the moment when high brightness appeared

### O65 Estimation and Compensation of *in-situ* ultrasound intensity using a 2D array therapy system and high frame rate imaging

#### Y. Peng^1,2^, B. He^1,2^, N. Deng^1,2^, X. Chen^1,2^, S. Chen^1,2^, C. Chin^1,2^

##### ^1^Biomedical Engineering, Shenzhen University, Shenzhen, Guangdong, China; ^2^National-Regional Key Technology Engineering Laboratory for Medical Ultrasound, Shenzhen, China

###### **Correspondence:** Y. Peng

**OBJECTIVES** We investigated the potentials for ultrasound to provide some patient-specific information which would improve delivery of optimal FUS treatment. Ultrasound had been shown to help locate the actual *in situ* focal point of FUS, and therefore, it is possible to compensate for navigational error due to beam distortion by the heterogeneous human body. However, ultrasound still cannot assist in determining correct ultrasound dosage in a realistic clinical setting. Microbubbles has been investigated as a biocompatible, internal “probe” to convert a local parameter to an echo characteristic that can be measured externally. We accessed the main challenge is that the multiple acoustic parameters are not easily isolated from the multiple measureable characteristics of the echo signals (such as frequency shifts and harmonic component). In order to isolate the multiple factors (such as attenuation and perfusion rate) contributing to measurable echo characteristics, we sought to exploit the highly specific behaviors of microbubble destruction when exposed to intense ultrasound. This paper reports a feasibility study of a pre-treatment scheme to determine effective attenuation and other relevant parameters and subsequently compensate for them during the actual therapeutic procedure.

**METHODS** A multi-channel transmission system and an array transducer was designed and built (Fig. 1). The current transmission system provides 128 physical channels that transmit arbitrary waveform with an analog bandwidth of 12 MHz at 4W sustained power or 50W peak power. The number of channels is scalable up to 1024. The transducer consists of a 125-element 2-D array operating at 2.1MHz. The complete system produces a high resolution focal spot of 0.8x0.8x5 mm, steerable to +/- 8 mm in any direction. Highly flexible treatment plans can be implemented with successive focal points can be targeted at an extremely high rate. Thus arbitrary exposure fields can be achieved. A phantom containing Sonovue ® microbubbles are exposed to the treatment beam. The treatment focus was scanned to expose an ROI of 8 mm diameter. A programmable high frame rate scanner system (Verasonic Vantage-128, USA) was used to capture echo data at very high spatial and temporal resolutions. A custom imaging sequence produced good quality B-scan frames at a rate of 1 kHz, which are interleaved between FUS exposure at various burst lengths and amplitudes. The treatment beam is normal to the imaging plane. Reconstructed echo image frames were analyzed to locate the treatment ROI automatically. The dynamics of the averaged echo signal in the ROI as functions of treatment parameters was recorded. The experiment was repeated with various attenuating layers added to the beam path between the target ROI and the FUS transducer. Four different layers with attenuation between 1.2 to 6.3 dB were used.

**RESULTS** The destruction of microbubbles by the treatment beam increases with treatment amplitude and burst length, as expected (Fig. 2). However, any single measurement cannot reliably reveal the *in situ* beam intensity without controlling the microbubble concentration. The data sets of destruction curves obtained with attenuating layers were matched to the un-attenuated reference data set using a multi-parameter fitting algorithm (Fig. 3). The resulting fitted beam intensity was found to match closely the actual values, verified by independent measurement of attenuation. The errors of *in situ* beam intensity measured with the proposed method were found to be less than 1.1 dB.

**CONCLUSIONS** Analyzing the complete destruction characteristics produced a feature-rich data set that can be fitted to more than one unknown parameters simultaneously. High frame rate imaging provides crucial speed advantage to this procedure. We proposed a scheme in which, at a pre-treatment phase, a patient is injected with microbubble contrast agent, exposed to low-dose ultrasound from the treatment device, have the destruction characteristics of the bubbles analyzed to ascertain ultrasound focus and *in-situ* intensity, and compensation to the therapy planning applied, before the actual course of treatment is applied. This study also demonstrated some capabilities of the in-house designed 2D array therapy system. Particularly interesting is that an arbitrary treatment ROI can be exposed in less than 32 ms. In this study the scanning speed of the treatment focus was exploited to ensure that the entire ROI is exposed evenly in between excessive imaging events.

**Fig. 1 (abstract O65).**
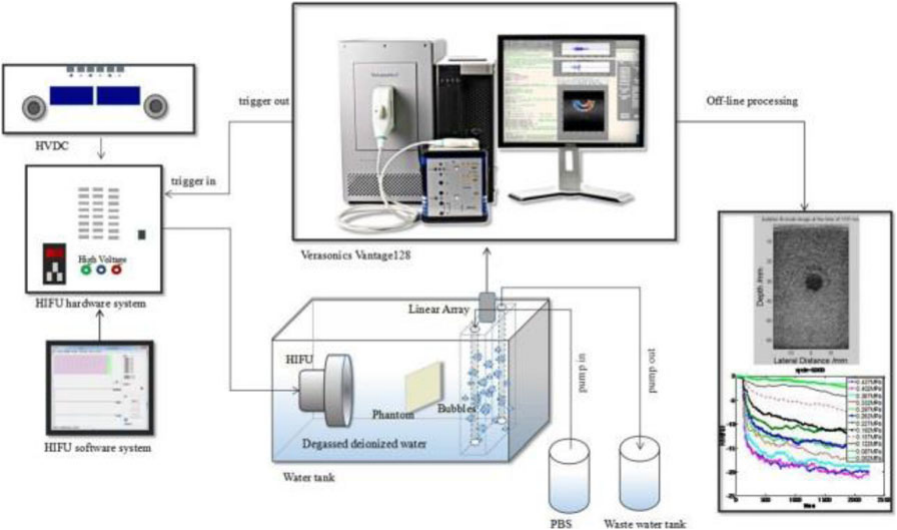
The experimental setup

**Fig. 2 (abstract O65).**
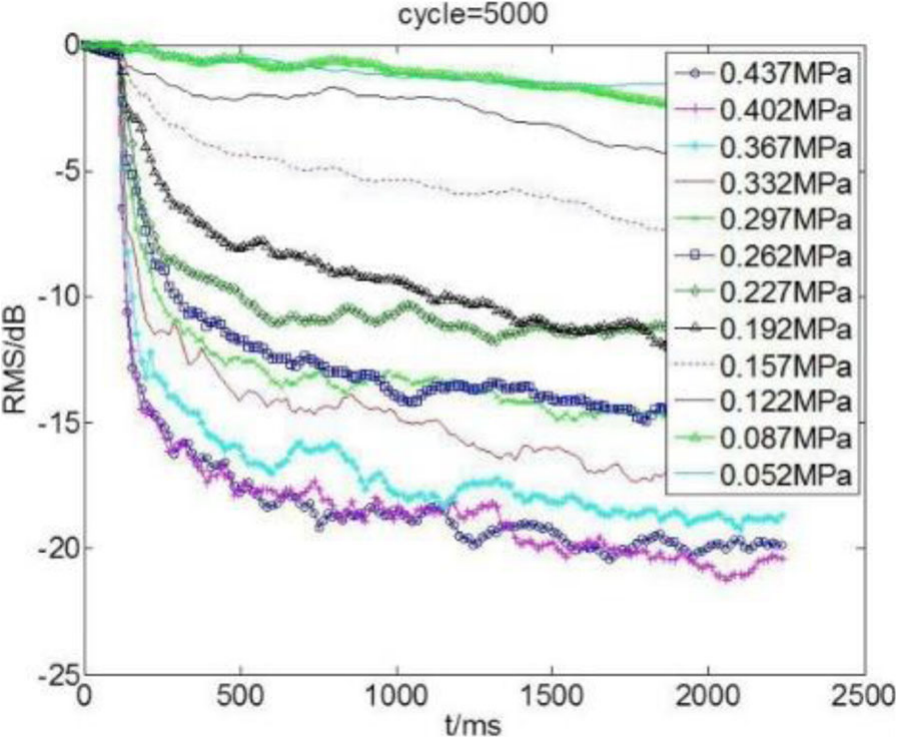
Destruction curves of microbubble in the treatment ROI for different excitation voltage at a specific burst length

**Fig. 3 (abstract O65).**
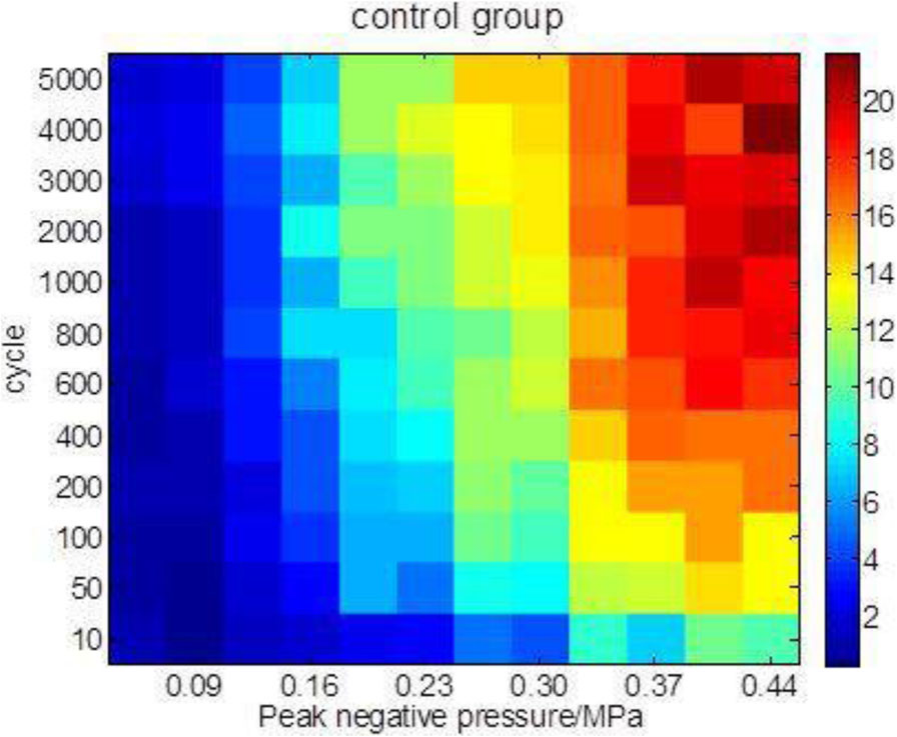
Extent of destruction measures at various incident intensities and burst lengths. Note that both axes are not linearly scaled

### O66 Enhanced sonodynamic therapy using oxygen-rich nano gas vesicle

#### Y. Yang, X. Hou, L. Sun

##### Interdisciplinary Division of Biomedical Engineering, Hong Kong Polytechnic University, Hong Kong, China

###### **Correspondence:** Y. Yang

**OBJECTIVES** Sonodynamic therapy (SDT), based on the synergistic effect of low intensity ultrasound and sonosensitizer, is a potential noninvasive approach for the treatment of cancers. Singlet oxygen, the major cytotoxic agent, is generated from dissolved oxygen within treatment region. However, because of the hypoxia microenvironment of solid tumor, oxygen deficiency restricts the singlet oxygen generation and consequently dramatically decrease therapeutic effect. To locally increase the oxygen level may potentially improve the treatment outcome. Here, we reported an oxygen enhanced SDT method utilizing oxygen-rich nano gas vesicle (Oxy-NGV). The objectives of this study is firstly to develop oxygen-rich nano gas vesicle and evaluate the oxygen releasing mechanism, then to investigate the improvement of the new oxygen-rich nano gas vesicle enhanced sonodynamic therapy as well as the underlying mechanism. If this Oxy-NGV based oxygen delivery strategy proven to be efficient, it will have great potential to extend applications to other oxygen based cancer therapy like radiotherapy, chemotherapy and photodynamic therapy.

**METHODS** Oxy-NGV was developed based on the nano gas vesicle (NGV) produced by cyanobacteria. It was then characterized regarding basic nanoparticle’s property and more importantly the oxygen releasing efficiency through being broken by ultrasound pulse (0.8 MPa). The therapeutic effect was evaluated by *in vitro* cytotoxicity by ultrasound treatment for 5 mins with the intensity of 5W/cm ^2^ after MCF-7 and HeLa tumor cells were incubated with sonosensitizer Protoporphyrin IX (PpIX) and Oxy-NGV. Then the singlet oxygen level, as the major cytotoxic agent, was imaged using Singlet Oxygen Sensor Green (SOSG) in both cell-free model and intracellular scenario. Meanwhile the oxygen level was also tested by dissolved oxygen meter compared with conventional SDT method. These studies were repeated in both normal oxygen level and hypoxia condition.

**RESULTS** Oxy-NGV was developed and characterized to be rod shape with 400 nm length and 100 nm diameter. Through cell-free experiment, oxygen inside oxy-NGV can be released by applying 0.8 MPa ultrasound pulse, consequently increasing oxygen concentration in solution immediately. Oxygen releasing rate and amount can be spatiotemporally controlled by ultrasound intensity. Cytotoxicity test in MCF-7 and HeLa cell shows increased cell death rate in Oxy-NGV mediated SDT group compared to pure PpIX one. The improvement of cell death rate is probably attributed to the increased singlet oxygen level as proven by increased SOSG fluorescence intensity. Meanwhile, both the cytotoxicity and singlet oxygen amount have positive correlation to the extracellular and intracellular oxygen level by changing Oxy-NGV concentration. In hopxia condition, the therapeutic improvement is more significant than normal oxygen condition.

**CONCLUSIONS** In summary, Oxy-NGV can efficiently deliver oxygen in a presicely controlled manner and consequently enhance SDT outcome through the mechanism of enhancing singlet oxygen production. NGV, as a novel stable nanometer size contrast agent and oxygen carrier, has great potential to enhance other oxygen mediated cancer therapy like radiotherapy, chemotherapy and photodynamic therapy.

### O67 Synergistic ablation of tumours *in vivo* by high-intensity focused ultrasound and ethanol

#### H. Murad, G. Halliburton, D. Luo, H. Yu, D. Khismatullin

##### Biomedical Engineering, Tulane University, New Orleans, Louisiana, USA

**OBJECTIVES** High-intensity focused ultrasound (HIFU) emerges as a powerful technology for noninvasive or minimally invasive non-ionizing treatment of cancer, with recent FDA approval. HIFU deposits a large amount of acoustic energy at the focal region within the target tissue (i.e., tumor), causing tissue heating and necrosis, a process known as thermal ablation. Noninvasiveness is an important advantage of HIFU over other thermal ablation methods, and our laboratory explores the ways for synergistic combination of HIFU with other therapeutic modalities to achieve the complete destruction of large and multifocal tumors. In this study we test the hypothesis that HIFU and percutaneous ethanol injection (PEI), a leading method for chemical ablation, have a synergistic effect on ablation of aggressive liver and prostate cancers *in vivo*.

**METHODS** This *in vivo* study was performed using the xenograft mouse models of human liver and prostate cancers. Hep3B human cancer cells and DU145 human prostate cancer cells (2.0×106) were injected on flanks of athymic nude mice. Tumors were allowed to grow to 8-10 mm size and then separated into the following treatment groups: HIFU alone, PEI (50%Etoh, 50 μl) alone, PEI+HIFU (50%Etoh, 50 μl), and sham. Tumor sizes were measured by caliper every day and a veterinary diagnostic ultrasound system was used pre-treatment, 5 days, and 12 days’ post- treatment. Tumor volumes were calculated from the ellipsoid formula V=πabc/6, where a, b, c are tumor sizes in three orthogonal directions. Tumors were surgically removed and fixed using 10% formaldehyde solution. Samples were sent for H&E staining with a single blinded pathologist, and live/dead percentages of tumor cross sections were determined at 5 and 12 days post treatment. Cryogenic-Scanning Electron Microscopy (Cryo-SEM) was also used to capture membrane disruption post HIFU+PEI exposure on DU145 prostate cancer cells.

**RESULTS** Tumor growth is significantly reduced or completely eliminated in tumors treated with HIFU in combination with PEI (Fig. 1). Tumors treated with HIFU alone showed a decrease in tumor size at 5 days, then rebounding to similar sizes as the sham. PEI alone tumors showed no significant reduction in size and continued to grow. Histology shows largest necrotic tissue area in tumors treated with PEI and HIFU at 5 and 12 days’ post treatment. Cryo-SEM images show large macropore (>1-2 micrometer) formation in cells treated with PEI+HIFU.

**CONCLUSIONS** The combination of HIFU and PEI shows a synergistic effect on tumor destruction at very low concentration of ethanol and lower acoustic power. This combination may become an effective minimally invasive treatment option that’s safer for patients with liver or prostate cancer. Utilizing these two FDA approved treatment in combination could lead to a disruptive and translational method of tumor treatment.

**Fig. 1 (abstract O67).**
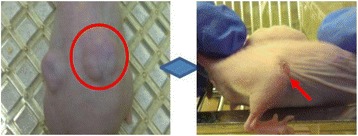
LEFT: Prostate tumor (8x9mm) before PEI+HIFU ablation, RIGHT: Tumor was eliminated at 2 weeks with no re-occurrence

**Fig. 2 (abstract O67).**
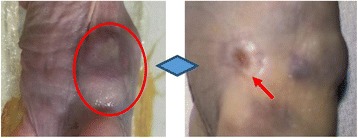
LEFT: Liver tumor (7x10mm) before PEI+HIFU ablation, RIGHT: Tumor was eliminated at 2 weeks with no reoccurrence

### O68 Multivariate ultrasound signal manipulation for effective gene delivery to pig livers

#### J. Harrang^1^, S. Song^1^, M. Kajimoto^1^, J. Chen^1^, R. Fu1, K. Morrison^2^, G. W. Keilman^2^, C. H. Miao^1,3^

##### ^1^Center for Immunity and Immunotherapies, Seattle Children's Research, Seattle, Washington, USA; ^2^Sonic Concepts Inc., Bothell, Washington, USA; ^3^Pediatrics, University of Washington, Seattle, Washington, USA

###### **Correspondence:** J. Harrang

**OBJECTIVES** Over several years our group has increasingly shown that ultrasound-mediated gene delivery (UMGD) can be enhanced by selecting favorable ultrasound (US) parameters. In particular, increasing pulse duration lowers the peak negative pressure (PNP) required for UMGD in mouse and cell models. This effect allows selection of conditions with minimal associated tissue damage and enables tuning to maximize the capabilities of piezo-materials. We have now further investigated scalability of this finding in pigs. In parallel, we examined several spatial effects which represent important considerations for eventual clinical application of this novel technology.

**METHODS** We have established an open surgery protocol used to explore optimal US parameters for efficient UMGD in pigs. First, the liver of each pig was exposed *via* a midline incision. Next, using contrast US to confirm placement and perfusion, we catheterized a specific branch of the portal vein. Just prior to therapeutic US exposure, the inferior vena cava was temporarily occluded. Then US exposure and infusion of a solution containing pGL4 plasmid and phospholipid microbubbles (MBs) were initiated simultaneously. Therapeutic US was delivered *via* either H105, an unfocused 52 mm disc transducer, or H185D, a 49 mm disc transducer with three cylindrical lenses, each focused to a depth of 20 mm from the exit plane. Our US pulse durations spanned 19 μs–22 ms, with PNPs spanning 0.6–6.9 MPa. 24 hours after surgery, pigs were sacrificed to harvest treated and control liver lobes. After sectioning, spatially- mapped samples were analyzed for luciferase expression.

**RESULTS** Our ongoing experiments have added further support for a species-generalized model that increasing pulse duration enables lower PNP for effective UMGD. Within a paired study, increasing pulse duration used with H185D from 19 μs to 200 μs at a constant 6.9 MPa PNP yielded up to 17-fold increases in sampled luciferase expression. In a repeated H185D study, we have also shown an increase in expression using lower-pressures and longer pulse durations (200 μs, 4.5 MPa and 2 ms, 2.7 MPa groups vs. the 19 μs, 6.9 MPa group). Nevertheless, pulse durations above 2 ms paired with lower pressure did not appear to further enhance expression. Despite these expression increases, ALT and AST values were comparable or lower in groups using longer pulse durations compared with groups using a 19 μs pulse duration. Experiments using H105 yielded a similar trend. Relative to an 18 μs, 2.7 MPa condition, we found increased expression in groups with conditions of 1 ms, 1.2 MPa; 4 ms, 0.8 MPa; as well as 22 ms, 0.5 MPa. Our 4 ms, 0.8 MPa group resulted in elevated AST levels, however changes in ALT and AST values of all other groups were nominal. From these experiments, we analyzed several spatial considerations between the two transducer designs. Of the two, the unfocused H105 treats a larger tissue volume simultaneously, but in exchange it generates lower PNPs at maximum operational power. Conversely, the cylindrically-focused H185D treats a condensed tissue volume which allows higher peak pressures at its operational limit. H105 outperformed H185D when comparing conditions of equivalent pulse duration and PNP, a reasonable result given H105’s larger volume. When instead comparing the two transducers in terms of average intensity across their entire active area, we found no obvious discrepancy in expression. However, when using H185D in its higher range of PNPs (4.5-6.9), individual tissue segments were observed to have higher maximum expression than the maximum values sampled using H105 at its limit PNP of 2.7 MPa. We are currently investigating whether this stems from the higher maximum PNP generated by H185D or whether using the transducer at those higher powers recruits greater UMGD efficacy from weakly-focused areas.

**CONCLUSIONS** By manipulating US pulse durations, our group achieved increased gene expression following UMGD in pigs. Such tuning has also allowed comparable expression at decreased PNPs, circumventing voltage limitations of piezo-materials. Since attenuation impedes high PNPs in transcutaneous UMGD applications, these results have promising implications for that modality. Our results demonstrate the advancement of efficient gene transfer in large animal models and we have also begun to elucidate the spatial requirements for effective UMGD.

### O69 Destruction of staphylococcus aureus biofilms on surgical mesh

#### T. A. Bigelow, H. Wu, C. Thomas

##### Iowa State University, Ames, Iowa, USA

###### **Correspondence:** T. A. Bigelow

**OBJECTIVES** It may be possible to significantly reduce the ~35,000 surgeries each year needed to replace infected surgical meshes following abdominal hernia repair. The use of a surgical mesh has become standard medical practice. However, mesh infection is a significant complication with incidence rates ranging from 1% to over 10%. Mesh infections require reoperation for mesh removal ~70% of the time resulting in the potential for hernia reoccurrence and the need for additional operations. Therefore, there is a critical need to develop new methods to noninvasively treat mesh infections without removing the mesh. Our goal is to develop ultrasound cavitation-based histotripsy to treat infections on surgical mesh.

**METHODS** S. aureus biofilms were grown on 1 cm square surgical mesh samples for 3 days. The samples were then rinsed with phosphate buffered saline prior to being inserted into Aquaflex Ultrasound Gel Pad Standoffs (Parker Laboratories Inc., Fairfield, NJ). The gel pads are bacteriostatic to minimize bacteria growth and have approximately the same mechanical properties as abdominal muscle. Mechanical properties have a significant impact on cavitation treatment (such as those used in our study), and therefore it is important they be relatively similar to real biological tissue. Each gel pad was cut in half allowing for two experiments per gel pad, and a slit was then cut in the gel pad to allow the insertion of the infected mesh sample. The focus of a spherically focused transducer (1.1 MHz, 12.9 cm focal length, 12.7 cm diameter) was then aligned on the mesh samples using a low-power signal from a pulser-reciever (Panametrics 5900, Olympus Corporation, Tokyo, Japan). Once aligned, the mesh samples were exposed to either a sham expsosure or histotripsy pulses (compressional pressure of 155 MPa, rarefactional pressure of 17 MPa) with tone burst durations of 3, 5, or 10 cycles at a pulse repetition frequency of 333 Hz for a duration of 15 seconds per exposure location with 5 repetitions per exposure group including the sham. The entire mesh was treated by scanning the focal spot in a raster pattern over the mesh using a step size of 750 μm. After treatment, the number of colony forming units (CFUs) on the mesh and the surrounding gel was independently determined.

**RESULTS** For the mesh samples (Fig. 1), sham exposures have statistically significantly more colony forming units than each of the treatment groups. The absence of a bar corresponds to when no CFUs were found. If we compare the means, we see a reduction of 2.00-log10 for the 3-cycle treatment, 3.25-log10 reduction for the 5-cycle treatment, and a 3.23-log10 reduction for the 10-cycle treatment. However, the differences between the 3-cycle treatment and 5- cycle/10-cycle treatments are not statistically significant once a Bonferroni correction has been applied. More observations are needed to determine if the 3-cycle exposures are significantly higher. The results from the gel pad (Fig. 2) showed no significant change in the number of CFUs for released bacteria from the biofilm for any of the treatments. However, the high variance in the numbers of CFUs for the gel samples means that while these numbers are not significantly different, we cannot claim that additional bacteria are not being released by the ultrasound exposures. Given that approximately 40% of the bacteria in the sham experiments were on the mesh, we would need to show that the bacteria in the gel are not statistically greater than this to make this claim. This would require more observations per treatment group.

**CONCLUSIONS** The ultrasound histotripsy treatments are effectively destroying most of the biofilm on the infected surgical mesh. We expect that further optimization of the exposure parameters will further enhance destruction of the biofilm.

**Fig. 1:**
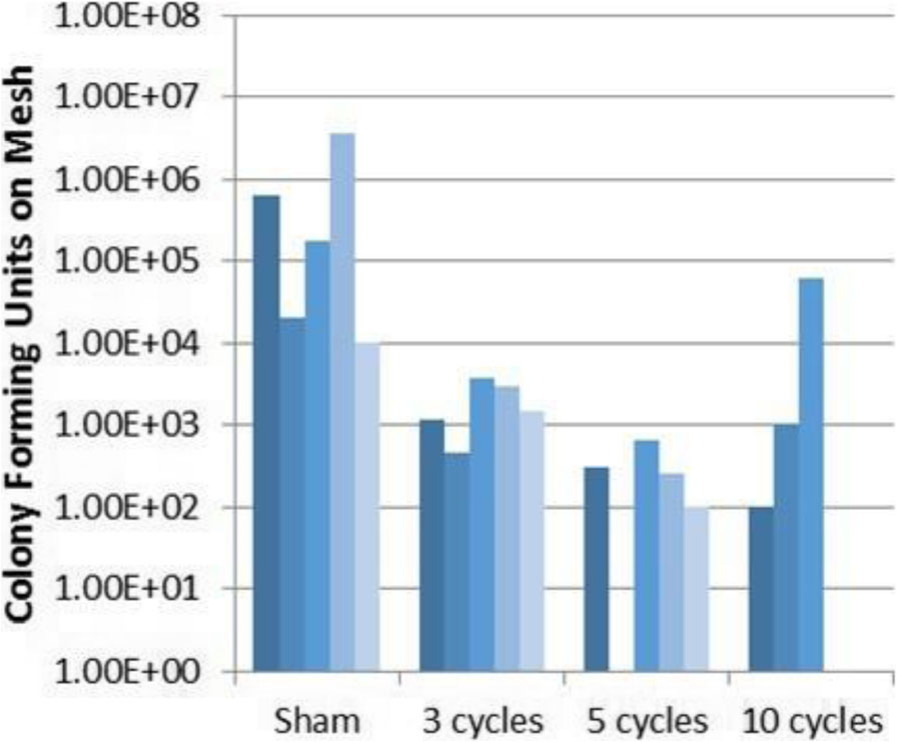
**(abract O69).** The number of S. aureus CFUs left on the mesh following the ultrasound exposures. The absence of a bar indicates no CFUs remained on the mesh

**Fig. 2:**
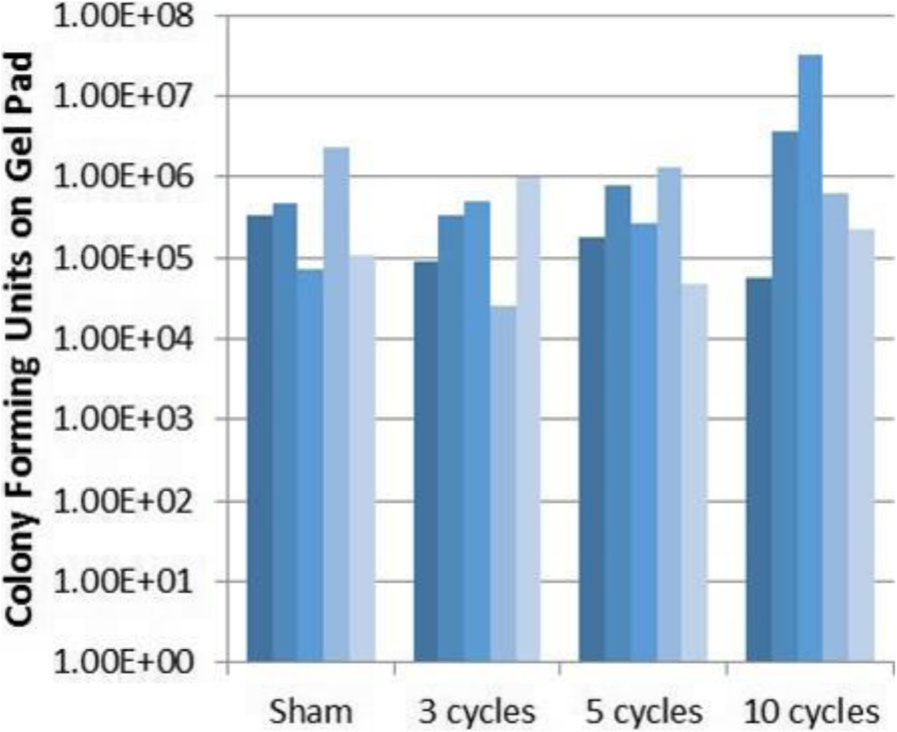
**(abract O69).** The number of S. aureus CFUs left on the gel pad following the ultrasound exposures

### O70 A special retinal ganglion cell responses to low-frequency focused ultrasound stimulation

#### H. Zhao, Q. Jiang, G. Li, M. Su, H. Zheng, W. Qiu

##### Shenzhen Institutes of Advanced Technology, Chinese Academy of Sciences, Shenzhen, China

###### **Correspondence:** H. Zhao

**OBJECTIVES** Acoustic retinal prosthesis has been put forward using high-frequency US with noninvasive and high- resolution advantages. But its application is limited by fabrication, energy consumption and mismatching to human eyeball’s anteroposterior axis. As our previous study demonstrated, the spatial resolution of low-frequency focused US (LFUS) can be improved by decreasing applied acoustic intensity. Thus we prefer the acoustic retinal prosthesis using LFUS and are interested in the electrophysiological properties of retinal ganglion cell (RGC) responses. This study has inspected the characteristics of one special type of RGCs’ responses to LFUS in comparison with their light responses, and examined the response changes in the presence of ON pathway blocker.

**METHODS** A 2.25 MHz focused US transducer (D=0.75 in., SF=2.0 in.) was used to stimulate retina which was cultured in a multi-electrode array system (MEA2100, MCS, Fig. 1a). The acoustic property was evaluated by hydrophone (UMS3, Precision acoustics). US stimulation was modulated at pulsed mode (Fig. 1b). Light stimulation was modulated in the same mode to give a uniform field flashes. The electrophysiological data collected from MEA was detected for neural spikes and sorted by Plexon Offline Sorter. Only channels recording single-cell activities were adopted for subsequent analysis. Peri-stimulus time histograms and raster plots were plotted for each RGC using Spike 2.

**RESULTS** LFUS can activate RGC and generate sustained excitation (Fig. 1c). A comparison between light and US- induced responses in the same RGC shows differences in response temporal pattern and polarity (Fig. 1d). Light produces sustained excitation at stimulus onset and prolonged inhibition at offset, which means the recorded RGC is ON-centered. But US elicits a transient peak of excitation followed by delayed sustained excitation at stimulus onset, and transient excitation at offset. 21 investigated RGCs show the same differences. A typical result of ON pathway blocking shows the delayed sustained excitation is eliminated by 100 μM L-AP4 (Fig. 1e). It is possible that this sustained excitation is generated from photoreceptors which transmission to bipolar cells is blocked. The exemption of ON- and OFF-transient excitation are probably because US directly activates interneurons or RGCs in retinal neural circuits.

**CONCLUSIONS** We have investigated the neurophysiological properties of RGC responses to LFUS. We have discovered some new temporal response patterns of RGCs that haven’t been reported previously, including the characteristic dual-peak response patterns in ON-sustained RGCs and transient RGCs. In addition, we have found that US can modulate the temporal-spatial characteristics of RGC firing activities, which suggests that US can encode the information transmitted by RGCs. These results of our study will provide an important foundation for the development of ARP.

**Fig. 1 (abstract O70).**
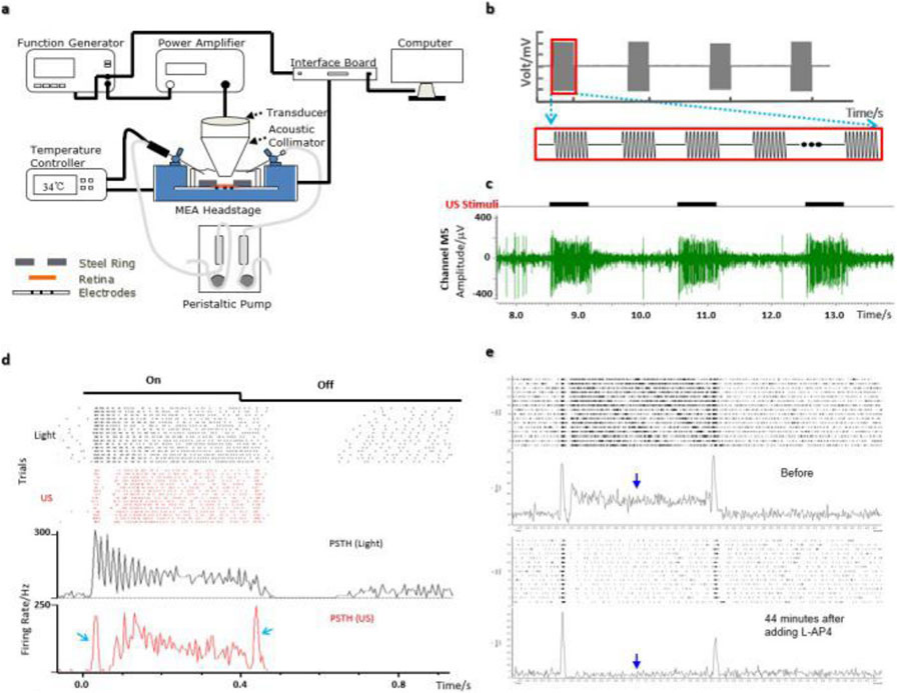
See text for description

### O71 Guided longer pulses from a diagnostic ultrasound and intraclot microbubbles enhanced catheter directed thrombolysis

#### S. Gao^1^, Q. Zhu^1^, X. Dong^1^, Z. Chen^1^, Z. Liu^1^, F. Xie^2^

##### ^1^Department of Ultrasound, Xinqiao Hospital, Third Military Medical University, Chongqing, China; ^2^Internal Medicine Cardiology, University of Nebraska Medical Center, Omaha, Nebraska, USA

###### **Correspondence:** S. Gao

**OBJECTIVES** Insufficiency of MB in and around the vessel-obstructing thrombi significantly reduces the effectiveness of ultrasound assisted thrombolysis (UT). Combined with intraclot infusion of MB, guided longer pulses ultrasound from a diagnostic transducer should be able to improve catheter directed thrombolysis (CDT) procedure.

**METHODS** In a thrombo-embolised rabbit IVC model, parallel to catheter directed rt-PA thrombolysis procedure, guided moderate MI longer pulses from a modified diagnostic ultrasound transducer combined with an intraclot infusion of MB were aplied to facilitate CDT. The thrombolysis efficacy score, pre and post-treatment plasma concentration level of D-Dimer, a product of fibrinolysis, were acquired and compared in the four groups (CDT+UT, CDT alone, UT alone, & control).

**RESULTS** The higher thrombolysis efficacy score (Fig. 1) and consistent elevated post-treatment plasma concentration level of D-Dimer (Fig. 2), both indicated a superior of CDT + UT over CDT/UT alone. There were no evidences of thrombo-embolism or local thrombus formation in the cardiac-pulmonary vessels.

**CONCLUSIONS** Combined with intraclot infusion of MB, guided longer pulses from a diagnostic transducer was able to improve catheter directed thrombolysis procedure. This strategy has a possibility to achieve earlier clot removal, lower dosage of thrombolytic agent administrated, and in consequence lower incidence of thrombolysis related side effects.

**Fig. 1 (abstract O71).**
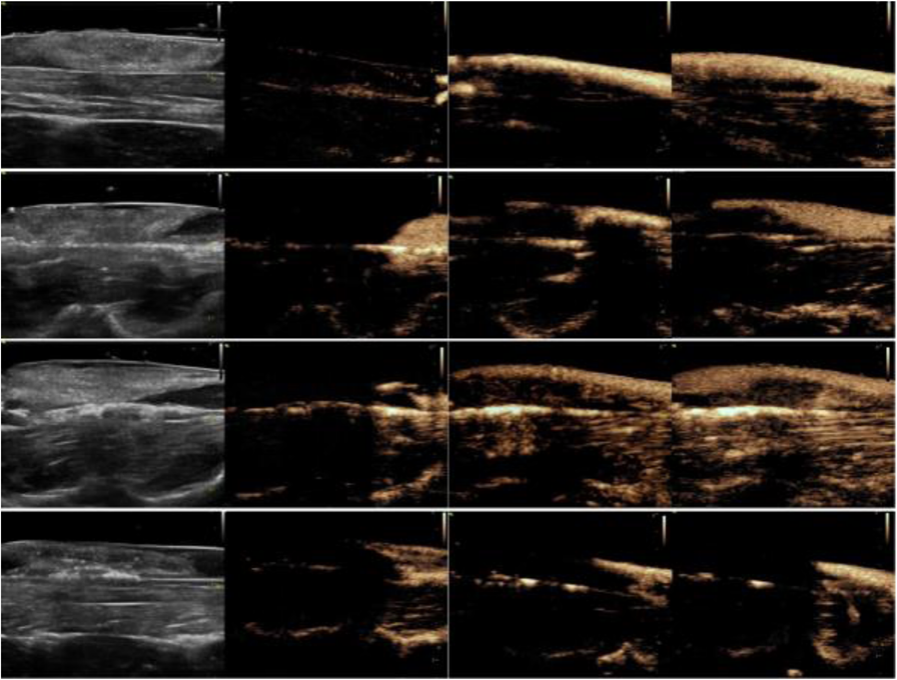
See text for description

**Fig. 2 (abstract O71).**
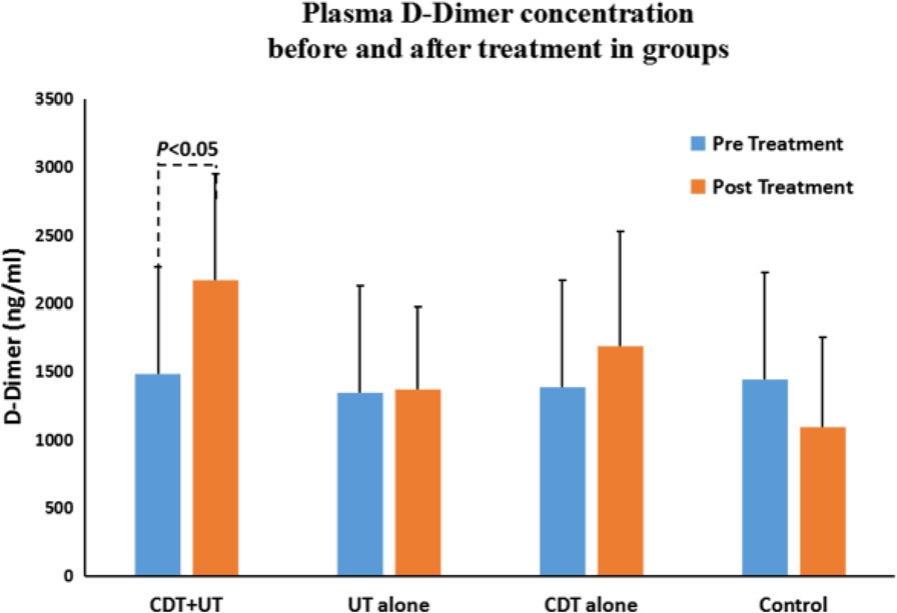
See text for description

### O72 Effects of f-number on the histotripsy intrinsic threshold and cavitation bubble cloud behaviour

#### E. Vlaisavljevich, T. Gerhardson, T. Hall, Z. Xu

##### University of Michigan, Ann Arbor, Michigan, USA

###### **Correspondence:** E. Vlaisavljevich

**OBJECTIVES** Histotripsy is an ultrasonic ablation method that fractionates soft tissue through the precise control of acoustic cavitation. Previous work has demonstrated that a cavitation cloud can be formed by a single acoustic pulse with one high amplitude negative cycle when the negative pressure exceeds an intrinsic threshold of ~25-30 MPa. Although previous work has provided significant insight into the process of intrinsic threshold histotripsy, the majority of these studies have used highly focused (i.e. f-number<0.6) transducers. In this study, we investigate the effects of f- number on the histotripsy intrinsic threshold and cavitation bubble cloud behavior, which is essential to the development of histotripsy for different clinical applications.

**METHODS** The effects of f-number on the histotripsy intrinsic threshold and cavitation bubble cloud behavior were investigated using a 235-element 500 kHz array transducer, with the effective f-number of the transducer varied from 0.51 to 0.89 by changing the active elements in the array. Ultrasound pulses of 1-2 acoustic cycles were applied to tissue mimicking phantoms, and the resulting cavitation activity was detected and characterized by passive cavitation detection and high-speed photography (Phantom V210, Vision Research). The intrinsic threshold at each f-number was defined as the pressure at which the probability of generating cavitation was 0.5. Optical images were further analyzed to determine the effect f-number on bubble cloud characteristics including the bubble cloud dimensions, the “bubble density” within the cloud, and individual bubble size. Finally, the effect of f-number on histotripsy fractionation efficiency was investigated by applying histotripsy to tissue phantoms embedded with a layer of red blood cells, with the resulting fractionation visualized using optical imaging.

**RESULTS** The intrinsic threshold did not significantly change with f-number, with the threshold remaining ~27-30 MPa for all conditions (Fig. 1A). The predictability of intrinsic threshold histotripsy was further demonstrated by experiments showing close agreement between the predicted and experimentally measured bubble cloud dimensions for all f-numbers. Quantifying the size of individual bubbles formed directly above the intrinsic threshold at different f- numbers showed no significant change in bubble size (~300 μm) with f-number. Comparing bubble clouds at different f-numbers showed a significant reduction in the “bubble density” with increasing f-number, ranging from 39.6±3.8 bubbles/mm^2^ for an f-number of 0.51 to 1.5±0.3 bubbles/mm^2^ for an f-number of 0.89 (Fig. 1B). Finally, experiments comparing the efficiency of histotripsy ablation demonstrated a significant increase in treatment efficiency for lower f- numbers. For example, the number of pulses required to fractionate 50% of the treatment zone increased from 14.5±9.5 pulses for an f-number of 0.51 to 209.3±17.4 pulses for an f-number of 0.89, corresponding to a >14-fold increase in treatment efficiency (Fig. 1C).

**CONCLUSIONS** The results of this study demonstrate that the histotripsy intrinsic threshold does not significantly change with f-number. In addition, results show that histotripsy fractionation efficiency decreases at higher f-numbers due to a decrease in the “bubble density” within the bubble cloud. Overall, this study provides significant insight into the effects of f-number on intrinsic threshold histotripsy that will help to guide the design of histotripsy transducers for specific clinical applications.

**Fig. 1 (abstract O72).**
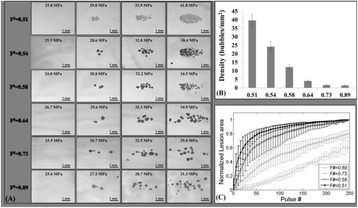
See text for description

### O73 Sonoporation-induced intracellular delivery and cytoskeleton disassembly with different microbubble-cell distances

#### Maochen Wang, Chenliang Cai, Pengfei Fan, Dongxin Yang, Zhiyang Jin, Juan Tu, Xiasheng Guo, Dong Zhang

##### School of Physics, Nanjing University, Nanjing, Jiangsu, China

###### **Correspondence:** Maochen Wang

**OBJECTIVES** Sonoporation mediated by ultrasound-driven microbubbles is being extensively studied as a promising technology to facilitate gene/drug delivery to cells, and microstreaming generated by pulsating microbubble near the cell membrane is regarded as one of the most important mechanisms in the sonoporation process. Thepresence of microbubbles is believed to help enhance ultrasound-induced bioeffects, but an in-depth understanding of cellular responses to sonoporation generated atdifferent microbubble-cell distances remained to be elucidated. The goal of this study was to investigate how the microbubble-cell distances influence the cell dynamics, such as the cytoskeleton arrangement and the cellular membrane permeability, and the experimental observations were compared with theoretical simulations.

**METHODS** The *in situ* cellular responses (e.g., cytoskeleton arrangement and intracellular delivery) to microbubble-mediated sonoporation process generated withdifferent microbubble-cell distances were systemically assessed based on an integrated system combining ultrasound exposure apparatus with real-time fluorescencemicroscope imaging. The microstreaming and shear stress generated by an oscillating microbubble was simulated based on an encapsulated microbubble dynamic modelwith considering nonlinear rheological effects of both shell elasticity and viscosity.

**RESULTS** The results show that: (1) as the microbubbles get closer to cells, the disassembly of the cytoskeleton will accelerate; (2) as the microbubbles get closer tocells, the permeability of cell membrane will have significant improvement; (3) the maximum microstreaming-induced shear stress on the cell membrane increasesrapidly with reducing the bubble-cell distance.

**CONCLUSIONS** In summary, by performing live cell fluorescent imaging over the process of sonoporation, the accelerating cytoskeleton disassembly and improvedmembrane permeabilization could result from microbubble-mediated sonoporation with reducing bubble-cell distance. The shear stresses resulting from microstreaminggenerated nearby pulsating bubbles with different bubble-cell distances were simulated theoretically, which could provide a better explanation for observed phenomena. The result suggests that in order to achieve more efficient sonoporation effect in therapeutic applications, it is better to find and optimal bubble-cell distance andmanipulate the microbubble location more rationally under certain conditions.

### O74 A pilot study of histotripsy using 1.1/2.2 MHz dual-frequency superimposition focused ultrasound pulses

#### Y. Li, R. Wang, M. Lu, W. Huang, F. Ma, L. Zhang, M. Wan

##### The Key Laboratory of Biomedical Information Engineering of Ministry of Education, Department of Biomedical Engineering, School of Life Science and Technology, Xi’an Jiaotong University, Xi’an, China

###### **Correspondence:** Y. Li

**OBJECTIVES** Kidney cancer is a severe disease which can be treated using a non-invasive, controllable and focused ultrasound surgery method, termed as histotripsy. However, the time of lesion formation following a single frequency histotripsy is long, so the dual-frequency mode histotripsy using second-harmonic superimposition hundred- microsecond pulses to reduce the lesion time is very necessary. The aim of this research is to explore the feasibility of enhancing histotripsy by increasing effective cavitation and efficient boiling.

**METHODS** By controlling the ratio of dual-frequency acoustic powers, the superimposition of two frequency pressures results in 9 split foci along beam axial within confocal region, and the maximal peak intensity of split focus can reach about 2 times the sum of two frequency intensities, indicating strong wave interference. Meanwhile, the cavitation threshold lowers and the nonlinear effect strengthens by the dual-frequency superimposition mode. The efficient boiling mechanism becomes dominant in histotripsy. The experiments implemented in polyacrylamide phantoms with bovine serum albumin and in porcine kidney *ex vivo*. The simplified two-stage hundred-microsecond pulsing scheme was used. The lesion formation process in the BSA phantom was monitored by high-speed photography and passive cavitation detection.

**RESULTS** The lesion inception time in gel-phantom was about 0.3s, which was 6 times shorter than that in single frequency mode (Fig. 1a). The enhanced pressure, the lowered cavitation threshold and the strengthened nonlinear effect by dual-frequency superimposition resulted in the lesion inception time decreased and boiling bubbles emerged frequently in the axial split foci during each lesion formation, indicating enhanced histotripsy (Fig. 1b). The interaction of boiling bubbles between split foci also had contributed to the lesion formation and the lesion size increased both radial and axial directions. The final lesion exhibited a long tear shape with smooth border (Fig. 1c). The lesion generated in *ex vivo* porcine kidney was shown in Fig. 2, and the voids appeared with no marked thermally coagulated component remaining after the homogenate had been removed. The root mean square (RMS) amplitude of the broadband noise from filtered passive cavitation detection (PCD) data revealed the strong inertial-cavitation activities, and the increase of RMS demonstrated that the boiling bubbles arose (Fig. 3a). Meanwhile, the inertial cavitation effect transferred to a higher band increment, which was beneficial to the frequency absorption efficiency (Fig. 3b).

**CONCLUSIONS** This study demonstrated the feasibility of enhancing histotripsy by increasing acoustic intensity, lowering cavitation threshold and strengthening nonlinear effect using dual-frequency superimposition focused ultrasound pulses. The increase of effective cavitation and boiling dominated in the lesion formation. The split foci occur by controlling the ratio of dual-frequency acoustic powers. The maximal peak intensity of split focus can reach about 2 times the sum of two frequency intensities, indicating strong wave interference. The lesion inception time decreased, compared with single frequency mode. The final lesion exhibited a long tear shape with smooth border. The root mean square amplitude of the filtered passive cavitation detection data revealed the strong inertial-cavitation activities, and the increase of RMS demonstrated the boiling bubbles arose.

**Fig. 1 (abstract O74).**
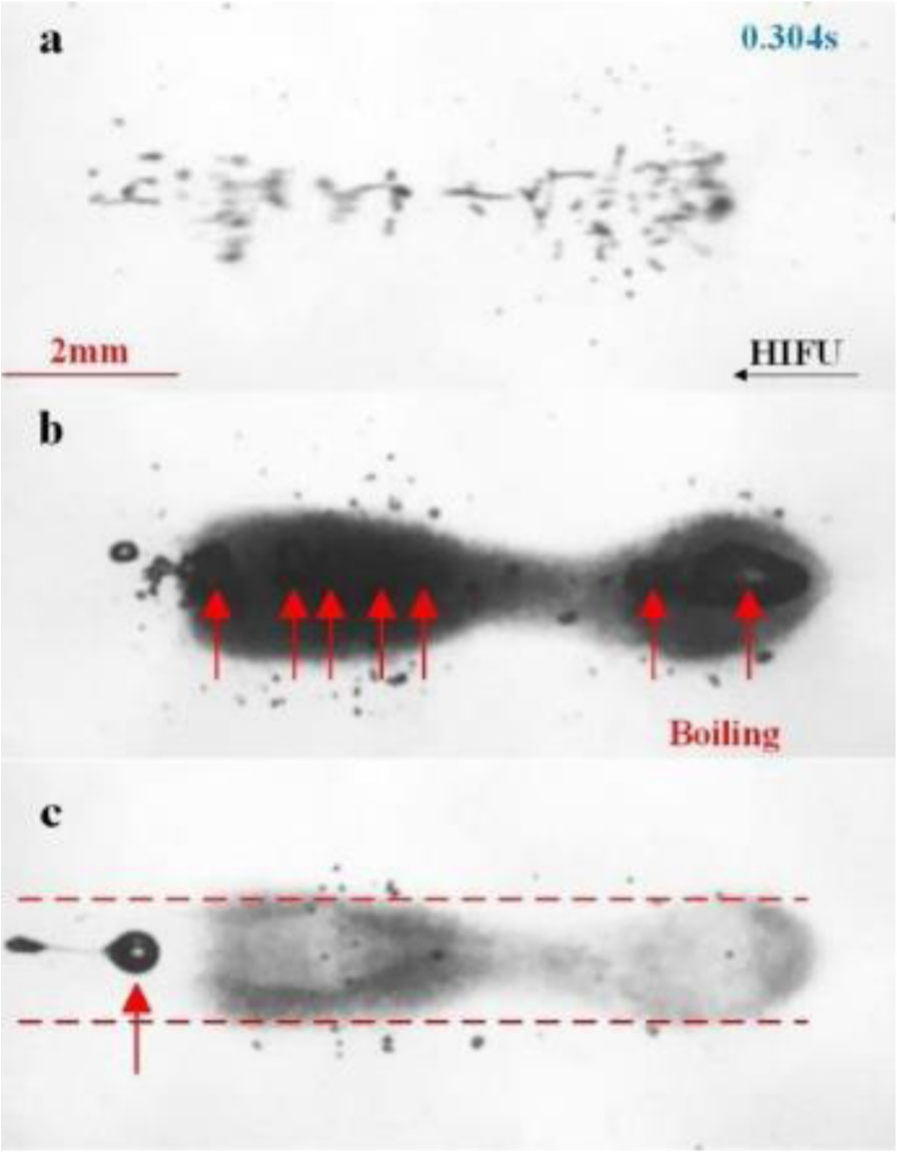
(a) The lesion inception time in gel-phantom was about 0.3s. (b) Boiling bubbles with about 1mm diameter in phantom acquired by high-speed photography. (c) The final lesion exhibited a long tear shape with dimensions of approximately 8.2×1.6 mm (axial×lateral)

**Fig. 2 (abstract O74).**
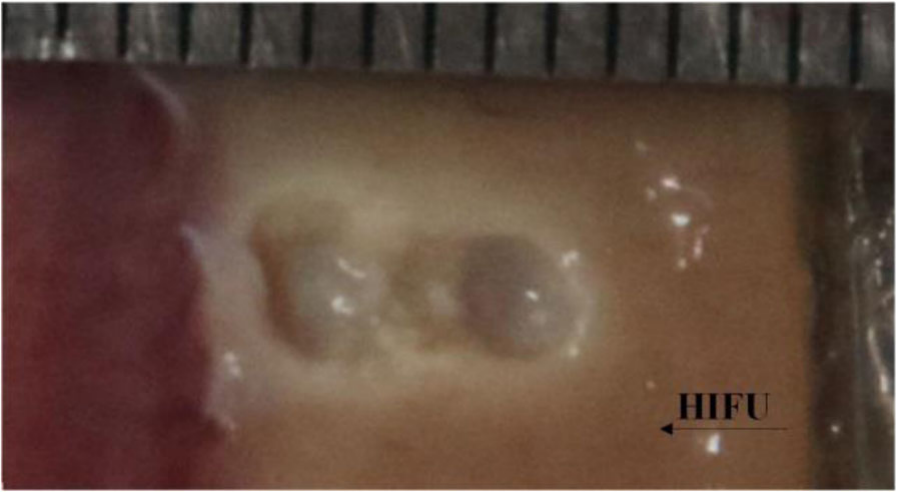
Representative gross morphology of the dual-frequency-superimposition histotripsy lesions induced in *ex vivo* porcine kidney with dimensions of approximately 6.0×2.8 mm (axial×lateral)

**Fig. 3 (abstract O74).**
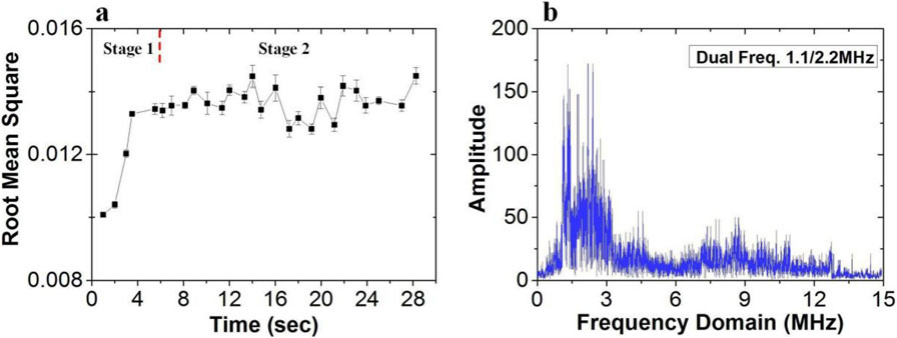
(a) RMS amplitude of the PCD signals using dual frequencies of 1.1/2.2 MHz on the phantom at different times. (b)Broadband noise in the frequency domain using dual frequencies of 1.1/2.2 MHz on the phantom

### O75 Ultrasound-guided high intensity focused ultrasound ablation for diffuse adenomyosis

#### J. Chen, Y. Feng, W. Chen

##### College of Biomedical Engineering, Chongqing Medical University, Chongqing, China

###### **Correspondence:** J. Chen

**This abstract is not included as it has already been published:**

Feng Y, Hu L, Chen W, Zhang R, Wang X, Chen J. Safety of ultrasound-guided high-intensity focused ultrasound ablation for diffuse adenomyosis: A retrospective cohort study. Ultrason Sonochem. 2017; 36: 139-145. Available from: http://www.sciencedirect.com/science/article/pii/S1350417716304096.

### O76 Preliminary results of synthetic aperture imaging using random phased array

#### M. Zubair, R. J. Dickinson

##### Bioengineering, Imperial College London, London, United Kingdom

###### **Correspondence:** M. Zubair

**OBJECTIVES** Randomized phased arrays have been used for generating and steering single focus and multiple foci with low levels of grating lobes due to the breakage of periodicity of the elements and are considered as useful source of HIFU. However, the reliance of HIFU on MRI for real time visualization of the targeted tissue is a major constraint in its clinical use due to the high cost of MRI and its low temporal resolution. There is a need to study the imaging capabilities of a therapeutic random phased array transducer for guiding the treatment process.

**METHODS** Dual mode ultrasound phased arrays would have the advantage of using the same array for both therapy and imaging due to the inherent registration between imaging and therapeutic frames of reference. The random spherical array would have limited field of view due to the fact that the array is optimized for therapy only and has large, directive elements sparsely positioned on a spherical surface. Nevertheless, images obtained will be useful for directing therapy as they will be perfectly aligned with the therapy transducer. Since strong scattering objects in path of HIFU beam are also in path of imaging beam, such scattering objects can be detected in real time and the HIFU beam can be adjusted accordingly. In our HIFU system the elements are randomly distributed with inter-element spacing much more than the required half a wavelength for reduced side lobes (Hand et. al. 2009), thus the spatial resolution of this system is poor. However, we use synthetic aperture imaging technique which has the potential to improve the spatial resolution of the random phased array. The simulations were carried out in MATLAB. The numerical results were performed for a 1 MHz 256-element random phased array, made by Acublate Ltd, London, UK. Simultaneous foci were generated in simulations as well as experimentally based on the theory described by Gavrilov and Hand (2000a) (Fig. 1).

**RESULTS** For imaging, preliminary simulations of synthetic aperture imaging with a 1MHz 256 element random phased array are shown. In Fig. 2, grey scale image of a wire target array is shown, which is a composite of point spread functions (psfs) at different on- and off-axis positions. The axial spacing between two wires is 10 mm, whereas the lateral distance is 5mm (figure 30. The -6 dB full width half maximum of the focused psf is 1.6 mm. Sub-apertures are being used to image the field of interest, where each sub-aperture contains only those elements which contribute to the pixel being imaged. This not only improves the resolution by decreasing the side lobes level but also reduces the computation time.

**CONCLUSIONS** It was observed that random phased arrays are capable of therapy as well as imaging for treatment guidance. The use of sub-apertures improves the resolution and reduce the computation time, however, it also limits the imaging field of view

**Fig. 1 (abstract O76).**
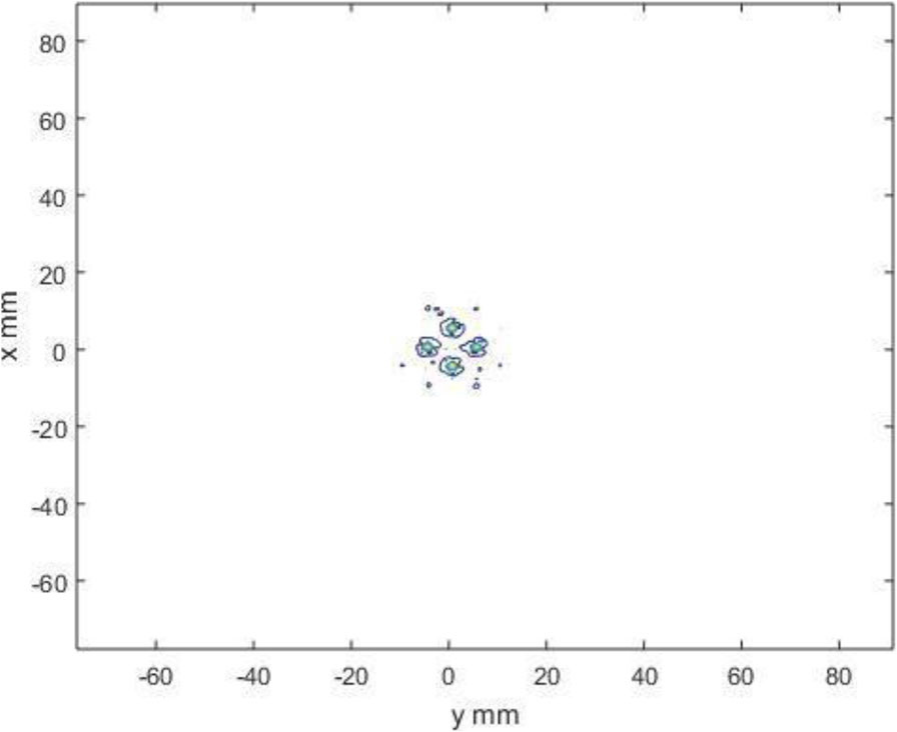
Distribution of 4 simultaneous foci at a depth of 130 mm

**Fig. 2 (abstract O76).**
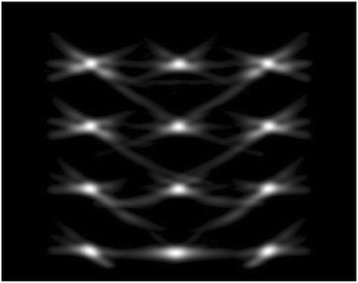
Grayscale images of wire target array using STA imaging approach

**Fig. 3 (abstract O76).**
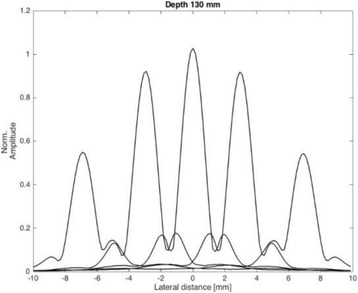
Lateral cross section at a depth 130 mm

### O77 Image-guided blood-brain barrier opening with focused ultrasound and microbubbles in mice using passive microbubble imaging

#### M. T. Burgess^1^, I. Apostolakis^1^, E. Konofagou^1,2^

##### ^1^Biomedical Engineering, Columbia University, New York, New York, USA; ^2^Radiology, Columbia University, New York, New York, USA

###### **Correspondence:** M. T. Burgess

**OBJECTIVES** Blood-brain barrier (BBB) opening with focused ultrasound (FUS) and microbubbles is a technique for targeted drug delivery to the brain and has led to the development of ultrasound-guided focused ultrasound (USgFUS) systems for appropriate treatment monitoring and guidance. Comprehensive detection of FUS-stimulated microbubble activity is critical for successful implementation of these systems. Current techniques passively image microbubble- related acoustic emissions with ultrasound arrays to spatially map the magnitude and location of microbubble activity throughout the focal volume of the FUS. In turn, this provides information about the extent and location of BBB opening. Current passive cavitation imaging methods with linear arrays suffer from poor axial image resolution due to the use of long FUS pulses and asynchronous transmit and receive sequences that degrades absolute time-of-flight information. The objective of this study was to preserve absolute time-of-flight information by using short pulses of FUS, along with synchronous transmit and receive acquisition, for utilization of image reconstruction similar to pulse- echo B-mode imaging. It is expected that this new passive microbubble imaging (PMI) technique will improve image resolution and offer a methodology capable of high resolution mapping of BBB opening.

**METHODS** PMI was carried out during BBB opening with FUS and microbubbles in a mouse model. An 18-MHz imaging array (L22-14v LF, Verasonics, Inc.) and 1-MHz FUS transducer were submerged in a tank of degassed water and placed approximately 2 cm above the mouse skull for transcranial application of FUS. The FUS transducer was aligned at an acute angle relative to the imaging array’s axis. A research-based ultrasound system (Vantage 256, Verasonics, Inc.) was used to operate the imaging array in a passive mode and synchronize the FUS transmission with receive acquisition. Short pulses of FUS (2-3 cycles, 200-400 kPa) at a pulse rate of 500-5000 Hz were used to insonify intravenously injected microbubbles as they flowed through the mouse brain microvasculature. In this scenario, the FUS pulses were used for the dual purpose of imaging and therapy (BBB opening). Receive acquisition frames were recorded for each FUS transmit and the data was saved for off-line processing. PMI images were formed using a custom GPU-based image reconstruction algorithm in MATLAB (The Mathworks, Inc.). The time delays for implementation of delay-and-sum beamforming were calculated to account for the propagation from the FUS transducer, to the mouse brain, and back to the imaging array. Blocks of PMI frames were further processed using spatiotemporal filtering to isolate microbubble emissions. PMI was compared with post-FUS dynamic contrast enhanced-magnetic resonance imaging (DCE-MRI) to correlate microbubble activity with BBB opening.

**RESULTS** PMI overcomes the poor axial resolution concerns of previous passive imaging methods and provides detailed maps of microbubble activity throughout the focal volume of the FUS transducer during the BBB opening treatment. PMI is able to provide the detailed structure of the brain microvasculature in manner similar to power Doppler imaging, although only within the focal volume of the FUS. Simplistically, PMI reveals the heterogeneous distribution of microbubble activity within focal area that can be used to predict where BBB opening is occurring. Indeed, post-FUS DCE-MRI images correlated with the distribution of microbubble activity by showing contrast leakage into the brain in areas of microbubble activity. In addition to the detailed microbubble maps, PMI provides important temporal information about the microbubble infusion kinetics and persistence within the focal volume of the FUS.

**CONCLUSIONS** PMI can address a key technological limitation of poor axial image resolution with current passive cavitation imaging techniques and provide a method capable of monitoring BBB opening with FUS and microbubbles. Limitations of this new technique will be discussed along with its similarities and differences with existing methodologies. This new USgFUS modality is not only a promising technique to be used for treatment of CNS diseases, but also for oncological, cardiovascular, and other medical conditions that utilize FUS in combination with microbubbles.

### O78 Long-circulating behaviour and acoustic characterization of lipid-coated microbubbles

#### Y. Yang, H. Li, X. Guo, D. Zhang, J. Tu

##### Nanjing University, Nanjing, Jiangsu, China

###### **Correspondence:** Y. Yang

**OBJECTIVES** Lipid-coated contrast agents have attracted much attention for contrast ultrasound molecular imaging as well as targeted treatment, and the connection between enhanced contrast capability and acoustic properties of the lipid-coated ultrasound contrast agents need to be elucidated.

**METHODS** In this study, microbubbles were fabricated using thin-film hydration and mechanical agitation. Then, the morphology and distribution of these microbubbles were investigated through transmission electron microscopy (TEM) imaging and dynamic light scattering (DLS) sizing technology. To demonstrate physical properties of microbubbles, inertial cavitation threshold and acoustic attenuation measurements were carefully assessed and shell parameters were further estimated. The imaging function of synthesized microbubbles was also compared with that of SonoVue microbubbles *in vivo*.

**RESULTS** The results showed that the synthesized microbubbles had a spherical shape, a smooth, consistent membrane and uniform distribution, with average diameter of 1.484 μm. Imaging studies showed that while exhibiting comparable imaging ability, synthesized microbubbles had a longer circulation time and better stability than SonoVue. Physical characterization showed that compared with SonoVue, synthesized microbubbles with smaller interfacial tension and dilatational viscosity, indicating less attenuation during the propagation of sound wave. In comparison with SonoVue, IC threshold of the microbubbles are prominently higher in both concentration ranges, explaining better stability of the microbubbles. Present study built a bridge between physical properties and *in vivo* imaging performance of synthesized microbubbles, which could provide guidance to the design and safe application of ultrasound contrast agents.

**CONCLUSIONS** While the average grey scale of various organs all increased following microbubbles application, tumor imaging showed that synthesized microbubbles stayed in the tumor area for longer period of time and has a longer enhancing time. The long-circulating behavior showed that synthesized microbubbles may be better suited for tumor imaging and therapeutic application in drug/gene delivery. Furthermore, *in-vivo* and tumor imaging performance of synthesized bubbles was well explained by acoustic property measurements and shell elastic and viscous parameters. Possible correlation between physical/acoustical properties and *in-vivo*/tumor imaging performance was revealed in this study, and could be of help in future design and practical application of ultrasound contrast agents.

### O79 Pulse inversion based broadband subharmonic cavitation imaging for monitoring high intensity focused ultrasound therapy

#### H. Zhong, X. Ma, M. Wan

##### Biomedical Engineering, Xi'an Jiao Tong University, Xi'an, China

###### **Correspondence:** H. Zhong

**OBJECTIVES** This paper proposed a cavitation imaging method by extracting broadband subharmonic based on pulse inversion technique during high intensity focused ultrasound (HIFU) therapy to obtain monitoring images with high cavitation-to-tissue ratio (CTR), high cavitation detection sensitivity and high resolution.

**METHODS** HIFU delivery is briefly interrupted with the HIFU completely off for transmission of a pair of inverted pulses (4.6 MHz) and acquisition of the backscattered signals from tissue samples during HIFU treatment. After summing the echoes of positive and negative pulses, the subharmonic filters are designed with the center frequency of 2.3 MHz and the bandwidth of 20%, 60%, 80%, 100% and 140% to obtain the cavitation images. For comparison, the second harmonic images were also obtained. A threshold is used to assess the cavitation detection sensitivity of different cavitation images. Cavitation bubble areas could be calculated by counting the number of pixels whose values are greater than the threshold. The larger is the cavitation bubble area, the higher is the cavitation detection sensitivity. Cavitation-to-tissue ratio (CTR) could be defined as the ratio of the intensity value averaged in the regions of interest (ROI) of HIFU treated area to that in the ROI of HIFU untreated area. We used the normalized −6 dB width of the autocorrelation function of the enveloped RF signal to quantify the resolution of cavitation images.

**RESULTS** The experiments with porcine muscle demonstrated that the cavitation images obtained by using 80%~100% bandwidth subharmonic filters have the greatest cavitation detection sensitivity. The cavitation bubble areas of broadband subharmonic images (1.15~3.45 MHz, including 1/2, 1/3, 1/4 subharmonic components) are 1.6~2.7 times of those of narrowband subharmonic images (2.07~2.53 MHz, including 1/2 subharmonic component) with the suitable threshold. The difference of the CTR values between the broadband and narrowband subharmonic images is not very large, but the CTR values of both broadband and narrowband subharmonic images are much greater than those of second harmonic images. It was found that the resolution of broadband subharmonic images was improved up to about 2 times compared with narrowband subharmonic images.

**CONCLUSIONS** The proposed broadband subharmonic cavitation imaging method could obtain much higher CTR value than second harmonic imaging method. And the method has higher cavitation detection sensitivity and resolution than normal narrowband subharmonic imaging method.

### O80 A generalized theoretical framework for understanding ultrasonic neuromodulation mechanisms

#### M. Plaksin, S. Shoham, E. Kimmel

##### Faculty of Biomedical Engineering & Russell Berrie Nanotechnology Institute, Technion – Israel Institute of Technology, Haifa, Israel

###### **Correspondence:** M. Plaksin

**OBJECTIVES** Low intensity US can noninvasively suppress or excite central nervous system (CNS) activity. Recently, we introduced the Neuronal Intramembrane Cavitation Excitation (NICE) framework to explain the observed aspects of ultrasonic neuromodulation, through intramembrane space-related capacitance variations leading to cell- type-selective activation effects. Here we expanded the framework to dissect also the impact of acoustic radiation pressure (ARP) - induced membrane capacitance changes on neuronal activity, as well as to explore the effect of cell environment effective viscosity on NICE model-related neural response.

**METHODS** We analyzed the relevant experimental literature using two sources of ARP gradients responsible for membrane dynamics: 1) ARP caused by ultrasonic field inhomogeneity and 2) ARP caused by acoustic impedance mismatch. In addition, live brain tissue ARP-gradients-subjected areal strains were evaluated in a viscoelastic brain model. In the context of the NICE model dynamics, the modified Rayleigh–Plesset-based intramembrane cavitation biomechanics was calculated with cell environment viscosity higher than water viscosity (water viscosity was used in our previous studies), to express the exact biological properties of cells. By coupling these biomechanical models to biophysical membrane models we predict dynamical biophysical responses of artificial bilayer membranes, and of common neocortical single cell Hodgkin-Huxley type models.

**RESULTS** The augmented viscosity conditions in the NICE model lead to parabolic relationship between US frequency and threshold intensities for cortical pyramidal neuron stimulation, wherein below 1 MHz the dependence on US frequency is negligible (with complete agreement to our previous studies). The new results were found to explain and predict the experimental results of Ye et al., Ultrasound Med Biol. 2016 that also captured the same behavior in their mouse study. The emergence of ARP gradients-induced membrane capacitance variations associated with membrane area changes fully explain artificial membrane experimental results and may also be responsible for neural excitation at in- vitro experimental conditions. However, these capacitance changes were found to be highly unlikely sources for neural excitation at *in-vivo* experimental conditions, when considering the areal strains expected to form in brain tissue during normal sonication.

**CONCLUSIONS** These results provide more complete framework for a large body of experiments in multiple preparations across the field of US neuromodulation, lending further support to the hypothesis that intramembrane cavitation is responsible for ultrasonic neuromodulation. They could thus pave the way towards new CNS therapeutic protocols, using the only method that currently allows targeted noninvasive neuromodulation with millimeter spatial resolution essentially anywhere in the brain.

### O81 Non-invasive high frequency transcranial focusing with a single element transducer: experimental validation of adaptative focusing through human skulls with a 3D printed acoustic lens

#### G. Maimbourg^2,1^, A. Houdouin^2^, T. Deffieux^2^, M. Tanter^2^, J. Aubry^2^

##### ^1^Université Paris Diderot, Paris, France; ^2^Institut Langevin, ESPCI Paris, CNRS UMR7587, INSERM U 979, Paris, France

###### **Correspondence:** G. Maimbourg

**OBJECTIVES** Transcranial ultrasonic brain therapy requires a wave front shaping device to compensate skull- induced aberrations. Up to now, this was done with a multi-elements probe: the phase of the signal emitted by each individual transducer is adjusted in order to compensate for the delay caused by the crossing of the skull. Historically, a growing number of elements was used (64 elements in 2000 [1], 300 in 2003 [2], 1024 in 2012 [3]) to improve the focusing. We propose to radically change the paradigm by achieving adaptive transcranial focusing with a single- element covered with a 3D silicone acoustic lens of variable and controlled thickness [4]. Similar lenses have been introduced in the past to perform single or multiple focusing patterns in homogenous propagating media [4,5] but recent 3d printing and milling capabilities make tailor-made 3D lenses a feasible option for transcranial adaptive focusing. The characteristic length of variation in thickness for the acoustic lens is less than 1mm. A 6cm radius lens- corrected single-element is thus equivalent to an 11,000 element transducer in terms of phase shaping capabilities.

**METHODS** The lens is made of silicone (Elite double 8, Zhermack Spa, Italy). The speed of sound is c_silicone_=1000m/s in silicone and c_water_=1485m/s in water. This difference can be used to modify the wave phase by controlling the local thickness of the lens, and thus correct skull-induced aberrations [6]. The study was conducted on three human skulls. The skulls were harvested and cleaned at the Saints-Pères Anatomy Institute (Paris Descartes University) for transcranial ultrasound focusing studies. A 3D simulation based on computed-tomography of the skulls was then performed to estimate the phase shift induced by the skull at surface of the transducer (single element, 59 mm radius of curvature, f-number of 1, operated at 914 kHz). The thickness of the lens was then adjusted to compensate the shifts by casting the silicone in a 3D-printed mold. The acoustic-lens-covered transducer was then immersed in water (Fig. 1). Lastly, the skull was positioned in front of the transducer with a 3D printed holder. The quality of the focusing through the skull was then assessed using a 3D scan of the pressure field with a needle hydrophone (HNA-0400, Onda Corp., Sunnyvale, CA, USA).

**RESULTS** Figure 2 shows the acoustic pressure obtained experimentally with skull A. Axial and transverse views are presented in the absence of skull (left), through the skull without correction (center) and with the acoustic lens (right). The lens qualitatively restores the focusing. Table 1 reports the quantitative outcomes for the three skulls noted A, B and C. The lens increased the maximum acoustic intensity by 97 ± 56 %. Compared to the reference in water with no skull in place, the mean -3dB volume of the focus increased by 472 ± 231 % when crossing the skull, whereas it only increased by 86 ± 29 % with the lens- based correction.

**CONCLUSIONS** We demonstrated that in 3D printing can be used to create custom-made acoustic lenses for CT- based non invasive skull-aberrations correction, making it possible to compensate for skull aberrations without a multi- element device. Due to its simplicity, the acoustic lens is expected to be a cheaper and less cumbersome solution than current multi-element probes. The ultrasonic absorption of the lens material remains to be optimized for thermal treatments, but the setup presented here could be of interest for low energy treatments such as neuromodulation or blood brain barrier opening.

**Acknowledgements**

This work was supported by the Bettencourt Schueller Foundation and the "Agence Nationale de la Recherche" under the program “Future Investments” with the reference ANR-10-EQPX-15.

**References**

[1] Clement G et al, A hemisphere array for non-invasive ultrasound brain therapy and surgery. Phys Med Biol, 2000 [2] Pernot M et al., High power transcranial beam steering for ultrasonic brain therapy. Phys Med Biol, 2003

[3] Jeanmonod D et al, Transcranial magnetic resonance imaging-guided focused ultrasound: noninvasive central lateral thalamotomy for chronic neuropathic pain. Neurosurg Focus, 2012

[4] Fjield T et al, Low-profile lenses for ultrasound surgery. Phys Med Biol, 1999

[5] Melde K et al, Holograms for acoustics. Nature, 2016

[6] Patent: FR 1556217, July 2015

**Fig. 1 (abstract O81).**
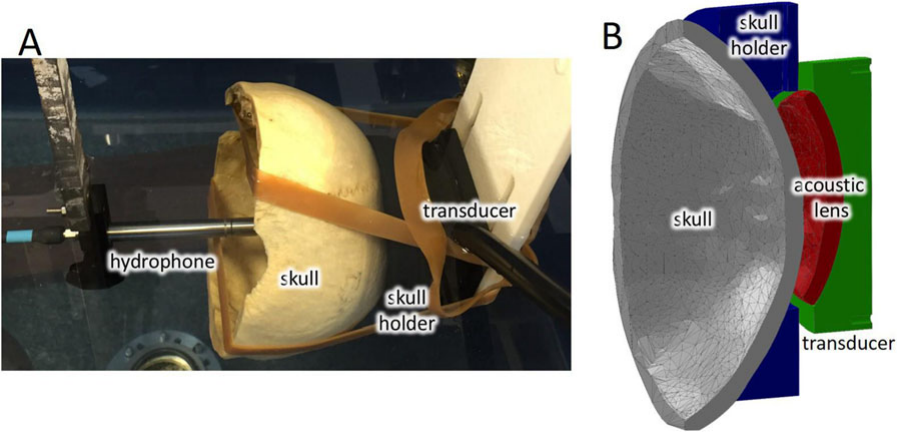
(A) Experimental setup in the water tank during the 3D-scan of the pressure field by the needle hydrophone. The skull is supported on the internally-designed holder by two elastic bands. (B) an axial cut of the setup. The acoustic lens covers the surface of the transducer

**Fig. 2 (abstract O81).**
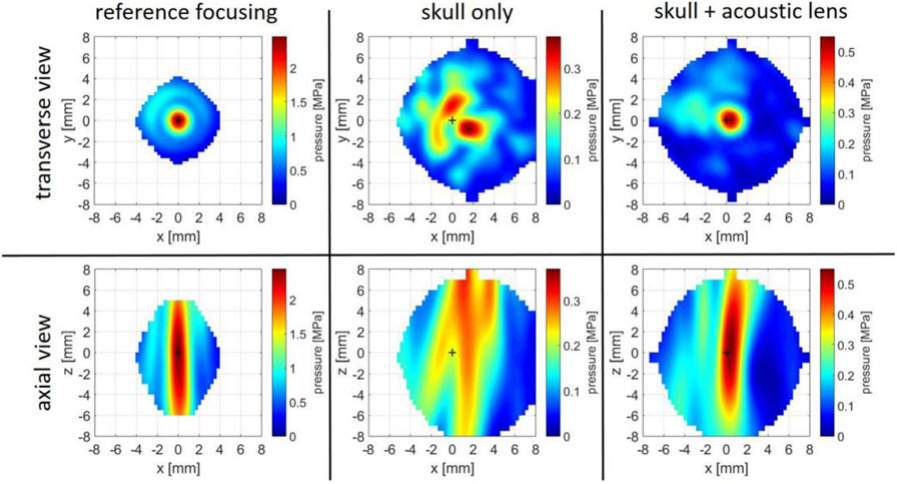
Acoustic pressure acquired by a needle hydrophone. From left to right: the reference focusing (no skull and no lens), the focusing through a human skull without any correction and the focusing through a human skull with the custom-made acoustic lens. The black crosses depict the position of the reference focusing

**Table 1 (abstract O81).**
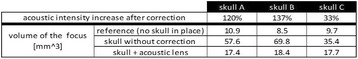
Quantitative results for assesing the efficiency of the acoustic lens. Outcomes were obtained for three skulls named A, B and C

### O82 Correlation of the lesion size in histology and mr images of the pig brain tissue by transcranial MR-guided focused ultrasound

#### D. Paeng^1,2^, Z. Xu^3^, J. Snell^4^, A. H. Quigg^5^, M. Eames^4^, C. Jin^1,4^, A. C. Everstine^3^, J. P. Sheehan^3^, B. S. Lopes^6^, N. Kassell^4^

##### ^1^Ocean System Engineering, Jeju National University, Jeju, Jeju, Korea; ^2^Radiation Oncology, University of Virginia, Charlottesville, Virginia, USA; ^3^Neurosurgery, University of Virginia, Charlottesville, Virginia, USA; ^4^Focused Ultrasound Foundation, Charlottesville, Virginia, USA; ^5^Medical School, Virginia Common University, Richmond, Virginia, USA; ^6^Pathology, University of Virginia, Charlottesville, Virginia, USA

###### **Correspondence:** D. Paeng

**OBJECTIVES** This study is to investigate the correlation of the lesion size in histology and MR images of the pig brain tissue, which was formed by a transcranial magnetic resonance-guided focused ultrasound (tMRgFUS) system and observed in 3 days after sonication. The lesion was generated by relatively low temperature between 46 ~ 52°C for an appropriate time to reach a target thermal dose in CEM (cumulated equivalent minutes). The target thermal dose was below 200 CEM which was obtained by variation of pulse duration of the tMRgFUS system for constant target temperature through a closed-loop system based on MR thermometry.

**METHODS** A tMRgFUS system (ExAblate 4000 Neuro 650 kHz system, InSightec, Israel) was used for this pig experiment. An MRI system (Discovery MR75-3.0T, GE Medical systems) was used for thermometry and pre- and post-imaging. A closed-loop control system was implemented on a personal computer to control pulse duration of the tMRgFUS system at a certain acoustic power in order to maintain a target temperature based on the MR thermometry. Temperature distribution and accumulated thermal dose in the target brain tissue was calculated every 3.7 seconds. Sonication was stopped when a prescribed thermal dose was delivered to the targeted tissue. The proportionate and integral coefficients of the PI controller were found to be 5 and 1.5, respectively, from several phantom experiments and first acute pig experiment while the acoustic power was varied up to a few hundred watts. Twelve chronic pig experiments with craniectomy were conducted, and post MR images within 1 hour and at 3 days were taken after sonication. Brain tissue was harvested in 3 days before euthanasia for histology. Four lesions were generated on both sides of the thalamus of each pig. Temperature in the pig brain tissue was estimated by rectal temperature for the MR thermometry baseline. This study was approved by the University of Virginia Institutional Animal Care and Use Committee (ACUC).

**RESULTS** Among 12 chronic pigs, one pig had a problem with introduction of air bubbles during surgery procedure and another one had an ACUC issue, so that these data could not be used. Three other pigs had a lower rectal temperature below 32°C so that the thermal dose computation was not reliable, leading to exclusion of the data analysis. No obvious lesions were observed in MR images taken in an hour of sonication for thermal dose less than 200 CEM. Figure 1 shows the lesion diameters measured in MR T2-weighted axial images and histology in 3 days of sonication as a function of thermal dose in CEM. There are lesions in all MR images and histology over 77 CEM except 101 CEM where no lesion is shown in histology while MR shows a lesion. The lesion diameters from MR images and histology are mostly 3 ± 1mm for thermal dose over 77 CEM. There are 6 lesions in MR T2-weighted axial image but no lesion was found in histology, whose thermal doses were 23, 30, 46, 53, 69, 101 CEM. Below 75 CEM, no lesion was found except one in 18 CEM in histology. One lesion on 18 CEM is shown in both MR image and histology, and the rectal temperature was low to 33.3°C. Lesion diameter observed in histology (y) is highly correlated with one in MR T2-weighted axial imaging (x), and their function and correlation coefficient are y=0.90x and r^2^=0.66, respectively. Even though the histology is sliced in coronal plane, the histology lesion diameter is highly correlated with MR axial diameter.

**CONCLUSIONS** The lesions were formed in the pig brain tissue in 3 days of sonication by tMRgFUS for thermal dose over 77 CEM, except one in histology for thermal dose of 101 CEM. The diameter of the lesions was measured to be mostly 3 ± 1mm in both MR T2-weighted axial images and histology, which is close to focal size. The diameter in histology is 10 % smaller than the one in MR T2-weighted axial images with correlation coefficient of 0.66. There are some differences in lesions between histology and MR images, and further systematic researches are required for the reasons.

**Fig. 1 (abstract O82).**
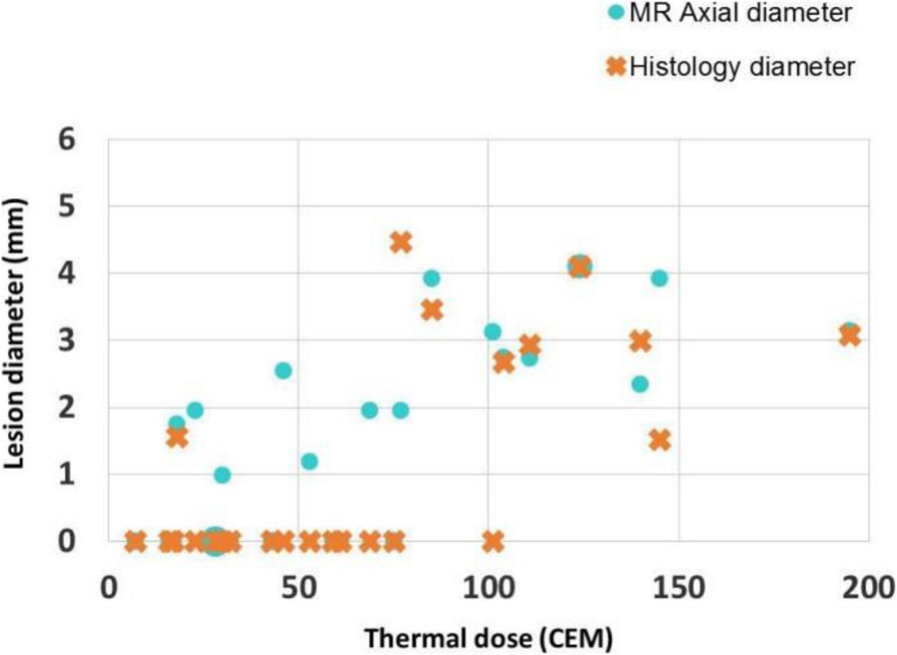
Comparison of the diameter of MR T2-weighted axial image with the one of histology as a function of thermal dose in CEM

### O83 Real-time image-based transcranial refocusing of dual-mode ultrasound arrays: *ex vivo* results

#### H. Aldiabat, P. D. O'Brien, D. Liu, E. S. Ebbini

##### Electrical Engineering, University of Minnesota, Minneapolis, Minnesota, USA

###### **Correspondence:** H. Aldiabat

**OBJECTIVES** Transcranial focused ultrasound (tFUS) is gaining wider acceptance in a range of therapeutic applications for the treatment of brain disorders. A major challenge towards widespread use of tFUS-based therapies stems from the complexity of the skull that could result in severe loss of focusing gain. Using extensive transskull hydrophone scans in rodent and human skulls, we have documented a range of tFUS beam distortions from mild shifting to complete splitting. These distortions could compromise the specificity of targeting brain circuitry and/or cause collateral damage at unintended locations. In this paper, we present first *ex vivo* results demonstrating the feasibility of image-based refocusing to regain the focusing gain.

**METHODS** A 3.5-MHz dual-mode ultrasound array (DMUA) prototype (64 elements, concave with 40-mm radius of curvature) was used. The DMUA was used to image and generate therapeutic focused ultrasound beams through rodent skull samples *ex vivo*. In some experiments, the target was the nose of a cone positioned near the geometric focus of the DMUA behind the skull sample (Fig. 1). In other experiments, skull samples were embedded in a tissue- mimicking phantom and positioned at a distance of approximately 32-mm from the apex of the DMUA (corresponding to the skull position during *in vivo* experiments.) A thermocouple was inserted so that its junction was closest to the geometric focus. Two modes of imaging were used: 1) Synthetic-aperture imaging, which provided larger field of view to guide the placement of the tFUS beam, and 2) Single-transmit focus (STF) imaging, which allowed for the characterization of the tFUS beam interaction with the skull. SA images were used to place target point(s) and critical point(s) used by the refocusing algorithm. Raw echo data received by each array element were used to form data matrices corresponding to the target and critical point(s). These matrices were used to solve an optimal refocusing problem based on the concept of propagation operators from the array elements to the target point(s). The refocusing algorithm ran automatically once the user specified the target and critical point. It was implemented on a software- defined ultrasound (SDUS) architecture that allowed the real-time computation of the refocused excitation vector and immediate download to the driver within milliseconds. To demonstrate the improvement in focusing gain, we have analyzed the change in echogencity from the target when insonified using geometric and refocused STF imaging beams. In addition, the temperature rise due to therapeutic tFUS beams with geometric focusing and optimal refocusing were directly measured. The heating rates were computed by taking the time derivative of the measured temperature profiles.

**RESULTS** Figure 1 also shows STF images generated from uniform element excitation (geometric focusing) and refocused excitation vector. The refocused STF image exhibits increased echogenicity from the target (thermocouple) and reduced echogenicity from the skull, where a critical point was located near the medial suture line. This is more clearly shown by the line graphs in Figs. 2 and 3. To demonstrate the increased therapeutic gain due to refocusing, the geometric focusing beam was used in therapeutic mode to produce heating at the thermocouple for 10 second duration followed by a refocused beam. Figure 4 shows the heating rates produced by the geometric focus and the refocused beam as estimated from the thermocouple measurements. It is quite clear that the refocusing gain was increased approximately by a factor of 3. Specifically, the heating rate due to the refocused beam was 3.636 C/sec while that of the geometrically focused beam was 1.179 C/sec. This result was typical, but variation in the refocusing gain were observed depending on which part of the skull was traversed by the tFUS beam.

**CONCLUSIONS** The results shown above demonstrate the feasibility of using STF imaging data for characterizing the quality of the tFUS beams noninvasively. More importantly, they demonstrate the feasibility of refocusing the beam to provide significant improvement in focusing gain as evidenced by the 3-fold increase in heating rate shown. The result shown here was typical of numerous experiments where the tFUS beam traversed different regions within the skull with different distortion. In every case, however, a significant improvement in focusing gain was achieved. The results shows were obtained using a 1D array and the refocusing was achieved only in the axial lateral directions. A 2D array would have produced even higher refocusing gain by taking advantage of the elevation direction.

**Fig. 1 (abstract O83).**
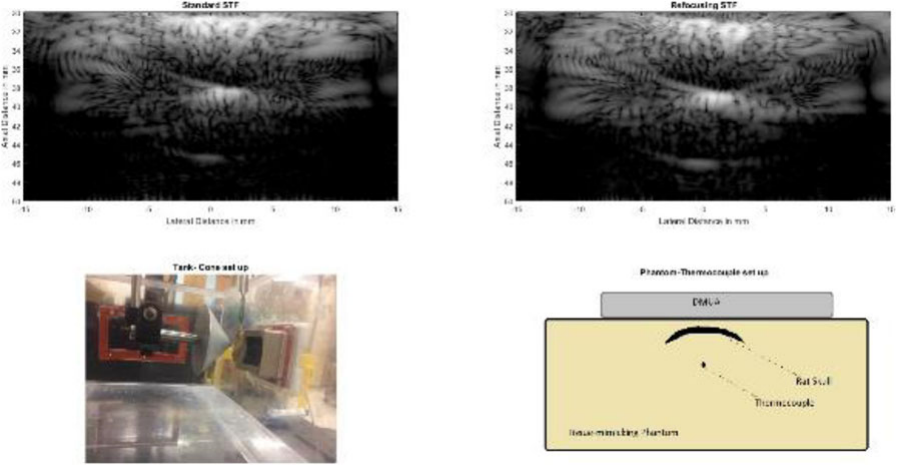
See text for description

**Fig. 2 (abstract O83).**
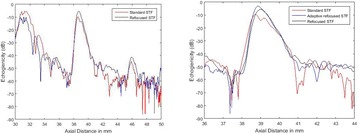
See text for description

**Fig. 3 (abstract O83).**
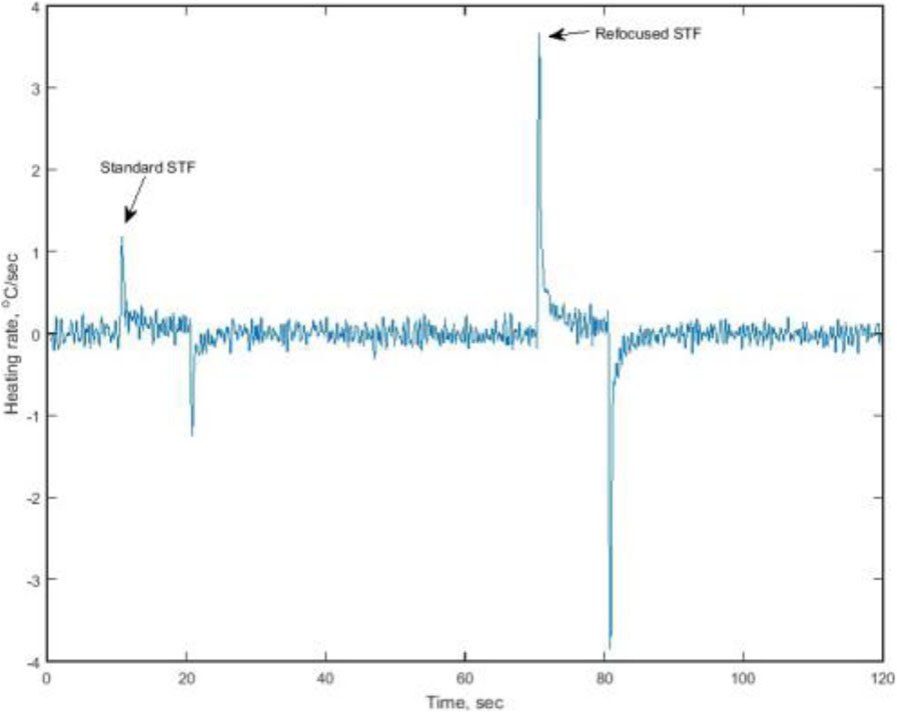
See text for description

### O84 A comparative study of transcranial histotripsy applied with and without aberration correction

#### J. R. Sukovich, T. Gerhardson, T. Hall, J. Macoskey, C. A. Cain, Z. Xu

##### Biomedical Engineering, University of Michigan, Ann Arbor, Michigan, USA

###### **Correspondence:** J. R. Sukovich

**OBJECTIVES** Histotripsy is an ultrasound therapy that generates cavitation bubble clouds to fractionate soft tissue using short duration, high-amplitude ultrasound pulses. Previous studies have shown that histotripsy is capable of producing lesions through the skullcap with or without using aberration correction. Transcranial ultrasound thermal therapy cannot treat within 2 cm of the skullcap and cannot treat large volumes in the brain due to skull heating, which makes it unsuitable for brain tumor treatments. In comparison, histotripsy can be used to treat targets near the skullcap as well as large volume targets transcranially with minimal skull heating by using low duty cycle pulses (<0.1%). This study presents comparative analyses of the pressure fields, cavitation clouds and lesions, and treatment rates of histotripsy applied transcranially with and without aberration correction.

**METHODS** Histotripsy pulses were delivered through *ex-vivo* human skullcaps mounted centrally within a 500 kHz, 256-element histotripsy transducer with transmit-receive capable elements. Aberration correction was applied in two ways, 1) based on hydrophone measurements and 2) based on histotripsy pulse-backscatter measurements (from bubbles generated transcranially without aberration correction) acquired using the array elements as receivers. Following aberration correction, hydrophone measurements of the focal pressure amplitudes and focal beam profiles were acquired, as were measurements of the focal pressure amplitudes as a function of the electronic steering distance through the skull. Lesioning experiments were carried out in red blood cell tissue phantoms to compare how the different aberration correction modalities affected lesioning. Finally, volumetric treatments of large clots through the skull were conducted and treatment efficacy for each aberration correction case was evaluated as a function of clot volumes liquefied.

**RESULTS** Aberration correction performed based on hydrophone measurements and backscatter methods increased the peak negative pressure through the skull at the focus by 90% and 60%, respectively, compared to the no aberration correction case. The electronic steering radius at which focal pressures above the nucleation threshold could be reached improved by more than 100% with aberration correction, increasing from 7 mm without aberration correction to over 15 mm with aberration correction. The cavitation clouds and lesions generated in both aberration correction cases were equal in size and up to 30% larger than the no aberration correction case using the same input power to the ultrasound array due to increased focal pressure amplitudes. The increased pressure amplitudes and steering ranges in the aberration correction cases resulted in significantly improved transcranial clot liquefaction speeds compared to the no aberration correction case.

**CONCLUSIONS** This study indicates that all measures of histotripsy applied transcranially benefit from aberration correction. While hydrophone aberration correction was seen to provide the greatest increases in focal pressure, steering range, and liquefaction rates, aberration correction applied based on backscatter methods was seen to perform comparably to the hydrophone case and provide significant improvements over the no aberration correction case. These results indicate that backscatter aberration correction can offer a non-invasive method of improving the efficacy of transcranial histotripsy comparable to hydrophone based correction methods.

### O85 accumulated thermal dose in guiding essential tremor treatment: repeated sonications with peak temperatures below 55°C

#### Y. Huang^1^, R. M. Jones^1,2^, N. Lipsman^3^, M. L. Schwartz^3^, K. Hynynen^1,2^

##### ^1^Sunnybrook Research Institute, Toronto, Ontario, Canada; ^2^Department of Medical Biophysics, University of Toronto, Toronto, Ontario, Canada; ^3^Division of Neurosurgery, Sunnybrook Health Sciences Centre, Toronto, Ontario, Canada

###### **Correspondence:** J. R. Sukovich

**OBJECTIVES** Magnetic resonance guided focused ultrasound has recently been approved by the FDA for the treatment of essential tremor. During these treatments, peak temperatures between 55 and 60°C are generally desired to produce reliable lesions at the target in the thalamus. However, in some patients a peak temperature of 55°C cannot not be reached, either because of their skull properties or due to a low tolerance to the pain associated with substantial skull heating. In these patients, repeated sonications with peak temperatures from 50 to 54°C were typically achievable. The accumulated effects of these sonications may also result in a sufficient thermal dose to produce a lesion. Therefore, establishing a proper threshold for the accumulated thermal dose (ATD) is important for guiding these repeated sonications with lower peak temperatures. In this study, clinical treatments at our institution with maximum peak temperatures below 55°C were retrospectively reviewed. ATD maps were calculated offline with corrections for chemical-shift artifacts and were correlated to the lesion sizes observed at follow-up imaging to find the best estimate for the ATD threshold in terms of cumulative equivalent minutes at 43°C (CEM_43_).

**METHODS** From Jul 2015 to Jan 2017, 28 patients with medication-refractory essential tremor have been treated with a clinical focused ultrasound system (ExAblate Neuro, 650 kHz central frequency, InSightec, Tirat Carmel, Israel) and a 3 T MR scanner (MR750, GE Healthcare, Milwaukee, WI, USA). Among these, 5 patients were treated with a maximum peak temperature below 55°C (53 or 54°C). For the ATD calculation over multiple sonications, chemical-shift artifacts in MR thermometry were corrected retrospectively in Matlab if the misalignment between successive sonications exceeded 1 mm by manually shifting the centre of the heating area along the frequency-encoding direction. The ATD was then integrated in the axial plane using the standard thermal dose model. The spatial dimensions of the ATD at 17, 40 and 240 CEM_43_ were measured and correlated to the lesion sizes measured in T1- (3D FSPGR, TR 8.3 ms, TE 3.3 ms, slice thickness 1.2 mm) and T2- (FRFSE, TR 5200 ms, TE 100 ms, slice thickness 3 mm) weighted images acquired on the first day follow-up. Logarithmic regression was applied on the correlation coefficients to find the best estimates for the thermal dose thresholds.

**RESULTS** Thermal lesions were successfully produced in all 5 patients. The lesion size in the axial plane (AP and LR) as measured on T2w images (5.0±1.9 mm) was 16% larger than that found on the corresponding T1w scans (4.3±2.0mm), the latter of which primarily revealed vascular damage. As a result, logarithmic regression showed that the best correlation of the ATD to the lesion size on T2w imaging was close to 100 CEM_43_ (linear regression slope=0.98, R^2^ = 0.62), whereas the best correlation of the ATD to the lesion size on T1w imaging was close to 200 CEM_43_ (linear regression slope=0.94, R = 0.84). 17 and 40 CEM_43_ significantly overestimated the lesion size in T1w images by 50% and 30%, respectively. These results are in good agreement with our previous study, which included 36 treatments with peak temperatures mostly above 55°C.

**CONCLUSIONS** An ATD of 100 CEM_43_ showed a good correlation to the lesion size measured on T2w images acquired one day post treatment. This threshold appears to be valid both for lesions produced by sonications above 55°C, and by repeated sonications below 55°C. These results provide important guidance for predicting the lesion size induced during focused ultrasound-based treatment of essential tremor. (No Image Selected).

### O86 Neuroprotection of low-intensity transcranial ultrasound stimulation in ischemic stroke rats

#### J. Sun, H. Li, S. Tong,

##### School of Biomedical Engineering, Shanghai Jiao Tong University, Shanghai, China

###### **Correspondence:** J. Sun

**OBJECTIVES** Transcranial Ultrasound Stimulation (TUS) is a non-invasive neuromodulation technique which modulates the neural activity by delivering specific pulsed ultrasonic waves through intact scalp and skull to the targeted brain regions. In recent years, studies have demonstrated that low-intensity TUS is a safe neuromodulation technique with high focality and deep penetration depth, and has therapeutic effects for brain diseases like epilepsy and stroke in animal models. With these advantages, we have applied TUS in preconditioning and neuroprotection of ischemic rats, so as to verify the neuroprotection effects of TUS in stroke rats.

**METHODS** In the first study, we applied a low-intensity TUS to the ischemic cortex of SD rats immediately after a distal middle cerebral artery occlusion (dMCAO), and used both behavior (neurological severity score) and histological measures were used to assess the neuroprotection effects of TUS on ischemic stroke rats. In the second study, we applied TUS stimulation as a preconditioning to rats before ischemia induced by photothrombosis, and both cerebral blood flow and histological measures was used to assess the neuroprotection effects of TUS preconditioning on ischemic stroke injury.

**RESULTS** In the first study, the rats received TUS after dMCAO showed significantly lower NSS (n=10, 5.5±2.5) than the Control group (n=10, 10.5±1.4) (p<0.01). In addition, the stroke rats received TUS had significantly reduced cortical infarct volume than the control group (13.78% ± 8.18%, n=16, versus 43.39%±2.33%, n=16, p<0.01). What’s more, Immunohistochemical staining indicated reduction of neutrophils in the affected area, and laser speckle imaging showed significant increase of a cerebral blood flow after TUS, which consistently supported the neuroprotection of pTUS in ischemic brain injury. In the second study, the ischemic stroke rats received TUS preconditioning had smaller ischemic areas during stroke induction, and 24 and 48 hours after the stroke, and smaller infarct volume (1.770 ± 0.169%) than the controls (3.215 ± 0.401%) (p<0.01). Moreover, the stroke rats with TUS preconditioning experienced lower volume of brain edema than the control group.

**CONCLUSIONS** In the first study, both behavior and histological results suggested that TUS on ischemic core immediately after ischemic stroke could be neuroprotective. In the second study, the results demonstrated that TUS preconditioning before photothrombosis could provide neuroprotection by increasing the brain’s tolerance to subsequently induced focal ischemic injury.

## Posters

### P1 Therapeutic effects of chemo-agent delivery across the blood-brain barrier using mrgfus: *in vivo* study using drug-resistant breast cancer brain metastasis model

#### Eun-Joo Park, Yuri Cheon, Yun Deok Ahn, Jae Young Lee

##### Radiology, Seoul National University Hospital, Seoul, Korea

###### **Correspondence:** Eun-Joo Park

**OBJECTIVES** Several studies have shown the promising results of drug delivery across the BBB using MRgFUS in combination with microbubbles. However, more studies are required to evaluate this treatment technique prior to apply in clinic. This study was designed to evaluate the therapeutic effects of chemo-agent for brain metastasis cancer by delivering drug across the BBB using MRgFUS andmicrobubbles.

**METHODS** *In vivo* studies were performed in two steps. For the first step, multiple BBB-openings were performed at four different FUS condition along with microbubbles (SonoVue) to find safe and effective treatment condition. The treatment was performed once a week for six weeks. In the second step, breast cancer cells (BT-474) were pre-treated with chemo-agent prior to the inoculation in the rat brain for the brain metastasis model. Animals were treated in three groups: control, chemo-agent treatment only, and chemo-agent treatment with BBB-opening. FUS condition and injection volume of microbubbles for BBB-opening were obtained from the first step experiment. Animals were treated on a weekly basis for six weeks and post-treatment tumor growth monitoring was followed for 12 weeks.

**RESULTS** As restuls of this study, histological evaluation of each BBB-opening FUS condition in combination with SonoVue, therapeutic effects of chemo-agent treatment with BBB-opening, and the survival rate of each treatment will be presented.

**CONCLUSIONS** This study evaluated the FUS condition for multiple BBB-opening in combination with microbubbles, SonoVue. With the treatment conditions, therapeutic effects of chemo-agent delivery across the BBB for brain metastasis cancer model by using breast cancer cells pre-treated chemo-agent.

### P2 Optimizing passive cavitation mapping by refined minimum variance-based beamforming method: performance evaluationsin macaque models for blood-brain barrier disruption

#### Tao Sun^1,2^, Calum Crake^1^, Brian Tracey^2^, Costas Arvanitis^1^, Eric Miller^2^, Nathan McDannold^1^

##### ^1^Radiology, Brigham and Women's Hospital; Harvard Medical School, Boston, Massachusetts, USA; ^2^Electrical and Computer Engineering, Tufts University, Medford, Massachusetts, USA

###### **Correspondence:** Tao Sun

**OBJECTIVES** Microbubble-mediated focused ultrasound (FUS) therapies harness mechanical and/or thermal effects to deliver drugs or ablate tissues. Passive acoustic mapping (PAM) enables the spatio-temporal monitoring of cavitation activity, which is critical for the clinical translation of this technique. Traditional PAM is based ondelay-and-sum (DAS) beamforming, a method whose quality tends to deteriorate due to issues including multi-bubble interference, distortion in the wavefront caused bythe presence of the skull, unmodeled variability of array elements, etc. To provide for robustness, here we consider the use of minimum variance adaptive beamforming toPAM and demonstrate significant improvement in image quality compared to DAS.

**METHODS** Experiments were performed in phantom with macaque skull and *in vivo* for monitoring FUS-induced blood-brain barrier disruption in a clinical MRIguided FUS system (ExAblate 4000, InSightec, Haifa, Israel), which was integrated with a clinical 3T MRI unit (GE Healthcare). A 1024-element phased array was driven to transmit 10-ms pulsed FUS at 220 kHz. RF data for PAM were acquired using a research ultrasound imaging system (Verasonics, Redmond, WA) passively. The imaging probe (L382, Acuson, WA) was a 128-element linear array (fc = 3.21 MHz; bandwidth: ~ 75%). Transition and receiving systems were synchronized and the first180 μs of RF-data were recorded for each burst.

**RESULTS** Minimum variance distortionless response (MVDR) method was evaluated and further improved by adding diagonal loading and using subarray for covariance estimate. Results demonstrate the resolution improvement and contrast enhancement using both traditional and refined MVDR Beamformers compared toDAS. Axial full width at half maximum was tightened down to 79.5% and 38.5% of that in DAS image for traditional and refined MVDR Beamformers, respectively. Moreover, refined MVDR method greatly enhances the robustness when traditional MVDR Beamforming induces more artifacts due to the self-nulling effects (shown in Fig. 1).

**CONCLUSIONS** It was demonstrated that the refined MVDR method improved the image quality significantly compared to traditional DAS method. We anticipate that the proposed methods will improve our ability to monitor and control FUS-induced cavitation-based therapies.

**Fig. 1 (abstract P2).**
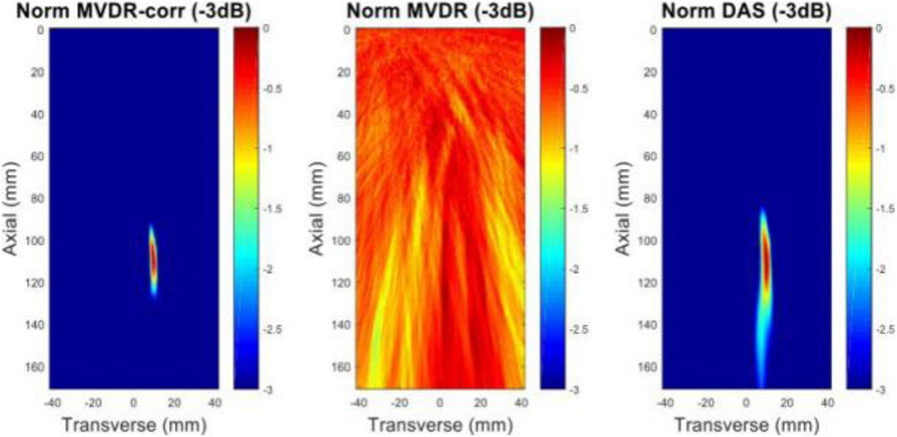
An example to show the -3dB profile. The presented method can enhance the robustness of traditional MVDR when the main scatters were self-nulled

### P3 Focused ultrasound-enabled delivery of radiolabeled nanoclusters to the pons with concurrent pet imaging

#### Dezhuang Ye^1^, Sultan Deborah^4^, Hannah Luehmann^4^, Yuanchun Tai^4^, Josh Rubin^2^, Yongjian Liu^4^, Hong Chen^3, 5^

##### ^1^Mechanical Engineering and Material Science, Washington University in St. Louis, Saint Louis, Missouri, USA; ^2^Pediatrics Hematology/Oncology, WashingtonUniversity in St. Louis, Saint Louis, Missouri, USA; ^3^Biomedical Engineering, Washington University in St. Louis, Saint Louis, Missouri, USA; ^4^Radiology - Rad Sciences, Washington University in St. Louis, Saint Louis, Missouri, USA; ^5^Radiation Oncology, Washington University in St. Louis, SaintLouis, Missouri, USA

###### **Correspondence:** Dezhuang Ye

**OBJECTIVES** Pontine glioma is the single greatest cause of brain tumor-related death in children. Pons is a major structure in the upper part of the brainstem, which is involved in the control of functions such as breathing, hearing, taste, balance, and communication between different parts of the brain. The unique location of the pons precludes surgical interventions while conventional chemotherapy shows little improvement in survival because the blood-brain barrier (BBB) remains intact in pontine glioma. Recent efforts have focused on improvements in chemotherapeutic agents employed and in methods of localized and targeted drug delivery. Several drug delivery strategies, such as convection-enhanced delivery and intranasal delivery, have been proposed to bypass the BBB for the treatment of pontine glioma but have met withminimal success. Focused ultrasound (FUS) combined with microbubbles has been successfully used in the noninvasive and localized trans-BBB delivery of various drugs and several nanoparticles for the treatment of brain cancer. 64Cu-alloyed gold nanoclusters (64CuAuNCs) composed of a few gold atoms and radiolabeled copper atoms are emerging theranostic agents for cancer treatment. Their small sizes with PET imaging capability present unique advantages to be integrated with the FUStechnique. The goal of this study was to demonstrate that FUS can enable the delivery of 64CuAuNCs noninvasively and locally to the pons with concurrent PET imaging capability for quantitative evaluation of the delivery outcome. The long-term perspective is to apply this novel image-guided drug delivery platform for the treatment of pontine gliomas.

**METHODS** First, six wild-type mice were treated by FUS for evaluating the feasibility of safe and localized FUS drug delivery to the pons using a model drug: 40 kDafluorescently-labeled dextran with a hydrodynamic diameter (~2.3 nm) that was close to that of the 64CuAuNCs (3-5 nm). Dextran delivery outcomes were evaluated based on fluorescence images of *ex vivo* brain slices and safety was assessed using histological staining. Second, another group of six mice was used for demonstrating the feasibility of FUS-enabled 64CuAuNCs delivery to the pons. FUS sonicated the left pons in the presence of systemically administrated microbubbles. After FUS sonication, mice were transferred to microPET/CT facility. 64CuAuNCs in 100 μL saline was injected into the mice *via* the tail vein, followed by PET scanning of themice at 1 hr, 4 hr, and 24 hr. To further validate the delivery of 64CuAuNCs, auto-radiography of *ex vivo* brain slices was performed, followed by inductively coupled plasma mass spectrometry (ICP-MS) quantification of the gold concentration in the brain. At last, the biodistribution of 64CuAuNCs was evaluated using gamma counting and ICP-MS.

**RESULTS** Fluorescence images of mouse brain slices showed localized delivery of the dextran at the FUS targeted left side of the pons. Hematoxylin and eosin stainingof the whole brain confirmed that the treatment did not cause histological level tissue damage. PET images showed targeted delivery of 64CuAuNCs to the pons and quantitative analysis of the PET images found the delivery efficiency was1.44%ID/g. Auto-radiography further validated the successeful delivery of 64CuAuNCs to the pons. ICP-MS quantification found 3-fold increase in gold concentration at the FUS-treated left side of the pons compared with the contralateral nontreated right side ofthe pons. Biodistribution study showed the 64CuAuNCs was cleared by liver and kidney, demonstrating the reduced systematic toxicity of this nanoparticle.

**CONCLUSIONS** The unique location of the pons and the intact BBB in pontine glioma present a critical need for noninvasive and localized techniques to overcome theBBB. Although FUS technique has been under development for brain cancer drug delivery for more than one decade, this is the first study that demonstrated the successful drug delivery to the pons. Meanwhile, nanomedicine is the next-generation platform technology for cancer therapy, and FUS has been shown promising in the delivery of several nanoparticles. This study demonstrated the unique integration of the FUS technique with nanoclusters for brain drug delivery. The small sizes of nanoclusters presented unique advantages in trans-BBB delivery and brain parenchyma penetration. Last but not least, contrast-enhanced MRI is currently the standard technique for FUS-induced BBB opening quantification. Contrast-enhanced MRI monitors contrast agent leakage and assumes an indirect correlation between the delivery of the contrast agent and therapeutic agents. PET imaging of radiolabeled nanoparticles provides a noninvasive, highly sensitive, and quantitative method for directly evaluating the trans-BBB delivery of nanoparticles.

### P4 An acoustic emission-feedback planar ultrasound system for localized blood-brain barrier opening and monitoring

#### Yu-Xian Lin^1^, Yu-Chien Lin^1^, Chih-Hung Tsai^1^, Wen-Shiang Chen^2^, Claude Inserra^3^, Hao-Li Liu^1,4^

##### ^1^Electrical Engineering, Chang Gung University, Taoyuan City, Taiwan; ^2^Physical Medicine & Rehabilitation, National Taiwan University Hospital, Taipei, Taiwan; ^3^Université de Lyon, Lyon, France; ^4^Neurosurgery, Chang Gung Memorial, Taoyuan City, Taiwan

###### **Correspondence:** Yu-Xian Lin

**OBJECTIVES** In this study, we proposed a novel planar ultrasound apparatus design that can provide PCD and real-time analysis, with the intention to perform real-time planar ultrasound BBB opening monitoring and control.

**METHODS** A planar ultrasound probe integrating with a lateral mode transducer ring was coaxially arranged, which the former one is to perform energy exposure and the later one is to passively receive the backscattered emission and characterize the subharmonic/ultraharmonic emission spectrum density (ESD) during treatment. Invivo tube phantom experiments were conducted to characterize the dependence of ESD change with microbubble infusion. In-vitro experiments were employed to characterize the dependence of ESD change, and in-vivo experiments were employed to characterize the effect to induce BBB opening. Evans blue extravasations and HE staining was conducted to provide histological confirmation.

**RESULTS** This study demonstrates the capability in using planar ultrasound system to open the BBB. The ESD response well corresponds to the animal brain section, where the EB well stained on the ultrasound exposure position to induce successful BBB opening. For animal group with BBB still intact after exposure, the peak ESD was observed to be 0± 3 dB. On the other hand, in animal group with successful BBB-opening, the peak ESD was observed to be significantly higher (17± 12 dB) than the BBB-intact groups (Fig. 1). The 4-dB ESD level was found to be a valid threshold level to well discriminate the BBB-intact and BBB-opened group. With the integration of the lateral-mode ring transducer, the proposed system also demonstrates the feasibility to monitor the BBB-opened status, making the real-time control of the process become feasible.

**CONCLUSIONS** This study may provide valuable information toward designing a planar ultrasound treatment apparatus for the purpose of BBB opening and brain drug delivery.

**Fig. 1 (abstract P4).**
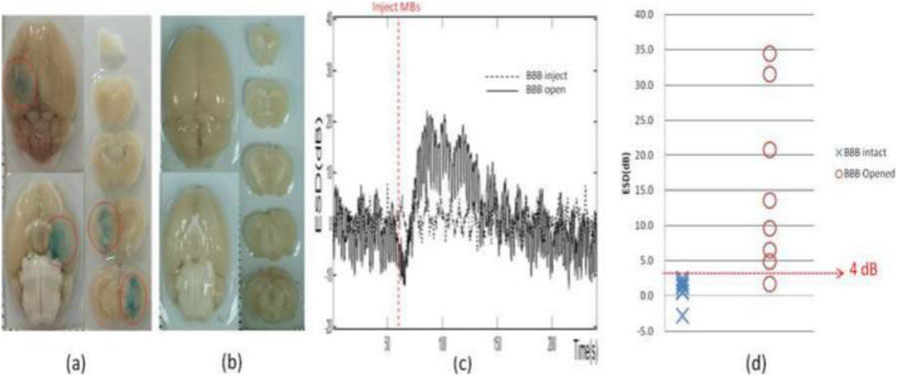
Typical ESD responses and the animal brain sections of a two animals with (a) or without (b) successfully induce BBB opening. The Red circles in (a) shows the EB stained regions which represent the BBB­opened region. (d) Comparison between the peak ESD magnitudes with the BBB status (intact or opened). n = 8 for eachgroup

### P5 Microbubble-facilitated focused ultrasound enhances the delivery of virus-like particles into the brain

#### Chia-Jun Lin, Hong-Wei Yang, Hao-Li Liu

##### Department of Electrical Engineering, Chang Gung University, Taoyuan City, Taiwan

###### **Correspondence**: Chia-Jung Lin

**OBJECTIVES** Microbubble-facilitated focused ultrasound (FUS) has been applied to transiently induce blood-brain barrier (BBB) opening noninvasively and locally. Although substance with moderate size has been shown its feasibility to be delivered into the brain *via* this technique, large molecular delivery (MW > 500 kDa) is still challenging and the delivered effeminacy is still unknown. In this study, we aim to test the CNS delivered efficacy of a novel large molecule, virus-like particles (VLPs;MW = 2000 kDa), into the brain *via* microbubble-facilitated FUS BBB opening, and investigate the penetration and distribution.

**METHODS** A total of 12 ICR mice were employed in this study. A 400 kHz spherically focused transducer was used to deliver ultrasound energy (0.155 MPa; burstlength = 10 ms, PRF = 1 Hz, duration = 60 s). The focal zone was located at left cerebral hemisphere with 2-3 mm depth. Brains were harvested at 4 hours post FUS treatment, and the frozen samples were sectioned and observed with a fluorescent microscope (TissueFAX Plus, Austria). Tissue slices were then stained byimmunofluorescence and observed to investigate the distribution of VLPs in brain cells. GFP-bounded dextrans with the size ranging from 70 – 2000 kDa were employed as another molecular surrogate and compare with the VLP delivery. Evans blue (EB) was used as the indicator of BBB opening.

**RESULTS** FUS exposure with the presence of microbubbles were shown to be able to locally open the BBB at the target site. VLP has shown evidence to be delivered into the brain (Fig. 1). When correlated with the observation of GFP-bounded dextrans, it was observed that 70 kDa dextrans spread in the left hemisphere, while 2000 kDadextrans aggregated at some spots. More VLPs are distributed near the hemorrhage sites in the left hemisphere after treated with microbubble-facilitated FUS, comparedto the non-hemorrhage sites. VLPs are co-localized with neuron nuclei (NeuN), while not obviously with gilal fibrillary acidic protein (GFAP). 2000 kDa dextranaggregated between the brain cells but showed no evidence of the entry in brain cells.

**CONCLUSIONS** In this study, we have demonstrated that large molecular delivery up to 2000 kDa is feasible when combining with microbubble-facilitated FUS BBBopening. We observed that the VLPs when entering into brain is able to penetrate into neuron cells, support the VLP-cell endocytosis is occurred. VLP preserve superiority in its high endocytosis feature, making the VLP has the potential to serve as a gene carrier to perform CNS gene delivery.

**Fig. 1 (abstract P5).**
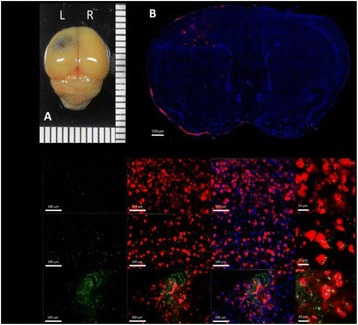
(A) After microbubble­facilitated FUS treatment at left hemisphere, EB entered into the brain, indicating the BBB is opened. Some hemorrhage was observed. One unit of scale is 1 mm. (B) VLP fused with mcherry fluorescent protein entered into the left hemisphere after BBB opening induced by microbubble­facilitated FUS.VLP was shown as red. Nuclei were stained by DAPI. (C) Compared with saline injection or VLP injection without FUS, more VLPs were co­localized with NeuN after BBB opening, indicated its entry into the neuron cells. VLP was presented as green, NeuN was as red and nuclei was as blue

### P6 The kinetics of P-glycoprotein after blood-brain barrier disruption in rat brain by MRI-guided focused ultrasound

#### Mun Han, Danbi Kim, Sanghyun Ahn, Juyoung Park

##### Daegu-Gyeongbuk Medical Innovation Foundation, Daegu, Korea

###### **Correspondence:** Mun Han

**OBJECTIVES** Multi-drug resistant proteins in brain endothelial cells pump drugs in brain tissues out into the blood and could diminish drug retention and drug efficacy. In previous our study, a representative multi-drug resistant protein, P-glycoprotein (P-gp), was significantly reduced at 24 hrs after blood brain barrier (BBB) disruption by focused ultrasound (FUS). In this study, we investigated the kinetics of the P-gp after FUS-induced BBB disruption.

**METHODS** The rat brain in thalamus was sonicated (0.5~0.55 MPa) transracially using a 1 MHz FUS transducer with 10 ms bursts of ultrasound wave at 1Hz pulse repetition frequency for 120s, combined with intravenous injection of a microbubble ultrasound contrast agent (Definity; 400 μl/kg). T1 and T2 weighted MRI scan were used to guide FUS sonication into the targeted brain and confirm the BBB disruption. Then, the sonicated rat brains were extracted at different time points (1 hr, 1, 2, 3, 5day) after BBB disruption. In order to measure the P-gp expression, choroid plexuses were removed from all ventricles and then western blot analysis was performed using antibody against P-gp (1:100) and β-actin (1:2000). P-gp expression sonicated area and non-sonicated area were compared.

**RESULTS** The BBB disruption was confirmed in the thalamus region through T1 weighted images with MR contrast agent (Fig. 1A). All the results of western blot were calculated on amounts of P-gp compared with β-actin. In addition, the change of P-gp in sonicated area shows percent assuming that P-gp of non-sonicated area is100%. At 1 hour, P-gp of sonicated region was reduced by 15% and showed the lowest reduction on 1 day (76%). From 2 to 5 day, P-gp seemed to be recover gradually. It was reduced by 61% on 2 day and 43% on 3 day. On 5 days, it was completely recovered and showed no decrease (Fig. 1B).

**CONCLUSIONS** The results of this study provide the information needed to take into account the dynamics P-gp change over time after FUS-induced BBB disruption. Therefore, these results could suggest more detailed treatment protocols when FUS-induced BBB disruption treatment along with P-gp substrate drug.

**Fig. 1 (abstract P6).**
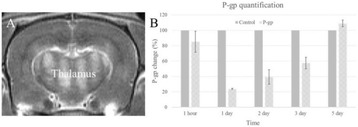
MR image (A), P­glycoprotein quantification graph (B)

### P7 Focused ultrasound-induced blood-brain barrier opening enhances GSK-3 inhibitor delivery for amyloid-beta plaque reduction

#### PoHung Hsu^1,2^, Yi-Hsiu Chung^2^, KunJu Lin^3^, LiangYo Yang^4,5^, Tzu-Chen Yen^2^, Hao-Li Liu^1,6^

##### ^1^Electrical Engineering, Chang Gung University, Taoyuan City, Taiwan; ^2^Center for Advanced Molecular Imaging and Translation, Chang Gung Memorial Hospital, Taoyuan City, Taiwan; ^3^Department of Nuclear Medicine, Chang Gung Memorial Hospital, Taoyuan City, Taiwan; ^4^Department of Physiology, School of Medicine, College of Medicine, China Medical University, Taichung, Taiwan; ^5^Department of Biotechnology, Asia University, Taichung, Taiwan; ^6^Medical Imaging Research Center, Institute forRadiological Research, Chang Gung University and Chang Gung Memorial Hospital, Taoyuan City, Taiwan

###### **Correspondence:** PoHung Hsu

**OBJECTIVES** Alzheimer's disease (AD) is a chronic neurodegenerative disease that is the leading cause of age-related dementia. Currently, therapeutic agent delivery tothe CNS is a valued approach for AD therapy. Unfortunately, CNS penetration of therapeutic agents is greatly hampered by the blood-brain barrier (BBB). Focused ultrasound (FUS) exposure has been demonstrated to temporally open the BBB, thus promoting therapeutic agent delivery to the CNS for AD therapy. In this study, we aimed to evaluate whether the use of FUS-induced BBB opening to enhance GSK-3 inhibitor delivery can promote Aβ plaque clearance and synthetic regulation in a transgenic small animal model (Fig. 1a).

**METHODS** FUS-induced BBB opening on APP/PS1 transgenic mice was performed unilaterally, with the contralateral hemisphere serving as a reference. AV-45PET/CT imaging was attempted to detect plaque distribution and concentration *in vivo*, and autoradiography (ARG) was conducted *ex vivo* to quantitatively confirm theAβ plaque reduction. Immunohistochemistry staining was also performed to confirm the GSK-3 expression level.

**RESULTS** Results from AV-45 PET/CT imaging showed positive Aβ plaque regulation *in vivo*, and *ex vivo* autoradiography (ARG) further showed significant radiolabelled tracer detectability (up to 26.72% Aβ plaque reduction). Immunohistochemistry revealed that the GSK-3 inhibitor effectively blocked plaque synthesis up to60.6% in the GSK-3 immunoreactive area, confirming the proposed therapeutic pathway.

**CONCLUSIONS** We demonstrated that FUS-induced BBB opening to enhance GSK-3 inhibitor delivery can efficiently reduce amyloid-beta plaques in transgenicmouse brains. The results showed that the use of AV-45 PET imaging can serve as a diagnostic tool for *in vivo* quantitation of plaque clearance, while *ex vivo* autoradiography (ARG) can provide radio-labelled tracer detectability. This study improves our understanding of how ultrasound can be used to enhance AD therapeutic molecule delivery, and promote advances in discovering new therapeutic strategies for neurodegenerative disease.

**Fig. 1 (abstract P7).**
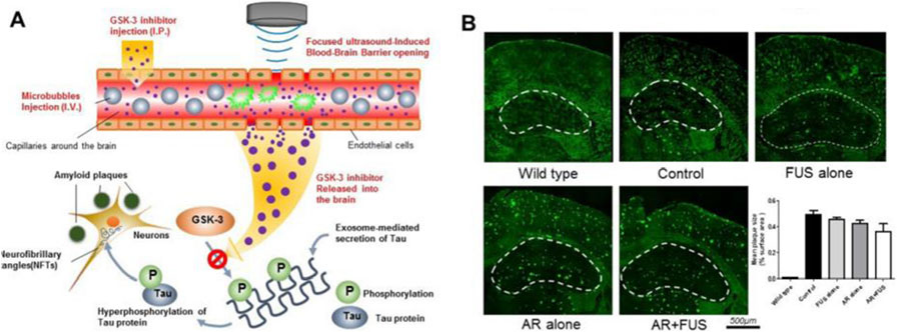
(A) The conceptual schematics of this study. (B) Thioflavin­S staining and quantification of amyloid­beta plaque sizes in the hippocampus area. There was a7.83% (FUS alone), 14.68% (AR alone) and 26.72% (AR+FUS) decrease in comparison to the control group. AR = AR­A014418; FUS = focused ultrasound (Fig. 1b)

### P8 Novel approach to study metabolic changes after FUS-mediated blood-brain barrier disruption

#### Thiele Kobus, Tom Peeters, Andor Veltien, Arend Heerschap, Tom Scheenen

##### Radboud university medical center, Nijmegen, Netherlands

###### **Correspondence:** Thiele Kobus

**OBJECTIVES** Focused ultrasound (FUS) in combination with microbubbles can be used to temporarily and locally disrupt the blood-brain barrier (BBB) [Hynynen, 2001]. However, little is known about the exact mechanism of this technique and its effects on the brain. To get more insight, we would like to study metabolic changes after disruption of the BBB. Many metabolites contain carbon-atoms of which approximately 1% is the isotope 13C that can be detected by magnetic resonance imaging (MRI). Due to the low abundance and low gyromagnetic ratio, the sensitivity of 13C MRI is low. Recently, a method has become available to increase the sensitivity of13C-labeled substrates more than 10.000 fold by dynamic nuclear hyperpolarization (DNP) and create a solution that can be injected [Ardenkjaer-Larsen, 2003]. After injection of the sample, the conversion of the substrate into other metabolites can be observed with 13C MRI enabling the study of fast dynamic metabolic processes in vivo. In this proof of concept, we demonstrate the feasibility of combining FUS-mediated disruption of the BBB with hyperpolarized 13C MRI using DNP. In the near future, this approach might reveal the influence of the BBB on the uptake of hyperpolarized agents or alterations in metabolism due to FUS-mediated BBB opening.

**METHODS** The experimental protocol was approved by the National Animal Research Authority. In two mice, the BBB was disrupted using FUS and followed by hyperpolarized 13C MRI. All experiments took place on a 7T animal system (Clinscan, Bruker). For BBB disruption, animals were anesthetized and positioned in a dedicated MR-guided FUS set-up (IGT, France). MR reference images were acquired for ultrasound targeting in the left frontal lobe of the brain. The sonication (duration=120s, 10ms bursts, burst frequency=1Hz) was performed immediately after the injection of microbubbles (Sonovue, Bracco, 500 ul/kg) using a 650 kHz transducer (focal spot 3.5 x 22 mm). Subsequently the FUS set-up was replaced with a set-up for 13C-imaging, containing a dedicated 13C surface coil inside a small 1H volume coil for anatomical reference imaging. An interesting metabolite to study is pyruvate, which is the product of the glycolysis of glucose and is further metabolized in the Krebs’s cycle or converted tolactate. Therefore, [1-13C] pyruvate was polarized in an in-house-built polarizer as described previously [Breukels, 2015]. The polarized substrate was rapidly dissolvedin a 1xPBS buffer solution to a final concentration of 80mM. 300μl HP pyruvate was injected within 15s after dissolution. To enable dynamic imaging of the conversion of pyruvate to lactate, a slice selective gradient echo sequence was developed that makes it possible to simultaneously image pyruvate and lactate based on their chemical shift difference [Steinseifer 2013]. Every 1.6s an image is acquired that consists of a pyruvate and lactate image [Example, Fig. 1]. A phantom containing [1-13C] pyruvate and [1-13C] lactate was placed next to the animal to obtain a 13C reference image for image registration. From these images, concentration curves of pyruvate and lactate can be obtained that by plotting the total signal intensity over time of a region of interest. These concentration curves show the conversion of pyruvate to lactate (Example, Fig. 2). Since gadolinium-based contrast agents cannot cross the intact BBB, Magnevist (0.25 ml/kg) was injected after 13C-imaging to verify opening of the BBB using pre- andpost-contrast T1-weighted MR imaging.

**RESULTS** Pre- and post-contrast T1-weighted images in Fig. 3 confirm a successful opening of the BBB, as we observed a hyperintense region were the brain was sonicated. We successfully obtained pyruvate signal in the 13C images, but the intensity of the lactate signals was too low to observe. The pyruvate image did not show any evident signal intensities that can be related to BBB-opening.

**CONCLUSIONS** In this study we successfully showed the feasibility of combining BBB disruption using FUS with dynamic imaging of hyperpolarized 13C-labeled compounds. The BBB was disrupted prior to, and remained open during our hyperpolarized 13C-imaging experiment, as confirmed by contrast-enhanced MRI. However,SNR of the hyperpolarized signals is currently too low to draw conclusions upon enhanced uptake or release of hyperpolarized metabolites in the targeted brain region.Therefore, improvement of polarization levels and further optimization of the imaging parameters is necessary.Despite challenges that have to be overcome, this approach enables delivery of hyperpolarized agents to the brain and will allow us to study metabolic alterations due todisruption of the BBB *in vivo* in the near future.

**Fig. 1 (abstract P8).**
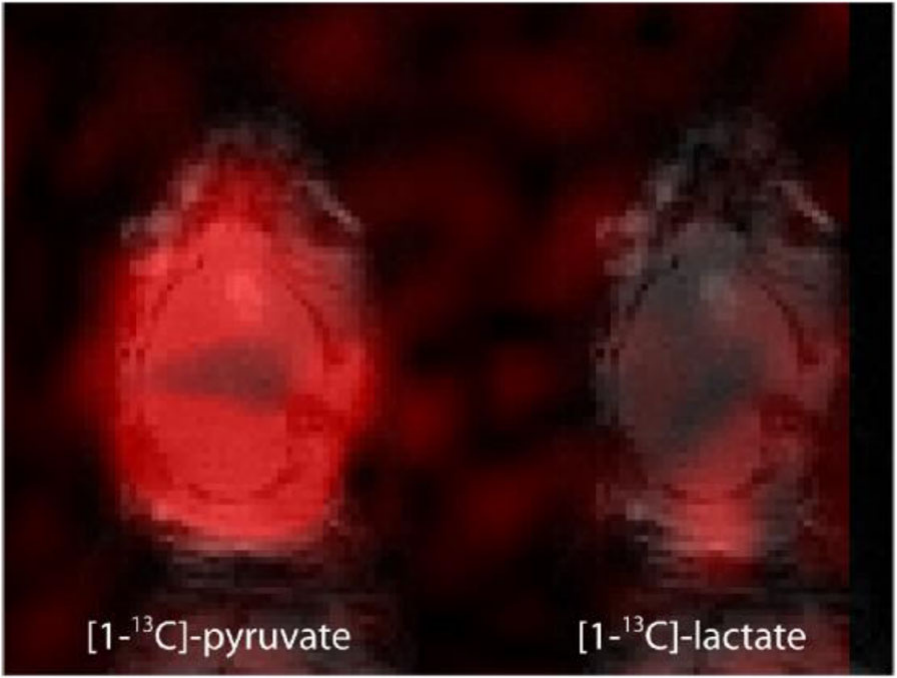
Example of a hyperpolarized pyruvate and lactate image mapped to the T2-weighted reference image (no BBB disruption)

**Fig. 2 (abstract P8).**
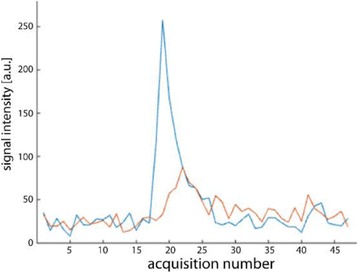
Example of a dynamic time evaluation of hyperpolarized pyruvate and lactate signals (no BBB disruption). The total signal intensity of a region of interest isplotted over time. Data were not corrected for T1­decay and signal loss due to RF pulsing

**Fig. 3 (abstract P8).**
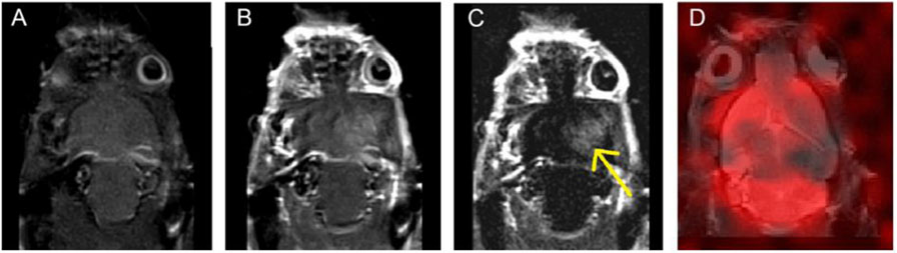
Pre­ (A) and post­contrast (B) images after gadolinium administration. The subtraction image (C) shows a hyperintense region where the BBB was successfully disrupted. The corresponding 13C image (D) of hyperpolarized pyruvate after BBB disruption

### P9 A low-cost phased array system for ultrasound neuromodulation

#### Wu Sun, Juan Zhou, WenBin Yan, JiaXing Yang, Weibao Qiu, Hairong Zheng

##### Shenzhen Institutes of Advanced Technology, Chinese Academy of Sciences, Shenzhen, China

###### **Correspondence:** Wu Sun

**OBJECTIVES** Ultrasound has been shown to non-invasively stimulate and inhibit neuronal activities under various conditions like stimulating auditory nerve responses and suppressing sensory-evoked potentials in the primary visual cortex. Currently most of the published works for ultrasound neuromodulation are based on single element focused transducer. In contrast with single element transducer, phased array transducer is more flexible to change the target location, and potentially to simulate multiple positions simultaneously. High intensity focused ultrasound (HIFU) array system can be applied to neuromodulation by decreased the ultrasound energy, however, such a system is usually quite expensive and bulky. Thus a low cost ultrasound array system specifically for ultrasound neuromodulation is needed. This paper presents an ultrasound phased array system that incorporates a 256-element hemispheric transducer for neuromodulation. Test results show that the system has potential to achieve multiple position neuromodulation.

**METHODS** Our system includes two parts: 1) ultrasound electronic system; 2) phased array transducer (256-element array transducer, Imasonic, France). The schematic of the ultrasound electronic system is shown in Fig. 1a. The system consists of five major circuit modules: an field programmable gate array (FPGA) (Cyclone V5CGXFC7D7F31C8, Altera, USA) based digital board which presents control kernel of the system, multiple channels power amplifier, high speed power sensing circuits, low pass filters, and a module of matching circuits. The FPGA-based digital board has three functions. Firstly, it is used as a communication unit with PC through an USB3.0 interface (CYUSB3014, Cypress, USA). Secondly, it can monitor the excitation power by incorporating high speed power sensing module. Lastly, the most important function for the board is to generate multiple channel source waveform and each channel waveform can be controlled individually. The phase information of the waveform is flexible to change so that the system can implement different beamforming algorithms. The power amplifier board is used to amplify the low voltage waveform to drive the transducer. The low pass filter is connected with the power amplifier board to shape the high-voltage waveform to match with transducer. A matching circuit is employed to adjust the impedance characteristics to achieve maximal power transfer. And a dynamic phased control beamforming algorithm is developed to achieve flexible beam focusing in the acoustic field.

**RESULTS** The prototype of the fabricated ultrasound system is shown in Fig. 1b. Each module is achieved by separate printed circuit boards (PCBs). The phase difference error for all channels is less than 7 ns when the center frequency of transducer is set to 800 KHz. It means that the phase error of the proposed ultrasound systemis about 2 degrees, which is able to support a good beamforming performance. Dynamic focusing can be achieved in a circle with a radius of 10 mm, and the focal depth can be adjusted in the range of 4-10 cm. The system can work in a pulsed mode or a continuous mode for 50-300 ms. The system is able to hit a water column which theheight is about 5 cm. The beamforming quality of the system is also evaluated by an acoustic hydrophone.

**CONCLUSIONS** In this study, we present a prototype design of a low cost ultrasound array system specifically for neuromodulation. Test results demonstrate that the proposed system has a great potential to improve the level of neuromodulation. Future work such as improvement of beamforming performance in different acoustic mediums and validation of primate animal studies *in vivo* will be carried out.

**Fig. 1 (abstract P9).**
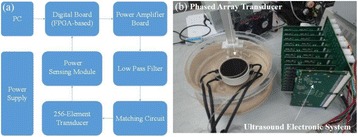
(a) The schematic of the ultrasound electronic system. (b) the prototype of the proposed ultrasound system

### P10 Test study for the electro-acoustic conversion efficiency of focused ultrasonic transducer based on virtual instrument

#### Yang Liu^1^, Jianwen Tan^2, 1^, Deping Zeng^1, 3^, Zhiming Zhong^1^

##### ^1^Biomedical Engineering, Chingqing Medical University, Chongqing, China; ^2^Chongqing Communication Institute., Chongqing, China; ^3^National Engineering ResearchCenter of Ultrasound Medicine, Chongqing, China

###### **Correspondence:** Yang Liu

**OBJECTIVES** The electro-acoustic conversion characteristic of ultrasonic transducer is required to be tested when developing focused ultrasound treatment equipment, with the actual electro-acoustic characteristics of transducer to determine working frequency and driver parameters of focused ultrasound therapy system. Therefore, when the HIFU therapy system developed, there is a massive workload in the measurement of electro-acoustic conversion characteristics of focus ultrasonic transducer. It has put forward higher request to the efficiency and convenience of the electro-acoustic conversion characteristics test system.

**METHODS** An automatic test system for the electro-acoustic conversion characteristic of the transducer based on virtual instrument has been developed in this letter. The input electric power of the transducer is measured by electric power meter based on directional coupler, radiation force balance is used to measure the output soundpower of the transducer. By use of the graphic development environment LabVIEW, an upper computer automatic test software was developed based on virtual instrument technology, which can operate with the real-time data acquisition and data processing, analysis and calculation of the data.

**RESULTS** Based on this test system, the electro-acoustic conversion efficiency of the focused ultrasound transducer at different frequencies and the driving power also tested in this paper, the bandwidth of the transducer was analyzed from the point of view that the measured electro-acoustic conversion efficiency of transducer, which discussed electro-acoustic conversion efficiency of transducer with the tracking problem of different driving power and operating frequency based on electrical reflectioncoefficient (Figs. 1, 2, 3).

**CONCLUSIONS** With the analysis of test results shows that the proposed real-time monitoring tool based on electrical reflection coefficient which measured by directional coupler can also applied to tracking the frequency of the ultrasonic transducer, which can improve the electrical power transmission efficiency and the output acoustic power of ultrasonic transducer, and there is important significance on the focused ultrasound transducer to ensure safety, stability and efficient operation.

**Fig. 1 (abstract P10).**
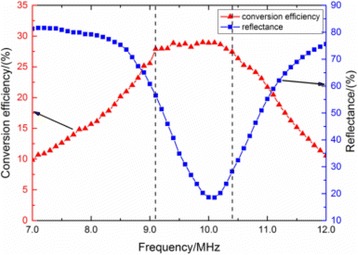
The electro-acoustic conversion efficiency and reflectance of focused ultrasound transducer at different frequencies

**Fig. 2 (abstract P10).**
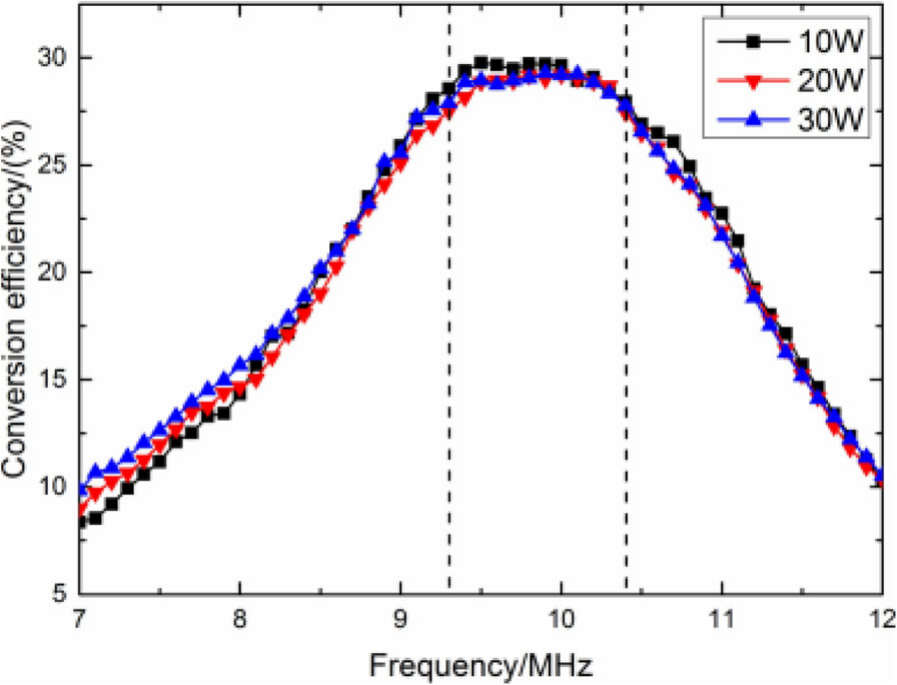
The electro-acoustic conversion efficiency and reflectance of transducer under different driving electric power

**Fig. 3 (abstract P10).**
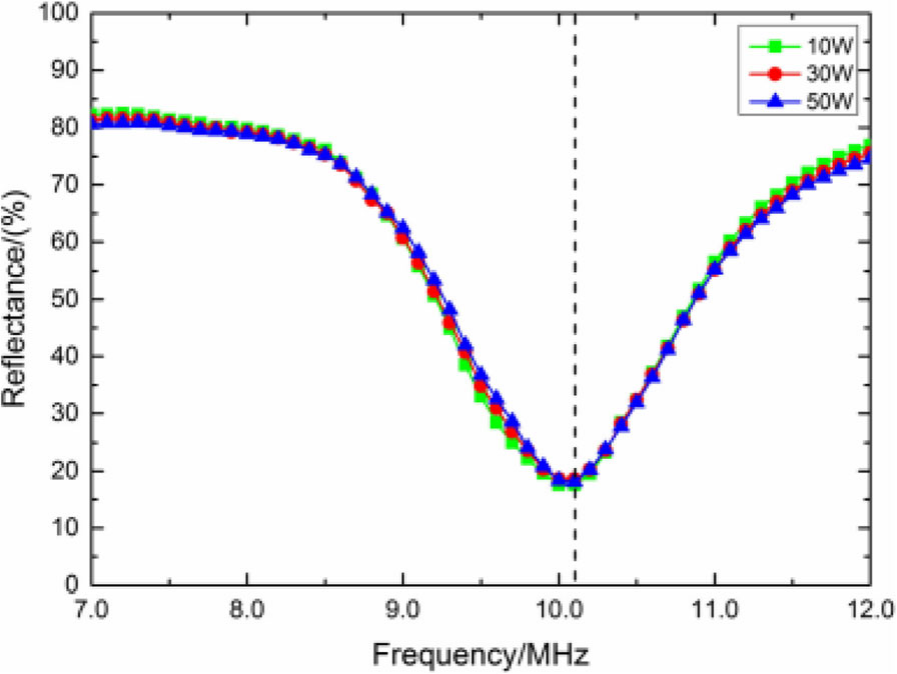
The reflectance of transducer under different driving electric power

### P11 Multifunctional pulse generator unit for high-intensity focused ultrasound system

#### Satoshi Tamano^2,1^, Shin Yoshizawa^2^, Shin-ichiro Umemura^2^

##### ^1^Hitachi, Ltd., Kokubunji, Japan; ^2^Tohoku Univ., Sendai, Japan

###### **Correspondence:** Satoshi Tamano

**OBJECTIVES** High-intensity focused ultrasound (HIFU) therapeutic systems have made it possible to treat diseases with fine spatial resolution. One critical technical challenge of HIFU is to generate high-voltage, large-current, multi-channel pulsed signals to effectively excite the low-impedance HIFU transducers. This paper presents the development of multifunctional pulse generator unit for HIFU system. The pulse generator can produce pseudo sinusoidal wave, triggered-HIFU mode pulse and second-harmonics superimposed shock wave lithotripsy pulses for HIFU transmission. We report the development of a novel multifunctional, reconfigurable pulse generator using FPGA and high-voltage MOSFETs.

**METHODS** The pulse generator is interfaced with a PC and receives commands, waveform data, focusing delay data through a high-speed USB 2.0 micro controller. The USB microcontroller transmits communication information with the PC to the control FPGA. The control FPGA stores instruction information from the PC internalregisters, and delivers transmission waveform information and transmission focus data to the transmission FPGA. For data communication between the control FPGA andthe transmission FPGAs, information is transferred using a dedicated local bus. In our system, 32 channels transmission can be performed per transmission board. Sincewe use HIFU transducer consisting of 128 elements, we used four transmission boards. The control board performs generation of actual ultrasonic wave sequences and arbitration of the transmission board control bus as well as the functions described above. The transmission board includes a transmission FPGA for generating a timing signal for ultrasonic transmission based on a command transmitted from the control FPGA, RAMs that stores transmission focus data, N-channel and P-channelMOSFETs that actually generates 400 Vpp ultrasonic transmission signal, MOSFET drivers for driving MOSFET. In this study, the operation of the transmission circuitwas evaluated using our prototype dual transmission frequencies transducer. This prototype transducer consists of seven elements that can transmit one and two MHz ultrasonic waves. With these two transmission frequencies, since the impedance of the transducer is around 150 Ω, it is necessary to apply a transmission circuit withlarge driving capability.

**RESULTS** In pseudo sinusoidal transmission waveform mode, the test results show that the power consumption is reduced by 14.8 % and MOSFET maximum temperature rise is reduced by 11.5 °C by using the newly proposed circuit than the our previous class D circuit. Also, it can be seen that the third and fifth harmonic components can be suppressed by 23.9, 30.5 dB, respectively. Therefore, the transmission waveform becomes closer to the sinusoidal waveform. As a result of the above, we quantitatively evaluated the power saving effect by increasing the number of power supply steps and the effect of reducing the device temperature rise. In the triggered-HIFU mode, high power (>300 Vpp) and a short time (several microseconds) ultrasonic radiation called trigger-burst, and a medium output (< 100 Vpp) andlong duration (several seconds) ultrasonic radiation called heating-waves are used in combination. In the transmission circuit, heating-waves has a larger amount of heat generation than trigger-burst, so there was a big problem how to realize a circuit with reduced device power consumption. The proposed circuit can 18.9% lower power consumption than conventional class D circuit, and 33.6 °C suppress MOSFET temperature rise. In peak negative enhanced second-harmonics superimposed shock wave lithotripsy mode, by using the proposed circuit, it was possible to realize a concavity of the positive voltage side output, and the second harmonic ratio approached thetheoretical value 2.0 dB from the class D circuit.

**CONCLUSIONS** We modified the HIFU ultrasonic transmission circuit previously reported at ISTU 2016 and created a HIFU transmission unit with 128 channelsintegrated. In particular, by increasing the transmission voltage level from four levels to seven levels, MOSFET device heat generation could be greatly reduced. At the same time, we achieved a significant reduction in power consumption. As a result, the risk of MOSFET damage is reduced, and HIFU transmission could be executedstably and safety. In our newly proposed circuit, it was demonstrated that it is effective not only for transmission of pseudo sinusoidal wave but also for triggered-HIFUmode and second-harmonics superimposed shock wave lithotripsy. The applicability of our proposed circuit has expanded.

### P12 The improvement effect of magnetic microbubbles on HIFU-induced hyperthermia effect

#### Dongxin Yang, Yanye Yang, Guangyao Xu, Xiasheng Guo, Juan Tu, Dong Zhang

##### Nanjing University, Nanjing, China

###### **Correspondence:** Dongxin Yang

**OBJECTIVES** Encapsulated microbubbles (MBs) have been widely used to enhance high intensity focused ultrasound (HIFU) -induced hyperthermia by making use of its ability of increasing acoustic energy absorption and lowering the cavitation threshold. To balance the needs of treatment efficiency and safety, there is increasing demand of more efficient encapsulated MB agents that can quickly achieve sufficient hyperthermia effect while minimizing the damage to surrounding tissues.

**METHODS** In the present work, superparamagnetic iron oxide nanoparticles (SPIO) were coupled to perfluorocarbon-filled, albumin-encapsulated microbubbles (referred as SPIO-albumin MBs) to enable a stronger enhancement of the HIFU-induced hyperthermia effect than regular albumin-encapsulated ones. The thermal enhancement capacity of SPIO-albumin MBs and albumin-encapsulated MBs was investigated based on both experimental measurements and numerical simulations ofthe temperature change at HIFU focus.

**RESULTS** The results show that, comparing with the use of albumin-encapsulated MBs, the adoption of SPIO-albumin MBs will bring about quicker temperatureelevation rate and higher peak temperature.

**CONCLUSIONS** An improvement can be made to the enhancement of the HIFU-induced hyperthermia by using SPIO-albumin MBs rather than albumin-encapsulated ones, which is because the thermal properties of the two kinds of microbubbles are different. With these advantages, these SPIO-albumin MBs can be introduced tospecific locations of interest to intensify the thermal effect of HIFU much more efficiently, which might enable more ideal non-invasive controllable hyperthermia treatment strategies and applications.

### P13 Axial controllable multiple traps of acoustic vortices generated by directional sources

#### Yuzhi Li^1^, Gepu Guo^1^, Qingyu Ma^1^, Juan Tu^2^, Dong Zhang^2^

##### ^1^Nanjing Normal University, Nanjing, China; ^2^Nanjing University, Nanjing, China

###### **Correspondence:** Yuzhi Li

**OBJECTIVES** Characterized by the pressure circle and phase spiral, acoustic vortex (AV) can be used to manipulate objects with its orbital angular momentum androtation torque. Compared with light, acoustic wave can go into media non-destructively with deeper penetration depth, which makes it possible to manipulate particles inside object using AV, exhibiting a prosperous future in biomedical engineering. In previous studies, AV was often investigated under the framework of point source radiation with acoustic diffraction. However, the point source based model is not practical for directional sources with big ka values, which are influenced by beampatterns with obvious side lobes. Consequently, more attentions should be focused on the distributions of AV generated by directional sources.

**METHODS** The phase coded approach is employed to generate controllable AV using directional sources. Based on the radiation theory of planar piston source andcoded initial phase, the physical mechanism of AV is theoretically investigated with explicit formulae. The principles of main-AV (M-AV) and vice-AVs (V-AVs) generated by the main lobes and the side lobs of the sources are analyzed. The number and locations of the formed M-AV, V-AVs and the corresponding vortex valleys (VVs) are calculated for different ka values. And the generations of axially controllable multiple traps are discussed based on Gorkov’s theory. The proposed theory isalso verified by numerical studies and experimental measurements for different ka values at the frequency of 1 MHz.

**RESULTS** It is proved that obvious M-AV, V-AV and VVs can be generated for higher ka with the least value of 3.83. Corresponding to the 8-source experimental systemfor ka=29.32, the measured axial profiles and the cross-sectional distributions at different heights show good agreements with the simulated ones, and obvious phase spirals of M-AV and V-AVs are also demonstrated. Several VVs with almost pressure zero are also observed on the center axis to form multiple traps, which can beaxially controlled for ka adjustment.

**CONCLUSIONS** It is demonstrated that, for bigger ka, besides M-AV generated by the main lobes of the sources, cone-shaped V-AVs produced by the side lobes are closer to the source plane at relatively lower pressure. Corresponding to the radiation angles of press valleys between the main lobe and the side lobes of sources, VVs with almost pressure zero can be generated on the central axis to form axially controllable multiple traps with the locations controlled by ka adjustment. The results provide the feasibility of deep-level control of AV inside object and suggest the application potential of multiple traps for particle manipulation in biomedical engineering.

### P14 Study on the acute injury effect on candida albicans by low-frequency and low-intensity ultrasound

#### Yang Xiang

##### Chongqing Medical University, Chongqing, China

**OBJECTIVES** To investigate the effects of Low-frequency and Low-intensity Ultrasound (LFLIU) on the acute injury of candida albicans, and to investigate the effect of LFLIU on the permeability of the cell wall.

**METHODS** Concentration is 1.5 x 107 cfu/ml of Candida albicans bacteria liquid, with 5 ml bacteria to single flageolet culture plate. With the frequency for 42 kHz, probe diameter is 5 cm circular planar ultrasonic treatment head, ultrasonic intensity was selected 0.13 W/cm2, 0.35 W/cm2 irradiation six well culture plate beads bacterium solution for 5 min, After 48 h count ultrasound irradiation on petri dish culture survival of colonies in a petri dish. Transmission electron microscope and scanning electron microscope were used to observe the external shape and internal structure of the bacteria.

**RESULTS** Different doses of ultrasound irradiation 5 min, 48 h after culture dish colony average count shows, Control group colony count to 21 cfu, ultrasonic intensity0.13 W/cm2 group of colony count for 20 cfu, ultrasonic intensity of 0.35 W/cm2 group of colony count for 14 cfu.LFLIU candida albicans, scanning electron microscope(SEM) visible thalli were swollen shape becomes large, Under the scanning electron microscope (SEM) visible thalli was swelling, the shape becomes large, the extracellular fluid into the cells increased obviously; Transmission electron microscope (TEM) see nuclear matter at the edge of the aggregation, cell membrane damage, formation of vacuoles, large amounts of water into cells.

**CONCLUSIONS** With the increase of ultrasonic irradiation intensity, significantly increased the mortality rate of Candida albicans; Candida albicans in LFIU can promote the increase of the permeability of the cell wall of.

### P15 Transducer directivity influence on artifacts reduction for magnetoacoustic tomography with magnetic induction

#### Gepu Guo^1^, Qingyu Ma^1^, Juan Tu^2^, Dong Zhang^2^

##### ^1^Nanjing Normal University, School of Physics and Technology, Nanjing, Jiangsu, China; ^2^Nanjing University, Nanjing, China

###### **Correspondence:** Gepu Guo

**OBJECTIVES** As a novel noninvasive modality of detecting electrical conductivity variation for tissues, magnetoacoustic tomography with magnetic induction (MATMI) has been demonstrated to have the capability of distinguishing the early pathological changes of biological tissues inside the object. In previous studies, the transducer was usually simplified as an ideal or omnidirectional receiver without the consideration of its directivity and scanning radius. However, the properties of transducer play a vital role in signal acquisition and image reconstruction. In order to optimize image reconstruction and eliminate the image artifacts for MAT-MI, the influence of transducer was investigated theoretically for a two-layer eccentric spherical tissue model based on the principles of acoustic dipole radiation and transducer reception.

**METHODS** Based on the principles of magnetic excitation, acoustic vibration, acoustic dipole radiation and transducer reception, numerical simulations are performed for a two-layer eccentric spherical phantom model. The factors that affect transducer directivity are analyzed, and the distributions of acoustic pressure and waveform are simulated using the transducers with omni-directivity, strong-directivity and uni-directivity. Then the MAT-MI images reconstruct with the collected acoustic waveforms are achieved and compared to the corresponding model to analyze the affect of artifacts reduction.

**RESULTS** It is demonstrated that the image artifacts of MAT-MI is obvious for waveform collection using omni-directional transducer. According with the increase of transducer directivity, the detection angle of the receiver decreases with an increased sensitivity. Especially for a uni-directional transducer, the collected pressure reflectsthe strength of acoustic vibration along the normal direction of the receiver, which can be used to reconstruct the conductivity contrast image without artifacts. In practical applications, large-radius transducer with strong directivity can be applied as an approximate uni-directional receiver to improve image quality with little artifacts aspossible. In addition, to realize narrow detection scope with a fixed transducer, the scanning radius should also be optimized to achieve acceptable signal to noise ratioand peak pressure ratio. The favorable results confirm the influence of transducer on MAT-MI and provide the fundamentals for transducer selection in further study to improve the accuracy of electrical impedance reconstruction.

**CONCLUSIONS** It is concluded that large-radius transducer with strong directivity can be applied as an approximate uni-directional receiver to improve image quality with little artifacts as possible. In addition, to realize narrow detection scope with a fixed transducer, the scanning radius should also be optimized to achieve acceptable signal to noise ratio and peak pressure ratio. The favorable results confirm the influence of transducer on MAT-MI and provide the fundamentals for transducer selection in further study to improve the accuracy of electrical impedance reconstruction.

### P16 A method for evaluating the relationship between the inertial cavitation duration and the acoustic parameters

#### Mouwen Cheng^1^, Yutong Lin^1^, Alfred C. Yu^2^, Peng Qin^1^

##### ^1^Department of Instrument Science and Engineering, Shanghai Jiao Tong University, Shanghai, China; ^2^Department of Electrical and Computer Engineering, TheUniversity of waterloo, Waterloo, Alberta, Canada

###### **Correspondence:** Mouwen Cheng

**OBJECTIVE** Inertial cavitation, triggered by the ultrasound and microbubbles, is considered to be the main mechanism for sonoporation-mediated macromolecule delivery. Inertial cavitation dose in most studies has been employed to evaluate the delivery efficiency and treatment efficiency. However, the temporal characteristic of the inertial cavitation are also closely related to the bioeffects accompanied by sonoporation. This study aims to propose a method for evaluating the temporal duration of inertialcavitation and determine its relation with the acoustic parameters.

**METHODS** An agarose-gel tissue-mimicking phantom was fabricated to hold 1% SonoVue microbubbles solution. 1-MHz plane transmission transducer and another 7.5-MHzfocused transducer were employed for triggering the cavitation of microbubbles and passively detecting acoustic signal, respectively. Then the signal was amplified and recorded by a high-speed digitizer. After the frequency domains characteristics of the signals were analyzed, the distribution of the broadband energies during the exposure time was obtained (Fig. 1). The full width at the half maximum in the time trace of broadband energy was proposed to determine the temporal duration of the inertial cavitation behavior.

**RESULTS** This study determine: 1) Peak rarefactional pressure (PRP) was negatively correlated with inertial cavitation duration. Inertial cavitation duration rapidly decreased from about 29.31 ms to 1.50 ms for the increasing PRP from 0.5 MPa to 0.7 MPa. But for the PRP above 0.7 MPa, inertial cavitation duration approximately approaches to 1.26 ms, suggested inertial cavitation duration tended to be saturated. 2) Inertial cavitation duration gradually increased from 0.60 ms to 1.23 ms with the increasing pulse duration (PD) from 10 μs to 400 μs, and was positively correlated with the PD. 3) Pulse repetition frequency (PRF) exhibited relatively weakendependent than PD on the inertial cavitation duration (Figs. 2, 3).

**CONCLUSIONS** The proposed inertial cavitation duration could be used to evaluate the kinetics of the inertial cavitation triggered by pulsed ultrasound and microbubbles. The relationship between acoustic parameters (PRP, PD, PRF) and inertial cavitation duration of SonoVue microbubbles were determined. These finding suggested inertial cavitation duration, in combination with the previous inertial cavitation dose, would be the important factors for evaluating cavitation-mediated therapy.

**Fig. 1 (abstract P16).**
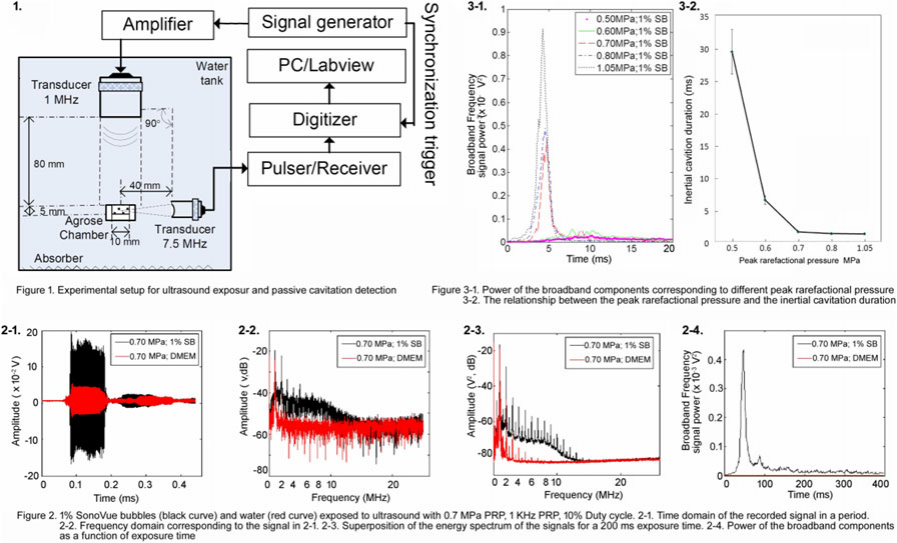
See text for description

**Fig. 1 (abstract P18).**
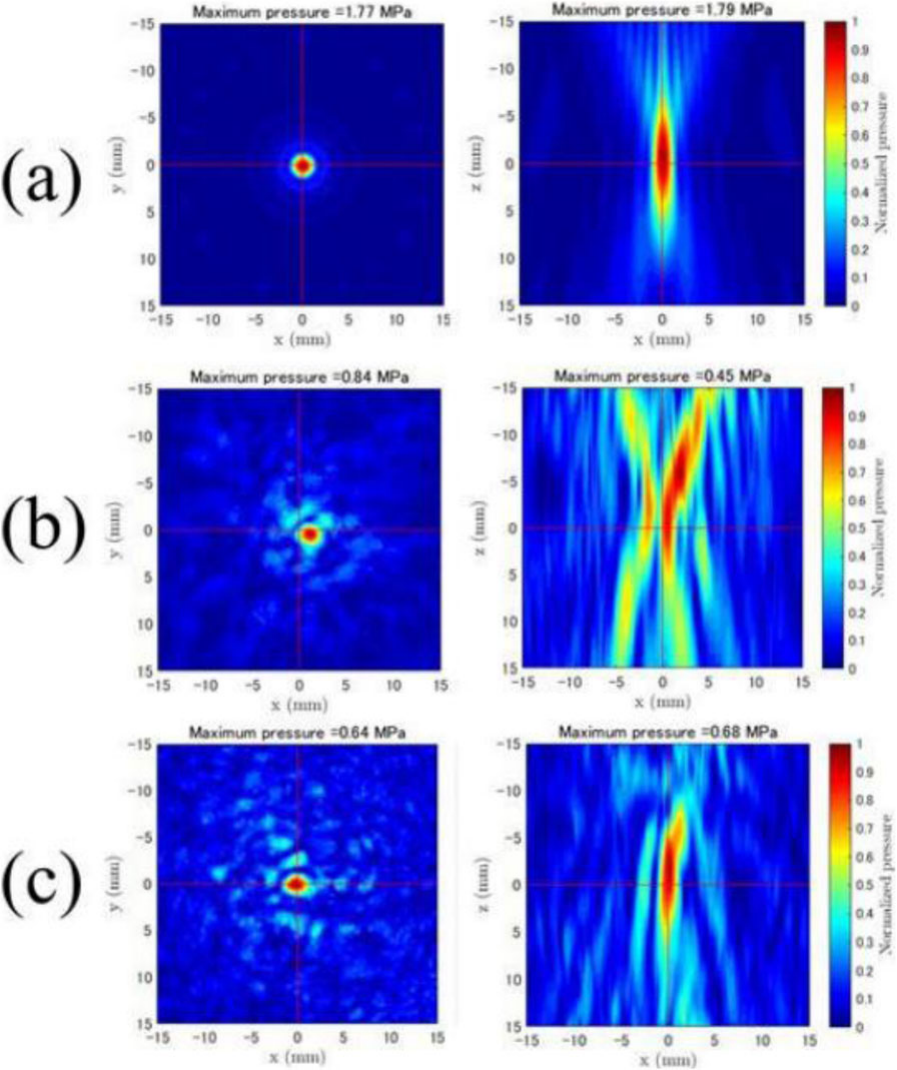
Pressure distribution surrounding target point(a) Water (b) skull without control (c) skull with control

**Fig. 2 (abstract P18).**
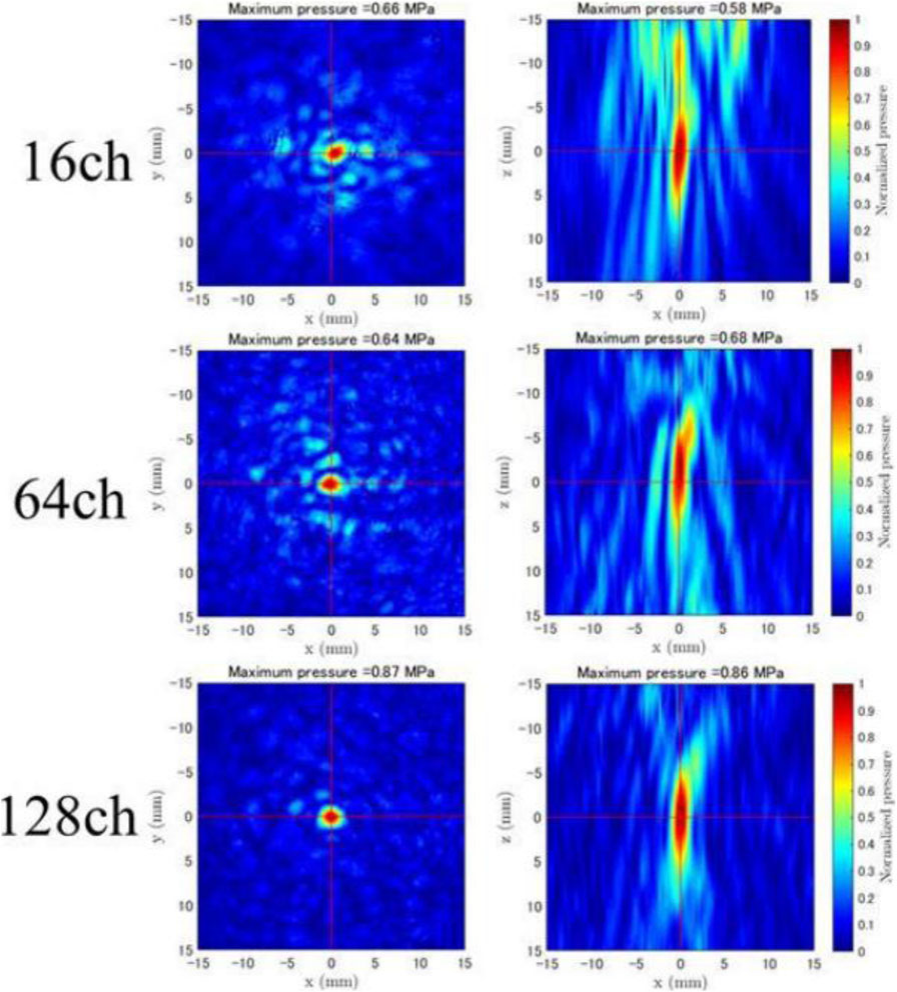
Pressure distribution surrounding target pointof three different number of ch

### P17 Sub-wavelength focal region achieved by a spherical focused ultrasound transducer with open ends in resonator modes

#### Hua Cao^1^, Min He^1^, Zhou Lin^2^, man luo^1^, Guangrong Lei^3^, Xiaobo Gong^3^, Jun Dang^4^, Deping Zeng^1^, Faqi Li^1^, Junru Wu^5^, Dong Zhang^2^, Zhibiao Wang^1^

##### ^1^Chongqing medical university, Chongqing, China; ^2^Institute of Acoustics, Key Laboratory of Modern Acoustics, MOE, Nanjing University, Nanjing, China; ^3^National Engineering Research Center of Ultrasound Medicine, Chongqing, China; ^4^Department of Oncology, 1st Affiliated Hospital of Chongqing Medical University, Chongqing, China; ^5^Physics, School of Engineering, the University of Vermont, Burlington, Vermont, United States

###### **Correspondence:** Hua Cao

**OBJECTIVES** High intensity focused ultrasound (HIFU) has become a new noninvasive surgical modality for cancer treatment, however, the HIFU focusing precision is handicapped by the diffraction limit of the wavelength of a traveling ultrasonic wave. A new HIFU transducer has been developed, which uses standing waves generated in a spherical cavity with open ends and break the diffraction limit to achieve subwavelength focusing region.

**METHODS** In order to describe the acoustic field, a finite element model is developed to numerically study the acoustic field generated from the spherical cavity transducer assembly and the experiment was setup to measure the frequency dependence of the acoustic field generated by this spherical cavity transducer.

**RESULTS** Our theoretical and experimental results demonstrate that in its resonant modes, the focusing zone is smaller while the focusing gain of sound pressure ishigher.

**CONCLUSIONS** These results indicate that the focal zone of the acoustic field inside a spherical cavity with open ends is compressed to a sub-wavelength level while the intensity of sound pressure in the focal region significantly increases.

### P18 Non-invasive therapeutic method for brain disease using TFUS system assisted by numerical simulation

#### Yohei Kobayashi^1^, Takashi Azuma^2, 1^, Tatsuya Umeda^3^, Tomomichi Oya^3^, Masashi Koizumi^3^, Ryo Suzuki^4^, Naoto Yamamura^1^, Kazuo Maruyama^4^, Kazuhiko Seki^3^, Shu Takagi^1^

##### ^1^Bioengineering Dept., The University of Tokyo, Tokyo, Japan; ^2^Faculty of Medicine, The University of Tokyo, Tokyo, Japan; ^3^National Center of Neurology andPsychiatry, Kodiara-shi, Tokyo, Japan; ^4^Teikyo University, Tokyo, Japan

###### **Correspondence:** Yohei Kobayashi

**OBJECTIVES** tFUS (transcranial focused ultrasound) has a great potential for non-invasive therapy for brain disease. The purpose of this research is to apply tFUS with microbubble for BBB opening for DDS and stimulation of red nucleus for motion trigger. BBB opening is the technique to enhance the permeability of blood brain barrier for efficient drug delivery. On the other hand, stimulation of red nucleus which exists in deep area of brain is said to be effective against rehabilitation from cerebralinfarction. For investigating these therapy, we conduct the experiment using macaque monkey which is closer to the human. In either case major issues concerning application of tFUS for brain therapy is focal displacement due to reflection and refraction through the skull. In this research we develop the focal controlling method with array transducer and simulation of ultrasound propagation utilizing CT data of macaque monkey.

**METHODS** Array transducer can control the focal point by adjusting phase of each element. Phase delay of each element can be decided by time reversal method using simulation. At first the sound source is set at the target point at the simulation model constructed by monkey’s CT data and the ultrasound emitted and propagates to the array transducer through the skull. Next the received signals are reversed and emitted from the array transducer to the target, which focus the ultrasound on the target correctly. Simulation solves the basic equation of continuum mechanics by FDTD method. And it analyzes the ultrasound propagation by treating the medium as the mixture of water and bone. In order to model the complicated living body tissues Hounsfield unit of CT images is translated to the volume fraction of bone. The density and sound speed of each voxel is calculated by the volume fraction. It allows to analyze very large scale calculation rapidly.

**RESULTS** To investigate the effect of phase controlling the simulation using actual macaque monkey’s CT data is conducted. Figure 1 is simulation results and each image shows the pressure distribution surrounding the target point. Intensity of pressure is normalized by maximum value of focal point. (a) shows the case of only watermedium and (b), (c) show the result under existence of bone without control and with control respectively. The focusing with control is improved compared to focusing without control. Figure 2 shows the result of 3 different ch number of array transducer. Compared with 16ch, 64ch has better focusing but there is no large difference between 64ch and 128ch.

**CONCLUSIONS** Simulation result suggests that the phase controlling can work effectively. And it is also founded that by comparing the result of 3 different number ofch, 64ch is enough to control the focal point. In the future in order to verify the simulation result we are going to compare it with experimental measurement. Then parameters and modeling method of simulation are reconsidered for the experiment using macaque monkey.

**Fig. 1 (abstract P19).**
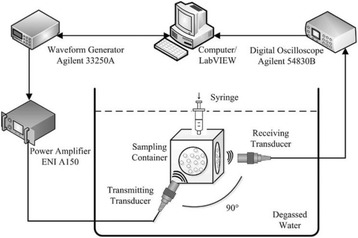
See text for description

**Fig. 1 (abstract P20).**
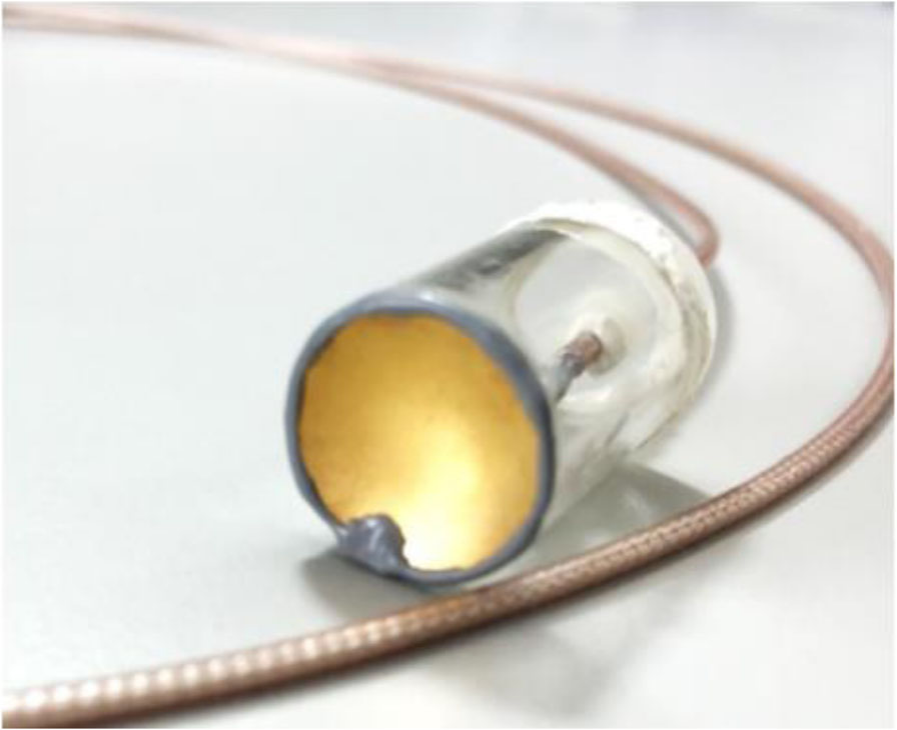
The photo of the 5­MHz focused ultrasonic transducer

### P19 Evaluation of ambient pressure using sub-harmonic response of microbubbles sonicated with chirp pulses

#### Zhiyang Jin, Siyu Liu, Xiasheng Guo, Juan Tu, Dong Zhang

##### Nanjing University, Nanjing, China

###### **Correspondence:** Zhiyang Jin

**OBJECTIVES** The sub-harmonic signal generated by ultrasound contrast agents’ stable cavitation has been proven to be a potentially effective and efficient cue for noninvasive blood pressure measurement. In this work, an improved ambient pressure evaluation method is proposed to enhance the sub-harmonic responses of microbubbles so that a more sensitive and accurate pressure measurement could be achieved.

**METHODS** Chirp signals, namely, linear frequency-modulated signals, are combined with microbubble sub-harmonics in this work. Both simulations and experiments are conducted to illustrate the advantage of chirp excitation in ambient pressure evaluation by comparisons with conventional sinusoidal excitation. Dependence of subharmonic response on chirp parameters, namely, acoustic pressure, central frequency, bandwidth and pulse length, are also studied for optimization of sub-harmonic responses under chirp sonication. All the simulations are based on Marmottant model, supposing microbubbles have a Gaussian size distribution. Commercially availableSonoVue microbubbles (Bracco Diagnostics, Geneva, Switzerland) were used for the experimental measurements and the experimental setup is illustrated in Fig. 1 uploaded.

**RESULTS** Sub-harmonic enhancement by chirp excitation: SonoVue microbubbles are driven by sinusoidal and chirp excitation sharing the same driving pressureamplitude and pulse length at Pov = 0 kPa (specifically, ambient pressure of 1 atm). The sinusoidal wave frequency and the central frequency of the chirp excitation werefs= fc = 3.5 MHz, and the chirp signal bandwidth was Δf = 0.4 MHz. A significant increase of 5.1 dB in the sub-harmonic component can be observed in the chirp excitation case. As the ambient pressure increases, the measured sub-harmonic responses induced by chirp excitation reduce more violently than sinusoidal case, providing better sensitivity in ambient pressure measurement. All the experimental results agree well with the simulations. Dependence of sub-harmonic response on driving acoustic pressure: In a similar setup with the former one, the amplitudes of sub-harmonic responses grow with increasing acoustic pressure for both chirp and sinusoidal excitations and this growth would reach a saturation level when acoustic pressure exceeds a specific threshold.The experimental results agree with simulations when the driving pressure is less than 300 kPa, but the saturation amplitudes of sub-harmonic responses for both excitations after that are much lower than the simulated results. When the driving pressure is greater than 100 kPa and less than 300 kPa, the sub-harmonic amplitudes excited by chirp signals are always 5-10 dB higher than those excited by sinusoidal signals. The stable cavitation threshold for chirp excitation is also observed to be much lower than for sinusoidal excitation. Optimization: As the bandwidth of chirp signal increase, the sub-harmonic amplitudes changes more greatly when the ambient pressure changes. This indicates that the wider bandwidth could offer a better sensitivity in ambient pressure evaluation by exciting a wider size range of microbubbles. However, bandwidths over 22.8% were not investigated because of the overlap effect wider bandwidths may bring. The studies on chirp pulse length indicates that the measured sub-harmonic responses increase with longer pulse length. The inherent reason for this behavior is that each individual microbubble may take a few cycles to reach a steady state when a strong non-linear effect is observed.

**CONCLUSIONS** The sub-harmonic waves produced by stable cavitation of bubbles provides the feasibility of enhanced blood pressure measurement. Due the non-negligible size distribution of commercial ultrasound contrast agent microbubbles, chirp signals rather than sinusoidal signals are applied in this article to excite a wider size range of microbubbles. Chirp ultrasound has been theoretically and experimentally verified to effectively enhance both the amplitude of sub-harmonic response and its sensitivity to ambient pressure, thus it overweighs conventional sinusoidal signals in pressure measurement. Wider bandwidths and longer pulse lengths for chirp excitation also prove to realize an optimized pressure evaluation routine. Further potential development of the proposed method will require enhancement of bubble dynamics models and improved microbubble fabrication techniques so that this method could work better for non-invasive blood pressure measurement applications.

**Fig. 2 (abstract P20).**
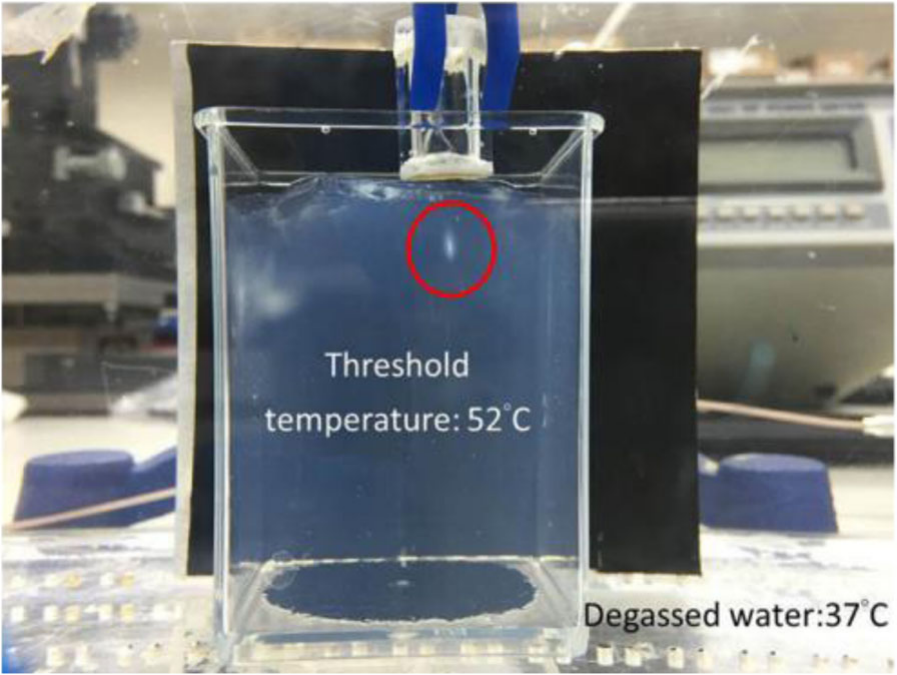
A white lesion was induced in the thermal­sensitive hydrogel phantom by the developed transducer

### P20 Development of a focused ultrasound device for skin ablation

#### Meng-Hung Tsai^2^, Li-Chen Chiu^1^, Win-Li Lin^2^, Gin-Shin Chen^1^

##### ^1^Institute of Biomedical Engineering and Nanomedicine, National Health Research Institutes; ^2^Institute of Biomedical Engineering, NationalTaiwan University

###### **Correspondence:** Meng-Hung Tsai

**OBJECTIVES** Focused ultrasound can concentrate acoustic power on the target tissues like subcutaneous fat and superficial muscular aponeurotic system to generate a steep temperature elevation, leading to collagen denaturation, contraction and remodeling. In the study, a single-element focused ultrasound transducer integrated with an automatic motion system was developed for multi-point ablation in the skin.

**METHODS** The transducer was made of 1-3 piezocomposite materials with a diameter of 10 mm and a radius of curvature of 17 mm (Fig. 1). The operation frequency was 5MHz. The electroacoustic conversion efficiency and focal zone were measured after electrical impedance matching. The effectiveness of the device was evaluated by the ablation experiments of phantom, ex-vivo and *in vivo* rat model.

**RESULTS** The efficiency of the transducer was 43.9±2.6%. The focal depth and focal width were 7.2 and 0.7 mm, respectively. With the ultrasonic parameters of electrical power of 10 W for 1 s, the lesions were formed in the 52°C thermo-sensitive hydrogel phantom (Fig. 2). Ex vivo and *in vivo* studies showed that the transducer could produce a hot spot to noninvasively cause superficial skin necrosis as the electrical power and time of ultrasonic sonication were in a range of 6-9 W and 2 s.

**CONCLUSIONS** A relatively high-frequency focused ultrasound device has been developed for skin ablation. In vitro and animal studies have verified the efficacy ofthe ultrasonic device (Fig. 3).

**Fig. 3 (abstract P20).**
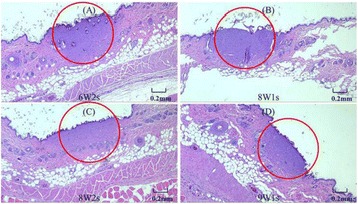
The H&E staining of the rat skin tissue after ultrasonic sonications

**Fig. 1 (abstract P22).**
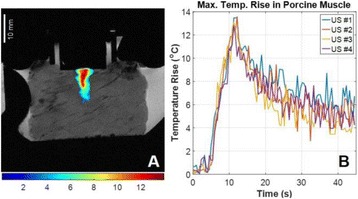
(A) Reconstructed temperature maps based on PRF shift superimposed with high resolution gem image; (B) Traces of the maximum temperature rise measured in porcine muscle from 4 subsequent ultrasound exposures

**Fig. 2 (abstract P22).**
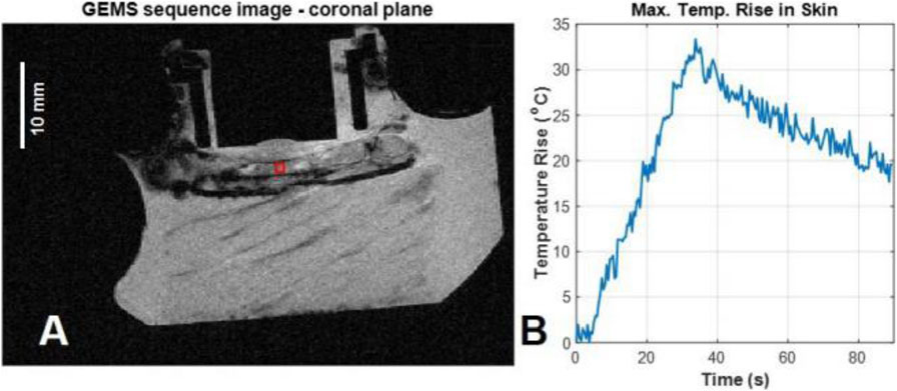
(A) MR image acquired using gems sequence. Post­mortem full thickness skin is roughly 1 cm thick overlaid on top of a piece of porcine muscle which helpedavoiding reflection from the bottom of the chamber. (B) Trace of the maximum temperature rise in the skin from a 30 second ultrasound exposure

### P21 Low intensity pulsed ultrasound stimulates hair follicle cells in 3D culture

#### Noboru Sasaki, Mitsuyoshi Takiguchi

##### Hokkaido University, Sapporo, Japan

###### **Correspondence:** Noboru Sasaki

**OBJECTIVES** Low intensity pulsed ultrasound (LIPUS) has been known to activate protein synthesis, DNA synthesis and cell proliferation in several different types of cells. This proof of concept study evaluated whether LIPUS stimulates proliferation of hair follicle cells in 3D culture.

**METHODS** The 3D culture was composed of three layers. In the top layer, Human Follicle Dermal Papilla cells (HFDPC; PromoCell) were embedded into Matrigel (Corning). The middle layer consisted of only Matrigel. In the bottom layer, Human Hair Outer Root Sheath cells (HHORSC; ScienCell) were embedded into Matigel.Low intensity pulsed ultrasound was exposed to cells from above the 3D culture. Ultrasound parameters are as follows; 1 MHz center frequency, 500 pulses, 1 kHz PRF (i.e. the duty factor was 50%), Isata 90 mW/cm2. After ultrasound exposure, cells in the 3D culture were stained by Calcein-AM and observed by a fluorescent microscopy.

**RESULTS** Both HFDPC and HHORSC were increased by single exposure of LIPUS. Moreover, HFDPC grew upward, i.e. from the bottom layer to the top layer.

**CONCLUSIONS** This study showed the feasibility of LIPUS for stimulating the proliferation of hair follicle cells. Further in depth study may assess mechanisms of thestimulation and optimize ultrasound parameters.

### P22 MR thermometry of a novel focused ultrasound application in post-mortem skin

#### Ziqi Wu^1^, Luis Hernandez-Garcia^2^

##### ^1^Access Business Group, Grand Rapids, Michigan, USA; ^2^Biomedical Engineering, University of Michigan, Ann Arbor, Michigan, USA

**OBJECTIVES** Intense focused ultrasound has been introduced in past years for non-invasive anti-aging facial treatment. By creating micro-necrosis in dermalor superficial muscular aponeurotic system (SMAS), wound healing process can be triggered followed by promotion of fibroblasts activity and collagenproduction. Recent research showed that repeated mild application of focused ultrasound energy in the skin with lower acoustic intensity also provided clinical benefits. Temperature rise generated inside the skin using this technique is hypothesized as the mechanism of action, and the estimation of temperature within the targeted region is important for the application efficacy and safety. In this study, we demonstrated that MR thermometry can be used to measure the 2Dtemperature changes generated by a novel focused ultrasound skin applicator with fine spatiotemporal resolutions in post-mortem skin.

**METHODS** A MR compatible prototype transducer was constructed (Access Business Group, USA) using a partial cylindrical ceramic element and a plastic waveguide. The focal distance was confirmed to be 4 mm in water by Schlieren imaging and the -6dB focal region was measured to be 1 mm in width and 20mm in length using a scanning hydrophone system. The maximum rarefraction pressure was less than 1 MPa while the spatial-peak pulse-average intensity was less than 30 W/cm2. The transducer was driven with a function generator and an amplifier at a center frequency of 4.5 MHz with 2.8 W average acoustic power. Pulsed ultrasound (400 ms) was generated with a duty cycle of 89% and total exposure time varied from 7 to 30 seconds. A 7T MR scanner (AgilentTechnologies, Walnut Creek, CA) was used to image porcine samples for repeatability of MR thermometry and followed by imaging the post-mortem skin. Highresolution MR images were acquired with multislice gradient-echo sequence (TR/TE = 20/4 ms, flip angle = 20, voxel size = 0.47 x 0.47 x 2 mm) at the beginning and end of the experiment while gradient echo EPI sequence (TR/TE = 400/11.32 ms, triple shots, voxel size = 0.42 x 0.83 x 1 mm) were performed before, during, and after ultrasound exposure. The ultrasound transducer and the MRI scanner were synchronized *via* TTL pulses, such that the EPI images were collected between ultrasound bursts. Spatiotemporal temperature changes were computed using proton resonant frequency shift relationship from the MR phase images. Finally, skin samples were stored in 10% formalin, fixed in paraffin, sliced, and stained with H&E and masson’s trichrome to investigate thermal damage on skin cells.

**RESULTS** The reconstructed temperature images in the coronal plane across the ultrasound beam width showed the best image quality (Fig. 1) whereas the sagittal plane images along the beam length were poor due to magnetic field inhomogeneity. In the porcine muscle, maximum temperature rise of 13.2 ± 0.3 °Coccurred inside the focal region and the MR thermometry results were repeatable with four subsequent ultrasound exposures. Transient changes were also observed on MR magnitude images. In the post mortem skin, 8 seconds ultrasound exposure generated temperature rise of 17.9 °C whereas 30 seconds exposure caused 33.3 °C temperature increase (Fig. 2). The maximum temperature rise occurred at 2 mm from the transducer surface into the dermis. No permanent histological cell damage was seen for shorter ultrasound exposure whereas necrosis was observed with longer ultrasound exposure.

**CONCLUSIONS** Using gradient echo EPI sequence, MR can measure the spatiotemporal temperature changes induced by a novel ultrasound skin applicator inpost-mortem skin and provide potential safety temperature or thermal dose threshold for focused ultrasound dermatological applications. (A) Reconstructed temperature maps based on PRF shift superimposed with high resolution gem image; (B) Traces of the maximum temperature.

**Fig. 1 (abstract P31).**
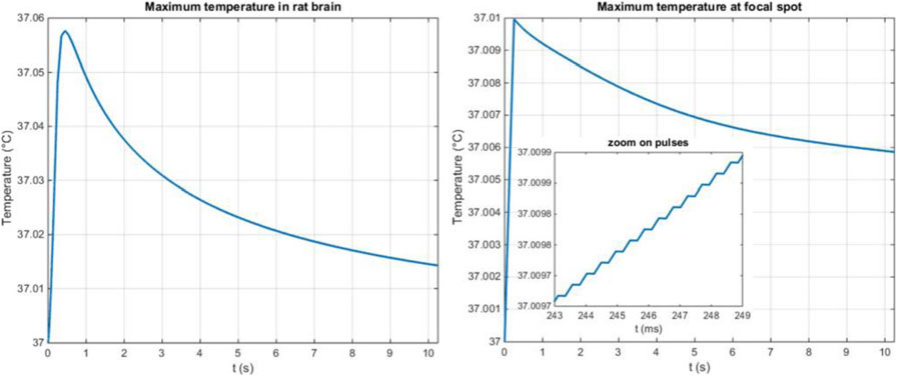
Maximum temperature in brain, with zoom on individual bursts (left) and at focal spot (right) in study [3]

**Fig. 2 (abstract P31).**
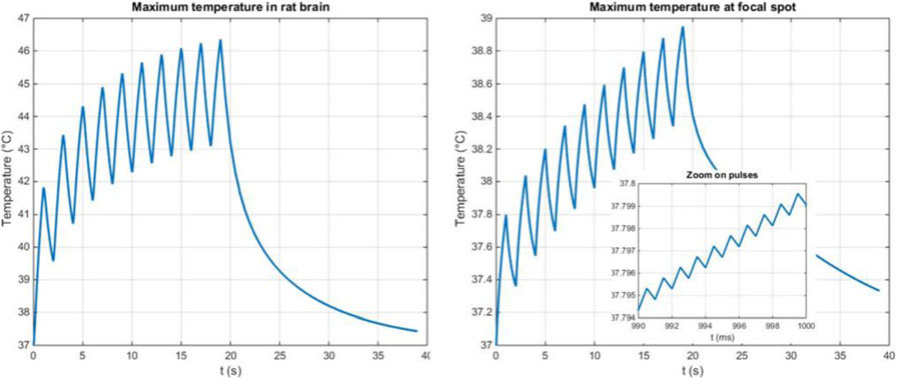
Maximum temperature in brain, with zoom on individual bursts (left) and at focal spot (right) in study [7]

### P23 Ultrasound-mediated transdermal delivery of hyaluronic acid into skin

#### Yi Yun

##### VITA-Sound Tech, USA

Transdermal drug delivery (TDD) can effectively bypass the first-pass effect, which can be valuable in cosmetic industry. In our work, ultrasound-facilitated TDD on fresh porcine skin was studied in various conditions of acoustic parameters. The delivery of fluorescent nanoparticles and high molecular weight hyaluronic acid (HA) in the skin samples was observed by laser confocal microscopy and ultraviolet spectrometry, respectively. The results showed that, with the application of ultrasound exposures, the permeability of the skin to these markers (e.g., their penetration depth and concentration) could be obviously raised above its passive diffusion permeability. Moreover, ultrasound-facilitated TDD was also tested with/without the presence of ultrasound contrast agents (UCAs). When the ultrasound was applied without UCAs, low ultrasound frequency will give better drug delivery effect than high frequency, but the penetration depth was in a less level around 200 μm. However, with the help of ultrasound-induced microbubble cavitation effect, both the penetration depth and concentration in the skin were significantly enhanced even more. The best ultrasound-facilitated TDD could be achieved with a drug penetration depth of over 600 μm, and the penetration concentrations of fluorescent nanoparticles and HA increased up to about 4-5 folds. In order to get better understanding of ultrasound-facilitated TDD, scanning electron microscopy was used to examine the surface morphology of skin samples, which showed that the skin structure changed greatly under the treatment of ultrasound and UCA. The present work suggests that, for TDD applications (e.g., nanoparticle drug carriers, transdermal patches and cosmetics) in cosmetic industry, protocols and methods presented had shown us the potentially attractive application for moisture and treatment of Skin Deapth.

### P24 Comparative study on ultrasonic monitoring of pHIFU and cHIFU peripheral ablation mode

#### Wen Jing

##### Chongqing Medical University, Chongqing, China

**OBJECTIVES** To investigate the feasibility of Ultrasonic monitoring on PHIFU and CHIFU peripheral ablation mode.

**METHODS** 60 cases ox-liver tissues were divided into group A (PHIFU, DC=15%, n=30) and group B(CHIFU,DC=100%,n=30).Under the guidance of B mode ultrasound, each group was performed with peripheral ablation, during and after the ablation, capture images of every single line and layer related to the target tissue, and then analyze the gray scale change. After the whole tissue peripheral ablation, slice the target and observe its damage situation.

**RESULTS** In the course of peripheral ablation, both groups were seen instant echo enhancement in the irradiated areas, with time varying, the Hyperecho were finally replaced by hypoecho in group A and the tissues were presented liquefaction necrosis, while linear Hyperecho were still found in group B and tissues were presented coagulation necrosis. After peripheral ablation, a cyclic hypo-echo were observed around the target and the gray scale of the internally areas were slightly changed and tissue observing nearly showed damaged. While in group B, the instant Ultrasonic monitoring images were not agreed with the form of the tissue damage situation for the entire target were presented time depended strong echo change, and the areas where there is actually coagulation necrosis were partial hypoecho or medium echo.

**CONCLUSIONS** The tissue damage character, ultrasonogram and gray scale change were all varied between PHIFU and CHIFU peripheral ablation mode. It is possible to apply Ultrasonic monitoring while performing peripheral ablation mode, but there is still limitation.

### P25 Experimental study of the effect of the target blood vessels angled with acoustic axis on the surface ablation of pHIFU

#### Yang S. Ying, Zou Jianzhong

##### Imaging and Nuclear Medicine, Institute of biomedical engineering, Yuzhong district, Chongqing, China

###### **Correspondence:** Yang S. Ying

**OBJECTIVES** To explore the effect of the target blood vessels angled with acoustic axis on the formation of the around coagulation necrosis under the model of the surface ablation of PHIFU with the same conditions of irradiation dose, To adjust the HIFU treatment model has certain guiding significance.

**METHODS** Embedded the rabbit thoracic aorta (diameter was 4.25±0.79 mm) into egg white body model and divided into 3 groups by the blood vessels Angled 0 °,45°,90 °with acoustic axis.The egg white body model were exposed to pHIFU with 200-300 w acoustic power,100Hz pulse recurrence frequency(PRF),50% dutycycles.The surface ablation size is 30 * 30 * 30 mm3,line scanning, Linear speed: 3 mm/s;Line length: 30 mm; line scanning time interval: 1.5 min;Layer spacing 2 mmand 16 layers in total.In the process of irradiation ultrasonographic observations and temperature measurement; After the irradiation, cut body model to observe theirradiation damage.

**RESULTS** In the process of Irradiation, near the acoustic source side of the target blood vessels Angled with acoustic axis, the temperature curve shown as: increase fast, long duration and falling fast and far from the acoustic source side one is relatively slow, short duration, decreased slower. And the ultrasonographic performance that far from the acoustic source side is low echo immediately, gradually enhanced, after 3 min the gray level change is not obvious and near the acoustic source side is immediatestrong echo, gradually weakened and after 3 min the gray does not change significantly.After irradiation, cut down the egg white body models,observed that far from theacoustic source side of the blood vessel Angled 45 °, 90° with acoustic axis were no white coagulation necrosis formed. And the 0°one coagulation necrosis around the blood vessels was narrowed.

**CONCLUSIONS** Adopted the surface ablation model, the existence of the target blood vessels angled with acoustic axis can affects its surrounding coagulation necrosisformation. The target blood vessels Angled 0 °with acoustic axis can narrowed the coagulation necrosis arround the vessels. And far from the acoustic source side of theblood vessels Angled 45 °,90 °could not formed the coagulation necrosis. And in the process of Irradiation, the change of the gray-scale ultrasonography could judge thedamage formation around the blood vessels to a certain extent.

### P26 Concentration of MSNC-PFP influence surface models in high-intensity focused ultrasound ablation of ex vitro bovine liver

#### Ding Xiaoya, Dazhao Ma, Wen Jing, Qi Wang, Jianzhong Zou

##### Chongqing medical university, Chongqing, China

###### **Correspondence:** Ding Xiaoya

**OBJECTIVES** To Discusse the influence of perfluoropentane drops-encapsulated mesoporous silica nanocapsules (MSNc-PFP) injected into the melting region withthe surface ablation of HIFU *in vitro* bovine liver.

**METHODS** Ox-liver tissues were randomly divided into 5 groups: PBS blank control group, 0.25 mg/ml group, 0.5 mg/ml group, 1 mg/ml group, 2 mg/ml group according to the concentration of MSNc-PFP, melting line isometric injection five points, measured 100 uL. Each group was surface ablationed that the edge internal were not included with the same dose of HIFU energy after rejection.

**RESULTS** 1 mg/ml group, 2 mg/ml group, the high-level echo of synergistic agent in the injection area was receding after injection; After HIFU irradiation, regional gray values were heighten and the scope was broadening changing with the concentration of synergist increased. Each group can form a complete coagulation necrosisexcept the control group. And the higher the synergistic agent concentration used, the width and the ablation range of oagulation necrosis were greater. 2 mg/ml group showed obvious thermal damage peripheral ablation region.

**CONCLUSIONS** Concentration of 1 mg/ml of MSNc-PFP had a good effect on the surface models of synergistic HIFU ablation.

### P27 Effects of subatmospheric pressure and dissolved oxygen concentration on the generation of lesions in *ex vivo* bovine livers by HIFU

#### Min He, Zhiqiang Zhong, Xiaobo Gong, Faqi Li, Deping Zeng, Zhibiao Wang

##### College of Biomedical Engineering, Chongqing Medical University, Chongqing, China

###### **Correspondence:** Min He

**OBJECTIVES** This study was aim to investigate the effects of subatmospheric pressure and dissolved oxygen concentration on the morphology and size of lesionsgenerated by HIFU in *ex vivo* bovine liver.

**METHODS** All HIFU experiments were conducted in a stainless chamber fulfilled with degassed water. A 1MHz HIFU transducer was used to generate the US exposureof 11700W/cm2 which was acutely cavitation under atmospheric pressure. The dissolved oxygen concentration (DOC) of degassed water were divided into three groups: 1.0 mg/L, 1.5 mg/L and 2.0 mg/L respectively. The *ex vivo* bovine livers were exposing 2 seconds per time under two ambient pressure of atmospheric pressure and subatmospheric pressure. B mode US monitoring the strong echo signal before and after HIFU exposing. A passive cavitation test system (PCD) test the acousticcavitation signal in the process of exposing.

**RESULTS** 1. The broadband noise of atmospheric pressure and subatmospheric pressure under the same dissolved oxygen concentration shows that cavitation was weaker under subatmospheric pressure than that under atmospheric pressure.2. The variation of difference of gray level value between before and after HIFU exposing on the B-scan image is increasing with the increase of dissolved oxygen concentration. Under atmospheric pressure, the difference of gray level value is larger than that under subatmopheric pressure.3. Under atmospheric pressure, the lesions are from tadpole shape to elliptical as the dissolved oxygen concentration decreased. But under subatmopheric pressure, thelesions are all elliptical. The size of lesions is increasing with the increase of dissolved oxygen concentration. Under the same dissolved oxygen concentration, the size oflesions is larger under atmospheric pressure than that of subatmospheric pressure.

**CONCLUSIONS** This study investigated the effects of subatmospheric pressure and dissolved oxygen concentration on the generation of lesions in *ex vivo* bovine liversby HIFU. The followings were clarified in the experiment.1. The reduce of dissolved oxygen concentration could decrease the volume of lesions generated by HIFU.2. Subatmospheric pressure could restrain the cavitation, thus reshape the lesions to elliptical and smaller the size of lesion in focus.

### P28 The effect of phased-hifu with discontinuous operating mode on coagulative necrosis region

#### Xiongfei Qu, Guofeng Shen, Nan Wu, Yazhu Chen

##### School of Biomedical Engineering, Shanghai Jiao Tong University, Shanghai, China

###### **Correspondence:** Xiongfei Qu

**OBJECTIVES** In the previous experiments, we noticed that Phased-HIFU with discontinuous operating mode (eg. 2s heating following with a 1s cooling, and repeating) produced shorter and thicker coagulative necrosis region in *ex vivo* porcine muscle, compared with slender spindle like region in continuous operating mode. The aim of this study was to demonstrate the mechanism and influence of this method on tissue ablation.

**METHODS** In this study, we investigated the influence of discontinuous operating mode on tissue ablation. Three different treatment procedures in 4 repeat cycle (each for 2.35s) were simulated using a DFDT method: (1) Continuous heating with constant 200 watts of acoustic power; (2)50% duty ratio (1.175s heating in a cycle) heating with 200 watts of acoustic power; (3)50% duty ratio heating with 400 watts of acoustic power. Then the *ex vivo* porcine muscle and tissue-mimicking phantom (NIPAMbased hydrogel phantom with cloud point temperatures at 52°C) heating experiments were performed in procedures (1) and (3), to investigate the shape of coagulative necrosis region and temperature above 52°C, respectively.

**RESULTS** The simulation showed that the short rod-like 240EM region of the discontinuous operating mode was shorter, thicker and larger, compared with slender spindle like 240EM region of continuous operating mode. However, the gradient of thermal dose in 240EM region boundary of discontinuous operating mode was much smaller, which may cause the instability of ablation border. These two simulation results were observed in the *ex vivo* porcine muscle and tissue-mimicking phantom experiments.

**CONCLUSIONS** The discontinuous operating mode can produce a larger short rod-like coagulative necrosis region, therefore may reduce the number of treatment shotsand improve the efficiency of treatment. However, it reduces the gradient of thermal dose in ablation boundary, therefore may reduce the stability of coagulative necrosis region.

### P29 Short-term and long-term efficacy of ultrasound ablation for diffuse and focal adenomyosis

#### Yujie Feng, Jinyun Chen

##### College of Biomedical Engineering, Chongqing Medical University, Chongqing, China

###### **Correspondence:** Yujie Feng

**OBJECTIVES** To compare the short-term and long-term efficacy of HIFU in treatment of diffuse and focal adenomyosis.

**METHODS** A total of 308 patients with adenomyosis who accepted HIFU ablation were collected. According to preprocedural MRI, the patients were divided into diffuse and focal group. The non-perfused volume ratio (NPVR) and the incidence of ablation were calculated. Preprocedural and postprocedural situation of dysmenorrhea and menorrhagia were evaluated.

**RESULTS** Effective follow-up were performed in 297 cases. The follow-up time was 1—50 months. There were 177 cases in diffuse group, while 120 cases in focalgroup. The incidence of ablation among 297 patients was 99.33% (295/297). The NPVR of diffuse and focal group were ([26.00±13.36] %) and ([44.32±19.93] %), respectively. The dysmenorrhea and menorrhagia score of post-procedure were significantly lower than those of pre-procedure in two groups (both P<0.05). The total remission rate of dysmenorrhea in 3, 6, 12, 24 and 36 months after ablation were 92.96% (264/284), 86.18% (237/275), 73.51% (197/268),60.71%(136/224), 46.83% (59/126), respectively. The remission rate of dysmenorrhea of focal group was higher than that of diffuse group in each follow-up period, while significant differences were observed in 6, 24 and 36 months (P<0.05). The total remission rate of menorrhagia in 3, 6, 12, 24 and 36 months after ablationwere 87.38% (187/214), 83.09% (172/207), 68.63% (140/204), 63.64% (105/165), 45.92% (45/98), respectively. The remission rate of menorrhagia of focal group washigher than that of diffuse group in each follow-up period, while no significant difference was observed between two groups (all P>0.05).

**CONCLUSIONS** HIFU is significant effective for adenomyosis, the short-term efficacy of focal and diffuse type are familiar, while the long-term efficacy of focal typeis better than that of diffuse type.

### P30 Ultrasound-guided versus mr-guided high intensity focused ultrasound for ablation of uterine fibroids by ultrasonic contrast agent: treatment efficacy, safety and efficiency

#### Yi Wang, Yonghua Xu

##### Chongqing Medical University, The Institute of Ultrasound Engineering in Medicine, Chongqing, China

###### **Correspondence:** Yi Wang

**OBJECTIVES** The time efficiency and sonication energy efficiency of treatment and safety were compared between MR-guided High-Intensity Focused Ultrasound (MRgHIFU) and Ultrasound-guided High Intensity Focused Ultrasound (USgHIFU) for complete ablation of T2 hypointense fibroids.

**METHODS** The treatment data and sonication parameters from 13 uterine fibroids in 10 patients treated with MRgHIFU and 28 uterine fibroids in 22 patients treated with USgHIFU were analyzed. All of the pre-treatment fibroids were hypointense signal in T2 weighted imaging and completely ablated by using MRgHIFU orUSgHIFU at one session treatment. The volume of the treated fibroid and the non-perfused volume (NPV) was calculated in contrast enhance MRI (CE-MRI), andcomplete ablation of fibroids is defined as non-perfusion region covering all volume of the treated fibroid immediately following the procedure. The treatment and sonication time, the EEF and NPV ratios were compared between MRgHIFU and USgHIFU, while the adverse events and complications were also assessed.

**RESULTS** The percentage rates of the completely ablated fibroids in the MRgHIFU and USgHIFU were 29.5% and 41.2%, respectively. The treatment time was174.5±42.2 minutes and 114.4±39.2 minutes, the sonication time was 24.7±9.0 minutes and 19.4±6.8 minutes, the sonication power was 310.2±62.5W and 391.6±16.6W, the sonication energy was 483.0±248.2 KJ and 463.2±156.4KJ, EEF was 5.1±3.0 KJ/cm3 and 6.8±5.2 KJ/cm3, and the mean treatment speed was 42.2±25.6 cm3/h and70.9±41.9 cm3/h in the MRgHIFU and USgHIFU treatment for complete ablation of fibroids, respectively. There was a negative linear correlation between the EEF andthe NPV of fibroids, and a positive linear correlation between the treatment speed and the NPV of fibroids in the both groups (P<0.05). There was a positive linear correlation between the sonication intensity and the NPV of fibroids in the USgHIFU group (P<0.05) and no correlation in the MRgHIFU group (P>0.05). There was no severe adverse event and major complication in both groups after treatment.

**CONCLUSIONS** MRgHIFU and USgHIFU both are feasible, safe, and effective with the equivalent energy efficiency for complete ablation of T2 hypointense fibroids;USgHIFU was superior to MRgHIFU in the time efficiency.

### P31 Estimation of thermal rise during ultrasonic neurostimulation in rodents: retrospective analysis of five recent studies

#### Charlotte Constans, Mickael Tanter, Jean-Francois Aubry

##### Institut Langevin, Paris, France

###### **Correspondence:** Charlotte Constans

**OBJECTIVES** The first ultrasonic neuromodulation studies were conducted with rather high intensity [1] and thermal effects were assumed to be the main cause [2]. More recently, multiple groups have reported successful low intensity focused ultrasound (LIFU) neurostimulation on rodents: movement elicitations [3] [4] [5] [7] or reduction of anesthesia time [6]. Given the low intensities used in most of them, mechanical effects are more prone to induce neuromodulation [4]. The mechanism of neuromodulation is still not fully understood, and a more thorough study of the thermal and mechanical effects is necessary to optimize the parameters for clinical applications. We simulated the thermal rise in 5 rodent studies in order to evaluate its potential impact.

**METHODS** The acoustic propagation of focused ultrasound was simulated in an entire rat head in order to investigate the pressure amplitude and spatial distribution. The simulations were performed with k-Wave [6], a k-space pseudospectral method-based solver. 3D maps of the skull, brain and tissues were extracted from a rat microcomputed tomography scan. Brain and tissues were assumed to have the same sound-speed and density as water, and the transducer was modeled according to each study’s materials. Absorption was taken into account in the skull (2.7dB/cm/MHz) and in the brain (0.21dB/cm/MHz) with a 1.18 power law of frequency. Ultrasound propagate in a cone filled with water before entering the rat head, the geometrical focal point being located about 7mm deep from the surface, inside the brain. For each study, we calculated the pressure at focal spot in water based on each study materials and methods. The simulations were first performed in water and compared to these extracted amplitudes. The peak negative pressure in the rat head was extracted from the simulation and thus takes into account reflections and absorption effects. The thermal code is based on the bio-heat equation without perfusion and metabolic processes. The thermal dose unit is CEM (cumulative equivalent minutes at 43°C).

**RESULTS** Parameters and results in brain and at the focal spot for all the studies are listed in Table 1. Temperature rise estimated for [3] and [7] are plotted on Figs. 1and 2 respectively for the most heated point in the brain (left) and at the focal spot (right). In study [3], the thermal does not exceed 3.3E-4 CEM in the skull, brain and skin. In study [7], TD reaches 2800 CEM in the skull, 60 CEM in the brain (close to thebone) and 50 CEM in the skin. The thermal dose in the skin is significant but not high enough to induce skin burns.

**CONCLUSIONS** Our retrospective analysis shows thermal rise ranging from 0.002°C to 9.3°C in the brain. For studies [3-6], corresponding to a frequency range of320kHz to 5MHz and a total sonication time ranging from 80ms to 20min, the maximum temperature elevation in the rodent brain is lower than 0.1°C. Sensitivity to temperature changes was found with a coefficient of 1.1 impulses/s/°C in some neurons [9]. Thus, in the case of study [7], the thermal rise cannot be neglected as apossible cause of neuromodulation.

**Acknowledgements**

This work was supported by the Bettencourt Schueller Foundation and the "Agence Nationale de la Recherche" under the program “Future Investments” with the reference ANR-10-EQPX-15.

**References**

[1] Fry et al. "Production of reversible changes in the central nervous system by ultrasound." Science (1958)

[2] Lele et al. "Effects of focused ultrasonic radiation on peripheral nerve, with observations on local heating." Experimental Neurology (1963)

[3] Younan et al. "Influence of the pressure field distribution in transcranial ultrasonic neurostimulation." Medical physics (2013)

[4] Ye et al. "Frequency Dependence of Ultrasound Neurostimulation in the Mouse Brain." Ultrasound in medicine & biology (2016)

[5] Li et al. "Improved Anatomical Specificity of Non-invasive Neuro-stimulation by High Frequency (5 MHz) Ultrasound." Scientific reports (2016).

[6] Yoo et al. "Transcranial focused ultrasound to the thalamus alters anesthesia time in rats." Neuroreport (2011)

[7] Kamimura et al. "Focused ultrasound neuromodulation of cortical and subcortical brain structures using 1.9 MHz." Medical Physics (2016)

[8] B. Cox et al, k-space propagation models for acoustically heterogeneous media: Application to biomedical photoacoustics, J. Acoust. Soc. Am., 2007.

[9] Burgoon et al. "Temperature-sensitive properties of rat suprachiasmatic nucleus neurons." American Journal of Physiology-Regulatory, Integrative and ComparativePhysiology (2001)

**Table 1 (abstract P31).**
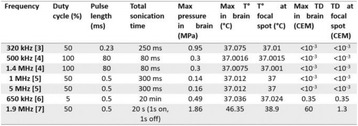
See text for description

**Fig. 1 (abstract P32).**
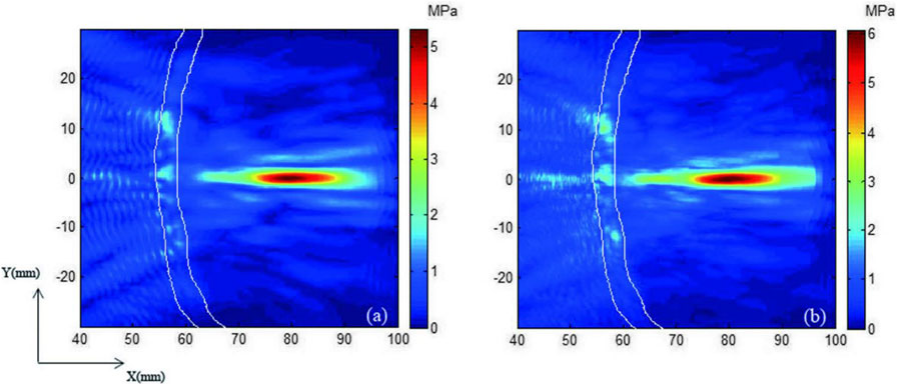
The acoustic pressure distribution (temporal bone window, 0.6 MHz, I_0_ =4.0 Wcm^­2^, t =16 s). (a) without and (b) with standing wave suppression

**Fig. 2 (abstract P32).**
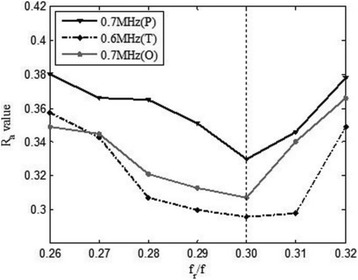
Plot of Rα against fr./f (temporal scales area at 0.6MHz, occipital area and parietal area at 0.7MHz)

### P32 Numerical simulation of the effect of phase transformation on standing waves and transcranial focusing in HIFU

#### Miaomiao Zeng^1^, Shihui Chang^1^, Rui Cao^2^, Xiqi Jian^1^

##### ^1^Biomedical engineering, Tianjin Medical University, Tianjin, China; ^2^Tianjin University of Science and Technology, Tianjin, China

###### **Correspondence:** Miaomiao Zeng

**OBJECTIVES** Reflections induced by heterogeneous structures and large differences in acoustic impedance between the skull and perienchyma result in undesired standing waves that cause energy loss in the treatment area in high-intensity focused ultrasound (HIFU) transcranial treatment and deposition of excess energy in healthy tissue. The goal of this work is to address these issues.

**METHODS** The simulations performed in this paper were based on the three dimensional finite difference time domain (FDTD) simulation of acoustic propagation and thermal behavior through the bone windows. The material properties of the skull were derived from 3D reconstructions of high-resolution computed tomography (CT) scans of selected patients. Phase transformation methods were used to reduce the standing wave by randomly changing the phase in time segments.

**RESULTS** The intensity of the standing wave decreased (Fig. 1). Meanwhile, the sound pressure rose and the rate of temperature rise at focal region increased when using phase transformation. Different bone windows exhibit different optimum excitation frequencies, in the range of 0.6-0.8MHz. The minimum standing wave intensities appeared when the ratio of the phase transformation frequency to the excitation frequency was 0.3 for all bone windows (Fig. 2).

**CONCLUSIONS** The phase transformation method has been proved to be effective in suppressing standing waves through variety bone windows. The advantage of this method is that it can enhance the energy of focal region and reduce the intensity of standing waves. The optimum excitation frequencies selected for different bone windows were obtained and it was confirmed that the excitation frequency and phase transformation frequency were relevant.

**Fig. 1 (abstract P35).**
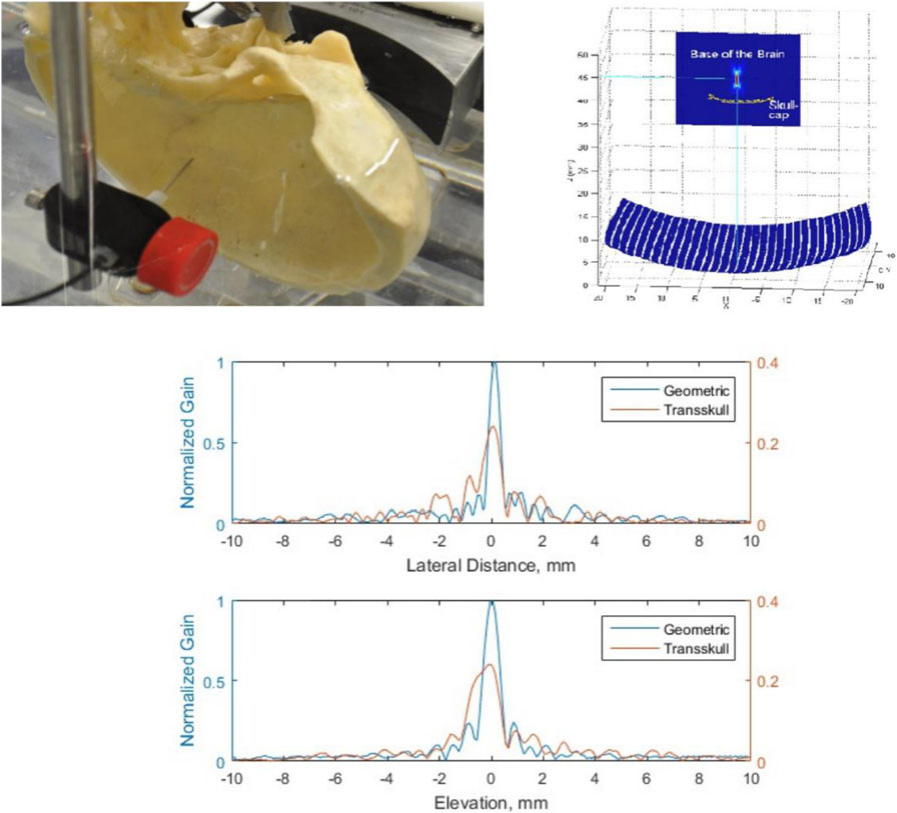
See text for description

**Fig. 2 (abstract P35).**
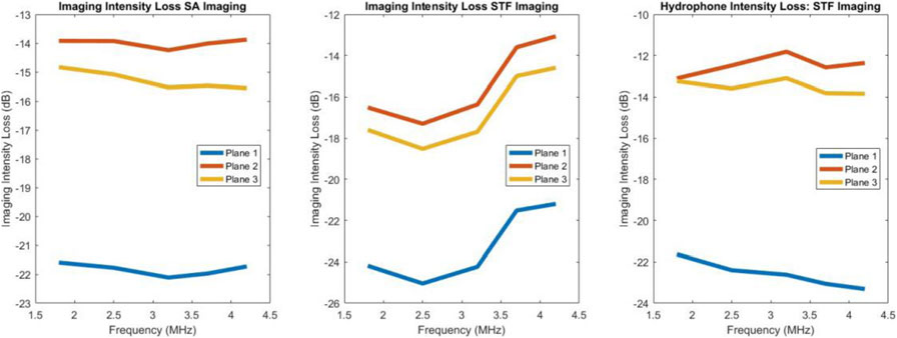
See text for description

### P33 Analysis and investigation of the major parameter effecting transcranial ultrasound phase aberrations: a preliminary study

#### Nan Wu, Guofeng Shen, Xiongfei Qu, Yazhu Chen

##### School of Biomedical Engineering, Shanghai Jiao Tong University, Shanghai, China

###### **Correspondence:** Nan Wu

**OBJECTIVES** Focused Ultrasound (FUS) is a noninvasive medical technology used for transcranial therapeutic applications. However, due to the complex acoustic properties of the skull, it is practically hard to obtain a sharp transcranial focus without phase correction. The aim of this research was to analyze and investigate the major parameter of the skull which has the greatest effect on phase aberrations when the ultrasound propagates through the skull. The correction of this parameter may simplify the method of ultrasonic phase correction.

**METHODS** The numerical model was based on the k-wave simulation environment, which was extensively tested and actually being used to simulate the ultrasound field before. The whole simulation environment was designed in water, and a digitized human skull profile, which was built from CT (computed tomography) images, was placed below the transducer. To investigate how each parameter of the skull effects phase aberrations, density, attenuation, velocity and geometry of the skull were taken into account individually. The wave propagation simulations were performed with one of the skull parameters, hypothesizing the others set to be the parameters of water throughout the simulation process. The benchmark configuration for this research was the spherically curved transducer driven at 700 kHz; the transducer had a curvature radius of 120 mm and a diameter of 90 mm. The focus acquired from different parameters was compared with each other after the simulations.

**RESULTS** A transducer placed in a homogeneous media (water), without and with the skull were simulated to be comparison groups. The focus of the transducer was shifted and defocused when the skull was placed in the sound field. However, a sharp focus still could be achieved when the density or attenuation of the skull was taken into account. An aberrant focus was generated when the velocity was set to 2850m/s (velocity of the skull). It was interesting to note that, the more velocity vectors penetrate the skull perpendicularly, the better a focus could be obtained.

**CONCLUSIONS** This research presented a simulation to analyze and investigate the major skull parameter effecting transcranial ultrasound wave phase aberrations. The velocity of skull could be the major parameter on phase aberrations, compared with the density and attenuation. Besides, the more velocity vectors perpendicular to the skull, the less phase aberrations and higher sound pressure could be obtained. A method on making more velocity vectors penetrating the skull perpendicularly that can simplify the correction of ultrasound wave phase will be done in the further research.

### P34 Protective effect of ultrasound on brain injury in mice

#### Feng-Yi Yang, Wei-Shen Su

##### Department of Biomedical Imaging and Radiological Sciences, National Yang-Ming University, Taipei, Taiwan

###### **Correspondence:** Feng-Yi Yang

**OBJECTIVES** The purpose of this study was to investigate the effects of low-intensity pulsed ultrasound (LIPUS) in mice with traumatic brain injury (TBI).

**METHODS** Controlled cortical impact (CCI) injury was used as a TBI animal model. Mice subjected to CCI injury were treated with LIPUS daily for a period of 28days. Behavioral assessments (mNSS and rotarod) were performed at day 28 after TBI. Histological examination was performed *via* cresyl violet staining. Edema regions were monitored by magnetic resonance imaging.

**RESULTS** Our data showed that functional impairments were significantly improved by LIPUS stimulation at day 28 after TBI. LIPUS significantly preserved brain tissue compared with the non-treated TBI group. Furthermore, LIPUS significantly reduced T2-weighted lesion volume in injured mice compared with the non-treatedTBI group.

**CONCLUSIONS** In summary, LIPUS stimulation improved long-term behavioral outcomes and attenuated brain tissue damage in mice subjected to TBI. Therefore, transcranial LIPUS stimulation may provide a potential treatment modality for TBI.

### P35 Broadband characterization of focused ultrasound transskull transmission

#### Parker D. O'Brien, Dalong Liu, Emad S. Ebbini

##### Electrical and Computer Engineering, University of Minnesota--Twin Cities, Richfield, Minnesota, USA

###### **Correspondence:** Parker D. O'Brien

**OBJECTIVES** The future of transcranial Focused Ultrasound (tFUS) for subtherapeutic (neuromodulation) or ablative treatments in the human brain relies on spatially specific therapeutic delivery with quantifiable power control to enable reversible and irreversible treatments. Single frequency transmission signals may not provide adequately defined foci nor minimal foci shift after propagating through the varying complex structures of the skull to target fine neuro-structures within the brain. Broadband transmission is highly likely to provide the most effective method of delivering specialized therapeutic treatments obtainted through its ability to recover distortion and loss from a single frequency with a wide range of available frequencies. This paper presents the broadband transmission characterization of FUS through rat skulls and human skulls *ex vivo* through various regions of the skulls to understand how different frequencies can be used to refocus distorted or recover lost transmission through the use of multiple frequencies.

**METHODS** Two dual-mode ultrasound arrays (DMUA) are used to transmit FUS through a series of human and rodent skulls. Both DMUAs are concave spherically focused, linear arrays with radii of curvature specific for the skull model used (r=100mm for human, r=40mm for rat) (Imasonic, France). Planar acoustic pressure measurements using needle hydrophones (Onda Corp, Sunnyvale, CA) were performed in degassed water with and without skull samples present (Fig. 1). Field scans were performed in prefocal and focal planes on a finely sampled grid with sub-wavelength spacing. Figure 1 shows the setup using a human skull in the water bath with the 600-micron hydrophone and DMUA (100-mm ROC) operating in the 0.7 - 2.5 MHz frequency range. Total power was estimated by integrating the square intensity (from hydrophone measurements). In addition, the focal plane measurements were back propagated to the interior of the skull to predict the shape of the acousticwavefront transmitted through the skull. Pre-focal field scans were performed to validate the backpropagation computational models between the focal plane and prefocal plane scans. Imaging distortion was characterized through a grid of single-point hydrophone measurements of synthetic aperture (SA) and single-transmit-focus(STF) imaging for the rat skulls with the 40-mm DMUA (operating bandwidth of 2.2 - 4.6 MHz). For all experiments, a range of frequencies relative to the human and ratmodel were chosen to accomplish a thorough broadband characterization for all relevant frequencies (Human: 1.00,1.35,1.70,2.10 MHz; Rat: 1.80, 2.50,3.20,3.70,4.20MHz).

**RESULTS** The hydrophone scans and backpropagation provide a thorough depiction of the transmission of focused ultrasound from the interior of the skull to the focalplane. The focal plane shows the distortion and loss of power caused by the skull. Figure 2 shows normalized therapeutic gain plots for the lateral and elevation directions. The hydrophone measured pre-focal planes showed strong agreement with back propagated simulations. As the frequency is increased, the distortion and attenuation increases. Figure 2 shows the back propagation results for a single frequency of 3.2 MHz. When analyzed as a whole frequency set, they show sections of the skull having different frequency propagation characteristics. Complex skull-bone structures and suture lines increase focal distortion and decrease total powertransmission. Contiguous regions display greater transmission with decreased distortion and foci shifting. Figure 3 shows the bregma suture line (Plane 1) causes stronger distortions than transmission regions between the bregma and lambda suture lines. The grid of points behind the skull show the distortion is not constant throughout thebrain. These distortions are dependent upon both frequency and the region of the skull the beam is traveling through to get to the respective imaging pixel. These results point towards creating a pre-treatment calculation of the effectiveness of a successful therapeutic delivery.

**CONCLUSIONS** The results demonstrate the feasibility of DMUA imaging (both SA and STF) to characterize the transmission loss through the skull as can be seen from the general agreement with the hydrophone estimates (Fig. 3). The results also demonstrate that a relatively wide transmission bandwidth in both rodent and human skulls. Based on these results, it would be possible to transmit broadband tFUS beams that can be designed to minimize the focal spot and improve the specificity of both ablative and subtherapeutic (neuromodulation) tFUS beams.

**Fig. 3 (abstract P35).**
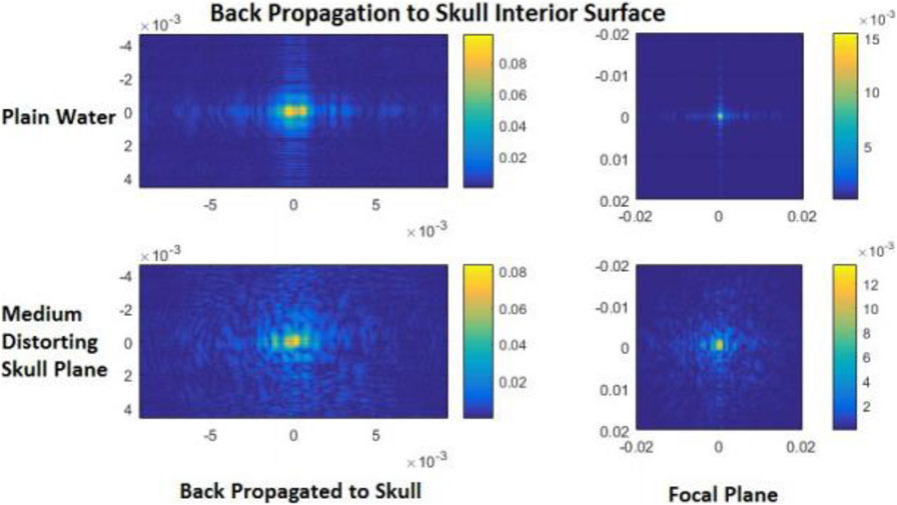
See text for description

**Fig. 1 (abstract P36).**
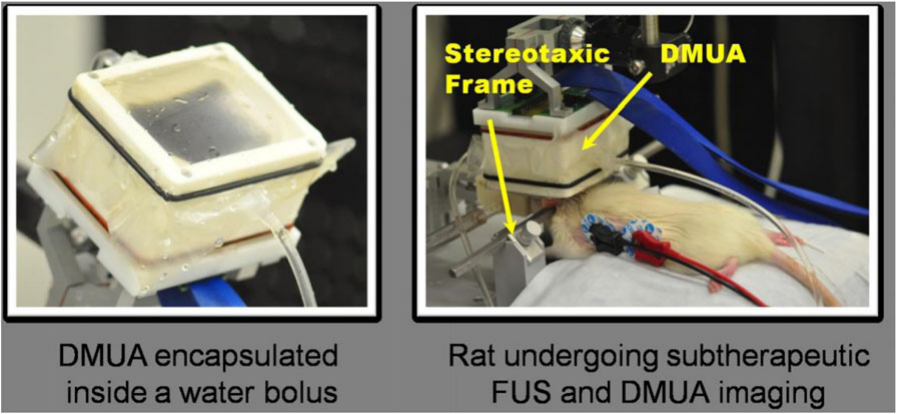
The dual­mode ultrasound array (DMUA) used in image­guided spatiotemporal control of tFUS in rat model *in vivo*

**Fig. 2 (abstract P36).**
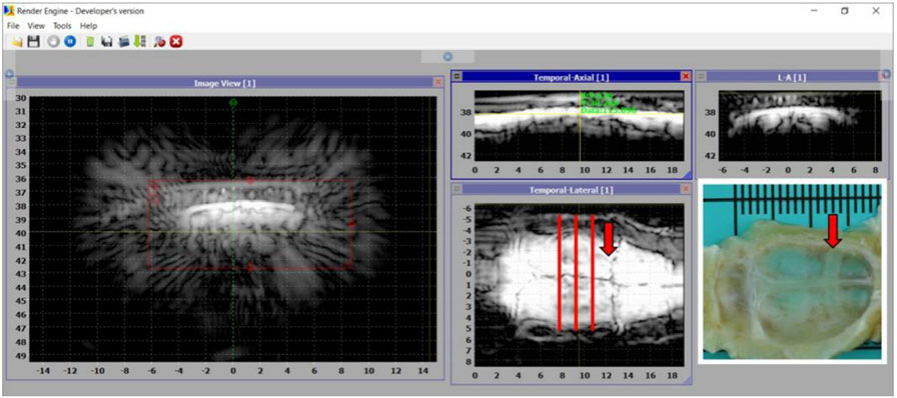
The 3D render interface used for visualization of the bregma suture line (thick arrows) as reference for the treatment planes (lines)

### P36 Real-time spatiotemporal control of transcranial focused ultrasound *in vivo*

#### Dalong Liu^2,1^, Emad S. Ebbini^1^

##### ^1^Electrical and Computer Engineering, University of Minnesota, Minneapolis, Minnesota, USA; ^2^Siemens, Seattle, Washington, USA

##### **Correspondence:** Dalong Liu

**OBJECTIVES** Transcranial focused ultrasound (tFUS) has been receiving increasing attention by numerous research groups worldwide. It is being investigated as a potential noninvasive treatment modality for tumor ablation, drug delivery through blood-brain barrier opening, Parkinson disease, etc. This renewed interest in tFUS canbe credited to advances in diagnostic imaging and image-guidance modalities, especially MRI. The distortion of tFUS beam through the skull continues to be a major challenge to the ultimate goal of reliably localizing and controlling the therapeutic tFUS dose to meet the demands of precision therapy of neurological disorders. The goal of this study is to establish the feasibility of precise real-time spatiotemporal control of tFUS energy application in a rat model *in vivo*.

**METHODS** The 3.5-MHz dual-mode ultrasound array (DMUA) prototype was used to deliver tFUS to anesthetized animals under IACUC-approved protocol. In each experiment, the animal was positioned prone on a stereotaxic unit with the head shaved and hair removed using depilatory cream to allow for good coupling with theDMUA through its water bolus (Fig. 1). A 3D scan of the skull was performed by mechanically translating the DMUA prototype using a 3-axis motor (caudal-to-rostral). This scan was performed to identify the lambda and bregma suture lines, which were used a a reference for the treatment planes. The DMUA system provided a 3Drender interface to allow the visualization of these markers upon the completion of the 3D scan and before starting the tFUS application in selected planes (based on thetarget circuitry for a given application.) All treatment planes were marked with respect to the bregma (e.g. bregma-2mm). Once the DMUA imaging/treatment slice was aligned with the desired treatment plane, imaging was performed before, during and after the application of the tFUS dose. Our system allowed for a full range of thermal and nonthermal control of tFUS by controlling the duty cycle. Spatial control of the tFUS beam was provided by precision refocusing using a multichannel arbitrary waveform generation driver. In addition, amplitude modulation of tFUS on a frame-by frame basis (up to 500 fps) was achieved using a closed-loop control system basedon DMUA feedback. For the purposes of this paper, we describe the spatiotemporal control of subtherapeutic tFUS beams for thermal therapy. A typical tFUS shot wasbetween 4 - 15 seconds with initial exposure of approximately 400 W/cm2 *in situ*, designed to reach the temperature set point in ~0.5 sec. Ultrasound thermography basedon DMUA beamformed echo data from the target region was used for feedback. A PID controller was employed to adjust the tFUS intensity to maintain the temperature at the set point. All animals were survived for 3 - 5 days after tFUS application and observed for any abnormal behavior or adverse reaction. Histological evaluation was performed to determine whether the delivered tFUS dose produced a BBB opening. For some animals, we extracted the skull and performed *in vitro* transskull field mapping experiments to characterize the actual distortion to the tFUS beam in different planes with respect to bregma.

**RESULTS** All animals that underwent the subtherapeutic tFUS applications described above (over 30 Sprague Dawley rats 275 - 475 gm, male and female) have survived the procedure and no adverse events were recorded. Figure 2 shows an example of the rendering of the 3D imaging data using the DMUA render engine. Onecan see the C-mode view with the lambda and bregma suture lines clearly visible with a thick arrow pointing to the bregma. The lines show the selected planes for tFUSapplication in a typical experiment. Figure 3 demonstrate the feasibility to visualize the tFUS beam access by rendering the DMUA elements and the skull (to scale) by using the results from the 3D scan. This allows for pre-treatment planning and post-treatment evaluation by bringing computational modeling and DMUA imaging data together in one computational model. Figure 4 shows an example result from a spatiotemporal control of tFUS-induced temperature rise of 4°C for 10 sec using the setup in Fig. 1. The pseudocolor overlay shows the spatial distribution of the heated region and the spatiotemporal map (axial-temporal) shows the localization in the axial direction.

**CONCLUSIONS** The results shown demonstrate the feasibility of real-time precise spatiotemporal control of tFUS dose application in a temperature control application (e.g. drug-delivery BBB opening), but the DMUA could be easily used to control nonthermal application of tFUS (e.g. neuromodulation). The results also demonstrate the unique advantages of the DMUA approach, where DMUA imaging data provides 3D volumetric rendering of the skull for target localization, pre-treatment planning and post-treatment evaluation. We envision a prescription-tFUS application using DMUA technology.

**Fig. 3 (abstract P36).**
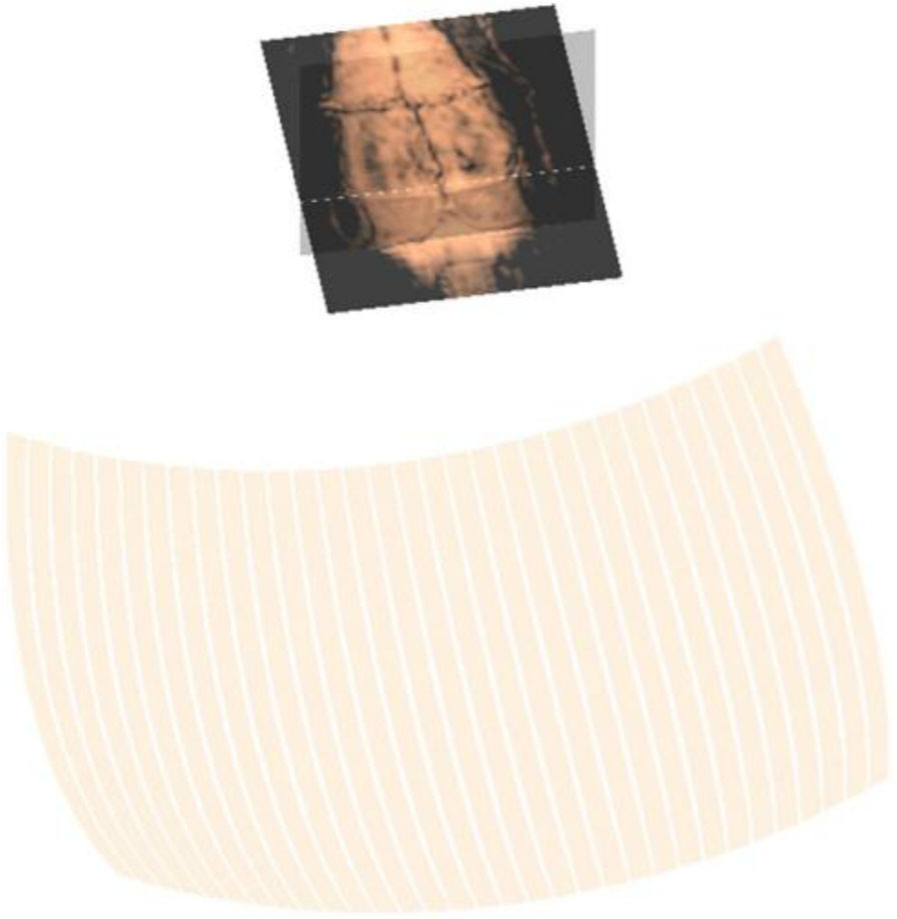
A 3D isometric view of the DMUA elements and the imaging/treatment slice with respect to the skull (as rendered from DMUA 3D imaging data)

**Fig. 4 (abstract P36).**
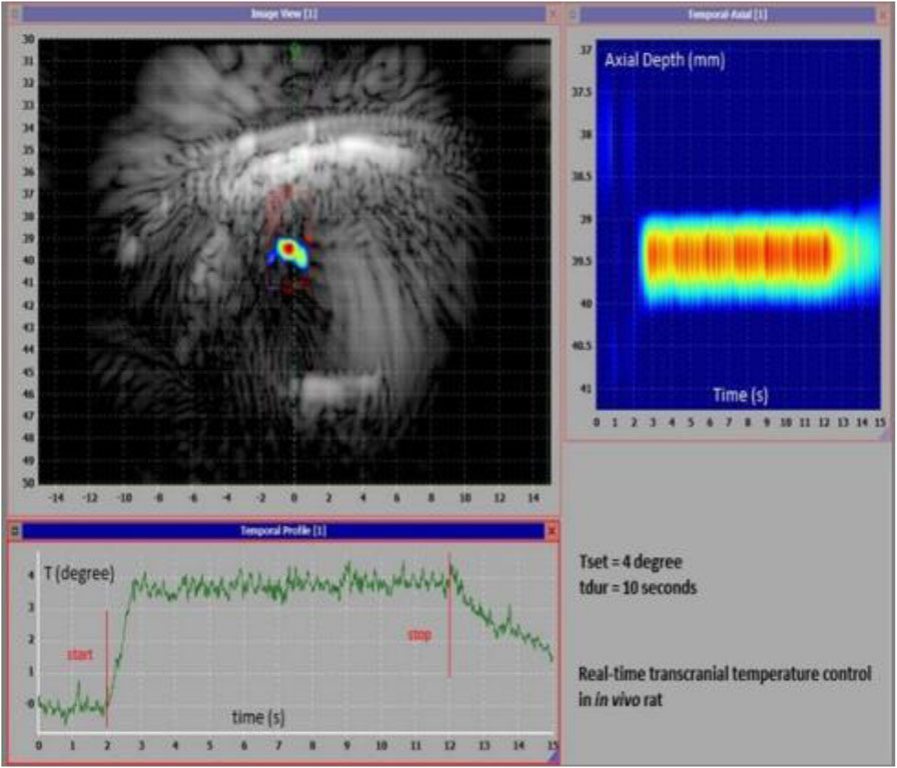
Typical result of real­time spatiotemporal control of subtherapeutic tFUS thermal application *in vivo* rat model

**Fig. 1 (abstract P37).**
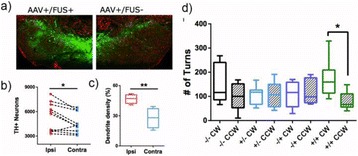
a) Immunofluorescence TH staining revealed much more dopaminergic projections on the AAV+/FUS+ side of the brain. b) Significantly higher number of TH+ neurons were found on the AAV+/FUS+ side of the brain compared to the contralateral side. c) Significantly higher dendrite density was observed on the AAV+/FUS+ side of the brain. d) Amphetamine-elicited behavioral studies revealed more frequent clockwise (toward the remaining lesion side) rotation, signifying more prominent dopaminergic function on the hemisphere receiving AAV+/FUS+ treatment

**Fig. 1 (abstract P38).**
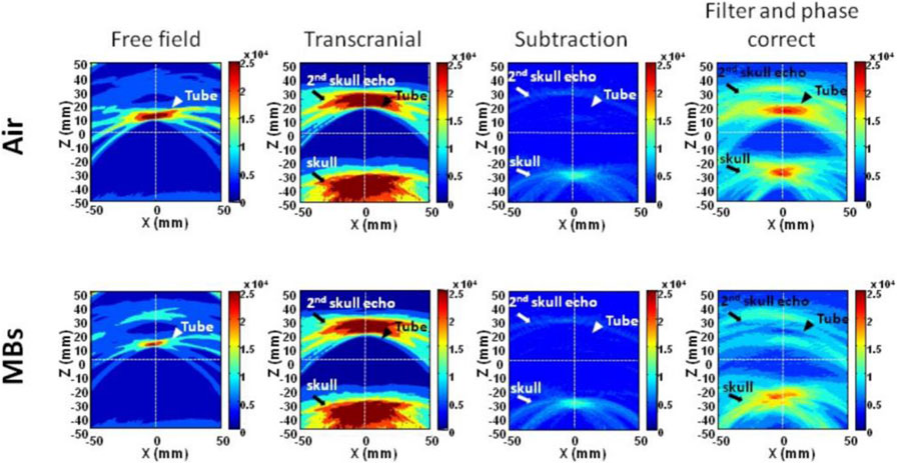
Passive imaging reconstruction: upper row shows the tube was filled with air, and lower row shows tube was filled with MBs

### P37 Focused ultrasound-facilitated AAV-GDNF delivery for neuro-protection and neuro-restoration in Parkinson ’s disease mice

#### Shutao Wang^1^, Oluyemi Olumolade^1^, Tara Kugelman^1^, Vernice Jackson-Lewis^2^, Maria Eleni Karakatsani^1^, Serge Przedborski^2^, and Elisa E. Konofagou^1,3^

##### ^1^Department of Biomedical Engineering, Columbia University, New York, NY, USA; ^2^Department of Neurology, Columbia University, New York, NY, USA; ^3^Department of Radiology, Columbia University, New York, NY, USA

###### **Correspondence:** Shutao Wang

**OBJECTIVES** Parkinson’s disease (PD) is the second most common neurodegenerative disorder affecting millions of patients worldwide. Currently, there is no cure for PD patients and only a few options are available to alleviate symptoms. With advances in gene therapy research, adeno-associated virus (AAV) has the potential to serve as carrier to introduce therapeutic genes to the human body. In contrast to invasive direct brain infusion, focused ultrasound (FUS) in combination with microbubbles has been shown to non-invasively and transiently open the blood-brain barrier (BBB). Here, our goal is to evaluate the potential neuro-protective and neuro-restorative effects of non-invasive AAV-GDNF delivery in a MPTP mouse model.

**METHODS** The PD mouse model was generated *via* intraperitoneal injections of MPTP toxin at 30 μg/kg over five consecutive days. Animals were then divided into four groups (n = 7-10 per group): control, FUS only, AAV injection only, and FUS+/AAV+. For the FUS only and FUS+/AAV+ groups, both striatum and substantia nigra were sonicated unilaterally using a single element FUS transducer. For the AAV+/FUS+ group, a 100 μl mixture of AAV-GDNF vectors and polydispersed microbubbles were administered intravenously before sonication. Mice were allowed to survive up to three months’ post sonication, which was followed by transcardial perfusion and tissue analysis.

**RESULTS** Systemically administrated AAV vectors were capable of crossing the opened BBB and viral transduction was observed to be concentrated at the FUS targeted brain regions (i.e. striatum and substantia nigra). The number of dopaminergic neurons at the point of sacrifice for each mouse was quantified by staining for tyrosine hydroxylase (TH) in the substantia nigra regions. As shown in Fig. 1, mice that received a combination of AAV-GDNF and FUS exhibited significantly higher protection to the subsequent MPTP insult. In addition, neurorestoration was observed in AAV+FUS treated mice that was previously dosed with MPTP toxin. The dopaminergic neuron projections on the FUS+/AAV+ hemisphere also had higher density than the contralateral side. Behavioral study was performed 12 weeks after the initial unilateral treatment, where amphetamine-elicited unilateral rotation was observed in mice from the combined (AAV+FUS) treatment group (p < 0.05).

**CONCLUSIONS** The findings of this study indicate the potential of gene delivery vectors for protecting and restoring the functions of dopaminergic neurons in PD and FUS as a non-invasive methodology for transcranial AAV delivery.

**Fig. 1 (abstract P39).**
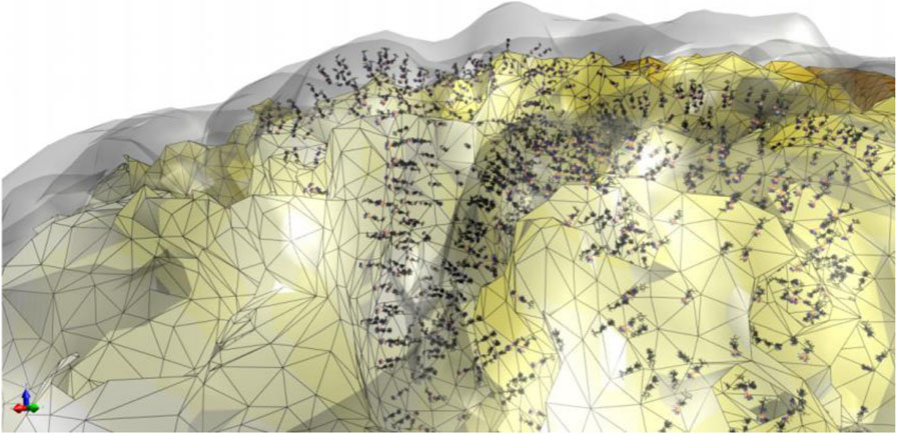
Cortex subregion of a human model with anatomically positioned pyramidal neurons and visualized transmembrane voltage activity

### P38 Transcranial focal passive detection and imaging: implementation on a multiple channel transmit/receive ultrasound phased array system

#### Chih-Hung Tsai, Hsin-Yu Chang, Chung-Han Wang, Hao-Li Liu

##### Department of Electrical Engineering, Chang Gung University, Taoyuan City, Taiwan

###### **Correspondence:** Chih-Hung Tsai

**OBJECTIVES** Burst-mode focused ultrasound (FUS) exposure combined with microbubbles (MBs) has been shown to induce temporal and local blood-brain barrier (BBB) opening. Contrast-enhanced imaging is now served an indicator to postoperatively confirm the occurrence of BBB opening. Developing a transmit/receive dualmode FUS apparatus has the potential to observe focal position and fulfill implementation of real-time monitoring of the occurrence of BBB opening. This study aims to disclose our recent development in using a self-designed multiple-channel transmit/receive system allow to perform passive cavitation analysis as well as to reconstruct focal beam distribution *via* passive imaging reconstruction.

**METHODS** Homemade 256-channel ultrasound phased array driving system was employed to drive a 256-channel FUS transducer to deliver focal transmit energy (fundamental frequency = 500 KHz, diameter = 120 mm, curvature = 100 mm). The transmit pulse was designed to be 0.006 ms of burst length, 2 Hz of pulse repeated frequency (PRF) and 3.56 MPa negative pressure output. Received circuits with the channel number ranging from 16-64 were employed to perform RF signal receiving. During the in-vitro experiments, either a strong needle reflector or microbubble (MB) tube was positioned with the flowed MBs concentrations been controlled, the multiple channel RF signals were received in parallel with the human skull were inserted. Passive cavitation detection was implemented, and passive imaging was reconstructed with the developed phase-corrected passive beamformed algorithm.

**RESULTS** We demonstrate the feasibility in using this self-designed multiple-channel system to serve as a platform to be operated at dual transmit/receive mode (Fig. 1). Multiple channels of RF data can be received in parallel to reconstruct the passive imaging. The system now can support up to 64-parallel channel receiving for the following signal analysis and passive imaging formation. Point-spread function (PSF) imaging can be reconstructed singly using 16-channel receiving, whereas higher channel provide superior SNR of imaging. The system also demonstrates the capability of the focal passive cavitation detection to real-time trace cavitation activity specifically originating from the focal point. We also demonstrated that the implementation of a filtered phase-correction processing been applied into the PSF reconstruction algorithm can successfully identify the focal ultrasound deposition when penetrating through the skull.

**CONCLUSIONS** We demonstrated the feasibility of the capability in using a self-built multiple-channel ultrasound transmit/receive system to perform passive imagingand real-time focal PCD. The system and architecture has the potential to be developed to real-time monitor the process of microbubble-facilitated FUS BBB opening process.

**Fig. 1 (abstract P40).**
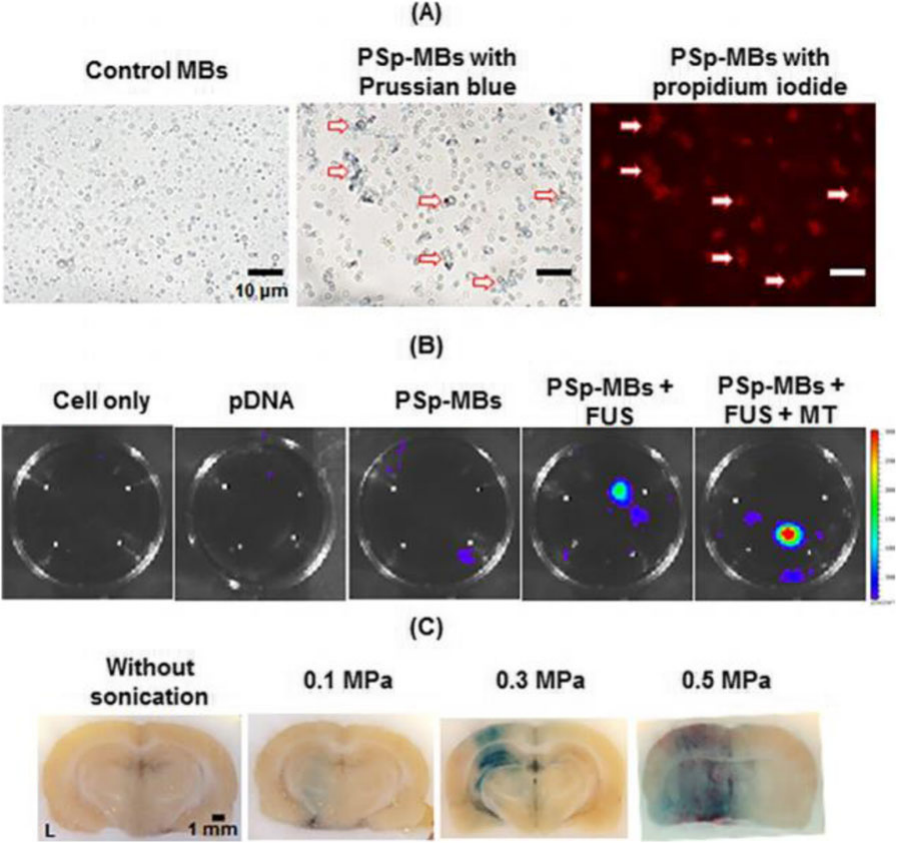
(A) Microscope images of the PSp­MBs with Prussian Blue and propidium iodide staining. The co­localization of the PSp nanoparticles in the Prussian Blue and propidium iodide staining indicated a good conjunction of PSPIO­pDNA and MBs. (B) Gene transfection efficency of PSp­MBs with different conditions, suggesting that PSp­MBs with ultrasound could achieve gene transfection. (C) BBB disruption of PSp­MBs cooperating with FUS by different parameters

### P39 Multiscale Modeling of transcranial focused ultrasound neurostimulation and experimental validation: initial results

#### Hazael Montanaro^1,2^, Mehmet S. Özdas^2^, Esra Neufeld^1^, Théo Lemaire^3^, Silvestro Micera^3^, Mehmet F. Yanik^2^, Niels Kuster^1, 2^

##### ^1^Computational Life Sciences, IT'IS Foundation for Research on Information Technologies in Society, Zurich, Zurich, Switzerland; ^2^Swiss Federal Institute ofTechnology (ETHZ), Zurich, Switzerland; ^3^Swiss Federal Institute of Technology (EPFL), Lausanne, Switzerland

###### **Correspondence:** Hazael Montanaro

**OBJECTIVES** Low intensity focused ultrasound (LIFU) has the demonstrated ability of non-invasively stimulating neural activity. This is of high value for therapeutic (stimulation, neuroprosthetics, etc.) and diagnostic (preoperative mapping, etc.) purposes. A multiscale simulation platform for image-based and personalized modeling of transcranial LIFU stimulation should be developed to allow mechanistic studies, hypothesis formulation and testing, device development, and, ultimately, personalized treatment planning, safety, and efficacy assessment. Experimental validation is crucial to establish confidence in and explore the limitations of the modeling.

**METHODS** The Sim4Life computational life sciences has been extended to: 1) Support full-wave acoustic simulation of transcranial sonication: For that purpose, new functionality to consider CT image-based skull inhomogeneity information (density, speed-of-sound, and attenuation maps) and partly compensate for related focus aberration and defocusing with multi-element transducer steering optimization has been implemented. 2) Allow for neuronal dynamics modeling: The NEURON library for compartmental neuronal dynamics modeling supporting detailed neuromorphology and channel dynamics has been integrated and parallelized simulations featuring large numbers of neurons and neural networks can now be performed. 3) Generate personalized, functionalized head models: Patient image data can be used to generate anatomical geometries by segmentation and/or morphing of presegmented models. CT image data informs on inhomogeneity, DTI image-data can be used for fiber tracking to generate neuronal axon models, and Python tools facilitate the anatomo-physiologically correct placement of cortical pyramidal neuron and deep brain stimulation relevant neurons (STN, GPi, IC). 4) Coupled acoustic and neuronal dynamics modeling: The Plaksin-Shoham-Kimmel (PSK) model of membrane-cavitation induced neurostimulation has been implemented and adapted for future use in combination with the compartmental cell models. Furthermore, coupled electromagnetic neuronal modeling is also supported. An experimental setup involving an acoustic transducer sonicating through a rat skull has been constructed. MicroCT image data has been acquired and the 3D pressure distribution inside the skull has been measured using computer controlled hydrophone scanning.

**RESULTS** The acoustic solver has been extensively validated previously against numerical and experimental data in homogeneous setups and setups with homogeneous obstacles. The new experimental data allows for the first time successful validation of a setup involving inhomogeneous media using the previously presented Gamma method for uncertainty assessment-based, objective comparison of 3D pressure distributions (Fig. 1).The neuron functionalized anatomical head models have been partly validated by comparing modeling of transcranial electric/magnetic and deep brain stimulation with experimental data from literature.The PSK-model could be simplified without significant impact on the results, thus enabling its integration into 3D, extended, morphological cell models. For that purpose, the previously bidirectional coupling of the electrophysiological and cavitation mechanics parts has been broken up and further separation allows to pre-compute costly parts of the model, accelerating the modeling by more than one order of magnitude.

**CONCLUSIONS** A multi-scale framework for the computational investigation of LIFU neuro-stimulation is being developed. It features image-based acoustic propagation modeling (including support for bone inhomogeneity) and focusing functionality, patient-specific functionalized anatomical model generation with realistic neuron placement, integrated neuronal dynamics simulation, and a coupled model of LIFU-induced neurostimulation that is currently still limited to 0D neuron models, but has been prepared for the future modeling of 3D-extended, morphologically-detailed physiological neuron models. Important parts of the platform (acoustic and neuronal simulations, functionalized models) could be successfully validated against new and existing experimental data.The presented progress is an important step towards the goal of allowing mechanistic studies, hypothesis formulation and testing, device development, and, ultimately, personalized treatment planning, safety, and efficacy assessment.

**Fig. 1 (abstract P41).**
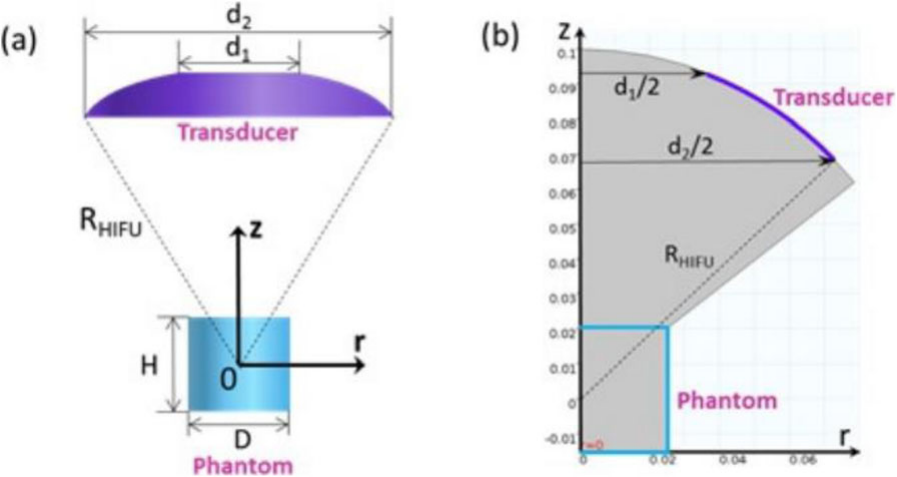
(a) Schematic of the physical model and (b) the geometry of the axisymmetric simulation spatial domain

### P40 SPIO-PEI-pDNA complex loaded microbubbles for ultrasound-based gene therapy in brain

#### Chun-Yao Wu, Ching-Hsiang Fan, Rih-Yang Huang, Chien-Wen Chang, Chih-Kuang Yeh

##### Department of Biomedical Engineering and Environmental Sciences, National Tsing Hua University, Hsinchu, Taiwan

###### **Correspondence:** Chun-Yao Wu

**OBJECTIVES** Recently, gene therapy has attracted much attention especially in neurodegenerative diseases. Currently, gene delivery within central nerves system mainly relies on invasive intracerebral injection or viral vectors to circumvent the obstacle of blood-brain barrier. Non-viral gene delivery *via* systematic transvascularroute is an attractive alternative since it is non-invasive. However, a high-yield and targeted gene delivery platform is still lacking. In order to improve the efficiency of gene delivery, this study proposed an ultrasonic sensing vector for gene delivery into brain through polyethylenimine (PEI)-superparamagnetic iron oxide (SPIO)-pDNAloaded microbubbles (PSp-MBs). Cooperating with ultrasound exposure, PSp-MBs could transport the PSp nanoparticles into the desired brain region by acoustic MBs cavitation activity. The rate of gene transfection would be enhanced by the modification of PEI onto PSp nanoparticles. In addition, by an externally applied magnetic field, magnetic targeting (MT) can further increase the deposition of PSp at the targeted location, enhancing the gene delivery.

**METHODS** The PSPIO was consisted of PEI molecular and SPIO nanoparticles (diameter: 10 nm) *via* ligand exchange. The PSPIO were then conjugated with pDNA and loaded onto the lipid surface of MBs by electrostatic force. PSPIO-pDNA (luciferase plasmid) modulated onto the MBs was confirmed by Prussian blue staining and propidium iodide staining. The size, concentration and PSPIO payload were measured by multisizer and plate reader, respectively. C6 glioma cell and Sprague-Dawleyrats (N = 4) were used in this study. The gene transfection efficiency and BBB opening region resulted from PSp-MBs with ultrasound (frequency = 1 MHz, energy = 0.1-0.5MPa, cycle = 5000, PRF = 1 Hz, sonication time = 60 s) were evaluated by bioluminescence imaging and Evans blue staining, individually. The MT process was performed by a 0.48 Tesla external magnet.

**RESULTS** Figure 1A shows the fabricated PSp-MBs. The co-localization of the PSp nanoparticles in the bright field images and the fluorescent image indicated a good conjunction of PSPIO-pDNA and MBs (as arrows). The mean size and concentration of PSp-MBs were 1.5 m and (5-10) × 109 bubbles/mL, respectively. The payload ofPSPIO and pDNA onto MBs were 134.5 g and 17.1 g, individually. Figure 1B shows that PSp-MBs with ultrasound could achieve gene transfection and the expression of gene could be further enhanced by MT process. We also confirmed that the PSp-MBs could perform successful BBB opening without bioeffects by the trigger ofultrasound with an acoustic pressure of 0.3 MPa (Fig. 1C).

**CONCLUSIONS** The MBs have fairly payloads of PSPIO-pDNA. We demonstrated that PSp-MBs with ultrasound can perform locally gene delivery and open BBB concurrently. In addition, the efficiency of gene delivery could be further enhanced by MT process. Future works include quantifying and tracking of distribution of gene delivery and Parkinson’s disease rats *via* magnetic resonance imaging.

**Fig. 2 (abstract P41).**
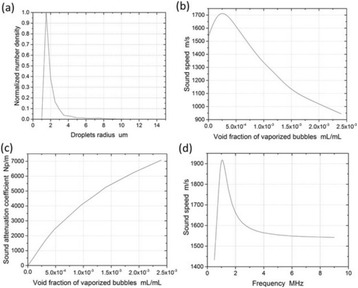
(a) Droplets size distribution used in the simulation. (b) Sound speed and (c) sound attenuation coefficient as a function of void fraction in the phantom. (d)Sound speed as function of frequency (void fraction is 2.52×10­4 mL/mL)

### P41 Numerical study of bubble area evolution during acoustic droplet vaporization enhanced HIFU treatment

#### Ying Xin^1^, Aili Zhang^1^, Lisa X. Xu^1^, Jeffrey B. Fowlkes^2^

##### ^1^School of Biomedical Engineering, Shanghai Jiao Tong University, Shanghai, China; ^2^Department of Radiology, University of Michigan Health System, Ann Arbor, Michigan, USA

###### **Correspondence:** Ying Xin

**OBJECTIVES** Acoustic Droplet Vaporization (ADV) has the potential to shorten treatment time of high intensity focused ultrasound (HIFU) while minimizing thepossible effects of microbubbles along the propagation path. Distribution of the bubbles formed from the droplets during the treatment is the major factor shaping the therapeutic region. However, there is no simulation report of this phase shift droplets assisted HIFU therapy, in which the bubbles form dynamically and only exists in places where certain acoustic conditions are met. In order to provide an approach for comprehensive parametric analysis, which could save time and effort in future studies, simulation of the dynamic formation of the bubbles inside the tissue during this treatment is carried out in this paper. The proposed model is verified using previously published experimental results. Numerical results predicting the effect of the presence of a preformed bubble wall are also obtained and discussed.

**METHODS** The schematic of the model setup and the axisymmetric geometry of the simulation spatial domain are shown in Fig. 1. The origin of coordinate system coincides with the HIFU geometric focus. The phase shift droplets are assumed to be distributed uniformly in the phantom. The vaporized bubbles also distribute evenly, which is expected in phantoms and likely the case for larger bubbles trapped within the vasculature in tissue. The average diameters of droplets are usually smaller than 5 microns (Kripfgans et al., 2000; Zhangand Porter, 2010), and the formed bubbles have a diameter 5 times of the one of the droplets based on ideal gas law, which is much smaller than the ultrasound wavelength (about 1.9 mm to 0.5 mm when the frequency is in the therapeutic range of 750 kHz to 3 MHz), so the bubble area is treated as a homogeneous medium. Linear sound equation with attenuation in the frequency domain is used to describe the acoustic pressure p. The density ρ, sound speed c and attenuation coefficient α are functions of the gas void fraction fG, which can be calculated directly from the bubble size distribution (Church, 1995). ADV droplets would be vaporized under certain conditions, which are related to ultrasound frequency, diameter of the droplets, super heat degree and total ultrasound ‘ontime’. The following assumptions are used to decide how much bubbles will form in certain acoustic field:(i) Threshold for droplets in phantom gel exposed to 750 kHz ultrasound is found to be 7.6 MPa as was found in experiment.(ii) The probability of droplets vaporizing increases linearly with rarefactional pressure in a range, which is function of the threshold according to the experimental results (Lo, et al, 2007). (iii) The incident wave got reflected at the interface of the bubble area based on the difference of the acoustic impedance of the two medium. Meanwhile the bubble area presents different impedance to different frequency. So the nonlinear part of the acoustic field is considered and simplified based on it.

**RESULTS** The ultrasound used in the model is 750 kHz, with a focal pressure of 9.8 MPa and 14.7 MPa. Each pulse is 20 μs. The droplets concentration is 1.3×106/mLand the size distribution of the droplets is shown in Fig. 2(a). The calculated sound speed and sound attencuation is shown in Fig. 2(b-d) The sectional view of pressure field and bubble area after 20 cycles of ultrasound exposure when focal pressure is 9.8 MPa is shown in Fig. 3. The sectional view of pressure field and bubble area after 200 cycles of ultrasound exposure when focal pressure is 14.7 MPa is shown in Fig. 4 (a, b). All the pressure was representing in dB scale using 9.8 MPa as the reference pressure. These results are close to the experimental result (Lo. at al, 2006) that they can describe the unique feature of the bubble area when the incident pressure varies. A layer of bubble has been proved to provide protection of the distal area in HIFU therapy. This model can also predict the bubble area when a bubble layer is created before treatment in the therapy (Fig. 4 (c, d)).

**CONCLUSIONS** This model can describe the bubble area evolution during the acoustic droplet vaporization enhanced HIFU therapy while using a simple linear acoustic model. The model can also be used in the case when a bubble layer pre-exists to protect the distal area from the HIFU ablation. The heating effect can be coupled to the model in the future and then it can be used as a planning tool in ADV enhanced HIFU therapy and predict the treatment outcome.

**Fig. 3 (abstract P41).**
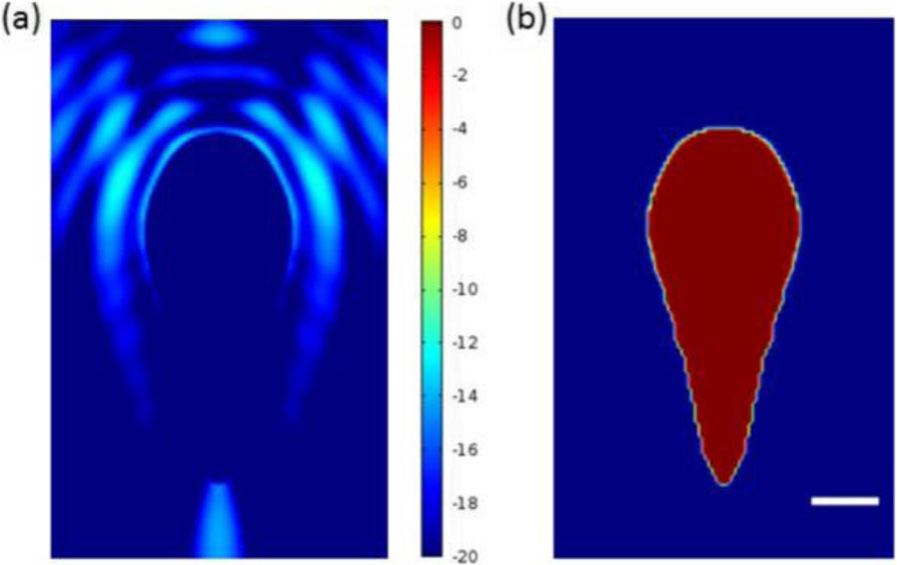
The sectional view of (a) pressure field and (b) bubble area after 20 cycles of 20 μs pulses of ultrasound exposure, of which focal pressure is 9.8 MPa and frequency is 750 kHz. The pressure was represented in dB scale using 9.8 MPa as the reference pressure. The white scale bar represents 2 mm

**Fig. 4 (abstract P41).**
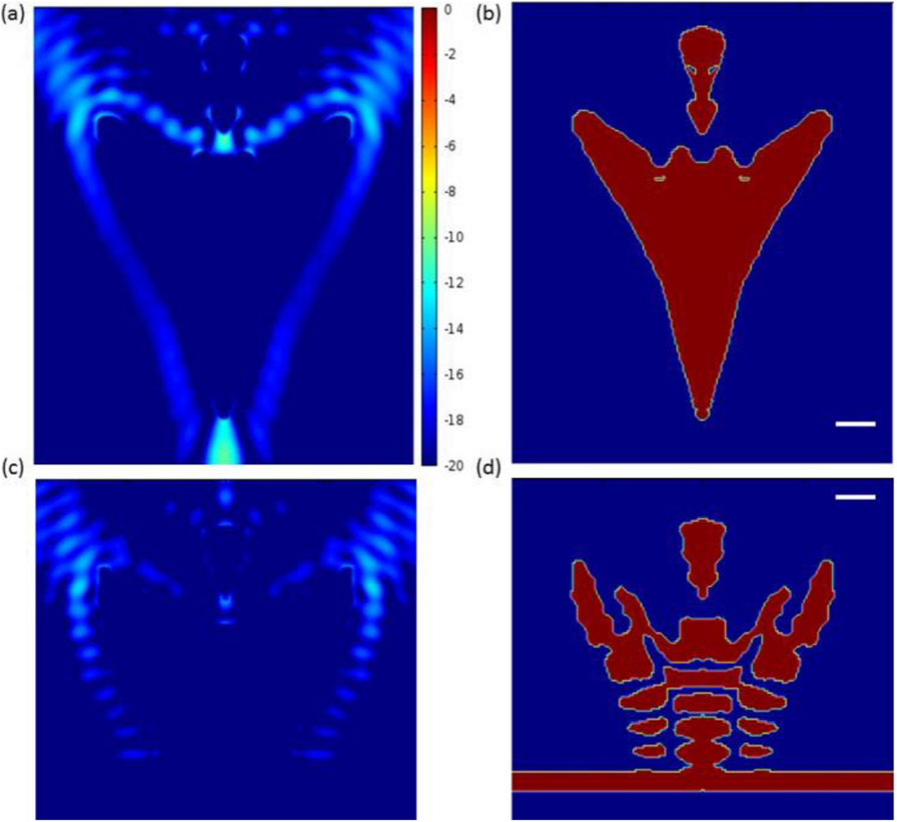
The sectional view of (a) pressure field and (b) bubble area after 200 cycles of 20 μs pulses of ultrasound exposure, of which focal pressure is 14.7 MPa andfrequency is 750 kHz. The sectional view of (c) pressure field and (d) bubble area when a 1 mm thick of bubble layer pre-existed. The pressure was represented in dB scaleusing 9.8 MPa as the reference pressure. The white scale bar represents 2 mm

**Fig. 1 (abstract P42).**
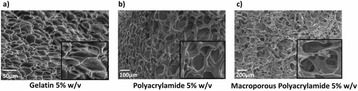
Scanning electron microscopy of (a) gelatin, (b) polyacrylamide and (c) macroporous polyacrylamide. Zoomed in sections show closed pockets or pores for gelatin and PAA but, MPAA pore structure provides fluid a path to follow throughout the gel. Fluid can travel from the pore on the gel surface to the deeper pore behind (bottom right rectangular areas)

**Fig. 2 (abstract P42).**
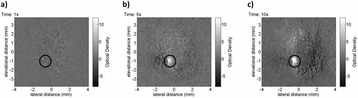
From images acquired, differences images at 1, 5 and 10 s after the start of the sonication (Pneg: 8MPa, PL: cotinuous wave) were obtained (a-c). The black circle denotes the sonicated region, as the darker dye is cleared from hydrogel, the lighter clearer gel surface is shown causing an increase in optical density

### P42 Noninvasive and localised acoustic micropumping – an *in vitro* study of an ultrasound method that enhances drug distribution through a physiologically-relevant material

#### Ahmed Elghamrawy^1^, Florentina de Comtes^1^, Hasan Koruk^2^, Ali Mohammed^3^, James J. Choi^1^

##### ^1^Department of Bioengineering, Imperial College London, London, UK; ^2^Mechanical Engineering, MEF University, Istanbul, Turkey; ^3^Materials, Imperial College London, London, UK

###### **Correspondence:** Ahmed Elghamrawy

**OBJECTIVES** Acoustic streaming - the displacement of fluid by sound - has been proposed as the mechanism for therapeutic effects, such as drug distribution enhancement and neurostimulation, yet there has been no direct observation or characterisation of this effect in soft tissue microenvironments, making it difficult to optimise and control. Post-mortem and indirect analyses have revealed changes in dye distribution and neuronal excitation, but it has remained uncertain whether these biological outcomes were due to acoustic streaming or other responses, such as acoustic cavitation. We aimed to be the first to directly observe ultrasound-induced streaming during sonication in a tissue-mimicking material.

**METHODS** Our initial challenge was to create an appropriate material for studying acoustic streaming. We hypothesised that existing hydrogels (e.g., polyacrylamideand gelatin) used as ultrasound phantoms, mimic the acoustic properties of tissue, but not the tissue microenvironment. We created three phantoms – gelatin, polyacrylamide (PAA), and a new macroporous polyacrylamide (MPPA) hydrogel – and analysed their structure using scanning electron microscopy (SEM) and mercury intrusion porosimetry (MIP). Based on our findings, we selected the MPPA for our tissue-mimicking material. Focused ultrasound (FUS) pulses were emitted from asingle-element transducer [centre frequency (fc): 5 MHz], which was driven by a function generator through a power amplifier. The focal point of a FUS transducer’s beam (axial FWHM: 3.2 mm, lateral FWHM: 0.45 mm) was placed at the distal surface of the MPPA. A model drug (Bromophenol blue) was injected in and around the focal region and different FUS pulses ([peak-negative pressure (Pneg): 8, 12MPa, pulse length (PL): continuous wave], [Pneg: 25MPa, PL: 5 ms, PRF: 20 Hz]) wereemitted. Dye movement (i.e., clearance) at the hydrogel surface was observed with a video camera.

**METHODS** Our initial challenge was to create an appropriate material for studying acoustic streaming. We hypothesised that existing hydrogels (e.g., polyacrylamide and gelatin) used as ultrasound phantoms, mimic the acoustic properties of tissue, but not the tissue microenvironment. We created three phantoms – gelatin, polyacrylamide (PAA), and a new macroporous polyacrylamide (MPPA) hydrogel – and analysed their structure using scanning electron microscopy (SEM) and mercury intrusion porosimetry (MIP). Based on our findings, we selected the MPPA for our tissue-mimicking material. Focused ultrasound (FUS) pulses were emitted from a single-element transducer [centre frequency (fc): 5 MHz], which was driven by a function generator through a power amplifier. The focal point of a FUS transducer’s beam (axial FWHM: 3.2 mm, lateral FWHM: 0.45 mm) was placed at the distal surface of the MPPA. A model drug (Bromophenol blue) was injected in and around the focal region and different FUS pulses ([peak-negative pressure (Pneg): 8, 12MPa, pulse length (PL): continuous wave], [Pneg: 25MPa, PL: 5 ms, PRF: 20 Hz]) wereemitted. Dye movement (i.e., clearance) at the hydrogel surface was observed with a video camera.

**RESULTS** SEM revealed that although gelatin and PAA were porous, they had pockets of water separated by walls of material (Fig. 1a, b). This suggests that acoustic streaming with these materials are unlikely to occur unless enough stress is applied to break the walls. In contrast, MPPA had interconnected pores throughout the gel – a feature that more accurately resembled the interstitial space of soft tissue (Fig. 1c). The gelatin, PAA and MPPA had a porosity of 81, 76 and 88%, respectively; a permeability of 2400, 1500 and 6500 mdarcy, respectively. We then selected MPPA for further study, because it most accurately mimicked the tissue microenvironment. Using focussed ultrasound in continuous wave (Pneg: 12MPa), we directly observed acoustic streaming in the MPPA material in the form of the dye progressively clearing from the focal region (Fig. 2). The clearance rate is represented by the change in optical density over time (Fig. 3). Pulsed ultrasound was applied (Pneg: 25MPa, PL: 5 ms, PRF: 20 Hz) having the same average intensity as 8MPa continuous wave and an increase in clearance rate was observed. Moreover, pulsed ultrasound reduced unwanted thermal effects.

**CONCLUSIONS** Our results reveal the first direct observations of acoustic streaming in a soft tissue microenvironment. We showed that although gelatin and polyacrylamide have acoustic properties suitable for ultrasound imaging and hyperthermia experiments, they are not effective in studying acoustic streaming. Instead, we recommend the use of MPPA, because it supports fluid flow through its microstructure by having interconnected pores or channels. Using this phantom material, we wereable to study acoustic streaming under different exposure parameters; finally demonstrating that pulsed ultrasound produces greater acoustic streaming with less risk ofthermal damage than with continuous wave emission using the same acoustic energies. Our future work is to study optimise ultrasound parameters to maximise acousticstreaming in this material and in in-vivo tissue for enhancing drug distribution and neurostimulation.

**Fig. 3 (abstract P42).**
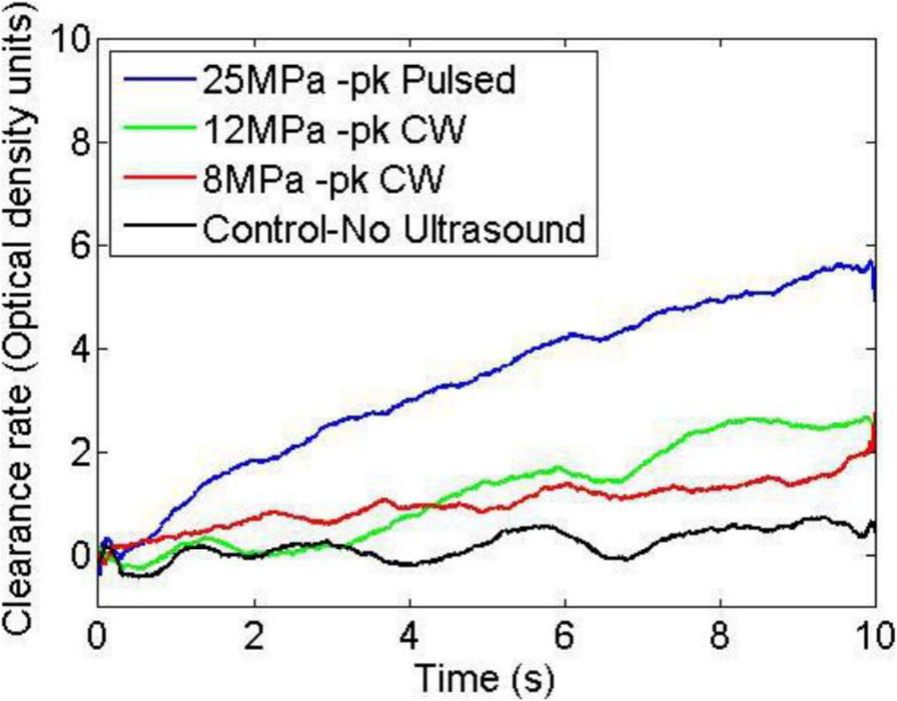
Clearance rate is the average of optical density values of the pixels in the black circle (Fig. 2) over time. As continuous wave ultrasound pressure increases from Pneg: 8MPa (red) to 12MPa (green) there is a slight increase in dye clearance rate. Pulsed ultrasound was applied (Pneg: 25MPa, PL: 5 ms, PRF: 20 Hz) having thesame average intensity as 8MPa continuous wave and an increase in clearance rate was observed (blue). All applied parameters initiated dye clearance compared to whenno ultrasound was applied (black)

**Fig. 1 (abstract P43).**
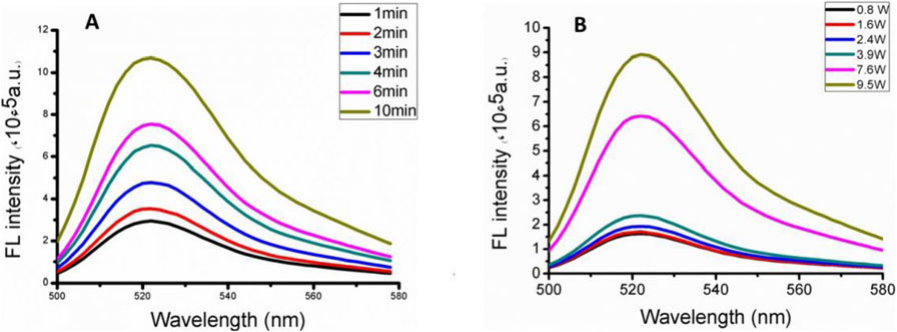
The ROS generation of HSA-Ce6 NAs under US irradiation. (A) Fluorescence emissin spectra of HSA-Ce6 NAs in DCFH-DA solution with the increase of US irradiation time. (B) Fluorescence emissin spectra of HSA-Ce6 NAs in DCFH-DA solution with the increase of US irradiation intensity

**Fig. 2 (abstract P43).**
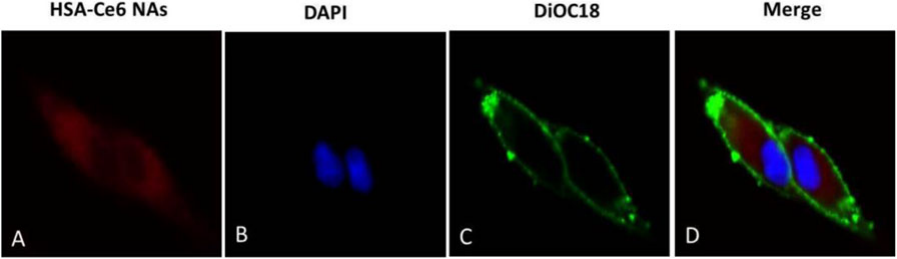
Fluorescence images displayed cellular localization of HSA-Ce6 NAs after 3 incubation with HSA-Ce6. Red represented the fluorescence HSA-Ce6NAs. Blue represented the fluorescence of DAPI and green represented the fluorescence of the cell membrane of U87 cells

### P43 MRI-guided focused ultrasound mediated smart albumin-sonosensitizer nanoassemblies for sonodynamic therapy of glioma

#### Qian Wan^1^, Dehong Hu^1^, Mengjie Chen^2^, Zonghai Sheng^1^, Jun Zhou^2^, Xin Liu^1^, Hairong Zheng^1^

##### ^1^Shenzhen Institutes of Advanced Technology, Chinese Academy of Sciences, Shenzhen, Guangdong, China; ^2^Medical College of Chinese Three Gorges University, Yichang, China

###### **Correspondence:** Qian Wan

**OBJECTIVES** Sonodynamic therapy (SDT) represents an emerging approach that offers the possibility of non-invasively eradicating solid tumors in a site-directed manner, which involves the synergistic effect on cell damage by the combination of the sonosensitizer and ultrasound, The sonosensitizer is the key factor of SDT.Chlorin e6(Ce6) has been widely used as a sonosensitizer in sonodynamic therapy (SDT) on many human tumors, indicating Ce6 possesses excellent sono-activities with tiny toxicity.In this study, we designed and synthesised multifunctional human serum albumin-chlorin e6 nanoassemblies (HSA-Ce6 NAs) that integrate imaging andtherapy functionalities into a single nano-platform for MRI guided sonodynamic therapy. We show the albumin-sonosensitizer nanoassemblies is highly accumulated in tumor tissue and had highly effective in destructing cancer cells under ultrasound and MRI-visible *in vivo* and thus, suitable for theranostic applications in cancer.

**METHODS** HSA-Ce6 NAs synthesis as follows: after dissolution of HSA in water, Ce6 was added into the solution to precipitate the HAS-Ce6, Then HSA-Ce6 NAs were mixed with MnCl2 solution at a molar ratio of 2:1 for the HSA-Ce6 NAs.To detection of ROS in HSA-Ce6 NAs, different samples were mixed with 1 mM 2′,7′-dichlorofluorescin diacetate (DCFH-DA), and then irradiated by different US times or US intensities.In vitro SDT: The ultrasound transducer (diameter: 1.5 cm, resonance frequency: 0.5 MHz, duty factor: 50 %, interval: 100 ms; ultrasonic intensity: 0.5 W/cm2) was fixed at the bottom of water bath. The cell culture plate was suspended 10 cm above the ultrasound transducer. In vivo MRI: The *in vivo* MRI imaging experiments were acquired using a 3.0 T clinical MR scanner (TIM TRIO, Siemens, Germany) with a small animal coil. T1-weighted MR images were acquired using the following parameters: TSE sequence, TR= 700 ms, TE =13 ms, FOV =32 × 45 mm, slice thickness= 1 mm and flip angle=180°.

**RESULTS** The sonodynamic effect of HSA-Ce6 NAs was confirmed by measuring reactive oxygen species using DCFH-DA as a detector, as shown in Fig. 1, thefluorescence intensity of ROS exhibits a time-dependent and an intensity-dependent enhancement, indicating ROS from HSA-Ce6 NAs upon ultrasound irradiation. Thecell uptake behavior of HSA-Ce6 NAs was investigated through confocal microscopy, the HSA-Ce6 NAs has high cell uptake efficiency with high fluorescence intensityin U87 cells (Fig. 2). Moreover, the SDT *in vitro* of HSA-Ce6 NAs could be further improved. It was found that single HSA-Ce6 NAs or ultrasound treatment could only induce partial cell death at the current conditions. In marked contrast, the combination treatments (SDT) were found to be highly effective in destructing cancer cells. Indicating that ultrasound has a good penetrability and SDT effect (Fig. 3). In addition, the HSA-Ce6 NAs can serve as chelating agents to capture Mn2+ for MRI imaging by forming stable chelates. The *in vivo* T1-weighted MR imaging of U87 tumor-bearing mice was carried out after iv injection of HSA-Ce6 NAs, The T1 signals of tumor on mice strengthened with the increase of time interval, and reached a peak after 24 h postinjection of HSA-Ce6 NAs (Fig. 4). This indicated the HSA-Ce6 NAs could provide the optimal time window for sonodynamic therapy so that the ultrasound irradiation could be conducted in the targeted lesion.

**CONCLUSIONS** We have shown our human serum albumin-chlorin e6 nanoassemblies are indeed visible by MRI *in vivo* and that they can be targeted by FUS to deliver and release reactive oxygen species (ROS) to kill cancer cells, paving the way for their theranostic applications under MRI-guided sonodynamic therapy.

**Fig. 3 (abstract P43).**
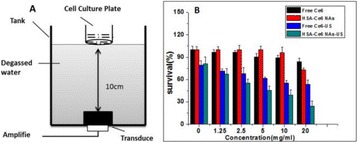
The *in vitro* SDT in U87 cells. (A) Schematic diagram of the insonation device. (B) Quantitative evaluation of cell survivals of each group

**Fig. 4 (abstract P43).**
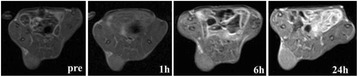
*In vivo* MRI images of the mice bearing U87 tumor after iv injection of HSA­Ce6 NAs at different times. I.V. injection dose of HSA­Ce6 NAs is 2.0 mg Ce6/kg

**Fig. 1 (abstract P44).**
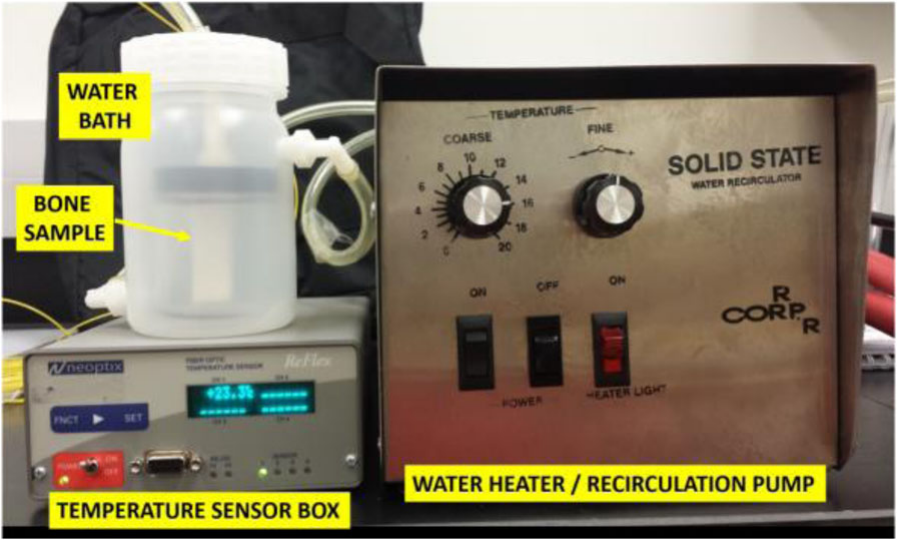
Experimental apparatus

**Fig. 2 (abstract P44).**
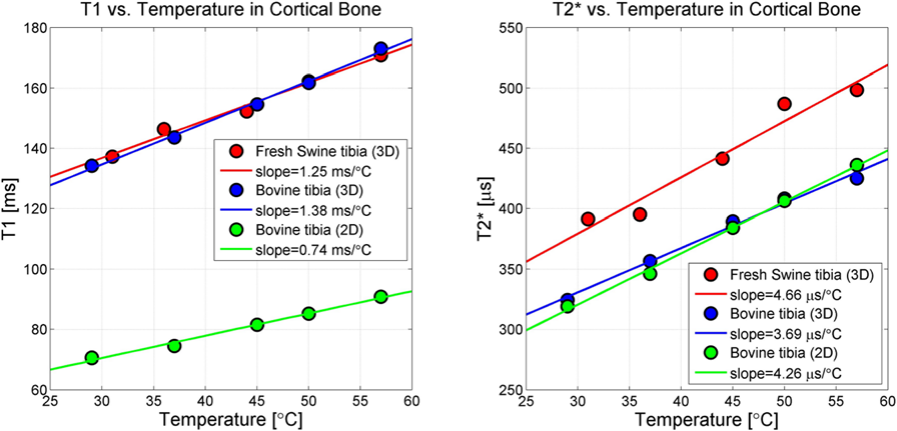
T1 and T2* dependence on temperature in both swine and bovine bones when measured with 3D techniques, and in bovine bone when measured with 2D techniques

### P44 Towards rapid MR thermometry in cortical bone

#### Phoebe Miller^1^, Sina Tafti^1^, David Keder^2^, Quinton Miller^3^, Darius Hossainian^3^, Wilson Miller^1^

##### ^1^Radiology and Medical Imaging, University of Virginia, Charlottesville, Virginia, USA; ^2^Physics, University of Virginia, Charlottesvle, Virginia, USA; ^3^Biomedical Engineering, University of Virginia, Charlottesvle, Virginia, USA

###### **Correspondence:** Phoebe Miller

**OBJECTIVES** Bone metastases are an important, FDA-approved treatment target for MR-guided focused ultrasound therapy. However, it is not currently possible to noninvasively measure temperature in bony tissues. Conventional PRFS-based MR thermometry is likely not feasible in cortical bone due to very fast T2* signal decay. Other MR properties, including T1 and T2*, also depend on temperature and thus offer alternative pathways for performing MR thermometry in bony tissues. To devise ascheme for performing thermometry based on these MR properties, we first must know the relationship between these variables and temperature. The first objective of this investigation was to quantify these relationships in ex-vivo tissue, with the eventual goal of developing a strategy for performing such measurements in-vivo during FUS sonication. To date, such measurements have been performed using 3D ultrashort echo time (UTE) acquisition pulse sequences, which severely limits the achievable temporal resolution. Therefore, our second objective was to explore the feasibility of performing accurate temperature measurements in cortical bone using much faster 2D acquisitions with slice selective RF pulses.

**METHODS** We measured the temperature dependence of T1 and T2* in cortical bone from a recently euthanized swine tibia and bovine long bone using both 2D and 3D pulse sequences. In order to accurately measure these MR properties, we had to create a system in which we could vary the temperature of the bone as well as keep the temperature constant within the MR scanner during MR imaging. To achieve this, we built a system that consisted of an MR-safe water bath, a combination waterheater/recirculation pump that regulated the temperature of the water bath, and fiber-optic temperature sensors that monitored the temperature of a bone sample immersedin the water bath (Fig. 1). The bone sample was placed inside of the regulated water bath and its temperature was monitored by the fiber optic sensors while we acquired our measurements that would allow us to calculate the temperature dependence of the MR variables.T1 was measured at 3T by acquiring a series of spoiled gradient-echo ultrashort UTE images, each having the same TR and TE but different flip angles, and then fittingthe measured signal-versus-flip angle curve to the theoretical dependence at each voxel contained entirely within the bone. In order to find this theoretical dependence, we derived an equation that would give us the steady-state incoherent signal as a function of T1 and T2*. T2* was measured by acquiring a series of spoiled gradient-echo images, each having the same TR and flip angle but different echo times, and then fitting the measured signal-versus-TE curve to a mono-exponential decay at each voxel contained entirely within the cortical bone. The T1 and T2* measurements were performed at different temperatures ranging from below body temperature to nearly60°C. The temperature of the water bath was held constant during each T1 and T2* measurement.

**RESULTS** T1 and T2* were both found to increase approximately linearly with temperature. The slope of this linear dependence was measured to be 1.25 ms/°C for T1and 4.66 μs/°C for T2* in swine tibia and 1.38 ms/°C for T1 and 3.69 μs/°C for T2 in bovine long bone, utilizing the 3D imaging pulse sequence (Fig. 2). Additionally, we measured T1 and T2* in bovine bone using the 2D imaging pulse sequence, yielding slopes of 0.74 ms/°C for T1 and 4.26 μs/°C for T2* (also Fig. 2). The quality of the theoretical fits to the measured signal was generally excellent (Fig. 3).

**CONCLUSIONS** The T1 variation with temperature was found to be similar in swine and bovine cortical bone when measured using a 3D pulse sequence with a nonselective excitation RF pulse. However, the T1 values measured using a 2D pulse sequence with a slice-selective excitation RF pulse were vastly different. This discrepancy is almost certainly due to naive application of the theoretical signal versus T1 relationship, which does not account for the non-ideal slice profile of our slice-selective excitation RF pulse. Such effects must be considered when performing T1-based thermometry using 2D acquisitions. By contrast, the T2 variation with temperature was found to be similar when the same sample was measured using 2D and 3D acquisitions. Thus it may prove to be more straightforward to achieve quantitative accuracy when performing T2-based thermometry using 2D acquisitions. Whereas our 3D pulse sequence required 1 minute to acquire a single image, our 2D pulse sequence required only 2.5 seconds. This time scale is much more favorable for monitoring temperature changes during FUS application *in vivo*.

**Fig. 3 (abstract P44).**
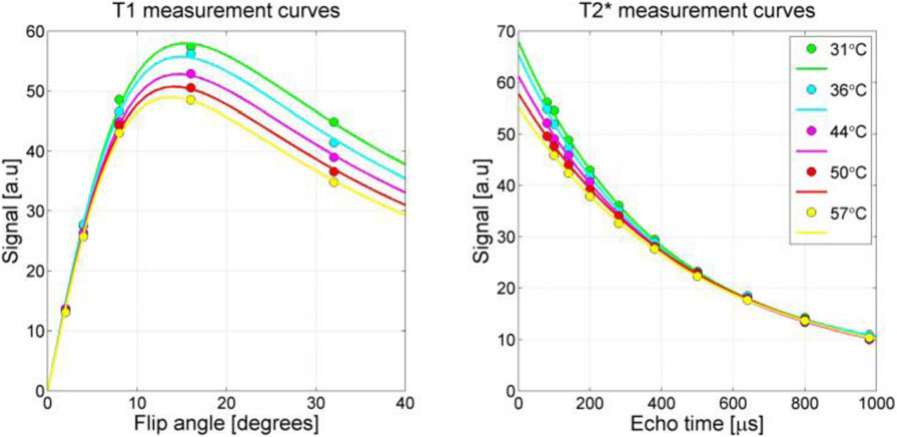
T1 and T2* measurement data, together with best fit curves, for swine tibia

**Fig. 1 (abstract P46).**
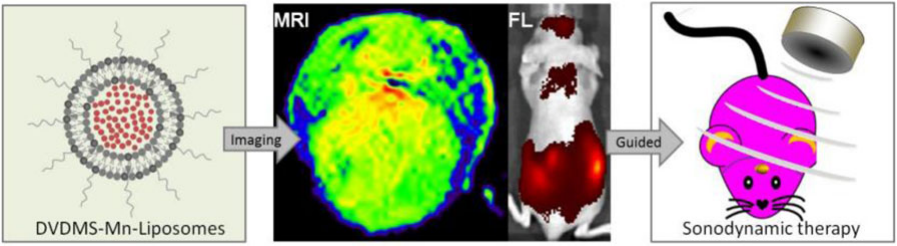
See text for description

**Fig. 1 (abstract P49).**
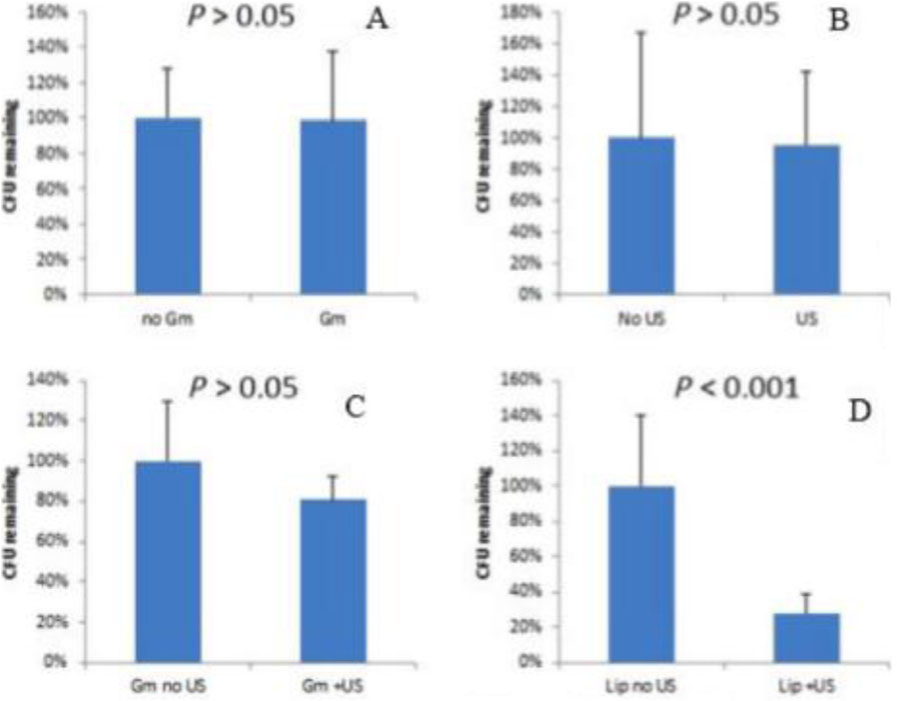
A statistical analysis was performed using the pair­wise comparison “F­Test Two Samples for Variances” method. The number of viable bacteria in the alginate­based biofilms were reduced by 72 percent. No statistical difference (p>0.05) was found among A, B, C cases

### P45 Multi-disciplinary integration of ultrasound molecular imaging

#### Zhigang Wang

##### Institute of Ultrasound Imaging, Chongqing Medical University, Chongqing, China

**OBJECTIVES** In recent years, with the rapid development of medical imaging, ultrasound molecular imaging becomes one of the hot spots in molecular imaging research field.

**METHODS** The design of molecular probes is the key point and prerequisites for ultrasound molecular imaging. People increasingly pay more attention to the targeted ultrasound contrast agents which are the ultrasound molecular probes. And the intersection of multiple disciplines will promote the development of ultrasound molecular imaging.

**RESULTS** It is also urgent to develop a set of special equipment for efficient ultrasound molecular imaging.

**CONCLUSIONS** The ultrasound molecular imaging instrument, microbubble/microsphere triggered device, imaging monitoring and ultrasound molecular probes can be integrated into the low intensity ultrasound molecular imaging and therapy system, which will hopefully bring about the integration of ultrasound molecular imaging, *in vivo* drug deliveryand controlled release and evaluation of treatment efficacy. It provides an innovative research platform for ultrasound molecular imaging and therapy.

### P46 MR and fluorescence dual-modal imaging-guided sonodynamic therapy of gliomas through a multifunctional theranostic nanoplatform

#### Fei Yan^1^, Meijun Zhou^2^, Hairong Zheng^1^

##### ^1^Shenzhen Institutes of Advanced Technology, Shenzhen, China; ^2^Department of Ultrasonography, The Third Affiliated Hospital of Southern Medical University, Guangzhou, China

###### **Correspondence:** Fei Yan

**OBJECTIVES** The aim of this study is to develop a multifunctional theragnostic nanoplatform for MR and fluorescence dual-modal imaging-guided sonodynamic therapy of gliomas.

**METHODS** Sinoporphyrin sodium (DVDMS), a NIR-absorbing sonosensitizer and photosensitizer, was firstly used to chelate with manganese ion (Mn2+) and then encapsulated into liposomes by thin-film rehydration method to fabricate DVDMS-Mn-Liposomes (DVDMS-Mn-LPs). The characterizations of DVDMS-Mn-LPs, their imaging capability *in vitro* and *in vivo* and SDT effect in subcutaneous and orthotopic glioma mouse models were examined (Fig. 1).

**RESULTS** The resulting nanoparticles are proved to be physiologically stable and biocompatible, allowing time-dependent and intensity-dependent generation of oxygen free radicals upon ultrasound irradiation. Good T1-weighted MR and fluorescence imaging capabilities were demonstrated. We further employed this nanoparticle to treat subcutaneous and orthotopic glioma, demonstrating that SDT with DVDMS-Mn-LPs significantly improved the anticancer effect than that of PDT with DVDMS-Mn-LPsin the presence of skull. Histological analysis further revealed much more apoptotic cells and lower tumor cell proliferation, confirming the advantageous anti-tumor effect of SDT over PDT against glioma.

**CONCLUSIONS** Our study developed a novel theragnostic nanoplatform and provided a promising strategy for imaging-guided SDT for glioma treatment.

**Fig. 1 (abstract P51).**
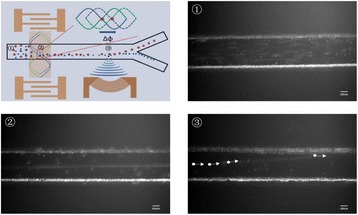
Schematic diagram of experiment. 1. The microspheres injected into the microchannel. 2. The microspheres align in the center of the microchannel by the SSAW 3. The microspheres were driven to the upper wall of the channel by the focused TSAW

### P47 Comparative study on the characteristics of PLGA microspheres with different drugs

#### Dong Yu

##### Chongqing Medical University, Chongqing, China

**OBJECTIVE** To compare the characteristics of PLGA microspheres containing different drugs and green fluorescent dextran, to provide the basis for drug treatment of fungi.

**METHODS** PLGA microspheres were prepared by double emulsification method, and different antifungal drugs were encapsulated, as well as green fluorescent dextran. The experiment consisted of PLGA microspheres, PLGA microspheres containing green fluorescent dextran, PLGA microspheres loaded with amphotericin B and PLGA microspheres loaded with fluconazole. The surface morphology, internal structure, particle size, potential, entrapment efficiency and drug loading were measured and compared.

**RESULTS** (1) The PLGA microspheres were uniform in size, well dispersed and spherical in shape and regular in morphology. (2) The particle size of PLGAmicrospheres was 343.17 ± 44.5nm. (3) PLGA microspheres containing green fluorescent dextran were used as the experimental control group, and the green fluorescence was observed under laser confocal microscope. Indicating that PLGA microspheres successfully encapsulated green fluorescent dextran.

**CONCLUSIONS** PLGA microspheres, PLGA microspheres loaded with PLGA and PLGA microspheres have good physical properties under the same conditions, which lays a foundation for the synergetic effect of microbubbles in the treatment of fungi.

### P48 Therapeutic effect of focused ultrasound combined with anticancer drug loaded microbubble complex for pancreatic cancer: preliminary study

#### Eun-Joo Park, Yun Deok Ahn, Yuri Cheon, Jae Young Lee

##### Radiology, Seoul National University Hospital, Seoul, Korea

###### **Correspondence:** Eun-Joo Park

**OBJECTIVES** As focused ultrasound (FUS) combined with microbubbles has been widely studied in cancer treatment, there is growing interests in developing nanoparticles for FUS enhanced cancer drug delivery. This study was designed to evaluate therapeutic effects of anticancer drug loaded microbubble complex in combination with focused ultrasound treatment for pancreatic cancer.

**METHODS** Immunodeficient mouse inoculated with CFPAC-1 were used as the pancreatic xenograft model for *in vivo* studies. Animals were treated in five groups: control, Doxorubicin-only (Dox), Doxorubicin combined with FUS treatment (Dox-FUS), Doxorubicin loaded microbubble complex (MB-NP-Dox) only, and MB-NPDox combined with FUS treatment (MB-NP-Dox-FUS). Animals were treated on a weekly basis for three weeks and post-treatment monitoring was followed for five weeks.

**RESULTS** As results, therapeutic effects of MB-NP-Dox-FUS will be presented as well as the side effects of each treatment to address the safety of using MB-NP-Dox.

**CONCLUSIONS** Based on this result, further study will be followed to improve therapeutic effects of the combined treatment as well as the drug loading efficiency of MB complex.

### P49 Bio-film mitigated by drug loaded liposomes promoted by pulsed ultrasound

#### Junru Wu^1^, Faqi Li^2^

##### ^1^University of Vermont, Burlington, Vermont, USA; ^2^Chongqing Medical University, Chongqing, China

###### **Correspondence:** Junru Wu

**OBJECTIVES** Biofilm's control is a critical issue for water treatment systems. The primary goal is to use low-intensity pulsed ultrasound to promote liposomes which encapsulate antibiotics into agarose-film based bio-film phantoms and release the encapsulated antibiotics by applying mild-intensity focused ultrasound.

**METHODS** Liposomes were synthesized using 1,2-Diacyl-sn-Glycero-3-Phosphocholine (PC) and 1,2-Dipalmitoyl-sn-Glycero-3-Phosphoethanolamine (DPPE) mixedto a percent molar ratio of 95:5 and dissolved in chloroform in a round-bottomed flask and then were dialyzed against PBS for 24 hours. Dialysate (one liter) was changed 4-6 times, resulting in a relatively pure liposome suspension that encapsulate a gentamicin solution, Liposome size distributions were determined by the autocorrelation function of the dynamic light scattering method. The1.5% sodium alginate solution was prepared by adding 3g alginic acid, sodium salt and 0.6mL glycerol to 200 ml distilled water in a 250ml beaker *via* continuous vortex-mixing overnight. Biofilms were generated using 1x106 Ralstonia insidiosa in 1.5% alginate solution and allowed to stand for 5 minutes to assure that the surface of the solution flat. 600 ml of 2% CaCl2 was added to the alginate from the top for two hours. After polymerization, the film was washed with sterile water and incubated with R2B overnight at room temperature. A two-step treatment was applied. The first step is the penetration of the liposomes into the biofilm. The front part of the transducer was immersed in the liposome suspension. The distance between the transducer surface and the top of the alginate-flm was 1cm.Tone-bursts ultrasound (2.25MHz with duty cycle 10%) were emitted downward into the liposome solution. Experiments were conducted with ultrasound spatially and temporally averaged intensity, ISATA = 0.14 W/cm2.The ultrasound transducer used is a single-element non-focusing piezo-ceramic transducer operated at 2.25 MHz, with active radius a =10 mm. An arbitrary waveform function generator was programmed to produce a tone-burst sinusoidal signal of duty cycle of 10% for 1 min insonation duration, and the output of the waveform generator was used as the input of a 55 dB RF power amplifier whose output was used to drive the transducer. The experiment step two uses a focused ultrasound to burst the liposomes inside the biofilm and release the drug from the liposomes *in situ*. The sample petri dish was mounted and submerged in a tank containing filtered, distilled and degassed water, the position of the sample petri dish can be transported in three orthogonal dimensions by a computer-controlled manipulator with step distance of 1 mm. A piezo-ceramic transducer operating at f0 =1.1 MHz (geometric focal length d = 62 mm, active radius, a = 32 mm) was mounted at the bottom of a tank filled with degassed water facing upward. The distance between the transducer and the biofilm wasadjusted to be equal to the geometric focal length (d=62mm). The same signal generator and amplifier were used, ultrasound tone-bursts (1.1MHz with duty cycle 10%) were emitted upward to the biofilm. The sample was moved in a horizontal plane near the focal plane of the source transducer by a manipulator, the ultrasound focal point was scanned all over the biofilm with 1 mm gap. The spatially- and temporally-averaged acoustic power was measured using the radiation force method to be 60 W/cm2by the measured acoustic power divided the surface area of the transducer. Experiments on a control group were conducted, following the same procedure but keeping the ultrasound off. Additional control was done by repeating the experiments using gentamicin solution alone without liposomes. All treatments were repeated at least three times.

**RESULTS** Bacterial colony forming units were measured. The four different cases include: antibiotic alone shown in Fig. 1 A; ultrasound alone US shown in Fig. 1 B; ultrasound plus the antibiotic drug in solution shown in Fig. 1 C; and focused ultrasound and the antibiotic encapsulated in liposomes shown in Fig. 1 D.

**CONCLUSIONS** Our experimental results have shown that the focused ultrasound can burst the liposomes and release the drugs. Its killing effects were very close to the case that uses the detergent to lyse the liposomes. The focused ultrasound plus gentamicin-encapsulated liposomes (D) reduce the number of viable bacteria residing inalginate based biofilm by 72 percent (p<0.001). Acknowledgment: Funded by NASA (Cooperative agreement number NNX13AD40A) & National Natural Science Foundation of China (Grant Nos. 81127901, 11574039,81201102, 11274404).

**Fig. 1 (abstract P52).**
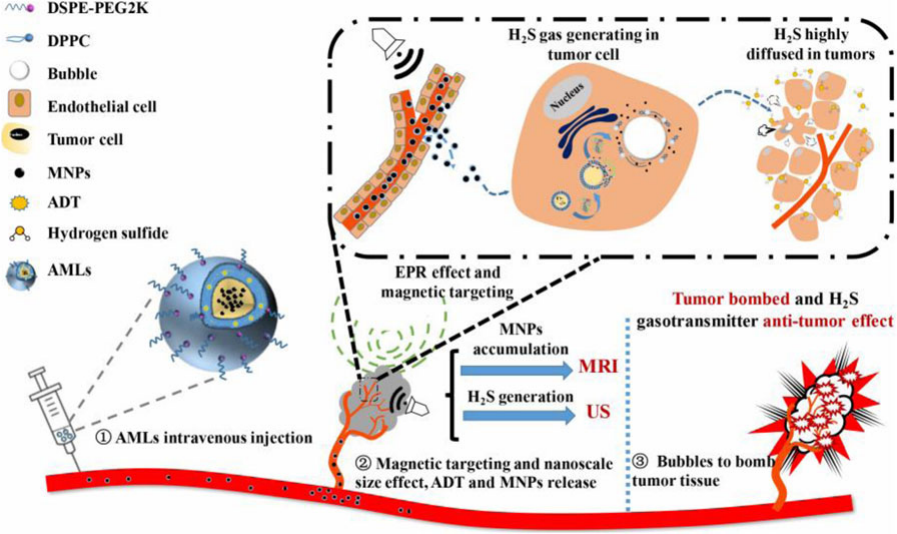
Concepts and schematics of AMLs and their nano to micro conversion for US/MR dual modal imaging and the spatiotemporal­ bombed combination tumoraccurate therapy

### P50 Preparation and characteristics of targeted phase-shift lipid nanoparticles mediated by tumor homing and penetrating peptide

#### Leilei Zhu

##### Ultrasound Imaging, Chongqing, China

**OBJECTIVES** To prepare a novel ultrasound contrast agent, targeted phase-shift lipid nanoparticles mediated by tumor homing and penetrating peptide tLyP-1, and to evaluate its characteristics.

**METHODS** The nanoparticles were prepared by filming-rehydration and acoustic-vibration methods. The morphology, distribution, particle size and zeta potential were detected. After heating and irradiating of low intensity focused ultrasound (LIFU), the phase-shift characteristic and the enhancement effect *in vitro* were observed. The tumor homing and cell-penetrating properties of the nanoparticles were examined by confocal laser scanning microscopy and flow cytometry. The cytotoxicity of the nanoparticles was evaluated by CCK8 assay.

**RESULTS** The size and distribution of nanoparticles were uniformed. The size and zeta potential of nanoparticles were (399.50±29.98) nm and (3.28±1.72) mv, respectively. When the nanoparticles were heated to a temperature of 45 °C or after irradiated by LIFU, nanoparticles generated phase-shift and enhanced ultrasound imaging *in vitro*(P<0.05). The confocal laser scanning microscopy showed that nanoparticles can targetedly aggregate to cell membrane of MDA-MB-231 and penetrate into the cell, but not to HUVEC. The flow cytometry showed that intracellular fluorescence intensity of MDA-MB-231 was higher than that of HUVEC(P<0.05). The CCK8 assay indicated that different concentrations of nanoparticles had no significant effects on cell activity (P>0.05).

**CONCLUSIONS** A novel ultrasound contrast agent, targeted phase-shift lipid nanoparticles mediated by tumor homing penetrating peptide tLyP-1, was prepared successfully. It can target to MDA-MB-231 cell and penetrate into the cell *in vitro*, and enhance ultrasound imaging *in vitro* after LIFU irradiation, which expected to be a novel tumor targeted ultrasound contrast agent and achieve ultrasound molecular imaging at the level of tumor cell.

### P51 Multi-stage surface acoustic wave for separation of cancer cells in microfluidic device

#### Kaiyue Wang, Wei Zhou, lili niu, Feiyan Cai, Fei Li, Long Meng

##### Shenzhen Institutes of Advanced Technology, Shenzhen, China

###### **Correspondence:** Kaiyue Wang

**OBJECTIVES** The separating of circulating tumor cells (CTCs) is significant in the early diagnosis, prognostic judgement, and provides an effective evidences for guideness of clinical chemotherapy. In order to detect CTCs effectively, many separation techniques have been developed to date, including functionalized polymers, pinched flow fractionation, hydrodynamic filtration, inertial microfluidics, deterministic lateral displacement, fluorescent activated cell sorting. With the advantages of non-contact and high throughput, acoustic manipulation has received increasing attention in the cell separation. To ensure the focused cells at a certain position relative to the microchannel, it is essential to align the piezoceramic transducer (PZT) and microchannel precisely, which affects the stability and reliability of the device. In this paper, a microfluidic device with two acoustic stages has been developed to concentrate and separate the cancer cells without the need of the precise alignment and sheath flow.

**METHODS** The standing surface acoustic wave (SSAW) device including a pair of straight interdigital transducers (IDTs) and a pair of circular IDTs was fabricated on the surface of a 128°<span style="font-size:12px; line-height:19.2px"> </span>Y-rotated, X-propagating LiNbO3 substrate. The straight IDTs were used to generate SSAW for cell concentration and the advantages of circular transducers were exploited to generate TSAW for cell separation. The polydimethylsiloxane (PDMS) microchannel was bonded to the SSAW generator using oxygen plasma treatment. And a sample of the mixture of U87 cancer cells and RBCs was used to demonstrate the manipulations of concentration and separation. The frequencies of continuous signals and pulse signal sent to the straight IDTs and the focused IDTs were set to be29.74 MHz and 38.74 MHz, respectively (Fig. 1).

**RESULTS** We examined the ability of the device by manipulating microspheres with about 2μm diameter. When the stream flowed in SSAWs, the microspheres promptly arranged in the center of the microchannel. By modulating the relative phase, △Φ, between two IDTs, the position of the microspheres relative to the microchannel could be adjusted arbitrarily. When the concentrated cells passed through TSAWs, the microspheres were accurately separate into two categories under theaction of radiation force generated by TSAWs, and thus the microspheres separation could be achieved.

**CONCLUSIONS** The experimental results reveal that the multi-stage acoustic-based approach utilizing microfluidic device a promising technique in effectively realizing the concentration and separation of cells. This kind of acoustic-based devices can avoid the cellular damage caused by shear force which is generated by water dynamics. In addition, the use of this method can avoid precise alignment of microchannel and IDTs, improving the stability and practicability of the system.

**Fig. 2 (abstract P52).**
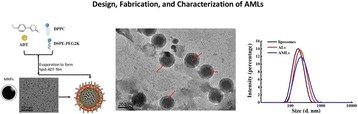
Fabrication and characterization of AMLs and size distribution of liposomes, ALs, and AMLs, respectively

### P52 Magnetic nanoliposomes as *in situ* microbubble bombers for multimodality image-guided cancer theranostics

#### Yang Liu, Fang Yang, Chuxiao Yuan, Mingxi Li, Tuantuan Wang, Ning Gu

##### School of Biological Science and Medical Engineering, State Key Laboratory of Bioelectronics, Jiangsu Key Laboratory for Biomaterials and Devices, Nanjing, JiangSu, China

###### **Correspondence:** Yang Liu

**OBJECTIVES** The physiological barriers imposed by the abnormal tumor vasculature and the dense collagen matrix have prevented nanocarriers from being delivered to unperfused deep tumor regions, which results in weakening the effectiveness of cancer therapeutics. Herein, we designed a nanoliposome drug delivery system aimed at the improvement of nanocarrier accumulation and distribution in the tumor’s poorly accessible regions for excellent diagnosis and potential therapeutic effects for imaging-monitored accurate tumor ablation.

**METHODS** The anethole dithiolethione (ADT) loaded magnetic nanoliposome (AML) delivery system was prepared by a hydration and membrane-filtering method, which consists of ADT, hydrogen sulfide (H2S) pro-drug, doped in the lipid bilayer, and superparamagnetic nanoparticles (MNPs) encapsulated inside (Fig. 1). The size distribution and morphology of the liposomes were characterized (Fig. 2). The generation of H2S gas in HepG2 cells was investigated by a real-time live cell optical imaging system (Figs. 3, 4). High-resolution MRI (7T) and microbubble-enhanced US imaging was performed pre- and post-injection of AML. The therapeutic efficiency of the AML in the tumor-bearing nude mouse model was also evaluated.

**RESULTS** The presence of MNPs trapped and dispersed in the core of the liposome (about 200 nm) was confirmed. The optical microscopic images of a Hep G2 cell showed when incubated with AMLs, a change in cellular morphology and even cell rupture was observed with the increase of incubation time. After 6h of incubation, the cells were disrupted and detached from the dish bottom. For *in vivo* applications, when intravenous injection of AMLs, the targeting of AMLs to tumor was enhanced under exposure to an external magnetic field. Time-dependent *in vivo* MR and US imaging of tumors in a HepG2-bearing mouse model was significantly enhanced. Moreover, after 7-day follow-up observation, AMLs with magnetic field treatments have indicated extremely significantly higher inhibitions of tumor growth.

**CONCLUSIONS** In summary, AMLs are feasible as both synergistic agents to strengthen tumor ablation efficiency and dual-mode contrast agents to provide significant contrast enhancement for MR and ultrasound imaging. This proposed strategy with both enhanced tumor accumulations of liposomes as well as the property of nanoparticles to microbubbles conversion holds great promise for multimodal image-guided accurate cancer therapy.

**Fig. 3 (abstract P52).**
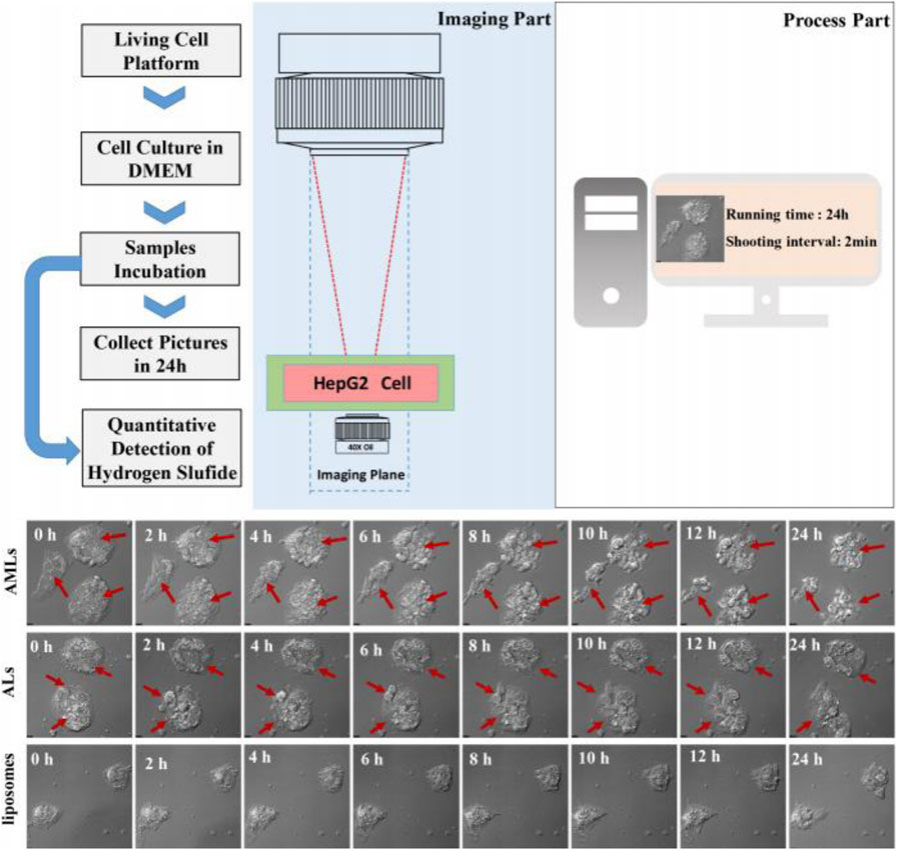
Live cell optical system for observation of cell morphology change after incubation with liposomes, ALs, and AMLs. For each sample, a time gradient was acquired

**Fig. 4 (abstract P52).**
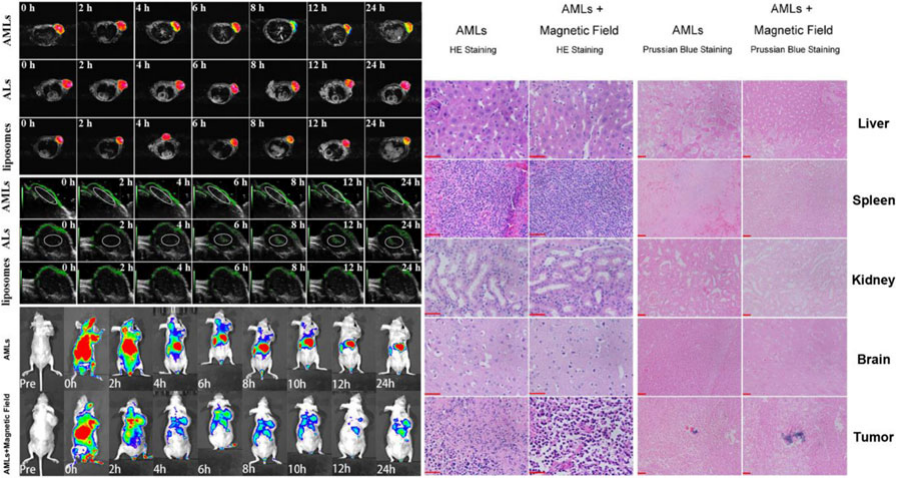
Time­dependent *in vivo* MR, US and NIR­fluorescence imaging of tumors after intravenous injection of samples in a HepG2­bearing mouse model, and histological analysis of excised tumors and major organs after AMLs with and without magnetic field (MF) treatment

**Fig. 1 (abstract P58).**
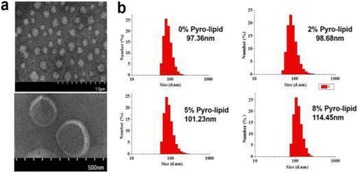
PPL-DOX characteristics. (a) Transmission electron microscopy of PPL-DOX liposome morphology under different magnifications with 2% Pyro-lipid (Molar ratio). (b) Particel size analysis of PPL-DOX with different Mol % porphyrin-phospholipid

**Fig. 2 (abstract P58).**
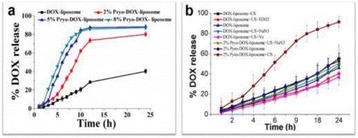
Ultrasound triggered drug release from PPL-DOX with (a) or without (b) free radicals scavengers

### P53 Incubation of free bubble water and phospholipids to prepare shelled nanobubbles ultrasound contrast agents

#### Jilai Tian, Fang Yang, Juan Jin, Ning Gu

##### Biological Science and Medical Engineering, Southeast University, Nanjing, Jiangsu, China

###### **Correspondence:** Jilai Tian

**OBJECTIVES** Nanobubbles less than 1 μm could make a promising application in ultrasound molecular imaging and drug delivery. However, the fabrication of stable gas encapsulation nanobubbles is still challenging. Free gas bubbles without any shell materials may have lots of applications such as water purification, drag reduction, generation of free radicals and so on. Herein, we try to make lipid shelled nanobubbles with free gas bubble water.

**METHODS** Phospholipids dried under vacuum or the lyophilized phospholipids were employed. The characters of size, size distribution, surface tension, viscosity and their ultrasound imaging *in vitro* of the prepared lipids-shelled nanobubbles were investigated. The mechanism of the incubation was also discussed.

**RESULTS** Results show that this type of GU-Liposome has mean diameter of 194.4± 6.6 nm and zeta potential of -25.2± 1.9 mV with layer by layer self-assembled lipid structure. The acoustic imaging analysis *in vitro* indicated that ultrasound imaging enhancement could be acquired by both perfusion imaging and accumulation imaging. The dispersed phospholipid molecular in the prefabricated free nanobubbles water was expected to be assembled to form controllable stable lipid encapsulation gas containing ultrasound-sensitive liposome (GU-Liposome). Compared with conventional mechanical agitation methods, pre-prepared free gas bubble-based nanobubbleshave exhibited the controllable nano-size, lower polydispersity index.

**CONCLUSIONS** Thus, by incubation of free bubble water and phospholipids, stable lipid-shelled nanobubbles could be prepared to broaden the biomedical application of nanobubbles in the theranostics in future.

### P54 Compare ultrasound-mediated cavitation between flowing phase-shift nanodroplets and lipid-shelled microbubbles during focused ultrasound exposures

#### Siyuan Zhang, Tianqi Xu, Zhiwei Cui, Sihao Liu, Dapeng Li, Rui Guo, Junjie Wang, Yujin Zong, Mingxi Wan

##### Department of Biomedical Engineering, Xi'an Jiaotong University, Xi'an, Shaanxi, China

###### **Correspondence:** Siyuan Zhang

**This abstract is not included as it has already been published:**

Zhang S, Cui Z, Xu T, Liu P, Li D, Shang S, Xu R, Zong Y, Niu G, Wang S, He X, Wan M. Inverse effects of flowing phase-shift nanodroplets and lipid-shelled microbubbles on subsequent cavitation during focused ultrasound exposures. Ultrason Sonochem. 2017; 34: 400-409. Available from: http://www.sciencedirect.com/science/article/pii/S1350417716302139.

### P55 Sonodynamic Therapy (SDT) polymer contrast agent for ultrasound/photoacoustic dual-modality imaging-guided synergistic High Intensity Focused Ultrasound (HIFU) therapy

#### Lan Hao

##### Chongqing Key Laboratory of Ultrasound Molecular Imaging, the Second Affiliated Hospital University, Chongqing, China

**OBJECTIVES** To prepare a hematoporphyrin monomethyl ether (HMME)-loaded poly (lactic-co-glycolic acid) (PLGA) microcapsules (MBHMME/PLGA), which could not only function as efficient contrast agent for ultrasound (US)/photoacoustic (PA) imaging, but also as a synergistic agent for high intensity focused ultrasound (HIFU) ablation for its sonodynamic therapy(SDT).

**METHODS** MBHMME/PLGA was prepared with the double emulsion evaporation method by Sonosensitizer HMME nanoparticles were integrated into PLGA microcapsules. After characterization, the cell-killing and cell proliferation-inhibiting effects of MBHMME/PLGA microcapsules on ovarian cancer SKOV3 cells were assessed. The characteristic fluorescence peak of FCLA free acid (reactive oxygen species (ROS)-based chemiluminescence reagent) at around 520 nm was detected with the fluorescence spectrophotometer. The US/PA imaging-enhancing effects and synergistic effects on HIFU were evaluated both *in vitro* and *in vivo*.

**RESULTS** MBHMME/PLGA were highly dispersed with well-defined spherical morphology (462 ± 0.52 nm in diameter, PDI = 0.932). Encapsulation efficiency and drug loading efficiency were 58.33 ± 0.95% and 4.73 ± 0.15%, respectively. The MBHMME/PLGA remarkably killed the SKOV3 cells and inhibited the cell proliferation, significantly enhanced the US/PA imaging results and greatly enhanced the HIFU ablation effects on ovarian cancer in nude mice by the HMME-mediated sono-dynamic chemistry therapy (SDT).

**CONCLUSIONS** MBHMME/PLGA represents a potential multifunctional contrast agent for tumor diagnosis and treatment, which might provide a novel strategy for thehighly efficient imaging-guided non-invasive HIFU synergistic therapy for cancers by SDT in clinic. The mechanism of singlet oxygen could be generated from HMMEand MBHMME/PLGA irradiated with HIFU needed further investigation in the future.

### P56 Preparation of phase-transition perfluoropentane nanodroplets modified by folate for ultrasound molecular imaging in ovarian cancer

#### Jianxin Liu

##### Chongqing Medical University, Chongqing, China

**OBJECTIVES** This study aimed to develop a hybrid platform based on folate-modified phospholipid-shell and perfluoropentane nanodroplets (FA-NDs), which could in vitro and *in vivo* target ovarian cancer and enhance ultrasound imaging after acoustic droplet vaporization (ADV) induced by low-intensity focused ultrasound (LIFU).

**METHODS** The nanodroplet was fabricated with HSPC, DSPE-PEG (2000) folate, DPPG, cholesterol and perfluoropentane using lipid film hydration method and rotary evaporation method. The nanodroplet stability was evaluated at 4 and 37 respectively. The *in vitro* targeted efficiency were tested with SKOV3 cells and *in vitro*ADV was appraised in jellium model with LIFU. The *in vivo* targeted efficiency and acoustic droplet vaporization were evaluated with SKOV3 tumor-bearing nude mice.

**RESULTS** The nanodroplets were successfully prepared with good size uniformity (particle size 321±67 nm). The nanoparticles remained stable for 48 h at 4 and 1 h at37. In vitro targeted experiments exhibited a perfect binding efficiency of FA-NDs to SKOV3 cells. In vitro ADV profiles displayed obvious ultrasound enhancement in both B-mode and CEUS-mode when LIFU power was elevated to 5 W. In vivo and *ex vivo* fluorescence imaging displayed that FA-NDs possessed outstanding specificity to targeted solid tumor. Both the qualitative and quantitative results of *in vivo* ADV in mice tumor nodule manifested that FA-NDs underwent phase transition upon the LIFU exposure.

**CONCLUSIONS** The results demonstrated that the FA-NDs system is a potential platform for targeted conjunction and enhancing ultrasound imaging *via* ADV using LIFU.

### P57 Targeted Pegylated PLGA coated prussian blue nanocomposite for dual-modality PA/MR imaging and synergistic chemo-thermal tumour therapy

#### Tingting Shang

##### Institute of Ultrasound Imaging Department of Ultrasound, The Second Affiliated Hospital of Chongqing Medical University; Chongqing Key Laboratory of Ultrasound Molecular Imaging, Chongqing, China.

**OBJECTIVES** To prepare a PEGylated poly (lactic-co-glycolic acid) (PLGA) targeting with folic acid (FA) coated Prussian blue nanoparticles (PB NPs) and paclitaxel(PTX), to construct multifunctional PLGA-PB-PTX-PEG-FA nanocomposite for both photoacoustic (PA) imaging, magnetic resonance imaging (MRI) and synergistic chemo-thermal tumor therapy.

**METHODS** Paclitaxel (PTX)-loaded Folic acid (FA) targeted PEGylated PLGA nanoparticles encapsulating Prussian Blue (PB) (PLGA-PB-PTX-PEG-FA) nanocomposite was fabricated by a modified double emulsion (water/oil/water) evaporation process. The morphology, size, UV–vis–NIR absorbance spectra, Fouriertransfer infrared (FTIR) spectrum were tested to evaluate the structural characterization of PLGA-PB-PTX-PEG-FA. Drug Loading and releasing of PLGA-PB-PTXPEG-FA nanocomposite were assessed by high performance liquid chromatography (HPLC). Additionally, *in vitro* cell targeting and *in vivo* tumor targeting were verified by confocal laser scanning microscopy (CLSM) and vital fluorescence imaging (VFI) to assess the targeting effect of PLGA-PB-PTX-PEG-FA nanocomposite. The efficacy of the contrast agent for PA imaging and MRI was evaluated by *in vitro* and *in vivo* imaging of subcutaneous MDA-MB-231 tumor-bearing mice following tail intravenous injection of the contrast agent. Finally, to visualize the photothermal cytotoxicity of PLGA-PB-PTX-PEG-FA nanocomposite, MDA-MB-231 cells were incubated with PLGA-PB-PTX-PEG-FA nanocomposite in 96 well-plate for 2 h and were irradiated with or without NIR laser. CCK8 method was used to measure the viability of the cells. To study the photothermal effect and synergistic therapeutic efficacy, PLGA-PB-PTX-PEG-FA nanocomposite suspensions of various concentrations nanoparticle dispersions were exposed to a laser with the wavelength of 808 nm and the output power of 0.647W for 10min *in vitro* or *in vivo* after vein intravenous injection.

**RESULTS** The highly dispersed PLGA-PB-PTX-PEG-FA nanocomposite with spherical morphology with smooth surface was directly observed by TEM and SEM and has good uniformity with an average hydrodynamic diameter of 236.6±55.04nm. That PB NPs being encapsulated by PLGA-FA-PEG was confirmed by TEM, UV–vis–NIRabsorbance spectra and FTIR spectrum of the PLGA-PB-PTX-PEG-FA and PB NPs. Both PB and PLGA-PB-PTX-PEG-FA nanocomposite displayed a broad absorption band from 500 nm to 900 nm with a strong absorption peak at ~702 nm. While the IR spectrum of the PLGA-PB-PTX-PEG-FA nanocomposite showed both the corresponded peaks of PLGA-FA-PEG and PB NPs. The results of drug loading efficiency and drug loading capacity tested by HPLC were 77.82% and 7.22%. The fast drug release from the nanocomposite *in vitro* with laser irradiation indicated the great potential as a controlled-release system for the anticancer drug. In vitro cell targeting and *in vivo* tumor targeting of PLGA-PB-PTX-PEG-FA nanocomposite, targeted group had obvious difference from non-targeted group and blank control group, which showed that good targeting effect to tumor cells or tumor *in vivo* of PLGA-PB-PTX-PEG-FA nanocomposite. After the tail intravenous injection of thePLGA-PB-PTX-PEG-FA nanocomposite, the PA images and MR images of tumor of nude mice were significantly enhanced, while there were almost no distinct enhancement in non-targeted group and blank control group. The result of the photothermal cytotoxicity of PLGA-PB-PTX-PEG-FA nanocomposite showed that the viability of the cells of PLGA-PB-PTX-PEG-FA nanocomposite+Laser group was lowest. The results of photothermal conversion property of PLGA-PB-PTX-PEG-FA suggested the character of photothermal effect was positively correlated with the heating power and the nanoparticles concentration and exhibited good photostability. Upon near-infrared laser irradiation, the PLGA-PB-PTX-PEG-FA nanocomposite showed an enhanced synergistic photothermal and chemical therapeutic efficacy forbreast cancer than solo photothermal therapy or chemotherapy.

**CONCLUSIONS** In conclusion, we prepared highly dispersed core-shell structure PLGA-PB-PTX-PEG-FA nanocomposite by coating PB NPs with PLGA-FA shell and then modifying with PEG. The prepared PLGA-PB-PTX-PEG-FA nanocomposite has good biocompatibility, excellent photo-thermal transformation capacity, and can enhance both PA imaging and MR imaging *in vitro* and *in vivo*. Furthermore, PLGA-PB-PTX-PEG-FA nanocomposite acts as a multifunctional drug delivery system with higher drug loading capacity and excellent drug controlled release-system. Finally, our result confirmed that PLGA-PB-PTX-PEG-FA nanocomposite has the ability of the synergistic therapeutic efficacy combined photothermal and chemical therapy effect, which further enhances the therapeutic efficacy to breast cancer.

### P58 Low intensity focused ultrasound regulates drug release from porphyrin-phospholipid liposomes and facilitates multi-functional theranostics

#### Xiaobing Wang^1,2^, Xiufang Liu^1, 2^, Fei Yan^1^, Hairong Zheng^1^

##### ^1^Shenzhen Institutes of Advanced Technology, Chinese academy of Sciences, Shenzhen, China; ^2^Shaanxi Normal University, Xi'an, China

###### **Correspondence:** Xiaobing Wang

**OBJECTIVES** Nanoscale drug delivery systems (DDS) facilitate multifunctional theranostics. Externally controlled drug release from DDS provides selective targeting. Here, we introduce an ultrasound-activatable porphyrin-phospholipid liposome (PPL) to show its bimodal imaging, robust carrier, drug release behavior and efficient therapeutic actions.

**METHODS** Dynamic light scattering and transmission electron microscopy were utilized to characterize the features of PPL and PPL loaded with doxorubicin (PPLDOX) (Fig. 1). The ultrasound controllable drug release was measured at distinct time points with or without different free radical inhibitors (Fig. 2). In vitro cellular uptake and cytotoxicity were examined in U87 glioma cells by confocal microscopy and microplate reader. In vivo, photoacoustic and NIR fluorescent imaging were applied to indicate the distribution of PPL following intravenous injection, along with the signals change after ultrasonic irradiation (Fig. 3). The tumor inhibition and overall survival timewere evaluated by PPL-DOX endowed with the optimal porphyrin-phospholipid ratio, maximum DOX loading and favorable ultrasound-responsible ability (Fig. 4).

**RESULTS** The obtained PPL, PPL-DOX were stable at 4°C for at least two months with appropriate 100 nm diameter. The ultrasound induced drug release from PPLDOX increased with higher molar phorphyrin-phospholipid, while the drug loading efficient declined as phophyrin-phospholipid ratio increased. 2 molar % porphyrin-phospholipid in PPL-DOX was compatible with good ultrasound-responsible and drug-loading capacities. Under this condition, DOX release from PPL-DOX could beregulated by ultrasound intensity and abolished with different free radical inhibitors. Following intravenous administration, the PPL demonstrates high sensitive photoacoustic and NIR fluorescent imaging and effective tumor site drug delivery in xenograft U87-bearing mice. Ultrasound exposure triggers sonodynamic damage of tumors cells and simultaneously initiates local drug release to exert chemotherapy. Exposure to focused ultrasound with PPL suppresses tumor growth several times more than without exposure to ultrasound. What’s more, PPL-DOX displays little damage on normal cells *in vitro* and even on normal tissues *in vivo*.

**CONCLUSIONS** The developed ultrasound-activable paradigm achieves simultaneous photoacoustic/fluorescence imaging and spatiotemporally regulates nano-drug release and initiates sono-chemotherapeutic effects.

**Fig. 3 (abstract P58).**
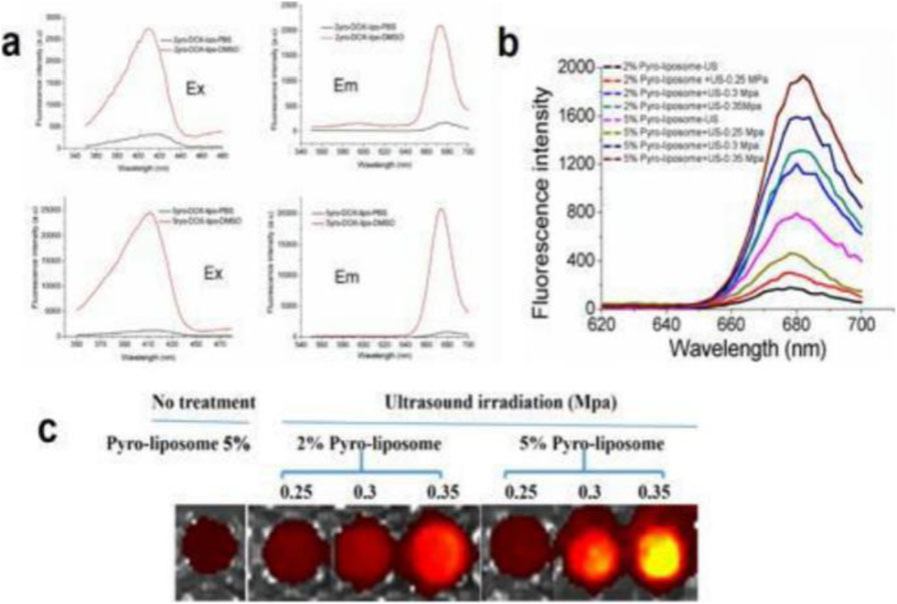
The characterization of PPL flourescene. (a) Flourescene emission of intact quenched PPL (in PBS) versus disrupted unquenched PPL. (b) Flourescene intensity of PPL upon ultrasound irradiation range from 0.25 Mpa to 0.35 Mpa. (c) Flourescene photographs of PPL post ultrasound irradiation was recorded

**Fig. 4 (abstract P58).**
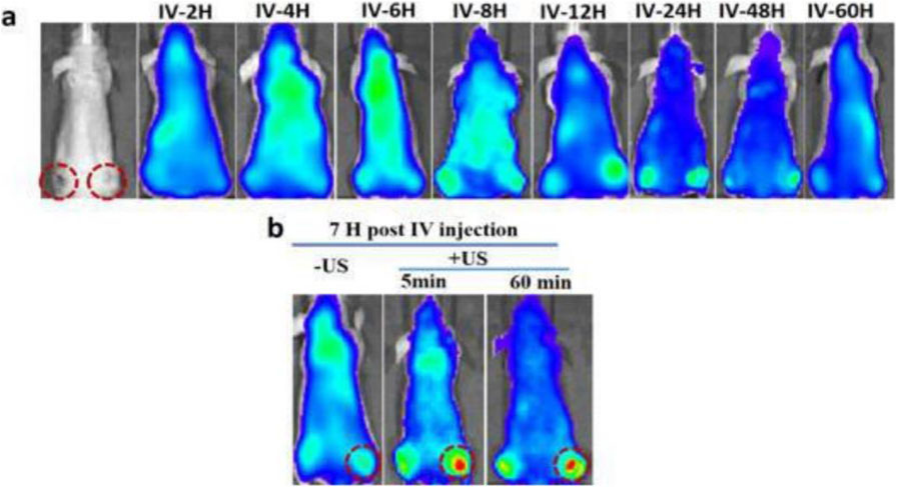
*In vivo* NIR flourescent imaging. (a) The distribution of drug in tumor after I.V. injection. (b) Ultrasound comnbined with microbubbles enhanced the fluorescence of Pyro-liposome, facilitating NIR and photoacoustic imaging and sonochemical therapeutics. A dual tumor model was used, with a tumor on the right flank was irradiated and the other was non-irradiated

**Fig. 1 (abstract P59).**
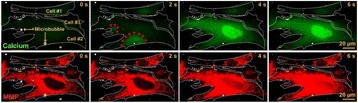
Sonoporation­induced calcium influxes and mitochondria membrane potential changes

**Fig. 1 (abstract P60).**
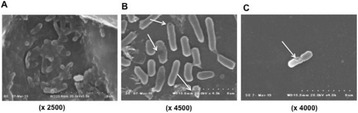
See text for description

### P59 Protective effects of static magnetic field on the sonoporation-induced cellular ionic imbalance

#### Yaxin Hu, Mei Yang, Yancheng Wang, Siping Chen

##### Biomedical Engineering, Shenzhen University, Shenzhen, Guangdong, China

###### **Correspondence:** Yaxin Hu

**OBJECTIVES** During the past two decades, great efforts have been made to investigate the perforation and recovery dynamics of sonoporation-created pores on the plasma membrane (PM). Although both acoustic and non-acoustic parameters have been extensively optimized to facilitate the pore-resealing process, sonoporation mediated intracellular delivery is still challenged by the fact that a significant proportion of the sonoporated cells would undergo apoptosis. It has been hypothesized in previous investigations that excessive ion exchanges across the PM pores could result in a cellular ionic imbalance, which might consequently activate the apoptosis signaling pathway. To provide direct evidence for this hypothesis, this work will study the ion exchange dynamics in sonoporation and its potential relevance to apoptosis induction. Particularly, our work will also examine the question of whether the ion exchange dynamics in sonoporation could be modulated by a magnetic field.

**METHODS** To investigate the ion exchange dynamics across the PM pores, a real-time sonoporation experiment platform was established. More specifically, an adherent microbubble (Targestar® P) was firstly introduced to the apical PM of a single MRC-5 cell. Then, inertial cavitation of the microbubble was triggered by a single ultrasound pulse (center frequency: 1 MHz; peak negative pressure: 0.85 MPa; pulse duration: 10 μs) and monitored by a high-speed camera (imaging rate of 680,000fps). In this way, the ion exchange dynamics in sonoporation could be imaged by a high-resolution confocal microscope (LSM 710). To record the calcium influx insonoporation, a green-fluorescent dye (Fluo 4, AM) was introduced to the cytoplasm. To evaluate the mitochondria damage in sonoporation, a red-fluorescent dye(TMRE) was used to indicate the mitochondria membrane potential (MMP). Nucleic acid stains of Sytox Blue and PI were added to the sonoporated cells at different timepoints (0 s and 10 min post-sonoporation, respectively) to examine the efficiency of PM resealing with and without a static magnetic field (680 mT). Qualitative andquantitative analyses of fluorescent images were carried out using the ImageJ software.

**RESULTS** As illustrated by the green-fluorescent images in Fig. 1, calcium influxes (indicated by red arrows, top row) into the sonoporated cell (cell #2) could be found at the microbubble-adherent sites (2 s post-sonoporation). The intracellular calcium concentration of the sonoporated cells increased to the peak value at 4 s post sonoporation, and the mitochondria membrane potential greatly changes at this time point (red fluorescence, bottom row). In the presence of static magnetic field, both the ion influx speed and the MMP change amplitude were reduced. With respect to the PM resealing, the magnetic field increased the percentage of cells with successful PM recovery by 17.3 % (n=38).

**CONCLUSIONS** Compared with the cellular organelles that are anchored to the cytoskeleton, the freely diffusible ions of the cytoplasm are more vulnerable to the sonoporation-induced disturbance. This study directly demonstrated that the calcium influxes across the PM pores could result in cellular ionic imbalance and mitochondria membrane potential changes. This study contributes to show that static magnetic field could protect the sonoporated cells in the scenario of intracellular delivery of neutrally-charged drugs by reducing the speed of ion exchanges and promoting PM resealing.

**Fig. 2 (abstract P60).**
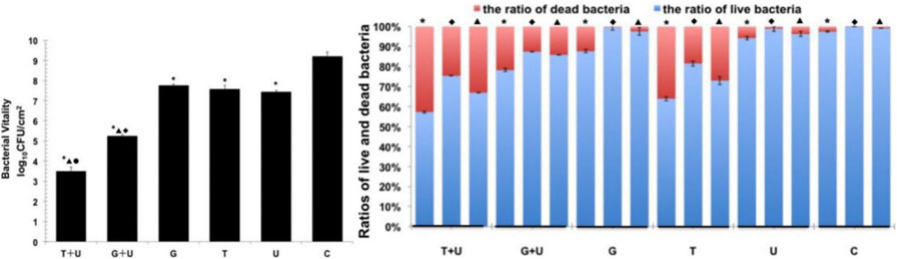
See text for description

### P60 Effects of low-intensity and low-frequency ultrasound combined with antibiotics on biofilms of Extended-Spectrum Beta-Lactamases (ESBLs) producing *Escherichia coli*

#### Hexun Jiang^1,2^

##### ^1^Shanghai First Maternity and Infant Hospital, Tongji University School of Medicine, Shanghai, China; ^2^State Key Laboratory of Ultrasound Engineering in MedicineCo-Founded by Chongqing and the Ministry of Science and Technology, State Key Laboratory of Ultrasound Engineering in Medicine Co-Founded by Chongqing andthe Ministry of Science and Technology, Chongqing, Chongqing, China

**OBJECTIVES** The purpose of this study is to investigate the effects of low-intensity and low-frequency ultrasound (LILFU), when combined with antibiotics, on bacterial viability and the morphology of antibiotic penetration associated with biofilms formed by extended-spectrum beta-lactamases (ESBLs) producing *Escherichia coli* (*E. coli*), a multidrug resistant bacterium strain.

**METHODS** Experimental groups and ultrasound irradiation. The biofilms were divided into 6 groups: the control (C); gentamicin (G) with a working concentrationof 200 μg/ml; tobramycin (T) with a working concentration of 200 μg/ml; ultrasound (U); ultrasound combined with gentamicin (U+G) with a working concentration of gentamicin of 200 μg/ml; ultrasound combined with tobramycin (U+T) with a working concentration of tobramycin of 200 μg/ml.The ultrasound transducer was smeared with ultrasonic coupling gel and attached to the bottom of the 6-well plate. Biofilms of groups U, U+G, and U+T were irradiatedat 42 kHz and 0.66 W/cm2 for 0.5-h, with continuous wave ultrasound. Bacterial plate count. The synergistic effect (SE) was calculated according to Formulation 1 as follow: Synergy Effect (SE) = the inhibitory ratio in the ultrasound combined with antibiotics group- the inhibitory ratio in the antibiotics group. CLSM analysis. Unattached dye was removed from the biofilms by washing. Signals were recorded using green (excitation wavelength at 488 nm and emissionwavelength at 515/30 nm) and red (excitation wavelength at 568 nm and emission wavelength at 600/50 nm) channels. Penetration of antibiotics through ESBLs *E. coli* biofilms. This part of the study was conducted according to the method described by Singh *et al*.

**RESULTS** Bacterial morphology. In group C, bacteria within biofilms were closely linked and wrapped by abundant fiber-like materials, causing the formation ofhighly organized multicellular population structure (Fig. 1A). In group U, however, less fiber-like material was observed in biofilms structure. Some bacterial debris, however, was visible and the bacterial surface exhibited deformation (Fig. 1B and C). Bacterial viability. Bacterial plate counting was performed to evaluate bacterial viability. Compared with group C, bacterial viability in groups U (P=0.002), G(P=0.003), and T (P=0.001) were significantly decreased. Moreover, bacterial viability was even more significantly decreased in groups U+G (P=0.001) and U+T(P=0.001) (Fig. 2). According to Formulation 1, groups U+G and U+T displayed enhanced inhibitory ratios on ESBLs E. coli biofilms of 3.7% and 2.4%, respectively. Assessment of ESBLs E. coli biofilms by CLSM. Results were analyzed using the Image J software. In all groups, the amount of living bacteria was the most in the intermediate layer, relatively less in the inner layer and least in the outer layer of biofilms. In addition, the percentage of dead bacteria in each layer of biofilms in groupsU, U+G, and U+T significantly increased when compared with the same layer of group C. This was especially true in group U+T (Fig. 3). In order to further observe the differences of biofilms morphology and surface viability among these groups, a 3-D surface shape of biofilms was reconstructed with pictures from CLSM. (Fig. 4) Compared with group C, thickness of biofilms in groups U, U+G, and U+T were significantly decreased (P<0.05), while group AP was significantly increased (P<0.05), indicating increased channel gaps in groups U, U+G, and U+T. Additionally, ADD was significantly decreased in groups U+G and U+T (P<0.05), suggesting the blocked nutrient supply of biofilms in these groups. With the exception of group T, TE decreased significantly in other treatment groups when compared with group C (P<0.05), indicating the decrease of uniformity of E. coli biofilms in groups U+G, U+T, and G. Collectively, these results indicate that biofilms morphology and viability suffer damage after ultrasound treatment, either with or without antibiotics. Antibiotic penetration. To clarify the effects of ultrasound and antibiotics on the permeability of ESBLs E. coli biofilms, a ciprofloxacin penetration test was conducted using the penetration model. This data suggests that LILFU can increase the permeability of ESBLs E. coli biofilms to antibiotics. Diameter of inhibition Zone (mm):C:14.0±0.3;G:21.5±0.4*;T:23.6±0.4*;U:26.1±0.2*;U+G:31.4±0.1*;U+T:33.6±0.3*(*Compared with group C, P<0.05. n=6.)

**CONCLUSIONS** In conclusion, LILFU enhances the penetrating capability of antibiotics into ESBLs E. coli biofilms, causing injuries of bacteria within biofilms. Ultrasound, combined with antibiotics, enhances bactericidal effects against biofilms. Our findings may provide a potential therapeutic method for killing bacteria insidebacterial biofilms generated by ESBLs E. coli *in vitro*.

**Fig. 3 (abstract P60).**
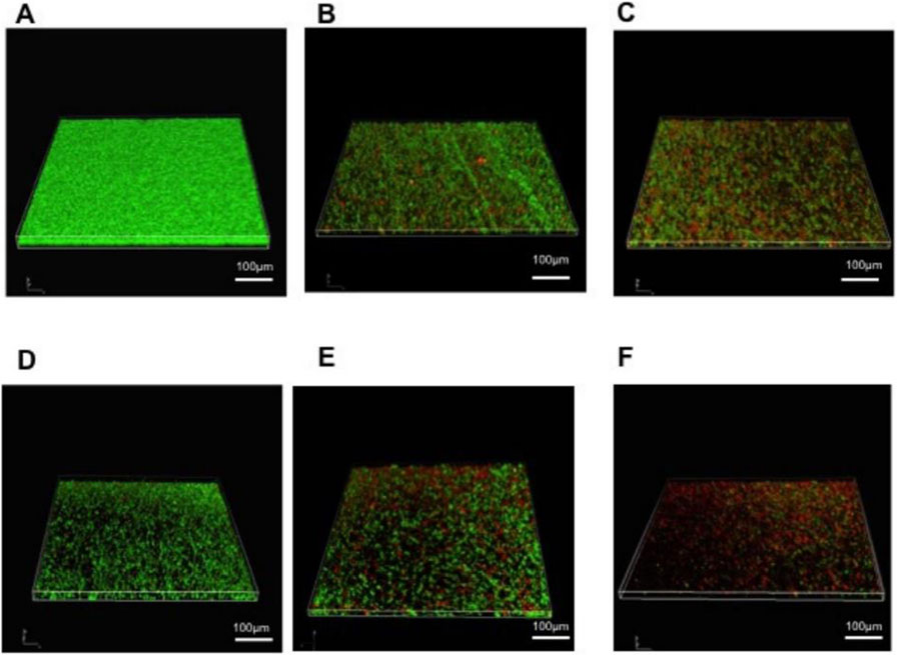
See text for description

**Fig. 1 (abstract P61).**
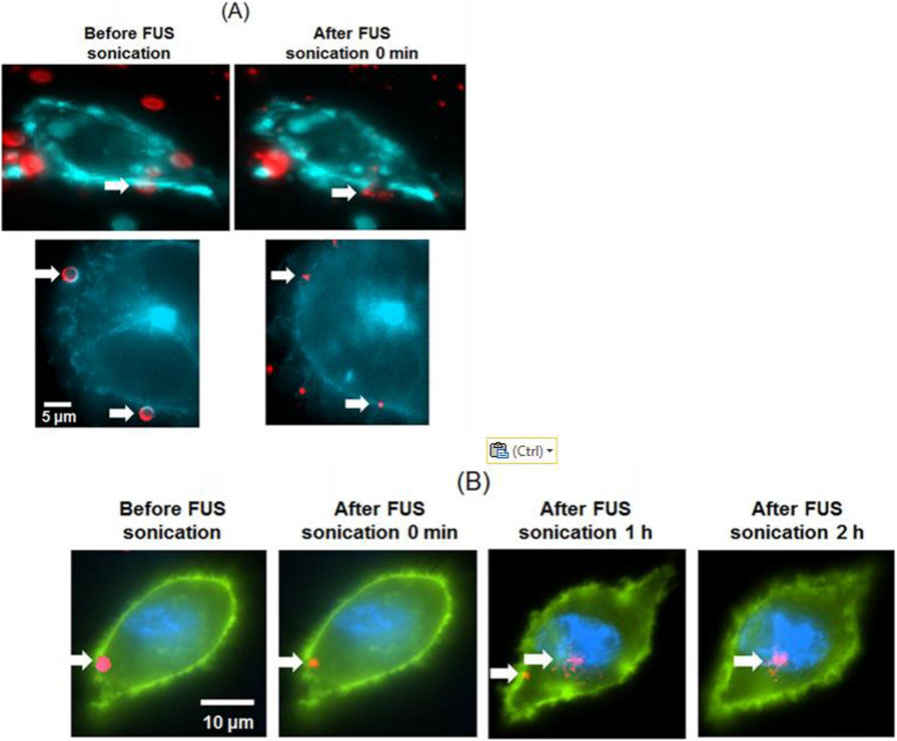
(A) Upper: The 3­D cell morphology reconstruction before and after ultrasound sonication. When DNA­loaded MBs were destructed into fragments, a pore onto cell membrane was generated and the fragments were spread around the site of pore; lower: another case, the debris was co­localized with the cell membrane after ultrasound sonication. (blue: cyan fluorescence protein labeled cell membrane; red: Rhodamine­labeled DNA­loaded MBs) (B) Time­lapse images confirmed the gene was transported to cell nuclei from DNA­loaded MBs after ultrasound sonicaton. (red: Rhodamine­labeled DNA­loaded MBs; green: YFP­labeled cell membrane; blue: DAPIdye labeled cell nuclei)

**Fig. 1 (abstract P64).**
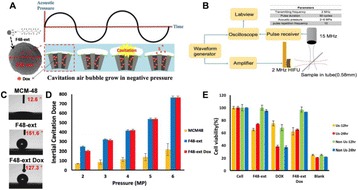
(A) The diagram of controlled drug release from Dox laoded superhydrophobic F48-ext with air layer; (B) the experimental setup of the ICD measurement; (C) the contact angle of the MCM-48, F48-ext with and without Dox; (D) the ICD of the MCM-48, F48-ext and F48-ext Dox; (E) the compare of cell cytotoxicity of F48-ext dox with and without ultrasound exposure

### P61 mechanisms of intracellular gene delivery *via* ultrasound and DNA-loaded microbubbles

#### Ching-Hsiang Fan^1^, Yu-Chun Lin^2^, Chih-Kuang Yeh^1^

##### ^1^Department of Biomedical Engineering and Environmental Sciences, National Tsing Hua University, Hsinchu, Taiwan; ^2^Institute of Molecular Medicine, National TsingHua University, Hsinchu, Taiwan

###### **Correspondence:** Ching-Hsiang Fan

**OBJECTIVES** In the past few years, several studies on ultrasound mediated gene delivery with DNA-loaded microbubbles (MBs) is vastly expanding since it’s noninvasive and non-viral vector properties. Nevertheless, little is known on the machinimas of enhanced cellular gene delivery following ultrasound with DNA-loaded MBs interaction. In this study, we tried to image the MBs and cells during ultrasound sonication for revealing the short-term and long-term mechanisms of intracellular gene delivery, including sonoporation, endocytosis, and lipid fusion.

**METHODS** DNA-loaded MBs were synthesized to have cationic phospholipid shell with C3F8 gas core, with DNA (green fluorescence protein plasmid) loaded via electrostatic interactions. The lipid shell of DNA-loaded MBs was modified with folate acid for binding with the folate acid receptor of C6 glioma cell. The measured mean concentration and size of DNA-loaded MBs were (2.1 ± 0.1) × 1010 MB/mL and 1.0 ± 0.2 μm, respectively, measured by multisizer. The DNA loading efficiency was 27.1 ± 3.1 %. Ultrasound exposure (frequency = 1-MHz, acoustic pressure = 0.5 MPa, cycle number = 500, pulse repetition frequency = 10 Hz, sonication time = 10s) was performed with presence of DNA-loaded MBs to achieve gene delivery with C6 glioma cells. To monitor the DNA trafficking from MBs into cell nuclei, the DNA, cell membrane and cell nuclei were fluorescently labelled with Rhodamine, yellow fluorescence protein (YFP) and DAPI dye, individually, and imaged by a live-cellmulti-color fluorescent microscope before and after ultrasound sonication. During the imaging, the cells were kept in a humidified atmosphere with 5% CO2 at 37 °C.

**RESULTS** The 3-D cell morphology reconstruction data show that a pore (Fig. 1A, white arrows) on the cell membrane was immediately formed when the DNA-loadedMBs (spherical red structure) are disrupted into fragments (red spot) by ultrasound sonication. We also observed that the debris of DNA-loaded MBs spread around the site of pore or co-localized with the cell membrane (white arrows), indicating that the DNA-loaded MBs vesicles might through sonoporation or lipid fusion pathways to delivery gene into the cells. The time-lapse images (Fig. 1B) also confirm that the gene indeed was transported and internalized into cellular nuclei from DNA-loaded MBs following ultrasound sonication.

**CONCLUSIONS** This study demonstrated the intracellular DNA trafficking from DNA-loaded MBs into cellular nuclei might rely on sonoproation, endocytosis, and lipid fusion. Future works include optimize the parameters of ultrasound and DNA-loaded MBs for improving the efficiency of gene delivery.

**Fig. 1 (abstract P67).**
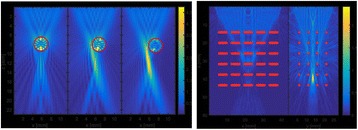
See text for description

### P62 Acoustic vaporisation of encapsulated droplets

#### A. Podkovskiy^1,2^, C. Olivier4, J.L. Thomas^4^, M. Guedra3, F. Coulouvrat^3^, T. Lacour^3^, Wladimir URBACH^1, 2^, N. Taulier^2^

##### ^1^ENS Paris, Lab. Phys. Stat., Paris, France; ^2^LIB, Sorbonne Universités UPMC and CNRSLIB, 75005-Paris, France; ^3^Sorbonne Universites, UPMC Univ Paris 06and CNRS UMR 7190F-75005 Paris, Institut Jean Le Rond d’Alembert, Paris, France; ^4^INSP, Sorbonne Universités UPMC 75005 Paris, France

###### **Correspondence:** A. Podkovskiy

**OBJECTIVES** The use of encapsulated droplets is currently largely investigated for medical applications, mainly because their reduced size allows them to enter targeted areas that cannot be reached by large microbubbles (contrast agents). Perfluorocarbon droplets can be vaporized under the action of an ultrasonic field, in order to turn them into echogeneous eventually cavitating microbubbles. The optimization of acoustic droplet vaporization (ADV) will be enhanced by understanding the physical mechanisms underlying ADV, which are currently not totally elucidated.

**METHODS** A recent model (JASA p3656–3667 (2015) doi:10.1121/1.4937747) describes this phenomenon paying particular attention to the finite size of the droplet and its encapsulation by a thin viscoelastic layer. Numerical simulations, done for droplets of micrometric radii and for frequencies of 1–5 MHz, reveal that droplet surface tension and shell rigidity are responsible for an increase of the acoustic droplet vaporization threshold. This threshold does not vary monotonically with frequency and thus an optimal frequency can be found to minimize it. To check the above theoretical results, we investigated *in vitro* the relationship between ADV and Inertial Cavitation.

**RESULTS** The threshold pressures required to induce ADV and IC were simultaneously determined for micron-sized PFC droplets as a function of droplet size and US parameters. Experimental conditions that yield ADV without IC and ADV with IC, that could enhance drug delivery *via* sonoporation, are determined by investigating parameters that could influence both thresholds; bulk fluid properties such as gas saturation, temperature, viscosity, and surface tension; droplet parameters such assurfactant type; and acoustic properties such as pulse repetition frequency and pulse width.

**CONCLUSIONS** In conclusion, our experiments allowed us to test a new model of droplets vaporization using ultrasound in an infinite medium. The study of droplets vaporization, located in confined medium more resembling the situation *in vivo*, will be the next step.

### P63 Ultrasound theranostics for tumor

#### Kazuo Maruyama

##### Faculty of Pharma-Sciences, Teikyo University, Tokyo, Japan

**OBJECTIVES** “Theranostics” is a treatment strategy that combines therapeutics with diagnostics. The combination of bubble formulation and ultrasound (US) is a good tool for “theranostics” due to have multi-potency both of diagnostics with enhanced echo signal from bubble and therapeutics with cavitation of bubble. To develop a novel bubble formulation for US imaging and therapy with small particle size and a good stability and test the formulation as US imaging contrast agent and for gene delivery *in vitro* and *in vivo*. Interleukin12 (IL-12) exhibits immunomodulatory antitumor effects and is considered an effective antitumor agent, but its short half-life and systemic toxicity following intravenous injection are major obstacles to its therapeutic use. Therefore, we transfected pDNA encoding the IL-12 gene (pCMV-IL-12) into tumor tissue using bubbles and US with the aim of achieving high local expression of IL-12.

**METHODS** Lipid-stabilized bubbles were prepared by homogenization of a lipid dispersion in the presence of perfluoropropane gas. Different phospholipid compositions were tested and evaluated. After bubble formation the bubbles were freeze-dried so that a dry sample containing bubbles was formed. After re-constitution of the samples they were analyzed for size, gas content and US signal intensity. The ultrasound theranostics capabilities of bubbles for the solid tumor were studied in Colon26 tumor bearing mice. Bubbles was injected to mice *via* tail vein and 9 MHzlinear ultrasound was exposed to solid tumor site transdermally. Following the recognition of neovasculature in tumor tissue, 1 MHz therapeutic ultrasound was exposed transdermally over the site of solid tumor tissue.

**RESULTS** Bubbles was injected to mice *via* tail vein and 9 MHz linear ultrasound was exposed to solid tumor site transdermally. The flow of bubbles in blood was observed and neovasculature of tumor tissue was imaged clearly. Following the recognition of neovasculature in tumor tissue, 1 MHz therapeutic ultrasound was exposed transdermally over the site of solid tumor tissue. This process induced cavitation of bubbles in the tumor tissue, resulted in rising the temperature of tumor tissue to 45-55C, and also significant reduction of tumor growth. Cavitation leads to localized heating and cloud be use for ablative cancer therapy. Transfection of pCMV-IL-12 with LBs and US suppressed tumor growth significantly. To investigate the mechanism behind the anti-tumor effects of pCMV-IL-12 transfected using bubbles and US, we assessed the involvement of CD4+ and CD8+ T cells and NK cells. The depletion of CD8+ T cells effectively blocked the antitumor effect of pCMV-IL-12 transfected using bubbles and US. These results suggest that the combination of bubbles and US can effectively induce sufficient IL-12 expression to cause anti-tumor immune responses.

**CONCLUSIONS** The combination of bubbles and US could be efficacious for neovasculature image and cancer therapy. We believe this new formulation shows great promise for both diagnostic and therapeutic applications thanks to its good stability, relatively small bubble size and the simplicity of handling.

### P64 DOX-loaded superhydrophobic mesoporous silica nanoparticles with ultrasound for dual-treatment in tumour

#### QiaoFeng Jin, Cheng-Han Wu, Chih-Kuang Yeh

##### Department of Biomedical Engineering and Environmental Sciences, National Tsing Hua University, Hsinchu, Taiwan

###### **Correspondence:** QiaoFeng Jin

**OBJECTIVES** Mesoporous silica nanoparticles (MSNs) have great potential for biomedical applications. Surface air bubbles adsorbed on the fluorine-modified superhydrophobic MSNs could be served as cavitation nuclei and prevent the leakage of chemotherapy drugs encapsulated into MSNs during circulation. Combination of superhydrophobic surfaces with ultrasound can cause cavitation to induce cell damage and facilitate drugs release. In this study, we propose a dual-treatment strategy by combining active cavitation and chemotherapy.

**METHODS** Synthesis of superhydrophobic F48-ext and Dox loaded F48-extThe parent MCM-48 type MSNs were synthesized with sol-gel method using BCDAC and C16E2 as soft templates. To fabricate fluorinated MCM-48 (names as F48-ext), the MCM-48 were dispersed in 10 mL toluene containing 0.2 ml of perfluorodecyltriethoxysilane and stirred at 100° for 48 hours, and the resulted solid was collected and dried at 60° for 12 hours. Finally, F48-ext NPs were obtained after removing the surfactants by repeated ion exchange in a dilute HCl-ethanol solution at 60°. The DOX was loaded into F48-ext NPs in ethanol which contains 2% (v/v) concentrated hydrochloric acid, then the ethanol were vaporized and replenished with 1ml ethanol contain 2% hydrochloric for thrice. Inertial cavitation dose measurement using a passive cavitation detectorA 15-MHz focused transducer was used as a passive cavitation detector to receive inertial cavitation (IC) signals, while a 2-MHz HIFU transducer was used to excite theNPs. The integrated value of the amplitude of frequency spectrum from 10-20 MHz was termed inertial cavitation dose (ICD) and used to assess the IC intensity. In vitro cell cytotoxicity with alamar Blue® with and without US exposureTRAMP cells were incubated with Dox-loaded F48-ext NPs, free F48-ext NPs and free Dox for 2 hours in 24 wells plate, respectively and then all samples were sonicated at 5 MPa pressure with a PRF of 100 for 5 min by the 2-MHz transducer. The cells without ultrasound sonication were set as control group. After 3 hours, the samples were washed and further incubated for 24 hours, and their cytotoxicity were measured by using alamar Blue® kit.

**RESULTS** MCM-48 and F48-ext NPs show average sizes of 200-300 nm, and show the zeta potentials of -30 mV, which contributed to the stability of such NPs. The F48-ext NPs had a contact angle of about 150°, and after loading Dox their contact angle decreased to 130° due to the hydrophilic Dox. The ICD of F48-ext NPs have significantly augmented with respect to that of MCM-48 NPs (Fig. 1). The ICDs of F48-ext and Dox-loaded F48-ext NPs both increased with acoustic pressures. Due to the superhydrophobic modification, the Dox leakage of F48-ext NPs was suppressed. We found that Dox releasing of F48-ext NPs was not increased with ICD increasing. For the short term, the cells damage caused by inertial cavitation of F48-ext NPs was observed and dominated the cell cytotoxicity. The Dox releasing from the F48-extNPs performed long term treatment mode.

**CONCLUSIONS** In summary, the degree of inertial cavitation of MSNs increased after fluorine-modification (F48-ext), and the Dox-loaded into F48-ext NPs shows better stability than MCM-48 NPs. In addition, DOX loading did not inhibit the IC activity of F48-ext NPs. Combination of mechanical effect and chemotherapy have shown the tumor dual-treatment mode. We demonstrated that F48-ext NPs were sensitive to ultrasound and with a stable drug-loading capacity.

**Fig. 1 (abstract P72).**
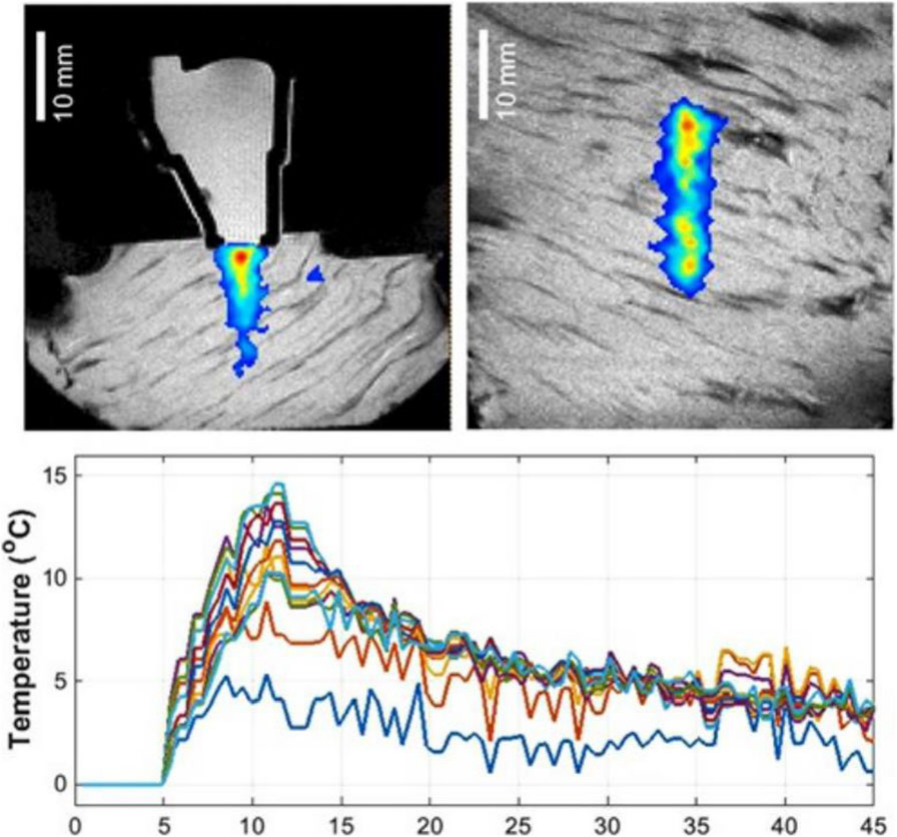
MRI image of applicator in pork muscle (axial section) after 7s showing thermal profile, and calculated temperature rise

### P65 The influence of exercise on uterine fibroid and adenomyosis after high-intensity focused ultrasound

#### Huang Xueyan

##### Biomedical Engineering, Chongqing, China

**OBJECTIVES** To evaluate exercise can improve clinical symptoms of uterine fibroid and relief dysmenorrhea of adenomyosis after high-intensity focused ultrasound (HIFU) treatment.

**METHODS** From January 2011 to August 2015. 83 patients suffered from uterine fibroid and 102 patients suffered from adenomyosis. All patients were treated withHIFU.Among the 185 patients, 83 of them completed one-year follow-up,102 patients of them completed half a year follow up. All the symptom improvement the uterine fibroid volume and the standards of Visual Analogue Scale(VAS) were recorded.

**RESULTS** In this study, 83 patients had uterine fibroid,51 of 83 patients were no exercise intervention group, the rest of 83 patients were exercise intervention group,Comparing with this two groups’ uterine fibroid change,P=0.003,considered as statistically significant.That meaning, exercise could help improve the absorption of uterine fibroid.102 patients had adenomyosis,45 of 102 patients were no exercise intervention group,57 of 102 patients were exercise intervention group.From the result found that exercise may relief the score of dysmenorrhea,P=0.002,considered as statistically significant.

**CONCLUSIONS** Based on the observations from uterine fibroid and adenomyosis, exercise have positive reaction on improving symptoms of uterine fibroid andadenomyosis. Hence, this clinical effective and response is a further treatment for woman who wants to protect the uterus.

### P66 Patient selection by screening pelvic MRI in MR-guided focused ultrasound surgery of uterine fibroid

#### Junhai Zhang^1^, Xiaoxia Liu^2^, Hairui Xiong^1^, Zhenwei Yao^1^, Qian Zhou^1^, Haoxiong Li^1^, Ying Tang^1^

##### ^1^Department of Radiology, Huashan Hospital, Fudan University, Shanghai, China; ^2^Department of Gynecology, Obstetrics & Gynecology Hospital, Fudan University, Shanghai, China

###### **Correspondence:** Junhai Zhang

**OBJECTIVES** To give advice about how to select suitable patients in MR-guided focused ultrasound surgery (MRgFUS) of uterine fibroid by screening pelvic MRI.

**METHODS** Retrospective analysis the MRI image and clinical data of 30 fibroid patients who successfully complete the MRgFUS treatment in Huashan hospital and also review the literature.

**RESULTS** The safe and effective use of MRgFUS is affected by fibroid type and location, position relative to adjacent anatomical structures and the presence of coexistent pelvic disease.

**CONCLUSIONS** Screening pelvic MRI for selection of patients in whom sufficient fibroid volumes can be treated safely using the MRgFUS system is critical forsuccessful outcomes.

### P67 Effect of fiducial markers and brachytherapy seeds on the delivery of HIFU salvage therapy in the prostate

#### Panayiotis S. Georgiou^1^, Jiri Jaros^2, 1^, Heather Payne^3, 1^, Clare Allen^3^, Taimur T. Shah^4^, Hashim Ahmed^4^, Eli Gibson^1^, Dean Barratt^1^, Bradley Treeby^1^

##### ^1^Medical Physics and Biomedical Engineering, University College London, London, UK; ^2^Computer Systems, Brno University of Technology, Brno, CzechRepublic; ^3^Oncology, University College London Hospitals, London, UK; ^4^Surgery and Interventional Science, University College London, London, UK

###### **Correspondence:** Panayiotis S. Georgiou

**OBJECTIVES** Prostate cancer is the most common cancer and the second leading cause of cancer-related death in men in Europe and North America. For patients with early-stage localized disease, the cancer is often treated using external beam radiation therapy (EBRT) or brachytherapy. In EBRT the radiation treatment is delivered from a source external to the patient’s body and the procedure usually involves implanting a small number of gold fiducial markers into the prostate to verify the position of the prostate gland between treatments. In brachytherapy the radiation dose required for the treatment is delivered locally by implanting a large number of radioactive seeds (approximately 100). In low dose rate (LDR) brachytherapy these seeds remain permanently in the patient’s body. For some of these patients, treated either by EBRT or LDR brachytherapy, their cancer will recur. In such cases, further treatment using an alternative (salvage) therapy can be considered. High intensity focused ultrasound (HIFU) is currently offered in hospitals as a minimally invasive salvage therapy for treating prostate cancer inpatients whose cancer has recurred. However, clinicians observe mixed results with salvage-HIFU for failed brachytherapy, which may be partly linked to inadequate heating caused by the implanted seeds. To date, the impact of implanted EBRT markers and brachytherapy seeds on HIFU treatment has not been thoroughly studied. The objective of this work was to investigate the effect of a single EBRT marker on the efficacy of HIFU treatment delivery and extend these results to a large number of brachytherapy seeds.

**METHODS** Using the k-Wave acoustics toolbox, we have performed coupled acoustic-thermal modelling of HIFU treatments by first modelling the prostate with the presence of a single EBRT gold marker at different positions and then, with the presence of multiple brachytherapy seeds. The medium properties were obtained from book values. The transducer model was based on the specifications of the Sonablate 500 HIFU probe. The distribution of the seeds was extracted from patients’ medical images. The target lesion volume, location and order of sonications were selected based on standard treatment protocols. The ablated lesion volume was estimated from the model data using a thermal dose metric based on cumulative equivalent minutes.

**RESULTS** The simulation results show that the EBRT marker obstructs the propagation of the ultrasound beam and distorts the focal volume when positioned within 5mm of the focus (Fig. 1). The distortion significantly increases when a large number of seeds is introduced in the model. Both the seeds and the markers act as scatterers, reflecting energy away from the intended focus. This leads to two undesired implications. Firstly, the intended treatment region receives less energy and as a result thetissue is not adequately heated. Secondly, the wave reflected by the seeds induces heating at unintended regions, which often include healthy tissue and the rectal wall.

**CONCLUSIONS** The distortion introduced due to the presence of the EBRT marker, and more importantly, due to the presence of the brachytherapy seeds may result in undertreated regions due to less energy arriving at the focus or overtreated regions due to reflections which may affect organs at risk. Depending on the position of the targeted regions and the distribution of the markers or seeds, both effects may be undesirable. The results presented have particular importance for patient selection and treatment planning for prostate salvage-HIFU after failed EBRT or brachytherapy and indicate the necessity to reconsider the treatment parameters used during theseprocedures.

**Fig. 2 (abstract P72).**
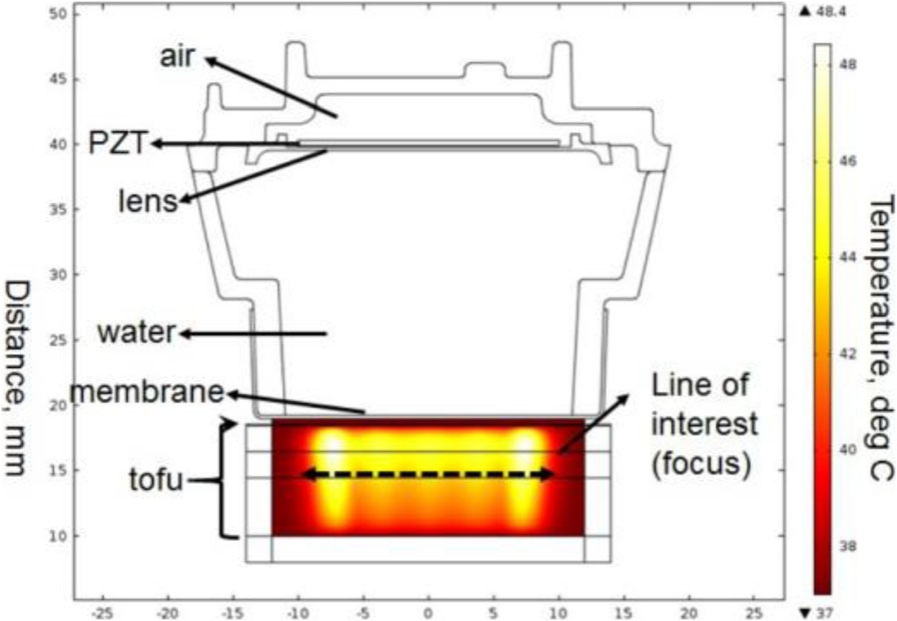
Simulated thermal model of TMM (tofu), showing ultrasound applicator and temperature distribution after 7s (longitudinal section)

### P68 The safety and short-term efficacy of MR guided focused ultrasound surgery for bone metastases-induced pain palliation

#### Hairui Xiong^1^, Qian Zhou^1^, Junhai Zhang^1^, Haoxiong Li^1^, Ye Chen^1^, Qiong Li^2^, Ying Tang^1^, Zhenwei Yao^1^, Xiaoyuan Feng^1^

##### ^1^Department of Radiology, Huashan Hospital, Fudan University, Shanghai, China; ^2^Department of Anesthesiology, Huashan Hospital, Fudan University, Shanghai, China

###### **Correspondence:** Hairui Xiong

**OBJECTIVES** To discuss the safety and short-term efficacy of MR-guided Focused Ultrasound Surgery (MRgFUS) for pain palliation of bone metastasis patients.

**METHODS** 14 patients with painful bone metastases were recruited in a prospective study. The treating efficacy is characterized by Numerical Rating Scale (NRS), theBrief Pain Inventory Quality of Life (BPI-QOL) survey, and Karnosky Performance Status Scale (KPS). the adverse events occurred pre and post-treatment were analyzed. Normal distributed statistics were analyzed with paired-samples t test, or Wilcoxon rank sum test was used.

**RESULTS** 14 patients were treated with MRgFUS. two patient dropped out of the study. The NRS ratings are 6.5(4), 5(5.25), 2.5(5), 2.5(4.75) for pre-treatment, one week, one month, two months, and three months, respectively. Such variances of NRS ratings are statistically significant (P<0.05). The BPI-QOL ratings are 42.42±8.27,30.67±12.29, 29.17±15.38, 29.92±17.67, 35.67±19.28, respectively. The BPI-QOL ratings decrease in the first two months after the treatment which is statistically significant (P<0.05) ; whereas for the third month, the BPI-QOL rating is statistically insignificant compared with the one before the treatment. The KPS ratings are80(28), 80(20), 65(45) for pre-treatment, one week and three months, respectively. Three months after the treatment, the KPS ratings decreases which is statistically significant compared with the one before the treatment (P<0.05). After the treatment, one patient developed deep venous thrombosis; three patients reported lower extremities numbness, two patients had soft tissue edema around the lesions.

**CONCLUSIONS** MRgFUS is effective for short-term pain palliation of bone metastases. Such noninvasive technique is safe and can improve patients’ living condition.

### P69 Characterization and validation of a 64-element Capacitive Micro-Machined Ultrasound Transducer (CMUT) annular array with a 256 element imaging array in the same plane, capable of tissue ablation of the prostate

#### Christopher Bawiec^1, 2^, William Apoutou N'Djin^1, 2^, Guillaume Bouchoux^1^, Nicolas Sénégond^3^, Nicolas Guillen^4^, Jean-Yves Chapelon^1, 2^

##### ^1^Inserm, U1032, LabTau, Lyon, France; ^2^Université Lyon 1, Lyon, Rhone-Alpes, France; ^3^Vermon, Tours, France; ^4^Edap TMS, Vaulx-en-Velin, France

**OBJECTIVES** The goal of this work was the validation and characterization of a recently developed CMUT probe that is capable of delivering a therapeutic ultrasounddose while simultaneously enabling the delivery to be monitored through ultrasound imaging. The primary application of this probe is the treatment of prostate cancer forthermally ablating the cancerous tissues. The currently used probe is a high intensity focused ultrasound (HIFU) transducer utilized treatment of prostate cancer. Thesedevices are fabricated using spherically focused piezoelectric materials, however, there are drawbacks to the current design. In this work, CMUT probes were investigatedfor the HIFU applications as they offer potential advantages over the current designs allowing decreased fabrication costs at an industrial scale and ease ofminiaturization.

**METHODS** A planar ultrasound probe consisting of a 64-element CMUT annular array around a linear 256-element CMUT imaging array was studied in simulation and experimentally (impedancemetry, microscopy and vibrometry as well as hydrophone measurements and *in vitro* lesion formation). Numerical models of the therapeutic acoustic field were performed using the Rayleigh integral to determine the feasibility of electronic focusing and inducing thermal lesions. For comparison, simulations of a geometrically focused existing piezo-based technology (16-element annular array) was also performed. Experimentally, key static parameters (collapse-, snapback-, andbreakdown voltages) for use in the different modes of operation (conventional, collapse, and collapse-snapback) were identified. The HIFU capabilities of the device were also investigated experimentally (pressure field and radiation force measurements) with the creation of *in vitro* lesions. The imaging capabilities were also compared between the existing device and the prototype in order to validate the modelling.

**RESULTS** Simulations showed that the planar CMUT design could dynamically focus from 3-7cm and create lesions comparable in size and shape to the geometrically focused transducer. Microscopy allowed visualization of the static collapse and snapback of individual cells which was confirmed in parallel with impedance measurements. Collapse occurred at 132.5±5V and snapback occurred at 95±5V. Dynamic behavior of the CMUT (vibrometry) in air and in water allowed identification of the collapse-snapback operational mode. In this mode, the probe was capable of generating up to 5W/cm2 surface intensity. Furthermore, experimental validation performed on the probe utilizing hydrophones and *in vitro* tissue confirmed that the probe was capable of creating lesions in tissue.

**CONCLUSIONS** The results of the investigation confirmed that the current prototype is capable of creating lesions in biological tissue. Both the simulations and the experiments were able to confirm the capability of this ultrasound probe. The ultrasound imaging part of the probe also provided an enhancement to the spatial resolution of the existing technology which allowed for a higher resolution improving the localization of the treatments. This new ultrasound probe prototype has the potential of providing an improved treatment method of prostate cancer that can increase the quality of the treatment and subsequent outcome of the patient while adding the benefits of a reduction in the cost and size of the probe. This project was supported by the French Single Interministerial Fund (FUI, 2013).

### P70 Precision microvascular therapy *via* the synergy of light and sound

#### H. Zhang^1,2^, Z. Hu^2,3^, J. Li^2,3^, Q. Liu^3^, S. Yuan^3^, Y. Paulus^4^, X. Yang^5^, and X. Wang^1,2^

##### ^1^Institute of Acoustics, Tongji University, Shanghai, P.R. China; ^2^Department of Biomedical Engineering, University of Michigan, Ann Arbor, MI, USA; ^3^Department of Ophthalmology, the First Affiliated Hospital of Nanjing Medical University, Nanjing, P.R. China; ^4^Department of Ophthalmology and Visual Sciences, University of Michigan, Ann Arbor, MI, USA; ^5^Department of Mechanical Engineering, University of Kansas, Lawrence, KS, USA

###### **Correspondence:** H. Zhang

Angiogenesis and neovascularization are hallmarks for a variety of pathological conditions, including cancer and many eye diseases, and play a crucial role in disease onset and progression. Antivascular therapies that aim at either removing microvessels or slowing down their growth represent a proven new strategy to intervene the progress of these conditions and improve the prognosis. Here we report the development of a photo-mediated ultrasound therapy (PUT) technique as a new concept of localized antivascular therapy. Unlike any of the previous optical- or ultrasonic-alone techniques, laser pulses and ultrasound bursts are synergistically applied in PUT, which facilitate noninvasive treatment of subsurface microvessels with unprecedented high precision. PUT takes advantages from the high native optical contrast among biological tissues, and has the unique capability to self-target on blood vessels without causing unwanted damage to surrounding tissue. As demonstrated through the experiments on animal models, PUT can treat microvessels in target tissue *via* different mechanisms, including blocking local vessels by inducing blood clots or disrupting vessels causing local hemorrhage, each with values in clinic. Moreover, PUT working at different optical wavelengths can selectively treat veins or arteries by utilizing the contrast in optical spectra between deoxy- and oxy-hemoglobin. PUT, as a novel antivascular therapy technique with the capability to precisely target vessels and precisely control the treatment effects, holds promise to impact clinical management of cancer and eye diseases by delivering optimized treatment outcome with minimized complication.

### P71 Effect of ambient pressure on cavitation bubbles at the focal point of a spherical resonator with open ends

#### Zonggui Chen^1,2^, Huan Liu^1, 2^, Xiaobo Gong^1^, Faqi Li^1, 2^

##### ^1^State Key Laboratory of Ultrasound Engineering in Medicine Co-Founded by Chongqing and the Ministry of Science and Technology, Chongqing, China; ^2^College ofBiomedical Engineering Chongqing Medical University, Chongqing, China

###### **Correspondence:** Zonggui Chen

**OBJECTIVES** Study on the activity of cavitation cluster under the different ambient pressure by the average intensity of sonoluminescence.

**METHODS** The experiments, activities of cavitation bubbles and the sonoluminescence are captured by general camera and emICCD under different ambient pressure, are conducted in the same experiment conditions. The system with the software light field can measure the average intensity of sonoluminescence.

**RESULTS** The intensity of multibubble sonoluminescence was increased, when ambient pressure increased from 0.1MPa to 10MPa. However, the region of cavitation cluster decreased gradually.

**CONCLUSIONS** The increasing ambient pressure caused active area of cavitation cluster to decrease. Besides the cavitation cluster activity will be more drastic, when electronic power reaches a certain value.

### P72 Method of temperature estimation in skin for a focused ultrasound applicator using a multi-validated numerical thermal model

#### Aghuinyue E. Umenei, Ziqi Wu

##### Global Discovery, Amway, Grand Rapids, Michigan, United States

###### **Correspondence:** Aghuinyue E. Umenei

**OBJECTIVES** Focused ultrasound through ablation has gained increasing use in aesthetic applications over the last decade. Recent studies show non-ablative focused ultrasound also provided clinical benefits through localized heating. Temperature measurements are critical for efficacy and safety for such applications but are not easily or cheaply obtainable under *in vivo* dermatological conditions. Current temperature measurement methods are expensive, time consuming, application specific/organ dependent and provide unsatisfactory spatiotemporal resolution. We propose a method including a multiphysics model to quickly estimate skin temperature distribution in 2D, which was validated using multiple approaches and lends itself to an iterative development process.

**METHODS** An acousto-thermal 2D FEA multiphysics model was used to estimate focused ultrasound thermal distribution in a tissue mimic materials (TMMs) which are acoustically similar to skin tissue, by incorporating acoustic field 2D surface measurements of the 3W applicator from its hydrophone scans (Fig. 1). The effects of ultrasound on tissue heating were modeled in two phases with parameters both measured in the lab and obtained from literature where possible. Initially, the acoustic pressure in theTMM generated by the applicator was initially solved using wave equations in the frequency domain. The thermal distribution in the tissue was subsequently calculated using the obtained acoustic energy as the heat source term to the bioheat equation in a time domain simulation over 7 seconds (Fig. 2). The simulated transversal 2D temperature distribution was compared to thermocouple measurements in the tofu for multiple applicators (10). The thermocouple measurements were taken at 0.5mm spacing along the longitudinal axis of the applicator, at 2, 4, 6, and 8 mm below the surface to give a 2D measurement grid of the TMM. The focal distance of the ultrasound applicators were measured to be 4±1 mm in water. Furthermore, simulations were performed using a porcine muscle model and the results were compared in a similar fashion to MR thermometry acquired in muscle samples. The validated multiphysics model was finally used to estimate a thermal distribution for multilayered skin model.

**RESULTS** The model estimated average maximum temperature rise from nominal (37 C) in tofu at the focus, at 6.9 C compared to 6.56 C from the thermocouple measurement along the focal line of interest. A good correlation (R2 = 0.89) was seen between modeling results and thermocouple measurements in tofu for 10 ultrasound transducers (Fig. 3). For porcine muscle, model estimated temperature rise along focal line of interest of 14.9 C whereas MR thermometry measured 14 C. Model accuracy was between 88%-93% once equipment error was factored, providing an acceptable temperature estimate for developmental research at the high resolutions need for skin tissue. Skin multilayer model estimated the maximum temperature was 19 C at the focal region.

**CONCLUSIONS** Model estimated temperature in tissue mimic material and porcine muscle were validated with thermocouple and MR thermometry approaches. Byinputting free-field acoustic field information and acousto-thermal properties of tissue media and building a validated 2D model, we have the proposed an easy method of estimating temperature changes in multilayered tissue such as skin with close approximation at a low cost and good accuracy.

**Fig. 3 (abstract P72).**
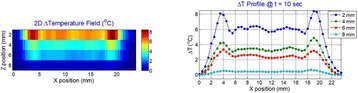
Surface map and line measurement of temperature rise of ultrasound applicator in tofu after 7s (longitudinal section)

**Fig. 1 (abstract P73).**
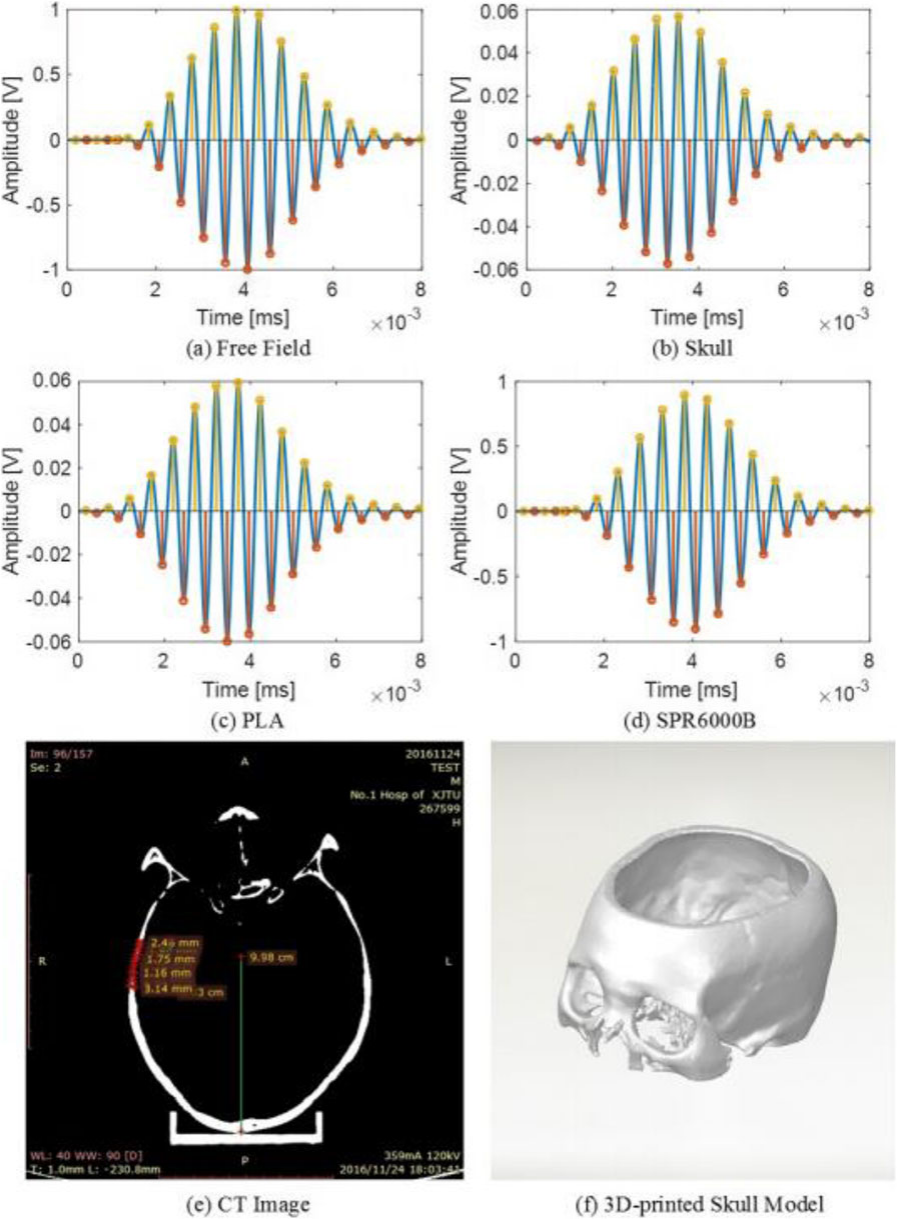
Receiving signals in the (a) free field, (b) skull, (c) PLA and (d) SPR6000B; (e) the CT scan image and (f) the 3D­printed model

**Fig. 1 (abstract P74).**
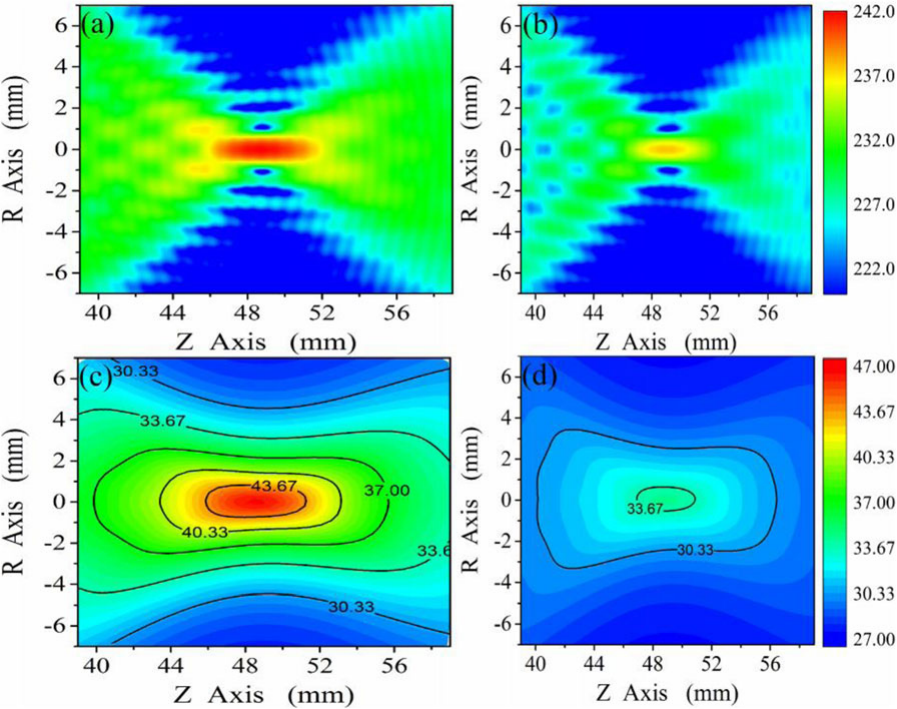
Simulated acoustic pressure lever and temperature distributions on the focal plane: acoustic pressure lever with (a) the corrugated transducer and (b)conventional transducer; and temperature distribution in phantom exposed with (c) the corrugated transducer and (d) conventional transducer

### P73 A 3D-printed skull model with corresponding acoustic characteristic of human skull for ultrasound brain imaging and diagnosis

#### Chen Bai, Meiling Ji, Dui Qin, Mingxi Wan

##### The Key Laboratory of Biomedical Information Engineering of Ministry of Education, Department of Biomedical Engineering, School of Life Science and Technology, Xi`an Jiaotong University, Xi'an, China

###### **Correspondence:** Chen Bai

**OBJECTIVES** Recently, more and more attention has been paid to the ultrasound brain imaging as it is non-radioactive compared with CT and low-cost compared with MRI. And the existence of skull limits the propagation of the ultrasound and then limits the frequency of transducer to be transmitted and furtherly limits the resolution ofthe resulting image. To realize the noninvasive transcranial brain imaging, the foreknowledge of the acoustic characteristic of the skull is essential. The ossa temporale (temporal bone), which is the thinnest part, has been proved that is the best acoustic window for imaging. However, the intact human skull was precious and hard to get the part we just need by excision. Instead, with the help of the 3D printing technology and related software, this problem can be overcome easily. Furthermore, various invitro research experiments about brain imaging can be proceeded with the 3D-printed model which matches the acoustic characteristic of human skull.

**METHODS** The acoustic attenuation coefficient and ultrasonic sound velocity for the temporal bone was firstly measured. The human skull was mounted on a threedimensional scaffold and immersed in a tank with degassed water. Two single element transducers were placed on the walls of the tank coaxially on each side of the skull manually parallel to the temporal bone, one for transmitting signal and the other for receiving. The transducer was excited by a 5-cycle Sine signal whose peak to peakvalue was 2 V and the receiving signal was collected with the NI data acquisition system. The direct ultrasonic wave in free field was normalized and recorded as a reference signal for calculating the attenuation coefficients and velocity. Each time, we measured at 10 different points of the temporal bone, and each point was repeated ten times and get the mean value (Fig. 1). For ensuring the optimum frequency for transcranial experiment, 5 pairs of transducers with frequency ranging from 1 to 5 MHz were tested. Moreover, the CT scan images of human skull was obtained, with 1mm interval between 2 layers, to measure the thickness of bone, and furtherly, to design and modify the model by MIMICS for 3D print. After that, to find a certain material matching the acoustic characteristics of the human skull and the cerebral vessels, the attenuation coefficients and velocities of 9 different kinds of materials including resin, nylon, PEEK, PLA, etc. with different thickness were measured. Additionally, considering the effect of the temporal bone’s curvature, the materials were modeled in the shape of the temporal bone rather than flat plate. By aid of the software MIMICS, we got the satisfying models with excision of the redundant part of the skull.

**RESULTS** According to the results of measurement, 1.8 to 2 MHz was determined with synthetic consideration of the attenuation, propagation and the resolution for further imaging. Hence, the averaged attenuation coefficient and velocity of the temporal bone for the human skull model measured with 1.9 MHz frequency were 7.34 dB/mm and 2398.13 m/s, respectively. And for the optimum material within the 9 kinds of materials, PLA, those were 7.11 dB/mm and 2270.24 m/s under the same testing condition, which means the thickness of the 3D-printed skull model with the PLA should be the same as that of human skull. In other words, the mean thickness of the temporal bone of the printed skull model using PLA was about 1.8 mm, in correspondence with the acoustic characteristic of human skull with the mean thickness of1.7 mm. For another, the material to print the vascular model, one kind of resin, the SPR6000B, with averaged attenuation coefficient 0.8 dB/mm and velocity 1963 m/s, approximating to the results in the free field, was picked out. Finally, by measuring the performance of the new models, it has been proved that the human skull and cerebral vessels can be conveniently and completely replaced by the 3D-printed models.

**CONCLUSIONS** In order to design a skull model which can completely replace the intact human skull in ultrasound brain imaging study, we measured the acoustic characteristics of temporal bone of human skull, and moreover, a number of different materials to ascertain the material whose acoustic attenuation characteristic was similar to the human skull or the vascular. The results showed that PLA met the requirement for skull model and the attenuation coefficient of SPR6000B was small enough to print the vascular model. Finally, we have acquired the satisfying skull model with excision of the cranial skullcap, and to fix the designed vascular model conveniently. In the following study of brain imaging, a scheme that has the contrast agents circulate in the vascular model fixed on the skull model, and manage to imagethe vascular through the temporal bone *via* a 128-element linear array transducer with 2 MHz frequency will be performed.

**Fig. 1 (abstract P77).**
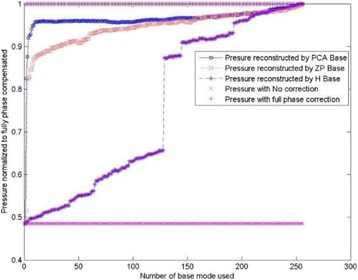
The focus pressure versus number of base mode used curves for PCA base, ZP base and H base. Note the rapid convergence of the PCA acceleratedmeasurement process, which only takes 4 base modes while ZP and H based process requires much more modes

### P74 Enhanced ultrasonic focusing and temperature rise by using sub-wavelength periodic structure

#### Chenghai Li, Xiasheng Guo, Juan Tu, Dong Zhang, Zhou Lin

##### Key Laboratory of Modern Acoustics (Nanjing University), Ministry of Education, Nanjing University, Nanjing, Jiangsu, China

###### **Correspondence:** Chenchen Bing

**OBJECTIVES** High-intensity focused ultrasound (HIFU) has been used in clinic as a non-invasive therapeutic method to treat solid tumors for decades. Although various (e.g., lens, spherically curved transducer and multi-element phased array) were developed to focus ultrasonic energy, the ultrasonic focusing efficiency is highly restricted by the size of transducers due to the limitation of manufacture technology. In the present work, a concave transducer with periodic array surface was designed to realize focusing enhancement.

**METHODS** To verify the enhanced acoustic focusing of concave transducer with periodically aligned subwavelength grooves, the acoustic pressure and temperature elevation generated by the new structure were investigated based on both experimental measurements and numerical simulations, and then compared with the results obtained from conventional concave transducer. The software of Comsol Multiphysics was employed to perform the numerical simulations based on the combination of2D Helmholtz equation and bio-heat transfer equation. An optical fiber hydrophone and thermocouple were utilized to measure the acoustic field and detect the temperature rise at the focus in a tissue phantom exposed to HIFU pulses, respectively.

**RESULTS** Both the experimental measurements and numerical simulations show that the peak pressure amplitude and the rate of temperature rise at the focal region are obviously larger in the case of corrugated transducer rather than the conventional transducer as Fig. 1. Different from previous studies originated from planar arrays of periodic structure, the application of spherically curved arrays of periodic grooves could result in extraordinary acoustic transmission close to Wood’s anomaly that at the wavelength slightly less than the groove periodicity.

**CONCLUSIONS** Numerical simulations and experimental measurements both show that, comparing with conventional concave transducer, the concave transducer with periodic array of subwavelength grooves is more efficient to improve acoustic focusing and enhance relative bioeffects. This work may open new possibility to design more favorable focused ultrasonic transducers that could significantly improve HIFU treatment effects.

**Fig. 2 (abstract P77).**
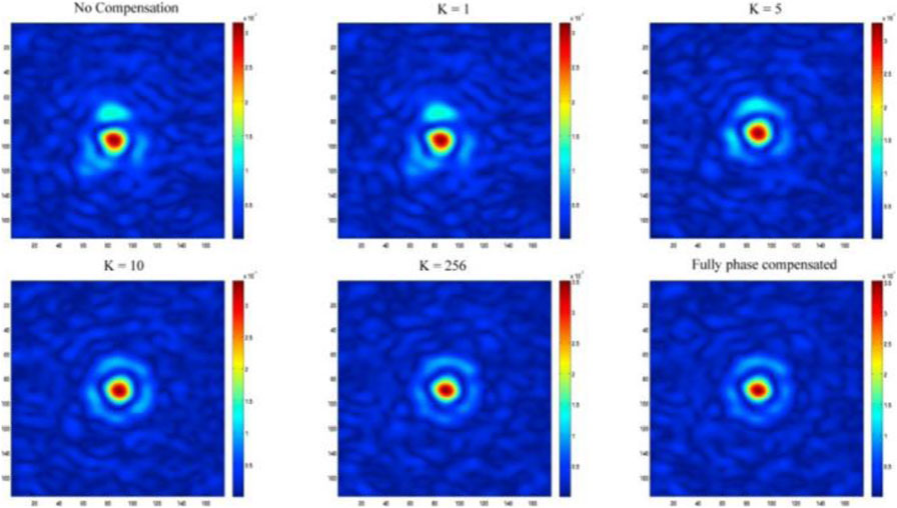
Simulated pressure maps obtained after 1x4, 5x4, 10x4, and 256x4 measurements of the autofocusing process. Note the pressure map acquired after 10x4 measurements is largely the same as fully phase compensated one, indicating a rapid refocusing process

### P75 Optimization of thermal fields by focal region modulation in HIFU brain tumour treatment

#### Shihui Chang^1^, RUI CAO^2^, Yabin Zhang^1^, Shijing Wu1, Xiqi Jian^1^

##### ^1^Tianjin Medical Univercity, Tianjin, China; ^2^Tianjin University of Science & Technology, Tianjin, China

###### **Correspondence:** Shihui Chang

**OBJECTIVES** The temperature rise during HIFU brain tumor treatment should be controlled accurately to avoid side effects by overheating such as cerebral hemorrhage. In this work a modulation method to control the shape and volume of focal region as well as the temperature distribution at focus was performed by adjusting the driving signals of the transducer elements.

**METHODS** The simulation model was reconstructed based on the CT data of a volunteer's head and the 82-element transducer. In this study, the finite difference time domain (FDTD) method was used to calculate the temperature distribution induced by HIFU in the brain. Two foci with a certain distance were generated on the acoustic axis by a HIFU source using two driving signals which were determined by time reversal method. This double foci fusion method was applied to create a uniform temperature distribution at focal region and adjust the length and volume of the focal region.

**RESULTS** Thermal field distribution at focal region can be adjusted by changing the time delay of the two driving signals. Thus, a uniform distributed temperature field can be created at focal region. What is more, this method was able to adjust the length and volume of the focal region using the same total acoustic energy by varying the distance between the two foci.

**CONCLUSIONS** The HIFU temperature distribution, shape and volume of the focal region could be modulated by the double foci fusion method. The focal region volume formed by a single irradiation could be enlarged effectively while avoiding the brain tissue overheated. This method can provide basis for the focal temperature distribution modulation in the treatment of brain tumor in further study.

### P76 Modelling and simulation for the focused ultrasound ablation of liver tumour

#### Maxim Solovchuk^1,2^, Tony W.H Sheu^2^, Manuel Diaz^1^, Peter Deng^1^

##### ^1^Institutes of Biomedical Engineering and Nanomedicine, National Health Research Institutes; ^2^Engineering Science and Ocean Engineering Department, National Taiwan University

###### **Correspondence:** Maxim Solovchuk

**OBJECTIVES** High intensity focused ultrasound is very promising new technology, that has many therapeutic application, among them are the treatment of cancer indifferent organs. One of the main difficultlies, that limits further development of HIFU, is very difficult treatment planning and unpredictable behavior of the necrosed area in the presence of bubbles. Computational fluid dynamics can greatly help in this regard. Calculation of ultrasound propagation in a patient specific geometry is very time consuming process. In order to achieve reasonable simulation time HPC (high performance computing) should be used. The problem will be solved using HPC on multiple GPUs. This work is a step towards the development of surgical planning platform for a non-invasive HIFU tumour ablative therapy in a real liver geometry.

**METHODS** The computational model has been developed for the prediction of temperature elevation in the patient specific liver geometry. The developed computational model is based on the nonlinear Westervelt equation with relaxation effects being taken into account, bioheat equations for the perfused tissue and blood flow domains. The nonlinear Navier-Stokes equations are employed to describe the flow in the large blood vessels. A new equation is derived for the description of large amplitude oscillations of bubbles in viscoelastic medium. The cavitation model is coupled with acoustic and thermal models.

**RESULTS** Three dimensional field coupling computational study has been performed. Explicit symplectic finite difference scheme has been developed for the solution of full wave equation. We used multiple GPUs for the modeling of nonlinear wave equation. Temperature elevation by focused ultrasound in a minipig has been studied experimentally by MRI and numerically. The method for the non-invasive thermal tissue properties has been proposed using MRI. Good agreement between the predicted and measured results has been obtained. It was also shown that in large blood vessel both convective cooling and acoustic streaming can change the temperature considerably near blood vessel.

**CONCLUSIONS** Three dimensional simulations of focused ultrasound ablation has been perfomed in a patient specific geometry. Simulation using multiple GPUs can sufficiently reduce the simulation time and can help to construct surgical planning platform. High ultrasound power and nonlinear propagation effects with appropriate treatment planning can sufficiently reduce the treatment time. We theoretically showed that the treatment time can be reduced to few minutes. The presented model can be used in planning tools for the thermal ablation of tumour in other organs.

### P77 Principal component analysis of acoustic aberrations for rapid HIFU beam refocusing: a simulation study

#### Xiaoxu Lei, Martijn de Greef, Chrit Moonen, Mario Ries

##### Imaging, University Medical Center Utrecht, Utrecht, Netherlands

###### **Correspondence:** Xiaoxu Lei

**OBJECTIVES** The propagation path of the HIFU beam frequently introduces acoustic aberrations, which degrade the focus quality. Non-invasive refocusing 1-3 based on acoustic radiation force imaging (ARFI) have been proposed, which address this problem. However, the refocusing process is generally lengthy and cumbersome, due to the multitude of independent measurements. Here, we evaluate the feasibility of accelerating this process by replacing the Hadamard (H) base and Zernike polynomials (ZP) base of the original approaches by a new base which is derived using principal component analysis (PCA) on simulated phase aberrations from a uterine fibroid model.

**METHODS** The pressure at HIFU focus can be expressed as the sum of the product of the acoustic wave fired from each individual transducer element and the acoustic aberration experienced along its beam path:p = ∑^N^_i_=1 gi.χi, (1)where χi is the complex source term of each emitter, gi the aggregated attenuation and phase shift along the ith propagation path for the element and p the complex focus pressure of all elements.For non-iterative adaptive refocusing approach, Eq.1 can be transformed into matrix form:pX = g.X, (2)Where pX is a 1 x K complex row vector whose elements are the focus pressures corresponding to each excitation pattern from base X, g is a 1 x N complex row vector containing the aberration information for each transducer element, X is the N x K orthogonal base whose column vectors are used for the actual measurement process, which requires 4xK independent measurements. Both, a Hadamard base H and Zernike polynomials base Z have been applied as X in Eq.2 for refocusing the beam. In order to accelerate the refocusing process, the less numbers of columns of X used to get accurate g in Eq.2 the better. Here, a method is proposed to apply PCA on prior knowledge of the phase aberrations obtained from acoustic simulations to form an orthogonal base (PCA base), which allows to describe the dominating phase aberrations in a more compact form and thus allows to significantly reduce the measurement process. Simulation studies were conducted based on 4 MRI datasets of uterine fibroid patients, which were obtained during MR-guided HIFU therapy. For each patient, 343 different focus positions within the ablation volume were chosen and the corresponding phase aberrations for each focus position were obtained using a stochastic ray tracing method4. Subsequently, 342 positions served to obtain the new compact base, which was finally validated for rapid autofocussing.

**RESULTS** As Fig. 1 shows, PCA based autofocussing typically re-established 90% of the fully phase corrected focus pressure after only 16 measurements, whileZernike and in particular Hadamard based correction require a significantly (5-20 times) longer measurement time, to achieve the same improvement. Figure 2 compares the pressures maps when applying different numbers of PCA base modes and indicates that using about 10 modes are able to reduce the side lobes and have similar results as fully phase compensated.

**CONCLUSIONS** Although ARFI based HIFU autofocusing for the compensation of beam aberrations has shown a substantial promise, so far the approach is frequently limited by unacceptably long measurement time of several minutes per focus point. Here, we demonstrate that autofocusing using an individually adapted base derived from prior knowledge can substantially shorten this process.

**References**

[1] Herbert et al., IEEE Trans. Ultrason. Ferroelectr. Freq. Control 56(11) 2388 – 99, 2009

[2] Larrat et al., IEEE Trans. Ultrason. Ferroelectr. Freq. Control 57(8) 1734 – 7, 2010

[3] Kaye et al., Medical Physics 39 6254 (2012)

[4] Koskela et al., J.Acoust.Soc.Am. 136(3), 2014

**Fig. 1 (abstract P78).**
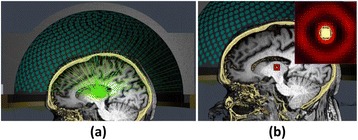
(a) The visualization of wave beam path using ray tracing method. (b) The Sagittal plane of simulated focal pressure

**Fig. 1 (abstract P80).**
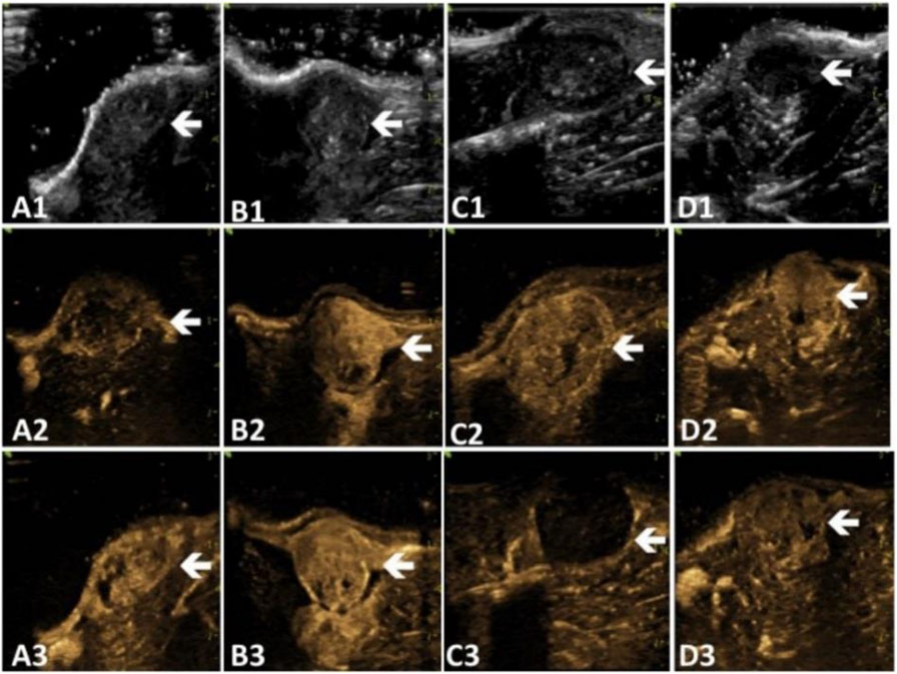
Ultrasound contrast image of VINNO70 before and after the treatment.(A, B, C, D on behalf of MI=0.3,0.7,1.4 group and control group, 1, 2 and 3 respectively represent the two­dimensional ultrasound,ultrasound contrast image before treatment and after treatment) Images show that the group of MI=0.3 combined with microbubbles can enrich the blood perfusion of VX2 tumor in rabbits, MI=1.4 group decrease the blood perfusion,and there was no significant change in the blood perfusion of the MI=0.7group and control group before and after treatment

### P78 A fast 3-D transcranial focused ultrasound simulation based on ray tracing

#### Changzhu Jin^1, 2^, John Snell^2^, Dong-Guk Paeng^1,2^

##### ^1^Ocean System Engineering, Jeju National University, Jeju, Korea; ^2^Focused Ultrasound Foundation, Charlottesville, Virginia, USA

**OBJECTIVES** Two objectives are explored: improved skull aberration correction and focal spot quality visualization. Others have used full wave acoustic simulation to explore the possibilities of more accurate phase correction and the simulation and visualization of aspects of focal spot quality [1]. However, the computational cost of such complete acoustic simulations prevents, at the current time, their routine clinical use. In current clinical practice, the location and quality of the hotspot must be evaluated and corrected for during the procedure itself through the use of a number of low-power sonications prior to a final therapeutic sonication [1]. It would bebeneficial to have pre-surgical planning information to inform patient selection and procedure planning in advance of the delivery of energy to a patient. Patient-specific skull characteristics and particular anatomical targets within the skull can make achieving prescribed focal spot temperature elevations difficult or impossible. The focal spot quality in terms of location and temperature could be improved by changing transducer parameters, relative angle between the transducer and patient skull, and morerealistic phase correction. A phase correction taking account of realistic parameters with sufficiently fast computation is desired to accomplish these optimizations closer to or within the clinical workflow. In this work, a simplified acoustic simulation tool based on GPU-accelerated ray tracing was developed to calculate skull aberration correction and to provide real-time visualization of an estimated three-dimensional (3D) pressure field around the targeted focal spot.

**METHODS** A modular 3D planning software system was developed for transcranial focused ultrasound procedure planning. Within this environment, CT and MRimages can be displayed and registered. Skull surfaces in the CT image are characterized using a threshold-based algorithm and a 3D edge detection operator. A single layer skull model was assumed. A virtual FUS transducer which has a 30cm diameter hemispherical shape and consists of 1024 Fibonacci patterned elements was designed. Each transducer element acoustic beam is represented by a computed path through the cooling water, skull and brain tissue. This path from the transducer to the focal point was divided into 3 individual ray segments (water, bone, and brain). The refraction caused by impedance difference at skull bone surfaces was computed based on Snell’s Law. Any ray with an incident angle at the outer skull surface greater than critical angle was neglected. The computation volume of pressure field contains 21×21×21 cubic sample points and the distance between each point on XYZ axis is 1mm. The center of the computation volume was set to lie on the target point. The projection vector of each sample point onto the third ray segment was computed and the length projection was taken as new third ray segment. In order to get a focused spot, the phase at the end of the ray trace was collected and utilized for phase correction prior to pressure computation. The pressure field contribution from each ray trace to the sample point was accumulated using a simplified beam profile model. The attenuation coefficient of water, skull, and brain was set as 0.0022, 0.3103 and 0.0690 dB/cm/MHz respectively. The density of the water, skull, and brain was set as 1000, 1900 and 1030kg/m^3. The assumption of exponential decay on the pressure along longitudinal direction was applied and a sinc function was implemented as pressure decay on the axial direction of the beam.

**RESULTS** This tool provides an intuitive 3-D view of pre-treatment imaging data and also improves the understanding of the focal quality on various FUS transducer setups interactively. The pressure field which takes account of attenuation and transmission loss was calculated and displayed within 1 Hz computation speed (Fig. 1b).

**CONCLUSIONS** A fast 3D patient data visualization and acoustic simulation software based on ray tracing method were developed (Fig. 1a). The phase correction andtransducer setup could be exported to a third party full-wave simulation software for more accurate prediction of the pressure field. It offers help to the clinician with patient selection and to decide the optimum transducer setup during pretreatment planning. Validation of the accuracy of the pressure field computed by this method as compared with full wave simulation methods and hydrophone data is the subject of future work.

**Reference**

[1] Clement, G.T. and K. Hynynen, Phys Med Biol, 2002. 47(8): p. 1219-36.

**Fig. 1 (abstract P81).**
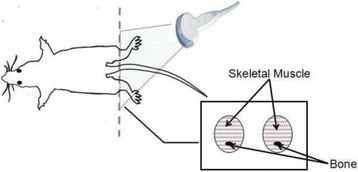
Illustration of simultaneous contrast imaging of both limbs through a transverse section

### P79 Efficacy of ultrasound mediated microbubbles in diclofenac gel to enhance transdermal permeation in rheumatoid arthritis induced rat

#### Ai-Ho Liao^1^, Ho-Chiao Chuang^2^

##### ^1^National Taiwan University of Science and Technology, Taipei, Taiwan; ^2^National Taipei University of Technology, Taipei, Taiwan

###### **Correspondence:** Ai-Ho Liao

**This abstract is not included as it has already been published:**

Liao A.H, Chuang H.C, Chung H.Y. Efficacy of ultrasound mediated microbubbles in diclofenac gel to enhance transdermal permeation in rheumatoid arthritis induced rat. IEEE. 2015. Available from: http://ieeexplore.ieee.org/document/7319152/.

### P80 Vascular effect of rabbit VX2 tumour induced by diagnostic ultrasound with microbubbles

#### Xueyan Qiao, Zhong Chen, Cuo Yi, Wenhong Gao, Shunji Gao, Zheng Liu

##### Ultrasound Department, Xinqiao Hospital, Third Military Medical University, Chongqing, China

###### **Correspondence:** Xueyan Qiao

**OBJECTIVES** To investigate the different vascular effect of rabbit VX2 tumor induced by diagnostic ultrasound combined with microbubbles (MB).

**METHODS** Forty rabbit with VX2 tumor were treated by diagnostic ultrasound with different strengths of Mechanical index (MI) 0.3,0.7,1.4 with MB as experiment group (n=10, n=10, n=10) and negative control (n=10) for 5 minutes. 0.1ml microbubbles in 5 ml normal saline was used as a therapeutic dose. The tumor blood perfusion was imaged with contrast-enhanced US (CEUS) before and after treatment which were analyzed by the contrast analysis software of VINNO 70 . After the treatment, the tumor samples were collected for pathological examination.

**RESULTS** The PI in MI=0.3 increased after treatment, the PI and AUC of MI=1.4 decreased after treatment (Fig. 1).

**CONCLUSIONS** Low-intensity diagnostic ultrasound (MI=0.3) combined with microbubbles can enrich the blood perfusion of VX2 tumor in rabbits, and high-intensity diagnostic ultrasound (MI=1.4) can decrease the blood perfusion.

**Fig. 2 (abstract P81).**
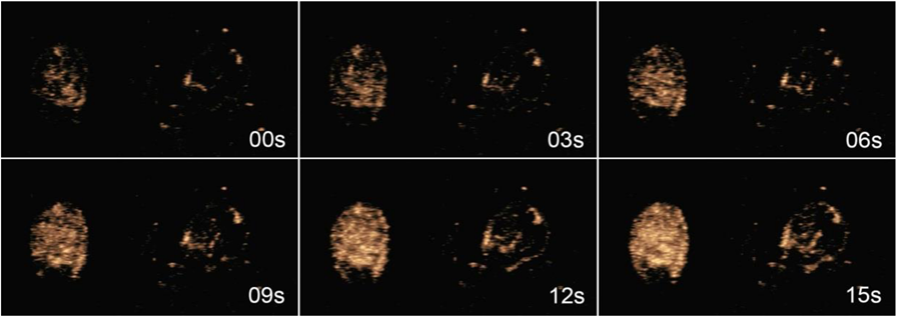
Continuous screen shots of contrast imaging after treatment procedure. The treated side (left) showed much more rapid and abundant perfusion of peripheral circulation than the control side (right)

### P81 Augmentation of muscle perfusion by microbubble mediated ultrasonic cavitation

#### W. Gao, C. Yi, X. Qiao, Z. Liu

##### Department of Ultrasound, Xinqiao Hospital, Third Military Mediical University, Chongqing, China

###### **Correspondence:** W. Gao

**OBJECTIVES** Ultrasound has been known capable of enhancing tissue perfusion through both thermal and non- thermal bio-effects. The main purpose of this study is to investigate if the enhanced non-thermal ultrasonic bioeffect- microbubble mediated cavitation could effectively agitate blood perfusion of mice skeletal muscle.

**METHODS** Twelve healthy nude rice were intravenously injected microbubbles for both ultrasonic cavitation and contrast imaging. Firstly, one randomly chose posterior limb underwent microbubble mediated cavitation, while the other side went through no treatment as an own control. Intermittent ultrasound applied for cavitation was set at a frequency of 4 MHz, pulse length of 18 cycle, pulse repetition frequency of 50 Hz, mechanical index of 1.4, work/rest interval of 0.48s/2s, and total irradiating time of 10 minutes. Microbubbles for cavitation with a number of approximately 8x10^7^ was injected slowly meanwhile irritate ultrasound was applied. Then simultaneous contrast imaging of both limbs through a transverse section was conducted right after cavitation procedure (Fig. 1). Dynamic contrast video was recorded for evaluating of skeletal muscle perfusion, and quantitative parameters including peak intensity, area under curve, and ascending slope were analyzed. Average values of treatment side and control side were statistically compared using paired sample T test.

**RESULTS** Contrast imaging records exhibited prominent acceleration and abundance of treated muscle perfusion. Entry of microbubbles into peripheral circulation is much more rapid and numerous in the treated side than in the control side (Fig. 2). Quantitative analysis results also showed a steady rise of perfusion on the treated side with statistical significance. Average values of peak intensity, area under curve and ascending slope in control side were (71.2467±10.86445), (6436.7658±981.94242), and (0.7342±0.30417), while in treated side they were (82.0100±11.23804), (7584.8033±916.03143), and (0.9208±0.39867),respectively (P<0.05) (Fig. 3).

**CONCLUSIONS** Microbubble cavitation irritated by high mechanical, low duty cycle intermittent ultrasound could effectively agitate mice skeletal muscle perfusion, leading significant raise of both blood velocity and blood volume.

**Fig. 3 (abstract P81).**
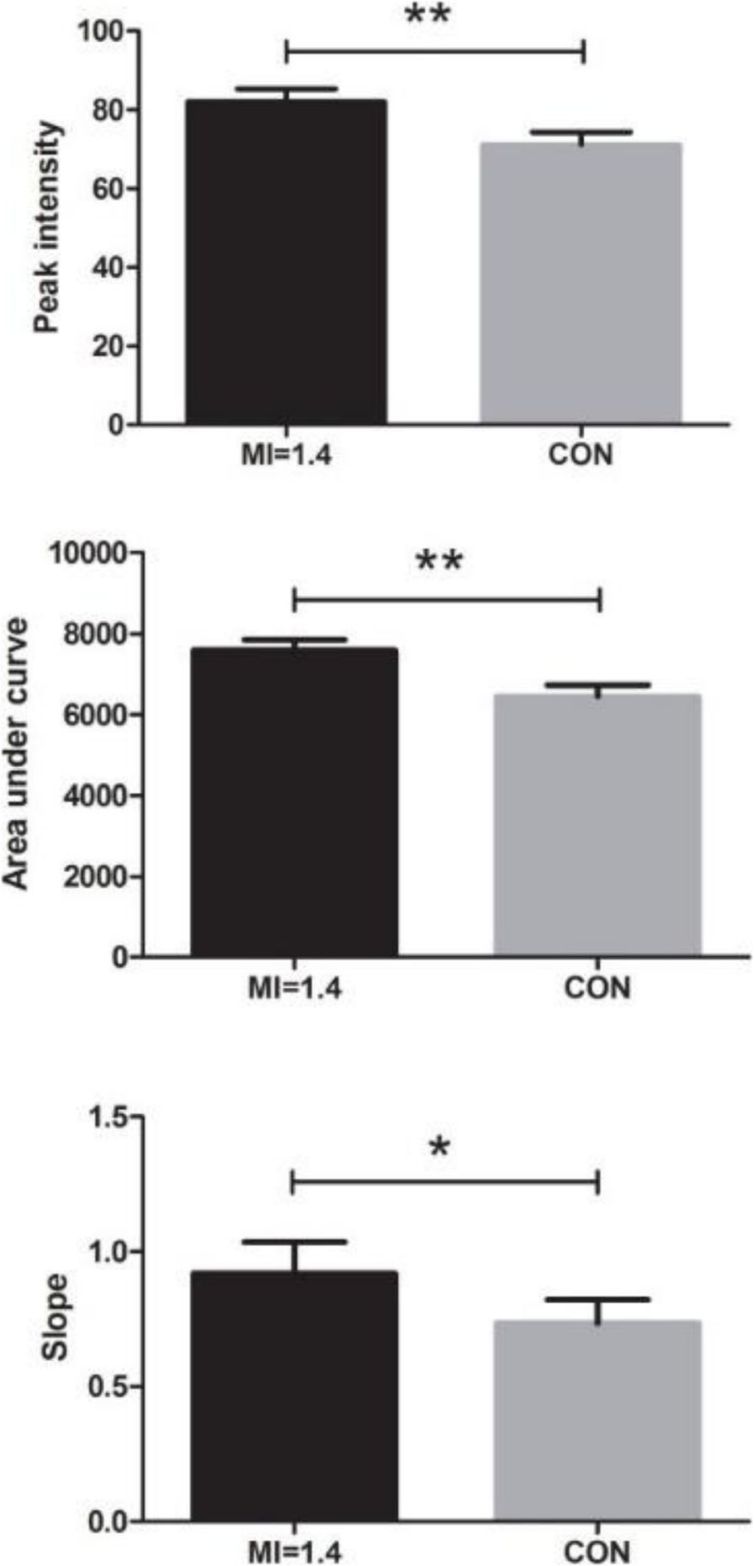
Histogram of peak intensity value, area under curve and ascending slope in the two groups

**Fig. 1 (abstract P82).**
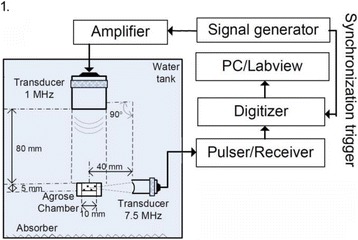
Experimental Setup for ultrasound exposure and cavitation detection

**Fig. 2 (abstract P82).**
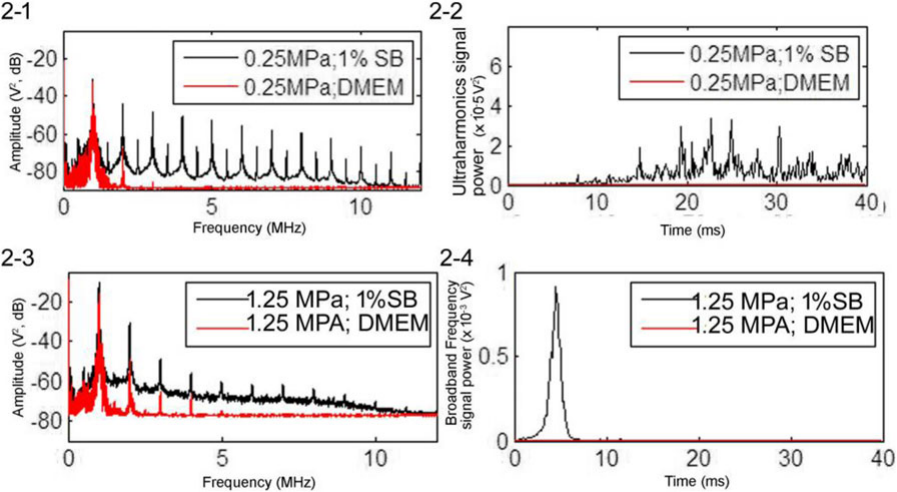
Panc-1 cells mixed with 1% SonoVue bubbles (black water) and water (as control, redcurve) exposed to different PNP (0.25 MPa and 1.25 MPa). 1 KHz PRP and 30% duty cycle. Frequency domain of the recorded signal in a period with 0.25 MPa (2-1) and 1.25 MPa PNP (2-3). Power of untraharmonics (2-3) and broadband components (2-4) as a function of exposure time

### P82 Relationship between different molecule sizes and delivery efficiency of pancreatic cancer cells mediated by different cavitation dose

#### Lizhou Lin^2,1^, Mouwen Cheng^1^, Fan Li^2^, Lianfang Du^2^, Alfred C. Yu^3^, Peng Qin^1^

##### ^1^Department of Instrument Science and Engineering, Shanghai Jiao Tong University, Shanghai, China; ^2^Department of Ultrasound, Shanghai First People’sHospital, Shanghai Jiao Tong University, Shanghai, China, 3Department of Electrical and Computer Engineering, The University of waterloo, Waterloo, Alberta, Canada

###### **Correspondence:** Lizhou Lin

**OBJECTIVES** Present studies have validated acoustic cavitation, triggered by ultrasound and microbubbles, could enhance the membrane permeability of pancreatictumor cells for improving macromolecular drug delivery. However, the efficiency of different molecular sizes internalized into the pancreatic tumor after acoustic cavitation is not clear. This work aims to address the relationship between different molecule sizes and delivery efficiency of pancreatic cancer cells under different types and doses of acoustic cavitation.

**METHODS** The pancreatic cancer Panc-1 cells mixed with 1% SonoVue microbubbles and FITC-dextran with different molecule sizes (4 kDa, 40 kDa and 500 kDa) were placed in a tissue mimicking chamber and exposed to 1-MHz ultrasound with 1 kHz pulse repetition frequency, 300 cycles and different peak negative pressure (0.25 MPa, 0.6 MPa, 1.25 MPa) (Fig. 1). Another 7.5-MHz focused transducer was employed to passively receive acoustic signals (Fig. 2). The intensities of the ultraharmonics and broadband components were respectively measured to quantify the dose of stable and inertial cavitation, respectively. The samples were stained by Propidium Iodide 30 min after sonication, and then were analyzed by flow cytometry to assess delivery efficiency and cell viability. Five replicates experiments were conducted to calculate the mean standard deviation and statistically analyze.

**RESULTS** 1. The delivery efficiency (5.90±1.37%) for different FITC-dextran and the necrotic ratio (4.44±1.05%) were not enhanced after Panc-1 cells underwent stable cavitation, compared with the control without exposure to ultrasound (3.41±1.37% and3.08±0.51% for delivery and necrosis ratio, respectively).2. No significant difference (P > 0.05) among the delivery efficiency of FITC-dextran with different mass was observed when Panc-1 cells were exposed to different inertial cavitation dose (ICD) (Fig. 3). Relatively high ICD induced 30.63±7.73%, 27.57±6.59% and 26.11±5.62% delivery ratio for 4 kDa, 40 kDa and 500 kDa FITCdextran, respectively.3. Both the delivery efficiency for all FITC-dextran and the necrotic cells were positively correlated with ICD (delivery and necrosis ratio increased by approximate 40%and 60%, respectively when ICD increased about 8 times). The number of the internalized FITC-dextran molecules exhibited negative correlation with molecule size (The fluorescence intensity of the internalized 4 kDa FITC-dextran was almost 110-fold high than that of 500 kDa FITC-dextran, respectively) (Fig. 4).

**CONCLUSIONS** Stable cavitation could not enhance the internalization of macromolecule into pancreatic cancer cells, while the delivery ratio of different molecule sizes was positively correlated with the inertial cavitation dose. Although the delivery rate induced by inertial cavitation was not dependent on the macromolecule size below 35 nm, the number of internalized molecules was negatively correlated with the molecule size. We believe these results would provide useful information for pancreatic tumor therapy mediated by acoustic cavitation.

**Fig. 3 (abstract P82).**
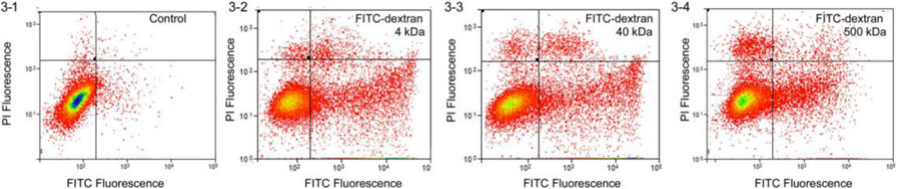
Delivery efficiency of different FITC-dextran (3-1: without ultrasound exposure; 3-2: 4 kDa; 3-3: 40 kDa; 3-4:500 kDa) and necrosis analyzed by flow cytometry Pan-1 cells mixed with 1% SonoVue bubbles and FITC-dextran with different molecule size (4 kDa, 40 kDa and 500 kDa) expsoed to high inertial cavitation dose, corresponding to 1 MHz untrasound 1.25MPa PRP, 1 KHz PRP and 300 cycles

**Fig. 4 (abstract P82).**
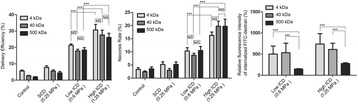
Delivery efficiency (4-1), necrosis ration (4-2) and the relative fluorescence intensity of internalized FITC-dextran analysis when the Panc-1 cells mixed with 1% SonoVue bubbles and FITC-dextran with different molecule size (4 kDa, 40 kDa and 500 kDa) exposed to different intertial cavitation doses (***, *p*<0.001; NS *p*<0.05)

**Fig. 1 (abstract P83).**
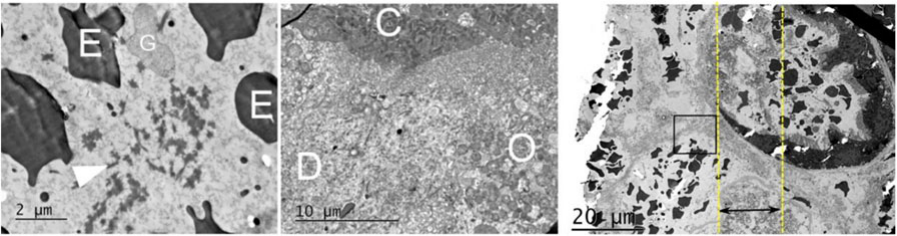
Representative TEM micrographs from the center of the lesion (left), at the border of the lesion (center) and lower magnification view of the lesion border (right). The lesion contained intact erythrocytes (E) in a slurry of cellular debris, fragmented collagen fibrils (arrowhead), and some ghosts of organelles (G). At the border, intact but damaged organelles (O) lie in between cellular debris (D) and intact cells (C). At lower magnification (right), the border region between fully intact and fully fragmented tissue is clearly observed (yellow dotted lines) and measures approximately 20 microns (black arrow)

**Fig. 1 (abstract P85).**
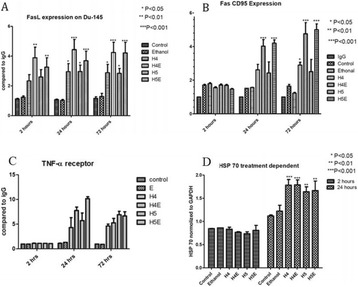
A: FasL expression in prostate cancer cells at 2, 24 and 72 hours post treatment. B: Fas (CD95) expression in prostate cancer cells at 2, 24 and 72 hours. C: TNF­α expression in prostate cancer cells at 2, 24 and 72 hours post treatment. D: HSP70 expression in prostate cancer cell at 2 and 24 hours

### P83 Electron microscopy of renal boiling histotripsy lesions created in an *in vivo* porcine model

#### Y. Wang^1^, S. Buravkov^2^, V. Chernikov^3^, T. D. Khokhlova^1^, G. R. Schade^1^, W. Kreider^1^, A. Maxwell1, M. Bailey^1^, V. Khokhlova^1, 2^

##### ^1^University of Washington, Seattle, Washington, USA; ^2^M.V. Lomonosov Moscow State University, Moscow, Russian Federation; ^3^Research Institute of Human Morphology, Moscow, Russian Federation

###### **Correspondence:** Y. Wang

**OBJECTIVES** Boiling histotripsy (BH) uses millisecond-long pulses of focused ultrasound (FUS) waves with shocks to mechanically homogenize tissue. Histological evaluation has demonstrated that lesions with microscopically fine borders can be generated. Here we report a preliminary evaluation of the ultrastructural characteristics of renal BH lesions created in an *in vivo* porcine model.

**METHODS** Pigs were treated with transcutaneous BH targeting volumes of both kidneys using a 1.5 MHz FUS transducer operating at peak electric power ranging from 0.6 – 4 kW under coaxial B-mode ultrasound guidance. Sonication protocols delivered 10 pulses of 1-10 ms duration and 1% duty factor to 4 adjacent focal points spaced 1 mm apart without respiratory gating. Euthanasia was performed immediately after treatment, and the treated regions of the kidney were carefully removed en bloc and placed in ½ strength Karnovsky’s fixative. The tissue was sliced into 1 mm sections parallel to the direction of treatment. Small regions from the identified lesions and their border (1x2mm) were sampled and processed for transmission electron microscopy (TEM). The remaining tissue slices were processed for histological evaluation. Semi-thin sections were taken from the TEM blocks and stained with 1% methylene blue to determine from which locations to take the ultrathin sections. Ultrathin sections were double stained with uranyl acetate and lead citrate and ultrastructural examinations were performed on JEM-100CX and Zeiss Libra-120 transmission electron microscopes.

**RESULTS** On histologic assessment, lesions appeared to contain a slurry of homogenized cellular debris without apparent cellular structures, areas of petechial hemorrhage, and a distinct lesion border between treated and untreated kidney. At the ultrastructural level, a border region between treated and untreated kidney was observed measuring 10-20 microns, in which cell membranes had been disrupted but intracellular organelles remained intact (Fig. 1). Within the lesion, ultrastructural analysis revealed regions of complete loss of structure with replacement of cells and organelles by electron dense sub-micron cellular debris alternating with small areas of completely disrupted cells containing organelle ghosts. Throughout the lesions, fragmented collagen fibrils were observed (Fig. 1). Within lesions intact erythrocytes were observed within the slurry of cellular debris (Fig. 1) consistent with post-treatment petechial hemorrhage.

**CONCLUSIONS** This data represents the first ultrastructural study of BH lesions generated in an *in vivo* animal model. TEM evaluation revealed that aside from the presence of intact erythrocytes, the lesion contents were similar in characteristic to that observed in *ex vivo* tissue. At the border of the lesion there is a gradation in ultrastructural changes not observed microscopically. Funding: NIH R01 EB7643, K01 EB015745, NSBRI through NASA NCC 9-58, RFBR 16- 02-00653, and Urology Care Foundation.

**Fig. 1 (abstract P87).**
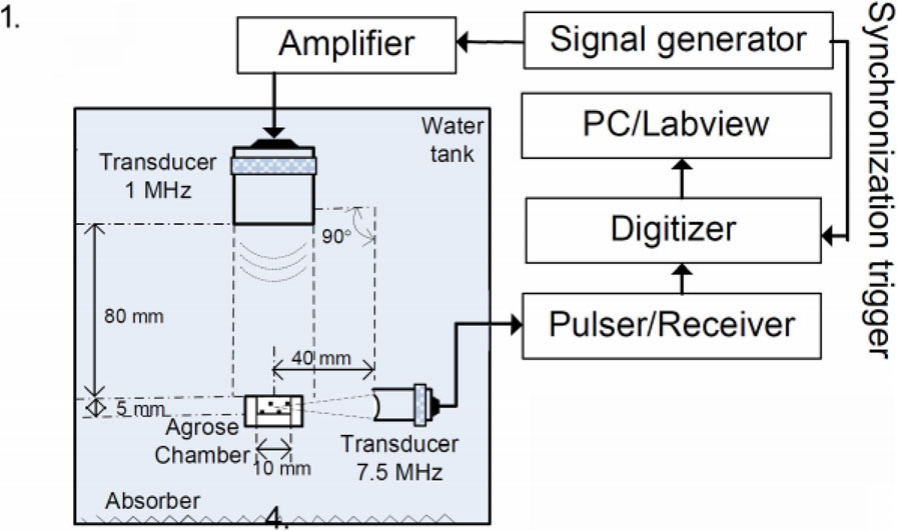
Experimetnal Setupfor ultrasound exposure and cavitation detection

### P84 Subcellular localization of sonosensitizer for autophagy cooperated apoptosis in sonodynamic therapy

#### L. Song

##### BME, The Hong Kong Polytechnic University, Hong Kong, China

**OBJECTIVES** Sonodynamic therapy (SDT), based on the synergistic effect of low-intensity ultrasound and sonosensitizer, is a potential noninvasive approach for the treatment of cancers. However, the specific cellular death mechanisms of sonosensitizers with different subcellular localization patterns remains unknown. In the present study, protoporphyrin IX (PpIX), either endogenously induced by 5-aminolevulinic acid (ALA) or exogenously administered, were used to investigate the effects of its subcellular localization pattern on key cellular activities including mitochondrial dynamics and cargo-selective pathways of autophagy in hela cells.

**METHODS** Different concentration and distribution patterns of ALA-induced PpIX and exogenous PpIX in Hela cells were determined by microplate reader and confocal immunofluorescence microscopy respectively. Cell viability, apoptosis, and autophagy were evaluated by microplate reader and flow cytometry. Mitochondrial dynamics and redox balance were examined by western blot and confocal immunofluorescence microscopy. cargo-selective pathways of autophagy were determined by confocal immunofluorescence microscopy.

**RESULTS** We demonstrated that both autophagy and apoptosis existed in ALA-induced PpIX and exogenous PpIX mediated sonodynamic therapy. However, ALA-SDT mainly caused mitochondria-specific autophagy by harming mitochondrial fusion and fission balance, exogenous PpIX-SDT caused non-selective autophagy by harming cytoplasmic proteins and organelles. Furthermore, ALA-SDT caused unbalance of mitochondrial redox system by excessive production of mitoROS, while exogenous PpIX-SDT caused unbalance of cytoplasmic redox system by excessive production of cytoplasmic ROS. Reciprocally, MnTMPyP, mitoROS scavenger, rescued both cellular autophagy and apoptosis in Hela cells treated with ALA-mediated sonodynamic therapy.

**CONCLUSIONS** Taken together, we conclude that different subcellular localization patterns of PpIX induce different cargo-selective pathways of autophagy, serving as a pro-death function, cooperated with apoptosis to dictate the fate of cell death in sonodynamic therapy.

### P85 Focused ultrasound reprograms ethanol-treated prostate cancer cells back to normal

#### Heng Yu, Hakm Murad, Daishen Luo, Damir Khismatullin

##### Biomedical Engineering, Tulane University, New Orleans, Louisiana, USA

###### **Correspondence:** Heng Yu

**OBJECTIVES** Prostate cancer is the most common and sixth deadliest cancer in men worldwide. Since the majority of prostate cancer patients are elderly men, often not suitable for invasive procedures, there is a need for minimally invasive therapies (e.g., focused ultrasound) against prostate cancer. Previous *ex vivo* and *in vitro* studies conducted in our laboratory showed that high-intensity focused ultrasound (HIFU) synergistically enhanced tumor destruction by ethanol injection. The objective of this study was to investigate the molecular mechanisms behind anti-cancer effects of HIFU. Specifically, we tested the hypothesis that focused ultrasound drastically reduced the metastatic potential of chemically-treated cancer cells *via* overproduction of heat-shock protein 70 (HSP70), death receptor Fas, its ligand FasL and TNF-α receptor.

**METHODS** DU-145 and PC-3 human prostate cancer cells were cultured in T-175 flask in full growth medium. The suspension of cultured cancer cells (100 μl, 2.7million cells/ml) was placed in a 0.2 ml thin-wall PCR tube. The cells were left untreated (control) or exposed to HIFU alone, 4% ethanol, or HIFU + 4% ethanol. The focused ultrasound signal was generated by a 1.1 MHz transducer in the continuous or pulsed mode, with acoustic power ranged from 4.1 W to 20.52 W. HIFU level 4(H4) in this experiment has the acoustic power of 8.72 W. The Fas, FasL and TNF-α expression in the cancer cells was measured by flow cytometry at 2, 24, and 72 hours post-treatment. The Hsp70 protein levels were determined by Western blot analysis at 2, 12, 24, 48 and 72 hours post-treatment. To confirm that cancer cells treated with ethanol and then HIFU lose their aggressiveness, we conducted a series of adhesion and spheroid culture experiments with treated cancer cells. Specifically, we measured the number of cancer cells adhered to TNF-α-activated endothelium under static conditions and tested the ability of the cells to form multi-cellular spheroids using a hanging drop method. The data (mean ± SEM) are the result of 3-4 independent experiments. Statistical significance was determined by Student’s t-test.

**RESULTS** Prostate cancer cells, treated or not with ethanol, significantly increased their expression of FasL immediately after being exposed to HIFU and continued to produce this molecule at a significantly higher amount than untreated (control) or ethanol alone-treated cells at 24 hours and 72 hours post-treatment (Fig. 1A). The highest expression of FasL was achieved for the combined treatment group. Similarly, the combined treatment led to significantly higher expression of Fas than any other treatment at both 24 and 72 hours (Fig. 1B). The expression of TNF-α receptor was also significantly increased in treatment group at both 24 and 72 hours (Fig. 1C). HSP70 expressed significantly higher in cancer cells exposed to HIFU than that in untreated or ethanol alone-treated cells (Fig. 1D). Our static adhesion assay showed that the cancer cells in the combined treatment group attached much rarely to TNF-α-activated vascular endothelium than the cells in other groups. The cells exposed to both ethanol and HIFU were unable to form three-dimensional tumor spheroids.

**CONCLUSIONS** Although Hsp70 plays a key role in cancer initiation and progression, its overproduction interferes with NF-κB signaling, thereby causing apoptosis and reduced expression of adhesion molecules required for metastasis. Here, we showed that focused ultrasound induces Hsp70 overproduction in prostate cancer cells and promotes cell apoptosis *via* an increase in expression of Fas, FasL and TNF-α receptor. All these factors lead to phenotypic changes in surviving cancer cells that reduce their aggressiveness.

**Fig. 2 (abstract P87).**
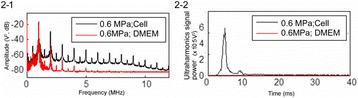
Panc-1 cells mixed with 1% SonoVue bubbles (black water) and water (as control, red curve) exposed to 0.6 MPa PNP, 1 KHz PRP and 30% duty cycle. Frequency domain of the recorded signal in a period (2-1). Power of broadband components as a funtion of exposure time (2-2)

### P86 Therapeutic effect of focused ultrasound combined with dendritic cell treatment for melanoma: preliminary study

#### Eun-Joo Park, Yun Deok Ahn, Yuri Cheon, Jae Young Lee

##### Radiology, Seoul National University Hospital, Seoul, Korea

###### **Correspondence:** Eun-Joo Park

**OBJECTIVES** As focused ultrasound (FUS) combined with microbubbles has been widely studied in cancer treatment, there is growing interests in immunotherapy combined with FUS as FUS treatment might trigger the immune response so that it results in therapeutic effects for cancer. To investigate the effects of FUS on the body’s immune system, this study was designed as a preliminary study that evaluates the therapeutic effects of antigen-pulsed dendritic cells (DC) with FUS treatment.

**METHODS** A total of 30 mice were inoculated with B16-F10 melanoma cells CFPAC-1 and divided into five treatment groups: control, antigen-pulsed DC only (DC), FUS only (FUS), and DC with FUS at two different FUS operating conditions DC-FUS1, DC-FUS2). Animals were treated on a weekly basis for three weeks and posttreatment monitoring was followed for one week.

**RESULTS** Animals in the group treated with DC and FUS2 which has higher mechanical index (MI) and short duty cycle (5%) showed slower tumor growth incomparison to control and DC only groups. Tumor growth in the group treated with FUS only also showed lower growth rate than DC only and control groups. However, the group treated with DC and FUS1 which has lower MI and long duty cycle (50%) showed faster tumor growth than DC only, DC and FUS2, and FUS2 only groups.

**CONCLUSIONS** Based on the result that FUS treatment with high MI and very short duration can enhance therapeutic effects of DC treatment, it is assumed that mechanical effects of FUS might be the main mechanism for enhanced treatment effects of DC therapy. In order to investigate the detail of FUS effects on different therapeutic outcome and to improve the treatment protocol for enhanced therapeutic effects of the combined treatment further study will be followed.

### P87 The long-term fate of the sonoporated pancreatic cancer cells is uncorrelated with the degree of the sonoporation

#### Lizhou Lin^2,1^, Caixia Jia^1^, Alfred C. Yu^3^, Lianfang Du^2^, Peng Qin^1^

##### ^1^Department of Instrument Science and Engineering, Shanghai Jiao Tong University, Shanghai, China; ^2^Department of Ultrasound, Shanghai First People’s Hospital, Shanghai Jiao Tong University, Shanghai, China; ^3^Department of Electrical and Computer Engineering, The University of waterloo, Waterloo, Alberta, Canada

###### **Correspondence:** Lizhou Lin

**OBJECTIVES** Sonoporation, refers to the reversible membrane perforation induced by the acoustic cavitation, has showed great potential for macromolecule delivery. Previous studies have determined the fate trend of the sonoporated cell. However, it is commonly believed that the sonoporation appeared to be heterogeneous due to discrete cavitation events adjacent to every single cell. The relationship between the long-term fate trend and the degree of the sonoporated cells is not still clear. This work aims to revealthe long-term fate of heterogeneously sonoporated cells.

**METHODS** The pancreatic cancer Panc-1 cells mixed with 1% SonoVue microbubbles and 40 KDa FITC-dextran were placed in a tissue mimicking chamber and exposed to 1-MHz ultrasound with 1 kHz pulse repetition frequency, 300 cycles and 0.6 MPa, and peak negative pressure (Fig. 1). Another 7.5MHz focused transducer was employed to passively detect the inertial cavitation dose (Fig. 2). The samples were firstly stained by Propidium Iodide at 30 min after exposure. After the sonoporated cells were identified by flow cytometry analysis, according to the relative fluorescence intensity (weak, medium and high) of the internalized FITC-dextran, the sonoporated cells were categorizedinto three sub-groups by flow cytometry sorting. After cultured for 6 H and 24 H and stained by Annex V-alex350 and Propidium Iodide, these three sub-groups ofsonoporated cells were detected to determine the apoptotic and necrotic ratio by flow cytometry analysis.

**RESULTS** The sonoporated cells were categorized into three sub-groups (sub-groups 1-3), which represented different degrees of sonoporation respectively (Fig. 3). The fluorescence intensity of the internalized FITC-dextran in sub-groups 2 and 3 was approximate 5.62-fold and 19.53-fold higher than that in sub-group 1, respectively. The apoptotic and necrotic ratio in all three sub-groups of the sonoporated cells gradually increased with the increasing culture time, compared with those in the control cells (Figs. 4 and 5). No significant difference in both the apoptotic (P > 0.05) and necrotic (P > 0.05) ratio were observed in three sub-groups of sonoporated cells which were cultured for 6 h and 24 h culture, respectively (at 6 H post-exposure, 2.48±1.65%,5.21±1.01 %, 5.05± 1.17 % and 5.95± 1.04 % apoptosis in the control and sub-groups 1-3 sonoporated cells, respectively. At 24 hour post-exposure, 4.40+2.81%,11.15± 2.48 %, 11.17± 4.93 % and 13.21± 3.78 % apoptosis in the control and sub-groups 1-3 sonoporated cells, respectively).

**CONCLUSIONS** Our results indicated some of the sonoporated cells would experience apoptosis and necrosis in the long-term after inertial cavitation. The apoptotic and necrotic ratio in the sonoporated cells exhibited no significant correlation with the degree of sonoporation. These findings preliminarily suggest that the signal, that triggers the apoptosis and necrosis of the sonoporated cells, may be not correlated with the physical damage caused by acoustic cavitation, but dependent on underlying cellular response.

**Fig. 3 (abstract P87).**
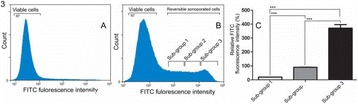
The sonoporated cells were catergorized into three sub-groups (1-3) according to the fluorescence of the internalized FITC-dextran. A. the fluorescence of the control; B. The fluorescence of the reversible sonoporated cells. C. the relative FITC of the three sub-groups 1-3

**Fig. 4 (abstract P87).**
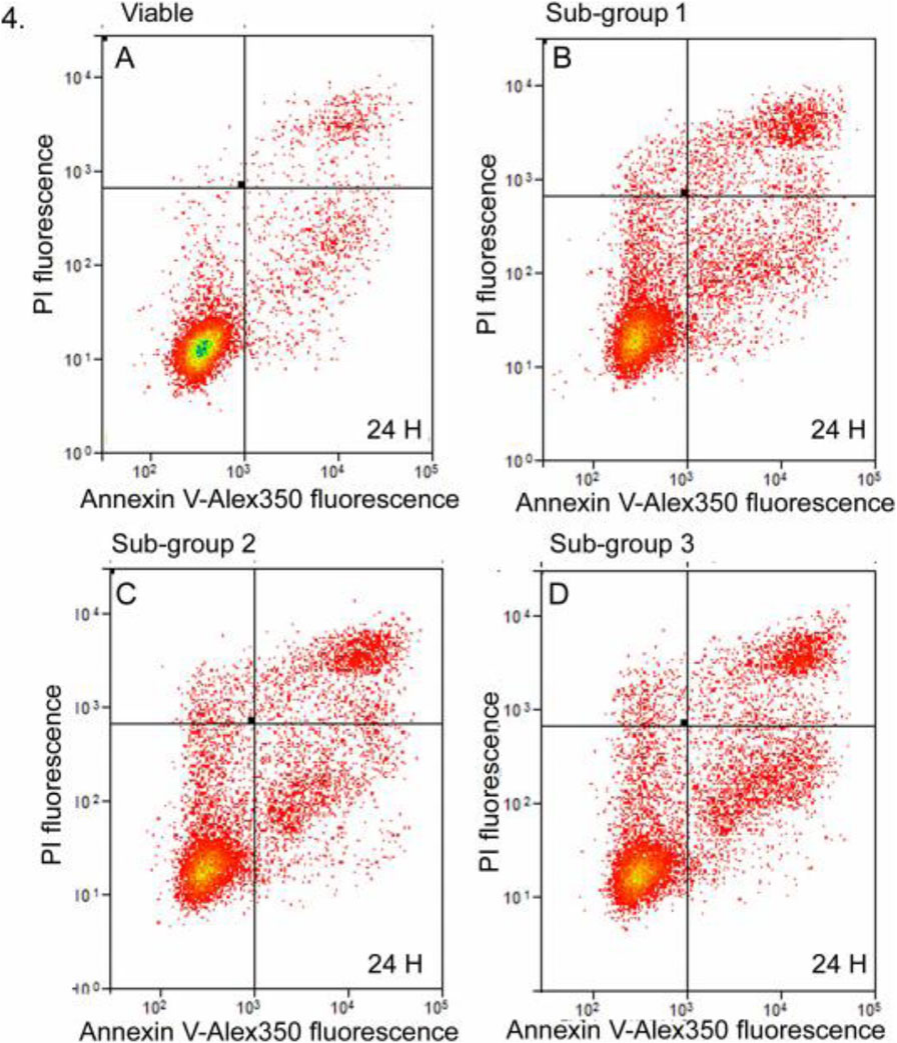
Three sub-groups (1-3) of the sonoporated cells were respectively cultured for 24 H after inertial cavitation, then stained by Annexin V-Alex 350 and PI, and detected by flow cytometry analysis (A) Viable cells, (B) Sub-group 1 sonoporated cells; (C) Sub-group 2 sonoporated cells; (D) Sub-group 3 sonoporated cells

**Fig. 5 (abstract P87).**
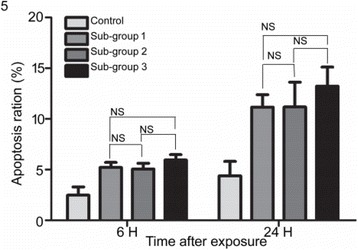
The apoptosis ratio of three sub-groups sonoporated cells at 6 H and 24 H after ultrasound exposure

**Fig. 1 (abstract P89).**
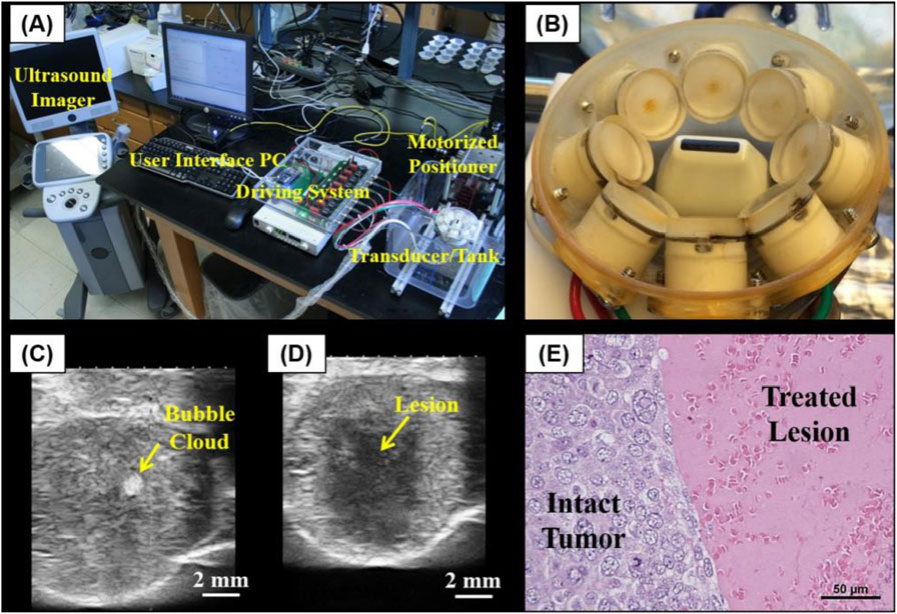
(A) A custom­built small animal system with a (B) 1 MHz therapy transducer was used to apply histotripsy to Hep3B tumors in an *in vivo* murine model. During treatment, the (C) bubble cloud and (D) tissue fractionation was visualized in real­time. After treatment, histological analysis showed precise lesions generatedinside the tumor

**Fig. 1 (abstract P91).**
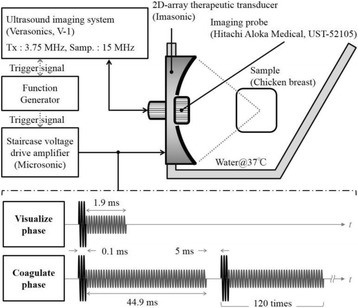
Schematic of experimental set up and HIFU sequence as push pulse and therapeutic beam

### P88 Tissue erosion at the fluid interface by dual-frequency HIFU excitation

#### Yisheng Lei, Yufeng Zhou

##### Nanyang Technological University, Singapore, Singapore

**OBJECTIVES** High-intensity focused ultrasound (HIFU) burst has been used to successfully erode the tissue or gel phantom at the interface of fluid. The performance of histotripsy or microtripsy [high peak negative pressure, p-, and pulse repetition frequency (PRF > 100 Hz) but short pulse duration (in the order of ms)] and those conventional HIFU bursts [moderate p-, long pulse duration (in the order of ms), and low PRF (a few Hz)] have been confirmed. In order to further understand the mechanism of tissue erosion and improve the capability, a new technology, using dual-frequency excitations, was proposed and investigated in this study.

**METHODS** The effect of frequency difference and modulation depth in the dual-frequency excitation on the produced erosion area and volume were quantitatively evaluated and compared to those of single-frequency excitation. In addition, the outcomes at the different PRFs were also compared. Bubble dynamics using different excitation strategies were captured by high-speed photography and passive cavitation detection (PCD). The acoustic field of dual-frequency excitation was simulated numerically and measured experimentally. The corresponding bubble oscillation was also simulated with the Gilmore model.

**RESULTS** Dual-frequency excitation is more effective to disintegrate the gel phantom and tissue than single-frequency excitation up to about 2 fold.

**CONCLUSIONS** In summary, this strategy can enhance the tissue erosion using the same output power, and operational parameters should be optimized for the bestoutcome. In vivo experiment will be carried out for the clinical translation.

### P89 Non-invasive liver cancer ablation using histotripsy in an *in vivo* murine model

#### Eli Vlaisavljevich, Tyler Gerhardson, Joan Greve, Shan Wan, Kim Ives, Timothy Hall, Theodore Welling, Zhen Xu

##### University of Michigan, Ann Arbor, Michigan, United States

###### **Correspondence:** Eli Vlaisavljevich

**OBJECTIVES** Current liver cancer ablation methods are primarily thermal-based and thus share inherent limitations such as inconsistent ablation in tissue with nonuniform heat dissipation patterns or incomplete tumor necrosis near major vessels. Recently, our group has developed histotripsy as a completely non-invasive liver cancer ablation method. Histotripsy is a non-thermal ultrasound ablation method that fractionates tissue through the precise control of acoustic cavitation. Previous experiments in an *in vivo* porcine model have shown the feasibility of using histotripsy to noninvasively create well-confined lesions in the liver through the intact chest, with sharp boundaries between treated and untreated tissue. In this study, the feasibility of using histotripsy for non-invasive liver cancer ablation was tested in an *in vivo* murine model.

**METHODS** Liver cancer patient-derived xenografts (Hep3B HCC cell line) were subcutaneously implanted in 8 NSG mice. When tumors reached ~1 cm, histotripsy was applied non-invasively using a custom-built 1 MHz histotripsy therapy system designed for small animal experiments (Fig. 1A/B). Guided by real-time ultrasound imaging, histotripsy was applied to the tumors using 1-2 cycle pulses, a pulse repetition frequency of 100 Hz, and an estimated *in situ* peak negative pressure >30 MPa.The targeted tumor volume was mechanically scanned using a robotic micro-positioner controlled by a PC console. After treatment, lesion characteristics were assessed using ultrasound imaging and MRI (7T small animal scanner), and the treated tissues were then harvested for gross analysis and histological evaluation. All procedures were approved by the ICUCA at the University of Michigan.

**RESULTS** Histotripsy-induced cavitation clouds were successfully generated inside the tumors of all subjects, with the bubble clouds clearly visualized as a hyperechoic zone on ultrasound imaging (Fig. 1C). Immediately after treatment, the histotripsy-induced tissue damage was visualized as a hypoechoic zone on ultrasound imaging (Fig. 1D). Gross and histological analysis demonstrated that histotripsy resulted in the complete fractionation of all tumor cells within the targeted region into an a cellular homogenate with no remaining cellular structures and sharp boundaries (<10 μm) between treated and untreated tissue (Fig. 1E). No gross damage to the surrounding tissue was observed in any subjects.

**CONCLUSIONS** The results of this study suggest that histotripsy has potential as a completely non-invasive liver cancer ablation method. Future work is ongoing to investigate long-term safety and biological response to histotripsy in relevant *in vivo* liver cancer models.

**Fig. 2 (abstract P91).**
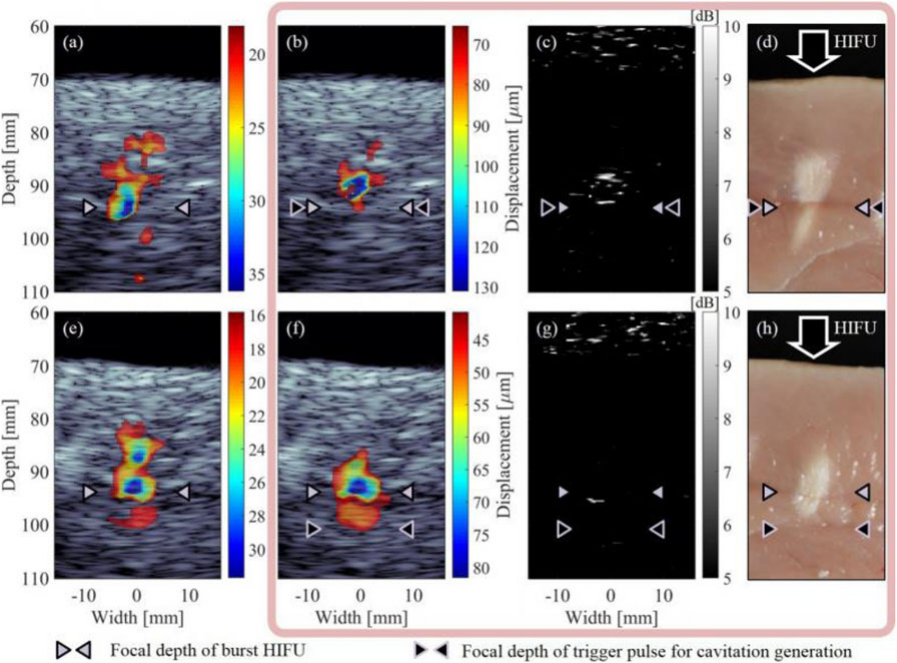
Distributions of axial displacements (b), (f), subtraction B­mode images (c), (g) and slices of samples after coagulation (d), (h) without and with a focal depth offset of 7mm, respectively, and without trigger pulses following push pulses (a), (e)

### P90 Comparison of ultrasound-guided high intensity focused ultrasound and surgery for abdominal wall endometriosis: a retrospective cohort study

#### Ling Zhao^2^, Jinyun Chen^1^, Chunquan Zhao^2^

##### ^1^College of Biomedical Engineering, Chongqing Medical University, Chongqing, China; ^2^Department of Obstetrics and Gynecology, The 1st Affiliated Hospital of Chongqing Medical University, Chongqing, China

###### **Correspondence:** Ling Zhao

**OBJECTIVES** To compare the safety and efficacy of high-intensity focused ultrasound (HIFU) and surgery for abdominal wall endometriosis.

**METHODS** With a retrospective cohort study design, fifty-four Chinese women with abdominal wall endometriosis who underwent ultrasound-guided HIFU between January 2012 and December 2014 were enrolled. In which, 29 cases included in surgery group, 25 cases in HIFU group. Technological success, adverse events and recurrent rate were assessed and compared between two groups.

**RESULTS** The clinical features are comparability, and the success rate was obtained of 100% both in the two groups. The clinical efficacy rate was 92% (22/25) in theHIFU group, and 100 % (29/29) in the surgery group. The numeric rating scales (NRS) after HIFU were significantly lower than those before the procedure from 6.92 to0.28 (P<0.05). During the mean follow-up of 32 months (range: 19-46 months), the duration pain relief were 39.00±30.02 months in the surgery group and 30.96±28.55 months in the HIFU group (P>0.05). Three cases (10.71%) experienced recurrence of pain in the surgery group, and 2 (8.00%) in the HIFU group. Adverse events occurred in 4 (13.79%) surgery cases and in 1 (4.00%) of HIFU cases respectively, without significant difference (P>0.05). Events in the surgery group included incision healing delayed and lung infection, and skin burn occurred in the HIFU group.

**CONCLUSIONS** HIFU appears to be safe and effective for the treatment of abdominal wall endometriosis.

### P91 Prediction of thermal lesion formation by short-pulse pre-exposure for cavitation-enhanced ultrasonic heating

#### Ryosuke Iwasaki^1^, Ryo Takagi^2^, Shin Yoshizawa^2^, Shin-ichiro Umemura^1^

##### ^1^Biomedical Engineering, Tohoku University, Sendai, Miyagi, Japan; ^2^Communications Engineering, Tohoku University, Sendai, Miyagi, Japan

###### **Correspondence:** Ryosuke Iwasaki

**OBJECTIVES** Acoustic cavitation microbubbles are known to enhance the heating effect of ultrasound. In high-intensity focused ultrasound (HIFU) treatment, utilizing microbubbles is expected to accelerate ultrasonic heating to reduce treatment time. However, it is not simple to control the position of generating cavitation bubbles. Therefore, it is necessary to visualize either the bubbles or the effective focal zone where HIFU is most absorbed. If such visualization is performed in prior to the cavitation enhanced HIFU treatment, both safety and effectiveness of the treatment will be ensured. The objective of this study is to compare the methods between visualizing the ultrasonic backscatter based on B-mode images and the visualizing the ultrasonic attenuation based on acoustic radiation force impulse (ARFI) technique.

**METHODS** Figure 1 shows the experimental setup and the sequences of HIFU exposure for pre-treatment focal zone visualization and tissue coagulation. A piezocomposite 2D-array HIFU transducer with both aperture diameter and geometrical focal length of 120 mm was placed in a water tank and connected to a staircase drive amplifier. A sector diagnostic array probe was set in the central hole of the transducer and connected to an ultrasound imaging system. For focal zone visualization, a high-intensity pulse called trigger pulse at an intensity of 60 kW/cm2 with a duration of 0.1 ms for generating cavitation bubbles was followed by a moderate-intensity HIFU burst at an intensity of 3 kw/cm2 with a duration of 1.9 ms for inducing displacements. A chicken breast was used as a sample tissue. Before and immediately after this push pulse exposure, RF data were acquired *via* high-speed ultrasound imaging. The distribution of axial displacements between before and after push pulse exposure was calculated from the phase shift in 1D cross-correlation. After that, tissue was coagulated by the repetition of the trigger pulse followed by a HIFU burst with a duration of 44.9 ms, which was continued for 6 s with a duty cycle of 90%. The resulting tissue coagulation was compared with the distribution of the HIFU induced displacement and the B-mode image.

**RESULTS** Figure 2 shows the distributions of HIFU induced displacements without and with the trigger pulse, the subtraction B-mode images, and the gross pathologiesof the coagulated tissue. The region of the heat source seems to have been shifted backward from the HIFU focal point. With the trigger pulse, HIFU seems to have been shielded by the cavitation bubbles. To test this, an offset of 7 mm was given to the focal length of the trigger pulse. By comparing Fig. 2(b)-(d) and Fig. 2(f)-(h), it is obvious that the offset extended the area of coagulation deeper, which was well predicted by the effective focal zone by the HIFU induced displacements. The regions of high brightness in the subtraction B-mode images were thought to be the regions where cavitation bubbles were generated.

**CONCLUSIONS** In this study, we proposed HIFU induced ARFI imaging employing ultrasonic high-speed imaging to visualize the effective focal zone of HIFU. The results showed that the proposed method was effective for prediction of thermal lesion formation induced by HIFU heating even under difficult situation with enhancement by cavitation bubbles.

**Fig. 1 (abstract P93).**
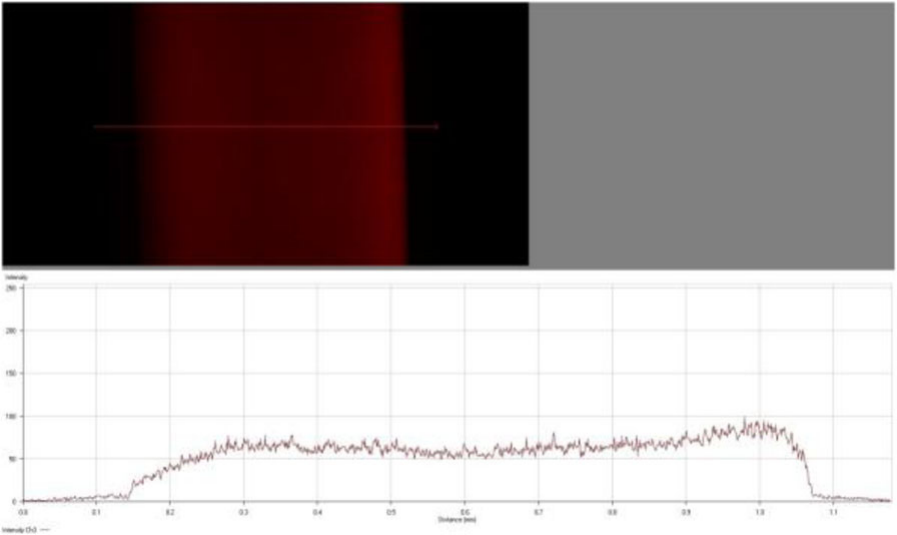
Control sample, no ultrasound applied. The X­direction is along the ultrasound beam, the Y­axis is the fluorescent intensity. The profile shows the cross­section the sandwich gel

**Fig. 2 (abstract P93).**
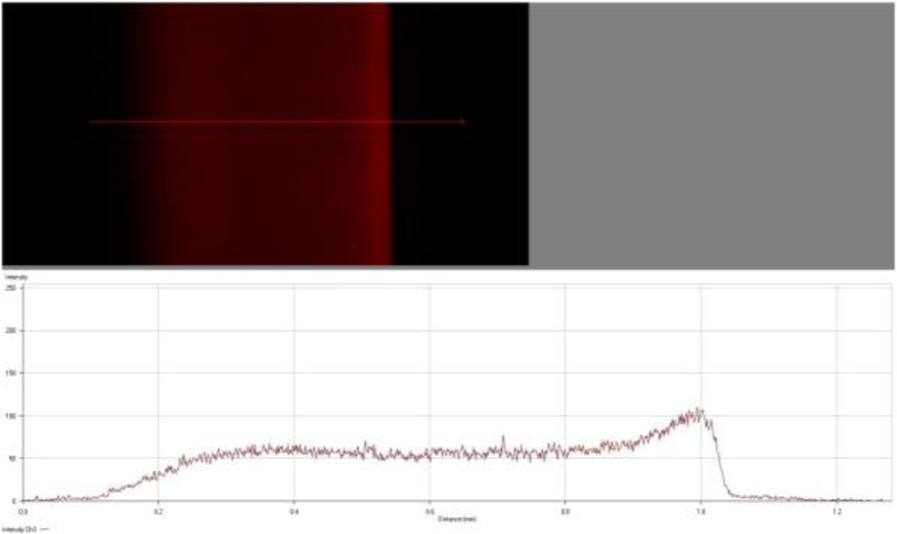
Control sample, no ultrasound applied. The X­direction is along the ultrasound beam, the Y­axis is the fluorescent intensity. The profile shows the cross­section the sandwich gel

### P92 Research on EFT immunity of ashi ultrasonic therapeutic apparatus based on statistical analysis

#### Zhiming Zhong, Fangwei Ye, Yang Liu

##### Biomedical Engineering Collage, Chongqing Medical University, Chongqing, China

###### **Correspondence:** Zhiming Zhong

**OBJECTIVES** Ashi ultrasonic therapeutic instrument can effectively alleviate the chronic soft tissue injury pain, the EFT immunity influences the therapeutic effect ofthe equipment. In order to diminish the randomness in the EFT immunity test, it is essential to adopt statistical analysis for research on EFT immunity of Ashi ultrasonic therapeutic apparatus.

**METHODS** The parameters of EFT generator were set according to test standard GB/T 17626.4 or IEC 61000-4-4, but the EFT initial voltage was set as 200V. The power line of Ashi ultrasonic therapeutic apparatus was injected with EFT. Then, the operative mode of equipment was observed as a evaluation criteria for judging whether the apparatus was disturbed. If not, the EFT voltage was increased with a step 200V, and continuing test after 30 seconds until that the equipment was disturbed. Then, one test was finished and the EFT voltage at this time was record, which was called EFT interference threshold voltage. According to the above method, the EFT immunity test was carried out in many times for obtaining multiple EFT interference threshold voltage data which was used to solve unknown parameters in two parameter Weibull distribution model through Maximum Likelihood Method. The distribution law of EFT interference threshold voltage of Ashi was analyzed by theprobability density function (PDF) and cumulative distribution function(CDF) curve of Weibull distribution model which has been verified by chi-square goodness of fittest.

**RESULTS** According to the result of chi-square goodness of fit test, two parameter Weibull distribution model was available for research on distribution law of EFT interference threshold voltage of Ashi ultrasonic therapeutic instrument. The PDF and CDF curves prove that EFT interference threshold voltage of Ashi equal 900V, and the EFT with different voltage has different interference probability for Ashi.

**CONCLUSIONS** The test standard GB/T 17626.4 or IEC 61000-4-4 proves that the standard voltage in the EFT immunity test equal 1000V which is the actual output voltage, the value greater than EFT interference threshold voltage of Ashi 900V. Therefore, the apparatus cannot pass EFT immunity test in great probability. The long data communication cable is coupled by EFT easily, which is main reason for Ashi cannot pass test probably. So, it is essential to shorten properly power line or data communication cable in the equipment. The statistical analysis is scientific and useful for obtaining EFT interference threshold voltage of equipment, which has important significance for the research on EFT immunity. Analyzing EFT immunity of equipment based on statistical analysis could decrease effectively randomness in the test result. And the distribution law of EFT interference threshold voltage of instrument will be analyzed accurately and clearly by statistical model.

### P93 Experimental study of the promotion of diffusion process in a biofilm by low intensity ultrasound

#### Dong Ma

##### Physics, University of Vermont, Burlington, Vermont, United States

**OBJECTIVES** Experimentally to show the Low intensity ultrasound can dramatically enhance nanometer liposome penetration into the biofilm films.

**METHODS** The biofilm model sample was made of three layers of 1% agarose gel; each layer has the thickness of 0.5 mm. The first layer of agarose contains fluorescent beads (diameter: 100 nm) was made in a petri dish, after the agarose was cooled down, a second layer was made on top of the first layer; at this point a two layer agarose gel was made. Flip it over, and made the third layer on top, then we had a three-layer gel with a fluorescent layer sandwiched between two non-fluorescent agarose gels. An ultrasonic tone burst (frequency=2.25 MHz, 10% duty cycle and spatially and temporally averaged intensity, ISATA 4.4 W/cm2) generated by a transducer of diameter 1.9 cm was used to treat the sample for 10 minutes.

**RESULTS** When ultrasound was applied, the diffusion distance (0.8 mm) is much longer than those without ultrasound (0.1mm). Figures 1 and 2 show that there are steep drops on the right side (which we are interested in), Figs. 3 and 4 show a very different profile.

**CONCLUSIONS** Certain intensity and exposure time of ultrasound can enhance the diffusion effect of small beeds solution in the agarose gel.

**Fig. 3 (abstract P93).**
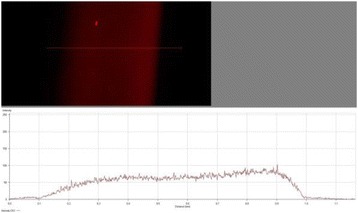
Ultrasound applied. The X­direction is along the ultrasound beam, the Y­axis is the fluorescent intensity. The profile shows the cross­section the sandwich gel

**Fig. 4 (abstract P93).**
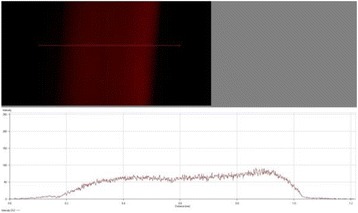
Ultrasound applied The X­direction is along the ultrasound beam, the Y­axis is the fluorescent intensity. The profile shows the cross­section the sandwich gel

**Fig. 1 (abstract P94).**
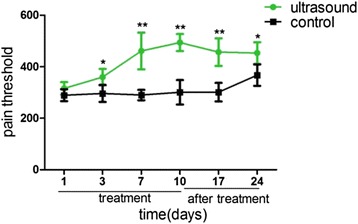
See text for description

**Fig. 2 (abstract P94).**
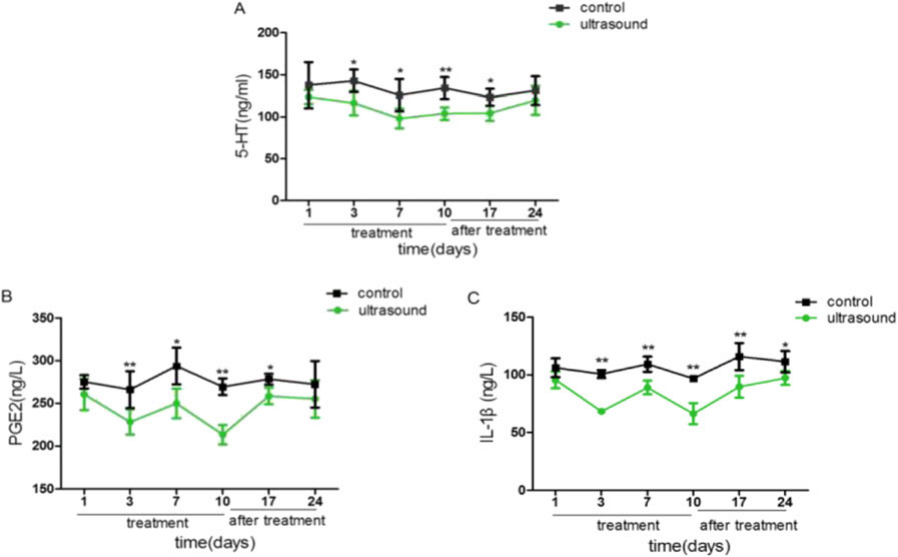
See text for description

### P94 The effect of focused ultrasound to treat the soft tissue injury on rabbit models

#### Dandan Liang

##### Chongqing Medical University, Chongqing, China

**OBJECTIVES** To evaluate the efficacy of focused ultrasound in the treatment of soft-tissue injury and preliminary discussed mechanism.

**METHODS** Ethics statement: The Ethics Committee of Animal Experiments at Chongqing Medical University (Production License No. SCXK 2012-0001) approved the experiment and animal care protocols. Animals: Male and female New Zealand White rabbits (3-4 months old, 2.0–2.5 kg) were chosen from the Animal Center at Chongqing Medical University. All animals were housed in individual cages with a 12:12-h light–dark cycle and a temperature- controlled environment (24 ± 2°C) and given a standard laboratory diet with drinking water at liberty. The animals were adapted to their environment at least 1 week before the experiment started. Soft-tissue injury model construction by hammer blow: As previously reported, the rabbits were fixed in the lateral and outside of the left hind was shaved by shaver and hit thrice on the thigh muscle by a cylindrical hammer (200 g in weight, 2.8 cm in bottom diameter), which fallen freely and vertically from inner of a hard smooth plastic tube (12 cm in length and 3 cm in inner diameter).The act repeated once every two days. When struck for four weeks and bred normally for three days later, the closed soft tissue injury was formed without femoral fractures. Intervention: All model rabbits were grouped into two groups randomly:ultrasound treatment group and non ultrasound group. The ultrasound group received a focused ultrasound treatment by a fixed and mobile method, which rabbits were treated fixedly for 20s, then taken a 5s break, lastly treated by mobile method with, 5–10 mm/s for 60s. They were treated per day for continuous ten days. Pain threshold determination: After treatment 30min, the pain threshold was measured by Analgesy-Meter. The rabbit was fixed and pressured gradually by the forcing bar and the value, the animal's pain threshold, was recorded when the animal retracted its leg. Measurement of PGE2,5-HT and IL-1β concentrations: The muscle 1g was homogenized in 2ml phosphate buffered solution (PBS pH 7.4). Then centrifuged and the supernatant was collected to detect the prostaglandin-E2(PGE-2),5-hydroxytryptamine(5-HT) and interleukin-1 beta (IL-1β) with an enzyme immunoassay at the 1st, 3rd, 7th, 10th, 17th, 24th days after the first of treatment. The parameter was measured according to the protocols of respective kits (Shanghai Hushang Bioengineering Institute, China). Statistical analysis: Results were expressed as mean ± standard deviation (SD). SPSS 19.0 (SPSS Inc., Chicago, IL, USA) was used for all statistical analyses. Independent samples t-test was used to analyze all experimental data. P < 0.05 (95% confidence interval) was considered statistically significant.

**RESULTS** Pain threshold value: When the stimulation intensity was increased gradually, the rabbits would show muscle contraction because of the acute pain. The pain threshold was recorded when the muscle contraction at the first time. The pain threshold value increasing of ultrasound group were statistically significant compared with the control group on the 3rd and muscle contraction at the first time. The pain threshold value increasing of ultrasound group were statistically significant compared with the control group on the 3rd and 24th (P < 0.05), 7th, 10th and 17th day (P < 0.01) (Fig. 1).Inflammation levels: The levels of the 5-HT (Fig. 2A) and PGE-2 (Fig. 2B) in the injured hind leg muscle of rabbit were reduced largely in focused ultrasound irradiation group from the 3rd dayto 17th day after the first treatment (P < 0.05), and the IL-1β level appeared a decreasing trend until the 24th day from the first treatment (P < 0.05) (Fig. 2C).

**CONCLUSIONS** It can be concluded that the focused ultrasound device used in this study is safe and effective in the treatment of soft-tissue injury by cutting inflammation factor expression and increasing pain threshold.

**Fig. 1 (abstract P95).**
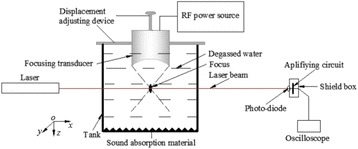
The normalized frequency spectrum obtained from the simulation of the theoritical model is consistent with the experiment results

**Fig. 1 (abstract P97).**
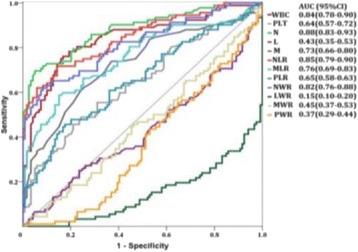
Comparison of differential peripheral blood parameters by the receiver operating characteristic curve. AUC>0.70 was considered to have a certain diagnostic value

### P95 Detection of harmonic signal of the focused ultrasound based on acousto-optic effect

#### Fu Li, Hua Wang

##### Chongqing Medical University, Chongqing, China

###### **Correspondence:** Fu Li

**OBJECTIVES** To explore a non-invasive method for detecting the harmonic signal of high intensity focused ultrasound (HIFU)

**METHODS** When a parallel laser beam passed through the focus of the focused ultrasonic field, the light signal was converted into an electrical signal by a photodiode in the distance, and the electrical signal was simulated by the Matlab. To verify the theoretical model, a focused ultrasonic transducer was used as the source of ultrasonic emission, and it was placed in degassed water. A He-Ne laser was used to transmit beam crossing the focus of the sound field, and the optical signal whose crossed focus was received by a photodiode (it was connected to the photoelectric detection circuit) in the distance, the output signal of the photoelectric detection circuit was collected by a digital oscilloscope, and the spectrum of the signal was obtained by Matlab (Fig. 1). Comparison the normalized frequency spectrum of theoretical and experimental obtained.

**RESULTS** The normalized frequency spectrum obtained from the simulation of the theoretical model is consistent with the experiment results.

**CONCLUSIONS** The method proposed in this paper is feasible to detect the harmonic signal of HIFU field, and it also lays a theoretical and experimental foundation tomeasurement the sound pressure of the nonlinear HIFU at focus.

**Fig. 2 (abstract P97).**
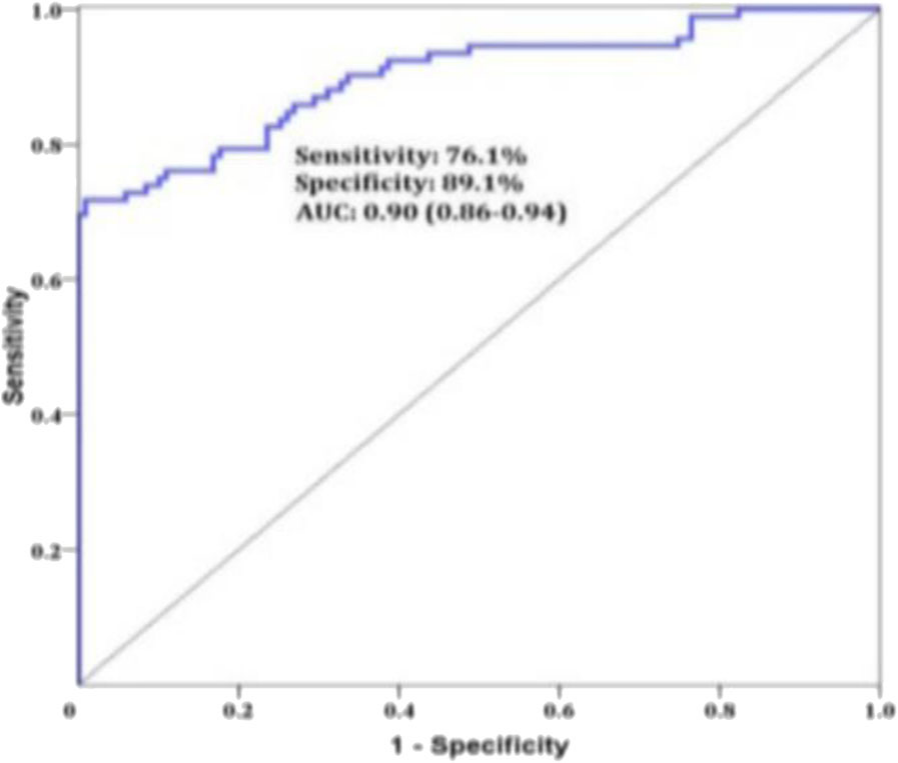
Receiver operating characteristic curve analysis for combined with six markers

### P96 Effect of cavitation induced by High Intense Focused Ultrasound (HIFU) on tungsten filament and stainless steel filament at 10 MPa static pressure

#### Yurong Zhang^1,2^, Faqi Li^1,2^, Xiaobo Gong^2^, Qi Wang^2^, Guangrong Lei^2^, Zhibiao Wang^1,2^

##### ^1^State Key Laboratory of Ultrasound Engineering in Medicine Co-founded by Chongqing and the Ministry of Science and Technology, College of BiomedicalEngineering, Chongqing Medical University, Chongqing, China; ^2^National Engineering Research Center of Ultrasound Medicine, Chongqing, China

###### **Correspondence:** Yurong Zhang

**OBJECTIVES** To illustrate the intense cavitation and high energy density induced by spherical cavity transducer with open ends.

**METHODS** We processing on tungsten filament and stainless steel filament with the same diameter based on the extreme transient condition, such as high pressure and high temperature during cavitation induced by continuous high intense focused ultrasound (CHIFU) and pulsed high intense focused ultrasound (PHIFU) at 10 MPa static pressures. The images are taken using a high speed camera, and scanning electron microscope (SEM) fractographic analysis is also conducted.

**RESULTS** The 0.2 mm diameter tungsten filament and stainless steel filament were severed by CHIFU (6000 W of electric power) cavitation within 1.39 s and 0.56 s, respectively. PHIFU (1,5000 W of electric power) cavitation could severed 0.2 mm diameter tungsten filament within 0.22 s. SEM fractographic analysis showed that the fracture of stainless steel filament was general fatigue fracture, but the fracture of tungsten filament was relatively complex, which might be involved annealing andrecrystallization texture.

**CONCLUSIONS** The tungsten filament is more difficult to be severed by acoustic cavitation than stainless steel filament, and HIFU can effectively improve plastic of tungsten filament. Next, we hope to calculate the pressure and temperature of cavitation core region, known as hotspot, from the perspective of micro fracture mechanicsto illustrate the high energy density of our system.

### P97 The value of the parameters in the peripheral blood routine test for the preoperative diagnosis of uterine sarcoma: a review of a multicentre study in western China

#### Dan Li, Wenzhi Chen, Jinyun Chen

##### College of Biomedical Engineering, Chongqing Medical University, ChongQing, China

###### **Correspondence:** Dan Li

**OBJECTIVES** The current study was to examine the accuracy of preoperative diagnosis of uterine sarcomas in western China and to evaluate the efficacy of the indicators in the peripheral blood routine test for the differential diagnosis between uterine sarcomas and leiomyomas.

**METHODS** A total of 102 patients with uterine sarcoma were evaluated, underwent surgery in the first affiliated hospital of Chongqing Medical University, the DapingHospital affiliated to the Third Military Medical University, People’s liberation Army of China and the affiliated Hospital of Southwest Medical University, covering from January 1st 2010 to December 1 st 2015. Meanwhile,119 women with leiomyoma, complete clinical and pathological information documented at the time of surgery were selected as controls between January 1st 2010 to December 1 st 2015. Study parameters included age at the time of surgery, clinical findings, blood test results, imaging examinations results (specifically ultrasonography and magnetic resonance imaging [MRI]), endometrial cytology findings, postoperative pathological diagnosis and follow-up. Receiver operating characteristics (ROC) analysis was used for specificity and sensitivity estimates. The resulting area under the curve (AUC) indicates the average sensitivity of a marker over the entire ROC curve. Multivariate analysis was performed using nonlinear model of Logistic regression analysis and partial leastsquares-discriminant analysis.

**RESULTS** At the final preoperative diagnosis, 59.8% (61/102) of uterine sarcomas were diagnosed as having malignant disease. 6 indexes including the WBC, neutrophil, monocyte, NLR, MLR and NWR had a certain diagnostic value evaluated by the ROC curve (AUC>0.70) (Fig. 1). A comprehensive system combined with by six markers for identification of uterine sarcoma were analyzed by the ROC curve,nonlinear model of Logistic regression analysis and partial least squares-discriminant analysis (Fig. 2).The AUC of this comprehensive diagnosis system was 0.90 (95%CI, 0.86to 0.94; sensitivity =76.1%,specificity =89.1%).Logistic regression analysis and partial least squares-discriminant analysis showed that the comprehensive system combined with by six markers had a better value of differential diagnosis between uterine sarcomas and leiomyomas.

**CONCLUSIONS** The comprehensive system combined with by six markers (WBC, neutrophil, monocyte, NLR, MLR and NWR) in the peripheral blood routinetest, which is convenient, reproducible, and inexpensive, had a potential value of differential diagnosis between uterine sarcomas and leiomyomas (Fig. 3).

**Fig. 3 (abstract P97).**
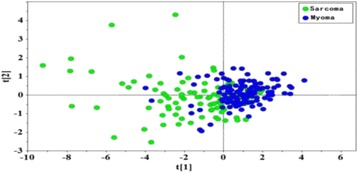
Partial least squares-discriminant analysis (PLS-DA) combined with six markers

**Fig. 1 (abstract P98).**
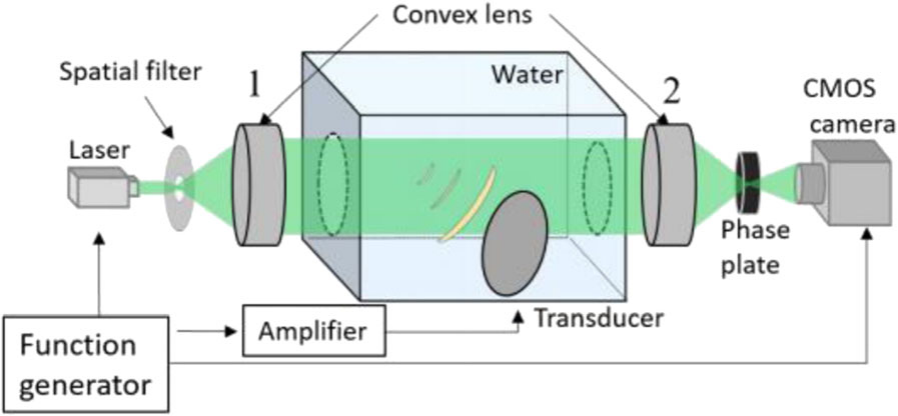
Optical phase contrast measurement setup

**Fig. 2 (abstract P98).**
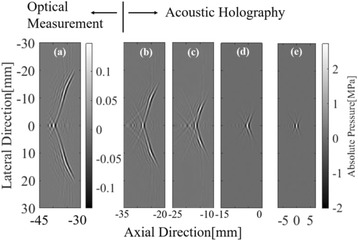
Optically measured pulsed pressure field upstream the focal point (a), and the numerically simulated fields (b) 8.6 µs (c) 17.1 µs (d) 25.7 µs after the input, and(e) at the focus

### P98 Quantitative measurement of high-intensity pulsed ultrasound pressure field using combination of optical phase contrast method and acoustic holography

#### Takuya Nakamura^1^, Hiroki Hanayama^2^, Ryo Takagi^2^, Shin Yoshizawa^2^, Shin-ichiro Umemura^1^

##### ^1^Biomedical Engineering, Tohoku University, Miyagi, Japan; ^2^Communication Engineering, Tohoku University, Miyagi, Japan

###### **Correspondence:** Takuya Nakamura

**OBJECTIVES** In recent years, ultrasound has been widely used for therapeutic as well as diagnostic purposes. To assure safety and efficacy of such application, accurate measurement of ultrasonic pressure field is necessary. Hydrophone scanning is the most common method to measure ultrasound pressure field. However, this method requires very long scanning time and has the risk of disturbing pressure field. In this study, pulsed ultrasound pressure field was quantified by numerical acoustic holography based on a fast optical measurement using a phase contrast method in combination with a CT algorithm.

**METHODS** Figure 1 shows the measurement setup of the optical phase contrast method. Ultrasound pressure field in water creates a modulation of the refractive index, which further modulates the phase of the light passing through the field. The non-diffracted component of the light was focused exactly at the focal point of the second convex lens, while the other component diffracted due to the phase modulation was laterally slightly away from the focal point. By shifting the phase of the non-diffracted component by the phase plate typically by 90 degrees, the phase modulation of the diffracted component was converted to amplitude modulation through interference with the phase-shifted non-diffracted component and then measured by the camera. The numerical calculation steps based on the measurement were as follows,1, An upstream field in which the optical phase does not wrap and the effect of nonlinear propagation can be ignored was chosen.2, The 3D pressure field was reconstructed from the projected 2D data from measurement by a CT algorithm.3, Nonlinear ultrasonic propagation was simulated using the obtained upstream pressure field as the input. In this study, an axisymmetric 8-element annular array transducer (outer diameter: 80 mm, inner diameter: 36 mm, focal length: 80 mm, center frequency: 1.4 MHz) wasdriven at 65.6Vpp, the pressure field 40 mm upstream the focal point was measured, and the numerical simulation based on a pseudo spectral method was performed. The pressure ±5 mm from the focal point was also measured by scanning a fiber optic hydrophone (Onda, HFO-690) to compare with the proposed method. High-intensity focal field at a therapeutic level can be calculated by multiplying the measured low-intensity pressure before inputting it to the simulation of nonlinear propagation. Here, it was assumed that the nonlinear propagation at the upstream of the measured area can be ignored even when the acoustic pressure is multiplied.

**RESULTS** Figure 2 (a) shows the optically measured axisymmetric pulsed pressure field upstream the focal point, and Fig. 2 (b)-(e) show the results from the numerical simulation using it as the input. Figure 3 shows the comparison of the obtained focal field between hydrophone scanning and the proposed method. The pressure distribution in axial and lateral directions is shown. The absolute positive and negative pressures measured by hydrophone were 2.89 and -1.75MPa, whereas they were 2.72 and -2.03MPa determined by the optical measurement. Good agreement was observed for the waveforms and absolute pressure in both directions.

**CONCLUSIONS** Pulsed ultrasound pressure field was quantified by numerical acoustic holography based on a fast optical measurement using a phase contrast method in combination with a CT algorithm. Both pressure waveform and absolute pressure from the proposed method agreed well with that from hydrophone scanning. Further studies underway are applications to high pressure field at a therapeutic level and to continuous wave field such as that of HIFU (high-intensity focused ultrasound).

**Fig. 3 (abstract P98).**
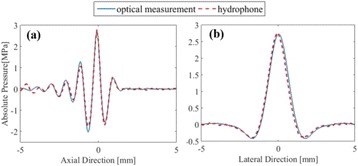
Comparison of pressure waveform between proposed method and hydrophone scanning in axial (a) and lateral (b) directions

**Fig. 1 (abstract P99).**
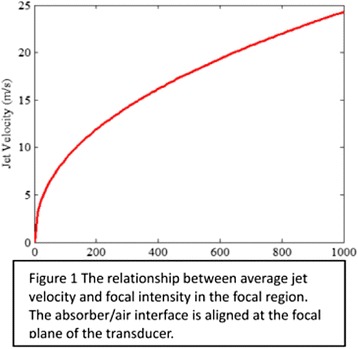
See text for description

**Fig. 2 (abstract P99).**
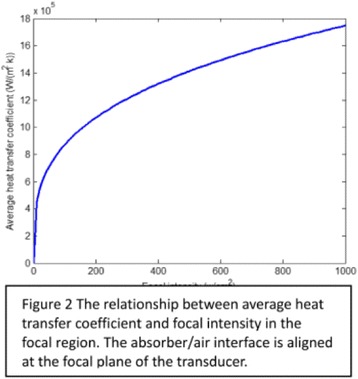
See text for description

### P99 Boundary analysis of absorber irradiated by the focused ultrasound

#### Ying Yu^1^, Guofeng Shen^2^, Chunlei Lei^1^, Helin Zhang^1^

##### ^1^School of Computer, Jiangxi University of Traditional Chinese Medicine, Nanchang, Jiangxi, China; ^2^School of Biomedical Engineering, Shanghai Jiao TongUniversity, Shanghai, Shanghai, China

###### **Correspondence:** Ying Yu

**OBJECTIVES** Characterization of focused ultrasound field is important factor for both efficacy and safety of clinical treatment. Recently, the infrared imaging has been used to estimating the intensity of focused acoustic field. The principle of this method is estimating the absolute intensity and relative distribution of focused ultrasound by the temperature elevation at the surface of an absorber which irradiated by the focused ultrasound. There were two interfaces, absorber/air and water/absorber, with large differences in thermal physics properties. In theory, the derived relationship between incident intensity and surface temperature is determined by the boundary condition of those two interfaces. Boundary condition of two interfaces is investigated theoretically in this study.

**METHODS** At the absorber/water interface, acoustic streaming generated by ultrasound lash the interface. In the focal region, the ultrasound field distributed like a cylinder-shape, so in this paper, the flow heat transfer analogy for a single round jet stream. According the time-average energy conservation from the transducer to the focal region, the relationship between average jet velocity and heat transfer coefficient at absorber/water interface and focal intensity can be derived, as show in Figs. 1 and 2. At the absorber/air interface, due to the ultrasound irradiation, the surface temperature elevated may cause the natural convection and radiant heat transfer. So the combined heat transfer can be expressed as ΦTotal =ΦConvection + ΦRadiation = AhcΔT +AhrΔT = AhTotalΔT, where htotal is combined surface heat transfer coefficient, hc is convective heat transfer coefficient, hr is radiant heat transfer coefficient, T is the temperature elevation. The relationship between transfer coefficient and temperature change is shown in Fig. 3.

**RESULTS** According to the boundary analysis, the average heat transfer coefficient at absorber/water interface has no significant impact to the absorber/air temperaturechanges, for absorber's thicknesses from 1~4 mm, as shown in Table 1. When the surface temperature changes below 100 °C, the results from combined surface heattransfer and heat transfer was closed, the difference of maximum temperature elevation and temperature change rate less than 0.7%, as show in Table 2.

**CONCLUSIONS** According the study, the boundary condition at water/absorber interface can be treated as infinite and constant temperature boundary conditions, when the heating time is very short. And at absorber/air interface, the heat conduction plays a major role, when the interface temperature changed below 100 °C.

**Fig. 3 (abstract P99).**
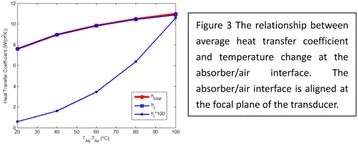
See text for description

**Table 1 (abstract P99).**
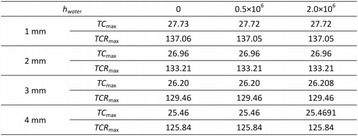
The table of maximum temperature change rate with different average heart transfer coefficient at absorber/water interface

**Table 2 (abstract P99).**
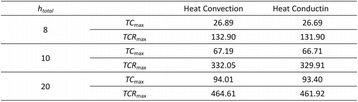
The table of maximum and the maximum temprature change rate with different average heat transfer coefficient at absorber/water interface and heart conduction

### P100 An inverse method for estimating the acoustic intensity in the HIFU field by infrared thermometry

#### Guofeng Shen^2^, Ying Yu^1^, Chunlei Lei^1^, Helin Zhang^1^

##### ^1^School of computer, Jiangxi University of Traditional Chinese Medicine, Nanchang, Jiangxi, China; ^2^School of Biomedical Engineering, Shanghai Jiao Tong University, Shanghai, Shanghai, China

###### **Correspondence:** Guofeng Shen

**OBJECTIVES** The 3D acoustic field distribution of HIFU is not only the key parameters for evaluating the quality of the transducer, but also an important indicator of the security and efficiency of HIFU system. Recently, a new method which based on infrared (IR) imaging was introduced. Authors (A. Shaw, et al and M. R. Myers, et al) have established the relationship between absorber surface temperature and incident intensity during the absorber was irradiated by the transducer. Theoretically, the shorter irradiating time makes estimation more in line with the actual results. In this study, an inverse method was introduced to estimating the acoustic intensity of HIFUfield using the surface temperature (Fig. 1).

**METHODS** The physical definition of this inverse problem is to reconstruct the unknown incident intensity distribution by measuring the thermal field of the absorber asa function of time. So, the thermal field of the absorber was measured with the IR camera not only during the heating time but also including the cooling time and preheating time. The algorithm for the solution consists of 9 steps.1) choose an initial guess2) solve the direct problem to obtain the surface temperature 3) solve the adjoint problem to find the gradient to the function J' 4) compute the conjugate coefficient 5) compute the search direction 6) solve the sensitivity problem with input data 7) compute the step size in the search direction 8) compute the new estimate 9) interrupt the iterative procedure if the stopping criterion is satisfied. Otherwise repeat the iteration until convergence is achieved.

**RESULTS** The method proposed for quantitative assessment of acoustic intensities using IR camera with inverse method has a satisfactory percentage difference, in both maximum intensity (< 13.73 %) and -6 dB beam width (< 10.04 %) in focal region, in comparison to the theoretical simulation using a three-layer medium model (Fig. 2).

**CONCLUSIONS** The percentage difference increase with the heating time with zero mean noise, but decrease with heating time when the noise can be ignored.

**Fig. 1 (abstract P100).**
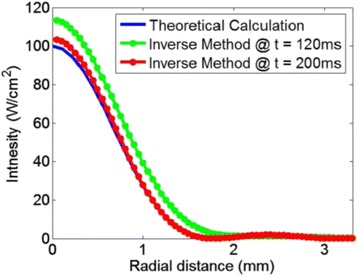
Comparison of the inverse method (red and blue dot line) and theoretical simulation (blue solid line) for heating of 120 and 200 ms

**Table 1 (abstract P100).**
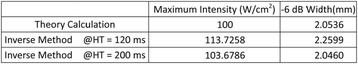
See text for description

**Fig. 2 (abstract P100).**
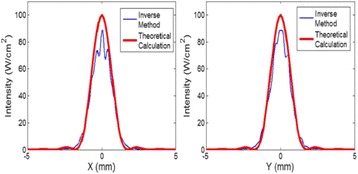
Comparison of the inverse method (blue dot line) and theoretical simulation (red solid line) for heating of 120 ms

**Table 2 (abstract P100).**
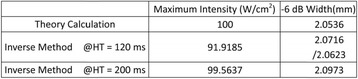
The difference between the maximum intensity on-axis using the proposed method in the simulated theoretical prediction for different heating time with same noise

### P101 Comparation of the focusing ultrasound of propagating and standing wave in sound field and bioeffects

#### Man Luo^2,1^, Faqi Li^2,1^, Zonggui Chen^2,1^, Yurong Zhang^2,1^

##### ^1^National Engineering Research Center of Ultrasound Medicine, Chongqing, China; ^2^State Key Laboratory of Ultrasound Engineering in Medicine Co-Founded by Chongqing and the Ministry of Science and Technology, College of Biomedical Engineering, Chongqing Medical University, Chongqing, China

###### **Correspondence:** Man Luo

**OBJECTIVES** This study aims to investigate the differences of this focusing modality with the conventional one in sound field distribution and the process of lesion formation under the same acoustic pressure.

**METHODS** Setting two identical transducers in opposition whose internal surface were on the same spherical surface and driving simultaneously to achieve the focusing of standing wave. And then driving one transducer and sheltering another one from ultrasound beams with sound absorbing materials to get the focusing of propagating wave. The sound field distribution of the two focusing modalities were acquired by fiber-optic hydrophone. The change of the acoustic pressure of focus with the increase of power were measured by fiber-optic hydrophone also and waveform of acoustic pressure were recorded to analyze nonlinear effects of the two focusing modalities on the same focusing pressure (16MPa). Linear fitting of acoustic pressure squared with power extrapolate the powers of getting the experimental acoustic pressure (18MPa). Tissue mimicking phantom were exposed with the extrapolating powers of the two focusing modalities. Meanwhile, the processes of lesion formation in phantom were recorded with high-speed camera to analyze the differences of lesion formation between the two focusing modalities and the focal 3-6MHz broadband emissions were recorded with PCD to analyze cavitation.

**RESULTS** Sound field distribution of propagating and standing wave focusing (Fig. 1) There were obvious differences of sound field distribution of sound propagation axis between propagating and standing wave focusing. Comparation of nonlinear effects of the two focusing modalities on the same focal pressure (Fig. 2) The nonlinear effect of propagating wave is stronger than that of standing wave because of the stronger distortion of waveform. Recording the formation of lesion in phanton with high-speed camera (Fig. 3) Lesion formation of the propagating wave focusing modalities has been formed the “ellipsoidal” lesion whose major axis was along with wave propagation direction (Fig. 3a). However, lesion formation of focusing of standing wave has been formed the “gourd string” lesion (Fig. 3b). The focal 3-6MHz broadband emission during exposure collected with PCD on the same focusing pressure (Fig. 4) There have been no significant differences of the cavitation signal between the two focusing modalities.

**CONCLUSIONS** 1. Lesion formed in the condition of standing wave focusing is obviously different from that in the condition of propagating wave2. Focusing modality of standing wave need lower power than that of propagating wave to get the same acoustic pressure3. Nonlinear effect plays an important role in the formation of lesion.

**Fig. 1 (abstract P101).**
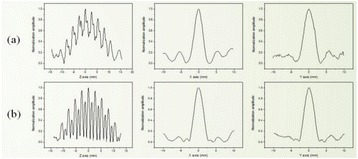
Sound field distribution of three axes of propagating wave focusing (a) and standing wave focusing (b)

**Fig. 2 (abstract P101).**
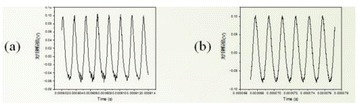
Acoustic pressure waveform of propagating wave (a) and standing wave (b) on the same focusing pressure (16MPa)

**Fig. 3 (abstract P101).**
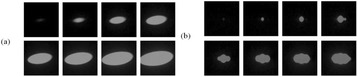
Recording the formation of lesion in phanton with high­speed camera; (a) the processes of lesion formation of the propagating wave focusing modalities; (b) the processes of lesion formation of focusing of standing wave

**Fig. 4 (abstract P101).**
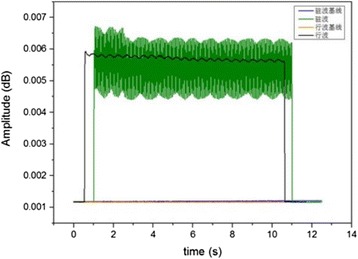
Cavitation signal of the propagating wave focusing modalities (the black curve) and the standing wave focusing modalities (the green curve) on the same focusingpressure (18MPa)

### P102 Feasibility of using nakagami distribution in structure characterization of the different lesions treated by HIFU

#### Meng Han, Na Wang, Mingxi Wan

##### Biomedical Engineering, Xi'an Jiaotong University, Xi'an, Shaanxi, China

###### **Correspondence:** Meng Han

**OBJECTIVES** High-intensity focused ultrasound (HIFU) is a noninvasive technique for tissue ablation and tissue erosion due to the thermal or mechanical effects. However, the microstructure of the treatment target has difference after treated by the different high-intensity focused ultrasound (HIFU) therapy mode. It is important to monitor the therapeutic effect. The Nakagami image was proposed to better visualize the tissue structure and scatter properties combined with the use of the B-modeimage simultaneously. The aim of this study was to explore the feasibility of using the Nakagami image for structure characterization of the lesions induced by focused ultrasound gradually from the ablation to tissue erosion.

**METHODS** Experiments were conducted on polyacrylamide phantoms with bovine serum albumin (BSA) using a single-element 1.6-MHz transducer to produce tissue ablation and tissue erosion. After the treatment, ultrasound B-mode images and corresponding RF data was recorded and the Nakagami images was presented after the RFdata was processed. B-mode image and Nakagami image were combined to visualize the tissue structure and scatter properties for structure characterization.

**RESULTS** For the B-mode images, the thermal lesion and the histotripsy both appeared to be hyperechoic in the region of the lesion (Fig. 1). In the Nakagami images, the thermal lesion appeared to be homogeneous blue, and has a clear boundary with the surrounding tissue. For the lesion treated by the histotripsy, it appeared blue in most of the lesion area, and there is an area of red in the center of the lesion. From the histogram of the M values of the lesion areas, we find that the values of the thermal lesion concentrated between 0.4 and 0.55 and the mean value is 0.47, while the values of the erosion treated by histotripsy distributed between 0.2 and 1.2 and the meanvalue is 0.40 (Fig. 2). From the results, we find that the mean values of the Nakagami parameter M for the thermal lesion and the mechanically homogenized lesion were different.

**CONCLUSIONS** The preliminary study on the tissue-mimicking phantom suggested that the Nakagami parameter M may have the potential possible to identify the lesions treated by HIFU of different modes and realize the structure characterization for the different HIFU lesions in the tissues.

**Fig. 1 (abstract P102).**
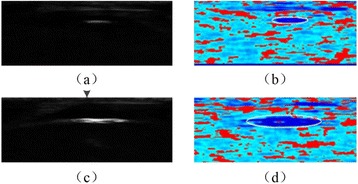
The images of the different HIFU lesions. (a) B­mode image of the thermal lesion; (b) Nakagami image of the thermal lesion; (c) B­mode image of the lesion byhistotripsy; (d) Nakagami image of the lesion by histotripsy

**Fig. 2 (abstract P102).**
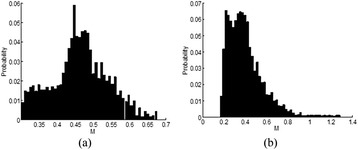
The histograms of the M values of the lesion area. (a) thermal lesion; (b) lesion by histotripsy

### P103 Dynamic modelling of piezoelectric hydrophones

#### Sun Yingqi, Yang Zengtao

##### Chongqing Medical University, Chongqing, China

###### **Correspondence:** Sun Yingqi

**OBJECTIVES** The conventional hydrostatic model based on off-resonance hydrophone design is incapable of analyzing the hydrophones operated around and upon the resonance frequencies. To expand the operating limits of the hydrostatic model.

**METHODS** A dynamic fluid–structure interaction model for analyzing the piezoelectric hydrophones is presented. An analytical solution for output voltage of the hydrophone operated in thickness-stretch vibration is derived.

**RESULTS** The output voltage is proportional to the acoustic pressure and the piezoelectric constants. The output voltage is also inversely proportional to the elastic stiffness and dielectric constants of the material, and the surface area of the hydrophone. Hence, a soft material with a higher-electromechanical coupling factor will produce higher output voltage. However, around the resonance range, the output voltage is seen to strongly depend on the operating frequencies of the hydrophone and the parameters of the fluid.

**CONCLUSIONS** The theoretical result shows that the output voltage obtained by dynamic model in the off-resonance range agrees well with those obtained by hydrostatic model. Furthermore, the dynamic model we present is sufficient to analyze the piezoelectric hydrophones over the entire frequency range, especially around the resonance, expanding the operating limits of hydrostatic model.

### P104 Experimental study of micro-bubble enhance low-frequency and low-intensity ultrasound-mediated plasmid transferring into mycobacterium smegmatis

#### Huimin Zheng

##### Chongqing Medical University, Chongqing, China

**OBJECTIVES** To investigate the synergistic effect of microbubble combined with low frequency and low intensity ultrasound (LFLIU) mediated plasmid transfer into mycobacterium.

**METHODS** A mixture of mycobacterium tuberculosis and plasmid were randomly divided into 3 groups: the control group, the ultrasonic irradiation group and the combination of microbubble and ultrasonic irradiation group. The transformation efficiency, the vitality of the bacteria and the expression of recombinant gene ClpP2 were measured respectively by colony-counting methods, flow cytometry methods, the Real-time quantitative polymerase chain reaction after the experiment processing.

**RESULTS** Positive colonies were selected on the LB agarose plate containing kanamycin after ultrasound exposure, the efficiency of the combination of microbubble andultrasonic irradiation group was 19.33×102CFU/ngDNA, which was more 19 times efficient than the ultrasonic group. The survival rate of the bacteria of ultrasonic irradiation group was 75.76%, which was more 2 times than the combination of microbubble and ultrasonic irradiation group. While the recombinant gene ClpP2 could be expressed, and there was no significant difference between the two groups (Fig. 1).

**CONCLUSIONS** Ultrasound microbubble could increase the efficiency of LFLIU mediated plasmid transfer to Mycobacterium tuberculosis. The results could provide experimental reference for LFLIU-mediated plasmid and DNA transformation into gram positive bacteria.

**Fig. 1 (abstract P104).**
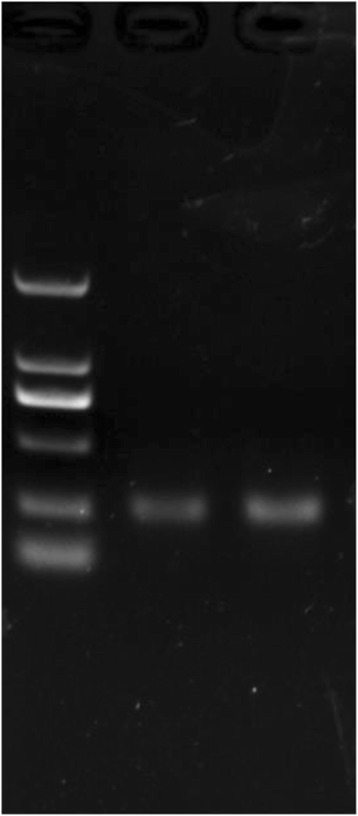
See text for description

### P105 Novel ultrasound contrast agent based on lipid containing sinapultide microbubbles for potential theranostics

#### Dong Liu^1,2^, Fang Yang^1,2^, Ning Gu^1,2^

#### ^1^School of Biological Science & Medical Engineering, Southeast University, Nanjing, China; ^2^State Key Laboratory of Bioelectronics and Jiangsu Key Laboratory forBiomaterials and Devices, Nanjing, China

##### **Correspondence:** Dong Liu

**OBJECTIVES** Since the bubbles were reported to have the effects of contrast enhancement, gas-filled bubbles used as ultrasonic contrast agents (UCAs) have been paid great attention [1, 2]. Apart from the usage of diagnostic agents, microbubbles have also widely used as drug and gene delivery vehicles [3]. Accordingly, the development of novel UCAs have become one of the most clinical potential fields in ultrasound medicine. In this study, we developed a novel lipid containing sinapultide microbubbles filled with sulfur hexafluoride (SF6). The good stability as well as appropriately size indicating that they have the potential to be used as ultrasound contrast agent in theranostics.

**METHODS** Stable microbubbles were fabricated by adding sinapultide to lipid mixture and then sonicating to generate microbubbles by cavitation from sulfur hexafluoride (SF6) in the mixture.

**RESULTS** The optimized preparation of sinapultide microbubbles had mean diameter of 1.82 ± 0.15μm and zeta potential of −55.2 ± 3.9 mV. The acoustic imaging analysis *in vitro* indicated that ultrasound imaging enhancement could be acquired by both perfusion imaging and accumulation imaging (Fig. 1).

**CONCLUSIONS** In summary, a novel lipid microbubbles encapsulated synthesized pulmonary surfactant sinapultide were fabricated, which makes a promising clinical potential for future theranostics of ultrasound imaging and therapy.

**Fig. 1 (abstract P105).**
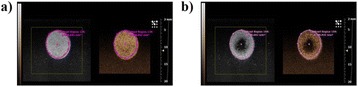
See text for description

### P106 Effects of focused ultrasound on expression of PAR2, SP and NK1-R in genital skin of SD rats with vulvar lichen simplex chronicus

#### Huan Yang, Huajun Tang, Yijin Fan, Chengzhi Li

##### College of Biomedical Engineering, Chongqing Medical University, Chongqing, China

###### **Correspondence:** Huan Yang

**OBJECTIVES** To explore the expression of protease activated receptor 2(PAR2), substance P(SP) and neurokinin-receptors in the genital skin of SD rats with vulvar lichen simplex chronicus(LSC) after focused ultrasound treatment.

**METHODS** Forty female rat models of LSC were established by Sally method, and then the rats were randomly divided into treatment group (n=20) and control group(n=20). The rats in the treatment group were treated by focused ultrasound, and those in the control group received sham treatment. After focused ultrasound intervention, the histological changes of vulva were observed by using HE staining. The total number and the degranulated number of mast cells were identified by staining slides with toluidine blue. Immunohistochemistry was used to test the expression of PAR2, SP and NK1-R. Differences among the groups were compared.

**RESULTS** After 2 weeks of focused ultrasound treatment, the rate of normal in treatment group was 75.00% (15/20) and that in control group was 10.00%(2/20),there was statistically significant between two groups (c2=17.289,P<0.05). The total number and the degranulated number of mast cells, the expression of PAR2, SPand NK1-R in treatment group were significantly lower than those in control group (all P<0.05).

**CONCLUSIONS** Focused ultrasound can treat LSC through inhibiting PAR2, SP and NK1-R expression, decreasing the total number and the degranulated number of mast cells and reducing the inflammatory reaction of tissues.

### P107 Feasibility study of the induction of a neuronal response in *L. Terrestris* with ultrasound stimulation

#### Jeremy Vion^1^, William Apoutou N'Djin^1^, Jean-Yves Chapelon^1^, Jahan Tavakkoli^2,3^

##### ^1^U1032, Inserm, Lyon, France; ^2^Physics, Ryerson University, Toronto, Ontario, Canada; ^3^Institute for Biomedical Engineering, Science and Technology, Toronto, Ontario, Canada

###### **Correspondence:** Jeremy Vion

**OBJECTIVES** Several works carried out these last years have demonstrated the ability of ultrasound (US) to activate neurons at a systemic level. However, no complete description of the biophysical mechanisms involved in this phenomenon has been validated so far. In order to identify experimentally the main mechanisms responsible for ultrasound neurostimulation, we here present an in-vivo study of the possible effects of US exposures on the generation of Compound Action Potentials (CAPs) in the common earthworm (*Lumbricus Terrestris*).

**METHODS** We chose to study in priority the effect of US radiation force, as proposed by Wahab et al. (2012), on a simple giant nerve model: the ventral nerve cord of L. terrestris. The system used was a geometrically focused piezoelectric US transducer (radius of curvature: 46 mm, aperture: 50 mm) working at the frequency of 0.55MHz. In each trial, a medial section of the anesthetized worm was placed within the US focal area (geometry: ellipsoidal, axial length: 29 mm at -3dB, axial width: 1.9mm at -3dB), before being exposed to US sequences similar to those proposed by Wright et al. (2015) on the peripheral crab nerve model (number of cycles: 25-28 cycles/pulse, PRF = 10 kHz, number of pulses: 80-200 pulses, peak-to-peak acoustic pressure: 4-14.4 MPa). The electrophysiological response of the nerve was monitored with two electrodes sunken through the animal in the vicinity of one end of the nerve. Before each trial, the functionality of the nerve was tested by applying anelectrical stimulation (pulsed, duration = 50 μs, amplitude = 5 V, PRF = 1 Hz, pulses number = 1 to 10) to the other end of the nerve. Complementarily, the response of the nerve to a mechanical stimulus applied to the superior end of the worm was recorded. The electrical and mechanical preliminary stimulations allowed repeatable observations of the CAP responses, which served as a reference for studying the nerve response to US exposures.

**RESULTS** The ultrasound sequences tested allowed inducing the generation of CAPs several times. The observed CAPs presented the same characteristics as those induced by the prior electrical and mechanical stimulations, as shown in Fig. 1. By computing the conduction velocity associated to the observed CAPs, we identifiedthe nervous structures stimulated to be the Medial Giant Fiber (MGF) and the Lateral Giant Fiber (LGF). Over the trials, both fibers were stimulated at least once butnever simultaneously. However, a great number of trials were performed, using various level of acoustic pressure, and this phenomenon appeared to be very littlerepeatable.

**CONCLUSIONS** Several hypotheses were considered to explain the low repeatability of our US neurostimulation results. First, it may be necessary to target specific areas in the giant nerve such as dorsal nodes, which could be performed in our future trials by using ultrasound guidance. Second, we cannot be sure that uncontrolled episodes of cavitation did not arise during some trials. This hypothesis is currently being tested in a series of trials where the ultrasound exposure is compatible with the controlled generation of cavitation. Finally, the myelin sheath embedding the giant nerve could also have an impact on the possibility of inducing mechanical effects on the neural membrane. Hence, we plan to adapt our set-up to the ventral nerve cord of the american lobster (Homarus Americanus). This unmyelinated nervous model has already been the object of preliminary studies performed during an international collaboration. In conclusion, we were able to demonstrate with this study the feasibility of inducing nervous responses with US and these responses were identical to those generated with electrical and mechanical stimulation. Further investigations are necessary to control this phenomenon and make it more repeatable. This project was supported by the Laboratory of Excellence (LabEx) DevWeCan and the MitacsGlobalink Research Award - Campus France.

**Fig. 1 (abstract P107).**
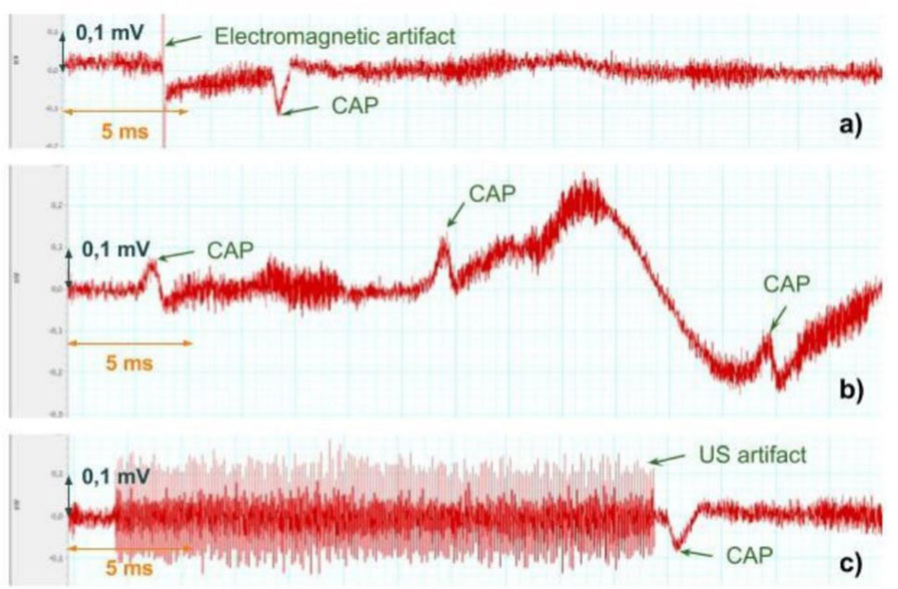
Examples of nervous responses to an electrical stimulus (a), a mechanical stimulus (b) and an ulstrasound stimulus (c)

### P108 Harmonic Motion Imaging (HMI) for pancreatic cancer detection and characterization in post-surgical human specimens

#### Thomas Payen^1^, Kenneth Olive^2^, Elisa E. Konofagou^1,3^

##### ^1^Biomedical Engineering, Columbia University, New York, New York, USA; ^2^Medicine and Pathology & Cell Biology, Columbia University Medical Center, New York, New York, USA; ^3^Radiology, Columbia University Medical Center, New York, New York, USA

###### **Correspondence:** Thomas Payen

**OBJECTIVES** Pancreatic ductal adenocarcinoma (PDA) has one of the lowest prognosis due to non-specific symptoms leading to late diagnosis and therapeutic inefficiency. PDA is characterized by an unusually dense stroma limiting chemotherapy perfusion. Harmonic Motion Imaging (HMI) assesses tissue mechanical properties by inducing localized oscillation resulting from a periodic acoustic radiation force. The amplitude of the induced displacement is directly related to the underlying tissue stiffness. The objective of this study was to evaluate the utility of HMI in post-surgical human pancreatic cancer specimens and its capability of differentiating the tumor for perilesional fibrotic tissue and normal tissue.

**METHODS** A 4.5-MHz focused ultrasound transducer (FUS) generates an amplitude-modulated beam resulting in harmonic tissue oscillations at its focus. Axial tissue displacement is estimated using 1D cross-correlation of RF signals acquired with a 2.5-MHz diagnostic transducer (P4-2, ATL) using a plane-wave beam sequence, confocally aligned with the FUS.Ten human pancreatic specimens (approximate dimensions = 24 x 12 x 80 mm3) were assessed immediately after surgery. They were immersed in PBS and their full volume was scanned for a maximum duration of 90 minutes. The imaging planes were chosen perpendicular to the pancreatic duct to correlate the HMI displacement withstandard histology analysis.

**RESULTS** Ten specimens of PDA were scanned using HMI (5 from distal pancreatectomy and 5 from Whipple procedure). Preliminary histology results showed that the scan did not damage the tissue in any aspect, and did not impact the subsequent analysis. HMI was able to scan the full volume of the specimens with measurements asdeep as 3 cm in the tissue. The whole range of stiffness from tumor to normal pancreas was assessed. The Fig. 1 shows the B-mode image of the maximal cross-section of the tumor, and the HMI map overlaid on the B-mode image. The mean displacement inside the tumor is 1.41 ± 1.00 μm versus 11.25 ± 3.69 μm in the surrounding tissue. Measurements in normal pancreatic tissue demonstrated a mean HMI displacement of 17.05 ± 3.98μm. A sharp mechanical contrast is evident on the HMI maps between the tumor and the surrounding tissue which correlates very well with the B-mode image.

**CONCLUSIONS** This initial feasibility study showed that HMI is capable of scanning whole resected pancreatic cancer specimens without any damage to the tissue. The technique can assess deep parts of the tissue with greatly difference stiffness in a limited amount of time. HMI significantly detected PDA within the specimen with mean displacement lower by a ratio of 9.8 compared to the surrounding tissue and 12.1 compared to normal pancreas away from the mass. This indicates the possible presence of fibrotic tissue close to the tumor. The sharp tumor delineation observed on HMI maps was confirmed on the B-mode image.HMI is thus shown promising for pancreatic cancer detection and characterization. The technique can provide images of the entire organ and preliminary results indicate that it can distinguish PDA, fibrotic tissue from normal pancreatic tissue. HMI could thus constitute a very important tool for screening patients at risk of developing pancreatic cancer and showing the non-specific symptoms.

**Fig. 1 (abstract P108).**
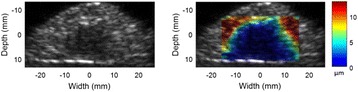
Pancreatic ductal adenocarcinoma in a distal pancreatectomy specimen. The B­mode image (top) is shown as well as the overlaid HMI displacement map (bottom)

### P109 Experimental study on the influence of low-frequency and low-intensity ultrasound on the permeability of the mycobacterium smegmatis cytoderm and potentiation with levofloxacin

#### Yu Dong^2^, Hang Su^3^, Yonghong Du^2^, Dairong Li^1^

##### ^1^Department of Respiratory disease, The First Affiliated Hospital of Chongqing Medical University, Chongqing, China; ^2^Chongqing medical university, Chongqing, China; ^3^Food and Drug Administration of Huiji, Zhengzhou, China

###### **Correspondence:** Yu Dong

**OBJECTIVES** Tuberculosis (TB) is an infectious disease caused by the bacterium Mycobacterium tuberculosis. The aim of this study is to investigate the synergistic potentiating effect of low-frequency and low-intensity ultrasound (LFLIUS) with levofloxacin on M. smegmatis (MS) [a "surrogate of MTB"] and to explore underlying mechanisms.

**METHODS** M. smegmatis was continuously irradiated by ultrasound of 42 kHz with several different doses (intensity and duration), respectively. The bacteria vitality, structure and morphology were subjected to plate counting and microscopic examinations. Flow cytometry was adopted to test the fluorescence intensity of bacteria that were stained by FAD, or PI dye. Spatially and temporally averaged intensity (ISATA) of 0.138 W/cm^2^ was then acted on mycobacterium smegmatis combined with levofloxacin to confirm the synergy between ultrasound and antibiotic.

**RESULTS** The results showed that the permeability of M. smegmatis increased after ultrasound exposure; and the survival rate, structure and morphology of bacteria of the low-dose (ISATA= 0.138 W/cm^2^ for 5 min) treated ultrasound group had no evident difference compared with those of the control group (P>0.05) ; but the survival rate of bacteria significantly decreased and the bacteria structure got serious damage in high-dose (ISATA= 0.329 W/cm^2^ for 20 min) ultrasound group. The bactericidal effect of ultrasound combining with levofloxacin was evidently higher than that of single ultrasound or single levofloxacin.

**CONCLUSIONS** A certain dose of LFLIUS can increase the permeability of M. smegmatis’ cytoderm and exert a synergy to bactericidal action of levofloxacin.

### P110 Intracellular calcium response of hela cells stimulated by the impulsive jetting flow from tandem bubble interaction

#### Fenfang Li^1^, Chen Yang^1^, Fang Yuan^2^, Defei Liao^1^, Guilak Farshid^3^, Pei Zhong^1^

##### ^1^Mechanical Engineering and Materials Science, Duke University, Durham, North Carolina, USA; ^2^HuaCells Corporation, Natick, Massachusetts, USA; ^3^Department of Orthopaedic Surgery, Washington University, St. Louis, Missouri, USA

###### **Correspondence:** Fenfang Li

**OBJECTIVES** Cavitation plays an important role in the bioeffects produced by therapeutic ultrasound. However, limited studies have been carried out to investigate the mechanisms of cellular mechanotransduction that are activated by bubble pulsations in a controlled manner, especially at the single cell level. Mechanotransduction is a process through which cells sense and respond to mechanical stimuli (e.g., membrane tension and strain) and convert them to biochemical signals. In many cases, the initial mechanotransduction signal involves an increase in intracellular calcium ion (Ca2+) elicited by mechanical stress, which subsequently triggers various downstream biochemical reactions that may alter the morphology and function of the cell. In this study, we examine the intracellular calcium response of single adherent HeLa cells evoked by the directional jetting flow from tandem bubbles (Rmax = 50 ± 2 μm).

**METHODS** Bubble dynamics, morphology, location, and adhesion conditions of individual cells were well-controlled in a microfluidic system with surface patterning. Two types of calcium responses, in the form of intracellular calcium waves (ICW), were observed at different standoff distance (Sd = 30 to 60 μm) of the tandem bubbleto the cell through time-elapsed fluorescence imaging.

**RESULTS** The ICW was initiated from the leading edge facing the jetting flow, and propagated toward the trailing edge of the cell. At Sd/Rmax = 0.6, a fast calcium response with short rise time and large amplitude in calcium concentration change was produced. In contrast, at Sd/Rmax = 1, a slow calcium response with long risetime and small amplitude was often observed. In all cases, the calcium response was initiated by the influx of extracellular calcium through the membrane by either poration produced predominately at Sd/Rmax = 0.6 or ion channel opening induced primarily at Sd/Rmax = 1.0, which were confirmed by mechanistic studies using calcium-free extracellular medium, membrane poration indicator propidium iodide, or the non-specific mechanosensitive ion channel blocker Gd3+. The elicited calcium transient propagated in the cytosol through calcium-induced calcium release (CICR) from endoplasmic reticulum storage, which was inhibited by thapsigargin. We further demonstrated that attaching 6 μm integrin-binding beads to the cell membrane can trigger calcium response under conditions (Sd/Rmax = 1.2) that do not elicit such a response by the tandem bubbles without the beads.

**CONCLUSIONS** These findings show that directional jetting flow from tandem bubbles can be used to induce highly controlled mechanical stimulation on individual cells.

### P111 The safety on subsequent pregnancy in women after ultrasound ablation of uterine fibroids: a single-central retrospective study

#### Junshu Li, Wenzhi Chen, Jinyun Chen

##### The State Key Laboratory of Ultrasound Engineering in Medicine Co-Founded by Chongqing and the Ministry of Science and Technology, Chongqing Key Laboratory ofBiomedical Engineering, College of Biomedical Engineering, Chongqing Medical University, Chongqing Collaborative Innovation Center for Minimally-invasive andNoninvasive Medicine, ChongQing, China

###### **Correspondence:** Junshu Li

**OBJECTIVES** A retrospective analysis to explore the impact of high-intensity focused ultrasound (HIFU) ablation of uterine fibroids in women on subsequent pregnancy.

**METHODS** A retrospective analysis was conducted of women with uterine fibroids who underwent HIFU ablation at the Clinical Center for Tumor Therapy, Chongqing Medical University, Chongqing, China, from January 1, 2010, to January 1, 2015. Inclusion criteria were: (1) aged 20-42 years (2) with fertility desire (3) have normal sexual life without contraception after HIFU. Exclusion criteria were: fertility barriers unrelated to HIFU, such as hysterectomy or bilateral oophorectomy. The registry of patient cases were screened by marital status and birth history before treatment, and telephone follow-up information including improvement of symptoms of uterine fibroids, sexual life, pregnancy, and delivery information after HIFU treatment.

**RESULTS** A total of 189 cases met inclusion criteria and followed-up effectively, the median follow-up time was three years. Majority of them have symptoms of uterine fibroids improve significantly after HIFU treatment, and volume of uterine fibroids decrease(P<0.05). Among the 189 cases, there were 131 cases pregnancy with a total of 133 times.Of 131 pregnant women,19 were on-going pregnancy, 21 cases of abortion, the remaining 91 cases in successful pregnancies and deliveries.The incidence rates of complications during pregnancy and labor were 10.8% (10/93) and 7.5% (7/93), respectively.The most common complications of pregnancy in this study were placenta previa and placenta implantation(5/10).The incidence rate of massive hemorrhage during labor was 6.5%(6/93),the majority (66.7%) were associated with resection intramural fibroid during cesarean section.The study presented here did not contain any cases of uterine rupture during pregnancy or labor after HIFUtreatment. Ninety-one women successfully delivered 93 times with a cesarean section rate of 72.0%(67/93). Among them,only fourteen women (20.9%) chose delivery by cesarean section for obstetric factors, while 79.1% chose delivery by cesarean section for social factors.

**CONCLUSIONS** In conclusion, HIFU is a safe and effective noninvasive therapy to treat uterine fibroids in women who wish to retain the ability to conceive and deliver after treatment. Additionally, HIFU effectively provides remission of symptoms, has a low rate of complications through pregnancy and labor, and does not increase the rate of spontaneous abortion or delivery by cesarean section. Based on our findings presented here, we believe that HIFU should be recommended treatment for uterine fibroids in women who wish to retain the ability to have children in the future, or who otherwise wish to not undergo hysterectomy.

### P112 Low intensity pulsed ultrasound relieves myelosuppression induced by cyclophosphamide in rabbits

#### B. Liu

##### Chongqing Medical University, Chongqing, China

**OBJECTIVES** This study is to investigate the effect of low intensity pulsed ultrasound (LIPUS) on cyclophosphamide (CTX)-induced rabbit myelosuppression.

**METHODS** A total of 40 New Zealand white rabbits were used to establish the myelosuppression models by daily ear vein injection of 40 mg/kg CTX for 4 continuous days. They were randomly divided into LIPUS group and control group. LIPUS was performed once a day for 20 minutes each time for 7 and 28 days, respectively. The treatment effects of LIPUS were examined by comparing general conditions, mortality rates, blood routine and bone marrow hyperplasia in bone marrow smears. The safety of the LIPUS irradiation was evaluated by HE staining.

**RESULTS** LIPUS improved the number of peripheral blood cells and bone marrow nucleated cells and thus reduced the mortality of model rabbits in different degrees. The irradiated parts of the skin did not burn, and the muscle tissue in acoustic channels showed normal structures and no obvious pathological changes, suggesting LIPUS irradiation is safe for the model rabbits.

**CONCLUSIONS** LIPUS is a safe and effective method to relieve CTX-induced myelosuppression, which can be used for the prevention and treatment of the occurrence and development of myelosuppression.

### P113 Cost-effectiveness evaluated by markov decision-making tree model of high-intensity focused ultrasound ablation versus surgery for uterine fibroids

#### Liang Hu, Jinyun Chen, Yujie Feng, Chang Liu, Wenzhi Chen, Zhibiao Wang

##### College of Biomedical Engineering, Chongqing Medical University, Chongqing, China

###### **Correspondence:** Liang Hu

**OBJECTIVES** To investigate the cost-effectiveness of high-intensity focused ultrasound (HIFU) ablation versus surgery (myomectomy and hysterctomy) for uterinefibroids.

**METHODS** Direct medical cost, indirect cost, efficacy and safety outcomes of patients with uterine fibroids who ever underwent HIFU, myomectomy, and hysterectomywere collected to build Markov Decision-making Tree Model based on health status of these patients. The Model was employed to make simulation of health status transition for uterine fibroids patients treated by these different procedures. Cost-effectiveness, cost-utility as well as sensitivity analysis were conducted for evaluation ofHIFU, myomectomy and hysterectomy for uterine fibroids.

**RESULTS** The Markov Decision-making Tree Model simulation showed that each unit improvement of physical summary scale (PCS) and mental summary scale (MCS)related to HIFU ablation consumed 1782.16 and 1120.88 respectively. By contrast, each unit improvement of PCS and MCS related to surgery consumed 1825.43 and 1605.15 respectively.

**CONCLUSIONS** The Markov Decision-making Tree Model simulation and healthy economic analysis demonstrate HIFU is dominatingly cost-effective for uterinefibroids.

### P114 Mechanisms of plasmid DNA intracellular uptake facilitated by ultrasound and targeted microbubbles is determined by bubble-cell interaction

#### N. Rong^1^, H. Zhou^2^, R. Liu^2^, Z. FAN^1^

##### ^1^Biomedical Engineering, Tianjin University, Tianjin, Tianjin, China; ^2^College of Life Sciences, Nankai University, Tianjin, Tianjin, China

###### **Correspondence:** N. Rong

**OBJECTIVES** Therapeutic Ultrasound combined with microbubbles can achieve non-viral, targeted delivery of genetic materials, as a promising delivery technique for gene therapy. However, insufficient understanding of plasmid DNA intracellular uptake process and the important role of microbubble plays during this process, limits the progress of improving gene transfection efficiency and translating this technique into clinics. Therefore, the aim of this study is to investigate the intracellular uptake mechanisms of plasmid DNA mediated by targeted microbubbles driven by different ultrasound conditions.

**METHODS** Targestar-SA microbubbles conjugated with RGD ligands were used to attach on the cell membrane of HeLa cells. A Plasmid encoding for green fluorescent protein (GFP) was used for gene transfection. Two ultrasound conditions were selected to stimulate two typical bubble activities while achieving good transfection efficiency and maintaining high cell viability, high pressure short pulse (0.6Mpa, 10μs) and low pressure long pulse (0.2MPa, 1ms). Multiply approaches were employed to investigate bubble dynamics, cell membrane responses and plasmid DNA internalization routes: (i) Fast frame video microsocopy (Photron FASTCAM SA2) was used to record the microbubble dynamics. (ii) Real-time fluorescent microscopy of propidium iodide to indicate and characterize pores. (iii) Confocal fluorescent microscopy of plasmid DNA (Cy3 labled), cell membrane (Alexa Fluor 647 labled), and nuclear (Hoechst 33342 labeled) (Fig. 1). And (iv) SEM images for the surface topography of cell membrane. All these observations were spatiotemporally correlated.

**RESULTS** (i) When stimulated by a high pressure short pulse (0.6Mpa, 10μs), targeted microbubble generates a transient and reversible pore on the cell membrane, with radius varies from several microns to several hundreds microns. While driven by low pressure long pulse (0.2MPa, 1ms), the microbubbles undergo gentle oscillation, unlikely to perforate cell membrane. (ii) When driven by high pressure short pulse, plasmid DNA can be directly and promptly propelled into the cytosol, even nuclear, through pores with the diameter larger than about 100 microns. (iii) When driven by low pressure long pulse, the gentle bubble-cell interaction significantly enhanced the plasmid DNA aggregation and retention onto the cell membrane, while the global endocytosis is responsible for plasmid DNA internalization. (iv) The determinant factor for different plasmid DNA uptake processes is the ultrasound driven bubble- cell interaction.

**CONCLUSIONS** By establishing direct spatiotemporal correlation between ultrasound-stimulated targeted microbubbles activities and the resulting intracellular plasmid DNA uptake process, this study revealed the complex and bubble-cell interaction dependent nature of plasmid DNA uptake mechanisms at single cell level.

**Fig. 1 (abstract P114).**
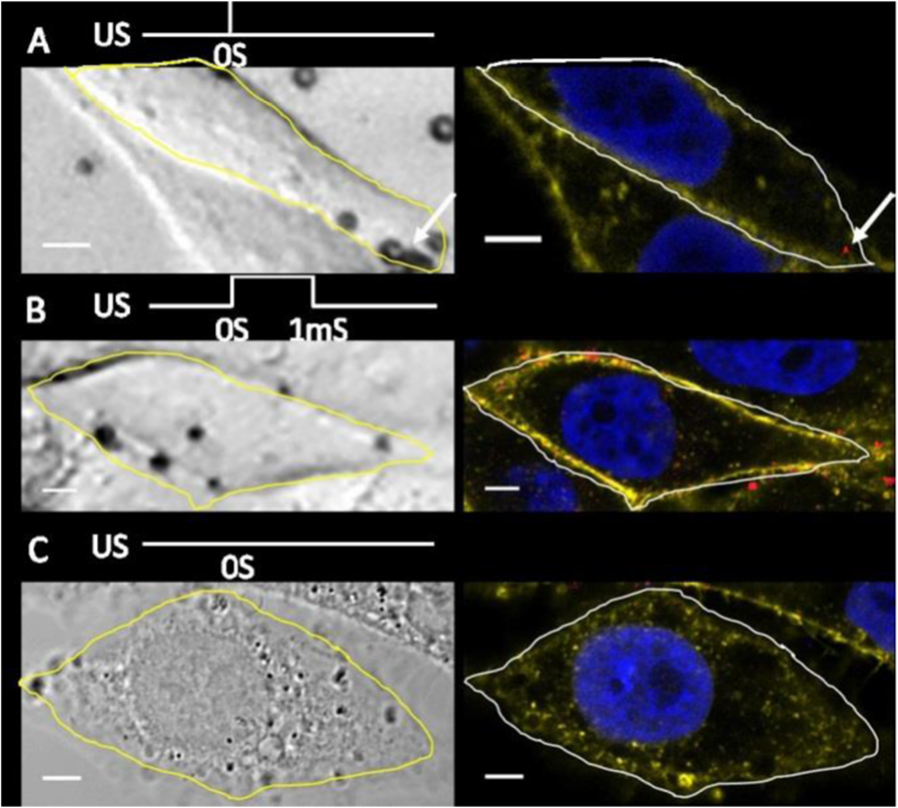
Cy3 labeled plasmid DNA distribution in Hela cells of different ultrasound parameter. Bright field images showing bubble information and representative confocal images showing pDNA distribution with Cy3 labeled pDNA (red), cell nuclei (blue) and membrane(yellow).(A) 0.6MPa,10μs, (B) 0.2MPa,1ms,(C) Control without ultrasound stimulation.Scale bar is 5um

### P115 Optimization of tumour therapy by combination of focused ultrasound and radiation guided by MRI and PET-MRI

#### Doudou Xu^1^, Lisa Landgraf^1^, Xinrui Zhang^1^, Michael Unger^1^, Ina Patties^2^, Johann Berger^1^, Shaonan Hu^1^, Lydia Koi^3,4^, Antje Dietrich^3,5^, Aswin Hoffmann^3,4^, Mechthild Krause^3,4^, Thomas Neumuth^1^, Andreas Melzer^1^

##### ^1^Innovation Center Computer Assisted Surgery, Universität Leipzig, Leipzig, Germany; ^2^Department of Radiation Therapy, Universität Leipzig, Leipzig, Germany; ^3^OncoRay - National Center for Radiation Research in Oncology, Dresden, Germany; ^4^Department of Radiation Oncology, University Hospital Carl Gustav Carus, Technische Universität Dresden, Dresden, Germany; ^5^German Cancer Consortium (DKTK), partner site Dresden, German Cancer Research Center (DKFZ), Heidelberg, Germany

###### **Correspondence:** Doudou Xu

**Introduction**

The two ZIK-Centers for Innovation Competence, ICCAS in Leipzig and OncoRay in Dresden, have joined forces to start a new multidisciplinary 6.3 million Euro research project: SONO-RAY - Tumor therapy combining image-guided (PET-MR and MR) focused ultrasound and radiation therapy. The goal of SONO-RAY is to combine noninvasive image-guided therapy approaches of magnetic resonance guided focused ultrasound and radiation therapy to improve the efficacy of cancer treatment.

**Hypothesis and Motivations:**

SONO-RAY project aims to combine therapeutic focused ultrasound (FUS) and radiation therapy (RT) to treat malignant solid tumors and tumor metastases. The hypothesis underlying this approach is that the combination of two tissue-destroying energies (the energy of high-intensity acoustic waves and ionizing radiation) is more effective in cancer treatment than the effect of employing one of the above two energy forms alone [1-3]. The central scope of the project is to develop tumor cell biology fundamentals, the computer-aided model formation and to evaluate the success potential of a future clinical use of a FUS-RT combination therapy. Within the framework of this project, the basic principle of the combined FUS and RT effect on tumor cells (*in vitro*) and the tumor tissue (*in vivo*) is going to be investigated preclinically. Clinical applications could be the treatment of various tumors of the head and neck, prostate carcinoma and glioblastoma.

**Project work package:**

In order to provide the basis for the combined FUS-RT method, *in vitro* and *in vivo* experiments are being be carried out to elaborate the thermal and mechanical effect on the tumor tissue [4]. After identification of the thermal and mechanical individual parameters for FUS, these are transferred into simulation models in order to predict the behavior of the fabric on FUS[5]. The simulation models are validated using the parameters. Based on the data obtained above, FUS and RT will be generated spatially dispersed biologically active doses. Algorithms will be used for the anatomical co-registration and accumulation of the biologically active dose distributions generated by FUS and RT on MR images. Furthermore, software modules will be developed and validated, which support the clinical user in therapy planning. Optimization of application sequences of the FUS-RT technology and the incorporation in therapy into the treatment process (clinical workflow) of the patient will also be investigated and developed.

**Experimental facilities in SonoRay**

**In vitro FUS and RT:** A high throughput *in vitro* 96-well sonicator was designed and allows individual sonication for each of the wells in a 96-well plate. It consists of 96 single transducers at an operating frequency of 1 MHz and a maximum energy of 0.05 W/cm^2^. A 150 kV X-ray machine (DARPAC 150-MC) was employed for irradiation at doses of 0 – 20 Gy. The analysis was conducted by using three different cell lines for prostate cancer (PC-3, Vcap, LNcap), glioblastoma (LN405, U87, T98G) and head/neck tumor (FaDu, UT-SCC 5, UT-SCC 8). Effects at the cellular level on metabolism (WST-1), proliferation (BrdU), membrane integrity (LDH release) and apoptosis (Annexin V) were detected after treatment.

**In vivo:** PET-MR and MR guided focused ultrasound system allows precise sonication treatment for small animals bearing tumors, under real-time MR-thermometry [6]. Small animal PET-MR system (nanoScan, Mediso) will be employed and integrated with a FUS transducer (11×11 matrix array), which allows the function of beam forming to achieve hyperthermia treatment. Local tumor irradiations under normal blood flow conditions will then be given with 200 kV X-rays (0.5 mm copper -filter) and 20 mA at a dose rate of ~ 1.1 Gy/min (X-ray machine type Yxlon Y.TU 320-D03) [7]. The self-filtering of the X-ray tube is 3 mm beryllium and 3 mm aluminum. Nude mice bearing heterotopic tumors on hind leg will be treated by both FUS and RT in sequence. Tumor size monitoring, histology study and UPLC molecular analysis will be investigated post the combination therapy.

**Robot installed in PET-MR:** A MR-compatible robotic arm system (INNOMOTION^TM^, Innomedic) [8, 9] was installed with Biograph mMR MR-PET (Siemens Healthineers) in the Department of Nuclear Medicine of the University Medical Center Leipzig to investigate the effects of a combination of focused ultrasound and radiation therapy. The robotic arm will reposition the ultrasound transducer during the sonication treatment. It is possible to detect residual tumor tissue after the treatment by using PET-MR imaging to provide an optimal treatment outcome.

**MR guided FUS-Sonalleve:** MR-guided high-intensity focused ultrasound (MR-HIFU) is a noninvasive technique for depositing thermal energy in a controlled manner deep within the body. The Philips Sonalleve MR-HIFU system, initially released in 2010, was installed in Leipzig University Hospital at the beginning of 2017, introduced a new approach for uterine fibroids[10, 11] and bone metastasis treatment [12, 13] by employing thermal ablation. Sonalleve system also offers solutions of hyperthermia research platform [14, 15] in combination with radiation therapy and chemotherapy in cancer treatment.

**MR guided FUS-prostate system:** The TULSA-PRO System (Profound Medical) is a transurethral MR guided FUS system for whole gland ablation of the prostate [16]. A test system was installed at the university hospital Dresden to perform the world’s first compatibility tests on a Philips Ingenuity TF PET/MR scanner. The system comprises a transurethral ultrasound applicator with 10 FUS elements working at 4 or 14 MHz, depending on the penetration depth required to heat the rim of the prostate (i.e. the so-called “thermal ablation boundary”) up to 55 deg Celcius. The system has an endorectal cooling device to prevent the rectal mucosa from overheating. The ultrasound applicator also has a cooling circuit to spare the urethra. The power and frequency of the 10 ultrasound transducers are individually steered by real-time MR-thermometry.

**References**

[1] R. Cirincione, F.M. Di Maggio, G.I. Forte, L. Minafra, V. Bravata, L. Castiglia, V. Cavalieri, G. Borasi, G. Russo, D. Lio, C. Messa, M.C. Gilardi, F.P. Cammarata, High-Intensity Focused Ultrasound- and Radiation Therapy-Induced Immuno-Modulation: Comparison and Potential Opportunities, Ultrasound in medicine & biology, 43 (2017) 398-411.

[2] T. Refaat, S. Sachdev, V. Sathiaseelan, I. Helenowski, S. Abdelmoneim, M.C. Pierce, G. Woloschak, W. Small, Jr., B. Mittal, K.D. Kiel, Hyperthermia and radiation therapy for locally advanced or recurrent breast cancer, Breast, 24 (2015) 418-425.

[3] S.K. Wu, C.F. Chiang, Y.H. Hsu, H.C. Liou, W.M. Fu, W.L. Lin, Pulsed-wave low-dose ultrasound hyperthermia selectively enhances nanodrug delivery and improves antitumor efficacy for brain metastasis of breast cancer, Ultrasonics sonochemistry, 36 (2017) 198-205.

[4] J. Beik, Z. Abed, F.S. Ghoreishi, S. Hosseini-Nami, S. Mehrzadi, A. Shakeri-Zadeh, S.K. Kamrava, Nanotechnology in hyperthermia cancer therapy: From fundamental principles to advanced applications, Journal of controlled release : official journal of the Controlled Release Society, 235 (2016) 205-221.

[5] J. Hu, Y. Ding, S. Qian, X. Tang, Simulations of adaptive temperature control with self-focused hyperthermia system for tumor treatment, Ultrasonics, 53 (2013) 171-177.

[6] A.J. Loeve, J. Al-Issawi, F. Fernandez-Gutierrez, T. Lango, J. Strehlow, S. Haase, M. Matzko, A. Napoli, A. Melzer, J. Dankelman, Workflow and intervention times of MR-guided focused ultrasound - Predicting the impact of new techniques, Journal of biomedical informatics, 60 (2016) 38-48.

[7] A. Yaromina, T. Kroeber, A. Meinzer, S. Boeke, H. Thames, M. Baumann, D. Zips, Exploratory study of the prognostic value of microenvironmental parameters during fractionated irradiation in human squamous cell carcinoma xenografts, International journal of radiation oncology, biology, physics, 80 (2011) 1205-1213.

[8] A.J. Krafft, J.W. Jenne, F. Maier, R.J. Stafford, P.E. Huber, W. Semmler, M. Bock, A long arm for ultrasound: a combined robotic focused ultrasound setup for magnetic resonance-guided focused ultrasound surgery, Medical physics, 37 (2010) 2380-2393.

[9] N.V. Tsekos, A. Khanicheh, E. Christoforou, C. Mavroidis, Magnetic resonance-compatible robotic and mechatronics systems for image-guided interventions and rehabilitation: a review study, Annual review of biomedical engineering, 9 (2007) 351-387.

[10] Y.S. Kim, B. Keserci, A. Partanen, H. Rhim, H.K. Lim, M.J. Park, M.O. Kohler, Volumetric MR-HIFU ablation of uterine fibroids: role of treatment cell size in the improvement of energy efficiency, European journal of radiology, 81 (2012) 3652-3659.

[11] M.J. Voogt, H. Trillaud, Y.S. Kim, W.P. Mali, J. Barkhausen, L.W. Bartels, R. Deckers, N. Frulio, H. Rhim, H.K. Lim, T. Eckey, H.J. Nieminen, C. Mougenot, B. Keserci, J. Soini, T. Vaara, M.O. Kohler, S. Sokka, M.A. van den Bosch, Volumetric feedback ablation of uterine fibroids using magnetic resonance-guided high intensity focused ultrasound therapy, European radiology, 22 (2012) 411-417.

[12] M. Huisman, M.K. Lam, L.W. Bartels, R.J. Nijenhuis, C.T. Moonen, F.M. Knuttel, H.M. Verkooijen, M. van Vulpen, M.A. van den Bosch, Feasibility of volumetric MRI-guided high intensity focused ultrasound (MR-HIFU) for painful bone metastases, Journal of therapeutic ultrasound, 2 (2014) 16.

[13] M.K. Lam, M. Huisman, R.J. Nijenhuis, M.A. van den Bosch, M.A. Viergever, C.T. Moonen, L.W. Bartels, Quality of MR thermometry during palliative MR-guided high-intensity focused ultrasound (MR-HIFU) treatment of bone metastases, Journal of therapeutic ultrasound, 3 (2015) 5.

[14] E.J. Dorenberg, F. Courivaud, E. Ring, K. Hald, J.A. Jakobsen, E. Fosse, P.K. Hol, Volumetric ablation of uterine fibroids using Sonalleve high-intensity focused ultrasound in a 3 Tesla scanner--first clinical assessment, Minimally invasive therapy & allied technologies : MITAT : official journal of the Society for Minimally Invasive Therapy, 22 (2013) 73-79.

[15] N.M. Hijnen, E. Heijman, M.O. Kohler, M. Ylihautala, G.J. Ehnholm, A.W. Simonetti, H. Grull, Tumour hyperthermia and ablation in rats using a clinical MR-HIFU system equipped with a dedicated small animal set-up, International journal of hyperthermia : the official journal of European Society for Hyperthermic Oncology, North American Hyperthermia Group, 28 (2012) 141-155.

[16] M. Burtnyk, T. Hill, H. Cadieux-Pitre, I. Welch, Magnetic resonance image guided transurethral ultrasound prostate ablation: a preclinical safety and feasibility study with 28-day followup, The Journal of urology, 193 (2015) 1669-1675.

### P116 Combination therapy for cancer by PET-MR and MR image guided focused ultrasound and radiation

#### Doudou Xu^1^, Lisa Landgraf^1^, Xinrui Zhang^1^, Michael Unger^1^, Ina Patties^2^, Johann Berger^1^, Shaonan Hu^1^, Lydia Koi^3,4^, Marc Fournelle^5^, Steffen Tretbar^5^, Thomas Neumuth^1^, Andreas Melzer^1^

##### ^1^Innovation Center Computer Assisted Surgery, Universität Leipzig, Leipzig, Germany; ^2^ Department of Radiation Therapy, Universität Leipzig, Leipzig, Germany; ^3^ OncoRay - National Center for Radiation Research in Oncology, Dresden, Germany; ^4^Department of Radiation Oncology, University Hospital Carl Gustav Carus, Technische Universität Dresden, Dresden, Germany; ^5^Fraunhofer IBMT, St. Ingbert, Germany

###### **Correspondence:** Doudou Xu

**INTRODUCTION** FUS-RT stands for the combination therapy of focused ultrasound (FUS) and radiation therapy (RT). The aim of SONORAY project is to use the synergistic effect of heat and mechanical effects of sound as well as ionizing radiation in order to improve the results of radiooncological treatments of malignant solid tumors and metastases. Physical and biological effects are being investigated and the synergy of FUS and RT will be quantified in simulation, cell, and small animal studies. The work is accompanied by the development of multimodal treatment planning and information systems to prepare for a seamless integration into the clinical workflow. The project aims at developing a proof-of-concept system and workflow for the translation into clinical use.

**METHODS** A high throughput *in vitro* sonicator with 1.14 MHz single transducer made by piezoelectric ceramic material was employed and allows individual sonication for wells in a 96-well plate. T98G glioma cells were exposure to acoustic intensity of 71 W/cm^2^ with hyperthermia (40-45°C) duration of 134 sec, and 109 W/cm^2^ with hyperthermia (40-45°C) duration of 65 sec, respectively. A 150 kV X-ray machine (DARPAC 150-MC) was employed for irradiation at doses of 0-20 Gy to investigate the radiation curve of T98G cells. For FUS and RT combinations, 5 and 10 Gy were used to treat T98G cells 24hrs post sonication. Effects at the cellular level on metabolism (WST-1), proliferation (BrdU) and membrane integrity (LDH release) were detected after treatment.

**RESULTS** The preliminary RT results showed dose dependent loss in cellular NAD(P)H levels of 60% for T98G cell lines at 20 Gy. A release of LDH was observed from 4% (0 Gy) to 17% (20 Gy). The highest impact of RT was detected during analysis of DNA synthesis (BrdU) which nearly stopped at dosages above 5 Gy. In terms of the first FUS and RT combination experiment, there is no higher LDH release in the combination therapy group in comparison to FUS or RT single treatment groups. In contrast, cells treated by combination of FUS hyperthermia at 40-45°C for 65 secs with 10 Gy irradiation treatment suggested lower cell viability of 81% (WST-1 assay) in comparison to treatment of FUS hyperthermia only (97% viable cells) and RT only (90% viable cells).

**CONCLUSIONS** In conclusion, longer FUS hyperthermia duration from 100 sec to 1000 sec should be further investigated in combination with RT treatment. The interval between FUS hyperthermia and irradiation treatment will be conducted within 8hrs according to literatures. A fitted treatment for different cell lines will be necessary based on the different radiosensitivities. Future *in vitro* investigations of effects of FUS hyperthermia as well as of a combined therapy on other tumor cells need to be conducted.

